# More P450s Are Involved in Secondary Metabolite Biosynthesis in *Streptomyces* Compared to *Bacillus*, *Cyanobacteria*, and *Mycobacterium*

**DOI:** 10.3390/ijms21134814

**Published:** 2020-07-07

**Authors:** Fanele Cabangile Mnguni, Tiara Padayachee, Wanping Chen, Dominik Gront, Jae-Hyuk Yu, David R. Nelson, Khajamohiddin Syed

**Affiliations:** 1 Department of Biochemistry and Microbiology, Faculty of Science and Agriculture, University of Zululand, KwaDlangezwa 3886, South Africa; fanelemngun@gmail.com (F.C.M.); teez07padayachee@gmail.com (T.P.); 2 Department of Molecular Microbiology and Genetics, University of Göttingen, 37077 Göttingen, Germany; chenwanping1@foxmail.com; 3 Faculty of Chemistry, Biological and Chemical Research Center, University of Warsaw, Pasteura 1, 02-093 Warsaw, Poland; dgront@gmail.com; 4 Department of Bacteriology, University of Wisconsin-Madison, 3155 MSB, 1550 Linden Drive, Madison, WI 53706, USA; jyu1@wisc.edu; 5 Department of Systems Biotechnology, Konkuk University, Seoul 05029, Korea; 6 Department of Microbiology, Immunology and Biochemistry, University of Tennessee Health Science Center, Memphis, TN 38163, USA

**Keywords:** *Streptomyces*, *Mycobacterium*, *Bacillus*, *Cyanobacteria*, cytochrome P450 monooxygenases, secondary metabolites, biosynthetic gene clusters, terpenes, polyketides, P450 blooming, non-ribosomal peptides

## Abstract

Unraveling the role of cytochrome P450 monooxygenases (CYPs/P450s), heme-thiolate proteins present in living and non-living entities, in secondary metabolite synthesis is gaining momentum. In this direction, in this study, we analyzed the genomes of 203 *Streptomyces* species for P450s and unraveled their association with secondary metabolism. Our analyses revealed the presence of 5460 P450s, grouped into 253 families and 698 subfamilies. The CYP107 family was found to be conserved and highly populated in *Streptomyces* and *Bacillus* species, indicating its key role in the synthesis of secondary metabolites. *Streptomyces* species had a higher number of P450s than *Bacillus* and cyanobacterial species. The average number of secondary metabolite biosynthetic gene clusters (BGCs) and the number of P450s located in BGCs were higher in *Streptomyces* species than in *Bacillus*, mycobacterial, and cyanobacterial species, corroborating the superior capacity of *Streptomyces* species for generating diverse secondary metabolites. Functional analysis *via* data mining confirmed that many *Streptomyces* P450s are involved in the biosynthesis of secondary metabolites. This study was the first of its kind to conduct a comparative analysis of P450s in such a large number (203) of *Streptomyces* species, revealing the P450s’ association with secondary metabolite synthesis in *Streptomyces* species. Future studies should include the selection of *Streptomyces* species with a higher number of P450s and BGCs and explore the biotechnological value of secondary metabolites they produce.

## 1. Introduction

Cytochrome P450 monooxygenases (CYPs/P450s) are biotechnologically valuable enzymes [1]. P450s have heme (protoporphyrin IX), an iron(III)-containing porphyrin, as a prosthetic group in their structure [2]. Because of the presence of this prosthetic group, these enzymes absorb wavelengths at 450 nm; thus, the name P450s has been assigned to these proteins [3,4,5,6]. Since their identification, a large number of P450s have been identified in almost all living organisms [7] and, surprisingly, in non-living entities as well [8]. The regio- and stereo-specific catalytic nature of these enzymes makes them essential for the survival of some organisms, and these enzymes are thus good drug targets in the case of pathogenic organisms [9,10,11,12,13]. Application of these enzymes in all fields of research continues, and excellent success has been achieved in using them for the production of substances valuable to humans or as drug targets or in drug metabolism, as reported previously [1]. One of the applications of P450s currently being explored is their role in the production of secondary metabolites, compounds with potential biotechnological value, owing to their stereo- and regio-specific enzymatic activity, which contributes to the diversity of secondary metabolites [14,15,16].

Unlike other enzymes, P450 enzymes have a typical nomenclature system established by the International P450 Nomenclature Committee [17,18,19]. According to the committee’s rules, P450s begin with the prefix “CYP” for cytochrome P450 monooxygenase, followed by an Arabic numeral which designates the family, a capital letter designating the subfamily, and an Arabic numeral designating the individual P450 in a family. The annotation of P450s (assigning family and subfamily) follows a rule that all P450s with > 40% identity belong to the same family and all P450s with > 55% identity belong to the same subfamily [17,18,19]. Worldwide, researchers follow this P450 nomenclature system. The nomenclature of P450s is also be verified by phylogenetic analysis to enable their correct annotation, as phylogenetic-based annotation could detect similarity cues beyond a simple percentage identity cutoff, as mentioned elsewhere [20]. 

The continued genomic rush has resulted in genome sequencing of a large number of species belonging to all biological kingdoms. P450s in the newly sequenced species need to be annotated as per the International P450 Nomenclature Committee rules [17,18,19] to enable researchers to use the same names for functional and evolutionary analysis of P450s. For this reason, large numbers of P450s have recently been annotated in bacterial species belonging to the genera *Mycobacterium* [21], *Bacillus* [22], *Streptomyces* [20], and *Cyanobacteria* [23]. These studies have revealed numerous P450s involved in the synthesis of different types of secondary metabolites. This type of *in silico* study is highly important for identifying unique P450s that can be drug-targeted and for P450 evolutionary analysis, as the P450 profiles in species have been found to be characteristic of species’ lifestyle [11,20,22,24,25,26,27]. 

Among bacterial species, *Streptomyces* species are well-known for producing over two-thirds of the clinically useful antibiotics in the world [28]. Because of this importance, *Streptomyces* species have been subjected to exhaustive secondary metabolite production studies [29,30]. *Streptomyces* P450s play a key role in the production of different secondary metabolites; their contribution to secondary metabolite diversity and applications in drug metabolism have been reviewed extensively [15,16,31,32,33]. In the latest study, comprehensive comparative analysis of P450 and secondary metabolite biosynthetic gene clusters (BGCs) in 48 *Streptomyces* species was elucidated [20]. The study revealed the presence of novel P450s in *Streptomyces* species and numerous P450s forming parts of secondary metabolite BGCs [20]. The study results indicated that lifestyle or ecological niches play a key role in the evolution of P450 profiles in species belonging to the genera *Streptomyces* and *Mycobacterium* [20]. 

To date, a large number of *Streptomyces* species genomes have been sequenced and are available for public use. This provided an opportunity to annotate P450s in these species to analyze and compare their profiles among different bacterial species, including the identification and comparative analysis of P450s involved in the production of secondary metabolites. This study thus aimed to perform genome data mining, annotation, and phylogenetic analysis of P450s in 155 newly available *Streptomyces* species genomes. It also included the identification and comparative analysis of P450s that are parts of secondary metabolite BGCs among bacterial species belonging to the genera *Streptomyces*, *Bacillus*, *Mycobacterium*, and *Cyanobacteria,* as the species belonging to these genera are known to have P450s and to produce secondary metabolites. 

## 2. Results and Discussion

### 2.1. Streptomyces Species Have Large Number of P450s

**Figure 1:**
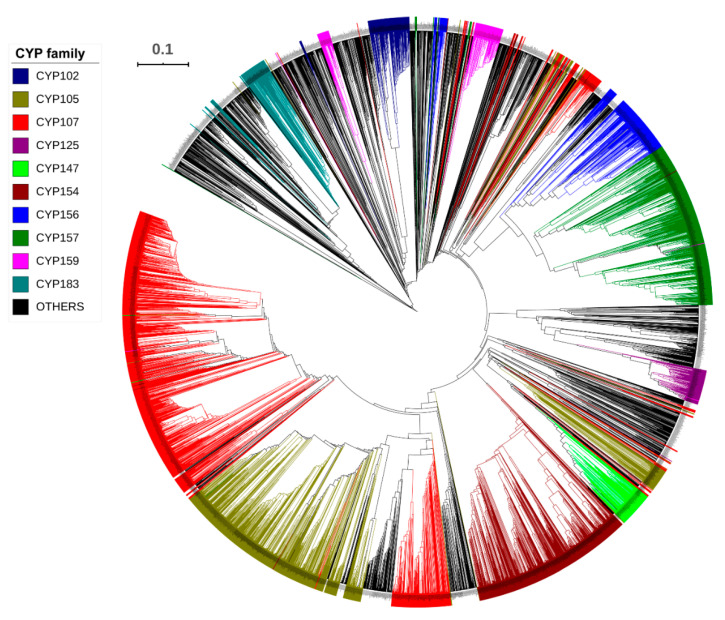
Phylogenetic analysis of *Streptomyces* P450s. In total, 5 460 P450s were used to construct the tree and the dominant P450 families are highlighted in different colors and indicated in the figure. A high-resolution phylogenetic tree is provided in Supplementary Dataset 2.

Genome-wide data mining and annotation of P450s in 203 *Streptomyces* species (Supplementary Table S1) revealed the presence of 5460 P450s in their genomes (Figure 1, Table 1, and Supplementary Dataset 1). The P450 count in the *Streptomyces* species ranged from 10 to 69 P450s, with an average of 27 P450s. Apart from the complete P450 sequences, pseudo-P450s (6 hit proteins), P450-fragments (114 hit proteins), P450-derived glycosyltransferase activator proteins (22 hit proteins), and P450 false-positive hits (2 hit proteins) were also found in some *Streptomyces* species (Supplementary Table S2). The presence of these types of P450 hit proteins in species is common and, because of the nature of these proteins, they were not included in the study for further analysis. Among *Streptomyces* species, *Streptomyces albulus* ZPM was found to have the highest number of P450s in its genome (69 P450s) followed by *S. clavuligerus* (65 P450s); the lowest number of P450s was found in *Streptomyces* sp. CNT372 and *S. somaliensis* DSM 40738 (10 P450s each) (Figure 1 and Table 1). Analysis of the most prevalent number of P450s revealed that 19 P450s was the prevalent number in *Streptomyces* species (Table 1). The average number of P450s in *Streptomyces* species was found to be higher than in *Bacillus* species [22] and cyanobacterial species [23], and almost the same as in mycobacterial species [21] (Table 2). A point to be noted is that the number of species greatly influences the average number of P450s and, thus, the higher the number of species in the analysis, the better and more accurate the results, as mentioned elsewhere [20,23]. This is the reason *Streptomyces* species showed a slightly lower average number of P450s in their genomes compared to mycobacterial species, since only 60 species were employed in the study [21]. Thus, future annotation of P450s in more mycobacterial species will provide accurate insights into this aspect.

### 2.2. CYP107 Family Was Found to Be Dominant and Conserved in 203 Streptomyces Species

Analysis of P450 families and subfamilies in 203 *Streptomyces* species revealed that 5460 P450s could be grouped into 253 P450 families and 698 P450 subfamilies (Table 2 and Supplementary Table S3). Among *Streptomyces* species, *S. clavuligerus* had the highest number of P450 families (30) and P450 subfamilies (58) in its genome (Table 1). Although *S. rimosus rimosus* ATCC 10970 had the same number of P450 families as *S. clavuligerus*, the number of subfamilies was the third highest (52 subfamilies) (Table 1). One interesting observation is that the species with the highest number of P450s did not have the highest number of P450 families, suggesting that some of the P450 families were populated (bloomed). Blooming of P450 families is common across species, and this phenomenon has been observed in different species belonging to different biological kingdoms [24,26,34,35,36]. Phylogenetic analysis revealed that some of the P450 families were scattered across the evolutionary tree (Figure 1). This phenomenon was also observed previously for *Streptomyces* species P450s, and it has been hypothesized that the phylogenetic-based annotation of P450s could be detecting similarity cues beyond a simple percentage identity cutoff [20]. Analysis of P450 families in the 155 *Streptomyces* species used in this study revealed the presence of 38 new P450 families, i.e., CYP1200A1, CYP1216A1, CYP1223A1, CYP1228A1, CYP1236A1, CYP1238A1, CYP1265A1, CYP1279A1, CYP1369A1, CYP1432A1, CYP1518A1, CYP1529A1, CYP1543A1, CYP1568A1, CYP159A1, CYP1607A1, CYP1658A1, CYP1759A1, CYP1810A1, CYP1832A1, CYP1866A1, CYP1896A1, CYP1920A1, CYP1929A1, CYP1931A1, CYP1940A1, CYP1941A1, CYP1943A1, CYP1972A1, CYP1984A1, CYP1994A1, CYP2076A1, CYP2080A1, CYP2134A1, CYP2180A1, CYP2349A1, CYP2427A1, and CYP2723A1. A detailed analysis of the number of new P450 families found in different *Streptomyces* species is presented in Supplementary Table S2.

**Figure 2:**
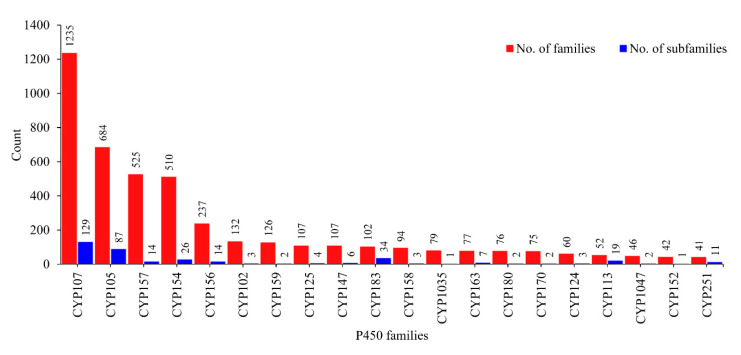
P450 family and subfamily analysis in 203 *Streptomyces* species. Only the dominant P450 families with more than 40 P450s are shown in the figure. Detailed data on P450 families and subfamilies are presented in Supplementary Table S3.

Among the P450 families, the CYP107 family was found to be dominant, with 1 235 P450s in *Streptomyces* species, followed by CYP105 with 684 P450s, CYP157 with 525 P450s, and CYP154 with 510 P450s (Figure 2 and Supplementary Table S3), indicating the possible blooming of these families in *Streptomyces* species, as observed in species belonging to different biological kingdoms [24,26,34,35,36]. It is interesting to note that the CYP107 family was also found to be dominant in the *Bacillus* species [22], indicating its dominant role in the synthesis of secondary metabolites in both the *Streptomyces* and *Bacillus* genera. An interesting pattern was observed when comparing subfamily diversity in the dominant P450 families (Figure 2, Table 3, and Supplementary Table S3). P450 families such as CYP107, CYP105, CYP183, and CYP113 had the highest diversity at the subfamily level, as numerous subfamilies were found in these families (Supplementary Table S3). This phenomenon of the highest diversity in P450 families being found in *Streptomyces* species is not uncommon, and this proved to be the key contributor in the production of diverse secondary metabolites in *Streptomyces* species compared to mycobacterial species [20]. Strong support for this argument is the fact that the CYP105 P450 family members in *Streptomyces* species have been shown to be involved in oxidation of numerous endogenous and exogenous compounds and in the generation of different secondary metabolites [32]. However, in contrast to the diversity at subfamily level for the P450 families CYP107, CYP105, CYP183, and CYP113, the rest of the dominant P450 families had single or double or triple subfamilies, indicating subfamily-level blooming in these P450 families (Table 3). 

**Figure 3:**
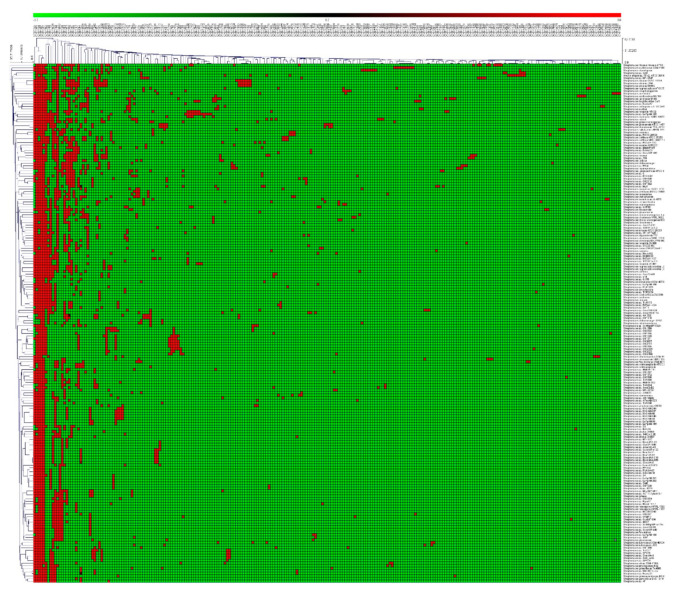
Heat-map of P450 family conservation analysis in *Streptomyces* species. In the heat-map, the presence and absence of P450 families are indicated in red and green colors. The horizontal axis represents P450 families and the vertical axis represents *Streptomyces* species.

P450 family conservation analysis revealed that the CYP107 family is conserved in all 203 *Streptomyces* species (Figure 3 and Supplementary Dataset 3). P450 families such as CYP156, CYP105, CYP154 and CYP157 are also present in the majority of the *Streptomyces* species (Figure 3 and Supplementary Dataset 3). 

### 2.3. Numerous P450s Involved in Secondary Metabolite Production in Streptomyces Compared to Other Bacterial Species

**Figure 4:**
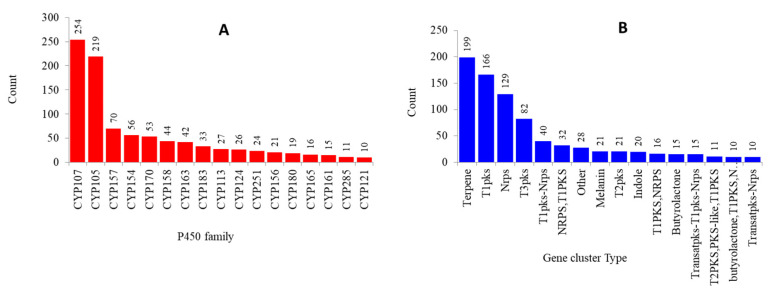
Analysis of P450s associated with secondary metabolite production in *Streptomyces* species. (**A**) Dominant P450 families (families with higher numbers of members) that are part of biosynthetic gene clusters (BGCs) and (**B**) dominant BGCs (present in higher numbers) containing P450s were presented in the figure. The numbers next to bars indicate the number of P450s in panel A and the number of BGCs in panel B. Detailed information is presented in Supplementary Table S5.

Analysis of 144 *Streptomyces* species’ genomes revealed the presence of 4457 BGCs in their genomes (Table 2 and Supplementary Table S4). The number of BGCs found in 144 *Streptomyces* species was found to be higher than in mycobacterial, *Bacillus*, and cyanobacterial species (Table 2), indicating the superiority of the *Streptomyces* species in producing secondary metabolites; two-thirds of the antibiotics used in the world currently come from these species [28]. The average number of BGCs in *Streptomyces* species was found to be double compared to mycobacterial species and close to four times higher than that in *Bacillus* and cyanobacterial species (Table 2). Analysis of BGCs revealed that a large proportion of *Streptomyces* species’ P450s are part of BGCs compared to other bacterial species; 1231 P450s in *Streptomyces* species compared to 112 in *Bacillus* species, 204 in mycobacterial species, and 27 in cyanobacterial species (Table 2). A total of 1231 P450s were found to be part of BGCs belonging to 135 P450 families (Figure 4 and Supplementary Table S5). Among 135 P450 families, P450s belonging to the CYP107 family were dominantly present in BGCs, followed by CYP105, CYP157, and CYP154 (Figure 4 and Supplementary Table S5). This clearly suggests that the P450 families that are bloomed in *Streptomyces* species are actually involved in the production of secondary metabolites. This strongly supports the proposed hypothesis that in *Streptomyces* species, P450s are evolved to generate secondary metabolites, thus helping these bacteria to thrive in their environment [20]. In order to assess the *in silico* results generated by this study, in which a large number of *Streptomyces* species P450s were predicted to be involved in secondary metabolite production, we performed an extensive literature review to identify *Streptomyces* P450s involved in the production of secondary metabolites. As shown in Table 4, a large number of P450s belonging to different P450 families, as predicted in this study, were found to be involved in the production of different secondary metabolites. This strongly supports the notion that the P450s identified as part of different BGCs in this study produce secondary metabolites. 

Analysis of P450 BGCs revealed the presence of 235 types of BGCs, where the BGC type, such as terpene, was dominant, followed by T1PKS, NRPS, and T3PKS (Figure 4 and Supplementary Table S5). A detailed analysis of P450s that are part of BGCs and types of BGCs containing P450s is presented in Supplementary Table S5. Analysis of the linkage between a particular P450 family and BGC revealed that some P450s are linked to a particular BGC (Supplementary Table S4), indicating horizontal transfer of BGCs between *Streptomyces* species. *Streptomyces* P450s such as CYP283A are linked to bacteriocin and bottromycin; CYP113K3 is linked to Bacteriocin-Nrps, CYP124G is linked to melanin, and CYP105A is linked to NRPS and butyrolactone. A point to be noted is that horizontal transfer of BGCs among different organisms is well-documented in the literature [37]. 

**Table 4:** List of *Streptomyces* species P450s involved in synthesis of secondary metabolites.

P450	Species	Function	References
CYP158A1	*Streptomyces coelicolor* A3(2)	Flaviolin biosynthesis	[38]
CYP1048A1	*Streptomyces scabiei*	Thaxtomin (phytotoxin) biosynthesis	[39]
CYP105A1	*Streptomyces griseolus*	Diterpenoids synthesis	[40]
CYP105A3 (P450sca-2)	*Streptomyces carbophilus*	Pravastatin synthesis	[41]
CYP105B28(GfsF) *	*Streptomyces graminofaciens*	Macrolide antibiotic synthesis	[42,43]
CYP105D6	*Streptomyces avermitilis*	Filipin biosynthesis	[44]
CYP105D7	*Streptomyces avermitilis*	Filipin biosynthesis	[45]
CYP105D8	*Streptomyces tubercidicus* strain I-1529	Avermectin oxidation	[32,46]
CYP105D9	*Streptomyces* sp. JP95	Griseorhodin biosynthesis	[32,47]
CYP105F2	*Streptomyces peucetius*	Oleandomycin biosynthesis	[48,49]
CYP105H1	*Streptomyces noursei* ATCC 11455	Nystatin biosynthesis	[32]
CYP105H3	*Streptomyces natalensis*	Pimaricin biosynthesis	[32,50]
CYP105H4 (AmphN) ^!^	*Streptomyces nodosus*	Amphotericin biosynthesis	[51,52]
CYP105H5	*Streptomyces griseus*	Candidicin biosynthesis	[32]
CYP105K1	*Streptomyces tendae* strain Tue901	Nikkomycin biosynthesis	[32,53]
CYP105K2	*Streptomyces ansochromogenes*	Nikkomycin biosynthesis	[32]
CYP105L1 (TylH1,orf7) ^!^	*Streptomyces fradiae*	Tylosin biosynthesis	[54,55]
CYP105L4(ChmH1) *	*Streptomyces bikiniensis*	Chalcomycin biosynthesis	[56]
CYP105M1 (orf10) ^!^	*Streptomyces clavuligerus*	Clavulanic acid antibiotic biosynthesis	[57]
CYP105N1	*Streptomyces coelicolor* A3(2)	Coelibactin siderophore biosynthesis	[58,59]
CYP105P1	*Streptomyces avermitilis*	Filipin biosynthesis	[44]
CYP105U1	*Streptomyces hygroscopicus*	Geldanamycin biosynthesis	[60]
CYP105V1	*Streptomyces* sp. HK803	Phoslactomycin biosynthesis	[32,61]
CYP105AA1	*Streptomyces tubercidicus* strain R922	Avermectin oxidation	[32,46]
CYP105AA2	*Streptomyces tubercidicus* strain I-1529	Avermectin oxidation	[32,46]
CYP107A1	*Streptomyces peucetius*	Dealkylation of 7-ethoxycoumarin	[62]
CYP107A1	*Saccharopolyspora erythraea*	Erythromycin biosynthesis	[63,64]
CYP107B (HmtN) ^!^	*Streptomyces himastatinicus* ATCC 53653	Himastatin biosynthesis	[65]
CYP107B (HmtN)	*Streptomyces himastatinicus*	Himastatin biosynthesis	[66]
CYP107C1	*Streptomyces thermotolerans*	Carbomycin biosynthesis	[67]
CYP107E40(chmPII) *	*Streptomyces bikiniensis*	Chalcomycin biosynthesis	[56]
CYP107EE2(chmPI) *	*Streptomyces bikiniensis*	Chalcomycin biosynthesis	[56]
CYP107FH5(TamI) *	*Streptomyces* sp. 307-9	Tirandamycin biosynthesis	[68,69]
CYP107G1	*Streptomyces rapamycinicus*	Rapamycin biosynthesis	[70,71]
CYP107G1 (rapN) ^!^	*Streptomyces hygroscopicus*	Rapamycin biosynthesis	[71,72]
CYP107L1	*Streptomyces venezuelae*	Macrolide antibioitics biosynthesis	[73]
CYP107L59(FosK) *	*Streptomyces pulveraceus*	Fostriecin biosynthesis	[74]
CYP107MD3(FosG) *	*Streptomyces pulveraceus*	Fostriecin biosynthesis	[74]
CYP107W1	*Streptomyces avermitilis*	Oligomycin A biosynthesis	[75,76]
CYP112A2	*Streptomyces rapamycinicus*	Rapamycin biosynthesis	[70,71]
CYP113A1	*Saccharopolyspora erythraea*	Erythromycin biosynthesis	[63,64]
CYP113B1 (TylI) ^!^	*Streptomyces fradiae*	Tylosin biosynthesis	[54,55]
CYP113D3(HmtT) *	*Streptomyces himastatinicus* ATCC 53653	Himastatin biosynthesis	[65]
CYP113D3 (HmtT) *	*Streptomyces himastatinicus*	Himastatin biosynthesis	[66]
CYP113HI (HmtS) *	*Streptomyces himastatinicus*	Himastatin biosynthesis	[66]
CYP122A2 (rapJ) ^!^	*Streptomyces hygroscopicus*	Rapamycin biosynthesis	[70,71]
CYP122A3	*Streptomyces hygroscopicus*	Rapamycin biosynthesis	[70,71]
CYP122A4 (FkbD) ^!^	*Streptomyces tsukubaensis*	FK506 (immunosuppressant) polyketide biosynthesis	[77]
CYP129A2	*Streptomyces peucetius*	Doxorubicin biosynthesis	[78,79]
CYP129A2 (dox A) ^!^	*Streptomyces* sp. strain C5	Doxorubicin biosynthesis	[80,81]
CYP131A2 (dnrQ) ^!^	*Streptomyces* sp. strain C5	Doxorubicin biosynthesis	[80,81]
CYP140M1(TtnI) *	*Streptomyces griseochromogenes*	Tautomycetin biosynthesis	[82]
CYP151A (AurH) ^!^	*Streptomyces thioluteus*	Aureothin biosynthesis	[83]
CYP154A1	*Streptomyces coelicolor* A3(2)	Polyketide synthesis and cyclization of a cellular dipentaenone	[84,85]
CYP154B1	*Streptomyces fradiae*	Tylosin biosynthesis	[54,55]
CYP154C1	*Streptomyces coelicolor* A3(2)	Macrolide biosynthesis	[86]
CYP158A2	*Streptomyces coelicolor* A3(2)	Flaviolin biosynthesis	[87]
CYP161A2 (PimD) ^!^	*Streptomyces natalensis*	Pimaricin biosynthesis	[88]
CYP161A3 (AmphL) ^!^	*Streptomyces nodosus*	Amphotericin biosynthesis	[51]
CYP162A1	*Streptomyces tendae*	Nikkomycin biosynthesis	[53,89]
CYP163A1 (NovI) ^!^	*Streptomyces spheroids*	Novobiocin biosynthesis	[90]
CYP163B3 (P450 Sky) ^!^	*Streptomyces* sp. Acta 2897	Skyllamycin biosynthesis	[91]
CYP170A1	*Streptomyces coelicolor* A3(2)	Albaflavenone biosynthesis	[92]
CYP170A2	*Streptomyces avermitilis*	Albaflavenone biosynthesis	[93]
CYP170B1	*Streptomyces albus*	Albaflavenone biosynthesis	[94]
CYP171A1	*Streptomyces avermitilis*	Avermectin biosynthesis	[95,96]
CYP183A1	*Streptomyces avermitilis*	Pentalenolactone biosynthesis	[96,97]
CYP244A1 (StaN) ^!^	*Streptomyces* sp tp-a0274	Rapamycin biosynthesis	[70,71]
CYP245A1 (StaP) ^!^	*Streptomyces* sp tp-a0274	Rapamycin biosynthesis	[70,71]
CYP246A1	*Streptomyces scabiei*	Thaxtomin (phytotoxin) biosynthesis	[98]
CYP248A1	*Streptomyces thioluteus*	Aureothin biosynthesis	[83]

Note: For some P450s, protein notations are given in parentheses. These P450s were annotated in this study (indicated with asterisk superscript) and previously (indicated with exclamation mark) [20] by browsing the individual biosynthetic gene-cluster sequences reported in the literature. To enable readers to match the P450s with the published literature, we have provided protein notations in the parentheses. If known, the name of the secondary metabolite of which P450s are involved in production is indicated in the table.

## 3. Materials and Methods

### 3.1. Information on Streptomyces Species and Genome Database 

In total, 203 *Streptomyces* species genomes (permanent and finished draft genomes) available for public use at the Joint Genome Institute Integrated Microbial Genomes and Microbiomes (JGI IMG/M) [99] and Kyoto Encyclopedia of Genes and Genomes (KEGG) [100] were used in this study. The 203 *Streptomyces* species included 48 *Streptomyces* species for which P450s and BGCs were annotated previously [20]. For these 48 species, P450 and BGCs data were retrieved from published articles and used in the study [20]. Thus, 155 *Streptomyces* species were data-mined for P450s and BGCs in this study. Information on the species used in the study is provided in Supplementary Table S1.

### 3.2. Genome Data Mining and Identification of P450s

Identification and annotation of P450s in *Streptomyces* species were carried out following a method described elsewhere [20,21,22]. Briefly, each *Streptomyces* species genome available at JGI IMG/M [99] was searched for P450s using the InterPro code “IPR001128”. The hit protein sequences were then searched for the presence of P450 characteristic motifs such as EXXR and CXG [101]. Proteins having one of these motifs were considered pseudo-P450s, and proteins that were short in amino acid length and lacking both motifs as P450 fragments. Neither the pseudo-P450s nor the P450 fragments were considered for further analysis. 

### 3.3. Allocating Family and Subfamily to P450s

The hit proteins that were collected were subjected to BLAST analysis against bacterial P450s at the website http://www.p450.unizulu.ac.za/. Based on the International P450 Nomenclature Committee rule [17,18,19], proteins with a percentage identity greater than 40% were assigned to the same family as named homolog P450s, and those that had greater than 55% identity were assigned to the same subfamily as named homolog P450s. Proteins that had a percentage identity less than 40% were assigned to a new family.

### 3.4. Streptomyces P450 Phylogenetic Analysis

Phylogenetic analysis of the *Streptomyces* P450s was carried out following the method described in the literature [102]. First, the *Streptomyces* P450 sequences were aligned using the MAFFT v6.864 program with an automatically optimized model option [103], available at the Trex web server [104]. The alignments were then automatically subjected to inference and optimization of the tree by the Trex web server with its embedded weighting procedure, and the best inferred tree was visualized and annotated by iTOL [105].

### 3.5. Streptomyces P450 Profile Heat-Maps

P450 profile heat-maps were generated following a method published previously [22,27] to check the presence and absence of P450s in *Streptomyces* species. Briefly, a tab-delimited file was imported into Multi-Experiment Viewer (Mev) [106] and hierarchical clustering using a Euclidean distance metric was used to cluster the data. In total, 203 *Streptomyces* species formed the vertical axis and P450 family numbers formed the horizontal axis. Data were presented as −3 for family absence (green) and 3 for family presence (red). 

### 3.6. Identification of P450s That Are Part of Secondary Metabolite BGCs

Secondary metabolite BGCs analysis and identification of P450s that are part of these BGCs were carried out following the procedure mentioned previously [102], with slight modification. For each *Streptomyces* species genome available at JGI IMG/M, the secondary metabolite BGCs were searched for the presence of P450s. The DNA sequence of BGCs with P450s was collected and formatted to fasta format using PSPad editor (http://www.pspad.com/en/). The fasta-formatted files were then used to identify the type of cluster and most similar known clusters using the Antibiotics and Secondary Metabolite Analysis Shell (anti-SMASH) program [107]. The results obtained were recorded on Excel spreadsheets and represented as species-wise BGCs, type and similar known BGCs, percentage similarity to known BGCs, and P450s that are part of specific BGCs. Some *Streptomyces* species genome IDs did not pass through anti-SMASH analysis, and thus these species were not included in P450s analysis as part of secondary metabolite BGCs. A list of *Streptomyces* species subjected to anti-SMASH analysis is presented in Supplementary Table S4.

### 3.7. Data Analysis

All calculations were done following the method described in the literature [23]. The average number of P450s was calculated using the formula: Average number of P450s = Number of P450s/Number of species. The average number of BGCs was calculated using the formula: Average number of BGCs = Total number of BGCs/Number of species. The percentage of P450s that formed part of BGCs was calculated using the formula: Percentage of P450s part of BGCs = 100 × Number of P450s part of BGCs /Total number of P450s present in species. For comparative analysis of P450s and BGCs, information for bacterial species belonging to the genera *Bacillus* [22], *Mycobacterium* [21], and *Cyanobacteria* [23] was resourced from published articles. 

## 4. Conclusions

In the last five decades, research on cytochrome P450 monooxygenases (CYPs/P450s) has mainly focused on their function and structural aspects, with little focus on evolutionary analysis, especially in microbes. The availability of a large number of microbial species genomes gives us an opportunity to focus on exploring the evolutionary aspects of P450s. Because a typical nomenclature system that has been established for P450s, each species genome needs to be data-mined and P450 proteins need to be annotated (assigning family and subfamily). In this way, researchers around the world can make use of uniform P450 names. In this study, we therefore annotated a large number of P450s in 203 *Streptomyces* species and found 38 new P450 families. Some P450 families were found to be bloomed in *Streptomyces* species even at the subfamily level. Comparative analysis of key P450 features among different bacterial species revealed that *Streptomyces* species had a greater number of P450s, more secondary metabolite BGCs, and the highest number of P450s as part of BGCs compared to the bacterial species belonging to the genera *Bacillus*, *Mycobacterium*, and *Cyanobacteria*. This further confirmed that the higher the number of P450s, the higher the secondary metabolite diversity in a species. This was true for *Streptomyces* species, as large number of P450s were found to be involved in the generation of diverse secondary metabolites. One interesting phenomenon observed was the linkage between a particular P450 family and BGC. This indicates that these BGCs were horizontally transferred among different *Streptomyces* species. This study is a good addition to the comparative analysis of P450s and BGCs among different bacterial populations. Data presented in the study will serve as a reference for further annotation of P450s in *Streptomyces* species and other bacterial species. *In silico* predicted BGCs need to be experimentally validated to assess the secondary metabolites’ biological properties. 

## Figures and Tables

**Table 1:** Genome-wide data mining and annotation of P450s in 203 *Streptomyces* species.

Species Name	P450s	No. F	No. SF	Species Name	P450s	No. F	No. SF
*Streptomyces* sp. Tu6071	22	13	20	*Streptomyces* sp. CNT372	10	8	10
*Streptomyces purpureus* KA281, ATCC 21405	22	17	20	*Streptomyces* sp. CNS606	16	9	14
*Streptomyces* sp. W007	28	12	24	*Streptomyces* sp. 303MFCol5.2	23	14	22
*Streptomyces* sp. TAA486-18	18	12	17	*Streptomyces acidiscabies* 84-104	47	22	44
*Streptomyces lysosuperificus* ATCC 31396	25	19	24	*Streptomyces roseosporus* NRRL 11379	19	10	16
*Streptomyces* sp. PVA 94-07	20	7	18	*Streptomyces* sp. OspMP-M45	19	9	19
*Streptomyces* sp. SPB78	20	12	20	*Streptomyces* sp. AmelKG-A3	19	9	19
*Streptomyces canus* 299MFChir4.1	28	17	27	*Streptomyces* sp. S4	19	9	19
*Streptomyces* sp. FxanaA7	30	15	29	*Streptomyces* sp. SM8	18	8	16
*Streptomyces sulphureus* DSM 40104	26	13	25	*Streptomyces* sp. LaPpAH-199	26	11	21
*Streptomyces* sp. MspMP-M5	44	20	41	*Streptomyces* sp. 140Col2.1E	22	9	17
*Streptomyces coelicoflavus* ZG0656	17	12	16	*Streptomyces* sp. DvalAA-21	24	10	22
*Streptomyces pristinaespiralis* ATCC 25486	18	11	17	*Streptomyces* sp. CNT371	17	13	17
*Streptomyces* sp. LaPpAH-201	19	8	19	*Streptomyces somaliensis* DSM 40738	10	8	9
*Streptomyces albulus* CCRC 11814	64	26	50	*Streptomyces* sp. 351MFTsu5.1	22	11	22
*Streptomyces viridochromogenes* DSM 40736	24	15	24	*Streptomyces* sp. DvalAA-83	24	10	22
*Streptomyces* sp. LaPpAH-95	24	9	22	*Streptomyces* sp. AmelKG-F2B	24	17	23
*Streptomyces mirabilis* YR139	42	26	41	*Streptomyces* sp. CNT302	26	13	22
*Streptomyces* sp. AA1529	26	15	24	*Streptomyces olindensis* DAUFPE 5622	26	14	22
*Streptomyces atratus* OK008	15	10	14	*Streptomyces* sp. CNY243	17	14	16
*Streptomyces* sp. PsTaAH-130	36	21	32	*Streptomyces* sp. AA0539	19	10	19
*Streptomyces* sp. CNT318	27	15	24	*Streptomyces atratus* OK807	31	13	27
*Streptomyces* sp. CNH099	16	12	16	*Streptomyces* sp. CNS335	16	13	17
*Streptomyces* sp. CNH287	16	12	16	*Streptomyces* sp. FxanaC1	27	15	24
*Streptomyces* sp. MnatMP-M77	32	14	27	*Streptomyces* sp. WMMB 322	19	11	17
*Streptomyces zinciresistens* K42	19	11	18	*Streptomyces* sp. TOR3209	20	13	19
*Streptomyces* sp. So1WspMP-so12th	22	11	19	*Streptomyces* sp. AmelKG-E11A	24	15	22
*Streptomyces* sp. GXT6	13	8	11	*Streptomyces* sp. PP-C42	16	6	14
*Streptomyces roseosporus* NRRL 15998	19	10	16	*Streptomyces* sp. DpondAA-E10	25	10	22
*Streptomyces* sp. LaPpAH-108	24	12	23	*Streptomyces* sp. HPH0547	32	18	32
*Streptomyces aurantiacus* JA 4570	30	20	30	*Streptomyces* sp. DpondAA-A50	25	10	22
*Streptomyces hygroscopicus* ATCC 53653	57	21	49	*Streptomyces* sp. TAA040	15	10	15
*Streptomyces* sp. Tu 6176	30	15	26	*Streptomyces* sp. PgraA7	23	10	20
*Streptomyces ghanaensis* ATCC 14672	35	20	34	*Streptomyces* sp. FxanaD5	15	11	15
*Streptomyces* sp. KhCrAH-337	26	12	22	*Streptomyces* sp. LamerLS-316	25	11	22
*Streptomyces* sp. LaPpAH-202	19	8	19	*Streptomyces viridochromogenes* Tue57	31	17	29
*Streptomyces* sp. UNC401CLCol	15	11	15	*Streptomyces* sp. GBA 94-10	20	7	18
*Streptomyces* sp. SirexAA-H	21	12	20	*Streptomyces* sp. CNQ-525	18	14	18
*Streptomyces turgidiscabies* Car8	28	20	27	*Streptomyces* sp. SceaMP-e96	41	18	36
*Streptomyces* sp. KhCrAH-40	26	12	22	*Streptomyces mirabilis* OK461	37	16	31
*Streptomyces rimosus rimosus* ATCC 10970	54	30	52	*Streptomyces* sp. LaPpAH-185	44	27	40
*Streptomyces gancidicus* BKS 13-15	18	11	17	*Streptomyces exfoliatus* DSMZ 41693	26	16	24
*Streptomyces auratus* AGR0001	35	14	33	*Streptomyces* sp. PsTaAH-137	29	16	28
*Kitasatospora* sp. SolWspMP-SS2h	25	15	24	*Streptomyces* sp. Amel2xE9	27	15	26
*Streptomyces* sp. NTK 937	17	8	17	*Streptomyces* sp. AmelKG-D3	22	11	19
*Streptomyces* sp. ScaeMP-e48	19	10	17	*Streptomyces prunicolor* NBRC 13075	44	18	39
*Streptomyces* sp. HmicA12	25	14	24	*Streptomyces* sp. e14	28	13	25
*Streptomyces griseoaurantiacus* M045	16	11	16	*Streptomyces* sp. CNX435	12	9	12
*Streptomyces afghaniensis* 772	28	17	29	*Streptomyces* sp. HCCB10043	17	10	14
*Streptomyces sulphureus* L180	19	11	19	*Streptomyces* sp. JS01	24	11	19
*Streptomyces* sp. KhCrAH-340	26	12	22	*Streptomyces chartreusis* NRRL 3882	29	19	26
*Streptomyces* sp. C	30	17	27	*Streptomyces* sp. CNY228	19	9	19
*Streptomyces violaceusniger* SPC6	13	8	12	*Streptomyces* sp. Amel2xB2	27	13	25
*Streptomyces* sp. HGB0020	23	13	22	*Streptomyces* sp. LaPpAH-165	24	9	22
*Streptomyces* sp. CNS615	27	15	24	*Streptomyces albulus* ZPM	68	27	51
*Streptomyces tsukubaensis* NRRL 18488	30	18	30	*Streptomyces albulus* NK660	64	27	50
*Streptomyces vitaminophilus* DSM 41686	18	10	15	*Streptomyces noursei*	64	26	52
*Streptomyces* sp. SA3_actG	21	12	20	*Streptomyces violaceusniger* Tu4113	50	16	42
*Streptomyces bottropensis* ATCC 25435 (2517572239)	31	19	30	*Streptomyces bingchenggensis*	49	26	44
*Streptomyces* sp. CNQ865	16	13	16	*Streptomyces rapamycinicus*	63	23	56
*Streptomyces* sp. CNT360	19	13	18	*Streptomyces* sp. 769	59	24	49
*Streptomyces* sp. 142MFCol3.1	27	14	24	*Streptomyces hygroscopicus* subsp. *jinggangensis* 5008	38	18	33
*Streptomyces* sp. ScaeMP-e122	25	11	23	*Streptomyces cattleya* NRRL 8058 = DSM 46488	41	21	38
*Streptomyces griseoflavus* Tu4000	20	15	19	*Streptomyces cattleya* NRRL 8057	40	20	37
*Streptomyces* sp. ACT-1	30	13	26	*Streptomyces hygroscopicus* subsp. *jinggangensis* TL01	37	18	33
*Streptomyces* sp. TAA204	18	10	16	*Streptomyces avermitilis* MA-4680	52	23	42
*Streptomyces* sp. SPB74	18	10	18	*Streptomyces collinus*	34	16	27
*Streptomyces* sp. CNQ329	13	10	13	*Streptomyces lydicus* A02	38	19	35
*Streptomyces* sp. 4F	16	11	15	*Streptomyces lydicus* 103	32	13	29
*Streptomyces* sp. KhCrAH-244	26	12	22	*Streptomyces* sp. Mg1	37	21	36
*Streptomyces chartreusis* NRRL 12338	23	15	23	*Streptomyces leeuwenhoekii* C34(2013)	36	17	34
*Streptomyces sviceus* ATCC 29083	19	12	19	*Streptomyces pratensis*/*flavogriseus* IAF 45	29	16	26
*Streptomyces* sp. CcalMP-8W	23	12	20	*Streptomyces reticuli*	47	26	43
*Streptomyces* sp. SS	15	11	15	*Streptomyces griseus*	28	13	24
*Streptomyces* sp. CNQ766	16	13	16	*Streptomyces* sp. PAMC 26508	29	16	26
*Streptomyces* sp. URHA0041	16	9	15	*Streptomyces* sp. SirexAA-E	24	10	22
*Streptomyces* sp. CNB091	27	14	24	*Streptomyces davawensis*	32	19	30
*Streptomyces flavidovirens* DSM 40150	24	15	23	*Streptomyces cyaneogriseus*	30	14	28
*Streptomyces yeochonensis* CN732	18	11	18	*Streptomyces lincolnensis*	24	15	23
*Streptomyces viridosporus* T7A, ATCC 39115	32	19	31	*Streptomyces pristinaespiralis* HCCB 10218	23	12	18
*Streptomyces* sp. FXJ7.023	27	12	23	*Streptomyces venezuelae*	23	16	21
*Streptomyces mirabilis* OV308	28	14	27	*Streptomyces* sp. CFMR 7	24	13	20
*Streptomyces* sp. AW19M42	27	12	24	*Streptomyces vietnamensis*	30	20	29
*Streptomyces* sp. ATexAB-D23	28	11	26	*Streptomyces xiamenensis* 318	19	12	19
*Streptomyces* sp. BoleA5	17	8	15	*Streptomyces coelicolor*	18	10	17
*Streptomyces* sp. AA4	35	17	29	*Streptomyces albus* J1074	18	9	18
*Streptomyces* sp. CNS654	27	10	22	*Streptomyces ambofaciens*	19	10	18
*Streptomyces ipomoeae* 91-03	44	26	43	*Streptomyces lividans*	20	10	18
*Streptomyces* sp. DpondAA-B6	19	9	19	*Streptomyces scabiei* 87.22	30	16	30
*Streptomyces* sp. PCS3-D2	25	18	24	*Streptomyces glaucescens*	18	11	17
*Streptomyces* sp. PRh5	57	20	51	*Streptomyces albus* DSM 41398	25	13	24
*Streptomyces* sp. CNR698	29	17	26	*Streptomyces fulvissimus*	19	10	16
*Amycolatopsis* sp. 75iv2, ATCC 39116	28	18	27	*Streptomyces* sp. CNQ-509	16	11	16
*Streptomyces cattleya* ATCC 35852	41	21	38	*Streptomyces rubrolavendulae*	20	12	19
*Streptomyces* sp. WMMB 714	21	10	18	*Streptomyces clavuligerus*	64	30	58
*Streptomyces scabrisporus* DSM 41855	37	27	36	*Streptomyces griseochromogenes*	46	24	40
*Streptomyces* sp. Ncost-T6T-1	25	14	22	*Streptomyces* sp. S10(2016)	20	15	20
*Streptomyces* sp. CNB632	16	12	16	*Streptomyces globisporus*	23	13	19
*Streptomyces mobaraensis* NBRC 13819	22	13	21	*Streptomyces* sp. CdTB01	26	17	25
*Streptomyces* sp. KhCrAH-43	26	12	22	*Streptomyces parvulus*	25	15	25
*Streptomyces* sp. PsTaAH-124	32	16	27	*Streptomyces* sp. SAT1	25	15	22
*Streptomyces* sp. Amel2xC10	15	10	15				

Abbreviations: No. F: number of P450 families; No. SF: number of P450 subfamilies.

**Table 2:** Comparative analysis of key features of P450s in different bacterial species.

	*Streptomyces* Species	Mycobacterial Species	*Bacillus* Species	Cyanobacterial Species
Total No. of species analyzed	203	60	128	114
No. of P450s	5460	1784	507	341
No. of families	253	77	13	36
No. of subfamilies	698	132	28	79
Dominant P450 family	CYP107	CYP125	CYP107	CYP110
Average no. of P450s	27	30	4	3
No. of BGCs *	4457	898	1098	770
Average no. of BGCs	31	15	9	7
No. of P450s part of BGCs	1231	204	112	27
Percentage of P450s part of BGCs	22	11	22	8
Reference	This work	[20,21]	[22]	[23]

Abbreviations: BGC: biosynthetic gene cluster. Symbol: * 103 cyanobacterial species [23] and 144 *Streptomyces* species were used for BGC analysis.

**Table 3:** P450 subfamily analysis in the dominant families in 203 *Streptomyces* species. The number of members in the dominant P450 subfamily is presented. Detailed data on different subfamilies are presented in Supplementary Table S3.

P450 Family	Dominant Subfamilies						
	A	B	C	D	E	F	G
CYP157	174		177				
CYP154	127		164	76			
CYP156		120					
CYP102		78					48
CYP159	125						
CYP125	104						
CYP147						73	
CYP158	91						
CYP1035	79						
CYP163		50					
CYP180	54						
CYP170	57						
CYP124							50
CYP1047	43						
CYP152				42			
CYP251	23						

## Supplementary Materials

Supplementary materials can be found at https://www.mdpi.com/1422-0067/21/13/4814/s1.
