# Supplementary material for: More P450s Are Involved in Secondary Metabolite Biosynthesis in Streptomyces Compared to Bacillus, Cyanobacteria, and Mycobacterium
Source: Int J Mol Sci. 2020 Jul 7;21(13):4814. doi: 10.3390/ijms21134814 (PMC7369989; doi:10.3390/ijms21134814)
Supplement: Supplementary file 1 [file ijms-21-04814-s001.zip › Supplementary Information/Mnguni et 2019 Supplementary Information.docx]

Article

More P450s are involved in secondary metabolite biosynthesis in *Streptomyces* compared to *Bacillus*, *Cyanobacteria* and *Mycobacterium*

Fanele Cabangile Mnguni ^1^, Tiara Padayachee ^1^, Wanping Chen ^2^, Dominik Gront ^3^, Jae-Hyuk Yu ^4,5^, David R. Nelson ^6^*, and Khajamohiddin Syed ^1^*

^1^ Department of Biochemistry and Microbiology, Faculty of Science and Agriculture, University of Zululand, KwaDlangezwa 3886, South Africa; fanelemngun@gmail.com (F.C.M.); teez07padayachee@gmail.com (T.P.); khajamohiddinsyed@gmail.com (K.S.)

^2^ Department of Molecular Microbiology and Genetics, University of Göttingen, Göttingen 37077, Germany; chenwanping1@foxmail.com (W.C.)

^3^ Faculty of Chemistry, Biological and Chemical Research Center, University of Warsaw, Pasteura 1, 02-093 Warsaw, Poland; dgront@gmail.com (D.G.)

^4^ Department of Bacteriology, University of Wisconsin-Madison, 3155 MSB, 1550 Linden Drive, Madison, WI 53706, USA; jyu1@wisc.edu (J-H.Y.)

^5^ Department of Systems Biotechnology, Konkuk University, Seoul 05029, Korea

^6^ Department of Microbiology, Immunology and Biochemistry, University of Tennessee Health Science Center, Memphis, TN 38163, USA; dnelson@uthsc.edu (D.R.N.)

***** Correspondence: drnelson1@gmail.com (D.R.N.) and khajamohiddinsyed@gmail.com (K.S.)

Received: date; Accepted: date; Published: date

**Table S1. List of the species used in the study.**

| **Species name** | **Reference** |
| --- | --- |
| *Streptomyces* sp. Tu6071 | This work |
| *Streptomyces purpureus* KA281, ATCC 21405 | This work |
| *Streptomyces* sp. W007 | This work |
| *Streptomyces* sp. TAA486 | This work |
| *Streptomyces* *lysosuperificus* ATCC 31396 | This work |
| *Streptomyces* sp. PVA 94-07 | This work |
| *Streptomyces* sp. SPB78 | This work |
| *Streptomyces* *canus* 299MFChir4.1 | This work |
| *Streptomyces* sp. FxanaA7 | This work |
| *Streptomyces* *sulphureus* DSM 40104 | This work |
| *Streptomyces* sp. MspMP-M5 | This work |
| *Streptomyces* *coelicoflavus* ZG0656 | This work |
| *Streptomyces* *pristinaespiralis* ATCC 25486 | This work |
| *Streptomyces* sp. LaPpAH-201 | This work |
| *Streptomyces* *albulus* CCRC 11814 | This work |
| *Streptomyces* *viridochromogenes* DSM 40736 | This work |
| *Streptomyces* sp. LaPpAH-95 | This work |
| *Streptomyces* *mirabilis* YR139 | This work |
| *Streptomyces* sp. AA1529 | This work |
| *Streptomyces* *atratus* OK008 | This work |
| *Streptomyces* sp. PsTaAH-130 | This work |
| *Streptomyces* sp. CNT318 | This work |
| *Streptomyces* sp. CNH099 | This work |
| *Streptomyces* sp. CNH287 | This work |
| *Streptomyces* sp. MnatMP-M77 | This work |
| *Streptomyces* *zinciresistens* K42 | This work |
| *Streptomyces* sp. So1WspMP-so12th | This work |
| *Streptomyces* sp. GXT6 | This work |
| *Streptomyces* *roseosporus* NRRL 15998 | This work |
| *Streptomyces* sp. LaPpAH-108 | This work |
| *Streptomyces* *aurantiacus J*A 4570 | This work |
| *Streptomyces* *hygroscopicus* ATCC 53653 | This work |
| *Streptomyces* sp. Tu 6176 | This work |
| *Streptomyces* *ghanaensis* ATCC 14672 | This work |
| *Streptomyces* sp. KhCrAH-337 | This work |
| *Streptomyces* sp. LaPpAH-202 | This work |
| *Streptomyces* sp. UNC401CLCol | This work |
| *Streptomyces* sp. SirexAA-H | This work |
| *Streptomyces* *turgidiscabies* Car8 | This work |
| *Streptomyces* sp. KhCrAH-40 | This work |
| *Streptomyces* *rimosus rimosus* ATCC 10970 | This work |
| *Streptomyces* *gancidicus* BKS 13-15 | This work |
| *Streptomyces* *auratus* AGR0001 | This work |
| *Kitasatospora* sp. SolWspMP-SS2h | This work |
| *Streptomyces* sp. NTK 937 | This work |
| *Streptomyces* sp. ScaeMP-e48 | This work |
| *Streptomyces* sp. HmicA12 | This work |
| *Streptomyces* *griseoaurantiacus* M045 | This work |
| *Streptomyces* *afghaniensis* 772 | This work |
| *Streptomyces* *sulphureus* L180 | This work |
| *Streptomyces* sp. KhCrAH-340 | This work |
| *Streptomyces* sp. C | This work |
| *Streptomyces* *violaceusniger* SPC6 | This work |
| *Streptomyces* sp. HGB0020 | This work |
| *Streptomyces* sp. CNS615 | This work |
| *Streptomyces* *tsukubaensis* NRRL 18488 | This work |
| *Streptomyces* *vitaminophilus* DSM 41686 | This work |
| *Streptomyces* sp. SA3_actG | This work |
| *Streptomyces* *bottropensis* ATCC 25435 | This work |
| *Streptomyces* sp. CNQ865 | This work |
| *Streptomyces* sp. CNT360 | This work |
| *Streptomyces* sp. 142MFCol3.1 | This work |
| *Streptomyces* sp. ScaeMP-e122 | This work |
| *Streptomyces* sp. ACT-1 XylebKG-1 | This work |
| *Streptomyces* sp. TAA204 | This work |
| *Streptomyces* sp. SPB74 | This work |
| *Streptomyces* sp. CNQ329 | This work |
| *Streptomyces* sp. KhCrAH-244 | This work |
| *Streptomyces* *chartreusis* NRRL 12338 | This work |
| *Streptomyces* *sviceus* ATCC 29083 | This work |
| *Streptomyces* sp. CcalMP-8W | This work |
| *Streptomyces* sp. SS | This work |
| *Streptomyces* sp. CNQ766 | This work |
| *Streptomyces* sp. URHA0041 | This work |
| *Streptomyces* sp. CNB091 | This work |
| *Streptomyces* *flavidovirens* DSM 40150 | This work |
| *Streptomyces* *yeochonensis* CN732 | This work |
| *Streptomyces* *viridosporus* T7A, ATCC 39115 | This work |
| *Streptomyces* sp. FXJ7.023 | This work |
| *Streptomyces* *mirabilis* OV308 | This work |
| *Streptomyces* sp. AW19M42 | This work |
| *Streptomyces* sp. ATexAB-D23 | This work |
| *Streptomyces* sp. BoleA5 | This work |
| *Streptomyces* sp. AA4 | This work |
| *Streptomyces* sp. CNS654 | This work |
| *Streptomyces* *ipomoeae* 91-03 | This work |
| *Streptomyces* sp. DpondAA-B6 | This work |
| *Streptomyces* sp. PCS3-D2 | This work |
| *Streptomyces* sp. PRh5 | This work |
| *Streptomyces* sp. CNR698 | This work |
| *Amycolatopsis* sp. 75iv2, ATCC 39116 | This work |
| *Streptomyces* *cattleya* ATCC 35852 | This work |
| *Streptomyces* sp. WMMB 714 | This work |
| *Streptomyces* *scabrisporus* DSM 41855 | This work |
| *Streptomyces* sp. Ncost-T6T-1 | This work |
| *Streptomyces* sp. CNB632 | This work |
| *Streptomyces* *mobaraensis* NBRC 13819 | This work |
| *Streptomyces* sp. KhCrAH-43 | This work |
| *Streptomyces* sp. PsTaAH-124 | This work |
| *Streptomyces* sp. Amel2xC10 | This work |
| *Streptomyces* *griseoflavus* Tu4000 | This work |
| *Streptomyces* sp. CNT372 | This work |
| *Streptomyces* sp. CNS606 | This work |
| *Streptomyces* sp. 303MFCol5.2 | This work |
| *Streptomyces* *acidiscabies* 84-104 | This work |
| *Streptomyces* *roseosporus* NRRL 11379 | This work |
| *Streptomyces* sp. OspMP-M45 | This work |
| *Streptomyces* sp. AmelKG-A3 | This work |
| *Streptomyces* sp. S4 | This work |
| *Streptomyces* sp. SM8 | This work |
| *Streptomyces* sp. LaPpAH-199 | This work |
| *Streptomyces* sp. 140Col2.1E | This work |
| *Streptomyces* sp. DvalAA-21 | This work |
| *Streptomyces* sp. CNT371 | This work |
| *Streptomyces* *somaliensis* DSM 40738 | This work |
| *Streptomyces* sp. 351MFTsu5.1 | This work |
| *Streptomyces* sp. Dva1AA-83 | This work |
| *Streptomyces* sp. AmelKG-F2B | This work |
| *Streptomyces* sp. CNT302 | This work |
| *Streptomyces* *olindensis* DAUFPE 5622 | This work |
| *Streptomyces* sp. CNY243 | This work |
| *Streptomyces* sp. AA0539 | This work |
| *Streptomyces* *atratus* OK807 | This work |
| *Streptomyces* sp. CNS335 | This work |
| *Streptomyces* sp. FxanaC1 | This work |
| *Streptomyces* sp. WMMB 322 | This work |
| *Streptomyces* sp. TOR3209 | This work |
| *Streptomyces* sp. AmelKG-E11A | This work |
| *Streptomyces* sp. PP-C42 | This work |
| *Streptomyces* sp. DpondAA-E10 | This work |
| *Streptomyces* sp. HPH0547 | This work |
| *Streptomyces* sp. DpondAA-A50 | This work |
| *Streptomyces* sp. TAA040 | This work |
| *Streptomyces* sp. PgraA7 | This work |
| *Streptomyces* sp. FxanaD5 | This work |
| *Streptomyces* sp. LamerLS-316 | This work |
| *Streptomyces* *viridochromogenes* Tue57 | This work |
| *Streptomyces* sp. GBA 94-10 | This work |
| *Streptomyces* sp. CNQ-525 | This work |
| *Streptomyces* sp. SceaMP-e96 | This work |
| *Streptomyces* *mirabilis* OK461 | This work |
| *Streptomyces* sp. LaPpAH-185 | This work |
| *Streptomyces* *exfoliatus* DSMZ 41693 | This work |
| *Streptomyces* sp. PsTaAH-137 | This work |
| *Streptomyces* sp. Ame12xE9 | This work |
| *Streptomyces* sp. AmelKG-D3 | This work |
| *Streptomyces* *prunicolor* NBRC 13075 | This work |
| *Streptomyces* sp. e14 | This work |
| *Streptomyces* sp. CNX435 | This work |
| *Streptomyces* sp. HCCB10043 | This work |
| *Streptomyces* sp. JS01 | This work |
| *Streptomyces* *chartreusis* NRRL 3882 | This work |
| *Streptomyces* sp. CNY228 | This work |
| *Streptomyces* sp. Amel2xB2 | This work |
| *Streptomyces* sp. LaPpAH-165 | This work |
| *Streptomyces* *albulus* ZPM | [1] |
| *Streptomyces* *albulus* NK660 | [1] |
| *Streptomyces* *noursei* | [1] |
| *Streptomyces* *violaceusniger* | [1] |
| *Streptomyces* *bingchenggensis* | [1] |
| *Streptomyces* *rapamycinicus* | [1] |
| *Streptomyces* sp. 769 | [1] |
| *Streptomyces* *hygroscopicus* subsp. jinggangensis 5008 | [1] |
| *Streptomyces* *cattleya* NRRL 8058 = DSM 46488 | [1] |
| *Streptomyces cattleya* NRRL 8057 | [1] |
| *Streptomyces* *hygroscopicus* subsp. jinggangensis TL01 | [1] |
| *Streptomyces* *avermitilis* | [1] |
| *Streptomyces* *collinus* | [1] |
| *Streptomyces lydicus* A02 | [1] |
| *Streptomyces lydicus* 103 | [1] |
| *Streptomyces* sp. Mg1 | [1] |
| *Streptomyces* *leeuwenhoekii* | [1] |
| *Streptomyces* *pratensis* | [1] |
| *Streptomyces* *reticuli* | [1] |
| *Streptomyces* *griseus* | [1] |
| *Streptomyces* sp. PAMC 26508 | [1] |
| *Streptomyces* sp. SirexAA-E | [1] |
| *Streptomyces* *davawensis* | [1] |
| *Streptomyces* *cyaneogriseus* | [1] |
| *Streptomyces* *lincolnensis* | [1] |
| *Streptomyces* *pristinaespiralis* | [1] |
| *Streptomyces* *venezuelae* | [1] |
| *Streptomyces* sp. CFMR 7 | [1] |
| *Streptomyces* *vietnamensis* | [1] |
| *Streptomyces* *xiamenensis* | [1] |
| *Streptomyces* *coelicolor* | [1] |
| *Streptomyces* *albus* J1074 | [1] |
| *Streptomyces* *ambofaciens* | [1] |
| *Streptomyces* *lividans* | [1] |
| *Streptomyces* *scabiei* | [1] |
| *Streptomyces* *glaucescens* | [1] |
| *Streptomyces* *albus* DSM 41398 | [1] |
| *Streptomyces* *fulvissimus* | [1] |
| *Streptomyces* sp. CNQ-509 | [1] |
| *Streptomyces* *rubrolavendulae* | [1] |
| *Streptomyces* *clavuligerus* | [1] |
| *Streptomyces* *griseochromogenes* | [1] |
| *Streptomyces* sp. S10(2016) | [1] |
| *Streptomyces* *globisporus* | [1] |
| *Streptomyces* sp. CdTB01 | [1] |
| *Streptomyces* *parvulus* | [1] |
| *Streptomyces* sp. SAT1 | [1] |
| *Streptomyces* sp. 4F | [1] |

Table S2. Information on hit proteins that are not part of the P450 analysis. Hit proteins along with their protein IDs and species in which they were found were listed at the bottom of the table according to the different categories.

| Species name | Pseudo P450 genes | fragments | P450-derived glycosyltransferase activator | false positive | New P450 families |
| --- | --- | --- | --- | --- | --- |
| *Streptomyces* sp. Tu6071 |  |  |  |  | 2 (CYP1200A1; CYP1972A1) |
| *Streptomyces purpureus* KA281, ATCC 21405 | 1 |  |  |  |  |
| *Streptomyces* sp. W007 |  | 3 |  |  |  |
| *Streptomyces* sp*.* TAA486-18 |  |  |  |  |  |
| *Streptomyces lysosuperificus* ATCC 31396 |  | 11 | 2 |  |  |
| *Streptomyces* sp. PVA 94-07 |  | 1 |  |  |  |
| *Streptomyces* sp. SPB78 |  |  |  |  | 2(CYP1200A1; CYP1972A1) |
| *Streptomyces canus* 299MFChir4.1 |  | 2 |  |  |  |
| *Streptomyces* sp. FxanaA7 |  |  |  |  |  |
| *Streptomyces sulphureus* DSM 40104 |  |  |  |  | 1(CYP1265A1) |
| *Streptomyces sp.* MspMP-M5 |  |  |  |  |  |
| *Streptomyces coelicoflavus* ZG0656 |  | 1 |  |  |  |
| *Streptomyces pristinaespiralis* ATCC 25486 |  | 1 |  |  |  |
| *Streptomyces sp.* LaPpAH-201 |  |  |  |  |  |
| *Streptomyces albulus* CCRC 11814 |  |  |  |  |  |
| *Streptomyces viridochromogenes* DSM 40736 |  | 2 |  |  |  |
| *Streptomyces sp.* LaPpAH-95 |  |  |  |  |  |
| *Streptomyces mirabilis* YR139 |  | 2 |  |  |  |
| *Streptomyces sp.* AA1529 |  |  |  |  |  |
| *Streptomyces atratus* OK008 |  | 1 |  |  |  |
| *Streptomyces* sp. PsTaAH-130 |  |  |  |  |  |
| *Streptomyces* sp. CNT318 |  |  |  |  |  |
| *Streptomyces* sp. CNH099 |  |  |  |  |  |
| *Streptomyces* sp. CNH287 |  | 1 |  |  |  |
| *Streptomyces* sp. MnatMP-M77 |  |  |  |  |  |
| *Streptomyces zinciresistens* K42 |  | 1 | 2 |  |  |
| *Streptomyces* sp. So1WspMP-so12th |  |  |  |  |  |
| *Streptomyces* sp. GXT6 |  |  |  |  |  |
| *Streptomyces roseosporus* NRRL 15998 |  |  | 1 |  |  |
| *Streptomyces* sp. LaPpAH-108 |  |  |  |  | 1(CYP159A1) |
| *Streptomyces aurantiacus* JA 4570 |  | 1 | 1 |  | 1(CYP1658A1) |
| *Streptomyces hygroscopicus* ATCC 53653 |  | 3 |  |  | 1(CYP1940A1) |
| *Streptomyces* sp. Tu 6176 |  | 1 |  |  |  |
| *Streptomyces ghanaensis* ATCC 14672 |  |  |  |  |  |
| *Streptomyces* sp. KhCrAH-337 |  |  |  |  |  |
| *Streptomyces* sp. LaPpAH-202 |  |  |  |  |  |
| *Streptomyces* sp. UNC401CLCol |  |  |  |  | 1(CYP1216A1) |
| *Streptomyces* sp. SirexAA-H |  | 2 |  |  | 2(CYP1200A1; CYP1972A1) |
| *Streptomyces turgidiscabies* Car8 |  |  |  |  | 1(CYP2134A1) |
| *Streptomyces sp.* KhCrAH-40 |  |  |  |  |  |
| *Streptomyces rimosus rimosus* ATCC 10970 | 1 | 1 |  |  | 4(CYP1236A1; CYP1896A1; CYP1929A1; CYP2349A1) |
| *Streptomyces gancidicus* BKS 13-15 |  |  |  |  |  |
| *Streptomyces auratus* AGR0001 |  |  |  |  |  |
| *Kitasatospora* sp. SolWspMP-SS2h |  |  |  |  |  |
| *Streptomyces* sp. NTK 937 |  |  |  |  |  |
| *Streptomyces* sp. ScaeMP-e48 |  | 1 |  |  |  |
| *Streptomyces* sp. HmicA12 |  |  |  |  | 1(CYP1238A1) |
| *Streptomyces griseoaurantiacus* M045 |  |  |  |  | 1(CYP2080A1) |
| *Streptomyces afghaniensis* 772 |  | 2 |  |  |  |
| *Streptomyces sulphureus* L180 |  |  |  |  |  |
| *Streptomyces* sp. KhCrAH-340 |  |  |  |  |  |
| *Streptomyces* sp. C |  | 1 |  |  |  |
| *Streptomyces violaceusniger* SPC6 |  |  |  |  |  |
| *Streptomyces* sp. HGB0020 |  | 1 |  |  | 1(CYP1943A1) |
| *Streptomyces* sp. CNS615 |  |  | 2 |  |  |
| *Streptomyces tsukubaensis* NRRL 18488 |  | 2 | 2 |  | 1(CYP1866A1) |
| *Streptomyces vitaminophilus* DSM 41686 |  |  |  |  | 1(CYP2180A1) |
| *Streptomyces* sp. SA3_actG |  | 1 |  |  | 2(CYP1200A1; CYP1972A1) |
| *Streptomyces bottropensis* ATCC 25435 |  |  |  |  |  |
| *Streptomyces* sp. CNQ865 |  | 1 |  |  | 1(CYP1279A1) |
| *Streptomyces* sp. CNT360 |  |  |  |  |  |
| *Streptomyces* sp. 142MFCol3.1 |  |  |  |  |  |
| *Streptomyces* sp. ScaeMP-e122 |  |  |  |  |  |
| *Streptomyces* sp. ACT-1 |  |  |  |  |  |
| *Streptomyces* sp. TAA204 |  | 1 |  |  | 1(CYP1832A1) |
| *Streptomyces* sp. SPB74 |  | 1 | 1 | 1 |  |
| *Streptomyces* sp. CNQ329 |  |  |  |  |  |
| *Streptomyces* sp. KhCrAH-244 |  |  | 1 |  |  |
| *Streptomyces chartreusis* NRRL 12338 |  |  |  |  |  |
| *Streptomyces sviceus* ATCC 29083 |  |  |  |  | 1(CYP1518A1) |
| *Streptomyces* sp. CcalMP-8W |  |  | 1 |  | 1(CYP1984A1) |
| *Streptomyces* sp. SS |  | 1 |  |  |  |
| *Streptomyces* sp. CNQ766 |  |  |  |  | 1(CYP1279A1) |
| *Streptomyces* sp. URHA0041 |  |  | 1 |  |  |
| *Streptomyces* sp. CNB091 |  |  |  |  |  |
| *Streptomyces flavidovirens* DSM 40150 |  |  |  |  | 1(CYP1228A1) |
| *Streptomyces yeochonensis* CN732 |  |  |  |  |  |
| *Streptomyces viridosporus* T7A, ATCC 39115 |  |  |  |  |  |
| *Streptomyces* sp. FXJ7.023 |  |  |  |  |  |
| *Streptomyces mirabilis* OV308 |  | 1 |  |  |  |
| *Streptomyces* sp. AW19M42 |  |  |  | 1 |  |
| *Streptomyces* sp. ATexAB-D23 |  |  |  |  |  |
| *Streptomyces* sp. BoleA5 |  | 3 |  |  |  |
| *Streptomyces* sp. AA4 |  |  |  |  | 3(CYP1543A1; CYP1759A1; CYP2076A1) |
| *Streptomyces* sp. CNS654 |  |  |  |  |  |
| *Streptomyces ipomoeae* 91-03 |  |  |  |  | (CYP1931A1) |
| *Streptomyces* sp. DpondAA-B6 |  |  |  |  |  |
| *Streptomyces* sp. PCS3-D2 |  |  |  |  |  |
| *Streptomyces* sp. PRh5 |  | 4 |  |  |  |
| *Streptomyces* sp. CNR698 |  |  |  |  | 1(CYP1223A1) |
| *Amycolatopsis* sp. 75iv2, ATCC 39116 |  |  |  |  | 2(CYP1432A1; CYP1994A1) |
| *Streptomyces cattleya* ATCC 35852 |  | 1 |  |  |  |
| *Streptomyces* sp. WMMB 714 |  | 1 |  |  |  |
| *Streptomyces scabrisporus* DSM 41855 | 1 | 4 |  |  | 2(CYP1369A1; CYP1568A1) |
| *Streptomyces* sp. Ncost-T6T-1 |  |  |  |  |  |
| *Streptomyces* sp. CNB632 |  |  |  |  |  |
| *Streptomyces mobaraensis* NBRC 13819 |  |  |  |  | 1(CYP1810A1) |
| *Streptomyces* sp. KhCrAH-43 |  | 1 |  |  |  |
| *Streptomyces* sp. PsTaAH-124 |  |  |  |  |  |
| *Streptomyces* sp. Amel2xC10 |  |  |  |  |  |
| *Streptomyces griseoflavus Tu4000* |  | 2 |  |  |  |
| *Streptomyces* sp. CNT372 | 1 |  |  |  |  |
| *Streptomyces* sp. CNS606 |  |  |  |  |  |
| *Streptomyces* sp. 303MFCol5.2 |  |  |  |  |  |
| *Streptomyces acidiscabies* 84-104 | 1 | 1 |  |  | 1(CYP1607A1) |
| *Streptomyces roseosporus* NRRL 11379 |  |  | 1 |  |  |
| *Streptomyces* sp. OspMP-M45 |  |  |  |  |  |
| *Streptomyces* sp. AmelKG-A3 |  |  |  |  |  |
| *Streptomyces* sp. S4 |  |  |  |  |  |
| *Streptomyces* sp. SM8 |  |  | 1 |  |  |
| *Streptomyces* sp. LaPpAH-199 |  |  |  |  |  |
| *Streptomyces* sp. 140Col2.1E |  |  |  |  |  |
| *Streptomyces* sp. DvalAA-21 |  |  |  |  |  |
| *Streptomyces* sp. CNT371 |  |  |  |  | 1(CYP1279A1) |
| *Streptomyces somaliensis* DSM 40738 |  |  |  |  |  |
| *Streptomyces* sp. 351MFTsu5.1 |  |  |  |  |  |
| *Streptomyces* sp. Dva1AA-83 |  |  |  |  |  |
| *Streptomyces* sp. AmelKG-F2B |  |  |  |  |  |
| *Streptomyces* sp. CNT302 |  |  | 2 |  |  |
| *Streptomyces olindensis* DAUFPE 5622 |  | 1 | 2 |  |  |
| *Streptomyces* sp. CNY243 |  |  |  |  | 1(CYP1279A1) |
| *Streptomyces* sp. AA0539 |  |  |  |  |  |
| *Streptomyces atratus* OK807 |  | 2 |  |  |  |
| *Streptomyces* sp. CNS335 |  |  |  |  | 1(CYP1279A1) |
| *Streptomyces* sp. FxanaC1 |  |  |  |  |  |
| *Streptomyces* sp. WMMB 322 |  |  |  |  |  |
| *Streptomyces* sp. TOR3209 |  |  |  |  |  |
| *Streptomyces* sp. AmelKG-E11A |  |  |  |  | 1(CYP2427A1) |
| *Streptomyces* sp. PP-C42 |  | 10 |  |  |  |
| *Streptomyces* sp. DpondAA-E10 |  |  |  |  |  |
| *Streptomyces* sp. HPH0547 |  |  |  |  | 2(CYP1920A1; CYP1941A1) |
| *Streptomyces* sp. DpondAA-A50 |  |  |  |  |  |
| *Streptomyces* sp. TAA040 |  | 1 |  |  |  |
| *Streptomyces* sp. PgraA7 |  | 1 |  |  |  |
| *Streptomyces* sp. FxanaD5 |  |  |  |  | 1(CYP1216A1) |
| *Streptomyces* sp. LamerLS-316 |  |  |  |  |  |
| *Streptomyces viridochromogenes* Tue57 |  | 2 |  |  |  |
| *Streptomyces* sp. GBA 94-10 |  |  |  |  |  |
| *Streptomyces* sp. CNQ-525 |  |  |  |  | 2(CYP1279A1; CYP1529A1) |
| *Streptomyces* sp. SceaMP-e96 |  |  |  |  |  |
| *Streptomyces mirabilis* OK461 |  | 1 |  |  |  |
| *Streptomyces* sp. LaPpAH-185 |  |  |  |  | 1(CYP2723A1) |
| *Streptomyces exfoliatus* DSMZ 41693 |  | 1 | 1 |  |  |
| *Streptomyces* sp. PsTaAH-137 |  |  |  |  |  |
| *Streptomyces* sp. Amel2xE9 |  |  |  |  |  |
| *Streptomyces* sp. AmelKG-D3 |  |  |  |  |  |
| *Streptomyces prunicolor* NBRC 13075 |  | 2 |  |  |  |
| *Streptomyces* sp. e14 |  |  |  |  |  |
| *Streptomyces* sp. CNX435 |  |  |  |  |  |
| *Streptomyces* sp. HCCB10043 |  |  | 1 |  |  |
| *Streptomyces* sp. JS01 |  |  |  |  |  |
| *Streptomyces chartreusis* NRRL 3882 |  | 17 |  |  |  |
| *Streptomyces* sp. CNY228 |  |  |  |  |  |
| *Streptomyces* sp. Amel2xB2 |  |  |  |  |  |
| *Streptomyces* sp. LaPpAH-165 |  |  |  |  |  |
| *Streptomyces albulus* ZPM |  | 1 |  |  |  |
| *Streptomyces albulus* NK660 |  |  |  |  |  |
| *Streptomyces noursei* |  |  |  |  |  |
| *Streptomyces violaceusniger* |  | 1 |  |  |  |
| *Streptomyces bingchenggensis* |  | 1 |  |  |  |
| *Streptomyces rapamycinicus* |  |  |  |  |  |
| *Streptomyces* *sp. 769* |  |  |  |  |  |
| *Streptomyces hygroscopicus* subsp. *jinggangensis* 5008 |  |  |  |  |  |
| *Streptomyces cattleya* NRRL 8058 = DSM 46488 |  | 1 |  |  |  |
| *Streptomyces cattleya* NRRL 8057 |  | 1 |  |  |  |
| *Streptomyces hygroscopicus* subsp. *jinggangensis* TL01 |  |  |  |  |  |
| *Streptomyces avermitilis* |  | 1 |  |  |  |
| *Streptomyces collinus* |  |  |  |  |  |
| *Streptomyces lydicus* A02 | 1 | 1 |  |  |  |
| *Streptomyces lydicus* 103 |  |  |  |  |  |
| *Streptomyces* sp. Mg1 |  |  |  |  |  |
| *Streptomyces leeuwenhoekii* |  |  |  |  |  |
| *Streptomyces pratensis* |  |  |  |  |  |
| *Streptomyces reticuli* |  |  |  |  |  |
| *Streptomyces griseus* |  |  |  |  |  |
| *Streptomyces* sp. PAMC 26508 |  |  |  |  |  |
| *Streptomyces* sp. SirexAA-E |  |  |  |  |  |
| *Streptomyces davawensis* |  |  |  |  |  |
| *Streptomyces cyaneogriseus* |  | 3 |  |  |  |
| *Streptomyces lincolnensis* |  |  |  |  |  |
| *Streptomyces pristinaespiralis* |  |  |  |  |  |
| *Streptomyces venezuelae* |  |  |  |  |  |
| *Streptomyces* sp. CFMR 7 |  |  |  |  |  |
| *Streptomyces vietnamensis* |  |  |  |  |  |
| *Streptomyces xiamenensis* |  | 1 |  |  |  |
| *Streptomyces coelicolor* |  |  |  |  |  |
| *Streptomyces* albus J1074 |  |  |  |  |  |
| *Streptomyces ambofaciens* |  |  |  |  |  |
| *Streptomyces lividans* |  |  |  |  |  |
| *Streptomyces scabiei* |  |  |  |  |  |
| *Streptomyces glaucescens* |  |  |  |  |  |
| *Streptomyces albus* DSM 41398 |  |  |  |  |  |
| *Streptomyces fulvissimus* |  |  |  |  |  |
| *Streptomyces* sp. CNQ-509 |  |  |  |  |  |
| *Streptomyces rubrolavendulae* |  |  |  |  |  |
| *Streptomyces clavuligerus* |  | 1 |  |  |  |
| *Streptomyces griseochromogenes* |  |  |  |  |  |
| *Streptomyces* sp. S10(2016) |  |  |  |  |  |
| *Streptomyces globisporus* |  |  |  |  |  |
| *Streptomyces* sp. CdTB01 |  |  |  |  |  |
| *Streptomyces parvulus* |  |  |  |  |  |
| *Streptomyces* sp. SAT1 |  |  |  |  |  |
| *Streptomyces* sp. 4F |  |  |  |  |  |
|  | 6 | 114 | 22 | 2 |  |

**PseudoP450s:**

(2516522252) *Streptomyces purpureus* KA281, ATCC 21405

(2545383032) *Streptomyces* rimosus rimosus ATCC 10970

(2516057102) *Streptomyces* scabrisporus DSM 41855

(2518016638) *Streptomyces* sp. CNT372

(2547558849) *Streptomyces* acidiscabies 84-104

>CYP107L52P(2652740080) Streptomyces lydicus A02

**Fragments:**

(2514827772) *Streptomyces* sp. W007

(2514827914) *Streptomyces* sp. W007

(2514822429) *Streptomyces* sp. W007

(2547449594) *Streptomyces* lysosuperificus ATCC 31396

(2547449800) *Streptomyces* lysosuperificus ATCC 31396

(2547450809) *Streptomyces* lysosuperificus ATCC 31396

(2547450877) *Streptomyces* lysosuperificus ATCC 31396

(2547450898) *Streptomyces* lysosuperificus ATCC 31396

(2547450924) *Streptomyces* lysosuperificus ATCC 31396

(2547454471) *Streptomyces* lysosuperificus ATCC 31396

(2547454745) *Streptomyces* lysosuperificus ATCC 31396

(2547454761) *Streptomyces* lysosuperificus ATCC 31396

(2547455540) *Streptomyces* lysosuperificus ATCC 31396

(2547455305) *Streptomyces* lysosuperificus ATCC 31396

(2597943189) *Streptomyces* sp. PVA 94-07

(2521769196) *Streptomyces* canus 299MFChir4.1

(2521769197) *Streptomyces* canus 299MFChir4.1

(2536367479) *Streptomyces* coelicoflavus ZG0656

(648861739) *Streptomyces* pristinaespiralis ATCC 25486

(645408928) *Streptomyces* viridochromogenes DSM 40736

(645414127) *Streptomyces* viridochromogenes DSM 40736

(2585311799) *Streptomyces* mirabilis YR139

(2585313749) *Streptomyces* mirabilis YR139

(2585298836) *Streptomyces* atratus OK008

(2524584987) *Streptomyces* sp. CNH287

(2532535361) *Streptomyces* zinciresistens K42

(2546763799) *Streptomyces* aurantiacus JA 4570

(645393084) *Streptomyces* hygroscopicus ATCC 53653

(645397707) *Streptomyces* hygroscopicus ATCC 53653

(645397708) *Streptomyces* hygroscopicus ATCC 53653

(2580702290) *Streptomyces* sp. Tu 6176

(2519147290) *Streptomyces* sp. SirexAA-H

(2519149156) *Streptomyces* sp. SirexAA-H

(2545383737) *Streptomyces* rimosus rimosus ATCC 10970

(2519058509) *Streptomyces* sp. ScaeMP-e48

(2546774595) *Streptomyces* afghaniensis 772

(2546776814) *Streptomyces* afghaniensis 772

(645383576) *Streptomyces* sp. C

(2541394753) *Streptomyces* sp. HGB0020

(2530555715) *Streptomyces* tsukubaensis NRRL 18488

(2530556468) *Streptomyces* tsukubaensis NRRL 18488

(650124476) *Streptomyces* sp. SA3_actG

(2524937753) *Streptomyces* sp. CNQ865

(2524962433) *Streptomyces* sp. TAA204

(648006388) *Streptomyces* sp. SPB74

(2551975180) *Streptomyces* sp. SS

(2585302911) *Streptomyces* mirabilis OV308

(2515915956) *Streptomyces* sp. BoleA5

(2515922864) *Streptomyces* sp. BoleA5

(2515922865)*Streptomyces* sp. BoleA5

(2580420291) *Streptomyces* sp. PRh5

(2580425623) *Streptomyces* sp. PRh5

(2580426841) *Streptomyces* sp. PRh5

(2580426890) *Streptomyces* sp. PRh5

(2504911443) *Streptomyces* cattleya ATCC 35852

(2522462118) *Streptomyces* sp. WMMB 714

(2516053686) *Streptomyces* scabrisporus DSM 41855

(2516055596) *Streptomyces* scabrisporus DSM 41855

(2516062277) *Streptomyces* scabrisporus DSM 41855

(2516062278) *Streptomyces* scabrisporus DSM 41855

(2525013413) *Streptomyces* sp. KhCrAH-43

(645415805) *Streptomyces* griseoflavus Tu4000

(645422196) *Streptomyces* griseoflavus Tu4000

(2547561509) *Streptomyces* acidiscabies 84-104

(2587272320) *Streptomyces* olindensis DAUFPE 5622

(2616905248) *Streptomyces* atratus OK807

(2616905545) *Streptomyces* atratus OK807

(2548248266) *Streptomyces* sp. PP-C42

(2548249058) *Streptomyces* sp. PP-C42

(2548249641) *Streptomyces* sp. PP-C42

(2548249834) *Streptomyces* sp. PP-C42

(2548251009) *Streptomyces* sp. PP-C42

(2548251411) *Streptomyces* sp. PP-C42

(2548251993) *Streptomyces* sp. PP-C42

(2548253201) *Streptomyces* sp. PP-C42

(2548253624) *Streptomyces* sp. PP-C42

(2548254890) *Streptomyces* sp. PP-C42

(2524959868) *Streptomyces* sp. TAA040

(2525360972) *Streptomyces* sp. PgraA7

(2533804035) *Streptomyces* viridochromogenes Tue57

(2533804524) *Streptomyces* viridochromogenes Tue57

(2616703446) *Streptomyces* mirabilis OK461

(2586355063) *Streptomyces* exfoliatus DSMZ 41693

(2567286375) *Streptomyces* prunicolor NBRC 13075

(2567286376) *Streptomyces* prunicolor NBRC 13075

(2547455945) *Streptomyces* chartreusis NRRL 3882

(2547456809) *Streptomyces* chartreusis NRRL 3882

(2547457179) *Streptomyces* chartreusis NRRL 3882

(2547458319) *Streptomyces* chartreusis NRRL 3882

(2547458439) *Streptomyces* chartreusis NRRL 3882

(2547459156) *Streptomyces* chartreusis NRRL 3882

(2547459757) *Streptomyces* chartreusis NRRL 3882

(2547461528) *Streptomyces* chartreusis NRRL 3882

(2547462429) *Streptomyces* chartreusis NRRL 3882

(2547462429) *Streptomyces* chartreusis NRRL 3882

(2547462635) *Streptomyces* chartreusis NRRL 3882

(2547463578) *Streptomyces* chartreusis NRRL 3882

(2547463766) *Streptomyces* chartreusis NRRL 3882

(2547464297) *Streptomyces* chartreusis NRRL 3882

(2547465028) *Streptomyces* chartreusis NRRL 3882

(2547466045) *Streptomyces* chartreusis NRRL 3882

(2547466069) *Streptomyces* chartreusis NRRL 3882

(2641170384) *Streptomyces* albulus ZPM

(2712578143) *Streptomyces* avermitilis

(646978401) *Streptomyces* bingchenggensis

(647544071) *Streptomyces* clavuligerus

(2649527539) *Streptomyces* cyaneogriseus

(2649527540) *Streptomyces* cyaneogriseus

(2649527873) *Streptomyces* cyaneogriseus

(648750691) *Streptomyces* violaceusniger

(2633776877) *Streptomyces* xiamenensis

CYP107CJ2(2652740805) Streptomyces lydicus A02

CYP107AS(2511977159) Streptomyces cattleya NRRL 8058 = DSM 46488

CYP107AS-fragment1(2511677887) Streptomyces cattleya NRRL 8057

**P450-derived glycosyltransferase activator:**

(2547446153) *Streptomyces* lysosuperificus ATCC 31396

(2547454902) *Streptomyces* lysosuperificus ATCC 31396

(2532534676) *Streptomyces* zinciresistens K42

(2532534687) *Streptomyces* zinciresistens K42

(645215040) *Streptomyces* roseosporus NRRL 15998

(2546764930) *Streptomyces* aurantiacus JA 4570

(2518400808) *Streptomyces* sp. CNS615

(2518400829) *Streptomyces* sp. CNS615

(2530553835) *Streptomyces* tsukubaensis NRRL 18488

(2530553842) *Streptomyces* tsukubaensis NRRL 18488

(648009744) *Streptomyces* sp. SPB74

(2515591352) *Streptomyces* sp. KhCrAH-244

(2515529209) *Streptomyces* sp. CcalMP-8W

(2562037229) *Streptomyces* sp. URHA0041

(645232145) *Streptomyces* roseosporus NRRL 11379

(2512067890) *Streptomyces* sp. SM8

(2518083496) *Streptomyces* sp. CNT302

(2518083517) *Streptomyces* sp. CNT302

(2587271846) *Streptomyces* olindensis DAUFPE 5622

(2587271867) *Streptomyces* olindensis DAUFPE 5622

(2586355969) *Streptomyces* exfoliatus DSMZ 41693

(2570466035) *Streptomyces* sp. HCCB10043

**False positives**

(648008380) *Streptomyces* sp. SPB74

(2598227426) *Streptomyces* sp. AW19M42

Table S3: P450 family and subfamily analysis in *Streptomyces* species.

| Family | Subfamily | P450 count |
| --- | --- | --- |
| CYP1004 | B | 1 |
|  | E | 7 |
|  | F | 7 |
| CYP1005 | B | 17 |
| CYP1012 | B | 1 |
| CYP1013 | A | 3 |
|  | D | 1 |
| CYP1027 | G | 4 |
| CYP1029 | A | 4 |
| CYP102 | B | 78 |
|  | D | 6 |
|  | G | 48 |
| CYP1031 | A | 8 |
| CYP1035 | A | 79 |
| CYP1036 | A | 8 |
| CYP1037 | A | 3 |
|  | B | 6 |
|  | C | 1 |
| CYP1038 | A | 22 |
|  | C | 2 |
|  | D | 1 |
| CYP1039 | A | 1 |
|  | C | 1 |
| CYP1041 | A | 1 |
| CYP1042 | A | 4 |
| CYP1043 | A | 17 |
| CYP1044 | A | 2 |
| CYP1045 | A | 1 |
|  | B | 1 |
| CYP1046 | A | 21 |
| CYP1047 | A | 43 |
|  | B | 3 |
| CYP1048 | A | 6 |
| CYP1050 | B | 1 |
|  | C | 1 |
|  | D | 1 |
| CYP1051 | F | 1 |
| CYP1053 | A | 1 |
| CYP1054 | A | 4 |
| CYP1055 | A | 1 |
| CYP1056 | A | 1 |
|  | B | 1 |
| CYP1057 | A | 1 |
| CYP1058 | A | 1 |
| CYP1059 | A | 2 |
| CYP105 | A | 27 |
|  | AA | 25 |
|  | AB | 14 |
|  | AC | 81 |
|  | AD | 2 |
|  | AE | 2 |
|  | AG | 1 |
|  | AH | 4 |
|  | AJ | 2 |
|  | AK | 42 |
|  | AN | 4 |
|  | AQ | 4 |
|  | AR | 1 |
|  | AT | 1 |
|  | AU | 1 |
|  | AV | 5 |
|  | AW | 1 |
|  | AX | 4 |
|  | AY | 3 |
|  | AZ | 22 |
|  | B | 127 |
|  | BA | 14 |
|  | BB | 2 |
|  | BC | 3 |
|  | BD | 3 |
|  | BE | 3 |
|  | BF | 3 |
|  | BG | 2 |
|  | BH | 1 |
|  | BK | 3 |
|  | BM | 1 |
|  | BN | 3 |
|  | BP | 3 |
|  | BQ | 2 |
|  | BR | 1 |
|  | BS | 1 |
|  | BT | 10 |
|  | BU | 2 |
|  | BV | 3 |
|  | BW | 1 |
|  | CD | 6 |
|  | CF | 4 |
|  | CK | 1 |
|  | CY | 1 |
|  | D | 110 |
|  | DB | 6 |
|  | DC | 1 |
|  | DD | 2 |
|  | DE | 1 |
|  | DF | 1 |
|  | DG | 1 |
|  | DH | 2 |
|  | DK | 1 |
|  | DL | 1 |
|  | DM | 2 |
|  | DN | 1 |
|  | DP | 2 |
|  | DQ | 1 |
|  | DR | 5 |
|  | DS | 2 |
|  | DT | 2 |
|  | DU | 2 |
|  | DV | 1 |
|  | DW | 3 |
|  | DX | 1 |
|  | DY | 6 |
|  | DZ | 1 |
|  | EA | 1 |
|  | EB | 1 |
|  | EC | 1 |
|  | ED | 1 |
|  | EE | 1 |
|  | EF | 1 |
|  | EG | 1 |
|  | EH | 1 |
|  | H | 26 |
|  | K | 1 |
|  | L | 1 |
|  | M | 5 |
|  | N | 20 |
|  | P | 2 |
|  | Q | 6 |
|  | R | 1 |
|  | S | 4 |
|  | U | 2 |
|  | V | 1 |
|  | Z | 6 |
| CYP1060 | A | 12 |
| CYP1061 | A | 3 |
| CYP1062 | A | 5 |
| CYP1063 | A | 1 |
| CYP1064 | A | 22 |
| CYP1065 | A | 1 |
| CYP1066 | A | 1 |
| CYP107 | A | 2 |
|  | AD | 4 |
|  | AE | 48 |
|  | AF | 1 |
|  | AH | 23 |
|  | AK | 2 |
|  | AL | 6 |
|  | AM | 34 |
|  | AQ | 3 |
|  | AT | 10 |
|  | AW | 2 |
|  | B | 10 |
|  | BB | 1 |
|  | BC | 1 |
|  | BJ | 1 |
|  | BK | 3 |
|  | BM | 17 |
|  | BS | 2 |
|  | BT | 5 |
|  | BU | 11 |
|  | BV | 1 |
|  | BW | 4 |
|  | BX | 57 |
|  | BY | 8 |
|  | BZ | 4 |
|  | C | 1 |
|  | CA | 6 |
|  | CD | 4 |
|  | CE | 3 |
|  | CF | 3 |
|  | CG | 4 |
|  | CH | 13 |
|  | CJ | 26 |
|  | CK | 1 |
|  | CL | 2 |
|  | CM | 3 |
|  | CN | 1 |
|  | CP | 1 |
|  | CQ | 2 |
|  | CR | 4 |
|  | CS | 5 |
|  | CT | 4 |
|  | CU | 2 |
|  | CV | 1 |
|  | CW | 2 |
|  | DD | 2 |
|  | DK | 1 |
|  | DU | 2 |
|  | DW | 5 |
|  | E | 60 |
|  | EA | 15 |
|  | EB | 4 |
|  | EC | 3 |
|  | ED | 2 |
|  | EE | 1 |
|  | EF | 1 |
|  | EG | 2 |
|  | EH | 4 |
|  | EJ | 1 |
|  | EK | 1 |
|  | EL | 2 |
|  | EM | 3 |
|  | EN | 2 |
|  | EP | 1 |
|  | EQ | 1 |
|  | ER | 1 |
|  | F | 65 |
|  | FF | 1 |
|  | FH | 2 |
|  | FV | 2 |
|  | G | 4 |
|  | JB | 3 |
|  | JK | 3 |
|  | JP | 1 |
|  | JS | 1 |
|  | JU | 1 |
|  | KT | 1 |
|  | KW | 2 |
|  | KX | 2 |
|  | KY | 2 |
|  | KZ | 1 |
|  | L | 175 |
|  | LA | 1 |
|  | LB | 1 |
|  | LC | 1 |
|  | LD | 1 |
|  | LE | 1 |
|  | LF | 6 |
|  | LG | 1 |
|  | LW | 1 |
|  | LX | 1 |
|  | LY | 1 |
|  | LZ | 1 |
|  | MA | 1 |
|  | MB | 1 |
|  | MC | 2 |
|  | MD | 3 |
|  | ME | 1 |
|  | MF | 1 |
|  | MG | 2 |
|  | MH | 3 |
|  | MJ | 1 |
|  | MK | 2 |
|  | ML | 1 |
|  | MM | 1 |
|  | MN | 1 |
|  | MP | 2 |
|  | MQ | 1 |
|  | MR | 1 |
|  | MS | 1 |
|  | MT | 1 |
|  | MU | 1 |
|  | MV | 3 |
|  | MW | 2 |
|  | MX | 1 |
|  | MY | 1 |
|  | MZ | 1 |
|  | NA | 1 |
|  | NB | 1 |
|  | NC | 1 |
|  | ND | 1 |
|  | P | 148 |
|  | Q | 2 |
|  | R | 4 |
|  | T | 17 |
|  | U | 203 |
|  | V | 1 |
|  | W | 6 |
|  | X | 46 |
|  | Y | 9 |
|  | Z | 16 |
| CYP108 | B | 5 |
|  | N | 2 |
| CYP1095 | A | 1 |
| CYP1112 | A | 8 |
| CYP1113 | A | 3 |
| CYP1115 | A | 1 |
| CYP112 | B | 1 |
| CYP1133 | D | 2 |
| CYP113 | AA | 1 |
|  | AB | 2 |
|  | AC | 1 |
|  | AD | 1 |
|  | AE | 1 |
|  | AF | 4 |
|  | AG | 1 |
|  | B | 2 |
|  | C | 4 |
|  | D | 9 |
|  | F | 1 |
|  | G | 1 |
|  | H | 1 |
|  | J | 2 |
|  | K | 11 |
|  | R | 2 |
|  | V | 1 |
|  | Y | 5 |
|  | Z | 2 |
| CYP1147 | A | 1 |
| CYP1151 | A | 1 |
| CYP116 | B | 1 |
|  | D | 2 |
|  | H | 1 |
| CYP1189 | A | 6 |
| CYP1190 | A | 3 |
| CYP1191 | A | 3 |
| CYP1192 | A | 6 |
| CYP1193 | A | 1 |
| CYP1194 | A | 2 |
| CYP1195 | A | 2 |
| CYP1196 | A | 1 |
| CYP1197 | A | 2 |
| CYP1198 | A | 3 |
| CYP1199 | A | 25 |
|  | B | 1 |
| CYP1200 | A | 5 |
| CYP1207 | A | 7 |
| CYP1215 | A | 6 |
| CYP1216 | A | 3 |
| CYP121 | A | 14 |
| CYP1222 | C | 1 |
|  | D | 1 |
| CYP1223 | A | 2 |
|  | B | 1 |
| CYP1228 | A | 1 |
| CYP122 | A | 3 |
| CYP1231 | A | 1 |
| CYP1232 | C | 2 |
|  | G | 2 |
| CYP1236 | A | 2 |
| CYP1237 | A | 4 |
|  | B | 1 |
|  | C | 1 |
| CYP1238 | A | 7 |
| CYP123 | D | 2 |
|  | H | 1 |
| CYP1240 | A | 3 |
|  | B | 21 |
|  | C | 5 |
| CYP1242 | A | 1 |
| CYP1248 | A | 1 |
| CYP124 | B | 9 |
|  | G | 50 |
|  | N | 1 |
| CYP1251 | A | 3 |
|  | C | 3 |
| CYP1253 | B | 3 |
| CYP125 | A | 104 |
|  | B | 1 |
|  | G | 1 |
|  | L | 1 |
| CYP1265 | A | 1 |
|  | B | 2 |
|  | C | 1 |
| CYP126 | B | 10 |
| CYP1274 | A | 3 |
| CYP1278 | B | 5 |
| CYP1279 | A | 6 |
| CYP1282 | B | 1 |
| CYP129 | B | 3 |
| CYP1301 | A | 2 |
| CYP130 | A | 3 |
| CYP1313 | A | 2 |
| CYP1316 | C | 1 |
| CYP1339 | B | 1 |
| CYP1341 | E | 2 |
|  | F | 2 |
| CYP134 | A | 5 |
| CYP135 | D | 1 |
|  | E | 2 |
|  | G | 1 |
| CYP1369 | A | 1 |
| CYP136 | C | 1 |
|  | D | 2 |
|  | E | 1 |
|  | F | 1 |
| CYP1373 | A | 2 |
| CYP1385 | A | 2 |
| CYP1386 | A | 1 |
|  | B | 1 |
| CYP1392 | A | 1 |
| CYP1394 | A | 1 |
|  | B | 1 |
| CYP139 | C | 1 |
| CYP1408 | B | 1 |
| CYP140 | C | 4 |
| CYP1416 | A | 2 |
|  | B | 1 |
| CYP1417 | A | 9 |
| CYP1418 | A | 1 |
| CYP1419 | A | 11 |
| CYP1420 | A | 3 |
| CYP1422 | A | 3 |
| CYP1423 | A | 3 |
| CYP1424 | A | 3 |
| CYP142 | F | 1 |
| CYP1432 | A | 1 |
| CYP143 | C | 3 |
| CYP1441 | A | 1 |
| CYP1448 | A | 1 |
| CYP144 | E | 1 |
| CYP1453 | B | 5 |
| CYP1455 | A | 1 |
| CYP1457 | B | 5 |
| CYP1459 | A | 1 |
|  | B | 6 |
| CYP145 | B | 7 |
|  | C | 6 |
|  | D | 2 |
|  | H | 1 |
|  | J | 2 |
|  | K | 1 |
| CYP1460 | B | 4 |
| CYP1469 | A | 1 |
|  | B | 3 |
|  | D | 1 |
| CYP146 | A | 8 |
|  | C | 2 |
| CYP147 | A | 2 |
|  | B | 25 |
|  | C | 2 |
|  | F | 73 |
|  | K | 4 |
|  | M | 1 |
| CYP1509 | A | 1 |
| CYP150 | A | 4 |
| CYP1510 | A | 1 |
| CYP1518 | A | 2 |
| CYP151 | A | 4 |
|  | B | 1 |
|  | D | 1 |
| CYP1524 | A | 1 |
| CYP1529 | A | 1 |
| CYP152 | D | 42 |
| CYP1530 | A | 3 |
| CYP1543 | A | 1 |
| CYP154 | A | 127 |
|  | AB | 1 |
|  | AD | 1 |
|  | AH | 2 |
|  | AJ | 4 |
|  | AK | 1 |
|  | AL | 2 |
|  | AM | 2 |
|  | AN | 1 |
|  | AP | 2 |
|  | AQ | 1 |
|  | AR | 1 |
|  | B | 16 |
|  | C | 164 |
|  | D | 76 |
|  | K | 12 |
|  | L | 5 |
|  | M | 12 |
|  | P | 3 |
|  | Q | 6 |
|  | R | 1 |
|  | S | 4 |
|  | T | 25 |
|  | U | 32 |
|  | V | 7 |
|  | Z | 2 |
| CYP155 | A | 19 |
| CYP1562 | A | 3 |
| CYP1568 | A | 1 |
| CYP156 | A | 20 |
|  | B | 120 |
|  | C | 24 |
|  | D | 2 |
|  | E | 5 |
|  | F | 2 |
|  | G | 26 |
|  | H | 20 |
|  | M | 1 |
|  | N | 1 |
|  | Q | 3 |
|  | R | 3 |
|  | S | 7 |
|  | T | 3 |
| CYP1571 | A | 1 |
| CYP1578 | A | 1 |
| CYP157 | A | 174 |
|  | B | 78 |
|  | C | 177 |
|  | F | 17 |
|  | G | 9 |
|  | H | 1 |
|  | J | 26 |
|  | K | 33 |
|  | P | 1 |
|  | V | 1 |
|  | W | 5 |
|  | X | 1 |
|  | Y | 1 |
|  | Z | 1 |
| CYP158 | A | 91 |
|  | B | 2 |
|  | C | 1 |
| CYP159 | A | 125 |
|  | F | 1 |
| CYP1607 | A | 1 |
| CYP1618 | A | 15 |
| CYP161 | A | 10 |
|  | B | 1 |
|  | C | 8 |
|  | D | 1 |
|  | E | 4 |
|  | F | 1 |
|  | G | 1 |
|  | H | 5 |
|  | N | 2 |
|  | NSF | 1 |
|  | S | 2 |
| CYP162 | A | 5 |
|  | B | 3 |
|  | C | 3 |
|  | T | 1 |
| CYP1634 | A | 1 |
| CYP163 | A | 6 |
|  | B | 50 |
|  | C | 8 |
|  | D | 4 |
|  | G | 1 |
|  | H | 3 |
|  | J | 5 |
| CYP164 | C | 1 |
| CYP1658 | A | 1 |
| CYP165 | B | 11 |
|  | E | 11 |
| CYP166 | A | 4 |
|  | C | 1 |
| CYP1694 | A | 3 |
|  | B | 1 |
| CYP170 | A | 57 |
|  | B | 18 |
| CYP171 | A | 3 |
| CYP1722 | A | 3 |
| CYP1759 | A | 1 |
| CYP177 | A | 1 |
|  | F | 1 |
| CYP178 | A | 2 |
|  | B | 3 |
| CYP179 | A | 13 |
|  | B | 1 |
| CYP180 | A | 54 |
|  | B | 22 |
| CYP1810 | A | 1 |
| CYP1813 | A | 18 |
| CYP181 | A | 8 |
| CYP1824 | A | 1 |
| CYP182 | A | 11 |
|  | B | 21 |
|  | C | 3 |
| CYP1832 | A | 2 |
| CYP183 | A | 11 |
|  | AR | 2 |
|  | AS | 1 |
|  | AV | 1 |
|  | AW | 1 |
|  | AX | 1 |
|  | AY | 1 |
|  | AZ | 1 |
|  | B | 9 |
|  | BA | 1 |
|  | BB | 1 |
|  | BC | 1 |
|  | BD | 1 |
|  | C | 1 |
|  | D | 1 |
|  | E | 4 |
|  | F | 3 |
|  | G | 8 |
|  | H | 8 |
|  | J | 10 |
|  | K | 1 |
|  | L | 4 |
|  | M | 4 |
|  | N | 1 |
|  | P | 1 |
|  | Q | 1 |
|  | R | 2 |
|  | S | 1 |
|  | T | 1 |
|  | U | 2 |
|  | V | 1 |
|  | W | 2 |
|  | X | 13 |
|  | Y | 1 |
| CYP184 | A | 33 |
| CYP1859 | A | 1 |
| CYP1866 | A | 1 |
| CYP186 | B | 8 |
|  | D | 1 |
|  | Q | 1 |
| CYP1882 | B | 1 |
| CYP188 | A | 4 |
| CYP1896 | A | 2 |
| CYP189 | A | 1 |
| CYP1914 | A | 1 |
| CYP1917 | A | 1 |
| CYP1920 | A | 1 |
| CYP1928 | A | 1 |
| CYP1929 | A | 1 |
| CYP1931 | A | 1 |
| CYP1940 | A | 1 |
| CYP1941 | A | 1 |
| CYP1943 | A | 1 |
| CYP194 | B | 4 |
| CYP1959 | B | 5 |
|  | C | 1 |
| CYP1972 | A | 4 |
| CYP197 | T | 2 |
| CYP1984 | A | 1 |
| CYP1994 | A | 1 |
| CYP1995 | B | 1 |
|  | C | 1 |
| CYP199 | A | 4 |
|  | R | 1 |
| CYP2006 | A | 1 |
| CYP2018 | B | 1 |
| CYP2027 | A | 5 |
| CYP2035 | A | 1 |
| CYP2045 | A | 2 |
|  | B | 1 |
| CYP204 | D | 2 |
| CYP2054 | A | 1 |
| CYP206 | D | 3 |
| CYP2073 | A | 1 |
| CYP2076 | A | 1 |
| CYP2080 | A | 1 |
|  | B | 1 |
| CYP208 | A | 17 |
| CYP2108 | A | 1 |
| CYP211 | A | 3 |
|  | D | 1 |
|  | F | 3 |
|  | K | 1 |
| CYP2134 | A | 1 |
|  | B | 1 |
| CYP215 | A | 1 |
| CYP217 | A | 1 |
| CYP2180 | A | 1 |
| CYP2189 | A | 1 |
| CYP2238 | A | 1 |
| CYP2266 | A | 3 |
| CYP2286 | A | 1 |
| CYP228 | A | 2 |
| CYP229 | K | 1 |
| CYP2307 | A | 2 |
| CYP2340 | B | 1 |
| CYP2349 | A | 1 |
| CYP2357 | B | 1 |
| CYP2365 | C | 1 |
| CYP2378 | A | 2 |
|  | B | 5 |
|  | C | 2 |
|  | D | 1 |
| CYP238 | B | 3 |
| CYP2427 | A | 3 |
| CYP244 | A | 3 |
| CYP245 | A | 8 |
|  | B | 1 |
| CYP246 | A | 3 |
| CYP247 | A | 8 |
| CYP251 | A | 23 |
|  | B | 1 |
|  | D | 1 |
|  | E | 1 |
|  | F | 3 |
|  | G | 6 |
|  | M | 2 |
|  | R | 1 |
|  | S | 1 |
|  | T | 1 |
|  | U | 1 |
| CYP253 | A | 1 |
|  | B | 1 |
| CYP2540 | A | 1 |
| CYP255 | A | 2 |
| CYP256 | A | 1 |
| CYP268 | A | 1 |
|  | D | 1 |
|  | H | 2 |
|  | NSF | 1 |
| CYP2723 | A | 2 |
| CYP274 | B | 1 |
|  | C | 1 |
| CYP282 | A | 1 |
| CYP283 | A | 5 |
| CYP285 | A | 13 |
|  | B | 3 |
|  | D | 1 |
|  | G | 1 |
|  | H | 1 |
| CYP291 | D | 1 |
| CYP294 | A | 2 |
|  | B | 1 |
| CYP295 | B | 1 |
| CYP298 | B | 1 |
| **253** | **698** | **5460** |

Table S4. Secondary metabolite biosynthetic gene clusters (BGCs) and P450s that are associated with BGCs in *Streptomyces* species. Standard gene cluster abbreviation terminology available at anti-SMASH database [2] was used in the table.

| Specie name | P450 count | BGCs | No. of P450s part of BGCs | P450 name | BGC type |
| --- | --- | --- | --- | --- | --- |
| *Streptomyces coelicolor* | 18 | 27 | 3 | CYP158A2 | T3pks |
|  |  |  |  | CYP170A1 | Terpene |
|  |  |  |  | CYP105N1 | T3pks-Terpene-Nrps |
| *Streptomyces avermitilis* MA-4680 | 52 | 37 | 17 | CYP105D6 | T1pks |
|  |  |  |  | CYP105P1 | T1pks |
|  |  |  |  | CYP147B1 | Nrps |
|  |  |  |  | CYP178A1 | Nrps-T1pks-Otherks |
|  |  |  |  | CYP178A3P | Nrps-T1pks-Otherks |
|  |  |  |  | CYP171A1 | T1pks |
|  |  |  |  | CYP180A1 | Terpene |
|  |  |  |  | CYP107Y1 | T2pks-T1pks-Otherks |
|  |  |  |  | CYP181A1 | T2pks-T1pks-Otherks |
|  |  |  |  | CYP107W1 | T1pks |
|  |  |  |  | CYP105B23 | T1pks |
|  |  |  |  | CYP183A1 | Terpene |
|  |  |  |  | CYP170A2 | Terpene |
|  |  |  |  | CYP107V1 | Butyrolactone-Otherks |
|  |  |  |  | CYP107U2 | Butyrolactone-Otherks |
|  |  |  |  | CYP158A3 | T3pks |
|  |  |  |  | CYP105R1 | T1pks |
| *Streptomyces griseus* | 28 | 36 | 11 | CYP105D1 | T1pks-Nrps |
|  |  |  |  | CYP124G2 | Melanin |
|  |  |  |  | CYP162C1 | T1pks-Nrps |
|  |  |  |  | CYP208A1 | T1pks-Nrps |
|  |  |  |  | CYP154M2 | T1pks-Nrps |
|  |  |  |  | CYP107BX5 | T1pks-Nrps |
|  |  |  |  | CYP107BY1 | Nrps |
|  |  |  |  | CYP163B5 | Ladderane-Arylpolyene-Nrps |
|  |  |  |  | CYP107BZ1 | Ladderane-Arylpolyene-Nrps |
|  |  |  |  | CYP107F4 | T3pks |
|  |  |  |  | CYP107CA2 | Transatpks-T1pks-Otherks-Nrps |
| *Streptomyces globisporus* | 23 | 27 | 5 | CYP107F4 | T3pks |
|  |  |  |  | CYP1373A2 | Arylpolyene-Ladderane |
|  |  |  |  | CYP107BX10 | Bacteriocin-T1pks-Nrps |
|  |  |  |  | CYP124G14 | Melanin |
|  |  |  |  | CYP105D30 | T1pks-Nrps |
| *Streptomyces scabiei* 87.22 | 30 | 32 | 11 | CYP154A4 | Terpene |
|  |  |  |  | CYP246A1 | Lantipeptide-Nrps |
|  |  |  |  | CYP1048A1 | Lantipeptide-Nrps |
|  |  |  |  | CYP156D1 | Lantipeptide-Nrps |
|  |  |  |  | CYP154L1 | Lantipeptide-Nrps |
|  |  |  |  | CYP107AM1 | T1pks-Nrps |
|  |  |  |  | CYP283A1 | Bacteriocin-Bottromycin |
|  |  |  |  | CYP107AL1 | Butyrolactone-T1pks-Otherks |
|  |  |  |  | CYP157C5 | Terpene |
|  |  |  |  | CYP156B2 | Indole-T1pks |
|  |  |  |  | CYP107AK1 | T1pks |
| *Streptomyces* sp. Sirex AA-E | 24 | 22 | 8 | CYP124G3 | Melanin |
|  |  |  |  | CYP105N1 | Nrps |
|  |  |  |  | CYP181A1 | T2pks-T1pks-Otherks |
|  |  |  |  | CYP107Y1 | T2pks-T1pks-Otherks |
|  |  |  |  | CYP105AZ1 | T1pks |
|  |  |  |  | CYP105AZ2 | T1pks |
|  |  |  |  | CYP107BX4 | T1pks-Nrps |
|  |  |  |  | CYP105A4 | T2pks-Butyrolactone-Nrps |
| *Streptomyces violaceusniger* Tu 4113 | 50 | 41 | 24 | CYP107BW1 | Terpene |
|  |  |  |  | CYP1013A2 | Terpene |
|  |  |  |  | CYP162A3 | Nrps |
|  |  |  |  | CYP107CK1 | Nrps |
|  |  |  |  | CYP124B3 | T1pks |
|  |  |  |  | CYP107BW1 | T1pks |
|  |  |  |  | CYP105AX1 | T1pks |
|  |  |  |  | CYP183F2; | Otherks |
|  |  |  |  | CYP105AV1 | Otherks |
|  |  |  |  | CYP105AQ2 | Bacteriocin-Lantipeptide-T1pks-Otherks-Nrps |
|  |  |  |  | CYP155A5 | Bacteriocin-Lantipeptide-T1pks-Otherks-Nrps |
|  |  |  |  | CYP107E9 | Bacteriocin-Lantipeptide-T1pks-Otherks-Nrps |
|  |  |  |  | CYP105AN3 | Bacteriocin-Lantipeptide-T1pks-Otherks-Nrps |
|  |  |  |  | CYP107AD1 | T1pks |
|  |  |  |  | CYP105AY1 | Lantipeptide-T1pks-Nrps |
|  |  |  |  | CYP105U1 | T1pks |
|  |  |  |  | CYP156C9 | Terpene |
|  |  |  |  | CYP125A20 | Terpene |
|  |  |  |  | CYP107U9 | Indole |
|  |  |  |  | CYP156B6 | Indole |
|  |  |  |  | CYP163B4 | Ladderane-Arylpolyene-Nrps |
|  |  |  |  | CYP107CF1 | Ladderane-Arylpolyene-Nrps |
|  |  |  |  | CYP107CE1 | Ladderane-Arylpolyene-Nrps |
|  |  |  |  | CYP147F5 | Terpene |
| *Streptomyces cattleya* NRRL 8057 | 40 | 24 | 10 | CYP107AS | T1pks |
|  |  |  |  | CYP107CR1 | T1pks |
|  |  |  |  | CYP107AE6 | Lantipeptide |
|  |  |  |  | CYP184A4 | T1pks-Nrps |
|  |  |  |  | CYP107CS1 | Transatpks-T1pks-Nrps |
|  |  |  |  | CYP107W2 | Transatpks-T1pks-Nrps |
|  |  |  |  | CYP158A13 | T3pks-Terpene |
|  |  |  |  | CYP105AA10 | Lantipeptide |
|  |  |  |  | CYP107CT1 | T1pks-Butyrolactone-Nrps |
|  |  |  |  | CYP105B25 | T1pks-Butyrolactone-Nrps |
| *Streptomyces cattleya* NRRL 8058 = DSM 46488 | 41 | 25 | 11 | CYP107AS | T1pks |
|  |  |  |  | CYP1274A1 | T1pks |
|  |  |  |  | CYP107CR1 | T1pks |
|  |  |  |  | CYP107AE6 | Lantipeptide |
|  |  |  |  | CYP184A4 | T1pks-Nrps |
|  |  |  |  | CYP107CS1 | Transatpks-T1pks-Nrps |
|  |  |  |  | CYP107W2 | Transatpks-T1pks-Nrps |
|  |  |  |  | CYP158A13 | T3pks-Terpene |
|  |  |  |  | CYP105AA10 | Lantipeptide |
|  |  |  |  | CYP107CT1 | T1pks-Butyrolactone-Nrps |
|  |  |  |  | CYP105B25 | T1pks-Butyrolactone-Nrps |
| *Streptomyces pratensis/flavogriseus* IAF 45 | 29 | 26 | 11 | CYP247A3 | Blactam-T1pks-Nrps |
|  |  |  |  | CYP107BX5 | Blactam-T1pks-Nrps |
|  |  |  |  | CYP105AZ2 | T1pks |
|  |  |  |  | CYP105AZ1 | T1pks |
|  |  |  |  | CYP1029A2 | T1pks-Nrps |
|  |  |  |  | CYP1423A2 | T1pks-Nrps |
|  |  |  |  | CYP285A2 | T1pks-Nrps |
|  |  |  |  | CYP157K1 | Terpene |
|  |  |  |  | CYP124G4 | Melanin |
|  |  |  |  | CYP1035A4 | Nrps |
|  |  |  |  | CYP156B9 | Nrps |
| *Streptomyces bingchenggensis* | 49 | 47 | 17 | CYP183C1 | Bacteriocin-T1pks-Nrps |
|  |  |  |  | CYP183D1 | Bacteriocin-T1pks-Nrps |
|  |  |  |  | CYP183E1 | Terpene |
|  |  |  |  | CYP1039A1 | Bacteriocin-Lantipeptide-T1pks |
|  |  |  |  | CYP105H6 | Transatpks-T1pks-Nrps |
|  |  |  |  | CYP107BK1 | Transatpks-T1pks-Nrps |
|  |  |  |  | CYP171A2 | Transatpks-T1pks-Nrps |
|  |  |  |  | CYP107BM3 | Nrps |
|  |  |  |  | CYP157B13 | Nrps |
|  |  |  |  | CYP113G1 | T1pks-Nrps |
|  |  |  |  | CYP154P1 | Other |
|  |  |  |  | CYP1037A1 | T1pks-Nrps |
|  |  |  |  | CYP268A4 | Otherks |
|  |  |  |  | CYP124B2 | T1pks |
|  |  |  |  | CYP163C1 | Otherks-Nrps |
|  |  |  |  | CYP161C1 | Transatpks-Terpene-Nrps |
|  |  |  |  | CYP183A2 | Transatpks-Terpene-Nrps |
| *Streptomyces hygroscopicus* subsp. *jinggangensis* 5008 | 38 | 38 | 10 | CYP105B22 | T1pks |
|  |  |  |  | CYP107X1 | Terpene |
|  |  |  |  | CYP163B6 | Nrps |
|  |  |  |  | CYP285B1 | Nrps |
|  |  |  |  | CYP105AZ2 | T1pks |
|  |  |  |  | CYP105AZ1 | T1pks |
|  |  |  |  | CYP158A14 | T3pks |
|  |  |  |  | CYP170A10 | Terpene |
|  |  |  |  | CYP180A6 | Terpene |
|  |  |  |  | CYP113K3 | Bacteriocin-Nrps |
| *Streptomyces hygroscopicus* subsp. *jinggangensis* TL01 | 37 | 38 | 10 | CYP105B22 | T1pks |
|  |  |  |  | CYP107X1 | Terpene |
|  |  |  |  | CYP163B6 | Nrps |
|  |  |  |  | CYP285B1 | Nrps |
|  |  |  |  | CYP105AZ2 | T1pks |
|  |  |  |  | CYP105AZ1 | T1pks |
|  |  |  |  | CYP158A14 | T3pks |
|  |  |  |  | CYP170A10 | Terpene |
|  |  |  |  | CYP180A6 | Terpene |
|  |  |  |  | CYP113K3 | Bacteriocin-Nrps |
| *Streptomyces venezuelae* | 23 | 30 | 10 | CYP157C14 | Lantipeptide-Terpene |
|  |  |  |  | CYP245A3 | Indole |
|  |  |  |  | CYP121A2 | Other |
|  |  |  |  | CYP158A5 | T3pks |
|  |  |  |  | CYP105AC2 | Other |
|  |  |  |  | CYP180A5 | Other |
|  |  |  |  | CYP1056A1 | Ladderane-Nrps |
|  |  |  |  | CYP107CL1 | Ladderane-Nrps |
|  |  |  |  | CYP162A4 | Ladderane-Nrps |
|  |  |  |  | CYP163B5 | Ladderane-Nrps |
| *Streptomyces davawensis* | 32 | 31 | 17 | CYP105BA1 | T1pks-Nrps |
|  |  |  |  | CYP1005B2 | Other |
|  |  |  |  | CYP179B1 | Bacteriocin-Lantipeptide |
|  |  |  |  | CYP183K1 | Terpene |
|  |  |  |  | CYP179A3 | Lantipeptide-T1pks-Nrps |
|  |  |  |  | CYP180A6 | Butyrolactone-Terpene |
|  |  |  |  | CYP107CN1 | Bacteriocin-Oligosaccharide |
|  |  |  |  | CYP107CP1 | Bacteriocin-Oligosaccharide |
|  |  |  |  | CYP170A9 | Terpene-Nrps |
|  |  |  |  | CYP113J1 | T1pks-Nrps |
|  |  |  |  | CYP113J2 | T1pks-Nrps |
|  |  |  |  | CYP162A5 | T1pks-Nrps |
|  |  |  |  | CYP125A22 | Otherks-Nrps |
|  |  |  |  | CYP163C2 | Otherks-Nrps |
|  |  |  |  | CYP158A7 | T3pks |
|  |  |  |  | CYP1041A2 | Terpene-T3pks-Cyanobactin-Nrps |
|  |  |  |  | CYP1058A1 | Terpene-T3pks-Cyanobactin-Nrps |
| *Streptomyces albus* J1074 | 18 | 22 | 7 | CYP107BX2 | T1pks-Nrps |
|  |  |  |  | CYP1420A1 | Otherks |
|  |  |  |  | CYP146A3 | Nrps |
|  |  |  |  | CYP170B5 | Terpene |
|  |  |  |  | CYP154A1 | Lantipeptide |
|  |  |  |  | CYP107F4 | T3pks |
|  |  |  |  | CYP105H3 | Lantipeptide-T1pks-Nrps |
| *Streptomyces albus* DSM 41398 | 25 | 35 | 10 | CYP105BK3 | Nrps |
|  |  |  |  | CYP107EJ1 | T1pks-Butyrolactone-Nrps |
|  |  |  |  | CYP113Y1 | T1pks-Otherks |
|  |  |  |  | CYP105DB1 | T1pks-Otherks |
|  |  |  |  | CYP107DU1 | T1pks |
|  |  |  |  | CYP170B5 | Terpene |
|  |  |  |  | CYP107T3 | Arylpolyene-Nrps |
|  |  |  |  | CYP107KW1 | Arylpolyene |
|  |  |  |  | CYP1193A1 | T2pks-Otherks |
|  |  |  |  | CYP1194A1 | T1pks-Nrps |
| *Streptomyces* sp. PAMC 26508 | 29 | 28 | 12 | CYP156B9 | Nrps |
|  |  |  |  | CYP1035A4 | Nrps |
|  |  |  |  | CYP124G4 | Melanin |
|  |  |  |  | CYP157K1 | Terpene |
|  |  |  |  | CYP285A2 | T1pks-Nrps |
|  |  |  |  | CYP1423A2 | T1pks-Nrps |
|  |  |  |  | CYP1029A2 | T1pks-Nrps |
|  |  |  |  | CYP105AZ1 | T1pks |
|  |  |  |  | CYP105AZ2 | T1pks |
|  |  |  |  | CYP1057A1 | Bacteriocin-Otherks |
|  |  |  |  | CYP107BX5 | Blactam-T1pks-Nrps |
|  |  |  |  | CYP247A3 | Blactam-T1pks-Nrps |
| *Streptomyces fulvissimus* | 19 | 34 | 3 | CYP107F4 | T3pks |
|  |  |  |  | CYP107BX3 | T1pks-Nrps |
|  |  |  |  | CYP124G2 | Melanin |
| *Streptomyces collinus* | 34 | 32 | 13 | CYP107CQ1 | Transatpks-T1pks-Nrps |
|  |  |  |  | CYP105AJ2 | Transatpks-T1pks-Nrps |
|  |  |  |  | CYP1059A1 | Bacteriocin |
|  |  |  |  | CYP105B21 | Bacteriocin |
|  |  |  |  | CYP158A2 | T3pks |
|  |  |  |  | CYP170A10 | Terpene |
|  |  |  |  | CYP105BC1 | T1pks |
|  |  |  |  | CYP105AH2 | T1pks |
|  |  |  |  | CYP180A6 | Terpene |
|  |  |  |  | CYP183A4 | Terpene |
|  |  |  |  | CYP113K3 | Bacteriocin-Nrps |
|  |  |  |  | CYP105AJ2 | Transatpks-T1pks-Nrps |
|  |  |  |  | CYP107CQ1 | Transatpks-T1pks-Nrps |
| *Streptomyces rapamycinicus* | 63 | 47 | 30 | CYP105AX1 | T1pks |
|  |  |  |  | CYP124B3 | T1pks |
|  |  |  |  | CYP161D1 | T1pks-Nrps |
|  |  |  |  | CYP105AT1 | T1pks-Nrps |
|  |  |  |  | CYP107L12 | T1pks-Nrps |
|  |  |  |  | CYP1013A2 | Terpene |
|  |  |  |  | CYP105AU1 | T1pks-Nrps |
|  |  |  |  | CYP147F5 | Terpene |
|  |  |  |  | CYP107CE1 | Nrps-Arylpolyene-Ladderane |
|  |  |  |  | CYP107CF1 | Nrps-Arylpolyene-Ladderane |
|  |  |  |  | CYP163B4 | Nrps-Arylpolyene-Ladderane |
|  |  |  |  | CYP156B6 | Indole |
|  |  |  |  | CYP107U9 | Indole |
|  |  |  |  | CYP125A20 | Terpene |
|  |  |  |  | CYP156C8 | Terpene |
|  |  |  |  | CYP194B3 | T1pks |
|  |  |  |  | CYP194B4 | T1pks |
|  |  |  |  | CYP107BS2 | T1pks |
|  |  |  |  | CYP105AW1 | T1pks-Nrps |
|  |  |  |  | CYP122A4 | T1pks-Nrps |
|  |  |  |  | CYP107G2 | T1pks-Nrps |
|  |  |  |  | CYP183F1 | Otherks |
|  |  |  |  | CYP107B3 | T1pks-Arylpolyene-Ladderane |
|  |  |  |  | CYP105AY1 | T1pks-Nrps |
|  |  |  |  | CYP107AD1 | T1pks |
|  |  |  |  | CYP105AN3 | Bacteriocin-Nrps-Lantipeptide-T1pks-Otherks |
|  |  |  |  | CYP107E9 | Bacteriocin-Nrps-Lantipeptide-T1pks-Otherks |
|  |  |  |  | CYP155A5 | Bacteriocin-Nrps-Lantipeptide-T1pks-Otherks |
|  |  |  |  | CYP105AV1 | Terpene |
|  |  |  |  | CYP107CD1 | T1pks |
| *Streptomyces albulus NK660* | 64 | 33 | 20 | CYP107L43 | Butyrolactone |
|  |  |  |  | CYP1190A1 | Other |
|  |  |  |  | CYP1192A1 | Other |
|  |  |  |  | CYP1191A1 | T1pks-Nrps |
|  |  |  |  | CYP107EB1 | Nrps |
|  |  |  |  | CYP163B9 | Transatpks-T1pks-Nrps |
|  |  |  |  | CYP105AA13 | Transatpks-T1pks-Nrps |
|  |  |  |  | CYP105H9 | T1pks |
|  |  |  |  | CYP161A7 | T1pks |
|  |  |  |  | CYP107B6 | Transatpks-Nrps |
|  |  |  |  | CYP1189A1 | Transatpks-Nrps |
|  |  |  |  | CYP1189A2 | Transatpks-Nrps |
|  |  |  |  | CYP107F9 | T3pks-Otherks-Butyrolactone-Nrps |
|  |  |  |  | CYP163C3 | T3pks-Otherks-Butyrolactone-Nrps |
|  |  |  |  | CYP113D6 | T2pks-Oligosaccharide-Nucleoside-Nrps |
|  |  |  |  | CYP157C28 | T2pks-Oligosaccharide-Nucleoside-Nrps |
|  |  |  |  | CYP107AE9 | Butyrolactone |
|  |  |  |  | CYP251G1 | Lantipeptide |
|  |  |  |  | CYP161A6 | Terpene-T1pks |
|  |  |  |  | CYP105H1 | Terpene-T1pks |
| *Streptomyces albulus ZPM* | 68 | 35 | 25 | CYP107EL1 | Nrps |
|  |  |  |  | CYP163B8 | Nrps |
|  |  |  |  | CYP107EA2 | Nrps |
|  |  |  |  | CYP107L43 | Butyrolactone |
|  |  |  |  | CYP1190A1 | Other |
|  |  |  |  | CYP147F21 | Other |
|  |  |  |  | CYP1060A2 | Other |
|  |  |  |  | CYP1192A1 | Other |
|  |  |  |  | CYP1191A1 | T1pks |
|  |  |  |  | CYP163B9 | Transatpks-T1pks-Nrps |
|  |  |  |  | CYP105AA13 | Transatpks-T1pks-Nrps |
|  |  |  |  | CYP105H9 | T1pks |
|  |  |  |  | CYP1189A1 | Transatpks-Nrps |
|  |  |  |  | CYP1189A2 | Transatpks-Nrps |
|  |  |  |  | CYP107F9 | T3pks |
|  |  |  |  | CYP163C3 | Otherks-Butyrolactone-Nrps |
|  |  |  |  | CYP113D6 | T2pks-Oligosaccharide-Nucleoside-Nrps |
|  |  |  |  | CYP157C28 | T2pks-Oligosaccharide-Nucleoside-Nrps |
|  |  |  |  | CYP107AE9 | Butyrolactone |
|  |  |  |  | CYP251G1 | Lantipeptide |
|  |  |  |  | CYP161A6 | T1pks |
|  |  |  |  | CYP105H1 | T1pks |
|  |  |  |  | CYP107EA2 | Nrps |
|  |  |  |  | CYP163B8 | Nrps |
|  |  |  |  | CYP107EL1 | Nrps |
| *Streptomyces lividans* | 20 | 27 | 3 | CYP105N1 | T3pks-Terpene-Nrps |
|  |  |  |  | CYP170A1 | Terpene |
|  |  |  |  | CYP158A2 | T3pks |
| *Streptomyces glaucescens* | 18 | 25 | 7 | CYP178B1 | Other |
|  |  |  |  | CYP163B10 | Nrps |
|  |  |  |  | CYP170A22 | Terpene |
|  |  |  |  | CYP180A9 | Terpene |
|  |  |  |  | CYP107AH4 | T2pks |
|  |  |  |  | CYP157K3 | Terpene |
|  |  |  |  | CYP113K5 | Nrps |
| *Streptomyces vietnamensis* | 30 | 27 | 7 | CYP121A2 | Other |
|  |  |  |  | CYP1029A3 | T2pks-Nrps |
|  |  |  |  | CYP1423A1 | T2pks-Nrps |
|  |  |  |  | CYP285A5 | T2pks-Nrps |
|  |  |  |  | CYP107LD1 | Lantipeptide |
|  |  |  |  | CYP154C11 | T2pks-Lantipeptide-Terpene |
|  |  |  |  | CYP157A19 | T2pks-Lantipeptide-Terpene |
| *Streptomyces* sp. 769 | 59 | 36 | 21 | CYP105BA3 | Butyrolactone-T1pks-Nrps |
|  |  |  |  | CYP107EM1 | T1pks-Arylpolyene |
|  |  |  |  | CYP1198A1 | T1pks-Arylpolyene |
|  |  |  |  | CYP105BV1 | T1pks-Arylpolyene |
|  |  |  |  | CYP157C31 | Terpene |
|  |  |  |  | CYP105H1 | T1pks |
|  |  |  |  | CYP161A6 | T1pks |
|  |  |  |  | CYP107AE10 | Butyrolactone |
|  |  |  |  | CYP107F9 | T3pks |
|  |  |  |  | CYP105DE1 | T1pks |
|  |  |  |  | CYP105BS1 | T1pks |
|  |  |  |  | CYP1197A1 | T1pks |
|  |  |  |  | CYP184A8 | Thiopeptide-T1pks |
|  |  |  |  | CYP1196A1 | Nrps |
|  |  |  |  | CYP107CA2 | Transatpks-Nrps |
|  |  |  |  | CYP154D14 | Transatpks-Nrps |
|  |  |  |  | CYP107L42 | Lantipeptide |
|  |  |  |  | CYP163B | Lantipeptide-Nrps |
|  |  |  |  | CYP1278B1 | Lantipeptide-Nrps |
|  |  |  |  | CYP105H8 | Terpene-T1pks |
|  |  |  |  | CYP105BA3 | T1pks-Butyrolactone-Nrps |
| *Streptomyces cyaneogriseus* | 30 | 29 | 9 | CYP107L35 | T1pks |
|  |  |  |  | CYP105BT1 | T1pks |
|  |  |  |  | CYP170A17 | Terpene |
|  |  |  |  | CYP107W3 | T1pks |
|  |  |  |  | CYP105AC8 | Siderophore |
|  |  |  |  | CYP161C4 | Terpene |
|  |  |  |  | CYP183A4 | Terpene |
|  |  |  |  | CYP157K4 | Terpene |
|  |  |  |  | CYP105AC17 | T3pks-Fused-Nrps |
| *Streptomyces xiamenensis* 318 | 19 | 20 | 7 | CYP157C32 | Terpene |
|  |  |  |  | CYP1029A4 | T1pks-Nrps |
|  |  |  |  | CYP285A6 | T1pks-Nrps |
|  |  |  |  | CYP1223B1 | Lantipeptide-Linaridin |
|  |  |  |  | CYP107LF1 | Linaridin-T1pks-Lassopeptide-Nrps |
|  |  |  |  | CYP183W1 | Terpene |
|  |  |  |  | CYP107F11 | T3pks-Terpene-Nrps |
| *Streptomyces* sp. Mg1 | 37 | 23 | 11 | CYP154Q2 | Terpene-Nrps |
|  |  |  |  | CYP157C26 | Lantipeptide-Terpene |
|  |  |  |  | CYP134A3 | Lantipeptide-Terpene |
|  |  |  |  | CYP105DF1 | Other |
|  |  |  |  | CYP2238A1 | Thiopeptide |
|  |  |  |  | CYP1048A3 | Thiopeptide |
|  |  |  |  | CYP154D15 | Terpene-Otherks |
|  |  |  |  | CYP157C27 | Terpene-Otherks |
|  |  |  |  | CYP105L3 | T1pks |
|  |  |  |  | CYP1995C1 | T1pks |
|  |  |  |  | CYP251F1 | Terpene |
| *Streptomyces* sp. CNQ-509 | 16 | 28 | 12 | CYP105AC15 | T1pks-Transatpks-Terpene |
|  |  |  |  | CYP107LC1 | Nrps |
|  |  |  |  | CYP105BK2 | Nrps |
|  |  |  |  | CYP1064A5 | T2pks |
|  |  |  |  | CYP1341E2 | Fused |
|  |  |  |  | CYP157A20 | T3pks |
|  |  |  |  | CYP154C13 | T3pks |
|  |  |  |  | CYP123D1 | T3pks-Terpene-Otherks |
|  |  |  |  | CYP105BA2 | T1pks-Nrps |
|  |  |  |  | CYP165E2; | T3pks-Nrps |
|  |  |  |  | CYP165B8 | T3pks-Nrps |
|  |  |  |  | CYP157C32 | Terpene |
| *Streptomyces ambofaciens* | 19 | 26 | 9 | CYP154K2 | T2pks-Butyrolactone |
|  |  |  |  | CYP156B15 | Indole |
|  |  |  |  | CYP170A1 | Terpene |
|  |  |  |  | CYP107EP1 | Oligosaccharide-T1pks-Nrps |
|  |  |  |  | CYP113B4 | Oligosaccharide-T1pks-Nrps |
|  |  |  |  | CYP157K5 | Terpene |
|  |  |  |  | CYP107EF1 | T1pks |
|  |  |  |  | CYP107EP1 | T1pks |
|  |  |  |  | CYP154K2 | T2pks-Butyrolactone |
| *Streptomyces pristinaespiralis* HCCB 10218 | 23 | 21 | 8 | CYP154A22 | Oligosaccharide-Ectoine-T2pks-Nrps-T1pks-Otherks |
|  |  |  |  | CYP107EH1 | Oligosaccharide-Ectoine-T2pks-Nrps-T1pks-Otherks |
|  |  |  |  | CYP113C2 | Oligosaccharide-Ectoine-T2pks-Nrps-T1pks-Otherks |
|  |  |  |  | CYP124G6 | Melanin |
|  |  |  |  | CYP154B6 | T2pks-Oligosaccharide-Nrps-Otherks |
|  |  |  |  | CYP113C2 | T2pks-Oligosaccharide-Nrps-Otherks |
|  |  |  |  | CYP107EH1 | T2pks-Oligosaccharide-Nrps-Otherks |
|  |  |  |  | CYP154A22 | T1pks-Ectoine-Otherks |
| *Streptomyces* sp. CFMR 7 | 24 | 38 | 5 | CYP124G2 | Melanin |
|  |  |  |  | CYP107BX7 | Bacteriocin-T1pks-Nrps |
|  |  |  |  | CYP154A18 | Nrps |
|  |  |  |  | CYP107F4 | T3pks |
|  |  |  |  | CYP105D20 | Arylpolyene |
| *Streptomyces* sp. CdTB01 | 26 | 32 | 5 | CYP145C3 | Indole |
|  |  |  |  | CYP180A29 | Terpene |
|  |  |  |  | CYP170A19 | Terpene |
|  |  |  |  | CYP157C39 | Terpene |
|  |  |  |  | CYP183B5 | Terpene |
| *Streptomyces reticuli* | 47 | 26 | 12 | CYP166A3 | T1pks |
|  |  |  |  | CYP113K6 | Bacteriocin-Nrps |
|  |  |  |  | CYP162B2 | Other |
|  |  |  |  | CYP121A5 | Other |
|  |  |  |  | CYP154U7 | Other |
|  |  |  |  | CYP158A14 | T2pks-T3pks-Otherks |
|  |  |  |  | CYP107LB1 | Nrps |
|  |  |  |  | CYP105BK2 | Nrps |
|  |  |  |  | CYP1037A2 | Butyrolactone-Amglyccycl-T1pks-Nrps |
|  |  |  |  | CYP170A23 | Terpene |
|  |  |  |  | CYP2045A1 | Nrps |
|  |  |  |  | CYP180A28 | Terpene |
| *Streptomyces* sp. 4F | 16 | 19 | 3 | CYP105DD1 | Nrps-T2pks-Otherks-T1pks-Phenazine |
|  |  |  |  | CYP170A16 | Terpene |
|  |  |  |  | CYP105DD1 | T2pks-Otherks |
| *Streptomyces leeuwenhoekii* C34(2013) | 36 | 31 | 23 | CYP107L35 | T3pks-T1pks-Nrps |
|  |  |  |  | CYP105BT1 | T3pks-T1pks-Nrps |
|  |  |  |  | CYP154B4 | T3pks-T1pks-Nrps |
|  |  |  |  | CYP157K4 | Terpene |
|  |  |  |  | CYP102B20 | Lassopeptide |
|  |  |  |  | CYP113K3 | Nrps |
|  |  |  |  | CYP107EG1 | Nrps-Transatpks-Terpene-Otherks |
|  |  |  |  | CYP105BR1 | Nrps-Transatpks-Terpene-Otherks |
|  |  |  |  | CYP107Q3 | Terpene-T1pks |
|  |  |  |  | CYP105D28 | Terpene-T1pks |
|  |  |  |  | CYP166A2 | Terpene-T1pks |
|  |  |  |  | CYP154Z1 | T1pks |
|  |  |  |  | CYP1416A1 | T1pks |
|  |  |  |  | CYP2266A2 | T1pks |
|  |  |  |  | CYP183A4 | Terpene |
|  |  |  |  | CYP161C4 | Terpene |
|  |  |  |  | CYP107L33 | T1pks-Siderophore |
|  |  |  |  | CYP105AC8 | T1pks-Siderophore |
|  |  |  |  | CYP107F10 | T3pks |
|  |  |  |  | CYP170A17 | Terpene |
|  |  |  |  | CYP1418A1 | T1pks |
|  |  |  |  | CYP1031A3 | T1pks |
|  |  |  |  | CYP107AM11 | Other |
| *Streptomyces rubrolavendulae* | 20 | 24 | 14 | CYP251A3 | Terpene |
|  |  |  |  | CYP157C21 | Terpene |
|  |  |  |  | CYP158A20 | Terpene |
|  |  |  |  | CYP245A6 | Indole |
|  |  |  |  | CYP244A3 | Indole |
|  |  |  |  | CYP159A23 | Nrps |
|  |  |  |  | CYP157B32 | Nrps |
|  |  |  |  | CYP183H3 | Thiopeptide |
|  |  |  |  | CYP183G3 | Thiopeptide |
|  |  |  |  | CYP251F2 | Terpene |
|  |  |  |  | CYP1207A10 | Lantipeptide-Nrps |
|  |  |  |  | CYP158A19 | T3pks-Butyrolactone |
|  |  |  |  | CYP107E12 | Nrps |
|  |  |  |  | CYP285D1 | Nrps |
| *Streptomyces parvulus* | 25 | 21 | 5 | CYP156B15 | Indole |
|  |  |  |  | CYP183J3 | Indole |
|  |  |  |  | CYP170A1 | Terpene |
|  |  |  |  | CYP157K6 | Terpene |
|  |  |  |  | CYP105D33 | Terpene |
| *Streptomyces* sp. SAT1 | 25 | 27 | 11 | CYP1618A1 | Phosphonate-Nrps |
|  |  |  |  | CYP180B6 | Thiopeptide-Terpene |
|  |  |  |  | CYP105B73 | Butyrolactone |
|  |  |  |  | CYP183X4 | Terpene |
|  |  |  |  | CYP158A25 | T3pks |
|  |  |  |  | CYP107P31 | Other |
|  |  |  |  | CYP170A18 | Terpene |
|  |  |  |  | CYP158A24 | Bacteriocin-T1pks |
|  |  |  |  | CYP107E32 | Phosphoglycolipid |
|  |  |  |  | CYP1722A3 | Bacteriocin-Terpene-Nrps |
|  |  |  |  | CYP1618A2 | Bacteriocin-Terpene-Nrps |
| *Streptomyces clavuligerus* | 64 | 26 | 11 | CYP105BG1 | Terpene-T1pks-Nrps |
|  |  |  |  | CYP163B7 | Terpene-T1pks-Nrps |
|  |  |  |  | CYP251E1 | Terpene-T1pks-Nrps |
|  |  |  |  | CYP107F7 | T3pks |
|  |  |  |  | CYP107NSF1 | Indole-Terpene-Nrps |
|  |  |  |  | CYP107BY2 | Indole-Terpene-Nrps |
|  |  |  |  | CYP136E1 | T1pks-Nrps |
|  |  |  |  | CYP105M1 | Blactam-Nrps |
|  |  |  |  | CYP124G5 | Melanin |
|  |  |  |  | CYP107AL2 | T1pks-Butyrolactone-Otherks |
|  |  |  |  | CYP154A14 | T1pks |
| *Streptomyces griseochromogenes* | 46 | 49 | 12 | CYP113V2 | Thiopeptide-Bacteriocin |
|  |  |  |  | CYP170A10 | Terpene |
|  |  |  |  | CYP247A3 | T1pks-Nrps |
|  |  |  |  | CYP105BC2 | T1pks |
|  |  |  |  | CYP180A26 | Terpene |
|  |  |  |  | CYP208A9 | Lantipeptide-T1pks-Nrps |
|  |  |  |  | CYP107BK3 | Lantipeptide-T1pks-Nrps |
|  |  |  |  | CYP107KX1 | Nrps |
|  |  |  |  | CYP121A4 | Other |
|  |  |  |  | CYP154U6 | Other |
|  |  |  |  | CYP183X2 | Terpene |
|  |  |  |  | CYP158A21 | Phosphonate-T3pks-Nrps-Ladderane |
| *Streptomyces* sp. S10(2016) | 20 | 27 | 4 | CYP154K3 | Butyrolactone |
|  |  |  |  | CYP170A15 | Terpene |
|  |  |  |  | CYP163B15 | Nrps |
|  |  |  |  | CYP113Z1 | Nrps |
| *Streptomyces lincolnensis* | 24 | 34 | 9 | CYP105B41 | Ladderane |
|  |  |  |  | CYP163A5 | Nrps |
|  |  |  |  | CYP1424A1 | Nrps |
|  |  |  |  | CYP158A15 | T3pks |
|  |  |  |  | CYP183Y1 | Terpene |
|  |  |  |  | CYP157C22 | Terpene |
|  |  |  |  | CYP170A20 | Terpene |
|  |  |  |  | CYP107L32 | Nrps-Siderophore |
|  |  |  |  | CYP105B41 | T2pks |
| *Streptomyces noursei* | 64 | 37 | 24 | CYP105BV1 | T1pks |
|  |  |  |  | CYP1198A1 | T1pks |
|  |  |  |  | CYP107EM1 | T1pks |
|  |  |  |  | CYP1060A2 | T1pks |
|  |  |  |  | CYP147F29 | T1pks |
|  |  |  |  | CYP157C31 | Terpene |
|  |  |  |  | CYP105H1 | T1pks |
|  |  |  |  | CYP161A1 | T1pks |
|  |  |  |  | CYP107AE10 | Butyrolactone |
|  |  |  |  | CYP1248A3 | Other |
|  |  |  |  | CYP107EQ1 | T1pks |
|  |  |  |  | CYP107KZ1 | T1pks |
|  |  |  |  | CYP1420A1 | T1pks |
|  |  |  |  | CYP107A3 | T1pks |
|  |  |  |  | CYP107F9 | T3pks |
|  |  |  |  | CYP113D4 | Thiopeptide-Bacteriocin |
|  |  |  |  | CYP105AB15 | T1pks |
|  |  |  |  | CYP107CA2 | Transatpks-Nrps |
|  |  |  |  | CYP154D14 | Transatpks-Nrps |
|  |  |  |  | CYP105AC16 | Transatpks-Nrps |
|  |  |  |  | CYP105A7 | Terpene |
|  |  |  |  | CYP163B; | Lantipeptide-Nrps |
|  |  |  |  | CYP1278B1 | Lantipeptide-Nrps |
|  |  |  |  | CYP105H8 | Terpene-T1pks |
| *Streptomyces lydicus* A02 | 38 | 35 | 17 | CYP105BW1 | T2pks |
|  |  |  |  | CYP147K2 | Transatpks |
|  |  |  |  | CYP1038A7 | Transatpks |
|  |  |  |  | CYP147F28P | Terpene |
|  |  |  |  | CYP107AE8 | Butyrolactone |
|  |  |  |  | CYP1469A2 | Other |
|  |  |  |  | CYP157C29 | Terpene |
|  |  |  |  | CYP107F8; | T3pks-Nrps |
|  |  |  |  | CYP105D25 | T3pks-Nrps |
|  |  |  |  | CYP157C42 | Terpene |
|  |  |  |  | CYP113D5 | T2pks-Oligosaccharide |
|  |  |  |  | CYP1278B2; | Lassopeptide-Nrps |
|  |  |  |  | CYP163B11 | Lassopeptide-Nrps |
|  |  |  |  | CYP121A3 | Other |
|  |  |  |  | CYP161A5 | T1pks |
|  |  |  |  | CYP105H3 | T1pks |
|  |  |  |  | CYP186D1 | T1pks |
| *Streptomyces lydicus* 103 | 32 | 26 | 10 | CYP147F26 | T1pks |
|  |  |  |  | CYP107FH2 | Terpene-T1pks-Nrps |
|  |  |  |  | CYP107B26 | Terpene-T1pks-Nrps |
|  |  |  |  | CYP163G1 | T1pks-Nrps |
|  |  |  |  | CYP147F27 | Thiopeptide |
|  |  |  |  | CYP107AE12 | Butyrolactone |
|  |  |  |  | CYP107FV4 | Melanin-Nrps |
|  |  |  |  | CYP1005B7 | Other |
|  |  |  |  | CYP157C36 | Nucleoside-Lassopeptide-Nrps |
|  |  |  |  | CYP107F15 | T3pks-Nrps |
| *Streptomyces* sp. Tu6071 | 22 | 18 | 11 | CYP105B | Terpene |
|  |  |  |  | CYP105N | NRPS |
|  |  |  |  | CYP107BC1 | PKS-like,terpene |
|  |  |  |  | CYP161B1 | PKS-like,terpene |
|  |  |  |  | CYP113F1 | PKS-like,terpene |
|  |  |  |  | CYP107BB1 | PKS-like,terpene |
|  |  |  |  | CYP170B | Terpene |
|  |  |  |  | CYP107BX | NRPS,T1PKS |
|  |  |  |  | CYP1200A1 | Butyrolactone |
|  |  |  |  | CYP208A | T1PKS,NRPS |
|  |  |  |  | CYP154AJ1 | T1PKS,NRPS |
| *Streptomyces* sp. LaPpAH-201 | 19 | 26 | 5 | CYP105DT1 | T1PKS |
|  |  |  |  | CYP170B | Terpene |
|  |  |  |  | CYP107F | T3PKS |
|  |  |  |  | CYP105H | T1PKS,NRPS-like,NRPS,lanthipeptide |
|  |  |  |  | CYP146A | NRPS |
| *Streptomyces* sp. W007 | 28 | 44 | 3 | CYP107L | NRPS-like,arylpolyene,ladderane,NRPS |
|  |  |  |  | CYP163B | NRPS-like,arylpolyene,ladderane,NRPS |
|  |  |  |  | CYP107BZ | NRPS-like,arylpolyene,ladderane,NRPS |
| *Streptomyces* sp. TAA486-18 | 18 | 23 | 3 | CYP157C | Terpene |
|  |  |  |  | CYP251A | Terpene |
|  |  |  |  | CYP105AB | NRPS-like |
| *Streptomyces* sp. PVA 94-07 | 20 | 24 | 10 | CYP105AK | NRPS |
|  |  |  |  | CYP107R1 | T2PKS |
|  |  |  |  | CYP107F | T3PKS,T1PKS,NRPS-like,NRPS,lanthipeptide |
|  |  |  |  | CYP105H | T3PKS,T1PKS,NRPS-like,NRPS,lanthipeptide |
|  |  |  |  | CYP105BT | butyrolactone,T1PKS,other,NRPS |
|  |  |  |  | CYP105BT | butyrolactone,T1PKS,other,NRPS |
|  |  |  |  | CYP105AC | NRPS,T3PKS,T1PKS |
|  |  |  |  | CYP105AC | NRPS,T3PKS,T1PKS |
|  |  |  |  | CYP107BX | NRPS,T1PKS |
|  |  |  |  | CYP170B | Terpene |
| *Streptomyces canus* 299MFChir4.1 | 28 | 33 | 7 | CYP157C | Terpene |
|  |  |  |  | CYP170A | Terpene |
|  |  |  |  | CYP182B | T1PKS |
|  |  |  |  | CYP183B6 | Terpene |
|  |  |  |  | CYP251T1 | Terpene |
|  |  |  |  | CYP163H | T1PKS,NRPS,NRPS-like |
|  |  |  |  | CYP113AF1 | NRPS,lassopeptide |
| *Streptomyces sulphureus* DSM 40104 | 26 | 26 | 12 | CYP105DH3 | T2PKS |
|  |  |  |  | CYP113AC1 | NRPS,T1PKS |
|  |  |  |  | CYP107MD1 | T1PKS |
|  |  |  |  | CYP107CS | transAT-PKS,T1PKS,transAT-PKS-like,NRPS |
|  |  |  |  | CYP105DM1 | transAT-PKS,T1PKS,transAT-PKS-like,NRPS |
|  |  |  |  | CYP1265A1 | T1PKS |
|  |  |  |  | CYP135E8 | NRPS |
|  |  |  |  | CYP107FF2 | NRPS |
|  |  |  |  | CYP107AM | Nucleoside |
|  |  |  |  | CYP105B | Nucleoside |
|  |  |  |  | CYP107AM | Nucleoside |
|  |  |  |  | CYP283A | bacteriocin,bottromycin |
| *Streptomyces* sp. MspMP-M5 | 44 | 48 | 15 | CYP107F | T3PKS |
|  |  |  |  | CYP1237C2 | oligosaccharide,T2PKS,PKS-like,NRPS |
|  |  |  |  | CYP1237B3 | oligosaccharide,T2PKS,PKS-like,NRPS |
|  |  |  |  | CYP105DN1 | oligosaccharide,T2PKS,PKS-like,NRPS |
|  |  |  |  | CYP163B | transAT-PKS-like,NRPS,T1PKS |
|  |  |  |  | CYP121A | NRPS-like,CDPS |
|  |  |  |  | CYP156B | NRPS-like,CDPS |
|  |  |  |  | CYP1035A | NRPS-like,CDPS |
|  |  |  |  | CYP107EB | other,NRPS |
|  |  |  |  | CYP107AE | Butyrolactone |
|  |  |  |  | CYP105AC | T3PKS,NRPS-like |
|  |  |  |  | CYP1004B2 | NRPS,arylpolyene |
|  |  |  |  | CYP125G3 | NRPS,arylpolyene |
|  |  |  |  | CYP105BA | T1PKS,NRPS |
|  |  |  |  | CYP157C | Terpene |
| *Streptomyces* sp. LaPpAH-95 | 24 | 27 | 6 | CYP107BT5 | NRPS |
|  |  |  |  | CYP107CD | T2PKS,terpene |
|  |  |  |  | CYP105DW1 | T1PKS |
|  |  |  |  | CYP1046A | T1PKS,NRPS |
|  |  |  |  | CYP154M12 | NRPS,T1PKS |
|  |  |  |  | CYP208A | NRPS,T1PKS |
| *Streptomyces mirabilis* YR139 | 42 | 38 | 6 | CYP1859A2 | NRPS-like |
|  |  |  |  | CYP163H | NRPS-like |
|  |  |  |  | CYP170A | Terpene |
|  |  |  |  | CYP180A | Terpene |
|  |  |  |  | CYP247A | NRPS,other,T1PKS |
|  |  |  |  | CYP166A | NRPS-like |
| *Streptomyces* sp. AA1529 | 26 | 28 | 12 | CYP158B2 | T3PKS |
|  |  |  |  | CYP107MK1 | T1PKS,transAT-PKS-like |
|  |  |  |  | CYP1031A | transAT-PKS,NRPS,T1PKS |
|  |  |  |  | CYP1192A | NRPS-like,T1PKS |
|  |  |  |  | CYP107MH2 | NRPS-like,T1PKS |
|  |  |  |  | CYP157C | NRPS-like,T1PKS |
|  |  |  |  | CYP183A | Terpene |
|  |  |  |  | CYP161C | Terpene |
|  |  |  |  | CYP1237A4 | T2PKS,T1PKS |
|  |  |  |  | CYP107A | T2PKS,T1PKS |
|  |  |  |  | CYP107R2 | T2PKS |
|  |  |  |  | CYP163B | T1PKS,NRPS-like,NRPS |
| *Streptomyces atratus* OK008 | 15 | 15 | 1 | CYP107F | T3PKS |
| *Streptomyces* sp. PsTaAH-130 | 36 | 37 | 12 | CYP146A3 | NRPS |
|  |  |  |  | CYP105BC | other,T1PKS,PKS-like |
|  |  |  |  | CYP105AH | other,T1PKS,PKS-like |
|  |  |  |  | CYP180A | Terpene |
|  |  |  |  | CYP105D | T1PKS |
|  |  |  |  | CYP105P | NRPS,T1PKS |
|  |  |  |  | CYP105D | NRPS,T1PKS |
|  |  |  |  | CYP1469D1 | CDPS |
|  |  |  |  | CYP1253B3 | NRPS |
|  |  |  |  | CYP163B | transAT-PKS-like,NRPS,T1PKS |
|  |  |  |  | CYP105B | Terpene |
|  |  |  |  | CYP1207A | NRPS,lanthipeptide |
| *Streptomyces* sp. CNT318 | 27 | 32 | 13 | CYP107MK1 | transAT-PKS,NRPS,transAT-PKS-like,T1PKS |
|  |  |  |  | CYP158B2 | T3PKS |
|  |  |  |  | CYP183A | Terpene |
|  |  |  |  | CYP161C | Terpene |
|  |  |  |  | CYP107AL | T2PKS,T1PKS |
|  |  |  |  | CYP105N | NRPS |
|  |  |  |  | CYP107E | T2PKS |
|  |  |  |  | CYP157K | T2PKS |
|  |  |  |  | CYP1192A | NRPS-like,T1PKS |
|  |  |  |  | CYP1192A | NRPS-like,T1PKS |
|  |  |  |  | CYP107MH2 | NRPS-like,T1PKS |
|  |  |  |  | CYP157C | NRPS-like,T1PKS |
|  |  |  |  | CYP1031A | transAT-PKS,NRPS,T1PKS |
| *Streptomyces* sp. CNH099 | 16 | 39 | 10 | CYP157C | Terpene |
|  |  |  |  | CYP105AC | T1PKS |
|  |  |  |  | CYP285A | PKS-like,NRPS |
|  |  |  |  | CYP146C1 | PKS-like,NRPS |
|  |  |  |  | CYP126B5 | T1PKS |
|  |  |  |  | CYP107AT5 | T1PKS |
|  |  |  |  | CYP107F | T3PKS |
|  |  |  |  | CYP105BA | NRPS,T1PKS |
|  |  |  |  | CYP1064A | other,terpene |
|  |  |  |  | CYP1265B1 | T1PKS |
| *Streptomyces* sp. CNH287 | 16 | 27 | 8 | CYP105AR2 | Terpene |
|  |  |  |  | CYP251D2 | Terpene |
|  |  |  |  | CYP251B2 | Terpene |
|  |  |  |  | CYP1044A3 | Terpene |
|  |  |  |  | CYP107BY | NRPS |
|  |  |  |  | CYP102B | hglE-KS,LAP,terpene |
|  |  |  |  | CYP107F | T3PKS |
|  |  |  |  | CYP163C | NRPS,PKS-like |
| *Streptomyces* sp. GXT6 | 13 | 24 | 3 | CYP170A | Terpene |
|  |  |  |  | CYP158A | T3PKS |
|  |  |  |  | CYP154A | Bacteriocin |
| *Streptomyces* sp. LaPpAH-108 | 24 | 21 | 3 | CYP170A | Terpene |
|  |  |  |  | CYP154U | NRPS |
|  |  |  |  | CYP158A | NRPS,T3PKS |
| *Streptomyces aurantiacus* JA 4570 | 30 | 49 | 12 | CYP105BT | other,NRPS,T1PKS |
|  |  |  |  | CYP247A | other,NRPS,T1PKS |
|  |  |  |  | CYP157K | Terpene |
|  |  |  |  | CYP183J | Terpene |
|  |  |  |  | CYP105DH2 | T2PKS |
|  |  |  |  | CYP113K | NRPS |
|  |  |  |  | CYP1658A1 | LAP |
|  |  |  |  | CYP163B | NRPS |
|  |  |  |  | CYP1031A | T3PKS,PKS-like,NRPS |
|  |  |  |  | CYP107ND1 | T1PKS |
|  |  |  |  | CYP170B | Terpene |
|  |  |  |  | CYP294A3 | T1PKS |
| *Streptomyces* sp. KhCrAH-337 | 26 | 26 | 3 | CYP105D | T1PKS |
|  |  |  |  | CYP105D | T1PKS |
|  |  |  |  | CYP163B | NRPS |
| *Streptomyces* sp. LaPpAH-202 | 19 | 32 | 6 | CYP107BX | T1PKS,NRPS |
|  |  |  |  | CYP170B | Terpene |
|  |  |  |  | CYP107F | T3PKS |
|  |  |  |  | CYP105DT1 | T1PKS |
|  |  |  |  | CYP146A | NRPS |
|  |  |  |  | CYP105H | T1PKS,NRPS-like,lanthipeptide,NRPS |
| *Streptomyces* sp. UNC401CLCol | 15 | 14 | 3 | CYP170A | Terpene |
|  |  |  |  | CYP1240A5 | Terpene |
|  |  |  |  | CYP1216A1 | Terpene |
| *Streptomyces* sp. KhCrAH-40 | 26 | 26 | 6 | CYP105D | T1PKS |
|  |  |  |  | CYP105D | T1PKS |
|  |  |  |  | CYP163B | NRPS |
|  |  |  |  | CYP1004F2 | NRPS |
|  |  |  |  | CYP1004E2 | NRPS |
|  |  |  |  | CYP1618A | NRPS |
| *Kitasatospora* sp. SolWspMP-SS2h | 25 | 33 | 9 | CYP105DX1 | Terpene |
|  |  |  |  | CYP251M2 | Terpene |
|  |  |  |  | CYP2027A | T1PKS |
|  |  |  |  | CYP105B | NRPS |
|  |  |  |  | CYP154A | ectoine,PKS-like,T1PKS |
|  |  |  |  | CYP107MG1 | T1PKS |
|  |  |  |  | CYP124G15 | PKS-like,butyrolactone |
|  |  |  |  | CYP1448A3 | LAP,NRPS,T1PKS,butyrolactone |
|  |  |  |  | CYP1265C1 | T2PKS |
| *Streptomyces* sp. NTK 937 | 17 | 24 | 1 | CYP105BA | T1PKS,NRPS |
| *Streptomyces* sp. HmicA12 | 25 | 18 | 7 | CYP285A | NRPS,T1PKS |
|  |  |  |  | CYP123H2 | NRPS,T1PKS |
|  |  |  |  | CYP154D | T3PKS |
|  |  |  |  | CYP158A | T3PKS |
|  |  |  |  | CYP170B | Terpene |
|  |  |  |  | CYP180A | Terpene |
|  |  |  |  | CYP1341F1 | fused |
| *Streptomyces griseoaurantiacus* M045 | 16 | 25 | 4 | CYP105N | NRPS |
|  |  |  |  | CYP170A | Terpene |
|  |  |  |  | CYP2080A1 | T1PKS |
|  |  |  |  | CYP105AC | fused,T3PKS |
| *Streptomyces afghaniensis* 772 | 28 | 47 | 7 | CYP107AH | T2PKS |
|  |  |  |  | CYP1424A | NRPS |
|  |  |  |  | CYP105EB1 | T2PKS |
|  |  |  |  | CYP170A | Terpene |
|  |  |  |  | CYP107CT | NRPS,T1PKS |
|  |  |  |  | CYP154A | Terpene |
|  |  |  |  | CYP154K | Butyrolactone |
| *Streptomyces sulphureus* L180 | 19 | 30 | 3 | CYP151D1 | Ectoine |
|  |  |  |  | CYP105DM1 | T1PKS |
|  |  |  |  | CYP1037B | NRPS,T1PKS,butyrolactone |
| *Streptomyces* sp. KhCrAH-340 | 26 | 26 | 3 | CYP105D | T1PKS |
|  |  |  |  | CYP105D | T1PKS |
|  |  |  |  | CYP163B | NRPS |
| *Streptomyces violaceusniger* SPC6 | 13 | 17 | 7 | CYP163B | NRPS,T1PKS,ladderane,NRPS-like |
|  |  |  |  | CYP158A | Terpene |
|  |  |  |  | CYP157C | Terpene |
|  |  |  |  | CYP251A | Terpene |
|  |  |  |  | CYP251F | T3PKS,terpene |
|  |  |  |  | CYP1207A | lanthipeptide,NRPS,T3PKS |
|  |  |  |  | CYP158A | lanthipeptide,NRPS,T3PKS |
| *Streptomyces* sp. CNS615 | 27 | 37 | 9 | CYP163J1 | NRPS |
|  |  |  |  | CYP107BM | Terpene |
|  |  |  |  | CYP158A | T3PKS |
|  |  |  |  | CYP158A | T3PKS |
|  |  |  |  | CYP105H | T1PKS |
|  |  |  |  | CYP208A | butyrolactone,T1PKS,NRPS,other |
|  |  |  |  | CYP154M | butyrolactone,T1PKS,NRPS,other |
|  |  |  |  | CYP211A | butyrolactone,T1PKS,NRPS,other |
|  |  |  |  | CYP1562A2 | butyrolactone,T1PKS,NRPS,other |
| *Streptomyces vitaminophilus* DSM 41686 | 18 | 25 | 2 | CYP105BA | T3PKS,other,NRPS-like,NRPS,T1PKS |
|  |  |  |  | CYP107KW | NRPS-like,other |
| *Streptomyces bottropensis* ATCC 25435 | 31 | 28 | 8 | CYP107AM | T1PKS,NRPS,T2PKS |
|  |  |  |  | CYP154A | Terpene |
|  |  |  |  | CYP157C | Terpene |
|  |  |  |  | CYP156B | Indole |
|  |  |  |  | CYP113AD1 | T1PKS |
|  |  |  |  | CYP105DS2 | T1PKS |
|  |  |  |  | CYP107AL | PKS-like,LAP,butyrolactone,T1PKS |
|  |  |  |  | CYP283A | bacteriocin,bottromycin |
| *Streptomyces* sp. CNQ865 | 16 | 46 | 13 | CYP126B6 | T1PKS |
|  |  |  |  | CYP107AT4 | T1PKS |
|  |  |  |  | CYP251A | Terpene |
|  |  |  |  | CYP157C | Terpene |
|  |  |  |  | CYP1459B5 | NRPS |
|  |  |  |  | CYP1279A1 | NRPS |
|  |  |  |  | CYP105AC | T1PKS |
|  |  |  |  | CYP165E | other,T3PKS,NRPS |
|  |  |  |  | CYP165B | other,T3PKS,NRPS |
|  |  |  |  | CYP154C | T3PKS |
|  |  |  |  | CYP157A | T3PKS |
|  |  |  |  | CYP1959B1 | T1PKS |
|  |  |  |  | CYP1064A | Terpene |
| *Streptomyces* sp. CNT360 | 19 | 19 | 6 | CYP2045B1 | transAT-PKS,NRPS |
|  |  |  |  | CYP155A | NRPS,T1PKS |
|  |  |  |  | CYP1039C1 | NRPS,T1PKS |
|  |  |  |  | CYP113AE1 | T1PKS |
|  |  |  |  | CYP186B8 | NRPS,lanthipeptide,T1PKS |
|  |  |  |  | CYP107E | NRPS |
| *Streptomyces* sp. 142MFCol3.1 | 27 | 30 | 7 | CYP157K | Terpene |
|  |  |  |  | CYP1417A | Terpene |
|  |  |  |  | CYP158A | T3PKS |
|  |  |  |  | CYP180A | Terpene |
|  |  |  |  | CYP154A | Terpene |
|  |  |  |  | CYP156C | Terpene |
|  |  |  |  | CYP170A | Terpene |
| *Streptomyces* sp. ScaeMP-e122 | 25 | 24 | 8 | CYP105N | NRPS |
|  |  |  |  | CYP124G | Melanin |
|  |  |  |  | CYP107BX | T1PKS,NRPS |
|  |  |  |  | CYP105A | NRPS,butyrolactone |
|  |  |  |  | CYP105AZ | NRPS,T1PKS |
|  |  |  |  | CYP105AZ | NRPS,T1PKS |
|  |  |  |  | CYP181A | T2PKS,PKS-like,T1PKS |
|  |  |  |  | CYP107Y | T2PKS,PKS-like,T1PKS |
| *Streptomyces* sp. TAA204 | 18 | 34 | 5 | CYP154A | NRPS-like |
|  |  |  |  | CYP1832A1 | NRPS-like |
|  |  |  |  | CYP113C | terpene,NRPS,PKS-like,transAT-PKS,furan |
|  |  |  |  | CYP251A | Terpene |
|  |  |  |  | CYP157C | Terpene |
| *Streptomyces* sp. CNQ329 | 13 | 36 | 9 | CYP157C | T1PKS |
|  |  |  |  | CYP1043A | T3PKS |
|  |  |  |  | CYP108B | thiopeptide,other,furan,LAP |
|  |  |  |  | CYP165E | NRPS |
|  |  |  |  | CYP165B | NRPS |
|  |  |  |  | CYP154C | T3PKS |
|  |  |  |  | CYP157A | T3PKS |
|  |  |  |  | CYP107AT6 | T1PKS |
|  |  |  |  | CYP126B5 | T1PKS |
| *Streptomyces* sp. KhCrAH-244 | 26 | 28 | 2 | CYP105D | T1PKS |
|  |  |  |  | CYP105D | T1PKS |
| *Streptomyces chartreusis* NRRL 12338 | 23 | 33 | 6 | CYP154K | Butyrolactone |
|  |  |  |  | CYP154A | Terpene |
|  |  |  |  | CYP105D | Terpene |
|  |  |  |  | CYP157K | Terpene |
|  |  |  |  | CYP170A | Terpene |
|  |  |  |  | CYP107AH | T2PKS |
| *Streptomyces* sp. CcalMP-8W | 23 | 38 | 9 | CYP107BX | NRPS,T1PKS,bacteriocin |
|  |  |  |  | CYP1984A1 | NRPS,T2PKS,PKS-like,other,oligosaccharide |
|  |  |  |  | CYP124G | Melanin |
|  |  |  |  | CYP154C | lanthipeptide |
|  |  |  |  | CYP157A | lanthipeptide |
|  |  |  |  | CYP105D | NRPS-like,arylpolyene |
|  |  |  |  | CYP107F | T3PKS |
|  |  |  |  | CYP154D | NRPS |
|  |  |  |  | CYP157F | NRPS |
| *Streptomyces* sp. SS | 15 | 23 | 3 | CYP102G | NRPS |
|  |  |  |  | CYP105D | Terpene |
|  |  |  |  | CYP105AC | NRPS |
| *Streptomyces* sp. CNQ766 | 16 | 50 | 12 | CYP157C | Terpene |
|  |  |  |  | CYP251A | Terpene |
|  |  |  |  | CYP1459B5 | NRPS |
|  |  |  |  | CYP1279A1 | NRPS |
|  |  |  |  | CYP105AC | T1PKS,transAT-PKS |
|  |  |  |  | CYP126B6 | T1PKS |
|  |  |  |  | CYP1064A | Terpene |
|  |  |  |  | CYP154C | T3PKS |
|  |  |  |  | CYP157A | T3PKS |
|  |  |  |  | CYP1959B1 | T1PKS |
|  |  |  |  | CYP165E | NRPS |
|  |  |  |  | CYP165B | NRPS |
| *Streptomyces* sp. URHA0041 | 16 | 39 | 7 | CYP123D | PKS-like |
|  |  |  |  | CYP183AR2 | terpene,phenazine |
|  |  |  |  | CYP1044A4 | terpene,phenazine |
|  |  |  |  | CYP154A | PKS-like,T1PKS |
|  |  |  |  | CYP107JK3 | T2PKS |
|  |  |  |  | CYP156B | Indole |
|  |  |  |  | CYP107MG2 | T1PKS |
| *Streptomyces* sp. CNB091 | 27 | 41 | 5 | CYP107BY | NRPS |
|  |  |  |  | CYP107BX | NRPS,T1PKS |
|  |  |  |  | CYP124G | Melanin |
|  |  |  |  | CYP107F | T3PKS |
|  |  |  |  | CYP2134B1 | NRPS,T1PKS |
| *Streptomyces flavidovirens* DSM 40150 | 24 | 24 | 9 | CYP105B | T2PKS,LAP |
|  |  |  |  | CYP121A | lanthipeptide,CDPS |
|  |  |  |  | CYP157K | bacteriocin,terpene |
|  |  |  |  | CYP124G | Melanin |
|  |  |  |  | CYP245B1 | Indole |
|  |  |  |  | CYP1634A2 | NRPS,T1PKS |
|  |  |  |  | CYP1228A1 | NRPS,T1PKS |
|  |  |  |  | CYP107F | T3PKS |
|  |  |  |  | CYP157C | T1PKS,NRPS,terpene |
| *Streptomyces viridosporus* T7A, ATCC 39115 | 32 | 32 | 9 | CYP105D | T3PKS |
|  |  |  |  | CYP163B | NRPS |
|  |  |  |  | CYP170A | Terpene |
|  |  |  |  | CYP1207A | NRPS,lanthipeptide,T2PKS |
|  |  |  |  | CYP105AC | fused,T3PKS |
|  |  |  |  | CYP154B | NRPS,T1PKS,other |
|  |  |  |  | CYP208A | butyrolactone,T1PKS,NRPS,other |
|  |  |  |  | CYP154M | butyrolactone,T1PKS,NRPS,other |
|  |  |  |  | CYP211F | butyrolactone,T1PKS,NRPS,other |
| *Streptomyces* sp. FXJ7.023 | 27 | 14 | 3 | CYP170A | terpene,betalactone |
|  |  |  |  | CYP158A | T3PKS |
|  |  |  |  | CYP105CD | NRPS |
| *Streptomyces* sp. ATexAB-D23 | 28 | 42 | 11 | CYP107BT3 | T2PKS,terpene,NRPS |
|  |  |  |  | CYP1394B1 | NRPS,betalactone |
|  |  |  |  | CYP107LX1 | NRPS,betalactone,T3PKS |
|  |  |  |  | CYP2027A | T1PKS |
|  |  |  |  | CYP105DW1 | T1PKS |
|  |  |  |  | CYP157F | T1PKS |
|  |  |  |  | CYP154D | T1PKS |
|  |  |  |  | CYP156B | Indole |
|  |  |  |  | CYP107JK2 | T2PKS,butyrolactone |
|  |  |  |  | CYP105CF3 | oligosaccharide,T1PKS |
|  |  |  |  | CYP161E7 | PKS-like |
| *Streptomyces* sp. BoleA5 | 17 | 23 | 7 | CYP107X | T2PKS,bacteriocin |
|  |  |  |  | CYP107BM | T3PKS |
|  |  |  |  | CYP107F | T3PKS |
|  |  |  |  | CYP154C | Terpene |
|  |  |  |  | CYP157C | Terpene |
|  |  |  |  | CYP251A | Terpene |
|  |  |  |  | CYP183B | Terpene |
| *Streptomyces* sp. CNS654 | 27 | 48 | 3 | CYP107B | bacteriocin,transAT-PKS,PKS-like,T1PKS,NRPS |
|  |  |  |  | CYP107BX | bacteriocin,transAT-PKS,PKS-like,T1PKS,NRPS |
|  |  |  |  | CYP163B | NRPS |
| *Streptomyces* sp. DpondAA-B6 | 19 | 28 | 4 | CYP107BX | T1PKS,NRPS |
|  |  |  |  | CYP163B | PKS-like,NRPS |
|  |  |  |  | CYP124G | Melanin |
|  |  |  |  | CYP157K | Terpene |
| *Streptomyces* sp. PCS3-D2 | 25 | 27 | 7 | CYP163J3 | NRPS |
|  |  |  |  | CYP105EA1 | NRPS-like,NRPS |
|  |  |  |  | CYP157C | terpene,thiopeptide,LAP,NRPS |
|  |  |  |  | CYP107L | Siderophore |
|  |  |  |  | CYP121A | CDPS,NRPS-like |
|  |  |  |  | CYP162B | NRPS |
|  |  |  |  | CYP147F | T2PKS,NRPS |
| *Streptomyces* sp. CNR698 | 29 | 39 | 11 | CYP1424A | NRPS |
|  |  |  |  | CYP180A | NRPS |
|  |  |  |  | CYP107BM | Terpene |
|  |  |  |  | CYP158A | T3PKS |
|  |  |  |  | CYP158A | T3PKS |
|  |  |  |  | CYP163J2 | NRPS |
|  |  |  |  | CYP146A | NRPS |
|  |  |  |  | CYP211A | Other |
|  |  |  |  | CYP1562A2 | Other |
|  |  |  |  | CYP208A | T1PKS,butyrolactone |
|  |  |  |  | CYP154M | NRPS |
| *Streptomyces cattleya* ATCC 35852 | 41 | 46 | 14 | CYP107AE | lanthipeptide |
|  |  |  |  | CYP107CT | butyrolactone,NRPS,T1PKS |
|  |  |  |  | CYP105B | butyrolactone,NRPS,T1PKS |
|  |  |  |  | CYP163D | NRPS,thiopeptide |
|  |  |  |  | CYP183L | Terpene |
|  |  |  |  | CYP105BF | T1PKS |
|  |  |  |  | CYP285A | terpene,NRPS |
|  |  |  |  | CYP1061A | terpene,NRPS |
|  |  |  |  | CYP158A | T3PKS,terpene |
|  |  |  |  | CYP107CR | T1PKS |
|  |  |  |  | CYP1274A | T1PKS |
|  |  |  |  | CYP107W | T1PKS |
|  |  |  |  | CYP107CS | T1PKS |
|  |  |  |  | CYP184A | NRPS |
| *Streptomyces* sp. WMMB 714 | 21 | 25 | 2 | CYP105BA | NRPS,T1PKS |
|  |  |  |  | CYP105BA | NRPS,T1PKS |
| *Streptomyces scabrisporus* DSM 41855 | 37 | 47 | 11 | CYP1199A | NRPS,indole |
|  |  |  |  | CYP1385A3 | thiopeptide,LAP |
|  |  |  |  | CYP151B2 | T1PKS |
|  |  |  |  | CYP1045B1 | Terpene, T1PKS |
|  |  |  |  | CYP105DZ1 | other,T1PKS |
|  |  |  |  | CYP107MZ1 | other,T1PKS |
|  |  |  |  | CYP107F | T3PKS |
|  |  |  |  | CYP1568A1 | NRPS,T1PKS,terpene |
|  |  |  |  | CYP285G1 | NRPS |
|  |  |  |  | CYP107NA1 | NRPS |
|  |  |  |  | CYP285H1 | NRPS |
| *Streptomyces* sp. KhCrAH-43 | 26 | 26 | 2 | CYP105D | T1PKS |
|  |  |  |  | CYP105D | T1PKS |
| *Streptomyces* sp. PsTaAH-124 | 32 | 42 | 12 | CYP107P | NRPS-like |
|  |  |  |  | CYP1722A | NRPS,bacteriocin |
|  |  |  |  | CYP107E | Phosphoglycolipid |
|  |  |  |  | CYP183X | terpene,other |
|  |  |  |  | CYP158A | T3PKS |
|  |  |  |  | CYP170A | Terpene |
|  |  |  |  | CYP105N | NRPS |
|  |  |  |  | CYP1618A | NRPS,phosphonate |
|  |  |  |  | CYP180B | LAP,thiopeptide,terpene |
|  |  |  |  | CYP105B | Butyrolactone |
|  |  |  |  | CYP1042A | NRPS |
|  |  |  |  | CYP105DU1 | NRPS,T1PKS,thiopeptide |
| *Streptomyces* sp. Amel2xC10 | 15 | 28 | 4 | CYP107Z | NRPS,other |
|  |  |  |  | CYP156B | Indole |
|  |  |  |  | CYP105EH1 | NRPS,T1PKS |
|  |  |  |  | CYP170A | Terpene |
| *Streptomyces* sp. CNT372 | 10 | 29 | 1 | CYP124G | Melanin |
| *Streptomyces* sp. CNS606 | 16 | 31 | 5 | CYP156Q1 | Terpene |
|  |  |  |  | CYP157C | Terpene |
|  |  |  |  | CYP251A | Terpene |
|  |  |  |  | CYP105AC46 | T1PKS,NRPS,butyrolactone |
|  |  |  |  | CYP1917A2 | T1PKS,NRPS,butyrolactone |
| *Streptomyces* sp. 303MFCol5.2 | 23 | 28 | 6 | CYP154C | T3PKS |
|  |  |  |  | CYP157A | T3PKS |
|  |  |  |  | CYP170A | Terpene |
|  |  |  |  | CYP105CD4 | T2PKS |
|  |  |  |  | CYP181A | T2PKS,PKS-like,T1PKS |
|  |  |  |  | CYP107Y | T2PKS,PKS-like,T1PKS |
| *Streptomyces* *acidiscabies* 84-104 | 47 | 45 | 12 | CYP246A | NRPS |
|  |  |  |  | CYP105D | T1PKS |
|  |  |  |  | CYP170A | Terpene |
|  |  |  |  | CYP107MR1 | betalactone,NRPS |
|  |  |  |  | CYP107MB1 | NRPS,T2PKS,T1PKS,NRPS-like |
|  |  |  |  | CYP158A | T3PKS |
|  |  |  |  | CYP124B | T1PKS |
|  |  |  |  | CYP1524A2 | NRPS |
|  |  |  |  | CYP107BT6 | NRPS |
|  |  |  |  | CYP156B | Indole |
|  |  |  |  | CYP105K3 | nucleoside,betalactone,NRPS-like |
|  |  |  |  | CYP162A | nucleoside,betalactone,NRPS-like |
| *Streptomyces* sp. S4 | 19 | 33 | 4 | CYP105H | lanthipeptide,NRPS,T1PKS,NRPS-like |
|  |  |  |  | CYP107F | T3PKS |
|  |  |  |  | CYP107BX | NRPS,T1PKS |
|  |  |  |  | CYP170B | Terpene |
| *Streptomyces* sp. DvalAA-21 | 24 | 27 | 7 | CYP124G | Melanin |
|  |  |  |  | CYP107BX | T1PKS,NRPS |
|  |  |  |  | CYP105A | NRPS,butyrolactone |
|  |  |  |  | CYP107Y | T2PKS,PKS-like,T1PKS |
|  |  |  |  | CYP181A | T2PKS,PKS-like,T1PKS |
|  |  |  |  | CYP105AZ | T1PKS |
|  |  |  |  | CYP105AZ | T1PKS |
| *Streptomyces* sp. CNT371 | 17 | 43 | 12 | CYP126B6 | T1PKS |
|  |  |  |  | CYP107AT4 | T1PKS |
|  |  |  |  | CYP157C | Terpene |
|  |  |  |  | CYP251A | Terpene |
|  |  |  |  | CYP1459B5 | NRPS |
|  |  |  |  | CYP157A | T3PKS |
|  |  |  |  | CYP154C | T3PKS |
|  |  |  |  | CYP1279A1 | NRPS |
|  |  |  |  | CYP105AC | T1PKS |
|  |  |  |  | CYP1064A | Terpene |
|  |  |  |  | CYP165B | NRPS |
|  |  |  |  | CYP165E | NRPS |
| *Streptomyces* *somaliensis* DSM 40738 | 10 | 26 | 3 | CYP158A | terpene,T3PKS |
|  |  |  |  | CYP157C | Terpene |
|  |  |  |  | CYP251A | Terpene |
| *Streptomyces* sp. 351MFTsu5.1 | 22 | 26 | 4 | CYP170A | Terpene |
|  |  |  |  | CYP182B | T1PKS |
|  |  |  |  | CYP107MC1 | T1PKS |
|  |  |  |  | CYP268H2 | T1PKS |
| *Streptomyces* sp. DvalAA-83 | 24 | 25 | 7 | CYP124G | Melanin |
|  |  |  |  | CYP105AZ | T1PKS |
|  |  |  |  | CYP105AZ | T1PKS |
|  |  |  |  | CYP107BX | T1PKS,NRPS |
|  |  |  |  | CYP105A | NRPS,butyrolactone |
|  |  |  |  | CYP181A | T2PKS,PKS-like |
|  |  |  |  | CYP107Y | T2PKS,PKS-like |
| *Streptomyces* sp. CNT302 | 26 | 28 | 7 | CYP105AH | other,T1PKS,butyrolactone |
|  |  |  |  | CYP158A | T3PKS |
|  |  |  |  | CYP158A | T3PKS |
|  |  |  |  | CYP107BM | Terpene |
|  |  |  |  | CYP163J2 | NRPS |
|  |  |  |  | CYP208A | T1PKS,butyrolactone |
|  |  |  |  | CYP154M | NRPS |
| *Streptomyces* sp. CNY243 | 17 | 41 | 13 | CYP1959B1 | T1PKS |
|  |  |  |  | CYP157C | Terpene |
|  |  |  |  | CYP251A | Terpene |
|  |  |  |  | CYP107AT4 | T1PKS |
|  |  |  |  | CYP126B6 | T1PKS |
|  |  |  |  | CYP1459B5 | NRPS |
|  |  |  |  | CYP105AC | T1PKS |
|  |  |  |  | CYP1279A1 | NRPS |
|  |  |  |  | CYP165B | NRPS,T3PKS,other |
|  |  |  |  | CYP165E | NRPS,T3PKS,other |
|  |  |  |  | CYP154C | T3PKS |
|  |  |  |  | CYP157A | T3PKS |
|  |  |  |  | CYP1064A | Terpene |
| *Streptomyces* sp. AA0539 | 19 | 21 | 10 | CYP186Q1 | oligosaccharide,T2PKS,NRPS |
|  |  |  |  | CYP107F | T3PKS |
|  |  |  |  | CYP157C | Terpene |
|  |  |  |  | CYP105N | NRPS |
|  |  |  |  | CYP183W | Terpene |
|  |  |  |  | CYP107AM | NRPS |
|  |  |  |  | CYP105CD | NRPS |
|  |  |  |  | CYP105EE1 | NRPS,T1PKS |
|  |  |  |  | CYP107LF | NRPS,T1PKS |
|  |  |  |  | CYP107EA | NRPS,T1PKS |
| *Streptomyces* *atratus* OK807 | 31 | 36 | 8 | CYP107R1 | T2PKS |
|  |  |  |  | CYP1240A4 | terpene,T1PKS,NRPS-like |
|  |  |  |  | CYP1216A11 | terpene,T1PKS,NRPS-like |
|  |  |  |  | CYP107LZ1 | terpene,T1PKS,NRPS-like |
|  |  |  |  | CYP107L | NRPS |
|  |  |  |  | CYP107L | NRPS |
|  |  |  |  | CYP107BX | T1PKS,NRPS |
|  |  |  |  | CYP107F | T3PKS |
| *Streptomyces* sp. CNS335 | 16 | 46 | 13 | CYP107AT4 | T1PKS |
|  |  |  |  | CYP126B6 | T1PKS |
|  |  |  |  | CYP1459B5 | NRPS |
|  |  |  |  | CYP251A | Terpene |
|  |  |  |  | CYP157C | Terpene |
|  |  |  |  | CYP105AC | T1PKS |
|  |  |  |  | CYP157A | T3PKS |
|  |  |  |  | CYP154C | T3PKS |
|  |  |  |  | CYP1279A1 | NRPS |
|  |  |  |  | CYP1064A | Terpene |
|  |  |  |  | CYP1959B1 | T1PKS |
|  |  |  |  | CYP165E | NRPS |
|  |  |  |  | CYP165B | NRPS |
| *Streptomyces* sp. FxanaC1 | 27 | 27 | 4 | CYP113AB1 | LAP,thiopeptide,lassopeptide,NRPS |
|  |  |  |  | CYP105DP1 | T1PKS,NRPS,melanin,T2PKS |
|  |  |  |  | CYP105AA | Terpene |
|  |  |  |  | CYP251G | terpene,amglyccycl |
| *Streptomyces* sp. TOR3209 | 20 | 55 | 7 | CYP152D | Terpene |
|  |  |  |  | CYP156B | Indole |
|  |  |  |  | CYP1417A | hglE-KS,T1PKS |
|  |  |  |  | CYP170A | Terpene |
|  |  |  |  | CYP158A | T3PKS |
|  |  |  |  | CYP157K | Terpene |
|  |  |  |  | CYP105H | T1PKS,NRPS-like |
| *Streptomyces* sp. DpondAA-E10 | 25 | 25 | 7 | CYP105AZ | T1PKS |
|  |  |  |  | CYP105AZ | T1PKS |
|  |  |  |  | CYP124G | Melanin |
|  |  |  |  | CYP105A | NRPS,butyrolactone |
|  |  |  |  | CYP107BX | T1PKS,NRPS |
|  |  |  |  | CYP107Y | T2PKS,PKS-like,T1PKS |
|  |  |  |  | CYP181A | T2PKS,PKS-like,T1PKS |
| *Streptomyces* sp. DpondAA-A50 | 25 | 27 | 7 | CYP124G | Melanin |
|  |  |  |  | CYP107BX | T1PKS,NRPS |
|  |  |  |  | CYP105A | NRPS,butyrolactone |
|  |  |  |  | CYP107Y | T2PKS,PKS-like,T1PKS |
|  |  |  |  | CYP181A | T2PKS,PKS-like,T1PKS |
|  |  |  |  | CYP105AZ | T1PKS |
|  |  |  |  | CYP105AZ | T1PKS |
| *Streptomyces* sp. TAA040 | 15 | 22 | 6 | CYP1207A | terpene,lanthipeptide,NRPS |
|  |  |  |  | CYP184A | NRPS,T3PKS,terpene |
|  |  |  |  | CYP105AH | other,T1PKS,LAP,thiopeptide |
|  |  |  |  | CYP161C | Terpene |
|  |  |  |  | CYP183A | Terpene |
|  |  |  |  | CYP156B | Terpene |
| *Streptomyces* sp. PgraA7 | 23 | 36 | 6 | CYP124G | Melanin |
|  |  |  |  | CYP107BX | bacteriocin,NRPS,T1PKS |
|  |  |  |  | CYP107F | T3PKS |
|  |  |  |  | CYP107MH1 | T1PKS,terpene |
|  |  |  |  | CYP183BC1 | T1PKS,terpene |
|  |  |  |  | CYP159F4 | T1PKS,terpene |
| *Streptomyces* sp. FxanaD5 | 15 | 14 | 3 | CYP170A | Terpene |
|  |  |  |  | CYP1240A5 | Terpene |
|  |  |  |  | CYP1216A1 | Terpene |
| *Streptomyces* *viridochromogenes* Tue57 | 31 | 38 | 8 | CYP107AH | T2PKS |
|  |  |  |  | CYP170A | Terpene |
|  |  |  |  | CYP121A | CDPS |
|  |  |  |  | CYP1059A | Bacteriocin |
|  |  |  |  | CYP105B | Bacteriocin |
|  |  |  |  | CYP154U | NRPS |
|  |  |  |  | CYP156H | NRPS |
|  |  |  |  | CYP183L | Terpene |
| *Streptomyces* sp. GBA 94-10 | 20 | 26 | 10 | CYP105AK | NRPS |
|  |  |  |  | CYP107R1 | T2PKS |
|  |  |  |  | CYP107F | T2PKS |
|  |  |  |  | CYP105H | T3PKS,T1PKS,NRPS-like,NRPS,lanthipeptide |
|  |  |  |  | CYP105BT | butyrolactone,T1PKS,other,NRPS |
|  |  |  |  | CYP105BT | NRPS,other,T1PKS,butyrolactone |
|  |  |  |  | CYP105AC | NRPS,T3PKS,T1PKS |
|  |  |  |  | CYP105AC | NRPS,T3PKS,T1PKS |
|  |  |  |  | CYP107BX | T1PKS,NRPS |
|  |  |  |  | CYP170B | Terpene |
| *Streptomyces* sp. CNQ-525 | 18 | 43 | 15 | CYP251A | Terpene |
|  |  |  |  | CYP157C | Terpene |
|  |  |  |  | CYP105AC | transAT-PKS,T1PKS |
|  |  |  |  | CYP1959B1 | T1PKS |
|  |  |  |  | CYP1064A | Terpene |
|  |  |  |  | CYP157A | T3PKS |
|  |  |  |  | CYP154C | T3PKS |
|  |  |  |  | CYP1529A1 | Terpene |
|  |  |  |  | CYP1279A1 | NRPS |
|  |  |  |  | CYP165B | NRPS,T3PKS,other |
|  |  |  |  | CYP165E | NRPS,T3PKS,other |
|  |  |  |  | CYP126B6 | T1PKS |
|  |  |  |  | CYP107AT4 | T1PKS |
|  |  |  |  | CYP108B | LAP |
|  |  |  |  | CYP107MN1 | T1PKS |
| *Streptomyces* *mirabilis* OK461 | 37 | 31 | 2 | CYP158A | T3PKS |
|  |  |  |  | CYP170A | Terpene |
| *Streptomyces* *exfoliatus* DSMZ 41693 | 26 | 33 | 6 | CYP1037B | NRPS-like,T1PKS,NRPS |
|  |  |  |  | CYP105AC | NRPS |
|  |  |  |  | CYP183AS1 | T2PKS,terpene,ectoine |
|  |  |  |  | CYP183AR1 | T2PKS,terpene,ectoine |
|  |  |  |  | CYP158C1 | T3PKS |
|  |  |  |  | CYP158A | T3PKS |
| *Streptomyces* sp. PsTaAH-137 | 29 | 39 | 6 | CYP1238A2 | terpene,bacteriocin |
|  |  |  |  | CYP180A | terpene,bacteriocin |
|  |  |  |  | CYP158A | T3PKS |
|  |  |  |  | CYP159A | Bacteriocin |
|  |  |  |  | CYP105D | Bacteriocin |
|  |  |  |  | CYP170B | Terpene |
| *Streptomyces* sp. Amel2xE9 | 27 | 35 | 10 | CYP105N | NRPS |
|  |  |  |  | CYP1043A | NRPS |
|  |  |  |  | CYP170A | Terpene |
|  |  |  |  | CYP1460B1 | NRPS |
|  |  |  |  | CYP105AE1 | T1PKS |
|  |  |  |  | CYP105AD2 | T1PKS |
|  |  |  |  | CYP107LF4 | NRPS,T1PKS |
|  |  |  |  | CYP158A | T3PKS |
|  |  |  |  | CYP107G8 | T1PKS |
|  |  |  |  | CYP2045A | NRPS,NRPS-like |
| *Streptomyces* *prunicolor* NBRC 13075 | 44 | 32 | 8 | CYP157K | Terpene |
|  |  |  |  | CYP170A | Terpene |
|  |  |  |  | CYP105AB22 | transAT-PKS,T1PKS,T3PKS,PKS-like |
|  |  |  |  | CYP107LW1 | transAT-PKS,T1PKS,T3PKS,PKS-like |
|  |  |  |  | CYP158A | T3PKS |
|  |  |  |  | CYP107AF2 | T2PKS |
|  |  |  |  | CYP157C | Terpene |
|  |  |  |  | CYP211D1 | T1PKS |
| *Streptomyces* sp. JS01 | 24 | 25 | 6 | CYP107BX | NRPS,T1PKS |
|  |  |  |  | CYP124G | Melanin |
|  |  |  |  | CYP1037B | NRPS,T1PKS |
|  |  |  |  | CYP107F | T3PKS |
|  |  |  |  | CYP105BN | T2PKS,PKS-like,T1PKS |
|  |  |  |  | CYP105D | T1PKS,NRPS |
| *Streptomyces* sp. CNY228 | 19 | 30 | 6 | CYP1420A | PKS-like |
|  |  |  |  | CYP107BX | NRPS,T1PKS |
|  |  |  |  | CYP170B | Terpene |
|  |  |  |  | CYP107F | T3PKS |
|  |  |  |  | CYP105H | NRPS-like,lanthipeptide,NRPS,T1PKS |
|  |  |  |  | CYP146A | NRPS |
| *Streptomyces* sp. LaPpAH-165 | 24 | 27 | 6 | CYP107BT5 | NRPS |
|  |  |  |  | CYP107CD | T2PKS,terpene |
|  |  |  |  | CYP105DW1 | T1PKS |
|  |  |  |  | CYP1046A | T1PKS,NRPS |
|  |  |  |  | CYP154M12 | NRPS,T1PKS |
|  |  |  |  | CYP208A | NRPS,T1PKS |
| *Streptomyces* *purpureus* KA281, ATCC 21405 | 22 | 27 | 11 | CYP157C | Terpene |
|  |  |  |  | CYP283A | bacteriocin,bottromycin |
|  |  |  |  | CYP121A | other,CDPS |
|  |  |  |  | CYP158A | T3PKS |
|  |  |  |  | CYP217A3 | NRPS-like,T2PKS |
|  |  |  |  | CYP159A7P | Other |
|  |  |  |  | CYP180A | NRPS |
|  |  |  |  | CYP245A | Indole |
|  |  |  |  | CYP244A | Indole |
|  |  |  |  | CYP1038A | LAP,thiopeptide,NRPS-like,arylpolyene,T2PKS |
|  |  |  |  | CYP107F | T3PKS |
| 144 | 3873 | 4457 | 1231 |  |  |

Table S5. Comparative analysis of P450s that are part of secondary metabolite biosynthetic gene clusters (BGCs) and the BGCs having P450s in *Streptomyces* species. For comparative analysis, secondary metabolites BGCs that does not contain P450s also listed in the table.

| P450 family | Total number of P450s | BGC type | Total number of BGCs |
| --- | --- | --- | --- |
| CYP107 | 254 | Terpene | 199 |
| CYP105 | 219 | T1pks | 166 |
| CYP157 | 70 | Nrps | 129 |
| CYP154 | 56 | T3pks | 82 |
| CYP170 | 53 | T1pks-Nrps | 40 |
| CYP158 | 44 | NRPS,T1PKS | 32 |
| CYP163 | 42 | Other | 28 |
| CYP183 | 33 | Melanin | 21 |
| CYP113 | 27 | T2pks | 21 |
| CYP124 | 26 | Indole | 20 |
| CYP251 | 24 | T1PKS,NRPS | 16 |
| CYP156 | 21 | Butyrolactone | 15 |
| CYP180 | 19 | Transatpks-T1pks-Nrps | 15 |
| CYP165 | 16 | T2PKS,PKS-like,T1PKS | 11 |
| CYP161 | 15 | butyrolactone,T1PKS,NRPS,other | 10 |
| CYP285 | 11 | Transatpks-Nrps | 10 |
| CYP121 | 10 | Lantipeptide | 9 |
| CYP147 | 10 | Lantipeptide-Nrps | 9 |
| CYP208 | 9 | Bacteriocin | 7 |
| CYP1064 | 8 | NRPS-like | 7 |
| CYP126 | 8 | NRPS-like,T1PKS | 7 |
| CYP181 | 8 | Terpene-T1pks | 7 |
| CYP146 | 7 | T1pks-Butyrolactone-Nrps | 6 |
| CYP162 | 7 | Ladderane-Arylpolyene-Nrps | 5 |
| CYP1279 | 6 | Lantipeptide-T1pks-Nrps | 5 |
| CYP1037 | 5 | NRPS,butyrolactone | 5 |
| CYP1192 | 5 | Otherks | 5 |
| CYP1207 | 5 | T2pks-T1pks-Otherks | 5 |
| CYP1459 | 5 | T3pks-Nrps | 5 |
| CYP184 | 5 | Terpene-T1pks-Nrps | 5 |
| CYP1959 | 5 | Thiopeptide | 5 |
| CYP247 | 5 | Bacteriocin-Lantipeptide-T1pks-Otherks-Nrps | 4 |
| CYP1029 | 4 | Bacteriocin-Nrps | 4 |
| CYP1031 | 4 | Bacteriocin-T1pks-Nrps | 4 |
| CYP1189 | 4 | Blactam-T1pks-Nrps | 4 |
| CYP125 | 4 | Ladderane-Nrps | 4 |
| CYP159 | 4 | NRPS,T3PKS,other | 4 |
| CYP1618 | 4 | NRPS,T3PKS,T1PKS | 4 |
| CYP211 | 4 | PKS-like,terpene | 4 |
| CYP245 | 4 | T2pks-Oligosaccharide-Nucleoside-Nrps | 4 |
| CYP283 | 4 | bacteriocin,bottromycin | 3 |
| CYP1004 | 3 | Bacteriocin-Nrps-Lantipeptide-T1pks-Otherks | 3 |
| CYP102 | 3 | lanthipeptide | 3 |
| CYP1035 | 3 | Lantipeptide-Terpene | 3 |
| CYP1216 | 3 | Nrps-Arylpolyene-Ladderane | 3 |
| CYP123 | 3 | NRPS-like,arylpolyene,ladderane,NRPS | 3 |
| CYP1237 | 3 | NRPS-like,CDPS | 3 |
| CYP1240 | 3 | Nucleoside | 3 |
| CYP1265 | 3 | oligosaccharide,T2PKS,PKS-like,NRPS | 3 |
| CYP1278 | 3 | Oligosaccharide-Ectoine-T2pks-Nrps-T1pks-Otherks | 3 |
| CYP1420 | 3 | Otherks-Nrps | 3 |
| CYP1423 | 3 | PKS-like | 3 |
| CYP1424 | 3 | PKS-like,NRPS | 3 |
| CYP155 | 3 | T1PKS,terpene | 3 |
| CYP166 | 3 | T1pks-Arylpolyene | 3 |
| CYP178 | 3 | T2PKS,T1PKS | 3 |
| CYP186 | 3 | T2pks-Nrps | 3 |
| CYP2045 | 3 | T2pks-Oligosaccharide-Nrps-Otherks | 3 |
| CYP1005 | 2 | T3PKS,T1PKS,NRPS-like,NRPS,lanthipeptide | 3 |
| CYP1013 | 2 | T3pks-T1pks-Nrps | 3 |
| CYP1038 | 2 | T3pks-Terpene-Nrps | 3 |
| CYP1039 | 2 | terpene,T1PKS,NRPS-like | 3 |
| CYP1043 | 2 | Arylpolyene | 2 |
| CYP1044 | 2 | bacteriocin,transAT-PKS,PKS-like,T1PKS,NRPS | 2 |
| CYP1046 | 2 | Bacteriocin-Oligosaccharide | 2 |
| CYP1048 | 2 | Bacteriocin-Terpene-Nrps | 2 |
| CYP1059 | 2 | butyrolactone,NRPS,T1PKS | 2 |
| CYP1060 | 2 | Butyrolactone-Otherks | 2 |
| CYP108 | 2 | CDPS | 2 |
| CYP1190 | 2 | Fused | 2 |
| CYP1191 | 2 | fused,T3PKS | 2 |
| CYP1198 | 2 | Indole-Terpene-Nrps | 2 |
| CYP1274 | 2 | lanthipeptide,NRPS,T3PKS | 2 |
| CYP1341 | 2 | LAP | 2 |
| CYP1417 | 2 | Lassopeptide-Nrps | 2 |
| CYP1469 | 2 | NRPS,arylpolyene | 2 |
| CYP151 | 2 | Nrps-T1pks-Otherks | 2 |
| CYP1562 | 2 | Nrps-Transatpks-Terpene-Otherks | 2 |
| CYP171 | 2 | nucleoside,betalactone,NRPS-like | 2 |
| CYP1722 | 2 | Oligosaccharide-T1pks-Nrps | 2 |
| CYP179 | 2 | other,NRPS,T1PKS | 2 |
| CYP182 | 2 | other,T1PKS | 2 |
| CYP194 | 2 | other,T1PKS,PKS-like | 2 |
| CYP2027 | 2 | other,T3PKS,NRPS | 2 |
| CYP244 | 2 | Phosphoglycolipid | 2 |
| CYP246 | 2 | T1PKS,butyrolactone | 2 |
| CYP268 | 2 | T1PKS,NRPS,butyrolactone | 2 |
| CYP1041 | 1 | T1pks-Otherks | 2 |
| CYP1042 | 1 | T1pks-Siderophore | 2 |
| CYP1045 | 1 | T2PKS,PKS-like | 2 |
| CYP1056 | 1 | T2PKS,terpene | 2 |
| CYP1057 | 1 | T2PKS,terpene,ectoine | 2 |
| CYP1058 | 1 | T2pks-Butyrolactone | 2 |
| CYP1061 | 1 | T2pks-Lantipeptide-Terpene | 2 |
| CYP1193 | 1 | T2pks-Otherks | 2 |
| CYP1194 | 1 | T3PKS,terpene | 2 |
| CYP1196 | 1 | T3pks-Otherks-Butyrolactone-Nrps | 2 |
| CYP1197 | 1 | T3pks-Terpene | 2 |
| CYP1199 | 1 | terpene,bacteriocin | 2 |
| CYP1200 | 1 | terpene,NRPS | 2 |
| CYP122 | 1 | terpene,phenazine | 2 |
| CYP1223 | 1 | Terpene-Nrps | 2 |
| CYP1228 | 1 | Terpene-Otherks | 2 |
| CYP1238 | 1 | Terpene-T3pks-Cyanobactin-Nrps | 2 |
| CYP1248 | 1 | Thiopeptide-Bacteriocin | 2 |
| CYP1253 | 1 | Transatpks | 2 |
| CYP134 | 1 | transAT-PKS,NRPS,T1PKS | 2 |
| CYP135 | 1 | transAT-PKS,T1PKS,T3PKS,PKS-like | 2 |
| CYP136 | 1 | transAT-PKS,T1PKS,transAT-PKS-like,NRPS | 2 |
| CYP1373 | 1 | transAT-PKS-like,NRPS,T1PKS | 2 |
| CYP1385 | 1 | Transatpks-Terpene-Nrps | 2 |
| CYP1394 | 1 | Arylpolyene-Ladderane | 1 |
| CYP1416 | 1 | Arylpolyene-Nrps | 1 |
| CYP1418 | 1 | bacteriocin,NRPS,T1PKS | 1 |
| CYP1448 | 1 | bacteriocin,terpene | 1 |
| CYP145 | 1 | Bacteriocin-Bottromycin | 1 |
| CYP1460 | 1 | Bacteriocin-Lantipeptide | 1 |
| CYP152 | 1 | Bacteriocin-Lantipeptide-T1pks | 1 |
| CYP1524 | 1 | Bacteriocin-Otherks | 1 |
| CYP1529 | 1 | Bacteriocin-T1pks | 1 |
| CYP1568 | 1 | betalactone,NRPS | 1 |
| CYP1634 | 1 | Blactam-Nrps | 1 |
| CYP1658 | 1 | Butyrolactone-Amglyccycl-T1pks-Nrps | 1 |
| CYP1832 | 1 | Butyrolactone-T1pks-Nrps | 1 |
| CYP1859 | 1 | Butyrolactone-T1pks-Otherks | 1 |
| CYP1917 | 1 | Butyrolactone-Terpene | 1 |
| CYP1984 | 1 | CDPS,NRPS-like | 1 |
| CYP1995 | 1 | Ectoine | 1 |
| CYP2080 | 1 | ectoine,PKS-like,T1PKS | 1 |
| CYP2134 | 1 | hglE-KS,LAP,terpene | 1 |
| CYP217 | 1 | hglE-KS,T1PKS | 1 |
| CYP2238 | 1 | Indole-T1pks | 1 |
| CYP2266 | 1 | Ladderane | 1 |
| CYP285 | 1 | lanthipeptide,CDPS | 1 |
| CYP294 | 1 | lanthipeptide,NRPS,T1PKS,NRPS-like | 1 |
| 135 | 1231 | Lantipeptide-Linaridin | 1 |
|  |  | LAP,NRPS,T1PKS,butyrolactone | 1 |
|  |  | LAP,thiopeptide,lassopeptide,NRPS | 1 |
|  |  | LAP,thiopeptide,NRPS-like,arylpolyene,T2PKS | 1 |
|  |  | LAP,thiopeptide,terpene | 1 |
|  |  | Lassopeptide | 1 |
|  |  | Linaridin-T1pks-Lassopeptide-Nrps | 1 |
|  |  | Melanin-Nrps | 1 |
|  |  | NRPS,bacteriocin | 1 |
|  |  | NRPS,betalactone | 1 |
|  |  | NRPS,betalactone,T3PKS | 1 |
|  |  | NRPS,indole | 1 |
|  |  | NRPS,lanthipeptide | 1 |
|  |  | NRPS,lanthipeptide,T1PKS | 1 |
|  |  | NRPS,lanthipeptide,T2PKS | 1 |
|  |  | NRPS,lassopeptide | 1 |
|  |  | NRPS,NRPS-like | 1 |
|  |  | NRPS,other | 1 |
|  |  | NRPS,other,T1PKS | 1 |
|  |  | NRPS,other,T1PKS,butyrolactone | 1 |
|  |  | NRPS,phosphonate | 1 |
|  |  | NRPS,PKS-like | 1 |
|  |  | NRPS,T1PKS,bacteriocin | 1 |
|  |  | NRPS,T1PKS,butyrolactone | 1 |
|  |  | NRPS,T1PKS,ladderane,NRPS-like | 1 |
|  |  | NRPS,T1PKS,other | 1 |
|  |  | NRPS,T1PKS,terpene | 1 |
|  |  | NRPS,T1PKS,thiopeptide | 1 |
|  |  | NRPS,T2PKS,PKS-like,other,oligosaccharide | 1 |
|  |  | NRPS,T2PKS,T1PKS,NRPS-like | 1 |
|  |  | NRPS,T3PKS | 1 |
|  |  | NRPS,T3PKS,terpene | 1 |
|  |  | NRPS,thiopeptide | 1 |
|  |  | NRPS-like,arylpolyene | 1 |
|  |  | NRPS-like,lanthipeptide,NRPS,T1PKS | 1 |
|  |  | NRPS-like,NRPS | 1 |
|  |  | NRPS-like,other | 1 |
|  |  | NRPS-like,T1PKS,NRPS | 1 |
|  |  | NRPS-like,T2PKS | 1 |
|  |  | Nrps-Siderophore | 1 |
|  |  | Nrps-T2pks-Otherks-T1pks-Phenazine | 1 |
|  |  | Nucleoside-Lassopeptide-Nrps | 1 |
|  |  | oligosaccharide,T1PKS | 1 |
|  |  | oligosaccharide,T2PKS,NRPS | 1 |
|  |  | other,CDPS | 1 |
|  |  | other,NRPS | 1 |
|  |  | other,T1PKS,butyrolactone | 1 |
|  |  | other,T1PKS,LAP,thiopeptide | 1 |
|  |  | other,terpene | 1 |
|  |  | Otherks-Butyrolactone-Nrps | 1 |
|  |  | Phosphonate-Nrps | 1 |
|  |  | Phosphonate-T3pks-Nrps-Ladderane | 1 |
|  |  | PKS-like,butyrolactone | 1 |
|  |  | PKS-like,LAP,butyrolactone,T1PKS | 1 |
|  |  | PKS-like,T1PKS | 1 |
|  |  | Siderophore | 1 |
|  |  | Siderophore | 1 |
|  |  | T1PKS,NRPS,melanin,T2PKS | 1 |
|  |  | T1PKS,NRPS,NRPS-like | 1 |
|  |  | T1PKS,NRPS,T2PKS | 1 |
|  |  | T1PKS,NRPS,terpene | 1 |
|  |  | T1PKS,NRPS-like | 1 |
|  |  | T1PKS,NRPS-like,lanthipeptide,NRPS | 1 |
|  |  | T1PKS,NRPS-like,NRPS | 1 |
|  |  | T1PKS,NRPS-like,NRPS,lanthipeptide | 1 |
|  |  | T1PKS,transAT-PKS | 1 |
|  |  | T1PKS,transAT-PKS-like | 1 |
|  |  | T1pks-Arylpolyene-Ladderane | 1 |
|  |  | T1pks-Butyrolactone-Otherks | 1 |
|  |  | T1pks-Ectoine-Otherks | 1 |
|  |  | T1pks-Transatpks-Terpene | 1 |
|  |  | T2PKS,bacteriocin | 1 |
|  |  | T2PKS,butyrolactone | 1 |
|  |  | T2PKS,LAP | 1 |
|  |  | T2PKS,NRPS | 1 |
|  |  | T2PKS,terpene,NRPS | 1 |
|  |  | T2pks-Butyrolactone-Nrps | 1 |
|  |  | T2pks-Oligosaccharide | 1 |
|  |  | T3PKS,NRPS-like | 1 |
|  |  | T3PKS,other,NRPS-like,NRPS,T1PKS | 1 |
|  |  | T3PKS,PKS-like,NRPS | 1 |
|  |  | T3pks-Butyrolactone | 1 |
|  |  | T3pks-Fused-Nrps | 1 |
|  |  | T3pks-Terpene-Otherks | 1 |
|  |  | Terpene, T1PKS | 1 |
|  |  | terpene,amglyccycl | 1 |
|  |  | terpene,betalactone | 1 |
|  |  | terpene,lanthipeptide,NRPS | 1 |
|  |  | terpene,NRPS,PKS-like,transAT-PKS,furan | 1 |
|  |  | terpene,other | 1 |
|  |  | terpene,T3PKS | 1 |
|  |  | terpene,thiopeptide,LAP,NRPS | 1 |
|  |  | thiopeptide,LAP | 1 |
|  |  | thiopeptide,other,furan,LAP | 1 |
|  |  | Thiopeptide-T1pks | 1 |
|  |  | Thiopeptide-Terpene | 1 |
|  |  | transAT-PKS,NRPS | 1 |
|  |  | transAT-PKS,NRPS,transAT-PKS-like,T1PKS | 1 |
|  |  | transAT-PKS,T1PKS | 1 |
|  |  | Transatpks-T1pks-Otherks-Nrps | 1 |
|  |  | 235 | 1231 |

**References**

1. Senate, L. M.; Tjatji, M. P.; Pillay, K.; Chen, W.; Zondo, N. M.; Syed, P. R.; Mnguni, F. C.; Chiliza, Z. E.; Bamal, H. D.; Karpoormath, R., Similarities, variations, and evolution of cytochrome P450s in *Streptomyces* versus *Mycobacterium*. *Scientific reports* **2019,** 9, (1), 3962.

2. Blin, K.; Pascal Andreu, V.; de Los Santos, E. L. C.; Del Carratore, F.; Lee, S. Y.; Medema, M. H.; Weber, T., The antiSMASH database version 2: a comprehensive resource on secondary metabolite biosynthetic gene clusters. *Nucleic acids research* **2019,** 47, (D1), D625-d630.

3. Parvez, M.; Qhanya, L.; Mthakathi, N.; Kgosiemang, I.; Bamal, H.; Pagadala, N.; Xie, T.; Yang, H.; Chen, H.; Theron, C., Molecular evolutionary dynamics of cytochrome P450 monooxygenases across kingdoms: special focus on mycobacterial P450s. Sci Rep 6: 33099. In 2016.

4. Mthethwa, B.; Chen, W.; Ngwenya, M.; Kappo, A.; Syed, P.; Karpoormath, R.; Yu, J.-H.; Nelson, D.; Syed, K., Comparative analyses of cytochrome P450s and those associated with secondary metabolism in *Bacillus* species. *International journal of molecular sciences* **2018,** 19, (11), 3623.
