# Supplementary material for: More P450s Are Involved in Secondary Metabolite Biosynthesis in Streptomyces Compared to Bacillus, Cyanobacteria, and Mycobacterium
Source: Int J Mol Sci. 2020 Jul 7;21(13):4814. doi: 10.3390/ijms21134814 (PMC7369989; doi:10.3390/ijms21134814)
Supplement: Supplementary file 1 [file ijms-21-04814-s001.zip › Supplementary Information/Supplementary Dataset 1.docx]

Article

More P450s are involved in secondary metabolite biosynthesis in *Streptomyces* compared to *Bacillus*, *Cyanobacteria* and *Mycobacterium*

Fanele Cabangile Mnguni 1, Tiara Padayachee 1, Wanping Chen 2, Dominik Gront 3, Jae-Hyuk Yu 4,5, David R. Nelson 6*,and Khajamohiddin Syed 1*

1 Department of Biochemistry and Microbiology, Faculty of Science and Agriculture, University of Zululand, KwaDlangezwa 3886, South Africa; fanelemngun@gmail.com (F.C.M.); teez07padayachee@gmail.com (T.P.); khajamohiddinsyed@gmail.com (K.S.)

2 Department of Molecular Microbiology and Genetics, University of Göttingen, Göttingen 37077, Germany; chenwanping1@foxmail.com (W.C.)

3 Faculty of Chemistry, Biological and Chemical Research Center, University of Warsaw, Pasteura 1, 02-093 Warsaw, Poland; dgront@gmail.com (D.G.)

4 Department of Bacteriology, University of Wisconsin-Madison, 3155 MSB, 1550 Linden Drive, Madison, WI 53706, USA; jyu1@wisc.edu (J-H.Y.)

5 Department of Systems Biotechnology, Konkuk University, Seoul 05029, Korea

6 Department of Microbiology, Immunology and Biochemistry, University of Tennessee Health Science Center, Memphis, TN 38163, USA; dnelson@uthsc.edu (D.R.N.)

***** Correspondence: drnelson1@gmail.com (D.R.N.) and khajamohiddinsyed@gmail.com (K.S.)

Received: date; Accepted: date; Published: date

Supplementary Dataset 1. *Streptomyces* P450 sequecnes identified in this study. Each P450 sequence is presented with its assigned name, protein code (in parenthesis) and species name.

>CYP105B(651285025)Streptomyces sp. Tu6071

MTETVSHAPDDLTEFPMPRAARCPFDPPEALRARQTEATLSRVRIWDGST

PWLVTRHDQVRALLADPRVSSDVTRSGFPHISPGSKAGSEVRRSFINMDD

PEHSRMRRMVTAPFAIRKMQALRPEVQRIADDLIDDLLAGPKPVDLVQAF

ALPVPSLVICALLGVPYADHGLFQRHTKVLVRRSSAPEDVSAASAALTDY

LDALLAEKQAHPEEDLLSDIATRQVATGQLTRQQAARMGVLLLAAGHETT

ANMIALGTLALLRNPDQLDVLRAADDPKTVAAAVEELLRYLSIVHSGRRR

VALEDITYAGVTIKAGDGIVLAGETANRDPEVFPDPDRLDLGRDARRHVA

FGFGVHQCLGQPLARMELQVVYSTLYRRVPTLALATDIERLEFKHDGAIF

GVYELPVTW

>CYP105N(651285285)Streptomyces sp. Tu6071

MAPVTLPTGRPAWVVTRYEDVRELLADPRASADIRRPAFPALGEGEQEVG

ARLRPFIRTDAPEHTRYRRMLLPEFTVRRVRAMRPAVQALVDGLVDTMLD

GGAPADFVPLYANAVSTSVICELLGIPRENLEFFRDVTRISGSRHSTAEQ

VSEALGGLFALLTDLIRAREENPGDDLLSRLVVQHLRTGALTLPELLSTV

GITINAGRETSTNMLALSTLLLLRRPDLVAELRADPGLMPAAVDELLRVM

SIADSIPLRVAAEDIDIPGGTVPRDEGVIALLAGANHDPEQFPHPERVDF

HRPANHHVAFGYGVHQCVGQHLARLELEVALETLIRRVPTLRLAEPEAEI

AFKHDSATFGIERLMVAW

>CYP107AE(651285327)Streptomyces sp. Tu6071

MQVVSSGPTGRSSYLVTGYAEARQALGDARLSKDTAAFFAGKGSSRALHP

ALVHHMLASDPPEHTRLRKLVTGAFTTGAVAELRPAITRITDELLDRWTP

DEPFDFVAGLAGPLPVTVICELLGVPDEDRPSIRRWSAELFAAAAPDVID

AASHSLATYMTELVASKRAHPGTNLLDRLIAAHAGDDALTENELVSLAVL

LLVAGHETTTNYLGNALLALLLHPAERDRLRAHPDAIPAALDELLRYDSP

VSTATFRFTTERLTLGGTEIPAGRPVLIALGAANRDPARFAAPDTLDLTR

DAAGHLAFGHGIHRCLGAPLAKAEAEIALGAVLRRFPAIQLAVPPEHIPW

RRTRLVRGPESLPVLHSGAEDVAPLRPRG

>CYP157B(651285548)Streptomyces sp. Tu6071

MLLDDDIPAWLALGYPEVAYVTGHDEEFGRDSRRWHQWENIPGDWPLLAY

AGYQPSVFFAEGEEHRRRAGALTRALEAMDMYDVSLVCESVGRRLIDRFV

HDGRVELLESYVHALPVRVMIELCGMPAGEDITENLAEDLRITLDAERGE

NPVEAYRRVARQLGALVAARRERPERDLTSYLVTDEAALTDTEAVHDLTV

LILAGQQPTSNWICNALRLLLLDDRFADRVAGGRVGVPEALTETLWLDTP

VQNFLGRWATRDTELGGRPVKQGDCVLLGLAAANTDPELWPHGGMGEGST

AHLSFSMGEHRCPHPSPQMARVIARTAIDTLLARLPDLAVAVEAEELRWR

RSLWKRGLEELPVVFSAGGVREWTDRGASRARSRETGLLRD

>CYP154U(651285774)Streptomyces sp. Tu6071

MGITLPGGIPAFAVTRYENLRTLILDPRVSKDPRKHWDRWPEASSNPEWG

WILGWVGVINMLSTYGVDHSRLRKLVAPSFTHHRTERLRGRVEEIVTRLL

DEIEARPEGEVVDLRAAFARQLPMEIICELFGVSGAHKAELVTLLDDFMD

VSRPVEEAMATLARLYAVLGELIGTKRAEPGDDLASDLVTMRDTDGDRLS

DDELRDTLLLVIGAGVETTVNLIGNAAFALLTHPEQLAQARAGELSWEKV

VTETLRWAPSIANLPMRFAVEDIQGPETGGVLIPRGSAILTTYAAANHDP

AHYGATAHLFDAHRDSGDHLAFGIGAHRCIGAPLAQLEAGVALPALFARF

PGLRLADPDPRQVPTFIAHGWAELPAYRTA

>CYP1240C1(651285775)Streptomyces sp. Tu6071

MGAHLVLDYRAAWEILTDQDGHWSKDPRPWEASLPSTPEAHGVLGMIGYR

PNALFTDGEVHDRFRQAVSDCFRMIQPHRLRATVTEIADDLVREFAAEGR

ADLVGQYARPLMARVINRLYGRSDASGRDLDRILTTLSNAVGADAVAANE

EFGRYMNELITAKYESPGDDLPSWFIGHPAQLAPEEVVQHVVLTLGAGNE

PSANLISNTTARMLTDERYYSSLTDGALSTREALAEVLRTDPSMANYGPY

FATRPVHFHGTWIQPAELVLVSYAGAGSDPTGLPAHADERSDGGAHLGFG

AGEHRCPAADPALLIAATAIDRLLTLLPGLELAVPFDELTWRPGPFHRAL

AALPVTFRPVRPDQPGVTPWTNSKPSLSTL

>CYP1199A(651285840)Streptomyces sp. Tu6071

MTSHTQTRPHERETLFAPPLEALLRDHLGADLFRPEPDTVGVAGPALVDR

ILAARPAGKTERPTFKPLLGRAIPHQQAATQMQAIGHDVRAALKREAPRD

PDLSGPWPHVGHVYLRDLILGGDPRRLRVLMNRALELTPKLTWTVIAAGA

ALPAPRRRTGTTRLGALSTGAATYQERRYAMGLYRRTAAPVCFTISTLVA

NALWLGAPFAAEVPSRHLVHESLRLLPPSWNILRNSSPEYPVIDARIAPA

DDVLVLPFLSHRDPALWENPEEFRPERWEELDPERVPGYLPFGHSSERCW

GRHMVMPLAEMLLDMIRSSGLVVDPGQRTARVPLTGLMGVDQVRLVQP

>CYP1972A1(651286215)Streptomyces sp. Tu6071

MTASGRLPRSHVPALASLAVRVARDPVPYYRLLHREPGPVRVPGTAGGGD

GIWLVGRDADVRAALAEPGLVADAATGDVPGLVDGAGLLAGEKVERAEGE

TGRATAAGHAPGRVLASADVRADAPPPHVAGVVARAGHVLAGRLGSRASA

DLVPEFCDWLPAVALGAALGLPYRRTEALRRWCRAGGPAPLPLPDALAVR

ARGAERATAYALASLLGTLLTRPAHLAAVRDEPALIDAVWAETLRHDPPA

PYLVLRARERVRLPGGAVAAGERVLCLLGAAGWDPAGYPDPGRWDPYRAG

PAPVPAHPWPDLGLLTVRYGLPPLLGVRGLALAPGFAPRAVGVRVRGPRR

LLVRLGG

>CYP107BC1(651286427)Streptomyces sp. Tu6071

MTESLETTSPDPTRSGNSDTGATPGYTVPKQVNDMWREKPVRRFSMRDGR

EAWLVTGRAEVRTVLADPRFSRVEARRLDAVMSPAVIFTRPGILDMDPPE

HTRLRRLVAGEFSARRMRALRPRIQQIADELIGTMKAAGPPADLAEGLSY

PLPIAVICEILGVPYADRERFRAWADRVSAPGTQPQEAMAALRSLFDYMG

GLVDDKHAHPDGSLLHGLVTARDEQGRLDNEELVTLGCGLLLAGYETTAT

MLGKGLLALLDNPDQLAVVRSDPRAVPAAVSEVLRHVTPGVDPHTGLIRA

TTADVELGGTVIPAHSVVVACNTAANFDPATFRDPDRFDVTRENAAAHLT

FGHGMHRCVGAQLAQIELEAAFAALFPAIPGLRLAVPADEITYTQSTLIR

GLRSLPVLW

>CYP161B1(651286433)Streptomyces sp. Tu6071

MAAQIQLPLDPPHPARPQHNLRALQKRAPVHRVSTAVGDPAWLVTGYQEI

KQLLADPRLGRSHHDPENAARSGASELLDGPRGDYDTEQKWIATMRSRLQ

PFFSAGHVKAMRPRVEALVGELLDRLEKQGPPADLAEALALLLPREVIGE

LLGVPVEERELLDEWTTAVSDARDRERSAAGLAALHRYGHELAARKRAHP

TQDVVSWLCEDGTLSDDQVAQLAMGVLFAGYETTATALCVGTALLLDSPE

QWRALRENPGLAPTAAEESVRVVSRLLPPVIRYTREDLEIAGAEVRRGEL

VMLDIYAATHDESIFPDPDRFDITREDNPHLCFGFGLRYCIGAPLARLEL

QAALTQLTTRFPRMRLTVPVEDLTVRPGSFAAIPTSLPVIW

>CYP113F1(651286443)Streptomyces sp. Tu6071

MTASEEGTRLADQSADAATMLAWFRRMRESEPVSHDEEAGVWHVFRHADV

ERVLADPGTFSSDFSSFMPAQEDVDRFIKGNLLRKDPPQHRKLRTLVSKA

FTPGVVARLAPRIEVIADELLDAKAGADQLELVADLASPLPVTVIAELLG

IPAEDRPMFGRWADSLVAPESQTSFIPNEDRTKAMAVVMREMNAYCLEHI

RRRRAAPSQDLVSKLVAAEVDGQRLDDDEIIGFVGLLLLAGHITTTALLT

NVILCLDEFPDIAAALRADPSALPGAIEEVLRFRTPLAPSMRRTTREVRV

GEHTVPAGRILLAWLASANRDERQFTAPDTFDIHRAPNPHLSLGHGIHFC

LGAPLARLEVRIAVEKMLGRWSGITVADGVECHDARGIVGAKKLPLRLRW

A

>CYP107BB1(651286445)Streptomyces sp. Tu6071

MDRVLDFLSSADSAGELQPRLVELSRQETLPRVLLADGQEAWLVTRNEDV

RTVLSDRSFTRDVMGERARQAGETPDGARSVNMDGRPHNELRALVSKAFT

VRRIEAMRPRIQAWTDELIDAMEETGPPADLVAHLAVPLPALAICELLGF

PVEDRQVLSGWCERITRLGEGGPDQRAWQELSAYIARRVPVERAAARGGL

APETSILTRLVHAHDSEDALSMEELLSLTVVVLAGGLETTQTAIGAGMVR

LFRNPAQLDKVRADPDLVVPAVEEILRYQPVIDVNRVQVATRTVRLGGQE

IRAGDLVQVSVNAANRDETVFPDSERCDVTRGPNPHLAFGYGAHHCLGAA

LARLELKTAFSTLLRRLPDLRPAVPLESLGWRGGHVTLGLEELPVAW

>CYP170B(651287019)Streptomyces sp. Tu6071

MLAVCAPDLVGTLLTRPEFTVGGPLWDTLEILLGKGVATSNGPRHRRQRR

TIQPAFRPERIASYARVMTEEAVRTAGSWRDGEITDVGAEMFRTGVRIVS

RSLLEVDATGTELADRIAHALHTVFGGLYQRMILSFGPLHRLPTPANRRF

NAALAELHTLVRQLVREARAHDSGEAAPDDDLLSVLLHATGENGAPLPDE

EIHDNVVSLVVAAAENVAATLAWTFHLLAQHPEWERGLREEVESVAPDRP

VDFEDLPKLTLTRNLVSESMRIRPAAWIFTRRAASDTELGGYRVPAGTDV

LYSPWALQHDPRSFPEPHRFDPDRWLPERAAEIPRHAFIPFGIGNRKCPG

DIYSLTELALVLANTVRDWHVENVPGRTDPRPRIGITLQPRATVLRVRRA

>CYP107BX(651287374)Streptomyces sp. Tu6071

MTSTLPAPLPPLHRIPSAPCAAPHPVVLPDGAAAWRVSRYADVREVLGGG

GFTRAALYAEEHDDRPSGGGIVDDPELLFNQDGPEHLRLRRTVGRAFTPR

AVARWEPWITATVEELLDGLVRHEGPLDLVSGLARPLPVTVITRLLGLEG

DAWEKIGYWSDLAFTDGSHSAAEVESGLADFTSFGGELLALRRKEPGDDL

VSGIVRAADAEGGIPESQLVRLVCGMVVGGHDSTMTMLGNVLLYLLGERR

DAWPRLTDVDAAGLAADRLLHLVPLGEDPGSLRRCVADTDLGGVRVPAGA

IVVADIVAANRDPEVFPPAETDDLFRELPAPTLAFGGGPHYCMGAWLARL

ELRTALNRLATRLPELRLVPGDGGVEWRLGTTSRGPKRLMAVG

>CYP1200A1(651287498)Streptomyces sp. Tu6071

MATSAPPQPSVDGIPNLDPVPTFVPLAARSGGGLPLIELPSGHTAVHLTR

YADVHRVLTDPSFGRTATNVEDGPSFLPTIMPKELLLNLDMPDHSRMRGF

VNADYSVAGVERLRPLVRELVAAKAARLRGAERPDLYATVLDTLPAEVNG

RFLGIPDADIAYYRPLGHTVQVASHEDVPGLVADFTELYTYLTGLVTGAR

PRIEGGVIDRFLAARDTVEPPLDDAELVGILLGSVLGADQNILSVASKIA

YVLLCRPVLWQRLAAEPGIAPRLVDELIRLIPLGNISAFPRIAGRRIELA

DGVVEEGDVVYPDAFAANRDPEVFPEPLLIDPDRTGKRHLQFGYGMHHCM

GAALARMEIAELLTTLAAEFPTLTLDTDPAGLTWDSGVILRRPVTLPVRW

>CYP107AE(651288647)Streptomyces sp. Tu6071

MSEPARDDTSPSRAAARSACPVRPVPNGPGRRDGYLVTGYAEARQALGDV

RLSKDTAAFFAGRESTRDPRPALAHHMLASGPPGHTRVRKFVTGTFTTGA

VAALRPAIARITDTLLDQWPPGEPFDFVAGPAAPLPVALAALLLVAGHET

TTNHLGNALLALLLYAAERDRLRAHPDAVPAALDELLRYDSPVSTATFRF

TTEPVTLGGTEIPAGHPVHIALGAANRDPARFAAPDTLDAAGHLAFGHGI

HRCLGALAKAEAEIALGAVPRRFPDIRLAVPPEHVRWRRTRPVRGPESLP

VRHGQPTP

>CYP107U(651288972)Streptomyces sp. Tu6071

MPRTPLPAEAPTPHAPALFSREFAADPYPSYAWLREHEPVAWTRLPSGVE

AWLVTRYEDARRALAEPRLSKNPAHHAEGAGARGKTGIPGERKAELMTHL

LNIDPPDHTRLRRLVSKAFTPRRVAAFAPRVAELTDHLIDGFAARGSADL

IHEFAFPLPIYAICDLLGVPREDQDDFRDWAGMMIRHGQGPRGGVGRAVG

KMRGYLAELIHRKRRDLGDDLISALIQAADDGEHLTENEAAAMAFILLFA

GFETTVNLIGNGTFALLQHPGQRASLHDALAGPDHGDALLTTGIEELLRF

DGPVELATWRYATESFDLAGRQIRSGDPVLVVLAAADRDPARFPGPATLD

LARRDNQHLGYGHGIHYCLGAPLARLEGKTALARLFTRLPDLALDADPGQ

LRWRGGLIMRGLRTLPVRFTPSGQGCEQS

>CYP208A(651289272)Streptomyces sp. Tu6071

MRLATRRRSARPRTGYASPPVRALPSLLRQLAGDRLAMMTAAAGYGDAVR

LPMGPKTLYFFNHPDHAKHVLADNAANYHKGIGLVQARRALGDGLLTSEG

ELWRRQRKVVQPAFQHKRIAGQADAVAQEAAALVARLRARAGHGPVRLDQ

ELTGLTLGVLGRTLLASDLGGFDGLGHAFEAVQDQAMFEMVTMNAVPTWV

PLPGQLRFRHARARLQHVVERLTESGHPKGADAGDGTDALSRLIASVGEE

TDASTGRRRLRDELITLLLAGHETTASTLGWTCYLIDRHPEVRERLHEEA

VEVLGDRLPSYEDLQRLPFTTATVEEVMRLYPPVWILPRQAQADDEIGGL

HVPAGADVLVSPFTLHRHPRFWEAPEQFRPERFLPGARGDRPRYAHIPFG

AGPRVCVGSSLGLMEAVFTVALLCRELRLEKVPGHRVVAQPMLSLGMRDG

LPVTVHPVR

>CYP154AJ1(651289297)Streptomyces sp. Tu6071

MGGGAVRRSESAPWKTLPAAPARHHGRTGIQPSSQEVDVDDDGLFVIDPA

GTDIHGEGARLRARGPVTRVLLPGGLAAWSVTGYDAARQVLADDRFAKNA

REHWPAYIEGTLGPDFPLIAWARMDNMSTADGERHTRQRHLVAGAFSPRR

IAFLRPRVERVVAGALDELAAYAAERPGEPVDLKLRYAHPVASRVIGELL

GVPDGDPDGILDRAGYVEPNPRKAAAEFAALRDKIEALVAHKRREPGEDL

ISDLIAAGSGGCPVSGAGEGSDAEVVGLAQLMLNTGAEPARNLITNTALA

LLTRPGQRALAEDGEVGWEDVVEETLRTDAPVAHLPFRFATEDVTIAGVT

IARGDPVVVGYAATGRDPQVHGAAAGEFDARHEDKRHVAFGHGVHRCVGP

VLSRMEVSIAVRELFTRFPGLRLAAEPGQLPGQGTFVMNGRLALPVHL

>CYP156B(651289958)Streptomyces sp. Tu6071

MNAHRASAPTGGGCPLHDAAFAADPRQVYEKLRAEGAAGAVELAPGIEGT

LVVDHGTALQVLRNTELFPRDSRRWRDLVEGRIAADNPLLPMLGYRPNTM

FSDGSTHLRLRKAVTDSLAKLNMSRIRRDVEPIADHLLDQFSERGRADLL

SDYAKLLPLLLFNKLFGCPADIGDTLTSSMSAIFDGRDPLRANEELTACL

MELIALKRRRPGDDITSWLIQHPSGLTDAELNDQLVMMMGAGVEPERNLI

GNTLLLMLSSSGGHDSGPQIEEAMDHVLWNAAPIANYAAHYPVQDVDFGG

HIAEANTPVLISFAGANGDPRLAEARGNQSKGAHLAWGAGPHACPAKDPA

QVIASAAIERLLNALPDLSLAVPEQELRWRPGPFHRALVSLPVNFSPAPA

NRMAAHRPQPAPGEDRQPHAAQRDTSGTGGTGGSGSTREPARKKGFWSTF

LDVFRA

>CYP107AE(651290113)Streptomyces sp. Tu6071

MQFVSSGPTGRSSYLVTGYSEARQALGDARLSKDTAAFFAGKGSSRALHP

ALAHHMLASDPPEHTRLRKLVTGAFTTGAAAELRPAITRITNELLDRWTP

DEPFDFVAGLAGPLPVTVICELLGVPDEDRPSIRRWSAELFAAAAPDVID

AASHSLATYMTELVASKRAHPGTALLDRLIAAHDGHDALTEDELVSLAVL

LLVAGHETSTNFLGNALLALLLHPVAKEHLREHPDRIPSALDELLRFDSP

VSTATFRFTTEPVTLGGTEIPAGRPVHIALGAANRDPARFPAPDTLDLDR

DAAGHLAFGHGIHRCLGAPLAKAEAEIALGAVLRRFPAIRLAVPPEHVRW

RRTRLVRGPESLPVLHSGSEGVARPRPCD

>CYP157W1(651290731)Streptomyces sp. Tu6071

MTTPVPPEGAVPPPGCPAHASAAPARPSPDYSGVPAPEGRDPATEVDNPN

IGPGRARRLNGPDAERDQQGLYDTLRLEYGEVAPVLLPRDVPAWLVLGHE

ENRFVCAHPELFTTDGREWRLLQDGTVGPDHPMAPIFMWQPVCAFVEGEE

HERLREPLEENLRVTDLRELRRLVQTGTYTLLNDLCEEGFCDLTEDYCER

LPMIVLLGLFGMSHLYDDRFLEAARDLIKGTETANASNEHLMRLFRAEVE

RARAEPGDDFASQLLAHRAGMNAREVSEHLRIVLIAAYETTANLLANAMR

ILLVQMEVRGRVGAGRLNIYEAIEQALWDEPPFSAMLGRYALQDVEVGGR

LIRKGDAVMLGYAAGNVDTRVRPELDAPVRDNRSHLAFGRGPHACPGQYL

GRQLCQLALDDLLAWFPDMRLAVPSSQLRWSGSLLSRHLVELPVRFEPRS

QRDEESIGTFGVPLPKGAGIFPPPDEEHDLMPVEVPEKVLEAEREAGEWR

AAKKRREQAEQDGPASVDAAGGAGAEAEALLPGPRRPLRDWLRR

>CYP105AC(2516515466)Streptomyces purpureus KA281, ATCC 21405

MGESIHTVTTLPTARQASCPFDPPAELLDARRHGPISRFTHPGGKPGWLI

TGYDLVRSVLADSRFSSRKELLNVGDFEVPPAPPGEFLLMDEPQHGRYRK

PLVGKFTVRRMRLLAERIERITADCLDAMEKAGPSADLVSAFAKPIPTIV

ICELLGVPYEDRGSFQEQIDTFMGGETSEEGLIAAYTATQQYLAELVAAK

RANPTDDVLSELTDSDLTDEELRGISLILLAAGFDTTANMLSLGTFALLQ

NPAQLAALRAEPSLADRAVEELLRYLTVAKSFMRTALVDVEVGGQTIEAG

TTVVLSYNTANRDPERFADPHVLDLSRQDGGHLAFGHGIHQCLGQQLARV

EMRVAIPALINRFPTLRLAVPAAEVGLRPETADIYGLKSLPVTWDV

>CYP125A(2516517254)Streptomyces purpureus KA281, ATCC 21405

MVRQTLETPMPCPALPDGFDFTDPDLLQSRIPFPEFAQLRQTAPVWWCAQ

PRGITGFDDEGYWAVTRHADVKYVSTHPELFSSTANTAVIRFNEHITREQ

IEVQRLIMLNMDPPEHTRVRQIVQRGFTPRAIRSLETALRARAHRIVEEA

RENTAADGSFDFVTQIAVELPLQAIAELIGVPQEDRARIFDWSNKMVAYD

DPEYAITEEVGADAAMELIGYSMNMAAARKECPAHDIVTQLVAAEGEGNL

NSDEFGFFVLLLAVAGNETTRNAISHGMHAFLTHPEEWERYKRERPATTA

EEIVRWATPVVSFQRTATQDTELGGQKIKKGDRVGLFYSSANHDPEVFEH

PEVFDITRDPNPHLGFGGGGPHFCLGKSLAIQEIDLIFNALADALPDLEA

VGDPRRLRAAWLNGIKELRVRVPG

>CYP157C(2516517544)Streptomyces purpureus KA281, ATCC 21405

MTTPPPPPGPTPPPGCPAHRLYGPEAEADPGALYEKLRAEHGAVAPVLVQ

GDLPAWLVLGHRENLDVARTPSRFSRDSRRWRDMQEGKVPPDHPLTPIAA

WQPVCNFQDGAEHERLRGAVTESLERLDRRGIRRYVTRFANQLIDAFADS

ARADLVRDFAEPLPMLVMTQLVGAPESYGPRLVEAARDMIKGTETAVASN

AYVTEALEQLVARKRAQPGRDLTSWLLEHSTSLTEAEIIEHLRLVLIAAF

STTANLIANALRLVLTDRRFRASLSGGHMTLPDALEQVLWDEPPLSAILG

RWATGDTMLGGQRIKEGDMVLLGLAAANVDPEIRPDLTVPVHGNRSHLAF

SGGPHECPGQDIGRAIADTGIDVLLARLPDLELAVPEDELRWDSSLMSRQ

LVALPVAFTPRGATVTSATPLPGTGAAQPLPEAAAAPAPGPQDRSPVRDS

WWSRLVGRR

>CYP107U(2516518040)Streptomyces purpureus KA281, ATCC 21405

MHDAKPPAPELFTWEFATDPYPAYAWLREHSPVHRTKLPSGVEAWLVTRY

GDARQALADQRLSKNPAHHDEPAHAKGKTGIPGERKAELMTHLLNIDPPD

HTRLRRLVSKAFTPRRVAEFAPRVQELTDRLIDGFIEKGSADLIHEFAFP

LPIYAICDMLGVPREDQDDFRDWAGMMIRHGGGPRGGVARSVKKMRGYLA

ELIHRKRENPGDDLISGLIKASDHGEHLTENEAAAMAFILLFAGFETTVN

LIGNGVYALLRNPSQRERLQRSLAAGESELLATGVEELLRYDGPVELATW

RFATEALTLGGQEIQAGDPVLVVLAAADRDPERFAEPDTLDLARRDNQHL

GYGHGIHYCLGAPLARLEGQTALATLLTRLPDVRLAVDPDDLRWRGGLIM

RGLRTLPVEFTPASTPPEADAPSRM

>CYP283A(2516518148)Streptomyces purpureus KA281, ATCC 21405

MRAENGPTPAPGQQGAGQCPVDFDFFNAPAEYRRVAAAHAREGAFYSGRG

SGFWVLTTYDGICEAFKDEDTFTVGRVSAAEGAEEERWIPLTVEGDQHTQ

WRRRLASWFTPQRVRELTPEIRRNARRRIEAFRDKGEVSFNEDFARPYVL

ENLMTAVGWPLDGLGLLLAINRAMIDSRSAPDPREAAYGELGLPALEKFA

RRHIELRRAEPADDLTTASFDWEIDGTPVSDDDRASLLCVLFLAGIDSTV

NHMANAIQHLARDEEDRARFLASREIRPAAVEEFLRTNSCMYPGRMAVHD

GAGGHAGRGQTVLLPLALANHDPEVFPEPERVDFDRERNPHIAFGTGPHQ

CLGAAFARAQILTALEEWHDLLPGYGVPPEQAGSPAPFLRNDYDLRLAW

>CYP154C(2516518304)Streptomyces purpureus KA281, ATCC 21405

MSVPPVPLSGPRFQKPLTLAARADGAAGDLLSHPRHRRLRVISLDPFVTD

LDAESAALRAAGPLAEVELPGGVRCYAVTHHAEARRLLTDKRLVKDIEQW

GAWRRGEIAPDWPLIGLANPGRSMLTVDGEDHRRLRTLVAQALTPRRVEL

MRKRITELTDGLLDALEGSAAGDVVDLKAAFAHPLPMYVISGLMGLDPAD

HPRLKVLFDKFFSTQTPPAEVLATLGELAAMMGKVVAARRAEPGDDLTSA

LILASDGGDRLTDEEIVSTLQLMVAAGHETTISLIVNAVVNLSTHPEQLA

LVRSGEVGWDAVVEETLRFSTPTSHVLIRFATEDVQVGEKVLPQGEALIV

SYGAIGRDERAHGPSAGIFDVTRPSENRHISFGHGPHVCPGAALSRLEAG

VALPALYGRFPGLRLAVPAGELRRRPVVTQNDLAELPVHLR

>CYP107P(2516519027)Streptomyces purpureus KA281, ATCC 21405

MDLAFDPSSSGFVADPYPAYTELRARGRVHWYEPTRQFLVPHHADVSALL

RDRRLGRAYPERPVPPAWQEPFHTLNDHGMLDLEPPDHTRIRRLVSKAFT

PRTVERLAPYVQGLADELVDRLVADGGGDLVAAVAEPLPVAVIAQMLGVP

EEDRPALRPWSADICGMYELNPSEEAARRAVRASAEFSAYLRELIAARRA

TPGDDLISGLIAVDLGEQEMVSTCALLLNAGHEATVNATVNGWWALFQHP

EALAALRADPALLPTAVEELLRYDTPLQLFERWVLEDIEVDGVPIPRGAE

VALLFGSANRDPAVFADPDTLDLTRRDNPHISFGAGIHYCLGAPLARLEL

TATLSALLTRAPSLTLAATPRRKPGFVLRGLESLPVTV

>CYP158A(2516520074)Streptomyces purpureus KA281, ATCC 21405

MSTTTTASSVTPSPEVHFWAVPDLTGLDFDPLLARLLHEEPVARVRLPHG

EGHAWLVTRYEDVKFVSVDPRFSRQAVWGRSVTRLAPHFIPMDGAVGFAD

PPDHTRMRRVVAKAFGARALSALRARAQEAMDGLLDTLAEHGPPADLMEG

VNRPFPLIMVSELMGVPPEDRPLMAHWSDTIISAAAGREASERAKEEMAA

YFRTLIGAPCAPGSETLAAVLAEAVEEEVLSEHEAVGLAVLIQIGGAHAV

RNNSANMVYALLTHPDQLARLRAEPALLPGAVDELLRYIPHRNAVGLSRI

ALEDVEVGGVVIPEGDPVYVSYLTANRDPSVFADPDTLDLGRPYNPHVAF

GHGPHYCPGSALARMESEILVQSLWDRFPGLRLAVPTAEIRWQRGALIRG

PETLPVSW

>CYP121A(2516520245)Streptomyces purpureus KA281, ATCC 21405

MTVEPLETLEFPLSRRGDVMPAECAALREKAPVARVRTLTGDSAWLVSSH

ALAKQVLEDERFSLRHTASEGVPRQYALTIPPEVVNNMGNINSAGLRSAV

MKALNPRAAGLTEWLRATADELIDALLTEGPPADFRAGFADPFSAALHCR

VIGVPFGEWRRLMSGLDIAFMTSAEPFDGSRVNWYKDVDLMVRRLNAPPE

RRSGLLGALAALREERESAHLTDEMLATVAVSLFGAGAVSTSSFLVLAVL

ALLQHPELIGYLREHPERMDRAVEELLRWNLSIGDGLPRIALEDVRLGET

LVRKGELVLVLVEAANFDPAVFKDPERLDLAREDNPHLSFGAGRHFCPAT

ALGRAHAATALAALVERMPELRLAVPPEQLVWRTGFIKRLPERLPVLW

>CYP158A(2516520487)Streptomyces purpureus KA281, ATCC 21405

MTSEPTTAEPPVRIWAVDDLPALDFDPLLTELLAEEPVARIRLPFAAHSE

AWLVTRYEDVRTVTSDPRFSRTALLDREVTGMTGHRVAAKESLNYADPPH

HTRLRKAVIRTFTGQNARRLRPLAQRTANELLDAMERQGKPADLMRHLHG

PLPLAVVCDLLGIPHEDRAQLASWPDVILSSGPGPRSSVAAKEQIRAYLV

QLLERRRREPHDDLAGELAESWAEGRISADEAVSLATAVLVSGAHAVRNN

SANMVYLLLTRPELMNRLRAEPQLLPEAVDELLRWIPHRNGVGLPRIATE

DVELGGVLIRAGEAVYASYLAANRDPEVFEDPHTVDFDRAAVAHMAFGHG

PHHCMGAMLTRMESEVMLSTLMARYPDLRLAGPPEETPWQSKGLIRGPRE

LLVTW

>CYP217A3(2516520707)Streptomyces purpureus KA281, ATCC 21405

MTTTTTIPRSDIDPYSDASILSPYEDYRRLRDLGPVVWLETHQVFAVARY

REVYDALHDHTTFSSGSGVGLTETLNKAQKGSSFTSDPPYHDYVRGLVAR

HLKPKALEQFHDYIEGWAARLVDELIEKGTFDAVTDFAQAFPLAVVPDLL

GWPVEEGKERLLEWATAGFNAFGPLNDRTKAGFPLLKEMSDFLQRMSIPG

NLRPDSWGAQLVSDAKDGKIEEYLLPGLLGDFLAPSMDTSVSALSSMLWL

LGSHPGQWDQIRADESLIPKAFNEIIRIESPLRGFTRLVTRERELGGLTL

EPDARVLLLYGSANRDERHWTDPDTFDIHRPGVAQHVGFGHGIHGCVGQA

LSRLEGHSLLKALSTRVRSIEIGEPTWRLHNTIRGIASLPVTLRT

>CYP159A7P(2516520837)Streptomyces purpureus KA281, ATCC 21405

MGDDPPVGIRRVGLRGAGTWPARPPTPARTKTPSPAKTPPAPHRPGSRTV

APSPLGHGVAKDPYPLYRILREEFPLTYDPDLRAWLLSRYADVAAALTDP

RFTRGHRPGDPPCAPAHQGDAAAGLRHVAERVAHVLARRLAGRHQVDLVE

DFCRWLPAGTAAAAVGVPYRDMMRLVRGRAAGSAAGRCTSLITLRQKALA

SFLANILDDPDQLAAVRTGPPELLRRAWTESLRRDPPVQIAVRRTTHEVE

VSGGTIPAGASVALLIGAAGRDPERFTAPDRFDLLRDDPGQLTFGTGFCP

AVVLAALEADFGLRALLEAMPRLRWADGFRPDHTGVITRAPRALLVRPAG

>CYP154A(2516520918)Streptomyces purpureus KA281, ATCC 21405

MGQQPIALDPAGADIHAEAERLRERGSAALVELPGGIRAWAPTGHALLKQ

LLADERVSKDPNLHWPEWIDGRYRESWINTWVGVTNMFTAYGANHRRLRK

LVSPAFTKRRTDALAPRIEEITTTLLDRMAEAPDGRAELRTAFAHPLPMQ

VICELFGLPEDRRSDTARLVEEIFDTTATPDQATATLQEILALLAGLVAD

KRATPGDDMTSALIAARDEEGSSLTEEELISTLLLVVGAGHETTVNLIGN

AVHALLAHPDQLARVLDGEIPWNDVIEETLRWAPSVANLPLRYAVEDIAL

PDGTVIARGDAILAAYAAAGRDPLRHGPTAGVFDLTRVDKEHLAFGHGVH

YCVGAPLARLEAAIALPALFARFPKLRADEEAGPARQSESFIAHGWATLP

VRLA

>CYP180A(2516521015)Streptomyces purpureus KA281, ATCC 21405

MTAATGLPDVYDPRRYATGLPHDAFRELRDHHPVARQEEYEVLGWPAGPG

FWAVTRHADVVRVLKDSTAYSSHLGATQIRDPDAADLPFIRRMMLNQDPP

DHGRLRRLVSRAFTPGRIERFEATARARARDLLRGARETGPTVDLVTAVT

DDYALLNLTDVLGVPASDRGLLLRWTERVIAYQDPDEPPVLDERGRPVNP

RSPAMLAEMFAYAQELAAYKRRRPGDDVMTSLAASPLADAELEMFFFLLT

VAGNDTVRSAAPGSLLALAEHPGQRRRLAAGEVAVDSAVDELLRWHPPVL

SFRRTAARDTELAGRPIAAGDKVVVFHASANYDERAFADPHRLDLGRSPN

PHVSFGDGPHVCLGAHFARLQLRVLHEEVRQALPDFELAGPPRRLVSNFI

NGVKSLPVRLPPSGPRP

>CYP179A(2516521293)Streptomyces purpureus KA281, ATCC 21405

MDAQSHALLDALQRDPYPLYRHARAQPGLTFVPELDAWLVARDADVREVL

LRPEDFSSAHVLRPDVLPAPAALAVLAGGLGDRPTVISTDGAAHRRHRAP

LNRALSATRTAALASFAAERAKELVRGLAEAGRRTELMESYARRLPGAVV

GRLVGLDPEDVPAAVHGSHRAEQLLFRPLPEAEQIAAAQDVVDLQHLLDR

YARARRADPKDDLCSTMVAALAPGSGELTLDQRHELVANLQNFLIAGHLT

TTALIGTTVLHLLRAPERWQLLCARPELIPAAIEEAARYDTAVQGFRRTT

TRAVTLAGTELPVGATVFVAYGAANRDPARHAHPDTFDLTRPPSRHLAFG

HGAHGCPGSQLARIQLRATLTLFTRAFPGLRLDPDHEVVMRPTLIHRSPE

ALRLTW

>CYP152D(2516521422)Streptomyces purpureus KA281, ATCC 21405

MAALVSDLGDSTAALLLKGYTWLPDRLRRGGAGPVRTRLLGRPAVALHGP

EAVGFFYDERNVRRRDALPGPVLDTLFGRGAVHTLDGEAHRVRKALFVER

LTDAEAVSALVERVREEWRRAFEARGGTVVLFDEAALVLARAVSAWAGIA

LSEAWTRRLARDAVAMVDGFATPGPRHLRARVARLRQQRRIERLVNRRRS

HGDGPGPAPTVLSAVTWHRDADGRLLDARTAAVELLNVVRPTVAIAWFVT

FAAHALHRWPEHRDRLADEPAYALAFAHEVRRFYPFAPFLGGLAARDTTW

QGTPVPEGTMLLLDVYGHNHDPALWPDPYHFHPDRFLGRAPGADEMIPQG

GGDAATGHRCPGEDITVAVLKALSAELATLDHTVPDQDLRISLRRIPARP

RSGFRMTLGGPGGR

>CYP105AC(2516521454)Streptomyces purpureus KA281, ATCC 21405

MQHEPSLTEPVTLPTQRAAGCPFDPPAGLAELREQRPLTRMTYPDGHVGW

LATGYPAVRAILGDPRFSSRYELLHYPFPGGPEGPLPPAPVGDMTGMDAP

EHTRYRRLLMGKFTVRRMRQLTDRVEEITAEHLDALEHRGPGADLVKAFA

RPIPALVICELLGVPYADRDRFQGHADTIMSMDAPPEEGYAAMAALQEYM

AHLVAAKRADPSDDLLGDLACDSDLTDEELIGIGAFLLAAGLDTTANMIA

HGTFALLTNPAQAEALRTDPDLAPRAVEELMRYLTVAPTTVRSALEDVEV

EGQMIKAGESVTISLEAANRDRERFPDPDVLDLHRKATGHLGFGHGIHQC

LGQQLARVELMVALPALLGRFPTLRLDVPADEVPLRTNMNIYGVHRLPVT

WDED

>CYP245A(2516521466)Streptomyces purpureus KA281, ATCC 21405

MSSATLPRFDLKGWDRADIADPYPVYRRYRESAPVHSGVSGTGGPQTFYV

FSYDEVVRVLSGRRFGRDARVASGDTGAASGPVPAEYAALRAVVENWLVF

LDPPRHTELRALLGAEFSPSVVAGLRPRITRLAHELLEGLGRDREADLVE

GFAAPFPILVISELLGIAREDHTWLRANAVALQEAGTTRSRGGPDRYVRA

EAAAGEFARYFRAEVRRRRGDERGDLLTLLVRARDGGAPLSVDGIVGTCV

HLLTAGHETTTNFLAKAVLALRAHPEVLDELRGAPGLTPGAVEELLRYDS

PVQAVTRWAYEDTRLGGLDVPRGSRVVALLGSANRDPARFARPDDLDVRR

PADRHLGFGLGIHYCLGATLARAEAEIGLRALLDGLPWLGLGGQRVEYAD

DMVFHGPTRLVLGTSDGP

>CYP244A(2516521470)Streptomyces purpureus KA281, ATCC 21405

MNTTTKTGLSEAPVTTMPTDPGPFDCMPQLLAAAKVAPVVRIPYLGQHAW

VVCDPELVRQALTHPKFAKDLTWVPDWMRQPGLMVGSQPDPEYARAMIMS

DGEHHARIRRLHAPVLSPRNTEKWGERVAVKVEGFLDELERSRSGATAEV

NVVTDYTHRIPLAFISEMLGLPPEAERQLRAITDVMLYSSDYPARTEAVG

ALYGAVEGWVRDPEPLADGVITGLLDSSDGPDAAVTEGEVIVWTLGMIIT

GYETTGSLISASLYEALRRPPEERPRTDEDIKAWVEEALRVHPPFPHPTW

RFATEDIDLGGYLIPKGAPVQVSIAAANRRPGEGADSFAAERRGQGHLSF

GLGMHYCIGASLVRLEAQIAVRGFLRRFPRARLSTSTAVEWESEWMIRRM

SVLPALLA

>CYP1038A(2516521490)Streptomyces purpureus KA281, ATCC 21405

MATEAPAPTPAPELSPAVVRQWRSAGEPLIELLTRAVAVGPVCVLRLGGT

TAALLTDPQGVQHVLARHPDRYVKRSHRLRALLGDGVVCAEGEPWKRQRR

FLQRHFTGQGIRRFHRAIEDAARGAAGRWEAYARTGEAFDLVEEMRWYSL

DVIWRALTGRGIDPSTQRELQTLQSIFAALPVLPGQGEQPSLAPHVARID

AVVAHTIEQARQAATHSMPTQDPGLLHTLLDAAAEHPEYTERLIRDEIVT

LIVAGHETTATTLTWLFLLLDRNPRERALALAAGPRGSAARTASLQALLS

ECLRLYPAAWLLPRHAIEDDTVAGHRIEAGTTVLSSPYFTHRDPGLWPEP

EAFRPRRFLTGEDRPTRPGAYYPFGLGPRACLGAQFALREATALLEHLLP

AFVPTLHGAPPGAAFAVTINPVGPVTTTLAPDPAA

>CYP107F(2516521958)Streptomyces purpureus KA281, ATCC 21405

MTAAETVPTCPFRHDDALEPDPFMARMRAEAPVARVRMPYGEGDCWLVTG

YREVQVVTSDRRFSRAALVGRDFPRITPAPIAQSGSINLMDPPALNRVRK

LVIRAFTTPRVEALRPWTQRTVDALLDTMAAADSPDVAAHLAEELPLMTI

CQLMDIPERDRPQLRAWAMSMMSMSAADRTAAVAAKAGLRGYFDELTRER

RRNPGEDLVSSLATARVGEEMLGEAELAVLAMLLVVTGHDTTTYDISNVA

YTLLTHPEHLAQLRERPQLLPQALEELLRFIPFRQGVGIPRVALADVELG

GVRIKAGDTVHVSYLTANRDGLVYDRPDTLDFERQDPAGHMAFGYGSHHC

LGSHLARMVLQVAIGTLLKRFPTLRLAVPAEEIQWNTVSIWRYPLALPVA

W

>CYP1062A(2516522138)Streptomyces purpureus KA281, ATCC 21405

TRTTDPLVRIGFGPVRLYLANDPALVHRIQVDTTTFERGRFFERLAANFG

NPLIASSGAPHQHQRRLLKPAFNRRAVRDHTLAVTQETEARIGAWRPGAV

VAADEEISDLVAAVVLRCLFTTQLPAHTVRDIRDTLYAIARRLLARTVFP

DALTRLPTPGNRRFAAQVGRFHTVVDTLVRERRAEGPLPEGHDVLDALLG

TVHPETGRPLSDAEIRAEFLMLLFAALETTSTSLNWAVFETATRQDVQRR

LQDEADQVLGRGPVSYAKLPELTFTRQVLDETLRLHAPMLFTRRTVTDVT

LAGIRVPAGSEIGYSPRAMHRAADLYPHPHTFDPDRAGPDQGTDRPRGAY

FPFGTGPHRCIGEHLALTLMTTALATLASRWHVRIAPGTTIREANSSLPH

VSHLPLIPTPR

>CYP124B(2514820927)Streptomyces sp. W007

MTYEPATDSATADELPVLPTVRHTGCPFDPPAALTALSDRPVRRLRYADG

HVGRLVTGHAAARAVLADPRFSSRYELLHLPMPMEGAPGELPPAPVGDIL

GLDAPEHTRYRRLLAGRFTVRRMRQLAGRIERFTADCLDAMEQAGTTADL

VEAFARPVPTLVICELLGVPYADRGRFLGLVEVIFDQAADAGARDEAYAG

LLRYVGELVLAKRAEPTDDLLSDLASPGPAPGPGSGGLAAPGPAASGLSD

EELAGIGGLLLAAGLDTTANMLGLGVFALLTDPGQLDALRADPDLAGPAA

EELLRYLSVADPLLRSALEDVEVEGELIRAGETVTVSVQAANRDPRRFPG

PDRLDIRRRATGHLSFGHGPHQCLGQQLARVEMTVALPALFARFPALRLA

VPPGEVPLRERSSIYGVVSLPVAWGEE

>CYP157B(2514821232)Streptomyces sp. W007

MGSGAVRLSGASYQQTPTELYRSLRREHGAVAPVLLDGDVPAWLVLGYAE

LSYVLTHDELFARDSRRWNQWETIPPDWPLMPFVGYQPSVLFTEGDEHRR

RAGVITEALEGIDQFELARDCRRIADRLIADFAGSGRTELMSSYVHALPM

RAVVEMCGMPVSGSDTQQLVDDLRISLDAGEGDDPVAAYGRVGDRLRQLV

KDKRAAPGPDVTSRMVTHGAGLTDEEIVQDLISVIAAAQQPTANWICNTL

RLLLTDERFAVNVSGGRLSVGEALNEVLWLDTPTQNFIGRWAVRDTQLGG

RHIRAGDCLVLGLAAANTDPEIWPESYVGAENSAHLSFSGGEHRCPYPAP

LLADVMARTAVETLLEQLPDLMLAVEPTKLSWRPSIWMRGLSTLPVQFSP

MSQ

>CYP159A(2514821233)Streptomyces sp. W007

MRQDAPLLWHEATGSYIVSRYEDVERVFKDKEGEFTTENYDWQIEPVHGR

TILQLSGREHAVRRALVAPAFRGSDLREKFLPVIERNSRELIDTFRDAGS

VDLVADYATRFPVNVIADMLGLDKSDYERFHGWYTAVIAFLGNLSGDPEV

TRAGERTRVEFAEYMLPIIRERRKAPGDDLLSTLCTAEVDGVRMGDEDIK

AFCSLLLAAGGETTDKAIAGIFTNLLRHPEQLEAVRADRTLIPRAFAETL

RYTPPVHMIMRKSATEVALGGGTVPAGSTVTCLIGAANRDEDRYRNPDAF

DIFREDLTATNAFSAAADHLAFALGRHFCVGALLAKAEVEIGVGQLLDAM

PDLRLADGFDPVEHGVFTRGPQSLPVRFTPVSG

>CYP1047A(2514821985)Streptomyces sp. W007

MSTQTGPALDTQPRGHAFVPGPKGLPLVGNLPQFGKNPLAFFELLRGHGD

MVRWRFGRNRCVFIADPDLVGELLTETERTFDQPKLGIAFRTVLGNGMLV

ARGRDWRRKRSLVQPSVRPKQVKSYATTMADCAVELADRWADGQRVDVKR

EMSALTQKIAVRTIFGVDTPADSEAMGRAMDVAQMEIGKEFAGLGALLPD

WVPTPGRARIRKAAAVIDAEVRRVVARHRGGEEERPDLLSRLLTAVDESG

THLSDEEIRDEAVTLYIGGHETTSTTLVWAWYLLARNPRVRDALAEELDR

VLGDREPGFEDYAQLHYAQAVVKETLRLFPAIWLITGIAKEGATIGGLPV

EEGTRVWSSQWATHRDARWFPEPEEFRPERWDATGGDEIPEYAWFPFGGG

PRVCIGTRFAMVESVLLLAVLARRFTLDVDPGEITPLTGLTLQPDRDLLA

TVRAR

>CYP154C(2514821998)Streptomyces sp. W007

MNCPHTAAAPADRGAGTVVIDPMVQDLDGETARLRDAGVLARIDLLGVPA

WTVTRHAEARQLLLDPRLVKDIDAWGLWQSGAVTRAWPLIGMIDAGRSMF

TVDGAEHRRLRTKTSQALTPRRLEAIRPEIEKFTQELLDALDAAQGDDGV

VDLKAVFAQPLPMRVVGMLMGVDESQHAMLTRQYKAFFSMLTPQDERLAL

LAELDVFYTELVREKTARPTDDLTSALILAEEGGEPLTEEEVVGNLKAMV

AAGHETTIGLVLNAVRALLSHPDQLRKVLAGEAGWDAVIEETLRWDTPTT

HLLMRFATEDITVGDDVIHKGEGVVVSYRVIGRDIEQHGPDADAFDITRP

TRNRHMTFGHGPHICPGAALSRVEAGIALPALFTRFPGLRLAVPDEEITK

LPVMTQNDMTAFPVLLG

>CYP157A(2514821999)Streptomyces sp. W007

MGAGTGAVALGGPGFPTEPQALYRSMRRDHGPVVPVELPGGFPAWLVIGY

RELHQVTSDGELFPRDVSLWNQWGHVPADWPLLPMVGTPMPSIYFTAGAE

HRRHVDMVVPALEGADPFEIRQHCEQLADRLIDAVCSRGTADLVAQFAEP

LPVLVLARLVGFPDDEGADIARVLKDLADGGPGAQKAHLRFGEHMRHLVA

AKRARPGDDVTSRMLAHPEPFTDEEYALDLMAITAAGHLTTADWISNSTR

LMLTEDQFADALSGGRHSVAEAMNEVLWEDGPTQILAGRWAARDTRLGGQ

NIARGDMLLLGLGAANADPHIRQQVTASAVRSGPGGNSAHLAFSHGEYRC

PFPAQEIAEIIARTGIEVLLDRLPDLELAVPATELVRRPSAFLRGTTALP

VRFTPVRTTGDAL

>CYP157A(2514822930)Streptomyces sp. W007

MTPVSGCPVTHTSVPLSGPRFQSDPVQLYRDIRRDHGAIAPVVLDGDVPA

WLVLGYRELHQVTGDPVLFSRDSDLWNQWDRIPDDWPLLPMIGRKQPSIL

YTVGERHSVRAMMISNALEGVDPFSLKRYAEEFADELIDRFCTKGSVDII

AEYAKLLPALVLARIYGFSDEHAFPLADSINDMIDGRERALAGQQHLGMS

MFQLLADKHAEPGDDVASRMLADTGGFTDEEVAQDLMVMMAAGHQPTCDW

MGNSLRLMLTDDRFAASLSGGRHSVAEAMNEVLWEDTPTQNVAGRWASRD

THLGGRHIRAGDLLLLGIAAANGDPQVRTHASALTGGNNAFLSFGHGEHR

CPFPAQETAEVIARTGIEVLLDRLPDVDLAVPAEQLTRRPSPWLRGLTDL

PVLFTPTPAVGRPGSFGGPA

>CYP154C(2514822931)Streptomyces sp. W007

MTRIALDPFVTDLDGESAALRAAGPLAEVELPGGVHVYAVTRHAEARALL

TDSRVVKDINVWNAWQRGEIPMDWPLIGLANPGRSMLTVDGADHRRLRTL

VAQALTVKRVERLRGGIEALTNASLERLAALPAGQPVDLKAEFAYPLPMN

VISELMGVDAADHPRLKELFEKFFSTQTPPEEVPQMMADLGTLFTKIVDS

KRANPGDDLTSALIAASEDGDHLSDEEIVNTLQLIIAAGHETTISLIVNV

VEALATHPEQRKKVLNGEIGWDGVIEETLRWNTPTSHVLIRFATEDIEVG

DQVLPKGEGLIVSFGALGRDEEQYGPTAGEFDATRTPNRHIAFGHGPHVC

PGAALSRLEAGIALPALYERFPELDLAVPASELRNKPIVTQNDLHELPVE

LGCPFGHGN

>CYP107L(2514823087)Streptomyces sp. W007

MTEIIDLGVGGSKFTIDPYPTYEELRARGPVHKVRTPDGQEVWLIVGHQE

AREALLDARLSKQPLPLLLGAESGSGALGNMLQSDPPRHTRLRKLVVREF

TGRRIEALRPGIERVAGELLDAMLAAPDHRADLVKALAFPLPITVIFQLL

GVPTEDVDTFHAWSTELMTPSSPMAEAATAQTVATYLVKLAEDKRVEPGD

DLLSALVSMTGEDDRLSDDELLGMCFLLLIAGHETTINLISNGVRALLDN

PAQMAALRADPSLIDNAVEEMLRFDGAVETSTDRITTEPVEIGGVVIPRH

AYVLVGLNAANRDPERFDAPAEFDIRRDPRGHLAFGHGIHYCMGAPLARM

EAQIAIRALLERCPDLELDADPAELPWVSGMLIRGVRRLPVRWTPGH

>CYP163B(2514823095)Streptomyces sp. W007

MTMATQMPGDVDLSDPQTFLDRELVPMWQRFRRESPVHWHAVEGWKVPGF

WVLSRYDDVMSVYRDNKRFTSERGNVLATLLEGGDSAAGKMLAVTDGRRH

RELRNLLLKAFAPRVLERVVEGVRRRADRLVRQGLERGDCDFAQDIAEHI

PMATIADLLGVPASDRDYLLSLTKQALSAEEAGQSGDEALVARQELLLYF

ADLAEDRREDPQSDVVSVLATATIDGEPLTEQEIVFNCYSVIIGGDETSR

LSMIGGMHELMEHPEQWRRLSSGEVSVDSAVEEVLRWVTPAMHFGRRALT

DVEIGGTTIRADEVVTLWNTSANHDEEVFDDPGTFDLGRTPNKHVSFGYG

PHFCLGAYLGRAEIHAMLTALRTQVSEVASTGPAKPIHSNFLRGYSSLPV

SMRPYR

>CYP107BZ(2514823102)Streptomyces sp. W007

MLTNDFTQDPYPILEYVRRESPVRELSMPGGGRVWVVTRYDDAKNALADP

RLSRDIHVHYELMSKRTGQPMTPPPEEANHLANLEPPRHTPLRRAISFAF

TPRRAEALRPRIEEIADELLDRLAEQPRPDLITGYADPLPVITIAELMGV

PADAWPDFLRWSAALRTYSPTDGSAVLDRSIQELSTYMSELIERKGREPG

DDLLSALIHAEPERRLTGTEILSTGFALMTGGNDTTASLLGGVLAALLTH

PAERSQLLAEPGRWGKSMDELIRYVSPISNALQRVTTEPVEIGGVTVPAG

EVVVISVMSTNRDTDQFPDHPDRLDMDRHKPAHLSFGFGIHYCSGAHLAK

VITEIGSRRLFERFPAVSLAVAPSDLRYQQNVVVRPLEELPVKLR

>CYP154AL2(2514823514)Streptomyces sp. W007

MRIELPESIPAWSVTRGDVMKKLLGHPHVSKDARKSWPGYVAGAIPWLNP

WVDVTSMFTTDGADHERLRSLVGKAFTPRRVQELRPAIEKIVDDLLDEMA

TTPAHESVDLRARFAHQVPNRLICDLFGMPEEKRAVMRRAISASLDTEAT

AEEAAATRDTMMTSMRDLVETKRREPGEDMTSDLIAAQLADGDRLSDDEM

ISTLFNMIAAGTETTASVIDWAVCELLDHPDQLEQVREDPSRWDDVVNET

LRLHAPIMHEPLRYATADIDLGEGVVIRRGEAIVVNFGRDPDLHQDPGSF

QLDRADKAHLAFGHGVHYCIGAPLSKLEANIALPALFARFRDLALAVPRH

ELQHQPLFIGNDYQSIPVYPHGLPTDA

>CYP105D(2514823601)Streptomyces sp. W007

MTESTTEPARQDPALTSPSARPASATLFPQNRDCPYHPPTGYEQLRADRP

LSRVTLFDGRPVWVVTGHALARRLLADPRLSTDRTHPDFPVQAERLANVQ

GRRVALLGVDDPEHNAQRRKLIPSFSVKRIAALRPRIQETVDGLLDAMER

QGPPSELVADFALPVPSMVICALLGVPYADHEFFEGCSRRLLRGPGAADV

DKARIELEDYLGALVDRKRVEPGEGLLDELIHRDHPDGPVERDELVSFAV

ILLVAGHETTANMISLGTFTLLRHPEQMAALRSGETTTAAVVEELLRFLS

IADGLQRLATEDIEVAGTTIREGEGVLFSTSLINRDTEVYEHPETLDWDR

PSRHHLAFGFGVHQCLGQNLARTELDIALRTLFERLPTLRLAVPAHEIRH

KPGDTIQGLLELPVAW

>CYP107CA(2514823795)Streptomyces sp. W007

MDRLDLDPTLVEVCGEHPVLRVRLPFGGDGWLVTRYADVRTVLSDPRFSR

AAAAGDHVPRTVAVAPPSTSIMGMDPPDHSRLRRRVMRAFTVRSIDALRP

RIEEIVNSLVDTMTERGAPADLATVLTWPLPITVICELLGVPPADQDRFT

EWVDGLLILDDPEQSAHARQQLGDYLAELIGQRRAEPTDDLLGELAADSG

KDPLSEEELVSLGVSLLSAGQEATANQIGNFVYTLLTRPALWRQLVADPS

LIPRAVEELSRFIPISATAGFTRVATEDLELGGQLIRAGDALVAELGMAN

RDSEVFDRPEEIDFHREQIPHVTFGYGIHHCLGAQLARVELRVVLETLVT

RLPGLRLAVPADRLAWRTERLIRGVAALPVRW

>CYP156B(2514824148)Streptomyces sp. W007

MHQQQTSLYGPEFAADPHRFYDAARTHGPAAPIELAPGVDATLIVQHEAA

LRVLQNPALFARDSRRWAALREGAVPMDSPVLPMMVYRPNCLFTDGAEHL

RLRKAVTESLSRLNSSRLSRDVERIADYLIDQFIERGTADLLNEYAKLLP

LLLFNQLFGCPGDIGDRLTRSMSAIFDGEDVLRANAELTECLMELVAIKR

RQPGEDITSWLIQHPAGLRDEELKDQLVMLMGAGVEPERNLIGNALLLML

AGDEPGAPERRGSGLLVEDALDDVLWNNPPIANYATHYPVRDIELNGVTL

KAETPVLISFAAANSDPGLTDARQTLSKGAHLAWGAGPHVCPAKSPATLI

ALTAIEKILNTVPDLSLAVPASGVAWRPGPFHRALVALPVRFTPTAARRA

GNGVQAPAQTSAQLPDPFRNAPAAPSVTPRHGQEPAKKEKGWWSSFLDVF

RV

>CYP1035A(2514824149)Streptomyces sp. W007

MGDVVRAPWNGYFVTGFDACSQVLRGRNWLAPDLAWQERQDDSKQWDAIA

TREMTTTLARLNAPEHTCQRRSLGNLFDRSTIERLTPDVERDADRLLDEL

AEKLRWGEADFVSTVSEQLPISTVGGWLGIPPEDYPDILEITHNQVFAQE

LLPTKSQLAVSAEATLRLRAYFTDLVARRRAEPRHDVLTGWIHTWDAMEP

DRERADEILYRLTMFVTIASLETTATLLTSMTSLLTEPGRWAWLRKYPEH

IDAAVDEVLRYDPPIHINTRIAAEDTVLAGVPIKKDSMIHVLYGAANHDP

RRNPDPGAFDILRGGSHLTFGGGVHYCLGAALAKLEARTLLARMLDRFPT

LHTVSRPVHAPRMVFRRITSLGVAL

>CYP124G(2514824584)Streptomyces sp. W007

MEVMGMTVPYQLSGDRVLRAADIDLADPAFWRLPRPERLRAFALLRELDA

PVLFTPRPGTARTAGKPFHALVRHADVRTASRTPQVFASAPGVTTPEPAG

WAKALFGNSMVNMDGPEHAALRRIISRRFTPRLLADVEENVGRLAGRLVD

ELIAERPRDFMPSAASRLPLEVICDLMGIPKAYRARIAEQIDHASEHVGV

ERRGRARLRIPGRGLASLARMQFVMGRLARERRRHPEDDLVSALVSADID

GEALSGQQLGAFFSLLLVAGVETTRNAIAHGLFLLDRHPEQRELLRSDFE

RYIDGAVDEIVRHSTPIIQFRRTVIAECALGGRTFLPGEKVALIYASANR

DEAVFTHPDRFDITRSPNPHLGYGGGGPHHCLGSHLARLEMTALFRELIG

RRPVMRNLGDPELVDSNFDNRVGSLPFTFGPTFT

>CYP107P(2514824760)Streptomyces sp. W007

MSFDPWSPAFVADPYPAYTALRAAGRAHWFEPTGQWLIPHHSDVSALLRD

RRLGRTYLHRFSHEEFGRTPPPAAHEPFTTLNGQGILDLEAPDHPRIRRL

ISKAFTPRTVDNLAPTVRRLAAELVDAFVAKGGGDLLAEVAEPLPVAVIA

EMLGVPEADRSLLRPWSAAICGMFELNPSEATAEAAVRASVDFSAYLRGL

IAERRADPGEDLVSALIAAHDEGERLTEQEMISTCVLLLNAGHEATVNTT

VNGWRTLFHHPEQLAALRADPALLPRAIEELLRYDTPLQMFERWVLDDIE

IDGQVIGRGAEVALLFGSANRDPERFARPDTLDLSREDNPHITFGAGIHF

CLGAPLARLELAASFGELLRKAPALRMTAEPEWQPGYVIRGLKELRAEV

>CYP107BY(2514825118)Streptomyces sp. W007

MTADPYPGYAWLREHDPVCPVGGPHVQGRMWLVTRYDDVRACLADRRLGS

GAPVNPDPHAPGLSNLDDPGHTRLRRLVAAAFTPAAVSRLRDRTARTCAH

AVESFAGRGRADLVAEYTREVPVAVMHDLLGVPEAERAPAADVLDLWYRA

KFQQPRDEVKLAELLGYVRKLVAYKRSHPGDDLPTRLIASGALTGDELEV

MVMTLIGAGHITTIQFLGTTVLRLLGHPALRAALLGGDVDWSRAINELLR

LDSPSHVAEYRYAGEDMTIADARVGEGDVVLLSLAAANRDPNRFPDPGAL

DLTRDARPHLAFGHGAHTCLGSHLVKLETEIAITTLFGRLPDLTLDIPSG

EVDWGYAPTFRGPRALPVTFTRSTGR

>CYP157C(2514825759)Streptomyces sp. W007

MTTPFHHEPGAVPPPQCPAHNLDIGPGGLRRLYGPEAENNPAGLYDKLRA

EHGTVAPILLHGDVPAWLVLGHSENLHVTRTPSQFSRDSRRWRALQDGSV

APDHPLAPIFTWQPVCVFADGAKHERQRGAVTDSMERIDTRGVRRHINRF

SNRLVNDFCEKGTADLVSQFAEHLPMMVVCAIFGMPEEYDERLVQAARDM

TRGTETAVASNAHIVSVLTRLVEARRNDPAPDLASWLVEHPATMTDTEVI

EHLRLIMIAAYESTANLIANVLRMVLIDPRFRARLSGGHMTVPEAVEQTL

WDEPPFTAVFGRWAVGDTELGGQQIKAGDALLVGIAPANTDPTVRPDLGA

DMGGNRAHLAFSGGPHECPGQDIGRAIADVGVDALLMRLPDLELGVGESE

LRWVGNIMSRHLVELPVKFAPSPQQKLDADPLSVMARAARPADAWEISSP

ARQVPEPRPEAAVAQPAHAPGAVPAPAPRPAPSPEPAPVATIPRQRRPAA

PTRLWQAVTRWWSGY

>CYP107L(2514825830)Streptomyces sp. W007

MSNEPLVDLAALGDEFTRDPYPAYAALRAKGPVHRVRIPEGADAWLVVGY

EAGRALLADQRLSKHWSRASPSLGVSKVSAGSSMLGSDAPDHTRMRKLVT

REFTPRRMEQLAPRIQEMTDGLLDSMLAAPARAADLVEALSFPLPMSVIC

ELLGVPSLDREAFRTWSGQAVSSVDPSLRASSTQAMTAYIAGLLADKREK

PGEDLLSALIHTSDEDGDRLSGDELIGMAWLLLVAGHETTVNLITNGVHN

LLAHPDQLDALRTDFTLIDNAVEEILRFEGPVETPTYRFTTDPVEVAGVM

IPGGGELVLVAMSDANRDPVRYPDGSRFDITRDARGHLAFGHGIHYCLGA

PLARIEARIAIRSLLERCPDLRLAADPATLAWRTGILMRGPLSLPVAW

>CYP107L(2514825831)Streptomyces sp. W007

MAVLDLRDLPDFTVNPYPYYAKLRAEGPVHIVRTDEMERIWLVVGYEEGR

AALADQRLGKDWRTTGRWEASEAVLSANMLELDAPHHTRLRRLVVREFTP

RRIEALRPRVTEITGRLLDAMVPRGSADLVDALAFPLPMTVICELLGVPD

IDRDAFRALSNGIVSPTPEQREGDPAGAMGDYLVDLIADKRRSPGDDLLS

ALIRTRDEGGDSLSSAELVGMAFLLLVAGHETTVNLIANGVRALLDHPDQ

LALLRADPGLIDSAVEEMLRYDGPVETATFRFAREPVKIGDTEIARDEAV

LVALASADRDPGRFPEPDAFDIRREPQGHLGFGHGAHYCLGAPLARMEAR

IAIGALLERCPDLARDPSGGELDWLPGLLMRGVRGLPVRW

>CYP107F(2514826273)Streptomyces sp. W007

MENTSVQNKETVRNCPFDYAQQLEFDPQLRQLLTEEPVSRIRMAYGEGEA

WLVTRYEDVRTVTTDRRFSRSAVLGRDFPRMTPEPIVQAESINLMDPPAS

SRLRGLVAKSFTPRRVEQMRGGTQRVVDRLLDEMEEEGSPADFVARVSAP

LPLITICEALDIPEADRPWLRAHAMTMMNVGAAGKQDAVRAKAELRGYFQ

ELTADRRRSPGEDLISTLATARDGDELLDDDELAVMAMVLLITGQDTTTY

QLGNIAYTLLTRPDLMRSLRAEPQRLPRTLEELLRHIPFRKGVGIPRIAL

EDVELSGVLIKAGDVVHVSYLTANRDSAKFDRPDELDPDRPTIPHMTFGW

GAHHCLGAPLATMELEVAFSTLLTRFPALRLDVPPADVSWNTTSIWRYPL

ALPVTW

>CYP107U(2514826323)Streptomyces sp. W007

MHRTVLPSGVEAWLVTRYGDAKEALAEARLSKNPANHAESPHAKGKTGIP

GERKAELMTHLLNIDPPDHTRLRRLVSKAFTPRRVAEFAPRVQELTDRLI

DDFVEKGNADLIHDFAFPLPIYAICDLLGVPEEDQDDFRDWAGMMIRHGG

GPRGGVARSVKKMRGYLAELIHRKRENPGDDLISGLIRASDHGEHLTENE

AAAMAFILLFAGFETTVNLIGNGTYALLRHPEQRARLEASLAAGEDALLA

TGIEELLRFDGPVEMATWRYATEALTLGGEEIAAGDPVLVVLAAADRDPE

RFTDPDTLDLARRDNQHLGYGHGIHYCLGAPLARLEGQTALATLLRRLPD

LRLAGEPDDLRWRGGLIMRGLRTLPVEFEPGRRSEKSDTVSTL

>CYP107MU1(2514826331)Streptomyces sp. W007

MDLGSDADRFRSDPYGLFAELYKDGPAHFVRLPNGETSWLIVGHSEVYRA

LNDPSLSKSRPRAEGSPGPRGNMLMTDPPDHTRLRRLVSRQFTARRVERL

RPEVRRATDELLSRVEHRGRMDVIQDFALPLSLDVITGLLGVPFLDRDAL

RAWTDDIIIPKGPERSRQVYQEMTAYIDRLIEAKRRQLGEDLLSSLIRAA

DDENGRLDHQELHSTFFLILVAGHETTTNLIANGVYALLTHPDQLKLLRA

DWDLIGRTVEEVLRWEGSLTFATSRYPTSPYRVGDVEIPADGSRILLALG

SAGRNTAVDADADRFVIDRPGRTHLAFGHGIHHCLGAPLGLLEGGTALQS

LLTRFPDLTLDTDTVEWRASMVSRSLERLPVRWSPPSAP

>CYP1046A(2514826433)Streptomyces sp. W007

MGGLAHSRALRFWLDPANLAARLDRAGPVVPTRTGPATAFQVNDPALLRK

IGSDEDTFRFWGPDPSLRDFNENGVVGLEGAAHRERRAVMRPAFTASRLT

ALGPSAQARTLRLLADLPADRPLDMRVEMSRLACGLLVSCVLNSELTPDT

LSKVAAARSTLSGGMFWRYALSPWPWVPVPRRRACRRALAELDEAVRQVR

ARHRPNPDGQDLVSLLEAAAPGNPHVVQRDIRALLIAGMETSASTLAWAC

YELGRNPHYQQALQEEADATPDSSRLQAHQLPLATAFIQEVTRLHGIPFL

VRRTRHQTCQGGVQIPAGAVVTLPLGALRRDRNRYRDPDVFDPQRWLPHA

DPPPAPTALLAYGLGPRYCPGAAAADAMLPVALATLAGSRTLRPARPGRK

VGVSLELTPTPKGLTMYATPCESRLADTRSAPDTNTGSGSGSGSGSGAPS

TEKGHA

>CYP107BX(2514826461)Streptomyces sp. W007

MSTIDPTQPAPEPMHRLFFEESGPPRPAELPGGGPAWLVSRYDDVRQVLS

DPRFGRAQLYAPDAPALSDVPDLVNDPDLMFNQDGPDHLRLRRTLRRAFT

PRAVARWRPWIAAIVDELLDRLETRQQPVDVVAEFTLPLPVAVISRLMGI

NGSAWDRMRHWSEHAFSDGTHEGEQVASVLKEFGAFGADLLTERRRTPGE

DLISSLVAAADEEGGVPEAQLVSLVCGLVVGGHDSTMTMLGNALLYLLGE

RRETWPRLGADEEAAGRLVDRLIHLVPLGDDRGSTRHAAEDVEIGGVTIP

AGAIVLADCGMANRDPEVFPPATLHDLFAPLEAPTLSFGAGPHYCLGAWL

ARTELQLALHRLAARFPDLRLTAPMDTVAWRTGTTSRSPRRLGVRW

>CYP125A(2514827771)Streptomyces sp. W007

MPMRCPHLPDGFDFTDPDLLQARVPHPEFALMRETAPVWWCTQPHNISGF

GDEGYWAVTRHADVKYVSTHPELFSSNTNTAVIRFNETISRDQIDVQKLI

MLNMDPPEHTRVRQIVQRGFTPRAVRSLETALRSRARSIVETAQASADTD

TGGSFDFVTNIAVELPLQAIAELIGVPQEDRSKIFDWSNKMAAYDDPEYA

ITEEVGAEAAMEIVAYSMNLAAARKECPAQDIVSQLVAAEGQGEPVLGRV

RVLRDPARGGRERDDP

>CYP107L(2526036341)Streptomyces sp. TAA486

MAADGIVELTQLSPDFVRDPYPVYAALRAEAPVHHVRVPDGRELWLVVGH

ETCRTAFTDPRLSRDWQNHGGIGQILNPGLPAIGHMLTTDPPDHTRLRRL

VAGSFTPRRIAALGPRVQQITDELLDAMLEKEERRADLIASFAFPLPMTV

ICELLGVPELDRQAFRGWSNAMVGPTSAEEEARAYEEMPAYLTGLIETKR

ETPGDDLLSAMIQEVDDGGDRLTGDELIGMCVLLLVAGHETTVNLIGNGM

RALFAHPDQLAQLRADFGLLEGAVEEMLRYDGPVETCTQRLALEDVELDG

TIVPAGSTVLIAMADADRDPERFEEPHCFDIRRDARGHIAFGHGLHYCLG

APLARLEGRIAVRSLLERCPEIAADADESTLRWLPGMLMRGVRRLPVRW

>CYP183H(2526037154)Streptomyces sp. TAA486

MPNTISPPADRTWRTGKAPGALPLLGHVLPLWRDPLGFLASLPAHGDVVE

VRIGPGRAYLVNHPELVRHVLLHPRVFDKGGIFDKARQLLGNSLSVSRGE

EHRYQRRLVQPAFRPEKIAEYTRAVADDTNSAIAEWSPGEVRNISDEMHA

LLMRVAARTLFSTGLDDATIEEARHCLRTVSLGIYKRAMAPLGVMEKLPT

PANRAYDHANTRLREIVATMIDTQRRSPSDRQDLVSALLRAEHPVTGVRL

TDRELLDQVVTFLIAGSETTASTLAFVFHLMGAFPEVEKRVHAEIDEAVG

DRAPAYEDLPSLPCTRNVITEALRLYPPSWMAMRVAVEDVELGGLQVRSG

AMVLYSAYAMHRNPELFPSPEAFDPGRWQADRAAQVPRGALLPFGAGTHK

CIGDVLALTETTLIVAAIAASWRLRPVPGTRLRPEPRATLEAGPLPMVCE

RR

>CYP154L(2526037155)Streptomyces sp. TAA486

VTLSLHDCEAVAIDPSGADIPAEAARLRALGDLVPVELPGGIRVWAPTRH

HILKSFLTDPNVSKDPRLHWDAWRSGSLHENPEAHWIYNWVGVENMLTAY

GDDHSRLRKLIAPAFTARRTEALRPAVETITADLLDALRATPPGETVDLR

AHYALPLPMRVICDLFGLVDGERAQVAAFVEVMMDTTTAPEIAGKMLANA

REALTQLIDRKRAEPGEDLTSALIAAHDGQDRLSETELVDTLILLLAAGH

ETTVNLIGNAVVALLRSPDQLAALRAGDVPWNNAVEETLRWAPSIANLPL

RFAVSDISVGSVTIRAGEAILATFGAAGWDPEFHGADAHRFDVRRAPSEH

LAFGHGAHRCVGAPLARAEANIALPALFDAFPAMRLAEEEFEAVPSFVAH

GHRTLKVFLRP

>CYP157A(2526037402)Streptomyces sp. TAA486

VTTPDPQSASDESRAATGDSADGRAAPSSGCPAHPDAVPMFGTRFISEDR

GKLYREMREQHGPIAPVTLVGDVPAWLVLGYRELHQITGNPGLFTRDSSV

WNMWDEIPPDWPLMPMVGRQPSILYEVGARHERRSTLVSDALREVDPFEL

RTHSEQHADELIDAFCGSARADLVDQYAMQLPALVLARIFGFTGEDGAAL

VPSINAVVDGSDGAIEGRQYLQRAMGTLLRSRAIAPAGDVATRMQTHPNS

GEFSSEEILEDMMVDLIAGHQPTADWISNSLRLMLTDDRFAASLAGGRAS

VGEAMNEVLWEETPTQNIAGRWTTRDTQLGGRRIQGGDLLVLSFAAANAD

PHIRPDQHSFTGGNSAFFSFGHGSHRCPYPAQEIAEGIARTGIEVLLDRL

PDVDLAVDEEELAWRPSPFLRGLESLPVTFTPTSAVGGR

>CYP154C(2526038451)Streptomyces sp. TAA486

MTAPASPSSSAGQVGCPVVIDPMVRDLDGETRRLRDAGTLTRIELLGVPA

WSITTHAEARRLLTDQRLVKDINQWTLWRTGEVTHEWPLIGMIDAGRSMF

TVDGAEHRRLRTKTAQAVTPRRLENLRPVIEGITHRLLDDLEVQGKEGAV

DLKSVFALPLPMSVISELMGVDPALHPRLHVLYKAFFSMLTPQDERLAVI

DELDVIFTDMVRARTADPRDDLTSALILADEGGEPLTEEEVVGNLKAMVA

AGHETTIGLILNAVRALLTHPEQLRMVLDGDVGWDAVVEETLRWDTPTTH

LLMRFATEDINVGEGVIQEGEGVVISYRAIGWDPEQHGPDADRFDIARPS

PNRHMTFGHGPHICPGAALSRLEAGIALPALFDRFPGIRFAVPVEEIVNQ

PVLTQNDLRSFPVHLHGD

>CYP157A(2526038452)Streptomyces sp. TAA486

VTAYQHAHQGDYGYGMPAPPPECPAHASDKAVPLSGPRFHTDPSGHYREM

RAEHGPVVPVLLPGDVPAWLVIGYQEMYQVTTDAKLFPRDSGLWNQWETL

PEDWPLRPMIGQRQPSIYYTVGAEHQRHLEMVQTAMQNISALELRTHCEE

LADQLLDGFCARGEADIIAEYAKPLPILVLARLLGFPDSDGPGLIKAMSA

LADGGADALEGFQYFGAKIHELVAEKQAAAGADLTSWMLAYPEYFTDEEY

ALDLMAITAAGHLPTADWIGNSIRLMLTDDRFSASLTGNRRSIPEAMNEV

LWEDTPTQILAGRWAAWDTWLGGQRIRTGDMLLLGLAGANADPKVHVDAA

GADTALRGGNSAHFAFSHGEFQCPFQAQEIAETIARVGIEVLLDRLPEID

LAVPAGALARRPSAFLRGMTALPVRFAPTPPIEGKP

>CYP159A(2526038517)Streptomyces sp. TAA486

VPTAPEAPDILSPQFAADPYSAYRVLREYFPLLWHEGTGSYIISRYEDVE

RAFRDEVFTTRNYDWQLEPVHGRTLVQMSGREHAAHRALVAPAFRGRMLE

EKFLPVIEQNARDLIDPLRDAGSADLVEDFATHFPINVIVDMLGLDKSDH

PRFHGWYTAIVAYFSNLSQDPEITRAGHRTRDELAAYMIPVIRERRENPG

DDLLSVLCEAEVDGTAMSDEDIKAFISILFAAGGETTDKAISSLFGNLLR

RPEQMAAVQEDRALIEQALAETLRCTPPVHMIIRETDNDVELSGGTVPAG

STVNCLIGAANRDATRFADPDSFDIFRTDLPTRSAFSAAARHLAFALGRH

FCVGALLAKTEVEVGVGQLLDAMPELKLAEHCELVERGIFTRGLESLPVR

FTRAVRR

>CYP156Q2(2526038972)Streptomyces sp. TAA486

VTSSPLSEPHAAPPGCPAHRPSAGTTGSDLPKLYDDDFAADPNALYDRLR

SQGGPAQWVELAPGVESILVTGYQAALEVLRSPYFSKDAHRWKALADGHV

PRDSPILPIMGPRKSLWFADGQEHLRLRTPVDRALAGIDPRQLRAVVQRS

ATELIDEFTEDGKADLVSQYAARIPVMVLTQLFGCPDHLNPRISRAFLQM

VNAVEPDAATQGVQDLVACLNELIELKYRTPGDDVTTRLLRHSANVTREE

LIDQLVVITGAGQVPGTAWISTATMMLLSDDRFAGDLTGGSLTVTDALNE

VLWLHAPHSNYSFVYAIEDYVLRDQDTGEETLIPTGVPVTISHAAANLDP

TLPTNQAERAANTSHLAFSAGPHACPAQDTASIIAEVAIDTVLDLLPEME

LAVPAEELSWRPGPFIRALTELPVRFTATPATHAAEAAAATSSTTSEPSG

TSSDAAAGHPPPDAPRGSRRRRWSLAGRRGR

>CYP124G(2526039139)Streptomyces sp. TAA486

MPPHTASASSAGPEAAAAPQAPSPAAAAAGVEASEAAGAFEAAGLPPETN

PADPRFWQLPHDQRHQVFAVLREREAPTYFAQPQHSLRKRQQPGFYALVR

HEHVQEASRNAAVFGSAPGVTTPHPAPWVKALFGESMVNVDGSDHAKLRK

IISRAFSPRLLAGTEKDIEQVVAAVVGNAVRQPSREFVTAVASLVPCEVV

CNMMGIPQRHRPRILAQLNDASENIGVRRGAGAKFRIPGKGLRALARMHV

MIAMLARKRRRHPTDDLISALVTADVDGQCLGNRQLGAFFTLLMVAGVET

TRNAISHGLALLSANPEQRELWKSDFEKYADTAVDEIVRCSTPIIQFRRT

LRTDHVMGGRTLRKGETVAMYYASANRDEAVFTDPGTFDITRDPNPHLGY

GGGGPHYCLGAHLARQELKALFRQLLTDSVAMRAVGLPDLVPSSFDNRIR

SLQFDIAPQNPAAARRSAL

>CYP157C(2526039897)Streptomyces sp. TAA486

VTSPPPDPDAPGRPDGFGPAPAAPGAGTPSAHTPGESVTGSPAAPGFDVF

DPSPAPPPGCPAHGLGPDGVRRLYGPEADADPIALYERLRSEHGAVAPVL

LPGDREAWLVLGHRENLEVSRTPSLFSRDSRLWRDMQEGKVPPTHPLAPL

TMWQPLCVFAEGEEHERLRRALTESLARFEARGIRRYVTRFAHQLIDEFE

PKGRADLVEQFADQLPLRVITQLFGMPEHHGPRLIDAAKDMIRGSETAVA

SNDYITEALQDLVDRKRASPGHDVASKLIEHPARLTNDEVREHLRVVLVS

ANEPTVNLIANTLRLVLTDQRFRATLAGGHMTLPDALEQVLWDEPPMTTN

IGRWATGDAQLAGKRIKAGDMLLLGLAAGNADPQIRPDKSTPMHGNRSHL

AFSGGPHECPGQDLGRAIVDTGIDTLLTRIPDLRLAVPEEHLKWETALMS

RRLVALPVEFAARRTNTAGRQGPGVPSVELPPLQPQEPASVPMPPVAPPP

VHTSWWTRLKRKLRGW

>CYP251A(2526039899)Streptomyces sp. TAA486

VSAALKAVPLAPNRLPLIGHALPLLRQPLDFMKSLRDTGDLVRVDVGTLP

IYFVTSAELTHELLVTKARNFDKGRFFIRARTLVGDALPTAPSDIHKRHR

RLMQPMFHHARIAGYANVMATRANAMAESWKPGETVAVDEELAQFSVATL

AETMFSAEISRPAAEAVIRDVPILLKNALVRTVTPRLMDRMPIPANRRFD

TAATRLRKVIDDVIAISHQQGDDDNEDLLSLLMAARDADTGDTLSDREVR

DELVAIMFAGTETTASTLAWTFHELATHPEVEDQLLAEIESVLGDRPVTF

EDVPKLEYMDRVLHEISRMHSVPLLMRQATAPVELGGTEMPVGTELAFSL

YALHRDARLYEDPERFDPDRWLPEHGAKVPREGYIPFGAGNRKCIGDGFA

WTEIVITLATVLRRWRFQRVPGHTVKEVASAVAHPDSLPMTVLPREG

>CYP102B(2526040260)Streptomyces sp. TAA486

MAQAQASERGARRKGFRSARLDWPELHRIPHPPRRVPLLGDVLGASVQSP

VQDSMRIGRELGPIFRRKAFNREIVFVWGADLAAELADESRFAKHVGLGV

ANLRPLAGDGLFTAYNHEPNWQLAHDILAPGFSRAAMEGYHPLMLDVARQ

LTARWDELSGAGDAVDVPGDMTKLTLETIARTGFGHDFGSFERSRPHPFV

DAMVGALSYAQRRNAAPPLVGPLLQRGAAKRNVVDREYLNSTVDQVVEAR

RGSDGSGARGDLLDRMLETAHPETGERLSPENIRRQVVTFLVAGHETTSG

ALSFALHYLSRNPDVLARAQAEVDEVWDGTEEPAYEQVARLRYLRRVLDE

SLRLWPTAPAFSREALHDTTLGGVHPLRHGAWALVLIMLLHRDPAAWGEN

AEVFDPDRFEPKAVRARPAHVFKPFGTGARACIGRQFALHEATLVLGLLI

RRYDFRSNPDYRLRVDERLTLMPRALRLHLTRRTPAQTPAAAPA

>CYP105AB(2526040448)Streptomyces sp. TAA486

MTSDQPTVTLPVPRTCPFSPPVEYERMREDSPVSRVGLPGGRTAWALTRH

EDVRVMLADPRFSADRTRPGFPVLAEGQQQLAARFKLSLISMDPPEHGPA

RRAVVGEFTVKRMAALRPRIQQIVDEHVDALLAGPRPADLVRALSLPVPS

LVICELLGVPYADHDFFQSRSARLLRRTEPMASREQARDELRTYLDALIT

QKEKNPTDDLLGRQVLKQQESGRGDHEDLVSLAVLLLIAGHETTANMISL

GTLALLKHPGERTRLGEDPDAMPEAVEELLRYFSIVDAVTSRVATEDVEI

GGVLIRAGEGVVGLGNSANHDPGVFSRPGELDLGRGDRHHVAFGYGAHQC

LGQNLARMELEMVFETLFRRVPGLRLAAPVDELPFKDDSNIYGLYELPVT

W

>CYP154T(2526040452)Streptomyces sp. TAA486

MTADQASPTSGVPVLELDPHGRDHMGEAARLRGLGPVVRVKLPGDVEAWS

VTRHNLLNELVMDPRISKDWHNWGAMQRGEIPDDWPLIGMVKVTNMVTAD

GAEHRRLRKLVSQTFTARRVEELRPTTAEIVENLLQALPSHADSDGTVDL

RKHFAYPVPMQVISDLFGIPDHERPELREAVDKIFRSDLSPEVVGANQIA

VYQLLARVVELRGKERGDDLTSALISAREAEPDALTQEELVGTLLVMLSA

GHETTLSLIVNAVRALLTHPDQLALTRGEEDMWPAVVEETLRWEAPIGNF

PFRYATEDIDVAGVTIPAGDAIMAPYSAVGRDPQQHGPDADTFDISREQA

RHLSFGHGVHACLGAPLARLEATIALRELFARYPDMILGAVPGTLDPVPS

MFSNSVTSLPVRLGRPAG

>CYP157J(2526040453)Streptomyces sp. TAA486

VTTPAPTRPFDSETLVSLTDITQSRDPHRIYHQLRLQHGPVAPVLLEPGV

PGWLVMGLEQLRIITEREALFSRDARNWRDLNDGIVSPDSGLLPMMAYRP

NVIGADQQEHRRLRQPLDDGVGRINHRALRRQVQRICKELINTFAQFGRA

DLVADYAAYVPMLAVASLFGLDTRQGHELRAALIALFSSQTDSQVGNRSF

EQILFDTLRERQQNPTEDLTTALMNHPNLQDDPEVLQSMVVMISAGNETT

TCWIAHTLYRMITDTSFDDRLRSGQLGVDDALDEVLWREPPMTHMPARYA

LRDIELGGQSIRHGDVLILGLSAANSDPALAPGGHRPPNNRAHMAYSAGP

HMCPAQDTSRVITRTAVDTALHLLPGLRLTVTPGEITWNPSPWTRCPTQL

PVAFAVPHEIRTPPASAARPIGETV

>CYP1240B(2526040490)Streptomyces sp. TAA486

VTPTPEPGPPVSGTPGPPVSGTPGPPEPGAPEPSTSGPGTPRRPAPADPV

TSARAAPGAEPHDAERPGTESHGEPVRLYGPEFAADPQAVYDRLRQYGPL

APVEISPGVAATLVTDYRAALDLLHDPVTWSKDSRLWQEKLPADSPILPM

LGWRPNVMFNDGDEHARYRQVITDGFDRIPSHELRTMVVSIADSLIAGFG

ADGHADLMGQFARVLPLKLFNRLFGLPDSSSDRLISSIAGMMEGDPAEAS

AASAEYQGYIGQLIAAKKRVRGRDLTSWFLDHPAGLSDEEVTHQIVLTMG

AGHEPTTNLIGNALSRMLTDERYYSTLSGGALTPRDAIQDVLLNEPPISN

YSAHFPRRDVYFHGTWIRTGQLVMVSYAAAGAAHGRAGAGASGSGASGSG

GGAHLAWSAGPHSCPVPRPALLIATTAIERLSAWLSDIELTVPHDELAYR

PGPFHRALVRLPARFTPISPDQAGATPWDSRESRHTKGGRSPGSSEGGER

PAEAPGVPGRS

>CYP107U(2526040985)Streptomyces sp. TAA486

VHDETDEHAATGPAPKHADTAEPAPGHVAATGPAPERAADPAPGHADPRT

ATGARAHAPTRPDGEAGADAPDRGPACAHATALPPPPMPPLFDWDFAADP

YPAYAWLREHAPVRRTELPSGVEAWLVTRYADARQALADQRLSKNPVHHH

ERSAHGKGKVGIPGERSANLMTHLLNIDPPDHTRLRRLVSKAFTPARVSE

FEPRVRELTDRLVDGFAARGEADLIHEFAFPLPIYAICDMLGVPAEDQDD

FRDWAGMMLRHHKPGEPGPPRGGVGRAVKKMRNYLAELIHRKREKPGEDL

ISGLIRASDHGEHLTENEAAAMAFILLFAGFETTVNLIGNGTYALLGHPR

QRSLLRDAVTAGDERLLATAVEELLRYDGPVEMATWRFATEPLEIGGARI

AEGEPVLVVLAAADRDPVKFADPDVLDLERRDNQHLGYGHGIHYCLGAPL

ARLEGRVALSALLRRVPDVRLAVPPSELRWRGGLIMRGLRTLPVEFTPEP

GTE

>CYP180A(2526042102)Streptomyces sp. TAA486

MASPTAVPDVFDPRVFARGIPHDSFRRLRDEAPVCRQDEHEVDDWPAGPG

YWAVTRYQDVREVLRDPAVYSSWLGATQIRDPAPDDLGFIRRMILNMDPP

EHNGIRRLAAAIFTRRRLEQFSEQITARAAALLDAVAGHGSCDLPVEVTD

DFPLANLAELLGVPASDRSLLLKWTNRVIGYQDPEHAEVVRDADGRPVNP

RSPAMLRDMFDYAHELARHKRRRPADDLMTALATATVEDGRLLTEEELAM

FFFLVVIAGNDTVRSALPGGVLALLEHPDAYRWLRADPALVPSAVEEILR

WHPPVLSFRRTVTRDTELAGQRMARGEKVVVYHASAHFDERAFPDPYRFD

ITRTPNDHLAFGQGPHMCLGAHFGRMQMRIFFTQLLARLPELELDGPPVR

LSSNFINGITHLPVRWTPTHPDSRPPLTE

>CYP228A(2547444467)Streptomyces lysosuperificus ATCC 31396

YRLLRSIAARGPVVRVPRVGVVVSDAALARDVLCDTAHFTKTGPGSPSDL

WTPVLGPAVLLNMEGPDHTALRRRLSGLFTPGYVTGLCDRVLAQPLAGLT

RRLAAGEEADLVSVAKSCAGAAICEITGMPAEPERFAAAHSDASEVTKAV

RLWRHSLPPRQIRHAREVLGRLTASAVDAYRRGGDDTLPGRLRGLGLTEE

EARGAVGAFLLTGTETLVSFIP

>CYP152D(2547445796)Streptomyces lysosuperificus ATCC 31396

LRARHARRRQEHDLAQLIVRVREQRETAPDGSALDVVARHRDADGSLLDP

HLAAVELLNIIRPTVAIAWFATFAAHALHRWPQHRGALRGDASGHLATRF

AHEVRRFYPFAPFVGGLAARDLEWHGETVPEGTLLLLDLYGQHHDAQWWD

HPYRFDPHRYDTGESLRHLIPQGGGNAAAGHRCPGEDITVTLLAAFVTEL

ARLDVSVPDQDLTIPLSRIPTLPRSGFRFRLG

>CYP124G(2547446442)Streptomyces lysosuperificus ATCC 31396

MTLPAQRHGTDPGRELAEPGFWQQPPAHRLAAFARLRAAEGPVFVPEGAG

HWALVRHAHVQEASRLPKVFASAPGVTTPEPARWVRVLFGDSMVNLDGPD

HAQLRKIVQRAFTPRLLAAAEADIHAVAARIVDDVLADRPDEFVSAVASR

LPLEVICNMMGIPERFRAEIADRVNHASENIGVERGLASRLRMPGRGLRA

LARMQRMVAGIGRERRANPTDDLISALVCANVDGQALGARQLGAFFSLLM

VAGVETTRNAITHGLTLLTDHPDQRDLLLSDFETYADGAVDEMIRHSTPI

IQFRRTVVAEHTLGGRVFRPGEKVVLYYASANRDEAVFPDPDAFVITRSP

NPHLGFGGGGPHFCLGAHLARVEMKALFRELLTRPVGLRAVGLPDLAGSN

FDNRVRSLRFAFERP

>CYP134A(2547446810)Streptomyces lysosuperificus ATCC 31396

MPLATPPFSVLSDEFAAAPEHFYARLRERTPVHYEPAIDSYFLSRHQDVK

RVLTDHDAFTTEMFQVRAEPVMRGRVLAQMTGAEHTAKRKIVVRGFTGSA

LQGQIRAIHTNAAELMAPFLPQGRVDLVNDFGKPFAVHAALDVLGLDRAD

WQQVAGWHAGVAEFVTSLALTPERRRHCLDCAERLDAYLAPVIRQRRSRP

GDDLISKLCTAEFDGVTMSDRDVTALIINVLIAAAEPADKTIALLFKHLI

DHPGQMAQVRRDPDLLGAAIAETLRFTPPVRLIPRQTQESAVFAGTTVPA

GATVFCMIGAANRDPAAFADADAFDIHRPDLGTARSFTAAAQHLAFGAGL

HQCVGAAFARAEIETVAALLLPLLDEVRYSPGFRYRETGLYTRGPVSLSL

DFTPVHASGTRRG

>CYP107L(2547447008)Streptomyces lysosuperificus ATCC 31396

MSLVDLTAHAEEFNADPYPFYEALRATGPVHRLVLGGERAWLVVGHQEAR

EALSHPALSKNWLGSELFEVAQVHAVATNMLDTDPPHHTRLRRLVAREFT

TRRVESLRPRVQQITDGLLDAMEALPGRRADLIRSFAVPLPMTLICE

>CYP107X(2547447027)Streptomyces lysosuperificus ATCC 31396

NLLNMDPPDHTRIRRLVSRAFTPRRTEQLRTPIRATADRLLDALGPAGTA

DLVAAYAAPLPIVVICDLLGVPEDRRLDFRTWTDALIAPDPSRPRAAKEA

VVAMLGFFTELMAHKRSRPGDDLLSDLIAVRDGGDSDSGSGSGSGSGGGG

GGGGGDRLSEDELMSLAFLILFAGYENTVQLIGNAIHTLLEHPEDLARLR

KDPTALPAAVEELARYEGPAPLAIRRFPTEDITIAGVTVPAGETVLLSLS

SANRDPARFPRPDLLDLDRDTSGHLALGHGIHYC

>CYP1147A3(2547447276)Streptomyces lysosuperificus ATCC 31396

MNRAEFHELRVLAASRPALPLLLHAGRMARPLKHIPRIGWVTADAATARS

VLTDPEHFTVLDRGGVGRLWAQLLGDWVDDLFDGEGHRALRTATRDLFGE

TTARALVDRVIGARLAETRIRLASGRPVDVAALGRTLVGRIVTELLRVPV

AGTDDAAYEKVFATARELAALSMEAAGGADLTDETVARGRRVVARLTGHV

EDEWRTGSAEHLVGRCREAGLDAHQTAGMAALVGVAATETAASAITRTVA

LLHDTGDQHRLLAAPELVPDAVREGLRVTAPAPVIGRTVRGDVRLAGRTL

RAGERITLLNYTANSLTGGFRLGRPPEPANRELWFGAGRHRCLGSAIAQA

EIGRTLETLAGVGRPWRIVERRYSRRVVIPTYERLTVTLV

>CYP180A(2547448016)Streptomyces lysosuperificus ATCC 31396

MDHAADVPDVFDPRLYAAGVPHERYRLLRDHHPVARQPEPEIAGWPAGPG

FWAVTRHADVVRVLRDHRTYSSWLGATQIRDPDPADLPFLRRTMLNQDPP

EHGRLRRLVARAFTPARVEAFAGRVRERARTLLAAARDAADDGAADLVRT

VTDEYALLNLTDLLGVPPADRALLLEWTVRVIGYQDPDDAPAPLLGPDGK

PLNPRSPALLGEMFAYARDLAAHKRRHPGDDVMTALATAGLDPAELEMFF

FLLTVAGNDTVRSAAPGGLLALAHAPDEYRRLAQGKVRPESAVEELLRVH

PPVLSFRRTAAADHEAAGQPVRAGDKVVVFHASANHDERVFTDPGRLDLG

RTPNPHVSFGDGPHVCLGAHFARLQLRTLYEEWCAAMPVPELAGTPRRLV

SNFINGITRLPLRVSGPPG

>CYP157C(2547448114)Streptomyces lysosuperificus ATCC 31396

MREGRVPADTPLGPMVSWVPVCNFTDGPVHERLRSAVVESLERFDKRGIR

RYVTRFANQLVDQIAAEGYADLVPAFSDQLPMLVMTQLIGAPDEHGPLLV

NAARDMLQGTETALQSDRYVTTTLENLVVERKAEPARDLASWLIEHPADL

TDTEVLMHLRVVLIAAYETTANLIANTLRTVLTLSLIHI

>CYP125A(2547449691)Streptomyces lysosuperificus ATCC 31396

MSCPALPDGFDATDPDLLQSRVPYPEFAQLRQTAPVWWCPQRRGVTGFDD

EGYWAVTRHADVKYVSTHPELFSSTTNTAIIRFNEHIQRDAIDAQRLIML

NMDPPEHSRVRQIVQRGFTPRAIRGLEEALRDRARKIVEEASAAAADGSF

DFVTHVACELPLQAIAELIGVPQKDRSKIFDWSNKMIAYDDPEYAITAEV

GSNAAMELIGYAMNLSAQRKECPAQDIVTQLVAAEGQGNLGSDEFGFFVL

LLAVAGNETTRNAISHGMHAFLTHPEQWELYKATRPSTAAEEIVRWATPV

VSFQRTATQDTELGGQKIKAGDRVGMFYSSANHDPEVFENPDVFDITRDP

NPHLGFGGGGPHFCLGKSLAVMEIDLIFNALADALPDLRPAGEGPRRLRA

AWLNGIKELRVSHG

>CYP163J4(2547449879)Streptomyces lysosuperificus ATCC 31396

MLTTATATAVNASTATATVAPGAVDLTDPDLWARPDTPALIAELRREAPV

HRTETADDGPVWSVLTYRESADVLRNAAVFSSESGSLLGSGEGNVPVGSG

RMMALTDPPRHRELRAPANPFFSKGGVRGAARSITERAGELFDRAVEQGE

VDLVDVVSALPLAVMCDLLDVPEKDRDMVVRVCDVAFLGRTPEERRAGHQ

KLIPYLMHQVMLRRSDPRDDLISMMATYKVGGRLLPIEDVVLNLDNIVVG

GVQTVRHTAAMGLHTLVQRPDLWRRLQRGEVSMDSAVDELLRWTSVGLHT

LRTATRDIELGGRRIRRGDRVAVWVWSADRDPEAFEQPEEIRLDRSPNKH

LSLGLGAHYCIGAPLAKAELSALYTAALEKAAAIEPTGPVTYNRSIINFG

LDHFPVRLTPR

>CYP107P(2547450311)Streptomyces lysosuperificus ATCC 31396

MGFDPWDAAFVADPYPAYRELRERGRAIWWEATGQWLVPHYADVSALLRD

RRLGRTYLHRFSHEEFGREAPPPEHEPFHVLNGNGLLDLEDPAHARVRRL

VAKAFTPRTVERLVPAVQRMAGELVGRLLADGGGDLLATVAEPLPVAVIA

ELLGVPESDRGLLRPWSADICGMFELRPDEETARRAVRASLDFSAYLREL

IAERRARPGEDLISGLIAAHDDEGRLSEQEMISTCVLLLNAGHEATVNTT

VNGWWALFRNPDQLAALRAGRDPEKLSTTVDELMRYDTPLQMFERWVLDD

IRVGDTDIPRGAEVALLFGSANRDPARFADPDTLDLERADNPHLTFGAGI

HYCLGAPLARRELEASFGALLADGVPPLRLVEEPQWQDGYVIRGLKSLLV

EF

>CYP107AH(2547450480)Streptomyces lysosuperificus ATCC 31396

VCRLTPPHGVDTYLITRHDDARDALSDPRFSKDMRGAIDTYHAVYGSFFD

ALDDNVLFSDPPRHTRLRRILRSAFTPRRVEQMRPRITEIAETILGECRR

AGTVDLMASFAFPLPVAVLCELMGIPQEDRPEILEQFAVVTRSRFDPSRR

AELKAAEERLQHRLERLISDTREHPSDTFLSDLLQAEERLEDPELVASLW

VLFFAGHKTTAYQIGNSVLNLLLHPDQAARLRENPRLVPGAVEEMLRFEG

SVETSTFRYAAEEARIRDTVIPKGSLVQIAISSANRDPERFDAPDELDVT

RKGLQGTHLGFGHGTHYCLGAPLARLELEIALTCLLREFPDMEPADLEET

RGAWLKGPVPAFRGLEHLRIVLEPSRRAGDPAGYPDGDPAGYPDGDPGEP

TLASVASGK

>CYP107U(2547450611)Streptomyces lysosuperificus ATCC 31396

MHDQSSTPSVGAAPELFTWEFATDPYPAYAWLRENAPVHRTKLPSGVDAW

LVTRYADARQALADQRLSKNPAHHAEPAHAKGKTGIPGERKAELMTHLLN

IDPPDHTRLRRLVSKAFTPRRVAEFTPRVQALTDHLIDGFAERGEADLIH

EFAFPLPIYAICEMLGVPREDQDDFRDWAGMMIRHGGGPRGGVARSVKKM

RTYLGELIHRKRDDLGDDLISDLIRASDHGDHLTEGEATAMAFILLFAGF

ETTVNLIGNGIHSLFMNPVQRERLQSSLAAGESGLLATGVEELLRYDGPV

ELATWRFATEPLTLGGERIETGDPVLVVLAAADRDPARFADPDTLDLSRT

DNQHLGYGHGIHYCLGAPLARLEGQTALATLLTRLPDLELAVPPQDLRWR

GGLIMRGLRTLPVRFTAQNTCATSD

>CYP179A(2547450708)Streptomyces lysosuperificus ATCC 31396

EDFSSANALLADVPLSEAALGVLPRGFGPRPTVVSSDGAAHRRHRAPLNR

GLSAARVAALVPHVRTYAAELVDGFAADGSADLVEAYAQRLPGMVVGRLI

GLDPADVPAAVHGGYRAEELLFRPLPPEGQAAAAEDVVALQHLLDGYVHD

RRAHPRDDLCSVMVAALAPGTGELTLEQRHELVTSLQNLLIAGFLTTGAL

IGTTLLHLLDDDRRQWRMLCADPSLIPAAVEEAARHDTAIQAFRRTTTRP

VTLGGTELPAGAAVLVAYGAANRDEERYERAGEFDITRPVNRQHLAFGHG

PHGCPGSRLAREQLRLTLELLTSRLPGLRPAPGRPRPRMRPTLIHRSPEA

LHVTW

>CYP154D(2547451114)Streptomyces lysosuperificus ATCC 31396

LSSHTAPVPQTPSGPDTPYVIDPAGGCPHALNARLRTEHGAVARVVLPGG

VPGAVVLGHDALKEFLAHPDVAKDARHFPALHDGSIPRDWPLRAFANAQG

MHTADEDDHRRLRALVGRAFTARQVERLRPRVEELTAELLDDLDRAAAAS

PDGVADLREHFALPLPMSVICELLGVNPEHRGRLHHLSNKVIIATDTTPA

EAMAAVRELVELMGTIAATRRAEPGEDLTSALIAARDEDGDRLSEAELIG

SMRLMLIAGHETTLNLVSNAVRALCTHRDQLSLVLEGKATWSDVVDETLR

WDGPVSWFPFRYPTRDLTVGGTVLPQGTPVLAGYTAAARDEDFHGPDADR

FDITRPTAARSLSFGHGAHYCIGAPLARLEATTALERLFTRFPDLDLARP

GAELPHQRSFIGNSVETLPVRLHRR

>CYP147F(2547451484)Streptomyces lysosuperificus ATCC 31396

MGKAVLRQIVDYSSRADPYPLYEELRRTPVFHDEDGPYLVSTYHEIHALL

HDPRISSDPRNLTLSSSDPLTQDEGDDSALPPVFLKLDPPDHDRLRRITN

RPFGPPHSPHKVHDMRGELGGIVAGLIDGIAASGNLDRVDLVEQFSYPFP

VTVICRLLGIPHEDESRFHGWADTIAASLDPAPDADPAERKKDASAAQME

LGMYLGGLIEERRSNPAEDMLSQLAAVDEEPDGSLSTLEIVSTAALLLIA

GHETTVNLI

>CYP1047A(2547451549)Streptomyces lysosuperificus ATCC 31396

VTATLHPGTRGGGPRRWPLLGNLPAFARDPLAFFESLRDDHGDWVPWSLG

PKRNVLVSLPEHAGELLGAVESTFRPTELGWAFHQLLGDGVVVATGDDWR

RKRALVQPAVRPRQVRSYAATMVECADALAGGWREGTRIDVHREMAGLTQ

RIAVRTLFGSDAAGREAPISAAMATAQRELGAEFRGLTLFLPPWVRTPGR

RRMREAVAVLDREIEHVIREHEAASAAGAERDDLLSRLLAARDEHGGPLS

RKELRDESITLYIGGHETTSTTLTWAWQLLSGAPEARARLTEELDRVLGG

RLPEYDDYARLPWTRQVVKETLRIYPPIWLISAVATEGATLGGRPVPAGT

SVWTSPWSVHRDRRWFPEPEAFRPERWDDDAPHPVPEHAWFPFGGGPRAC

LGARFALVEAALVLAVLGQRFHLDSGSERAGVFPGLTLQPDRPVLATLRR

PAGAPEA

>CYP1419A(2547451621)Streptomyces lysosuperificus ATCC 31396

MALPNRPSPRNPVSHARARRRDRRVYLGGHPVLFGLLAATRGRPVRRIGG

TLLVHGADAYRQALTRLPLDRTAAGTTGGAARSALAGDGGVLFDQEGGAH

RADRRDLAGGLGAAGVEELRARWRPLLARRLAPLAAGGEVDLVVLARELS

GTVVCALLGSGADPCEVARAAAEAAAASVRSHLPGPRRPRAEAAAARTAG

RLRGLLAPAEGAPDGGPGCEVPAADGALAAMVAVAAVNTTVAALPRAVAW

CADAGLWDQACDAALRPALAAELLRVTAASPLLPRVAAADGTVGGCPVRA

GDRLL

>CYP162B(2547451754)Streptomyces lysosuperificus ATCC 31396

SDTFLSARGMRLGSDPAAVAAVAQRMLIVSDVPHHTRLKRALGQAFGPQQ

MPRLEALVEQVVADLVAEAAERDELDFIDLAKQLPNRVVCAILGIPRADW

AWIGALTTDAFDSPDEAVRSSAHSEIFLYFIELLAERRARPGDDLVSQIA

HDTLVDEGDGSERPLSDHEIVFNLNGVLSGANETTRYSAAGAVHMFAEHP

DQWRLLRALGQDGIAPAVEEILRWTTPGVHALRTASRDTEINGTPVAAGA

RVTLWNVSANRDEDVFADPHSFRVDRRPNRHVAFGHGRHLCLGARLARFE

LGALLTEMLAVLDGFELTGPATFNSSNFTWGINSLPVRLLRSRAFAK

>CYP1038A(2547452327)Streptomyces lysosuperificus ATCC 31396

AQGAVAAELARIDALSSRAIAAARTAGAGPHGPGLLHVLLEAAETSPEYT

DRLIRDELVTLLVAGHETTATTLTWLHLLLDRNPQARERALAAGAEGSPQ

RHRAVQALVHETLRLYPSAWILPRHAAGDDVLAGYAIEAGTDVLVCPYLT

HRDPGTWEDPGRFDPDRFLTPGRRPTRPGSYAPFGLGPRACLGLQFALRE

STVLLEHLLPAHVPHFHSVPAKASYGLTVRPDGPTPATLTAAATA

>CYP184A(2547453590)Streptomyces lysosuperificus ATCC 31396

MAFGRNAGGVDAGTEVPVLPGAPLLGSLHDLKSDSLGTYLRAQQRRGDVV

RVSAGPPGMRADLYCVFSPEGVQQVLASQAANFRKDNSFYQEVRESFGNG

LLTSQDEDYLRQRRLVQPLFTRRRVDGYAGAVAAETASVVAAWEEAVDGV

VDVSDEMTHLALRAVARILFGTDVEATADVVARCFPVITDYVLRRGYSPA

NIPRHWPTPGNRRAAAAMDELYGICDQIIEQRRRAGANAPGEDASPGGTS

AGGAPGEDASAEGASAEQASGEDLLTLLAAARSSDDGEFDAAELRDQVLI

FLLAGHETTATSLCFALHLLSRHPEEQDRAREEITRVLGDRTPQASDLDR

LPYLSQVVKEAMRLYPAAPVIGRKAVAATRIGEHAIPAGADVILAPWVTH

RHPEHWPDPDRFDPERFTPEAEAGRPRYAWYPFGGGPRACIGQHFSMLES

VVALAMILRAYSFEAVDTEIPVAAGITLRTEGPARCRVRRLAR

>CYP125L2(2547453714)Streptomyces lysosuperificus ATCC 31396

VGVPSADERLVFDWSNRMAGGQDPELSAGKQDSDLAAAEIYAYCDALAGE

RRARPREDILSTLVHAEVDGDRLSQHEVNMFFVLLCVAGNETTRNLLSHT

LLALVEHPAARAELAAAPHDELLWNSATEEFLRWGGSIHNFRRTATRDTV

LRGRYIAAGQKVVTYYTSANRDEEVFGDPFTFDIRRTPNEHLAFGGGGPH

FCLGAGLARTQIKALIRELVRRHPDFELAGEVRRLRSDFINGIKYLPVRF

A

>CYP107L(2547454053)Streptomyces lysosuperificus ATCC 31396

TLICELLGVPNLDRARFRYWSGEIVAPLDGVGADPRVLEEMTAYLSELVA

AKAQDPGDDLLSALIRTRDEDGDQLSPDELIGMAFLLLVAGHETTVNLIG

NGVRALLDHPGQLAALRADPEGLIDGAVEEMLRYDGPVQHATYRFADTDL

ELGGVPITTGSSVMVALAAADRDPARFTGPGPGPEVFDIRRTGPGHLAFG

HGIHYCLGAPLARAETEIALAALLERYPVLEPAGP

>CYP121A(2547454897)Streptomyces lysosuperificus ATCC 31396

MPTDTLLDFPFSARGDQLPPEVEQLRDEPVKRVRTIAGDEAWLVSSYSLC

KQVLEDPRFSLKDTSAPGVPRQYALTIPPEVVNNMGNITGAGLRKAVLKA

INPKTGNLTGWMRDQANTLVDEILRSGAPVDLRGAFTNPYAENLHCRILG

IPEADAPQLAASLDIAFMNSACPVTGAKLNWDRDMAYMVERLDDPATVGL

MAELAALREDPDYAHLTDEMLAVVGVTLFGAGVISTMGFLTMAIVSLLQN

PDVWEQLRKAPEKIPAAVDELLRVNLSIADGLPRLALEDVTLGDVEVKKG

ELLLVLVEAANTDPAVYSDPHVFDIDRANAGTHLSFGGGPHYCPATALGK

RHTEIAIEVLLEKMPNLQLAVPFDQLVWRTRFMKRLPERLPVLW

>CYP105AK(2597940791)Streptomyces sp. PVA 94-07

MPIAAPIRTVRTARSSIASWLTRRYLSRLRRKGTTLDLNALSKLPEPALL

PLRRNGLDPVPEIGALRDREPVSRLPVPAVPVWLVTGYDEAKEVLGDARA

FSNDFAHLVGTNGVAEHHEPGGLGFADPPDHTRLRRLLTPEFTMRRLNRL

TPLIHSIIEERLDALEAAADADGRVDLVEHFALPVPALVICELLGVPYEE

RDAFQQFSVARFDVLGGLGASFGAISQSREYLRGVIEQQRREPGDGLLGM

IIREHGDAVTDEELTGLADGVLTGGLETTASMLALGTLVMLQDRTHFTAI

READDPGAVATPFVDELLRHLTVVQTAFPRFARENTVVGGQAISAGDIVI

VSLSAADRDKRLGPEMDAFDPSRPPASSHLAFGYGIHRCVGAELGRMELR

AAFPLLVERFPALRLAVEPQELEFRKLSIVYGVDSLPVRLK

>CYP1047A(2597942670)Streptomyces sp. PVA 94-07

VLRGACAGEVAAGANRTSCFRFRSTPLLDAFVVDLSVVGRGRMRREPPVS

GRRAAGRGVREMSADTGARGQDRQGEVPVVVPGPKGVPLLGSLPEFGKDP

LAFFERLRDQGSVVSWNFGGKPSLFIADPDLVGELLREVESTFDQPDLGV

AFRAVLGDGVTVARGRDWRRKRSLVQPSVRPKQVKSYAATMASCAVDTAD

GWRDGQRIDIKREMAALTQRIAVRTIFGTDAEGDVEAIGRAMDIAQREIG

AEFSGIGAVLPDWVPTPGRRRVKKAAAVIDREVGRVVAAHREDGERPDLL

SRLLTAQDETGARLSEEEIRDETVTFYIGGHETTSSTLVWAWYLLARNSR

VRAALDEELDRVLGDREPGFDDFARLPYAQAVVQETLRLYPILWLLTGIA

KEGASLGGLPVAPGTRVWTSQWAVHRDPRWYGDAEVFRPERWLEGAEESI

PEYAWFPFGGGPRVCIGARFATVEAVLILAVLGRRYDLDIDPGEIRPMTT

LTLQPDRDMLATVRARGGGA

>CYP157C(2597942675)Streptomyces sp. PVA 94-07

VTTPSTPPSFPGTPAGPPPGCPAHGADPVGPFGAGGLRRLYGPEAERDAP

GLFEKLRAEHGPVAPALLHDDVPIWVVLGHSENLHMLRTHSVYTRNSRRW

RLVQDGTLGPDYPLTPLFAWQPICSFAEGAERERLRGAVNNAMKQIDYRG

VRRAINRHSNRLVNEFGQDGRADLVSQFTDHLPMLVMLDVLGLPEEYNEQ

MVDAARDMLQGTETANASNAAIMGILERHVARRRAQPDDDFTSSLLEDEA

RLTDDEVAQHLRLVLIAAYEATSNLTANVLRMVLTDPRFRAQLNGGQMTV

PEAVEQTLWDQPPFSNMLGYFAVQDAELGGQQIRKGDALLLNIAAANVDP

VVRPDLEANMQGNRAHLAFGGGVYECPGDDLGRAIADTGVDALLMRLPDV

ELAVPETDLHWTNSLISSHLKELPVVFGPRQPLEISSSPGQAGASRTDWE

ISSPAPRPPAAGPVPPQAAPHVPAPPAADGSVATVPAQRRGAWRRLVEWV

RGG

>CYP107R1(2597943009)Streptomyces sp. PVA 94-07

VTTPSQQLRDFPFAPPAELHMEPAFAQLREEEPISRVRLPYGGEAWLVTR

YRDIKTVLGDPRFSRAATQHAQAPRIQPDPAGEGVLMSLDPPDHTRLRKT

VAGVFTKRRVEQLRPATQMIAEELLEAMEASGAPADLVASYALPLPVTVI

CDLLGVPRDDRDQLRGWSDALLSTTACTPAESAAAAQAMADYFAALVSQR

RRQPTDDLLGALVQTWDREEGLLRDEELVLLTRDLLIAGHETTASQIANC

TYLLLQRPHDMDRLRTDPSAMASAVEELLRFIPLGSGSFRARVATEPVEL

CGVRIQPGETVFAPTVAANWDPDVFTDPGRLDIDRSLNSHVAFGHGVHHC

LGAQLARLELQVALGVLLRRLPRLRLAVDEAEIAWKTGMQVRGPKTLSVK

W

>CYP107F(2597943292)Streptomyces sp. PVA 94-07

VSTEAVTPVPESAEVPTCPFDFAQGLDFDPALLAMLRSEERVARVRMPYG

EGDAWLVTRYEDVRTVTTDRRFSRNAVTGKDFPRMTPEPIVQTGAINLMD

PPESSRLRRLVRQSFAPRHLERMRGRTQSVVDGLLDAMAESGSPTDLFAH

LALPLPLVTICEVLDIPEADRHWLRAHAMTMMNMKPAGKEAAVRAKGELR

EYFARLTAERRRSPGADMISTLATAREGGETLGEDELTVMAMVLLITGQD

TTTYQLGNISYLLLTRDDVRERLRGDPDSLPRVLEELLRHIPFRKGVGIP

RIATEDVELGGALIRAGDTVHVSYLTANRDGEKFERPDEIDLDRPSVPHM

TFGWGSHHCLGSPLAVMELEIALSTLLRRFPDLALAVEPGEVEWNATSIW

RYPLALPVTW

>CYP105H(2597943348)Streptomyces sp. PVA 94-07

MTTSPGPTVVDFPRRTPREPLPLSQYAEHRKQNGLVQTHLPNGRPIWLVT

RHEDVRAVLTHPRISANPDNEGFPNVGETMGVPKQEQIPGWFVGLDSPEH

DRFRKVLIPEFTVRRVRELRPAIERTVDERIDAMLAGGNTADLVNDFALP

VPSLVISALLGVPSADRDFFESRTRTLVAIRTSTDEERAEATRQLLRYIN

RLIVIKKKWRGEDLISRLLSTGKLSDEELSGVLLLLLIAGHETTANNIGL

GVVTLLSHREWIGDDRLVEELLRLHSVADMVALRVAVDDVEIAGQTIRKG

EGIVPLLASANHDTEAFGCPHAFNPERTERRHVAFGYGVHQCLGQNLVRV

EMEIAYRKLFERIPELRLAVPEDQLAYKYDGILFGLHELPVRW

>CYP105BT(2597943496)Streptomyces sp. PVA 94-07

MANPTYPMTRQCPMAPPAQYNDLRGRGPTKVDMPDGGWAWLLTAYDDVRQ

AMNDPRFSSDDEKMGRARTELPTGHDLNSFWRKDEPEHGRLRHMMMTEFT

AHKIKSWRPRIQALVDELLDRLEELPRPLDLYSEFCLALPTQVIAQLLGV

PQEDYRTFAKQSREVLSLDNPEVSWRAFGEMNDYLNQLIEEKEREPKDDL

ISRLIVDRVHTGELDREDLLPMVRFILVSGYETTTSQIALSALTLMTNPD

VRARLIEEPERITAFVEESLRFWSVSQDNVLRVVDQDMEFSGARMSVGEL

VILAVPSANHDERAFPDPENFDLDRGENRHVAFGFGTHLCAGASLARREV

EIAITSLLARYPEVRLVGEVDDLTFRQQSLVYGLEHLPVTW

>CYP105BT(2597943569)Streptomyces sp. PVA 94-07

MANPTYPMTRQCPMAPPAQYNDLRGRGPTKVDMPDGGWAWLLTAYDDVRQ

AMNDPRFSSDDEKMGRARTELPTGHDLNSFWRKDEPEHGRLRHMMMTEFT

AHKIKSWRPRIQALVDELLDRLEELPRPLDLYSEFCLALPTQVIAQLLGV

PQEDYRTFAKQSREVLSLDNPEVSWRAFGEMNDYLNQLIEEKEREPKDDL

ISRLIVDRVHTGELDREDLLPMVRFILVSGYETTTSQIALSALTLMTNPD

VRARLIEEPERITAFVEESLRFWSVSQDNVLRVVDQDMEFSGARMSVGEL

VILAVPSANHDERAFPDPENFDLDRGENRHVAFGFGTHLCAGASLARREV

EIAITSLLARYPEVRLVGEVDDLTFRQQSLVYGLEHLPVTW

>CYP105AC(2597943881)Streptomyces sp. PVA 94-07

MTVGDPLYTVTTMPTQRPPERPFDPPAEMLEARRHGPISRYVFPGGVDGW

LITGYDLVRSVLADPRFSSRWELLRHPTVDHGGVEPDPAPPGEFLFMDEP

RHGRYRKPLMGKFTVRRMRLLTERIEEIAAEHLDAMELAGPPTDLVTAYA

KPLPAVTICELLGVPHDERPSFQEQVDAFRDLESDRDDLIAAYHATRAHL

AALVAAKRARPTDDVLSELTDGDLTDEELVGMSLFLLAAGLDTTMHMIGL

GTYALLRHPGQLAALRADPALADRAVEELLRYLSVGKTFLRTALEDVEVG

GHTITAGTTVILSYNTANRDPARFTDPHRLDLHRREGAHIAFGHGAHQCL

GQQLARVQMRVAFPALVNRFPTLRLAVPAEEIALRPEAEIADVYGVKSLP

VTWDA

>CYP105AC(2597943889)Streptomyces sp. PVA 94-07

MGDPLHTVSTLPAARRPGRPFDPPAELLEAQDHGPLSSYTFPGGRVGYLV

TGYDLIRSVLSDPRFSSRRELMRHPTVDYGGLELPPVPPGEFLLMDDPQH

SRYRKPLIGKFTVRRMRLLTERVEEITAEHLEAMRESGPPADLVSAFAKP

VPARVICELLGVPSAARASFQAQFESFMDAEARDEDTVVAYTTTRERLGE

LVAAKRAHPTDDVLSELTDSDLTDEELLGMALVLLAAGLDTTANMLALGT

FALLRHPEQLAALRDDPGLTDRAVEELLRYLSVGKTFMRTALEDVRLGGR

TVEAGSSLILSYNTANRDPSRFTDPHTLDFQRQEGGHVAFGHGIHQCLGQ

QLARVEMRVGFPALLNRFPSLRLAVPAEEIALRTELSDIHGVKSLPVTWD

A

>CYP107BX(2597943962)Streptomyces sp. PVA 94-07

LSAASPAPAGAVPPFAPLHHRAPAEPGPPRPCTLPDGSPGWLVDRYADVR

QVLSDSRFGRAGLYLQDGPSRSQAAGLVDDPELMFNQDGVEHLRLRRTLR

RAFTPRAVARWRPWIASIVDQMLDDLAARSGPVDAVAEFTLPLPVAVISR

LMGLDASVRGRMRHWSEHAFSDGTRPKDEVDAALAEFTSFGARLLAQRRR

APGDDLVSSLVRAADAEGGIPEDRLVSLVCGLVAGGHDSTMTMLGNSLLY

LLGERPEVWPRLADEASAELAAARLIHLIPLGDDPGSTRCATEDAEVGGV

LIPAGAVVLADSTTANRDPSVFPAAQTEALFTPLPAPTLAFGAGPHYCLG

AWLARLELHLALHRLAVRFPGLRLAEPEKPVRWRPAGTSRSPERLPVTW

>CYP107L(2597944336)Streptomyces sp. PVA 94-07

MTDVVDLTAYGERFTTDPYPVYAELRARGPVHRVSLPGVDGPAEAWLVVG

YEEAREALADARLSKDPTTLGIQIPEGELIGRHMLVADPPEHTRLRRLVA

REFTARRVQALAPLVQRITDELLDAMVPLGRADLVQSFAFPLPLTVISEL

LGVPVADRAAFRRMSSEVVAPSGKVPPEANLAELGTFLDALIEEKRRSGT

TGDLIGDLIRTADDEGDQLSSSELRAMAYLLLIAGHETTVNLIASGVHTL

LRHPDQLAALRADPGLTEGAVEEILRYEGPIETATWRHTGEPVEIGGVRM

GKGEPVLVSLASAGHDPARFPDPERFDIRRRTQGHLAFGHGLHFCLGAPL

ARLEGRIALTSLLARCPELTADGEPSGWAPGLLIRGVRDLPLRW

>CYP154T(2597944529)Streptomyces sp. PVA 94-07

VTTTPDAAVPEITLDPYGSDHHGEAARLRALGPVVRVRLPGDVRAWAVTR

HDLLAELVADPRMSKDWRNWNAIRRGEIEDGWPLIGMVKVTNMVTADGQE

HRRLRKLVMQTFTPRRVAELRPRVEAIVAELLDELPSHADADGVVDLRRH

YANPVPMRVICELFGVPGHEQPRLKELMDNIFRSDLGPEEVTASQVEQYQ

LLARVVAARRAEPGPDLTSALIAAREADPDALSEEELVGTLLLMLSAGQE

TTLSLVTNAVRALLTHPDQRALAEAGGEEVWEAVVEETLRWDAPIGNFPF

RYPLEDVEIAGVPIARGEAIMAPYSAVGRDQAQHGEDADRFDLTREQNRH

LAFSHGPHFCLGAPLARLEAALAIPAVFARYPGLSLAVDPATLTPVPSMF

SNSSATLPVRLAHDT

>CYP157J(2597944530)Streptomyces sp. PVA 94-07

MTAAPPPGCPAHASTGLVPLTAATGAPDHREVYRRLRAEWGDVARVELEP

GVAAWLVMGYREMLTLTRNEQLFSRDARNWRDLREGVVSPHSGLLPMMGW

RANVIGADGPEHRRLRAPLDDGVARIDQRRARRQVEALCEELIAEFAARG

SADLVGEYATIVPMLALASLFGLDSAGGHELLRALIALFGSADDSQAGNR

QFEKILLDTLRERRARPADDLTTAFLDHPDLHNEAEHLQSVVVMISAGNE

TVTAWIAHTLRLLLCDPRFASRLHGGRLGIDDALDEALWRDPPMNNMPAR

YALRDTELGGHRIRQGDCLILGIAAANDDPLIRPEDELTEPGNRAHLAFS

AGPHVCPAQVPARLITRTAVKTVLHRLPGLRLTVPAEEIGWRPSPWTRVP

TALPVAFPPAPRPSKENR

>CYP170B(2597945316)Streptomyces sp. PVA 94-07

VSAESTTERAAAPDPRTPPLVAGSVPVLGHAPSLVRDPLAFLTGLRDHGD

LVRVKLGPKTAYAVCAPGLVGALLRNSQEFQVGGPLWENLEVLLGKGVAT

SNGADHRRQRRMMQPAFRPERIASYATVMEQEARAMADRWRDGQVVDIGA

ETFSCAVRVVARSLLEVDSIGAKADRISASLHTVFSGLYRRMILSFGPLH

RLPTPANRRFERALADLHHLVDEVIAERHAQGKGADDLLDILLTSEDGEG

RPLSDQEIHDHLVSLIVAGAENVASTLAWCFQLLTEHPAEEQRVAEEALS

VATGRPVNFGDLGSLDHTRNVIVESMRIRPAAWIFTRRAVTDTELGGYRI

PAGADIIYSAYAMQHDPRSFDRPDVFDPDRWIPERAEKVPQYAMMPFSTG

NRKCPGDHFSMVEATLMLATVLPRWRLVPVPETDPAPRIGITLQPKRAVF

RVESR

>CYP107U(2597945714)Streptomyces sp. PVA 94-07

MSETQAAGRPELFGWEFAADPYPAYAWLRTHEPVHRTRLPSGVEAWLVTR

YADARQTLADPRLSKNPVHHAADEAGRSRTGIPGERSAGLMTHLLNIDPP

DHTRLRRLVSKAFTPRRVAQFAPRVQELTDGLIDGFAGRGEADLIHEFAF

PLPIYAICDLLGVPREDQDDFRDWAGMMIRHGGGPRGGVARSVKKMRNYL

AELIHRKRADLGDDLISGLIRASDHGEHLTEEEAAAMCFVLLFAGFETTV

NLIGNGTYALLRNPAERARLQRALADGDDALLDTGIEELLRYDGPVEMAT

WRYATEPLRIGGADIAAGDPVLVVLAAADRDPERFAGPDRLDLARTDNQH

LGYGHGIHYCIGAPLARLEGKAALATLLTRLPDLRLGAEPEELRWRGGLI

MRGLRTLPVEFTPEQANGAKKV

>CYP157A(2597945972)Streptomyces sp. PVA 94-07

VTPPDTPSATTDAPGATTPAAPTTGGCPVAHGAGGAEPAPLLLGGDRFQS

DPIGLYRDLRRDHGPVAPVVLPGKLPAWLVIGYRELHQVTSDPVLYSRDS

DLWNQWDRVPENWPLLPMIGKQESILYTVGERHRRRAAVMEGGLEAVEAH

ELRATTERLADSLIDDFCGSGEADVIADYAMMLPALVLFQLFGLPESEGR

GLAHAINDMINGGERALAGQQHIRECVGRMVAGLHVHPRDHVTSRMLRLG

GHLPGEDRAVITDEFSATEIIQDVLVMVVAGHQPTADWIGNTLRLMLTDT

RFAASLFGGRHSIAEAMNEVLWEDTPSQNIAGRWATRDTHIGGRRIREGD

LLVLSFAGANYDPLVRTDQSALTGGNNAFFSFGHGEHRCPFPAREIAEIV

ARTAIEVILDRLPDIDLAVAAEELTRRPSPWLRGLTRLPVSFTPVPAG

> CYP154C(2597945973)Streptomyces sp. PVA 94-07

MSLSETSATPTPATPPPGCPAHAHGAVGPEAYIALDPLVRDLDGESARLR

AAGPLAKVMLIGDVPVYSVTHHAEARKLLTDSRLVKDINHWNAWNNGEIP

ADWPLMGLANPGRSMLTADGPEHRRLRNFVAQALTVRRVQKLRPGIEALC

ARMLDTMEQAADERGVVDLKAHYAHPVPMTVITELFGMPSHHIPRLKELF

DIFFSTVVPREQVPPMMAELDDIFQAFVQSKRDEPGDDLTSGLLEAAADG

DTLTNEEIVNTLKIIVTAGHETTISLIVNAVRALSAHPEQRARVLAGEIP

WSQVIEETLRYNTPTSHLLIRFPTEDIEVGDQILPKGEGLIVSFSAIGRD

ENQHGPTAGEFDATRDPIRHIAFGHGPHVCPGASLSRVEAEVALPALYAR

FPELTLAVDDSELRNKPILTQNDLFDLPVRLHG

>CYP154A(2597946004)Streptomyces sp. PVA 94-07

MTSSAPPEPEPLVLDPRGADPHAEHRALHARGPATRVDVLGVPAWSVSDP

ALLKTLLTSPDVSKDGRAHWPAFAETVRSWPLALWVAVRNMFTSYGADHR

RLRRIIAPAFSARRVAALRPVVERITGGLLDELAALPPDAPVDLREKLAY

PLPVAVIGHLMGVPEERYARFRAVVDGVFDTTLGREEAMANTGALYVVLD

ELIATRGAEPGDDLTSLLLATPDEEGAGPALSREELRDTLLLVISAGYET

TVNVIDQAVYALLSVDGQLELVRSGRVGWADVVEETLRHEGAVKHLPLRY

AVRDIPLPDGRTIARGEAILASYAAANRHPGWHDDADVFDAGRLSKEHLA

FGHGVHFCVGAPLARLEVEVCLRLLFARFPGLALDLPAGGLPPFPSLISN

GHRSLPVWLHGAGPG

>CYP156B(2597946026)Streptomyces sp. PVA 94-07

MESHPPYASATGRCPMHDANFAADPQAVYEQLREQGPAGPVELAPGVDAT

LVVGYETALRVLQNPTSFPRDARRWAALNEGRVPMDSPVLPMMAYRPNAL

FTDGAVHLRLRKAVTDSLAKLNITRIRRDVEPIADYLIDQFSERGRADLL

AEYAKLLPLLLFNKLFGCPAEIGDTLTTEMSAMFDGKDPVRTKARLDACL

MQLIAIKRREPADDVISWLIQHPAGLTDEELKDQLVMLMGAGIEPERNLI

GNALLELLSGGAGGRGAGMMVEEAVDHVLWHRTPIANYAAHYPAQDTDFG

NGVIAPAGSPVLISFAGANSDPALAEARRSAGRGAHLAWGAGSHACPAKD

PAQVIAVTAVEKILNALPDLTLGVPEEELQWRPGPFHRALVSLPVAFSPT

PATRMAAALQSRGVPQQQVAPATPQPQHTAPASSDGGGRRKGFWSSFLDI

FRV

>CYP105DY3(645969743)Streptomyces sp. SPB78

MTIWDGSTPWLITRHDHIQQLLGAVGMISADVTDPGFPNTSDAQPAVEGN

IFFRKDGTEHLPVRRILNPDFTAKRSEALRPRIAELTDRLLDDLLAMDGP

VDLFEHFALALPTVVICEVLGVPYEDSTTVHESSKKMVQLAIAQDEKVAA

HRALHELLARHMEAKRSRPDNGLLSRLVNTHVDDKGDLTFEEAVGLGVLV

IGAGHETTANMIALGILALLREPSQRDLLLSDPVAYSSTAVEEMMRYWSM

VQTEPRRVLTEDIEVGGVLLKAGEGIICNLPAANRDPLVFADPERLDITR

RERKHIGFGYGVHQCLGQNLSRVEMQVAWPKLFERIPGLRLAVDESELHF

HDDALTYGVETLPVTW

>CYP105AG2(645969744)Streptomyces sp. SPB78

MTDIPVNQPVTRVTGAPLDPPAEYTRLREDQPIVKARFPNGSTGWLVTRF

EEGSQVFTDPRLSARRPRHDTPEGEIAEAGEDAPFDAGFVMMDEPEHGAY

RRLLTARFTPKSVQNKLQPYLDKIVDEHLDAIAAGPETFDFVQAMALPIP

CLVICELLGVPYEDRDGFHDATVDLMDMAKSREERDRGAHWLIDYITSLV

ADKRRTGARDGILAELIHKSDGDDAALTEKQLIGLGVLLLFAGHDTTAAM

MGLSTLTLLTHHEQREQMLAHPEKTGTMVEELMRYLTIVQFGLGRVAKED

LELAGAQVKKGDLVVVAMNAANRDPRAFQDPDTLDIDRKMARHMGFGYGV

HACLGQNVARAELRTVLPKLFQRFPNLRLATPLEEVPMDFTGTNYGVRKL

MVTR

>CYP154T(645969773)Streptomyces sp. SPB78

MIADPRFSKDWRNWSAIIRGEIEDGWPLIGMVKVTNMVTSDGQEHRRLRK

LVTQTFTPRRVQEMRPRIEHIIDDLLDALPDRADADGSVDLRRHFAHPVP

MQVICELFGVPAHERRRLQELMDNIFRSDLPPEEVTASQIEQYQLLGRVV

EARRDDPGDDLTSALIAAREADPDALSEEELVGTLLLMLSAGQETTLSLI

TNAVRALLTHPDQRALATGGDASVWADVVEETLRWDAPIGNFPFRYPLED

VEIAGVPIPKGEAIMAPYSAVGRDQRQHGSDADRFDITRERHRHLAFSHG

PHFCLGAPLARLEAGLAIPAVFRRYPDLSLAVDPGALVPVPSLFSNSSST

LPVRLGN

>CYP157J(645969774)Streptomyces sp. SPB78

MPLSAAVGSGDPQAAYRRLRDEWGNVAKVELEPGVPAWLVLGYRELLTIT

RQEQLFSRDARNWRDLNDGLVPLHSGLLPMMAWRANVIGADGPEHRRLRM

PLDNAVARMDQRRVRREVEALCTELIAEFSPRGRADLVNEYATIVPMLSL

ASLFGLDTTEGRELLDALIALFGSADNSQSGNRNFEQIILDTVRERRRNP

TSDMTTAFVSDPNLHNEAEHLQSVVVMISAGNETVTAWISHTLRLMLTDP

RFAARLRGGRLGIDDALDEALWRDPPMNNMPARYALHDTELGGHVIQRGD

CLILGLCGANDDPAIRPGSDEESWGNRAHLAFSAGPHVCPAQVPARLITR

TAVQTALHLLPDMRLSIPAEEVTWRPSPWTRVPVSLPAEFSASRIATGIR

R

>CYP157W1(645970583)Streptomyces sp. SPB78

MLLPRDVPAWLVLGHEENRFVCAHPELFTTDGREWRLLQDGTVGPDHPMA

PIFMWQPVCAFVEGEEHERLREPLEENLRVTDLRELRRLVQTGTYTLLND

LCEEGFCDLTEDYCERLPMIVLLGLFGMSHLYDDRFLEAARDLIKGTETA

NASNEHLMRLFRAEVERARVEPGDDFASQLLAHRAGMNAREVSEHLRIVL

IAAYETTANLLANAMRILLVQMEVRGRVGAGRLNIYEAIEQALWDEPPFS

AMLGRYALQDVEVGGRLIRKGDAVMLGYAAGNVDTRVRPELDAPVRDNRS

HLAFGRGPHACPGQYLGRQLCQLALDDLLAWFPDMRLAVPSSQLRWSGSL

LSRHLVELPVRFEPRSQRDEESIGTFGVPLPKGAGIFPPPDEEHDLMPVE

VPDAVLEAEREAGEWRAAKKRREQAEQDGPAPVDAPAGDAGAEAEGLLPG

PRRPLRDWLRR

>CYP156B(645971250)Streptomyces sp. SPB78

MNAHRASAPTGGGCPLHDAAFAADPRQVYEKLRAEGAAGAVELAPGIEGT

LVVDHGTALQVLRNTELFPRDSRRWRDLVEGRIAADNPLLPMLGYRPNTM

FSDGSTHLRLRKAVTDSLAKLNMSRIRRDVEPIADHLLDQFSERGRADLL

SDYAKLLPLLLFNKLFGCPADIGDTLTSSMSAIFDGRDPLRANEELTACL

MELIALKRRRPGDDITSWLIQHPSGLTDAELNDQLVMMMGAGVEPERNLI

GNTLLLMLSSSGGHDSGPQIEEAMDHVLWNATPIANYAAHYPVQDVDFGG

HIAEANTPVLISFAGANGDPRLAEARGNQSKGAHLAWGAGPHACPAKDPA

QVIASAAIERLLNALPDLSLAVPEQELRWRPGPFHRALVSLPVNFSPSPA

NRMAAHRPQPAPGEDRQPHAAQRDASGTGGAGGTGSTREPARKKGFWSTF

LDVFRA

>CYP1035A(645971251)Streptomyces sp. SPB78

MNAVGHHPGTSTVSRRAVIPLLRRLRSAEGAANPRPVWAALHELGDVVPA

PWGGYFVVGFDACNEVLRGRKWQSTDFDWQQRQDNPERWSDLATREMTRT

LPRLNAPAHTCQRRAVGTPFDRASLEALRPMIAALISSLLDELEAEIRAE

GHADFMRVVGEQLPALSVGEWIGIDRADHAHLLDFTHRQVYAQELLPTRS

QLAVSEQATLEMREFFTRLIRERRARPGDDVLTQWIAHFDASSGGDRAAA

DQIIYDLTMFVTIASLETSAVLLGNTVHLATSDPARAAWLTAHPEHIAST

VEEALRWDAPISINSRVASEDLVLAGVPIPRDTTVHVMYGAAAHDPRRNP

DPDTFDPLRRGGHLSFGGGPHYCLGAGLARLEAQELLGQVLKRFPTLRAV

TPPVFDDTRLVFRRLVSLDVQI

>CYP105A(645971808)Streptomyces sp. SPB78

MTSPTALPPQPVARDRARPLDPPTAATELREERPVSRVRFPNGLSGWLVT

RFEEGCQVFSDPRMTALRPRHDTPEGEVAEAGHDAPFDAGFVMMDEPEHA

VYRRLLTARFTPKAVATRLQPYLDRIVDEHLDAIAAGPRTFDLVQALALP

IPCLVICELLGVPREDRDAFHEATVKLMDMGVPRAERDRGAHWLMDYIAK

LVAEKRASDAEDDGILAMLARRGPDAEPPLTDRQLVGMGALLLFAGHDTT

AAMLGLSSLTLLTHDEQRHDLVTHPEKVGPAVEELMRFLTIVQFGLGRVA

TEDLELGGEQIAAGDLVVVAMNVANRDPRVFAEPERFDIDRKMVRHMAFG

YGVHACLGQNVARAELRTILPKLFTRFPELRLAVPLEEVDMDFTGTNYGV

RSLMVTR

>CYP154AJ1(645971874)Streptomyces sp. SPB78

MDDDGLFVIDPAGTDIHGEGARLRARGPVTRVLLPGGLAAWSVTGYDAAR

QVLADDRFAKNAREHWPAYIEGTLGPDFPLIAWARMDNMSTADGERHTRQ

RHLVAGAFSPRRIAFLRPRVERVVAGALDELAAYAAERPGEPVDLKLRYA

HPVASRVIGELLGVPDGDPDGILDRAGYVEPNPRKAAAEFAALRDKIEAL

VAHKRREPGEDLISDLIAAGSGGCPVSGAGEGSDAEVVGLAQLMLNTGAE

PARNLITNTALALLTRPGQRALAEDGEVGWEDVVEETLRTDAPVAHLPFR

FATEDVTIAGVTISRGDPVVVGYAATGRDPQVHGAAAGEFDARHEDKRHV

AFGHGVHRCVGPVLSRMEVSIAVRELFTRFPGLRLAAEPGQLPGQGTFVM

NGRLALPVHL

>CYP208A(645971899)Streptomyces sp. SPB78

MPHITGRIRRRVLTPKVSETLLEKRGFHRKDPESQRLLETVGARFLEGYG

HAVAASSTAEAEEGLRQVPDQFRGFAYEGAGMGAAMLDGIPGLGRGHVRR

LLAGEGAAHEYMIYVGIGWSMGRLPKFLWADIDALDPLLRWLVLDGYGFH

QAYFRTERYVRQHYREARFPWPAHDHPEYAGNAIDQGIGRALWFVCGTDT

DLVADTIGTFPEARRSDLYGGVGLAATYAAGSGERELTGLVKRAGEHRGS

LAQGSAFAAEARLRAGLLMPQTARATRVLCGMEPEEAAAVTQDVRPRGAG

DGPAPGVRGVAPGDRRPDHRRRRGAAVRAPGVRLATRRRSARPRTGYASP

PVRALPSLLRQLAGDRLAMMTAAAGYGDAVRLPMGPKTLYFFNHPDHAKH

VLADNAANYHKGIGLVQARRALGDGLLTSEGELWRRQRKVVQPAFQHKRI

AGQADAVAQEAAALVARLRARAGHGPVRLDQELTGLTLGVLGRTLLASDL

GGFDGLGHAFEAVQDQAMFEMVTMNAVPTWVPLPGQLRFRHARARLQHVV

ERLTESGHPKGADAGDGTDALSRLIASVGEETDASTGRRRLRDELITLLL

AGHETTASTLGWTCYLIDRHPEIRERLHEEAVEVLGDRLPSYEDLQRLPF

TTATVEEVMRLYPPVWILPRQAQADDEIGGLHVPAGADVLVSPFTLHRHP

RFWEAPEQFRPERFLPGARGDRPRYAHIPFGAGPAGLRGEQPGAHGGGLH

GGAALPRAAPGLGAGSPGGGAADALAGHEGRAARDGPPRAVTACGGAAGP

AAPPRHPAARTMSVSNAFDRTRCRATRMGGSRTRISRSRNAVGLTGASCR

ISSGPREPT

>CYP107U(645972219)Streptomyces sp. SPB78

GDDLISALIQAADDGEHLTENEAAAMAFILLFAGFETTVNLIGNGTFALL

QHPGQRASLHDALAGPDHGDALLTTGIEELLRFDGPVELATWRYATESFD

LAGRHIRSGDPVLVVLAAADRDPARFPGPATLDLARRDNQHLGYGHGIHY

CLGAPLARLEGKTALARLFTRLPDLALDADPEQLRWRGGLIMRGLRTLPV

RFTPSGQGCEQS

>CYP1200A1(645973893)Streptomyces sp. SPB78

MATSAPPQPSVDGIPNLDPVPTFVPLAARSGGGLPLIELPSGHTAVHLTR

YADVHRVLTDPSFGRTATNVEDGPSFLPTIMPKELLLNLDMPDHSRMRGF

VNADYSVAGVERLRPLVRELVTAKAARLRGAERPDLYATVLDTLPAEVNG

RFLGIPDADIAYYRPLGHTVQDASHHDVPGLVADFTELYTYLTGLVTGAR

PRIEGGVIDRFLAARDTVEPPLDDAELVGILLGSVLGADQNILSVASKIA

YVLLCRPVLWQRLAAEPGIAPRLVDELIRLIPLGNISAFPRIAGCRIELA

DGVVEEGDVVYPDAFAANRDPEVFPEPLLIDPDRTGKRHLQFGYGMHHCM

GAALARMEIAELLTTLAAEFPTLTLDTDPAGLTWDSGVILRRPVTLPVRW

>CYP107BX(645974035)Streptomyces sp. SPB78

MTSTLPAPLPPLHRIPSAPCAAPHPVVLPDGAAAWRVSRYADVREVLGGG

GFTRAALYAEEHDDRPSGGGIVDDPELLFNQDGPEHLRLRRTVGRAFTPR

AVARWEPWITATVEELLDGLVRHEGPFDLVSGLARPLPVTVITRLLGLEG

DAWEKIGYWSDLAFTDGSHSAAEVESGLADFTSFGGELLALRRKEPGDDL

VSGIVRAADAEGGIPESQLVRLVCGMVVGGHDSTMTMLGNVLLYLLGERR

DAWPRLTDVDAAGLAADRLLHLVPLGEDPGSLRRCVADTDLGGVRVPAGA

IVVADIVAANRDPEVFPPAETDDLFRELPAPTLAFGGGPHYCMGAWLARL

ELRTALNRLATRLPELRLVPGDGGVEWRLGTTSRGPKRLMAVG

>CYP170B(645974418)Streptomyces sp. SPB78

MSPRTPPTAPPAGTPRTAPLAAGALPLLGHAPALARGPLPFLEGLRAHGE

VVRIALGPKQVLAVCAPDLVGTLLTRPEFTVGGPLWDTLEILLGKGVATS

NGPRHRRQRRTIQPAFRPERIASYARVMTEEAVRTAGSWRDGETTDVGAE

MFRTGVRIVSRSLLEVDATGTELADRIAHALHTVFGGLYQRMILSFGPLH

RLPTPANRRFNAALAELHTLVRQLVREARAHDSGEAAPDDDLLSVLLHAT

GENGAPLPDEEIHDNVVSLVVAAAENVAATLAWTFHLLAQHPEWERGLRE

EVESVAPDRPVDFEDLPKLTLTRNLLSESMRIRPAAWIFTRRAASDTELG

GYRVPAGTDVLYSPWALQHDPRSFPEPHRFDPDRWLPERAAEIPRHAFIP

FGIGNRKCPGDIYSLTELALVLANTVRDWHVENVPGRTDPRPRIGITLQP

RATVLRVRRA

>CYP1972A1(645975170)Streptomyces sp. SPB78

ASADLVPEFCDWLPAVALGAALGLPYRRTEALRRWCRAGGPAPLPLPDAL

AVRARGAERATAYALASLLGTLLTRPAHLAAVRDEPALIDAVWAETLRHD

PPAPYLVLRARERVRLPGGAVAAGERVLCLLGAAGWDPAGYPDPGRWDPY

RAGPAPVPAHPWPDLGLLTVRYGLPPLLGVQGLALAPGFAPRAVGVRVRG

PRRLLVRLGR

>CYP1199A(645975459)Streptomyces sp. SPB78

MTSHTQTRPHERETLFAPPLEALLRDHLGADLFRPEPDTVGVAGPALVDR

ILAARPAGKTERPTFKPLLGRAIPHQQAATQMQAIGHDVRAALKREAPRD

PDLSGPWPHVGHVYLRDLILGGDPRRLRVLMNRALELTPKLTWTVIAAGA

ALPAPRRRTGTTRLGALSTGAATYQERRYAMGLYRRTAAPVCFTISTLVA

NALWLGAPFAAEVPSRHLVHESLRLLPPSWNILRNSSPEYPAIDARIAPA

DDVLVLPFLSHRDPALWENPEEFRPERWEELDPERVPGYLPFGHSSERCW

GRHMVMPLAEMLLDMIRSSGLVVDPRQRTARVPLTGLMGVDQVRLVQP

>CYP1240C1(645975493)Streptomyces sp. SPB78

MSNRYVPGATPLYEPEFAADPHAAYARLRARHGAVAPVEVAPGVGAHLVL

DYRAAWEILTDQDGHWSKDPRPWEASLPSTPEAHGVLGMIGYRPNALFTD

GEVHDRFRQAVSDCFRMIQPHRLRATVTEIADDLVREFAAEGRADLVGQY

ARPLMARVINRLYGRSDASGRDLDRILTTLSNAVGADAVAANEEFGRYMN

ELITAKYESPGDDLPSWFIGHPAQLAPEEVVQHVVLTLGAGNEPSANLIS

NTTARMLTDERYYSSLTDGALSTREALAEVLRTDPSMANYGPYFATRPVH

FHGTWIQPAELVLVSYAGAGSDPTGLPAHADERSDGGAHLGFGAGEHRCP

AADPALLIAATAIDRLLTLLPGLELAVPFDELTWRPGPFHRALAALPVTF

RPVRPDQPGVTPWTNSKPSLSTL

>CYP154U(645975494)Streptomyces sp. SPB78

MDQLQAVTLDPVGADIPAEAARLRERGPVVGITLPGGIPAFAVTRYENLR

TLILDPRVSKDPRKHWDRWPEASSNPEWGWILGWVGVINMLSTYGVDHSR

LRKLVAPSFTHHRTERLRGRVEEIVTRLLDEIEARPEGEVVDLRAAYARQ

LPMEIICELFGVSGAHKAELVTLLDDFMDVSRPVEEAVATLARLYAVLGE

LIGTKRAEPGDDLASDLVSVRDTDGDRLSDDELRDTLLLVIGAGVETTVN

LIGNAAFALLTHPEQLAQARAGELPWEKVVTETLRWAPSIANLPMRFAVE

DIQGPETGGVLIPRGSAILTTYAAANHDPAHYGATAHLFDAHRDSGDHLA

FGIGAHRCIGAPLAQLEAGVALPALFPASRACASRTR

>CYP107P(645975505)Streptomyces sp. SPB78

MPPDDAARTGPGPRGGLVTRSRALYRPAMSAPAAAPRPLAPAAFDHGDPA

FLADPYPVYAALREAGRVLHYAPSGQWLVPHHADVSALLRDRRLGRSYQH

RYTHEEFGRTPPPRAHAPFHELNDLGMLELEPPDHTRIRRLVSHAFTPRT

VEALRPYVTGLARDLADALAERGGGDLLTEVAEPLPVAVIAELLGVPEAD

RPLLRPWSAAICAMYELNPSEETARRAVAASAEFSAYLRALIADRARRPG

DDLVSALVAAREAGDRLSEAEIVATCALLLNAGHEATVGATVNGWHALLT

HPDQLAALRADHGLVPTAVEELLRYDTPLQLFERWVLDAIEIGGVTIPRG

EELALLFGSANHDPAVFAEPHRLDLTRPAVPHVSFSAGIHYCVGAPLARL

ELTASLAALLDRAPGLHLAGEARRRPNFVMRGFAELPVGV

>CYP157B(645975726)Streptomyces sp. SPB78

MKNPLFPPPSDDYGPEPLRPWDHRPKESATPPLRGPTAAGGPPPACPAHG

DRWEDEPVRLSTLDSDRPPAELYRELRARFGPVAPVLLDDDIPAWLALGY

PEVAYVTGHDEEFGRDSRRWHQWENIPGDWPLLAYAGYQPSVFFAEGEEH

RRRAGALTRALEAMDMYDVSLVCESVGRRLIDRFVHDGRVELLESYVHAL

PVRVMIELCGMPAGEDITENLAEDLRITLDAERGENPVEAYRRVARQLGA

LVTARRERPERDLTSYLVTDEAALTDTEAVHDLTVLILAGQQPTSNWICN

ALRLLLLDDRFADKVAGGRVGVPEALTETLWLDTPVQNFLGRWATRDTEL

GGRPVKQGDCVLLGLAAANTDPELWPHGGMGEGSTAHLSFSMGEHRCPHP

SPQMARVIARTAIDTLLARLPDLAVAVEAEELRWRRSLWKRGLEELPVVF

SAGGVREWTDRGAGRARSRETGLLRD

>CYP125A(2521766734)Streptomyces canus 299MFChir4.1

MSCPALPDGFDFTDPDLLQSRVPLPEFAELRRAEPVRWIPQPGNVAGFQD

EGYWAVTRHADVKYVSTHPEIFSSYLNTAIIRFNERIERDAIDAQRLILL

NMDPPEHTRVRGIVQRVFTPRAIRALEERLRNRAHAIVESASALPGGSFD

FVTQVACELPLQAIAELIGIPQDDRAKIFDWSNKMISYDDPEYAISEQVG

QESAMEVIAYAMNMAADRKQCPAKDIVTTLVAAEDEGNLNSDEFGFFVLM

LAVAGNETTRNAITHGMHAFLTHPDQWELYKAERPSTAAEEIVRWAAPVN

AFQRTATQDTELGGKQIKKGDRVGIFYAAANHDPEVFENPDVFDITRDPN

PHLGFGGGGPHYCLGKSLAILEIDLIFNAIADAMPNLRATGDPRRLRSAW

VNGVKELRVTLG

>CYP145C(2521767580)Streptomyces canus 299MFChir4.1

MPMRTEIPVYHPDLYSASAIRDTYPHYAALRELGPVVWLSKQKVYALPRY

AECKQVLLDDDTFVSSGGVALNPVANRVGQGTTLFSDGEDHARRRSLLAH

RLTPRALRTMKDTVDQQSAAVVEDAVARRTVDAVEVATALPMSVVPDLVG

WPRQGREHLLRWAGATFDALGPLNCQAARTLPASLGMLRYARGVVRDRSA

LDGSMGHDLLRAADEGQIMPAECATMMIDYLAPSLDTTISAISSALYLFA

LHPEQWRLLKANPDLVPKAVNEVVRYESPIRAFSRTAAHDTELAGIALPK

GSRVLVLYGSANRDPLEWDDPDTFDIRRDAARQLGFGQGTHGCAGQGLAR

METSAILRALVERVDRIEVTGVPEWALNNVIHRLERLPLELIPA

>CYP102B(2521767798)Streptomyces canus 299MFChir4.1

MAETTTTATRPTGFRSAELGWPELHRIPHPPRRLPLLGDVLGVDRATPLQ

DSVRFARRLGPIFRRRAFNREFVFTWGAELVADLADESRFAKHVGLGVAN

LRPVAGDGLFTAYNHEPNWQLAHDVLAPGFSREAMAAYHPMMLAVAGQLT

DHWDREQMAGRSVDVPGDMTKLTLETIARTGFGHDFGSFERDRPHPFVTA

MVGTLTYAQRLNSVPFPALVRRAARRNADDIAYLNRMVDDLVRARRGARG

DGDLLDRMLETAHPETGERLSPENVRRQVITFLVAGHETTSGALSFALHY

LSRHPEVAARARDEVDRVWGATDAPGYDQVAKLRYVRRVLDESLRLWPTA

PAFAREAVEDTVLAGQHPMRRGAWTLVLTPMLQRDPEVWGADAERFDPDR

FDPKAVRSRPPHVFKPFGTGARACIGRQFALHEATLVLGLLLRRYELRAD

PGYRLSVTERLTLMPDGLRLHLERRAGSAAMAAPVANPDEAPVPASESRC

PVHRAGD

>CYP107BX(2521767828)Streptomyces canus 299MFChir4.1

MSTDSTARTLCPMHRIPLPENGPPRLTTLATGTPVWLVTRYADVRQVLMD

PRFDRQSLRDEDAPPLLLVPNLLDSPDGLLNQDGPPHQLLRGTVQRAFTP

RAIARWRPWTASVVETLLDDLARQPQPADIVEAFTRRLPVSVISRLMGLE

HADWERIRDWADHALSGGAHTAEEVGAAMREFGLFCAELIAERRKEPGDD

LVSALVVAGDGLGIEEPRLVVLVLGLVVAGHETTMTALGNIVVHLLTDGR

DAWPGLAESQEAAGTAVEQLLRTIPLSEGRVLPGLIRRAVDEVEVGGVTI

PAGAVVAVQTNAANRDPDVFPRPEETDLFAPLTTPSVVFGAGPHHCLGAW

LARLELGLALHRLAVRFPGLRAEFTPEAIAWREGQMTRGPRRLPVSW

>CYP105D(2521767849)Streptomyces canus 299MFChir4.1

MTETEPVAFPQDRTCPYHPPTGYDALRADRPLSRITLFDGRPAWLVTGHG

TARALLADPRLSTDRTRDGFPAPTARFAAVRNRRTALLGVDDPEHRVQRR

MMVPAFTLRRATELRPRIQEIVDERIDAMIAQGPPAELVSAFALPVPSTV

ICALLGVPYTDHDFFEGQSRRLLRGPTADDVMDARDQLEAYFDELIDRKQ

KQPEPGDGVLDELVHRQLRDGEVNREELIALAIILLVAGHETTANMISLG

TFTLLQHPDRLAELRTDPALIPNAVEELMRMLSIADGLLRMALEDIEVAG

TTIRAGEGVLFSTSVVNRDEDVYTDPDTLDWHRSARHHIAFGFGIHQCLG

QNLARAELEIALHTLFERLPTLRLAAPAEEIPFKPGDTIQGMLELPVTW

>CYP156B(2521768044)Streptomyces canus 299MFChir4.1

MDVHDTAAAGAARCPMYDDSFAADPQKVYDYLRAHGPAGPVELAPGVDAN

LVVDHETALRVLQNPNLFARDGRRWTALNDGRVPLDSPVLPMMAYRPNCL

FTDGAQHLRLRKAVTESLDRLNVTRIRRDVEPIAAYLIDQFSERGRADLI

NDYAKLLPLLMINKLFGCPAAIGDRVTSAMADMFDGKDALKASDELTACF

MELVSLKRREPGDDITSWLVQHSAGLSDEELKDQLVMLIGAGVEPERNLI

ANALLLLLDRDGEDGPSMQIEDALDHVLLNNPPIANYATHFPLQDVDLGG

VVVQANSPLVISFAGANSDPALASSGQAHSRGAHLAWGAGPHACPAKSPA

QVIAVTAIELILNALPDLLLSVPAQELQWRPGPFHRALATLPVQFSPTPA

TRMASAYGVPTAARPTVVQQPQQAARVSATAAQKPAKKGFWSSFLEIFRV

>CYP157C(2521768447)Streptomyces canus 299MFChir4.1

VTPESFSPAGTYDPAGAPPGCPAHGIGPGGLRRLYGPGAEDLESVYEELR

TEHGAVAPALVHEDVPIWVVLGHSENMHLVRTPSHFSRDTRLWGPMQDGT

VKPDHPLMPHLAWQPLCCHAEGDEHLRLRAAVNGAISTIDHRGVRRHINR

ATQHLVNRFCEQGRADLVSQFAERLPMMVMCEILGMPDEYNDRIVEAARD

LPKGTETAIASNEYIMDVLMRLTARRRAQPEDDFTSHLINHPARLTDQEV

SQHLRLVLIIAYESTANLLANVLRVVLTDPRFRAQLNGGQMTVPQAVEQS

MWDEPPFSTILGYFAKQDAELGGQRIRKGDGLILGIAPGNVDPRVRPDTK

ANMQGNRSHLAFSGGPHECPGQDIGRAIADVGVDALLTRLPDAELACDED

DLQWEETLSNRHLVALPVKFAPRSQQDVMARPAHPAPSQRRTDWQVSTPQ

REPVAPEPVAPAPVPEPAVRLGAFQRLLRWWRGE

>CYP107BU(2521769129)Streptomyces canus 299MFChir4.1

VIAFPLGAATTPAELAGDPHPRLAELRAHEPVSWLPELNGWLVTRRDLAL

DVMRDAETFTVDDPRFSTARIVGPSMLSLDGDEHTRHREPFTEPFRPRAV

RDGFAEFIERETDRLVTGLEPAGAAELRRAFAGPLAVAVVTEALGLVDAG

TDTVLAWYDAIVRSVSDITEGRAAGPAGPEAYVQLRRAVEATVAQGADSS

LLASAAGRLALPEVASNAAVLMFGGIETTEAMITNALLHLLRHPDQLALV

QADFALLDNAIEESLRLEPGAAVVDRYATRDLTLGPAAIRRGDLVTVSLT

GANRDPAVFPDPDRFDVRRPNARLQLAFAHGPHYCLAAHLARLETRIALR

HLLDRLPALRLDPDHPTAPHGLVFRKPPTLHVLWG

>CYP107L(2521769884)Streptomyces canus 299MFChir4.1

MAEAVIDLGEYGDAFRVDPHPVYARLRGLGPVHRVRPPGSDADYSTWLVV

GHEEARAALADPRLSKDGRRIGMVFDGEGLIGRHLLGSDPPEHTRLRGLV

SRAFTMRRVEQLRPRIQRITDDLLDAMLPHGHADLVQYLAYPLPITVICE

LLGVPEMDRTEFRKLSTEVVAPTSPESSYDAVRRLGAYLTELIEDKRCAG

PSDDLLGDLIRTTAEDGDRLSPSELRGMAFLLLIAGHETTVNLITNGVHA

LLTHPDQLAALRADMSLVDGAVEETLRYEGPVENATFRYAAEPLEIAGRT

IAKGDPVMICLSAADRDDSRYPAPDRFDIRRDPRGHLAFGHGIHYCLGAP

LARLEARTAIRALLERAPALALDGPTGEWLPGMLIRGVRSLPVRWQDSTA

>CYP107P(2521770067)Streptomyces canus 299MFChir4.1

MAAFDPWDPAFLADPYPAYAGLRAHGRVQYYEPTNQWLVPHHADVSALLR

DRRLGRTYQHRFTHEDFGRTAPPAKHEPFHTLNDHGMLDLEPPDHTRIRR

LVSKAFTPRTVEQLKPYVTRLAGELVDRLVARGGGDLLTDVAEPLPVAVI

AEMLGIPETDRAPLRPWSADICGMYELNPPEDVAARAVRASVEFSDYLRD

LIAERRKEPGDDLISGLIAAHDEGDRLTEQEMISTCVLLLNAGHEATVNA

TVNGWNALFRNPGQLAALRADHSLVPGAVEELMRYDTPLQLFERWVLDDI

EIDGTTVPRGAEIALLFGSANHDPEVFRDPERLDLTRTDNPHISFSAGIH

YCIGAPLARIELAASMTAVLERAPTLALAAEPERKPNFVIRGLEGLAVEV

R

>CYP170A(2521770691)Streptomyces canus 299MFChir4.1

MTVESVKTEATVAPESGEPPLAGGGVPVLGHGLRLARDPLSFLSGLRRHG

EVVRLRLGPKTVYAPTTPELTGAVALNPDFIIAGPLWESLEGLVGKEGVA

TANGPQHRRQRRIIQPAFRLDAIPAYGPIMEEEAQALAERWQPGETVDAT

LESFRVAVRIAARCLLRGAYMDERAERLCVALTTVFRGMYRRMVIPAGPL

YKLPLPANLAFNRALADLHRVVDEIVAERRASGQKPDDLLTALLAAKDAN

GDPIGEQEIHDQVVAILTPGGETVASTIMWLLQVLAEHPEHADKVCAEVE

SVTGGRPVAFEDVRSLRYTNYLVVETMRLWPAVWILTRRAVRDTELGGYR

IPAGADIVYSPYAIQRDAKSFDDSAVFDPLRWSPERAKSVPKHAMSPFST

GNRKCPSDHFSMAQLTLLTAALARKYRFEQVAGSDDTPRPGITLRPNRLL

VRPMPR

>CYP107X(2521770907)Streptomyces canus 299MFChir4.1

MDPADGLLDHPYDVYRRLRDTAPVHRIAGPDGTPAWIVTRYDDVRAALAD

PRLSLDKRHATAGTYKGFSLPPALDANLLNMDPPDHTRIRRLVGRAFTPR

RIERLRGPIRNTADRLLDALGDDGTTDLIASYAAPLPITVICDLLGIPDE

HRLDFRVWTDTLVAPDPAAGPGAAKEAVVAMLGYFTRLLADKRRDPADDL

LSDLIAVRDEGDRLSEDELMSLAFLILFAGYENTVQLIGNAALALFQHPE

QLTALREDPSLLPAAVEEFLRYEGPALLAIRRFPVEDVTLGGVTIPAGET

VWVSPSAANRDPARFPDPDRLDLDRPDASGHLSLGHGIHYCLGAPLARAE

TEIALAALLERFPDLALADPAPRWRASLRARGLLALRVSYGRNLPHDR

>CYP182B(2521771039)Streptomyces canus 299MFChir4.1

MGVQLDSGSAAGHESLESMSLEPLMTRDYERDPSIVYERLRQAHGPVAPV

DLLGVPVWLVLGYEEAYEVLRDDHAWPKGLENWRARREGRLPEDWPLAPS

LDVNHVLIQGGEGYRGLKVAWDTALRPFQDPRHPVAKRLKAQITRDADEL

ISLLTQGGRTGWADLSAQFSRPLPLMVASHLLGFPGSQHDDALMDMWRVL

DAGPDAGPALERLLATLSELGAWKMKEPGDDFPSYLLASHPGLSVEELSR

ELMMLLGMTSDHVGILISNTVVEVITGNLPASASLSAGLIRETMNRVVMQ

KPPLINFVPRFPLKDMRLGNYTVAAGDPVWVSVGAAHADPAFAGKVCPES

TISTRAHLSWGAGPRQCPARELAGGAAAIGVSRLFERLSGLELSLPVDQL

PWRSSPFIRGLRSLPVRYTVSDTPAQPVPPPPPLPLPLPLPAAAGSAPAD

EAAGRPRSSLWRYLAGLVRGR

>CYP107U(2521771758)Streptomyces canus 299MFChir4.1

VTDHPSATPAPTTPALAPPTPELFSWEFATNPYPAYAWLREHAPVHRTRL

PSGVEAWLVTRYADAKQALADQRLSKNPAHHDEPAHAKGKTGIPGERKAE

LMTHLLNIDPPDHTRLRRLVSKAFTPRRVAEFAPRVQELTDRLIDGFAAT

GTADLIHDFAFPLPIYAICDLLGVPREDQDDFRDWAGMMIRHGGGPRGGV

ARSVKKMRGYLAELIHRKREALPEEPAPGEDLISALIRASDHGEHLTENE

AAAMAFILLFAGFETTVNLVGNGVYALLTHPEQRNRLQESLAGPDRDLLE

TGVEELLRYDGPVELATWRFATEPLRIGGQDIAAGDPVLVVLAAADRDPE

RFADPDVLDLSRRDNQHLGYGHGIHYCLGAPLARLEGQTALTTLLTRLPD

LQLAVDSADLRWRGGLIMRGLRNLPVEFTPVR

>CYP183BA1(2521772917)Streptomyces canus 299MFChir4.1

MSAIETIPRASGILPLLGHIAPLARDPLEFVSNLHSYGPLVRIQMGTKPV

VVVNDPAVIRKVLLDDRTFDKGGPLFERGREALGNGLITCPHSDHRRARR

LSQPAFHPKRMAGYAAIMTEHADKVTRSWHDGQVLDVTSEMMALTVEVTV

RTLFTNSLPRATASQITDDFLTLMTGFFRRMISPPLLNQAPSKGNREYAQ

AIRRLRRTVGDLIAARRADESDHGDLLSALCSAADPDSMTGRTDTELVDE

VLTFLLAGTETTAGTLSWAMHLLSQNPEVERRVYQEVESTRRGSPLDFTH

LPALETTGNAVKETLRLYPAVWMLTRVTTSDVVLDGHLLPEGTIVAFSPY

QVHRRADLYDEADQFTPDRWRDRSRAQEIYIPFSAGPRKCMGDQFALTQA

TLAVATVVSRWQLSPITDKPLQPSVKASLTPRRLRLRVTARGSQRQADEP

AGGTTHAEG

>CYP183B6(2521772959)Streptomyces canus 299MFChir4.1

MKKLPSIPAAPKGLPLVGHLLPLLRNPTAFLDSLPAYGDMVKIRVGPFSM

VVLCDPGLTHQVLLDDRTFDKGGPIYDRAREVSGNGIGTSHHDEHRRLRR

LVQPSFHPSRLPGYAETMTACIDETASSWRSGQILDVPAEMMAITSKIAI

ATLFSGALSQDELDKLLDASMTLLAGFYRRMFLVPPLDRLPLPSNRAYTR

ARNHLHEVCGQIIAQRRADGTDHEDLVSALLAAHDVETDGRGMTDAEIID

TIVAILLAGIENTASALAWSLDFLAQHPEVERQLHAEVDTVLNGAAATHA

DLHHLEFTNRVITETLRLRPPAWFFTRTVTADTHLGGHFLPAGTNVAYSP

YLIHHRHDLHNAPEDFDPDRWDPQRPQPHRHAMIPFASGARKCPGDTFAM

AEATLALASITARWQLEHAPGIERRPALSLTLKPRALPMRTRPRTVVR

>CYP251T1(2521772960)Streptomyces canus 299MFChir4.1

MTTTTVPHLPPSARGRLPLIGHALQFVRGPLNVLQGLRGQGDIVALHVGK

LPIYVVNSPQLLHRVLVTDADSFTKGRMYDKTRPVVGNGLATSEGALHHR

QRRLILPAFHRPRLRGYSEVMREQAEAVSASWQPGQRIAVDRAMHTMSVG

TTVRTLFGTGIDSATIAEIDHCVGVFLTQIIVRTMTPNVVERLPLPGTRN

FEAARKRLKAIVDSILDVRLRATEEPDDLLTLLLAARDEETGQPLSRRQL

HDEVVTMLVAGADSTAYTLAWLLYELGRNPAIEARLHHELDTVLDGRPVT

FDDLPRLQYTQRLIQETLRLHSVSWIQMRRTITPVTLGDMHLPAGAEILF

SATTMHRDPDLYPDPLRFDPDRWLTPPRRESFQPFSAGPRKCPGDHFALT

QLAIGIATIAARWRLVPATRAKVREVPAAVLRPNRLPMVVQARD

>CYP163H(2521774073)Streptomyces canus 299MFChir4.1

VTVTQTGVPADLGEVNLADPATFVRLDMDGTWQRIRAERPVWRHPKTSFG

PEFWALSRYADVLSLFSDDRFSSVPGNMLPSLLKAGGDPASGKILALTDN

PRHKALRTNFLKAFTPRIRALIADRLQERTDHLIGARIGIGEFDFAREVA

EPIPIGTICDLLGFPPEDHERLLELSRDALSSDEVGQTEEDTWLARNELL

LHCMQLMEQRRADPQDDLVSVMVNCLVDGEPLTDDEVIVNIYGFILAGDH

TSRLAMVSALLEFARRPEQWRALKEARVSQSTAVDEILRWSTPVMHIGRT

ARADVHLGGQLIRAGDLVTAWTIAANRDESVFPDPGDFDLARKPNKHLSL

GHGPHFCLGAYLGRAEITAVLNTLVNTVDSIELAGEPQAVYSTFLRGYSS

LPLALTGRPKALER

>CYP113AF1(2521774245)Streptomyces canus 299MFChir4.1

MTDLAALTGLSFDLRTKAFLDYLDRRRAQTSVVKSDQPGVWEVFGYDEAV

QVLGDHGTFSNDMNDLIPEDERELASAAQGNFLGIDPPRHTQMRELVRRA

FTPHVVAGLEPVIERTAEQLLDEVELSADGTGTVDLVAGFASPLTARVIA

KLFGIPEQDHVLFTIWADALLGARPAGELGKADESAVRELGDLVRSASAY

LLELIHERRAHPRDDLISSLAQAEVDGRGLDDHEIVGVIGMFVIAGHLPS

SVLIGSTVMALDTHPDTFAEVRADHGLLPQVVEEVLRWRPPLVRDQRVTA

CETELGGERIPAGAMVCLWLASAHRDASRFPQADAFDIHRENRTRHIAFG

KGIHFCLGAPLARMETRIALTALLRRYDRIEVLRDGGIQFHPSIGVLGPV

RLPTAVHALAPTV

>CYP158A(2521774410)Streptomyces canus 299MFChir4.1

MSEETITEILPPIRHWPALDLNGVDFDPVLTELMREGPLTRIRLPHGEGW

AWLVTRYDDVRTVTEDPRFSREAVMDQPVTRLDPHGTPRRGAVTFLDPPH

HTRLRRSVAAAFSAEGVERIRERSRRMLDDLVDELLQDGPPADLTAAVLS

PFPAAVLCELMGVPAADRHGMHAWTRLILSPSRGARSGERAREEMGAYFA

DLIGLREGGTGEDVASLLGAAVGRGEATLEEAAELAVLLQIGGAAVTDNT

GQLFHLLLTRPDLAERLRAEPEIRPRAVEELLRFIPHRNTVGLARIALED

VGIRGVRIRAGDAIYVSYLSANRDPDVFPYPETIDFSRSPNPHMAFGSGP

HHCPGGTLARLVTGLLVDALLDRVPGLRPSVPPEQVPFKKGTSIRGPEAL

PVTW

>CYP1530A4(2521774701)Streptomyces canus 299MFChir4.1

MHVPSATPRQHDEDGEDGRIRSRLTVSDLPEPPGLPLLGHLPALARGGTM

HRTLSDWRDRYGPTFRVRLGRKDAVVTSAPAILDTVLRERPDTFRRARSL

SDLIAELGASGLFDSEGADWQRLRRMATRGLNSGQLRDSYSAIARSTGRL

RERWEAMAGARVPVLEDLMRYTLDVTVALTMGHDLDAVRRADEDGLHQRL

PLVFDTLTKRMNSPFPYWRWMRLPGDRRVDAVVAEAGALIRELYADSRHR

MGAGEKPRTYLDSLAMASLDNDEHITEGDVIGTILNMMIAGEDTAAATTA

WAIHHLAGHPEVQQRVRTEAEAVLGSDGYPAEPADLARLPYAGAVVNETI

RLNPVSPFILLEAVTDTTVSDGTTDLHIGRGTVIVVLLTHGSDRDPLRYP

DPGRFEPERWLSSGARTSPQEQPFMPFGGGPRFCPGRNLALMESTLVVAM

TCHRFTVEPDTSAGPVGERVTLAVLPTHLNVRLHPASASAQ

>CYP107AE(2521774761)Streptomyces canus 299MFChir4.1

MISMRARMGSSRQLTLKRPVLGVSSMTVSQSPLESESMTDPTQDPRFLQD

PYPALAAMRSRCPVQSMPTASGGRPSYLVTGYTEAREALCDPRLSKDTAA

FFAGKESRRRLHPAVAHTMLATDPPRHTRLRKLVTKAFTAGAVARLRPFI

GRVTEELLDRWPVGERFDFVSGLAVPLPVIVICQLLGVPQEDRPDVQRWS

GELFAAGRPDVIDAASHSMAGYMTGLVAAKRLDPGDGLLDRLISARDGAE

RLSEEELVSLAVLLLVAGHETTTNFLGNAALALLQRPAALDHLRRNPDDV

PAALDELLRFDSPVSTATFRFTTEAVALGGVDIPPGAPVLVALGAANRDP

VRFPSPDLLDLDRDAAGHLSFGHGIHRCVGAPLAKAEAEIALRAVLTRFP

GIRLAVPSDRLEWRRTRLVRGLAELPVTV

>CYP294B1(2521775056)Streptomyces canus 299MFChir4.1

MQWAKAPSGVGMWVVSDYHLARQILSDKRFSRSLAAGPNGAKMGITDPSP

ESIISMDGQEHAELRRLVAGTFTHARIAELQPFVETLVTELLDDVASRGP

QGDLVSGLAVPLPIHVICHVMGIPLEDRDYFAGHVAVLFDLTGAPGDGED

RTLSLIRYMMGLIARKRREKKRGTDLISVLIEACDNDGKLTNRAMVTLCL

AILMAGYETTVDQISLTILDLLSQPEKTDALRNKPSLIPDFVEDALRTNP

AVTTTFARMATESVEMAGMTIEPGEAVIVDVLGANYGLPEVETQKSFRPM

HLTFGHGVHRCLGSHLAKIQLTEAVRGVLTRLPAVSLAEDIHALGWKTGH

ATRGLNRLLVSW

>CYP157B(2521775516)Streptomyces canus 299MFChir4.1

VSDPTGSPSAPTPPPGCPAHDAAVQLGGLEYQQTPSQLYRSLRREHGAVA

PVLLDNDIPAWLVLGYPEVSFVTSHDELFARDSRRWNQWDHIPADWPLLP

FVGYQPSVLFAEGEEHQRRAGVITQALEGVDQFELSRECELIADRLISSF

SGSGQAELMSAYAHALPARGVLWMCGMPQDSTDTEQLVDDLRISLDAGEG

DDPVAAYTRVGARIMQLVKEKRERPGPDVTSRMLLDPAGLSDEEIVQDLI

SVIAAAQQPTANWIGNTLRLLLTDERFALNVSGGRVSVGDALNEVLWLDT

PTQNFIGRWAVRDTQLGGRLIREGDCLVLGLAAANTDPQIWPDSHVGAEN

SAHLSFSNGEHRCPYPAPLLADVMARTAVETLVERLPDLVLAVEPEELTW

RPSIWMRGLTSLPVQFTPAVQ

>CYP159A(2521775517)Streptomyces canus 299MFChir4.1

VSTAQQVPDILSPEFAEDPYPAYAAMREKEPLIWHEATQSYIISRYADVE

RVFKDKKAEFTTDNYNWQLEPVHGKTILQLNGREHAVRRALVAPAFRGSD

LQEKFMPVIERNSRELIDAFRHTGTADIVSDYATRFPVNVIADMLGLDKA

DHARFHGWYTTVIAFLGNLSGDPEVTAAGERTRVEFAEYMIPIIRERRER

PGDDLLSSLCAAEVDGVRMSDEDIKAFCSLLLAAGGETTDKAIAGILANL

LRHPDQLAAVREDRSLIPAAFAETLRYTPPVHMIMRQSATEVELTGGTIP

AGATVTCLIGAANRDEQRYREPDRFDIFRTDLTTTSAFSAAADHLAFALG

RHFCVGALLAKAEVETGMNQLLDAMPDVRFADGFEPVERGVFTRGPQSLP

VRFTPTGS

>CYP105DY4(2521775649)Streptomyces canus 299MFChir4.1

MSNDTMAEAPAFPLPRGCPFAPPETATAFREAGVPRRVTIWDGSRPWLIT

RHDHVQQVLGPNGRFGADVTDAGFPNTSSAQPAVEGNIFFRKDGDEHLPI

RRILNPDFTVKASEKLRARIADLTEQLLDELADKAEPVDLIEEYALALPT

MVICEILGVPLDQRHAVHESAKKVVQLSLPKEEKLAAHRKQVENIRRTMA

DKRDHPDDGLLSRLVNTWVDDKNVLTFDEAVRLGMLVIGAGHETTANMIG

LGILGFLREPAQRDTLLADPRKNAAPAVEEMMRYWSMVQTEPRRVCLQDT

EIGGVLIKAGEGVICNLPAANRDPAVFTDPERLDITRTERRHIGFGFGVH

QCLGQNLSRVEMQEAWPRLFQRFPTLRLAVAESELHFHDDALTYGLEELP

VTW

>CYP107L(2521776051)Streptomyces canus 299MFChir4.1

VTDIVDLGEFGEELRRDPHPVYAQLRARGPVHRVRLPEPDPRPEVWLIVG

YEEARAALADPRLAKDDSKNGFTAFDEELIGKHLLAADPPQHTRLRSLIT

RAFTPRRVEELQPRIQQITDELLDAMLPQGRADLIESFAYPLPITVICEL

LGVPELDRTEFRKISNDAVLPADLDSMRDAFVRLADYLDRLIEDKQRGGP

GTDLLSDLIRTTAEDGDRLSPDELRGMAYVLLVAGHETTVNLITNAVHAL

LTHPDQLTALRADMTLLHGTVEEALRYEGPVETATYRFAAEPLEIAGVPI

AKGEWVLVGLAAADRDGDRFSAPERFDIRRDTRGHVAFGHGIHHCLGAPL

ARLEARIALRSLLERAPDLALDGPPREWLPGVLMRGVRSLPVRW

>CYP163B(2521776064)Streptomyces canus 299MFChir4.1

MNQDAQTGRGASDTASSDPLEELPADLSSLDLADPQTFLRHDQDELWRRL

RAEHPVFWNAPSGGRPGFWALTRYDDIMHVYRESSTFISARGNVLATLLH

GGDSATGKMLAVTDGSRHRELRKVFLKAFSPRTISTVAEKVQENTESLIR

SAVAEGSCDFAQDIAEKIPIRTICDLLGVPEPDQDELLILTKKALSAEDD

QITEFDSILARNEILVYFSSLLEEFRKNPREGVIGTFADAQASGTELSDE

EIVLNCYSLIIGGDETSRLSMISSIPALVEHPAQWAALSSGSIGSQTATE

EILRWTTPAMHFGRTTLKDTTVSGHAINQGDVVTLWNVSANRDEAVFASP

DVLNLSRSPNRHLTFGYGPHFCLGAELGRIEIRAMLDALCRFAADITLTG

TPKRVRSNFLSGISSLPVQFTPR

>CYP1037A(2523554979)Streptomyces sp. FxanaA7

MTVVTPDLAGVVDPELHASGEVHELWRWMRRHAPVHWHEPGDLPGFWSLT

RHDDIRDVYRNPAVFSSARGVLLRPTELGEDPGSGLTMALADPPRHRALR

GQLADRFSERCARSLAGEMRAEIRSVVARAVEAGTCDVVHDIGARLSGHN

IGRLLGVPPQDRERLLTWTTEAFRDGKPLTAHLPLMRYFIELMYARMEEP

ADDAMGMFVHNEVLGELSTETEILLGVENLVGASENAGLSMAAGILALAA

HPRQWERLVRERDGGFVRTAAEEVLRWTSSATHSMRTATEDTVLRGRRIA

AGDRVVLWIPSANRDESVFPDPDRFDLGREPNRHLALGAGEHVCIGSTMA

RHQTRMLLETLAERVAVVEPVGEAEPLRSITVNGPAHATVRLVAR

>CYP1038A(2523555043)Streptomyces sp. FxanaA7

MDTEAGPALIDLLTQARERLGGVAAFRLGPKPTVLVTDPQAVREVLVVRP

ERYVKRSHRARVLIGDGLLAADGAAWKRQRRMLQSQFTGTGMRRYEQRIT

AAARTTAARWDAYARTGETFDLGQEMRRFALDTIWRSLTGHALDDETERE

LAAVEAVGAALPALPADADEARDAIAAELARIDAVARRAIDDARGGAAGP

HGPGLLHVLTEASVEHPEYTDRLIRDEFVTLLAAGHETTATTLTWLHLLL

DRHPEARAQALADGDEGSAQRRQAVQALVHETLRFYPSAWILARHAAEDD

VLGGHTVEAGSDLLVCPYLTHRDPDLWPDPERFDPRRFTSPGGRPTHPGA

YFPFGIGPRACLGLQFALRESTVLLEHLLPAHLPVFHADPPKAVHGITVR

PDGPTPVSLKPPRD

>CYP157A(2523555527)Streptomyces sp. FxanaA7

LDFATSENGDGHSVSGAVSGAVPVPVPLFGARFQGEPASLYRELRWEHGE

VVPVLLDGGVPAWLVLGYRELHQVTSDPELFSRDSELWNQWPNIPADWPL

LPVISREQPSVLGTVGERHRQRAALIEEAWEAVDPLELRGHVERFADELI

DVVCGVGAADLVGQFAAPLPARVLAFLFGFREEHGPGLVAALNDILDGQG

RAVAAEGYLRSAMGRLVAERRQRPGDDVVSRLLANARAYEVTVEEVTHDV

MVMLAVGHQPTADWIGNSLRLMLTDERFAASLFGGRSSVAEAMNEVLWED

APIQNAAGRWATRDTRLGGRVLRAGDLVLLGLQGANSDPRVRTDGSALTG

GNNAHFSFGHGEHRCPFPAQETAEVIARTGIEVLLDRLPDLDLAVPAASL

TRRASPWLRGLVELPVRFSPGPVRGG

>CYP107U(2523555824)Streptomyces sp. FxanaA7

VTDHPSSLSSPAPSLFTWEFATDPYPAYAWLREHAPVHRTRLPSGVEAWL

VTRYADAKQVLADARLSKNPAHHDEPAHAKGKTGIPGERKAELMTHLLNI

DPPDHTRLRRLVSKAFTPRRVAEFTPRVQELTDQLIDRFAETGEADLIHD

FAFPLPIYAICDLLGVPREDQDDFRDWAGMMIRHGGGPRGGVARSVKKMR

GYLADLIHRKREGLTEHPAPGEDLISGLIRASDHGEHLTENEAAAMAFIL

LFAGFETTVNLIGNGTYALLTHPDQRTRLQTSLAAGETTLLETGVEELLR

YDGPVELATWRFATRPLTIGGQDIAQGDPVLVVLAAADRDPARFEAPDTL

DLSRRDNQHLGYGHGIHYCLGAPLARLEGQTALATLLTRLPDLRLAVDPA

DLRWRGGLIMRGLRTLPVQFTPQGRSEGIS

>CYP107L(2523555944)Streptomyces sp. FxanaA7

MAHRHVIDLGEYGPGFTENPHPVYAALRARGPVHRVRLPEHDAHHEVWLV

VGYEEARAALADPRLSKDGAKVGRTFLDEELIGKYLLVADPPQHTRLRRL

IAREFTARRVERLRPRVQEITDSLLDAMLARGRADLVESFAHPLPLTVIC

ELLGVPELDRAAFRKLSTEAVAPTSGESEYAAFVQLAAYLEQLVEDKRCA

PPADDLLSVLIRTTDEDGDRLSPAELRGMAFILLIAGHETTVNLITGAVH

ALLTHPGQLAEVRADMSLVEAVVEETLRHEGSVENATFRFAAEPLEIGGT

VVPAGDAVMIGLAAADRDGARYPGPDRFDIHRDTRGHLAFGHGIHFCLGA

PLARMEAGVALRALLRRCPDLASDGSPGEWLPGMLIRGVRSLPVRW

>CYP105Z(2523556018)Streptomyces sp. FxanaA7

MTAHPPLPTLRPLLDPSPEYAKWREEEPIRRVTIWGDNSPWLITRHEDAR

TVLADPRFSADTTRDGFPGFRPQSPPRAPGQFFMMDPPDHTRLRRVLIPD

FTFRRIEQLRPALARICGDLLDAMTADGAMTADLVTAYALPLPSLAVCEL

LGVPYEDHDFFQRQANAFSSLSSGPEEMMAARKALHTYLGELLARRGREP

ADDLLSRLARDRVATGEVSASEAVGIASLLLVAGHETTANMFPLAVVALL

RHPAQLAALRADPGLWPGAVEELLRHLTVAHSGLRRVATEDVEVGGVRIR

AGEGVVVALQAANRDPDAFPEPDTLDVRRSAAGHLAFGHGLHQCIGQSLA

RAELQVGLPALFDRLPGLRLTAPPEDFALTMTTVHGVRSLPVGW

>CYP107LF3(2523556370)Streptomyces sp. FxanaA7

MRPEPALPYPFAPRGLELHPAYAELREQPLARIRMPYGDGAWLVTRYEDV

RAVLADPRFSLAAAMGRDQPRMRPVARTGAGLFSTEPPEHTRLRSLVARE

FGARRVERLRARAVELADELLDRIVEAGQPADLVEDFSIPMPTTIICEVL

GLPAEDHRMLWAWAETVLSAVTPGEVLATEGQAFMDYMARVLELRGREPG

DDLLTTLVRACREQKLIGEEELLSIACDLLIAGFVSTTNQIGNFFHQLLY

NTAELARLRERPGLIPRAVEELMRYVPLLTGFSLPRYATADVELGGVTVR

AGEAVMIATAAANRDPRVFPEPERLVLDRPANPHIGFGHGVHYCVGAHLA

RLELRVAIERVLARLPGLRPAVPEHELRWKQDAMVNGLQALPVAW

>CYP157C(2523556621)Streptomyces sp. FxanaA7

VPRSPLRIPALPHHLRHRIHLSSKAITVTPESKSLTGTDDPTLGPPPGCP

AHGAGADGLRRLYGPEAEDLGAVYEKLRAEHGSVAPALLHDDVPIWVVLG

HGENLHMVSTPSQYSRDTRLWNKLQDGTFKPDNPLMPHIAWQPICCHAEG

DEHLRLRGAVTGAMSTINFRGIRRSINRYTQQLVNEFCEEGEADLVSQFA

EHLPMAVMCEILGMPEEYNDRIVHAARDMLKGTETAIASNAYIMEALMGL

TARRRANPQDDFTSHLINHPAGLNDEEVSQHLRVVLIAAYEATTNLLANV

LRVVLTNPGFRAQLKGGQMTVPEAVEQSLWDEPPFSTQLAYFAKQDTELG

GRRIRKGDGLLFGIAPGNVDPRVRPDLTANMQGNRSHLAFGGGPHECPGQ

DTGRAIADVGVDALLARLPDIQLNCEEHELRWRASISTRHLVALPVRFEP

KEKEVLEPRPLGRPVPAQRAGQQAATACPVSGAAPSGAAPSGTARSAPTA

AEPPARPGLLRRVLLWMSGR

>CYP107AM(2523557144)Streptomyces sp. FxanaA7

MSKVETGSTGTSDGEVVRLPMRADGPLDPPAAWERLRAQCPVATVELPSG

DRAKFVSRYDDVRALLSDSRFSRPRAGDDAARLSADGSGGVAADASPEYT

LAIPEHGEQHLRWQRQVGRYFTAKRMAALRPGMARIAESLLDEMIARGEP

GGPADLKAALGFPLPVYVICDLLGAPAEDRDRFSHWSDAFLNVSRFTKEE

TRTAFTKFAGYMSALVEARRAAPGEDLLSTLIVESAALPEEERLTDAELL

ATGMGLLVAGHETTANMIGKMVALLLADRGRWEQLLADPSLVRTAVEEVL

RFDANLGAFGMRRYLSEDVELDGKLLPGGTTVFCGMSSANRDDRVFAAPD

EMDLTRSPNPHLTFGAGPHSCLGQALARTELQVVLEVLLRKLPTLELAVP

AAELRKVEGLLVGGLREVPVRW

>CYP182A(2523557792)Streptomyces sp. FxanaA7

MRVGMETHPFADATAGGPGLESLPVEPLLTSEFDGDPGSVYERLRSTYGP

VAPVGLMGVPAWLVLDYREVLEVLRNESVWRRDVRHWRARAEGRLPRDWP

LLAGYEVRQTMFFDDEEHRHARLSYHSAMRPFQDGHSPEGWELRAAVARY

ADELIDVLAAESGTTGFADLAAQYTRPLLLMVTTKLFGCPVELGDEMVMD

LWRMLDGGPDAGAATGRALGTMTRLAAHRRSRPGEDLTSYLLLSDPSLSD

EQLGRELFMNAVYLNDITGNMVCNTLLEVLRGNTTVRRSLSNGQLGETVN

RAALVNPPTANLCFRFAARDVRLGNFLIRAGDIVSPSVAAAHRDLLKIGS

SHLVDSAVSTRAHLAWGAGQHQCPSAARELAGTIVTTAVGRIFEHFGRAE

LTLPPDQLPWRSGPVVRGLRLLPVRYELVGRGVGERPVGVPVVSPGGGGG

GVEVEVRRGESSGLMGLLRRMMSSRRKPG

>CYP179A(2523558726)Streptomyces sp. FxanaA7

VDPELHARLDELQRDPYPLYARARAAEGLTYVPELDSWLVARDADVREVL

RRPEDFSSANALRPDVMPAPAALAVLGGGFGGRPVVVTADGALHQELRAP

IVRGLSPARVAAVLPYAAERAAALVDDFVKNGEGGRVELMSAYAGRLPGE

VVGRLVGFDPDDVRALVHGGHRAEQLLFRPMPEAEQIAAAEDVVATAHRL

DAFVRARRADPRADLGTELITSVAGPGTGELTLDERHQVVAHLQNLLIAG

HLTTTALIGTTVLHLLRDRRQWELLCAEPERIPAAVEEAARYDTALQGFR

RVTTRPVTLAGTELPAGTPVFLAFAGANRDASRHPRPDDFDITRHAGSRH

LSFGLGTHTCPGSQLAREQLRLTLEQLTSRLPELRLAEGRRITMRPTLIH

RSPERLDLVW

>CYP154A(2523558861)Streptomyces sp. FxanaA7

MGHPSPLVIDPTGRDIHGEAARIRETGPATRVVLPGPPVVEAWAVSSPEL

LKRLLTDPRVSKDARQHWPRFAAGEITPEWPLFTWVAVQNMFTAYGGDHK

RLRTLIAKAFTARRTNALQPRIEQITEGLLDRIEEGLRGGATVDLREEFC

YPLPIQVITDLFGLPEERGAELRELVDKIFDTSADPGEMSAAFGRLQGVL

GELVATKRETPGDDLASGLISARDEDDARLSEQELIDTLVLMISAGHETT

VNLLDQAVHALLTHPEQLAHVQEGRATWDDVIEETLRVQAPVASLPLRYA

VEDLDLAEFGGPEGVVIARGEPILAAYAAAGRSPERHGKDADVFDVSRAD

KEHLAFGHGVHHCLGAPLGRLEARIALPALFTRFPNLRLASTGADLGHVE

SFISNGHRHLPVRTT

>CYP156C(2523558862)Streptomyces sp. FxanaA7

VTPAPPSRPTGPSDTPGPSGTFGPPPGCPAHQPMYGPEFAADPAAFYRRA

RATGPTAPVELAPGVRATLVTSYDAALHVLRGTETFSKDPRRWNDLADGT

VPPDSPVVPMMMYRPNALFSDGEEHRRLRGAITDSLARVEPHTLRGYVER

SADTLIDRFAPTGEADLLGEYAQVLPLLVFNHLFGCPAELGLKLVEGMSG

IFDGVDAERADALLGATLLELVTLKRKRPRADVTSWLTLHPAGLTDEEMV

HTLVVLMGAGTEPQQNLIAGGLRLLLSDDRFAGDLAGGSLPVEDALDEVL

WTDPPMANYAVHYPRRDVVYEGALLKAGAPLVVSLAAANTDPTLTTDQRT

GNRAHLAWSAGPHNCPARSEARLIASVAVEKLLDRLPDVDLAVPVDALEW

RPGPFHRALAALPVTFPPAPVTERPPSRGPTTPEARTRPDPTTRAPAPIH

PASPWTRLLSWWRGE

>CYP180B(2523559025)Streptomyces sp. FxanaA7

MTTSARPASVREAVQEPMPLDDVDLADLDNFTDGVTPWRMFHTLRHRDPV

HWQPEEAPNSGFWAVTRHADIARVDRDADTFTSTRFVNLEEVDEDQIRTR

ASILELDGVRHRALRSVIQRQFGASVINSYTDFLRGLTATTLDAALAKGT

FDFVADVSADFPINVLARLLDVPPEDNQKLIDWGNRIIGNTDPDYADVLL

SSAESEQYRHLPFRSPASLEVFEYGRELARQRRGGDGTDLVSRLVNTTPR

DGVPLSAQDFDNYFLLLVVAGNETTRHTITHSMLALLQHPEQLARLQEDP

SLIPVATEEFLRWASPVYHFRRTATRDVELGGKQVKEGDKVVMWYASGNR

DEEVFGNPYDFDVARADNDHVTFGKGSPHLCLGNLLARTEIRIMFEELIP

RLADIRLVGDVPRVRSNFVNGIKKLPVEVTLA

>CYP102B(2523559057)Streptomyces sp. FxanaA7

MTATTGPTAVRGFRSAELGWPELHRIPHPPYRIPVIGDALGTNVRTPVQE

SVRLGRRLGPIFRRKAFGKEVVFVGGAALAAEMADESRFAKHVGVGVANL

RPVAGDGLFTAYNHEPNWQLAHDVLAPGFSREAMAGYHPMMLDVAERLME

HWDRAGAAGSAVDVPGDMTKLTLETIARTGFGHDFGSFDRDRPHPFVAAM

VGTLTHAQRRNIVPDPLMPLLRGAARRNQADMAFLNETVDSVVRARQESG

RSHAGGRGDGDLLDRMLETAHPETGERLSAENVRRQVITFLVAGHETTSG

ALSFALHYLARHPDLAARARAEVDRVWGDAVRPGYEQVARLRYVRRVLDE

ALRLWPTAPAFSREARADTVLGGVHPMRRGAWALVLTAMLHRDPQVWGAD

ADRFDPDRFDAAAVRSRAPHTFKPFGTGARACIGRQFALHEATLVLGLLL

RRYELTQEPGYRLRVAERLTLMPEGLRLNVERRSRRPVTGAGD

>CYP107X(2523559414)Streptomyces sp. FxanaA7

MSTTTPEQPPLVDPAELVADPYAVYARLREAGPVHRITGTDGLPAWLVTR

YDDVRQALADPRLSLDKRNATPGGYHGLALPPALDANLLNMDPPDHTRIR

RLVSQAFTPRRVARLRDPIRRTADALLDAVAPHGRADLIASYAAPLPIAV

ICDLLGVAPHDRHDFRSWTDTLIAPDPARPERAKEAVRDMLGFFTRLIAG

KRAEPSDDLLSALIAVRDDEDRLSEDELMSLAFLILFAGYENTVHLIGNS

TLALLTHPGQLRALRADPGRLGGAIEELARYDGPVPLAIRRFPVEDVTLG

GVVVPAGETVLLSLAAAHRDPHRFADPDRLDVGRDASGHLALGHGIHYCL

GAPLARMETEIALAALLDRFPDLALDIAPDEVRWRPSMRARGLLDLPVRF

>CYP152D(2523559425)Streptomyces sp. FxanaA7

MKPSPRTPLADSSLAALVKGYTWLPDRRRRTAGPLVRARLTGRHTVALWG

PEAVRFFYDERHVERATALPGPVLSTLFGHGAVHTLDGPAHRVRKEMFLS

QLTGPKAVSDLVDHVGAAWDAAAESWPGRRSVVLFDEASRVLALGVCRWA

GISLDEASAADTARDMVAMVDGFATPGPRHWRARRARDRSEAWLEGLVRD

IREGAATAPPGSPLDTVVRHQDADGLPLDPHTAAVELLNIVRPTVAVCWF

VTYAGHALRLRPDVRERLAEDDPEYAVAFAHELRRFYPFAPFVGGLAVTD

LEWRGEPIPAGAMVLLDLYGQNHDPELWDLPYTFEPQRFLERPPQRDDLV

PQGGGDRATGHRCPGEDVTVALLRALGPRLARLTYDVPAQDLRIPLTRMP

ARVRSGFVMESVRVPTRVHAPG

>CYP182B(2523559545)Streptomyces sp. FxanaA7

MVRETQSGEGVSVELDGAGLEELSPEPLLTRDYETRPSLVYERLRQRHGA

VAPVDLLGVPAWLVLGYREALQVLQDDAGWPKGLENWRARTEGRVPADWP

LGPSLEVNHVLIQGGPGYRTLRTAWDQALKPFQDPRNPQAKRLKAAVTVY

ADELITLMGQAGGTGLADLSAQFSRPLPLMVASHLLGFPGSQGDDALMDM

WRVLDAGPDAGPALDRLLGALAELAELKLKTPGEDFPSYLLAAHPDLSLD

ELARELFMLLGMTSDHVGILVSNTVVEVLSGEDSVRASLSAGMVREAMNR

VVMRKPPLVNFVPRFAAEDTKLGEYTIRAGDPVWVSSAAAHADPLFADHV

APSTTVSTRAHLSWGAGPRQCPARELASTVAAVGVGRLFERFANLDLALP

VDQLPWRSSPFMRGLRSLPVRYELAPTAALPAPEPTQPDTDTTTQPVTPD

PSARQRSSLWRYLTDLIRPGR

>CYP268NSF(2523559597)Streptomyces sp. FxanaA7

MAIQSTETASRAYDPVSISPLSFWAGTWEEREKAFRTLRDERPVSWHPPL

EGALMPPENDGVWAVTRHEHIAEVSKNPEIYCSARGVMVEEIPEDILEAA

SSFLAMDAPRHFQLRRLVSSAFTPRQVARIHDQIRRQATTIVDDLLTTRE

GDFVSQVSKRLPMWTVYEMMGLPEDQRELAAHHADGMVSWADEDVAAGRE

PGEVLNEALVTLLQIGLEHAERRRAEPKDDLWTSLVQAEVDGRRLTDDEI

AAYFVLLSVAGNDTTRNTISVTARALQDFPDQKALLLEDFGGRIAPAVEE

FVRYVTPVQTFRRTATRDTVLGGQEIREGDWVLMMYASGNRDERVFKDPH

LLDILRPANNHLAFGGGGPHYCMGNFLAKMQLTEIFDQLLHRAPTLRVGE

PVYLAGNFVRAVKSMPYTL

>CYP125A(2523559793)Streptomyces sp. FxanaA7

MPCPALPDGFDFTDPDLLHHRVPLPEFAELRRTEPVHWIPQAPGLAGFAD

EGYWAVTRHADVKYVSTHPELFSSTVNTAIIRFNEHIERDAIDAQRLILL

NMDPPEHTRVRQIVQRVFTPRAIRALEDNLRHRALTIARQAVARSGPFDF

VTEIACELPLQAIAELIGIPQEDRIRIFEWSNRMISYDDPEYAITEEVGQ

QSAMELIAYAMNMAADRKQCPAKDIVTTLVAAEDEGNLASDEFGFFVLML

AVAGNETTRNAITHGMHAFLTHPDQWELYKRERPATAAEEIVRWAAPVNA

FQRTATQDVELGGKLVKEGDRVGIFYASANHDPDVFDHPDSFDITRDPNP

HLGFGGGGPHFCLGKSLAVLEIDLIFNAIADAMPGLRLAGEPDRLRSAWI

NGVKHLQVTT

>CYP107AE(2523560406)Streptomyces sp. FxanaA7

VTNPAPDPLQDPGFFTDPYPTYARLREAAPVSKVATGSGGRHSYVVTGYA

EAREAFTDPRLSKDTARFFAGRPSQRDLHPAVSRNMLATDPPEHARLRAL

VAKAFTTGAVARLRPYISGVVDELLDAWPDHGTVDLVAELAVPLPVTVIC

ELLGVPEPDRASVRTWSSDLFAAGDPRRIDAASHAIGDYMTALVATKRAA

PGNSLLDDLIAVRDGQDRLSEEELVSLAVLLLVAGHETTTNFIGNAALAL

LRHPESLARLRAEPRLIGDVLDELLRFDSPVGIATFRYTTEALTLGGTEI

PAGAPVLIAPGAANRDPDRFPDPDRLDLTRSATGHLAFGHGIHRCLGAPL

ARAEAELALHAIITRHPRASLATPAAALPWRHTRLTRGLSHLPLTL

>CYP107P(2523560468)Streptomyces sp. FxanaA7

MAAALDLVFDPWDPAFLADPYPAYAELRALGRVRYFEPTNQWLVPHHADV

SALLRDRRLGRTYQHRFTHEDFGRTAPPAEHEPFHTLNDHGMLDLEPPDH

TRIRRLVSKAFTPRTVEQLKPYVRELAGELVAGLVEAGGGDLLADVAEPL

PVAVIAEMLGIPESDRAQLRPWSADICGMYELSPSEETAKRAVRASVEFS

EYLRELIAHRRAEPGEDLISGLIAAYDEGDRLTEQEMISTCVLLLNAGHE

ATVNATVNGWWALFRNPEQLAALRADRSLVPTAVEELMRYDTPLQLFERW

VLDDIEIDGTTVPRGAEIAMLFGSANHDPAVFAEPEKLDLTRRENPHISF

SAGIHYCIGAPLARIELAASMTALLEQAPTLTLATEPTRKPNFVIRGLDA

LAVEI

>CYP105Q(2523561194)Streptomyces sp. FxanaA7

MADTLTDAVSETGGPIPDYPMPRASGCPLAPPPAAGELRGDRPITKVRIW

NGTTPWLITRHADQRTLLTDPRVSNDDHEPDFPHVNAHRAVIAPHTPKLI

TNTDAPEHTRLRRSVNAPFLVKRIEAMRPAVQKIVDDLIDAMLAGPHPAD

LLTALALPVPSLVIADLLGVPYKDHEFFQENSNRALDSALTAEEASAAAR

NLGGYLDALFREKLVEPGDDVLSEMAGRVKSGEMAHEEAVSMGVAMLIAG

HETTATMISLGTLALFEHPDQLAVLRDTEDPKVVAGAVDELLRYLSIVHS

GLRRVAKDDIEIGGQIIRKGDGLLFDLQTANWDPSAFPEAERLDLSRPAR

QHNAFGYGPHQCLGQNLARLELQVVYGTLYRRVPTLGPAAPLDQLAFNHT

GTTYGVKCLPVTW

>CYP107MT1(2523561681)Streptomyces sp. FxanaA7

MDVAANIHRFPMAEPVGFAYPPCYRELQENEPLARVRMAYGMEARLITRH

ADVRAVLADPRFSRAATAGVDIPRSSPVMLHDASIVTMDPPEHTRLRRMV

AQAFTKRGVEAMRPRVVESVARLLDAMEEQGPPADLVTALAQPLPILAIC

ALFGVVDDRSDTFRELAHTMFTGTEQTAVESAFGEIMAMITALVAERRRT

ASEDLLGRLVSACDDDDRLSEEELVGLALALLVGGFENSAQEIANCAALV

LRSPDHLAELRARPDLVPSAVEEVLRFAPLSVTGGLVRIAVDDVELGSGT

IRKGEAVIIDTQSANLDPAVFDDPTEMDIHRSPNPHLIFGHGAHRCIGAE

LARMELQVSLGSLLQRFPRLALAVPAEDIPFTGGMLRGPACLPVTW

>CYP105EG1(2523561693)Streptomyces sp. FxanaA7

MPLDIEIPLPYQRDAVCPFDPGPRLREAQRNDPVHRWRLPDGRHIWVVTR

YDDVRQMFTDPRLSSALTPLTLMLPGLDEDQLQVAPGSFVNMDPPEHTRL

RRTVASSFTARRMRELTPRLEQLAEDCLDGMERMGAPADFMTAFAFPFPL

FAICELLGLSEEERAEYLALTAKAAVGLAGTAEEVKEVVDAAHRFMLDAV

TTRRAQPDDGMIGMLVREHGDDLADEEIVGISNMFLSAGFDTTANTLGLG

LLALFDNPGQLALLRDDPGVLDTAVEELMRYLSVISATSGRTATEDLEIG

GVTVKAGEYVVPALAVANRDPDHYADPDRLDLTRTPAQQLAFGHGIHRCT

GAAMTLLELKIAFPAVLRRFPGLRLAVPREELTYRGYNMVHGALSIPVAW

>CYP105DS1(2523561700)Streptomyces sp. FxanaA7

VTVTPEREDIAFPLLRKCPYTPPDEYVKFRETEAPTPGQLYNGRRVWLVT

RYEQVRAILGDERFSSDITNPGYPIYAEAFEGSRGFPMLFTMDPPQHTVQ

RKAVIREFTLRRAEDLRPQMQRKADELIDAMLAEGNSADLVEKFAGPFSG

IITCWALGMEYSDMQAWLAGNREVREKAHAVLDTEQVGAEVSMRMMALQE

YFLRFIEAKEEEPGDDPVSRVIAQHVTTGTLSRTELANLCFLIFIAGQTP

VQAMLTVGVGLLLERPDQLALVRDDPKVLPGTVDEMMRMVSPLDLMARVA

LEDVEVGGQLIRAGEGLLVANGGANHDPAEFPDPGRLDVRRAARGHLAFG

SGIHHCLGANLTRVGLEVAYGTLLRRLPGLRLAVPMEEMYDRPLWHPELQ

RMPVTW

>CYP107JB2(2523561890)Streptomyces sp. FxanaA7

MTAPAPPTELFGYEYYQNPYPTLDWLRENSPMHRFRFPVGDIPLWIATRF

DDVQQLLGDPRFSTNPAWASKEFVDGGMAVGKGTAIERIITMLDPPDHTR

VRRLAMSTFTPRRIAQWEEPTHAVVEAELDRLEKQDQPDIMDYAGAIPAA

LMGNILGFPLDRFNEMIHAIERAFHTDPEDEDANRRAFEEIAEYGRGLIA

HKRRAPADDLASALIEARDGEDRLDETELVAMIAVLIMAGLDTTRNLIGT

SILSLLERPDQRRLLLERPELKDTAVEEFLRFNGGVAVGFFRFAKEDLEF

AGVSLPAGAPVIPAVGSANRDPRRWPDADRLDITRTGPRHVGLGHGLHNC

LGAALARLEAAAAIPAIMRRFPEMSLAVPAEELRYDEMWLVRSITSLPVH

LHGDGKR

>CYP156B(2523562066)Streptomyces sp. FxanaA7

MTPDSPLPSPPPGCPAHGSGLRIPLYGPEYAADPQAYYAYMRHYGPSAPV

EIAPGVDATLVTDHATALKLLQDPGNFRKDARRWRDVAEGKVGPDSPVVP

MLGYRPNAMFTDGAEHARLRQAITDSLAKVDSRRISDMTKRASDYLLAQV

SGRGSIDLMNDYVKQLPLLVFNELFGCPADIGDRVVFGISGVFEGVNAEK

ANQVLGQAVFELVALKRARPADDVTSYLMRHEARLTDEELVHQLILLLGA

GAEPLRNLIGNTLHRLLLHDRYADGGLIEEAIDDTLWENPPMANYAPHYP

AADMEFGGAKLRAGDLVLVSMTAANTDPALVASGQTGGRRAHLSWSAGPH

ACPSKELARLVTMVAIENLLNRLHDIELAVPEDSLTWRPGPFHRALAALP

CRFTPQVVQRPMPPRVAEPSAGEENAPAGRKAERGVWGSFLQWLKG

>CYP154A(2523563441)Streptomyces sp. FxanaA7

VHTPPVPLVLDPSGQDLHGEARRLRQLGPVAPVVLPGGVTAWAVNRYDLI

KQVLMDPRVSKDAYRHWPKWISGEVNDSWPLAMWVSVRSMLTAYGEEHTR

LRKLVAGAFTARRVADLQPRISEITDELLTRLEKVPPGHTVDLRREFAYE

LPIRVISELLGLEGRESHDFHRVVNVLFTTSTDPVQAQENQAELYALLGD

LVGRKRAEPAADLTSALIATRDEDGEGLSEKELVDTLHLILGAGHETTVN

LLDHTICALLTHPEQLRLVREGTVGWEDVVHETLRSQAPVANIPLRFAVE

DIDLDGTVIPAGDPMVLSLVTAGRDPGTHGDDANTFDVTRPTRRDHLAFG

HGVHHCLGRLLAMSEATTALPALFERFPDMRPAVPADELGLLESFISSGH

TELPVLLTPAF

>CYP1005B(2523563471)Streptomyces sp. FxanaA7

VRQVLLDPDVFLPDNAQHSVTPLPVAVLRVLARGGFSLPPALANNGSPTH

AGLRRVVTRFFHANRVADAVPVIERIADELLVDEVRSRIDSTGDCDLFTS

FAQLLPCRVLMELLGIRGVEPATLIRWSDASLELFWGRPTPERQLELAKL

VVDFHQWLTATVRGGMAASDSFIGALARHRLPDGGPLDVETAVGVCFFVF

IAGQSTTGQLITTVLRRAMAESGMWLRVAGEAGLAEAWVEEVLRREPPVT

TWRRVTAQSTELSGVRLPAGAQVLVMLLGSGSDPEVFPSPERMCPHRENI

RHHLAFGAGRHRCPGASLARTEAAVALRAAARRLPDIRPAAGAGEPPMLG

LLSFRAPLKMVVE

>CYP105DH3(2519021981)Streptomyces sulphureus DSM 40104

VLNLDPEEVAGLPRYPVGRRCPYEIPEIYTRLRAEEPVSKVVMSDGQPVW

FLSRYEDVRAVLSDPRFSADRLADGFPNLALGQREGLSKQPKFMISMDGA

EHSAARRRVISDFSVRRVAELTPMVQKLVDDCIDRILDLPQPVDLVAELA

LPVPTLLLAELVGADHADHAYFIDLVHRMLWRKTSGEERVQISIGLRKYF

DDLIAEKEANPGDDIISRQIAFQREENGEVDREGLNSLAQLLLIAGYESS

ASMIALGVHTFLTRPEWLETIRKDPAKTPVAVEELLRFYSILDVAAGRVA

LADVEIGGVTIRTGDGVLASVFAANRDPAAFPDPDRLNLERGARHHVAFG

YGPHQCLGQNLSRLELQIVFDTLFERIPTLRPAVDEADLPFKYDALAFGL

YELPVVW

>CYP113AC1(2519022430)Streptomyces sulphureus DSM 40104

MYEPQTAPPSSTAEGGRVLRDWLAENRRKNPVRWDEESGSCEVFGFAETQ

QMLRDPETFSSDFARLVPPQETPPGQRPVPNLLEGHLSVTDPPRHHKLRK

LVSQALSPRAVLELEGAVVQITADLLDAMEGRDEVEMVSSMFNPLPVTVI

ARLLGVPASDHARFRAWADALVSANHDAGAVDSSRLQVPATMDALLLEME

SYILDHAAHRRRHPSDDVLSRLVAAEVDGERLSETEVFTIALMLLLAGHI

STTLLLGNSLLCLDADPAALAAVRSDPDAVPGAVEEVLRYSPPAPLMYRL

TSRATSLGGVDVPADTMVASWVLSANHDERQFSEPERFDITRTPNPHLSF

GHGIHFCIGAPLGRMEARIALSMLLERCKELNFAETPVYYDSPTIFGVRR

LVLDSPVG

>CYP204D1(2519023150)Streptomyces sulphureus DSM 40104

MPHAVRPPQLSGARPFVGHAAPFLRDPYAVLSRGQAEHGRAFSFRLGNRQ

ATALLGSEYSGWMLKAPDDDLSIRASYPFFKHMFGPDFFFWAEFEEYRRQ

RELVLPRFRAGQLDSYLGRMETEARALMARLGGEGEMELVETLGETVMHI

SAHAFLGADVNERIRGFFPTFRRFSEGLDPVLPGWVPAPHLLRSRRARDS

LRRDVLALVRRRRQHPWQEPDFLQTLVDSHFEDGEPVPDAVLVNLILMFL

WAGHETTTGHLAWALVDLLEHPAHLARVRAEQRDAGLGEGASAPLTTKDL

HRLPLLAAALKETQRLHPVAPMLMRRARRELTVDGWLIPHGSLVLVSPAL

THRLPEEWRQPDAYRPERFLKGSGERTPELIGFGGGLHRCLGERFAHLEM

EAVLTLLLRHYELELLDSPVLPVPGSTTKWPKSPCRVRYRAKSAAGAAV

>CYP107MD1(2519023663)Streptomyces sulphureus DSM 40104

VSESLSETPGLPLSRSCPFDPPDGYKRLREEEPVSRVRFADGNEGWLLTK

FDDVRTFLLDTRFSSNRNRAARTRHTHEKPKLPAGAMVSMDPPEHTRYRR

LLAGQFTVRRVRQLEPRIVEYVNGQLDEMEAAGPGVDLVETFALPVPSLV

ICDLLGVPTRDRHDFQKWAQTVLNVDLPLDEVHRAKEKLFDFITALVHEK

AEHPQDDIISGLLHAPPEEQLTREEIIGISVLLLIAGHESTSNMLSIGAY

ALLRHPRELAKLKADPSLIDNAVEELLRYLSIVHLEFIRTATEEIEFAGH

LIKPGETVKGSMVSANRDPDRYPEPDVLDLNRKDVHHLTFGHGIHQCIAQ

QLARVELRVSFSLLFSRFPTLRPAIAPEDVRFKDDSVAYGVKELPVAWDV

RKP

>CYP291D1(2519023964)Streptomyces sulphureus DSM 40104

MPQAAIPGLGPDLTDPMFFAEDPHPTYARLRAEDPVHWSEQGRFWALSKY

DDVRAAGKNTKLFSSTRGTLISDAHSRDTNGPHMAGATHLIRSDAPTHTE

LRKLIAGAFTPKAVARMREKARQIVRDLIAEIDGAGVHNAVEAISAPATT

YVIAELLGVPRDRWSDFWRWTNSALLQTDARNEPEHAANIAELLDFFREL

CAQRRKDPQDDVVSSLVHAEFRGEPLSEVNLLTFCKLLLGAGTETTRGLI

TGGIQLLSDHPEQRERLASEPELMESAVEEMLRLVSPVVAFARTATAETE

VRGRRIARGDYVVLLYQSANRDEEVWADPDAFDITRPIKRNLAFGFGPHI

CIGAPLARMEAAVIFGELLAAYPHFDVAGPPVKRGSTLVSMVLDLPVAFG

GR

>CYP105DY1(2519023968)Streptomyces sulphureus DSM 40104

MTIGTENVEAPSFPLQRGCPFAPPEAATAFREEGAPHRVTIWDGSRPWLL

TRHDHIQQVLGARGTFSADVTDPGFPNTSAAQPTVEGDIFFRKDGDEHLP

IRRILNPDFTAKRSEALRPRIRELTDQLLGELLASKTKPVDLVEAYALAL

PTVVICEVLGVPYEESPVIHESSKKLVQLGLPTEEKLAAHTAMHENLSRN

MERKRSAPDDGLLSRLVNTHVDEKGELDFEDAVRLGALVIGAGHETTANM

IGLGVLAFLRDRPQRDLLLSDPEQYAAPAVEELMRYWSMVQTEPRRVCVE

DVEVGGVTIRAGEGIICNLPAANRDPEVFTAPEELDITRTERRHMGFGFG

IHQCLGQNLSRVEMQEAWPRLFQRIPTLQLAVDESALRFHTEALTYGLEE

LPVTW

>CYP105A(2519023971)Streptomyces sulphureus DSM 40104

VTSSTLPSMPVERTTLFDPPQEYTALREEHPVTKVRFPNGSTGWLVSRFE

EGSEVFAHPKMSARRPRHDTPEGEVAEAGNDAPFNPTFVMMDEPDHSQYR

RLLSGRFTPKSIAKNLQPYLDRIVDEHLDAIEKGPKVFDFVEALSLPIPC

LVICELLGVPYADRDGFHSATEQMMDLANSREERDKGAHWLIDYITRLVA

EKRRTGVQDGILAELIATADDEGSFLKDDEVIGMGVLLLFAGHDTTASML

GLSTLTLLTHPEQRRDLEEHPEKIGPAVEELLRYLTIVQFGLGRVALEDV

EIGGQQIAAGDLVVVAMPAANRDPRAFDAPDAPDFDRKMTRHLAFGYGVH

QCLGQNVARAELKTVLPRLFERFPNLHLAVAPEEVPMDSLGTNYAVKKLM

LRR

>CYP157A(2519024106)Streptomyces sulphureus DSM 40104

VSNAPAHGGPEAPAGCPAHSEPVELGGPRFQEDDRAGLYRELRRVHGPIA

PVELPGGVPAWLVLGYRELHRITEDPGLFSRDSALWNQWDDIPEDWPLLP

MVGKQESILYTIGERHARRARLLGEALDAVDPFELRRHAERFADQLIDRF

CGRGDADLVAEYAKVLPALVLARLYGFEDTAGFELVPSVNALVDGDAGAL

EGRDRVHGAVARLLSARRASPGADVSSRMVGHPLSDEFSEAEVLEDMMVN

IVAGHQPTADWMGNSLRLMLTDERFAASLGGGRHSVGEAMNEVLWEDTPT

QNIAGRWATRDTSLAERRIRRGDLLILSFAAANGDPQVRPDQHSFTGGNN

AYFSFGHGDHRCPYPAQDIAEGIARTGIEVLLDRLPDVDLAVGAEELQWR

SSPWLRGLGSLPVQFTPTTAVGGTP

>CYP154C(2519024107)Streptomyces sulphureus DSM 40104

VTQCPVTGAGTQPSETVVLDPLVQDLAGEGARLREAGPVARVELPGGVPC

WAVTHHAEARQLLTDARLVKDIGKWGAWRRGEISPSWPLIGLAAPGPSML

TYDGAEHRRLRTLTAQALTPRRVEAMRPVIEEITQRLLDELAESADERGA

ADLKATFAYELPMSVIAELLGVATNDEPRLRKLYEGFFSSVTPPEEVQQV

MTELAEFFTGVLEEKKRNPGDDLTTALLQAAEEGDTLTDEEVLATIQVLV

AAGHETTISLIVNAVRALLTHPEQLSAVLAGDVSWDSVIEEVLRWDAPTT

HVLIRFATEDVEVGDTVIGEGDALIIAYGAIGRDEAQHGSDAAEFVSTRS

PNRHISFGYGPHVCPGAPLSRLEARVALPALFARFPDMVLGVPASELRNK

PAVTQNELQALPVLLHGAKEGLATA

>CYP155A(2519024156)Streptomyces sulphureus DSM 40104

MSVERTRTRKTGPVPPPATGCPVGKGADGVWEVRGHETARALLRSSGTLQ

AGLGIETARIIPSSVRRPVLYQDGEGHRDHRRQTARFFTPRRVDEEYRGT

MERVAEEQLDVLRTRRTAELSDLSFELAIGVAAAVIGLTESRPGIRKRLD

AFFPQQFGTPGFTSLHGLRWSGRLVKNWLRLHLADVRPAVRARRKHRRDD

LISHLLDEEYNTVEILSECLTFAAAGMITTREFVNVAAWHLFDDPELLAR

YRRADEPERLAVLHEILRIEPVVNLLRRRTTAPVALPGEEGEVTVPAGTL

VDIRLDSANTDPRAVGDAPHDLCPARPLPSGTASTGLSFGDGPHRCPGRD

IAILETDVFLTRLFALPGLRMSSTPDVGFKSSIGSYELRGLVVEVEGT

>CYP107CS(2519024422)Streptomyces sulphureus DSM 40104

MTETARSEPARAGEPQPDGVEYDLSDPAFVTDPAAADRWLSAPRAACPGR

SLDGSPVLVVTRYETAREVLSDPRFTSRPPGDSHLRGLLRQGVPEDLAPL

MSSTLLSMDGPDHDRVRPLVNAAFSAKRIRSLRPRIDDLVCGLLDGLDAE

REVDLLAELADPLPLSVIGELLGVGEEDRRRWLASARTFSGANPATPEET

GPALRGIAAVLTELIAKRRAEPGDDLLSELVRKRDEDETRISERELLALA

MLVIQAGHDSVRQFISQSACLMLARPERAERLRSDPSLWRTALPEVMRHA

TPVKHAFRRFATEPVEVEGQTVGAGEGVLVVLAAANRDQGQFPAAGTLDL

ERTPNQHLGFSHGPHFCPGSSLALAQAEIALSRLFERFPAIRLAAPAEEL

APRFLIGMQRLPVHLR

>CYP105DM1(2519024427)Streptomyces sulphureus DSM 40104

MTDALPIPGAQPRRCPFDPPDDYRRAQQEGGPVMVTMQQGRAGRLLTRYQ

EVRKALKDGRFSSSLHVLPPVIEGRSPRGWIFGMDPPEHTRYRRLLAEMF

SERRMRRFEPRLVRIVTGRLEELKEQGSPADLMNRYAWPISSRVSCDLLG

TTLDDQEEFERRVGRLVAPDVTPEAFVATYQSMWYGMRELVRAKRGEPGD

DMLTELLGAEDAQGPLSDEEAASIGLQLRIAGKDLVAHAIGLGIFALLRH

PDQLALVRDAPDRLDKAVDELLRYLPINNMGSVRKAAAEAEAGEQEFAEG

ELVVAALTAANRDERVFTDPDRLDLTRPNARQHVAFGHGAHRCLGQHQAR

LMITVALRELLRALPRMELAVPADEVPLYEDTAFYGVAELPVAW

>CYP107L58(2519024965)Streptomyces sulphureus DSM 40104

LPVDLHAHGDAFVRDPHPLFSSLLAGGQAHRVRFTEGFDGWLIVGHEASR

NALNDPRLINDPRRVPETDGPGPGGARRFDDNLLSLDAPDHTRLRRLVAK

EFTARRVRGLEPRVQEIVDGLVGTLAEGPRERVDLVEALSFPLPITVIAE

LLGVPFLERETFRHLSSGVLESTNQEEAAAMSEDLHAFLARLVDAKRAAG

GEDLLGALLEAASREGEEDSLITGAELLGMANLLLVAGYETTVNLISSTV

LCLLQHPEQLAQVRADPSLVAAAVEETLRFEGPVARSIPRFATEPIPFDD

GTVIPAGEVVCIGLASAGRDPERFPHPDEFDIHRDTGGHLAFGHGAHFCL

GAPLARLEARTAVTTLLARIPALALDVPASELSWRTGTLLRGPRQLPVRL

G

>CYP156B(2519025644)Streptomyces sulphureus DSM 40104

MHHPDIEGTPSRCPVSGNVELYGPEFGADPESHYAHLRSLGPSAPVDIAD

GVEVELVTSYDAALSILHDPATFVRDSRRWNALNEGRVPQDSPALPMMGY

RPNALFSDGAAHARLRQAVTDSLGAVDELQLVRQTKQSAEYLISQFSSDR

HGRAELMNEYAHQLPLLVFSDLFGCPPEIGDRVIVGISGIFDGTPEADQI

LAEALSELIALKHRRPGNDVTTRLMQHSAQLTDEEVLHQLVTLLSGGTTP

LAAAIGTSSALILDESWQQGLPVETAVSQALWNYAPIANYAAHYPTRDVE

LGERRIRANDPVVISFAAANTDPRLAQHREQLSSKAHIAFGAGPHACPAK

DPAFLVAVTAVECLLNRLPDVEVCAPFETLEWAPVPWSRVLLELPVSFTP

SLPQPGPAAAEQQPSAPVPQQVHVPAQRQESGKAERKGGLFSRFLAWTRG

E

>CYP1035A(2519025645)Streptomyces sulphureus DSM 40104

MCQIRVGYAWRLMTGRGSSAMSLTTLQSPDRRTTVSLFSRLRTAKGQADP

FPLYEELRSGGHVTPAPWGGNLVTGYDLCDQVVRSREWVEPDRQWRARQG

ESTRWGAPSVREMAHTLPALNPPEHTRARRAAGSFDRGSIQEMTKNVTTT

TSRLLDTLTLQLRDGEADFNTLVSEELPVAVIGGWLGLPSADWPRLRELT

HDQVFAQELLPSASQLALSDAATAELRTYFTELVRERRARPGDDPVSRWI

AAWDTMEPDREKADAGVYYLAFFVLLAALETTTSLLSMATLQLLDRPRWW

EALAADPDLAPAFVEESLRYDAPTHVITRVATRDGELGGAEYRTGEMVHL

MVGAAGRDPERHRRAEEFDPHRKPAHLAFSGGIHYCLGAPVARMEAQVLL

RELVRRLPRLTLARRPRWAPRVAFRRLLNLDVVTA

>CYP1265A1(2519025958)Streptomyces sulphureus DSM 40104

MTRQAVVPAADDTNTADAAFASILFSPEGNQNPYPYYHTLRESSPVHQSI

LGMWVVTRYDDVLSTLRDKTIGKNVERFMQGRFGGDWWSHAALRRMGTSL

LWANPPMHTRLRRLANQSFTAAVVAAQRERIVRLLDDLLDPIAEAGGGDI

VNDVAYPLPLSVVATLLGMPLEDAPLMREPMRNFQRTFELGLTAQELAAA

DVGAQFSDDYFTEMIERKRREPGDDLASSLIQVSDADGEALSPIELVGVC

NMIIGAGFETTTHTLGNGLRALLKNPDQMSLLRARPELLEGAVEEVLRYD

APVQMAPRMLDEPRVIGGVEIPAPAQLMLAIGAANYDPTHFTEPDEFRIT

RDESPAISFGAGIHTCLGWRLAKLQAEVFYSTLLRRFPQMELAVEPVYRP

RATLRGLESLHISVTNS

>CYP135E8(2519026224)Streptomyces sulphureus DSM 40104

VTPQVWSTVQAKGSVMTSEPTAREAARIRRLPLPPGPRLPRVVQTALYRR

VPYRLTPKLRGTYGDIVLLRLFPEGTVVQLADAQHIQALLKGSDAVFHGG

EGNAIVEPILGSGSLMLADEAEHRRARRLLMPAFRTAALRGYEDMVGRLA

AREAEQWRPGVAFRAHDRMNGLTLEIIMQVVFGVAEGAHLGELRHLMGEF

VKVKPLDFLGWHMPALQKVGRWRRKLAQQRRLDELIQALIAERRAAKDTA

ERHDVLSQLLSVPAESDALTDAELRDQLVSLLLAGHETTATALAWSLHAL

AWDSALQEKAAAAAVSDDRKYLEAIVKEALRLHPVISEIARKVTQEVEIG

GFRVPARAVVMPMIGQVHTDAEHHEDPGAFRPERFLDGRVPRGSWLPFGG

GPRYCLGAGFALMESALVLREILTRYRLSPCGDRPEEPRARHVILVPARG

CKITTTRRG

>CYP107FF2(2519026230)Streptomyces sulphureus DSM 40104

MSLPAERLLLDNDFMHDPHTAAAALREKGPVLPVVLPTGLPVWLVTGHEQ

ARTLLMDARLSSHGVYDRLERLRELSDGKPSTFSSDLARHLLNVDPPDHT

RLRKLVNKAFTPRTIAGLRPRIVQIADELIAEFRTEGPVELLEAYAYPLP

IRVICELLGIPADDRESFSTWSSVLVSAASPEEIGRASASMAQYLTELLE

RKKNAPTDGLLSALLEISEDGDRLTVSELVAMTFVLLIGGYETTVSLIGS

GLLALLRNPDQLAALRADPGLLPDAVEECLRYETPNNLSSPRYTTAPVDI

DGVTIPAGEFVMVSLLGVNRDGSQFDDPDRFDITRSAGGHLGFGHGIHFC

VGAPLGRLEGEVALGRLLASYSRIELAVPAGKLKWRASTAMHGLESLPVL

LD

>CYP154A(2519026510)Streptomyces sulphureus DSM 40104

VAWSVGRHALLKRLLGDKRVSKDPRQHWTAFRNGEVPADWSLMSWVGVEN

MFTAYGSDHRRLRTLVSQAFTPRRTERLAPRIEEITARLLDDLDAAQPGP

VDLREGFAYPLPITVICELYGVPEHDREPLRTSVDALFATSTSSEEAQGH

MATLMRVMSDLAAAKRETPGEDLTTALLAARDGEGSRLSEQELLDTLFLM

IGAGHETTVNLLHHAIRNLAENPGELARIRSGERTWDDAIEESLRHQPPI

ANLPLRYAVEDIELDDGTVIPAGEAILANYAGAGRDPEHHGPDADTFDLD

RPTRTDHLSFGHGVHFCVGAPLARLEGRIALPALFDRFPDLALTDDPRNE

AATESFIANARQTLMVRLDAA

>CYP156T1(2519026511)Streptomyces sulphureus DSM 40104

VTQQDAPAVPGAGPDDQAPLLLYGEEFTQDPAPLYARARETAPIVRARVH

EDVEVSLVVRYHTALKVLHHPEMSKNSGPWFEARERQGLPMPPVLEMMRV

RPNALYEDGEEHRRLSTAVRDSLGRLDPPILRRTARETADRLVDTFAERG

EADLMGEYAGVLPLQMFFAMFGVPDDLGLRIQQLTATVFDGGPEAAQANV

ELSATWQELVARKRREPGEDVASWLLAHPSSNDDDSEIINQLLLVMGAGT

EPEQNLIASTLLQLLSDDSSYGRLLSGSQRIAEALETTLLDYAPMANYAT

HYPVRDLEIDGILLPAWEPVMVSFGSCNQDEALRGDATGGTGDSGYRAHL

AWSAGPHECPAWSVAMMMASVAVEVLLDRLPDIELATPRPQLSWRPGPYQ

RGLSALPVVFPPQPVRPQDATADPYGSAGGHAPWQPSTTATAPSTPQRTP

SPYDSTRSEATSTARQQPSAPRGLRRVWNSLAEWWRGR

>CYP107AM(2519026879)Streptomyces sulphureus DSM 40104

VTDAQAPVPYPLKARTPLQPAEEWAELREKCPVARVVLPSGDEAALLTRY

EDVRTALSDPRFSREGLARPDAARIAAGDSEGIFSSPMARTLNAEGHERW

RRMVGKWFTAKRMTALRPDMEAMADRLVDGMVERGSPADIVADLAFPLPV

YVICTMLGVPEADRDRFKTWSDTFLTMTRYTKDETAAAHREFAAYMAGLV

GGKRAEPGDDLLSLLMSGADGEGEPMTEEALVATGQALLLAGHETTAGFI

GLMLAHLLSDRSRWERLLADPSLTRLAVEESLRFDPNGANFGMLRYADEE

AELAGGTVPAGATVVCSMASANRDEDAWEYASEMDLGRSPNRHLTFGAGA

HSCLGQPLARTEMQAVLSVLLRRLPGLELAVDAAELRAHEGLLTAPLREL

PVTWRR

>CYP105B(2519026883)Streptomyces sulphureus DSM 40104

LTLAPDAYDDLPEFPNPRAASCPFGVSPPMRGLFDRGPLMRVASWGASTP

WVVTRHAEQRALLADPRLSADFSHPGFPSPVDPEKARGGHSDMSFVGMDD

PEHARLRRMVSGAFTVKRVQAMRPAVQRMTDEFLDAMLAGPKPADLVQQL

ALPIPSLVISHLLGVPYEDHDFFQANSKTIVSSVASAEARQTAHGNLSGY

LDDLVGRKLTEPGDDLLSQLAERVGKGELARREAAAMGVLLLLGGHETTA

NMITLGTLLLLEHPEQLAAVRDTEDPKVVEAAVEELLRYLSIVHLGRRRT

ALEDIEVGGETIRAGEGVILLSELANRDPAVFPEPDTLDITRNARPHLAF

GAGTHHCVGQPLARLELQVVYPTLFRRVPTLRAAVAVEDIPFKYDAVIYG

VHKLPVTW

>CYP107AM(2519026885)Streptomyces sulphureus DSM 40104

MSQHEQTLRYPLSQEDALAAPAEWERLRSECPVAAVELPSGDRATLLTRY

EHVKQLLSDPRFVRPTSADGAARVAPGGVGGAAATGDSTAVPVRGEEHQK

WRRMVGKWFTARRMSALRPSIAEIAETLIDDMVEQGEPADLKAALGFPLP

VYVICDMLGVPAEDRARFSHWSDAMLSLTRFSQDEIDTAHREFVSYMGDH

IAAKRAAPADDILSMLITESAEEGGGLTDSTLLGTGMGLLVAGHETTANM

IGKMVAMLLAERARWERLVADPTLVRTTVEESLRFDANLGFGMRRYVSED

VEVGGEMLPAGTTAICSMQAANRDETVFATADDMDLGRSPNPHLTFGAGP

HSCLGQALARTELQVTLEVLVRRLPSLELAAPVSELRRVEEMLVGGLREV

PVRW

>CYP157J(2519026938)Streptomyces sulphureus DSM 40104

MTRSFVRTADATVSLTELTQTPDPEPLYRSLRSEYGSVAPVVLEPGINAW

LVMGVQELRSVTERQKLFSRDARNWRDLTAGTVPPDSGLLPMMGYRDTVV

GKDLAEHQRLRRPLDDAVRGIAPRLLRRQVRERCDLLIDAFSGRGSADLV

TEYAVHAPMQAFSVLFGLDVAQGHELNSALLALFGSQPDSQAGNRDFEQI

LREHLRDREKVPRTDRPDDLTSAFLAHPGLHSEDEQLQAMVMMIAGGNEP

VTCWIAHTLLRVITDPSFGARLRGGDLGVNEALDQVLWAEPPMTNMPARY

ALRDTDLGGHRIRRGDALILGFHATHATDPTLPPDPRPAGNRGHFAYSAG

AHMCPAQDPAWTITHTAVTTALHRLAAVRLSISRDEVTFNPSPWTRRPSN

LPVTFTVPMVRTPKSEDEEGDEEPMADAKTG

>CYP107U(2519027329)Streptomyces sulphureus DSM 40104

VHDETPPIANPARPDGRPAPTRPPGDRHGPARPPLPEETRSTGTRRPGVH

GEEPADRASASGASTGPTNGTRTVPGLRAAAPGHEDGSGGARTPGAREAQ

AGAACNAPSSDGSCAQGDEETAGASVFGAVSEGSAVSGTHTAPGGAARNF

ARSSGGWSDGEEWADDVSASEGHDVDAEHARLSDEPDGASAPGPVPAGGA

VSGARTAPPSDEHAGERPTIPDPAQAPETRSTRPPGPDLPLVEARTEAAP

ASGAATAGAFNAPPPTARCPHAGDRTSAPSPRPDPSDAPPDGPPAPPPMP

PLFDWRFASDPYPAYAWLREHAPVHRTTLPSGVEAWLVTRHTDARAALAE

QRLSKNPGRHSESAHDKGKVGIPGERGANLMTHLLNIDPPDHTRLRRLVS

QAFTPRRVAAFAPRVQALADELIDAILARREPGAPVGEADLIHEFAFPLP

IYAICDLLGVPAQDQDDFRDWAGMMLRHGNAPRGGVGRAVKKMRRYLAEL

IHRKRELLPPQPAADEDLISGLIRASDHGEHLTENEAAAMAFILLFAGFE

TTVNLIGNGTLALLRHPEQRALLQGAVERGGPQDEALLARAVEELLRFDG

PVEMATWRFATEPLVLGGARIAEGDPVLVVLAAADRDPERFESPDALDLT

RTDNQHLGYGHGIHYCLGAPLARLEGRTALATLFRRLPALELATPPDELR

WRGGLIMRGLRTLPVTYRADPD

>CYP283A(2519027399)Streptomyces sulphureus DSM 40104

MQADPSTGTTDPEAPPGVCPVTGRRADPVDFEFFNGPSAYREAAATHADE

GAFYSASGDGFWVLTTYEGICEAFRDEEQFTVGRVSAADGAEEDRWIPLT

IEGRQHTEWRRRLGAWFTPQRVRELTPSIEANARRRIEGFVDDGEVSFNE

DFARPFVLENLMTAVGWEFDRFDQLLRIDRAMIDSRYAPDPREAAFEEVG

IPALEKLAREQVERRRAEPADDLTTATFGWEVEGRPVTDDDRVSLLCVLF

LAGVDSTVNHLANAVQHLVCHDADKRRFLASREVRPRAVEEFLRANSCMY

PGRLAARDGAGGTARRGETVLLPLAVANYDPEVFHDPEQVDFDRERNPHI

AFGTGAHQCLGAALARAQIRTALDVWHELIPHYGFPPQPEPSEPRFLRNA

YDLRLVW

>CYP105AK(2515555204)Streptomyces sp. MspMP-M5

MTTLRSRIFAWAGRLYLARTRKKGFDLSRMSFLPDSVLMPLRRDGLDPVP

DLAAVRERAPISRLPVPIAANVWLVTGYDEAKAVLGKADAFSSDFTNLVG

KAGAGAEQNPGGLGFADPPVHTRLRRLLTPEFTMRRLNRLTPRIHDIVEE

RLDAMEKAGRDGDPVDLVAAFALPIPSLVICELLGVPYEDRDDFERLSAA

RFDLFSGANASFGAISESLSYFREVVKKQRANPGDGLLGMIVREHGDSVT

DEELAGLADGVLTGGFETTASMLALGALVLLQDPEHFAALKDGDDAVDRY

VEELLRYLTVVQVAFPRFAREDLEIAGVQISAGDVVLCSLSGADRDAELG

PDMERFDPHRPKVPSHLAFGYGIHRCVGAELARMELRAAYPALVRRFPTM

RLAVPPEALSFRKLSIVYGLDSLPVRLDD

>CYP105B(2515555706)Streptomyces sp. MspMP-M5

MTTAERTAPPDALTVPASRAPGCPFDPAPGITDATRTEPITRATLWDGSP

CWLVTGHQEVREVLGDPRFSADAHRPGFPFLTAGAREIVGNSRTFIRMDD

PEHARLRRMLTADFMVKKVEAMRPEVQRLADELLDRMTDGRTTADLVTDF

ALPLPSLVICLLLGVPYEDHEFFQERSRILLSLRSTPAEVRAAQDELVEY

LARLARAKRERPDDAIVSRLVARGELSDADIATMGRLLLVAGHETTANMT

ALSTLALLRNPDQLARLRAEPALVKGAVEELLRYLTIVHNGVPRIATEDV

VIGGRTIRAGEGVLCMINSANRDAEVFPGGDTLDVSRDARRHVAFGFGVH

QCLGQPLARVELQIALETLIHRLPGLRLAVPYEEIPFRSDMAIYGVHSLP

IAW

>CYP107F(2515556155)Streptomyces sp. MspMP-M5

MAQETDSTVWNCPFDYAEALEFDPTLRRIMTEEPVARIRLPYGEGWAWLV

TRYDDVRTVTTDRRFSRHAIVGRDFPRMTPEPIVQDEAINVMDPPASSRL

RSLVSKAFAPKQVERMRARTQHVVDELLDRMLEDGAPGDLMENLASPLPL

TTICEVLDIPEDERAQLRGYARTMMNVSLENKENAVRAKAEMREYFAELT

ARRRRDPGDDLISALATARVGDEVLDAKELTVMAMVLLITGQDTTTYEIG

NLSYMLLTRPKDLAMLRENPEALPHAMEEMLRFIPFRKGVGIPRVALEDV

ELSGVTIRAGDIVHVSYLTANRDARKFERPDELDLTREPTGHMTFGWGAH

HCLGAPLALTEMQVALSTLLKRFPELRLAKPAEELRWNTTAIWRYPLALP

VVW

>CYP107CJ(2515557035)Streptomyces sp. MspMP-M5

MHPSEAMNPTDPPRLVELYGADYKRDPYPLYAELRARGPVHRVRFPSGVC

AWLVTGYDAAQKALTDPRLGKHHSRGNAAWRARASIMPEPQHSQLQVHLL

HQDPPRHTAMRRMITDAFAPQRVEALRPRFQEMADALLDRFAGAGRADLV

ADFAAHFPFRVLAEVIGLPAEFAERFDRDWGKVVQPVGPTDPGRPAYEAR

LHGLQQYIADLVAHQRRTAGGDLLSRLVSARDAGELAPEELDSMIFQLLV

AGQEPVTNQITTALVTLLRHPGHLAALAARPELLPRAVEELLRHDAAFEL

TTWRFFAEDSELHGTRIPAGDSVIVSLCAANRDPRRFADADTLDFDRSPN

PHLAFGQGIHFCPGAALARAELQIALGTLLRRLPDLRLTAQEDELPWIPA

VLARGVDRLPVAFGPESSGPAPRGCPISGAHAPSTPDAAC

>CYP107E(2515557046)Streptomyces sp. MspMP-M5

VTTADTAPLSYPFNIAESLDLAAEYDQVRDRPGLLKVQLTYGEPAWLVTR

YAEARFVLGDLRFSRAESARHDEPRQSEGTRNSGILSMDPPDHTRLRTLV

AKAFTVRQVEKLRPQVKELTRELLDELEAAGPPADLVDRYALPIPVAVIC

RLLGVPTEDRPQFRVWSDAALSTSSLTAEEFDANREELRAYMAKLIDQHR

QEPQDDLMTALIDAREVDVERLTELELIDLCVGILVAGHETTATQIPNFV

LALLDHPEQLALLREQPELIGGAVEELLRFVPLGSGAGQARYATEDIEVG

GTLVRAGEPVLVAVGAANRDALRFDAPGKLDIRRTGNQHLGFGHGVHHCL

GAPLARLELQEALLALLTRFPDLHLAGDVEWKTQMLVRGPRVLPVGW

>CYP105AC(2515557055)Streptomyces sp. MspMP-M5

MSHHPEREPLTELPITRPEGCPFDPPAELAELREEQPLRRMRYPDGHVGW

LATGYSVSRAVAGDPRFSSRYELMHAPFPGTEEVTFPPAPVGDLTGIDPP

HHTRYRRLLMGKFTVRRMRELTSRVEQITVEHLDAMERQGPPVDLVKAYA

HPLPALMICELLGVPYAERELFQEHAMGASSTDGTMEDQYAAMAALQEYV

GKLVLAKRARPTDDLLSDLTGTDLTDEELAGVGSFLLGAGLDTTSNMLGL

GTFALLTHPDQAAALRADPALADQAVEELMRYLTIAHTGVRTALEDVELD

GQLIKAGETVTLAIQAANRDPARFPDPDTLDLRRRATGHLAFGHGIHQCL

GQQLARIEMRVAFPALFTRFPTLRLAIPPEEVPLRTDLTMHGVQSLPVTW

DAA

>CYP1064A(2515557135)Streptomyces sp. MspMP-M5

VPGGVAALPRCLGLGKSRPSVLHWMTQRMTRPANGAVAVGSRCPEVPVES

PAAGRQSEEAAASIPGPEPRPDGGAGAIAAAGGLHTYQLQLHAAYGPVVR

FQLPGVEWAVSVADPVLLEATARLDERPDALFAFLDPLCEAGNIQVMPAA

EHAPWRRLLLSVLAGRPSHERHFARFTALTTQLADRWAERADRRPVELQK

DLTALSLRMICAYALGSEIADPESVIAAFEEVLTEHLGRLYETPGGDAQE

GRAEQAAGGVKAAKAARALAHLRATVDQVVTAHRTVDRTGAGTGNGTDRT

DRSDLIGVLLAAGERPARIRDTVMMTMLAAHHTTGVAVSWTLYLLGRHPE

VAARVADELDRVLGDRAAPDYPDLRRLTYLEMTLKEAMRLYPPGPYGARE

AVEPLLLGGHLIPAGATVFYPIWAVHMNPDYWPQPEKFLPERFAPDEVAK

RPRLAHIPFGFGPRSCEGAGLAMIEAELVLAVLLKRFRFRPAPGHQVTPI

ERFVLWAADDIRMNVSRRSPG

>CYP105AA(2515557621)Streptomyces sp. MspMP-M5

MSQPPPVPQGLPMDRDAGPFDPPSRITRLRETRPVSPLAFPDGHQGWLVT

GYEEVRRLMADTRFSSRQDIGVLHVPYETPGMPTPTEPSPQIPGLFVAMD

PPDHTRLRRRLTGAFTVKRMKQLEDHIIEITERQLDELARLAPPVDLVKE

FALPVPSLVICALLGVPYADRDTFQVNSAKFMEKDIALDEKMAAYGALTS

YLAELVTRKRAEPGEDVLSDLARHEDLSIEELTGAAFLLLLAGHETTANM

LALGTFALLEHPEQLAELRADPELLPDAVEELLRYLSVGDIFYRYATEDL

ELGGETITEGSTVVVSLLAANRDPRRFENPDTLDLHRRARGHLSFGHGIH

QCLGQQLARIEMRAGFGGLLRRFPTLELAVPAHEVRLRTDMNIYGVHELP

VTWTEKTR

>CYP1237C2(2515557789)Streptomyces sp. MspMP-M5

VTGRTLTYPFAWPSPSEQPKEFTGLHDEPVVTVVLPSGDEALLVTRYQDI

RALLTDPRVSKNRNRPGIARMTTRKVKAFQSQVSMDPPAHTRMRSLISKA

FTPARVEGLRPRIEELAEELLDAMEAGPRPVDFATAFAFPLSIQVICELL

GVPADEREQFALKQTPPWDYMTELIARKRADPGDDLISALIEVHDEEDGR

LDATELHWWSTMLLLAGYETTANQLCSAVVMLTSHPDQLAKLTQDPGLLP

GAVEELLRCQVVGTSLSMLRYVTDDIEVGGVTIPRGSSIITALEVANHDP

SAFRCPAHFDVTRSGDSPQLTFSVGRHFCVGASLARAELQIGLASLLHRF

PNLRLGVPADRLVRVEDAFTQGFREVPVTW

>CYP1237B3(2515557790)Streptomyces sp. MspMP-M5

MAERCPYPFPRPTALGVPERLAGVHDEPLVPVVLPSGDEAVLLTRYRDVR

QVLTDDRLSRNLNRPDAARISRHNSMFQDPRINPDPPEHTRVRNLVAKAF

SAARVQRLEPHIRAVVDELLDAMERSGGPLDLNEALAFPLPVRVICSLLG

VPDEDVALFRHWTSAFLSISHFPPEEIRAAMAEMSRYIAEHISHRRAHPG

DDLVSAMIRARDDEGQLSEYELQWWCRLLLLVGYETTATQLGGGIAMLLS

HPEQLRLLRADPALLPNAIEELLRWKLVGSSVSMLRYATDDIELEDCVIK

KGMSVIPGADCANQDPERFPDPRAFDITRSENPHLTFSLGPHFCIGASLA

RAELRIATEALLARFPNLRLAVPAEELKREEGALLEGFTEIPVSW

>CYP105DN1(2515557805)Streptomyces sp. MspMP-M5

MNAPDGRGDRPVAGDKCPVGAVPGGAPLFPMRRPCPFGLPPAYAEMREHA

PVRRARLAGGALVWLVSRHEDIRTLLGDARLSADRSRPGFPFLDSETAQL

ADVPVFLSMDAPDHLAYRHMFRSEFRPGQVDELRPFVQHCVDEHLDRMVT

QGPPADLVTSLAMPVPALTAGRILGVPEGELPRLEELSVAALTHRDTKAY

EELFNFVREIADDAAESPASAGMIGRVLREYVAAGQLDAWSMLATVFVIL

MAGYETTAHMISLGVLTLLHHPEQLAAVRRDPALVPQAVEELLRYLSVGE

LAAVRIAAEDIELHGTTIRAGEGVVLLGAAGNRDPRVFEDPERFDIHRSP

ARHLAFGHGPHACIGARVARLELAVVLTTLLERLPGLRLAQRITDDAVDY

EAVLFGLKRLEVTW

>CYP107X(2515557886)Streptomyces sp. MspMP-M5

MQNTAETGPDAPIDTTPLLDDPYTALAALREAGPVHRITGTDGQPAWLVT

RYDDVRSALADPRLSLDKRHATPGNYRGFSLPPALDANLLNMDPPDHTRI

RRLVVKAFTPGRVEALRGPVQRIADELLDAMAAKGRAELVADYAGPLPIT

VICDLLGIPAEDRRNFLAWSDALITPDPGRPQAMKEAIGAMLEFYTALIA

AKRAEPGDDLLSDLIAVRDDAAGTTDADRLGEDELTSLAFLILFAGYENT

VQLIGNAVLALLDHPEQLTALRRNPAEVTPAVEEFLRYDGPAALAIRRFP

VEDMEIGGVRIPAGESVLLSLASANRDPHRFPAPDRLDPARDLSGHLALG

HGIHYCLGAPLARMEAEIAIGALISRFPGLRLDVPRDEVRHRRTIRARGL

ISLPVAW

>CYP105B(2515557918)Streptomyces sp. MspMP-M5

MTEMIDTTVPAAPAPLPVEPPSGCPFHPPAEFGTLRTEEPISKISLPDGS

WAWLATRYADIRAILGDTRFSSDTTLHGYPLSGMTGGANTKNRGFIRMDP

PEHTRLRRMVTREFMVKRVEALRPEIQRLTDELCDAMERRAGEPVDLVEA

LALPVPSLVISLLLGVPYDDHDIFQRLTGTLLSRTVTDEEREAARTELRD

YLDALVTAKEQEPGDDILGRLIVEQQRTGEISHDDVAAFAALLLIAGHET

TANMIGLSALTLMQDPDAAERLRQDPALIRGAVEELLRYHSIIRNGPRRV

ATTDIEVGGQLIRAGEGVVAAVPSANHDDRVFDDPDRLDLARPNAQHHVA

FGYGIHQCLGQALARVELQVVIATLLRRFPAMRPAVPVEEIPFRTDMAIY

GCHSLPVTW

>CYP102G(2515557928)Streptomyces sp. MspMP-M5

MTDTIAPQTGDRTADAGVPVADLTATGISHAPIQQAMDLARIHGPAFKRK

FGARETLFLSSLDLVTEVADETRFAKGVSVVLEDVREFAGDGLFTAYNDE

PNWARAHELLMPAFALGSMRTYHPAMLKVARRVLASWDRRMAEGRPVEVA

DDMTRMTLDTIGLAGFGFDFESFSRDTPHPFVESMVRCLEWSMTKFSRQP

GTDHSAADAAFRADADHLASVVDEVIAARTAGGERRDDDLLGLMLTAAEG

NAAHQGPVLDLANIRNQVITFLIAGHETTSGALSFALYHLLKDPVALRLA

QREADELWGDDPDPDPSFEDIGRLPFTRQVLNETLRLWPTAAAFTRQART

DTVIGGRYPVAAGEQVTVLTPMLHRDPVWGDNPEAFDPFRFTPQAEAARS

PHAYKPFGTGERACIGRQFALHEATMLLALLVHRYRLIDHTGYRLRVKET

LTLKPEGFTLTLAPRTPADRAAVRAALAVLPGAAADDATGAAPVDGLPTR

VPQDTGLLLLHGTNYGTCREFAERLADEATALGFATEVAPLDAHAGGLPA

DRPVVIVAASYNGQPTDDATAFTAWLGTAPEGAAAGVRYAVLGVGDRNWA

ATYQQVPTLLDDRLAALGGERLLPRAEADASGDLTTTVKEFGAALRTELL

VRYGDPATVGAPGSDAAGTDDTGYTVTALTGGPLDALAARHGLVPMTVTE

AHDLTAEGWPRPKRFLRLALPDGVSYRTADHLAVLPANTPEAVARTARAL

GADLDTVLALRPPTGRPARDTLPVDRPLTIRQLLTHHLELGARPTRAQLA

LLAAHNPCPPERHALENLPEDDPRTLVELIETHPALRGALPWPVVLELLP

PLRTRHYSLSSSPAADPRHADLMVSLLPGGTGSTHLHTLRPGDTLLARVQ

PCREAFRLDPADDTPVIMVAAGTGLAPFRGAVADRVAAGRTTPARLYFGC

DDPDGDFLHAAEFAAAERAGALTVHPVFSARPENGHRFVQHRIEAEAAEV

WELLQAGARVYVCGDGSRMAPGVRDAFRAVHRAATGASEQESEAWLRELT

AAGRYIEDVYAAG

>CYP163B(2515558031)Streptomyces sp. MspMP-M5

VTVTTPALDTVDLGDPGTFAKHDLDGFWRHLRDSAPVHWNPPADGRRGFW

VLSRHEDILAAYRDDVHFTSERGNVLVTLLGGGDAGAGRMLAVTDGHRHH

ELRKILQRVLSPRVLSEVAAAVRVNTRQLIREAVEAGGCDFAEQIAGRIP

MNTISNLLGVPAQDREYLLAQTKTALSTDAEDVDEVDSEMARNEILLYFM

DMVEERRESPGDDVISMLIGSSIDGVPLSDEDIVLNCYSLIIGGDETSRL

TMIDSIHTLAARPEEWRRLKHGEVAIDTAVDEVLRWASPTLHFGRSVVRE

TELHGVRLRPGEIVTLWHASGNRDERVFDRPGTFDLGRTPNKHMAFGYGP

HFCVGSHLAKVEIAELLMALRDFTAGFEQTGEALPIRSNFLTGFSTLPVR

FRPDHSATKEVD

>CYP107Z(2515558051)Streptomyces sp. MspMP-M5

MSELAESPFSKHVGKHPGEPNMMEPALLTDPFTGYGELRERGPVVRGRFV

DDTPVWFITRFDEVRDVLRDQRFANNPKCAAGGEGGETPTDRLLELMGLP

EHYRVYFSGSILNMDAPDHTRLRRLVSRAFTARKITDLRPRVEEIADDLL

RRLPEHAEDGVVDLIKHFAYPLPITVICELVGIPEADRPQWREWGAKLVS

LQPKPLSEAFPAMVEHIHELIRERRAALTGDLLSELIRVHDDDGGRLSDI

EMVTMVLTLVLAGHETTAHLIGNGTAALLTHPDQLQLLKSDPELLPRAVH

ELMRWCGPAQLTQMRYATEDVEVAGVQIKKGEAVTPILVAANFDPRHYAD

PERLDLTRHPAGRAENHVGFGHGMHYCLGAGLARQEGEVAFGKLLARYPD

VALAVEPEALQRVPLPGNWRLAALPIRLG

>CYP121A(2515558173)Streptomyces sp. MspMP-M5

MSEPLLSTTLTGFEALPFCENCRSVFERGDHLLIGVSPGNSYFTHQRIAQ

LVAWAREFFAAVDVVYADLQVEAQFMACGYPAEQALRRAAKEVKATYRRI

HRGVLEADRSRVRIHALSDFLSHPAYRRLGRAVREALTGDREFREATEGM

ARSFLKARLDQGLAPSAEQVATGVEYIAAELPFLLNTPALLDVPSSVACY

HVELHVTAVLYGREGGLRAVPEQGYAVVRPVSRPQTALPDPSTTHQMESR

MTTTTQPEAAFPLSRRGDVLPEQAHLLCEKHPVARVRTMTGDVAWLVSNY

ALAKQVLEDERFSLKDTANPGVPRQYALTIPPEVVNNMGNINSAGLRNAV

MKTLSPRADKELAGWLETEAHQLIDRLVEQGAPGELRDGFTQPYSAALHC

RLLGIPTDDWRRLMSGIDVAFITSPTPFEGAAHNWYKDLRYMLDRLNADP

EPTEGLLGRFVELRRSPDVCDQVSDELLATVALSLFGAGAVSTSSFLLHA

IIALAQRPELAERIRREPPVIDRAVDELLRYNLSIGDALPRLALADVQIG

DVLVRTGELVLVLIEGANYDPEVFPHPERIDFDREPNPHLAFGAGRHFCP

ASAIARTHAQIALTALVQRLPELRLALPVEQLVWRPGFIKRLPERLPVLW

>CYP156B(2515558190)Streptomyces sp. MspMP-M5

MQSHPEFQTPPPGCPAHADGMRTSLHGPEFAAAPHAVYERLRQYGPSAPV

ELAPGVEAELVTDYATALQVLQNPDVFARDSRRWRALNEGRVPLDSPVLP

MMMYRPNALFSDGAEHMRLRQSVTASLAKVDTHRLGRHVGRVAAYLVGQF

SSRGRVDLVADYAQLLPLLVFNDLFGCPEEIGDRLVFGISGIFDGVEAEK

ANEVLTEALRELVALKRRSPGDDVTSWLTQHPSGLSDEEMVHQLVTLIAA

GTEPTQNIITSALLLLLSDERYAGDHNTAGLLVDDAINDVLWNSAPIANY

GVHYPVREVEIGGAKLAAGDPVVISFAAANTDPALSTGRQMLSKRAHLAW

GAGPHACPAKDPATLVANLAIEKLLNSLPDIELALPTESLTWRPGPFHRA

LEALPARFTPVRPERRSPDSSAWGQSAPENAPAPGKSGAQKGKWWSSFLS

WWKV

>CYP1035A(2515558191)Streptomyces sp. MspMP-M5

VEPGIVSLRTSPAVDRRSVARLLSRLRTPDGQANPLPHYAEIRSMGDVVP

APWGGYLVNSYGLCDRILRSKSWLVPDSSWRARQGRTPRWTAASSQEMAA

MLPGLNPPHHTRVRRSVGKIFDRRALVDIQKSVERFTEELLDELDVQLKD

GEADFGAIVSDQLPVLTIGDWLGLPTADYPLLRSLTHDQVFAQELLPSAS

QLALSDAATEILREYFTALVKERRKAPGDDPVSGWIRTWDHLERDREKAD

EAVYRLVLFVVLAALETTANLLSTAVWLITSHPRQMGWLRAQPENIPAAV

EEVLRYDSPTHVVTRVAGEDTVLAGVPVRKDQLVHCMVGVANHDPAQHVD

PHLFDARRRAGHLSFSSGIHYCLGSALARLEAHTLLTAVLRRFPGLRPSR

MPEWAPRVAFRRLLTLPVAQAGVGVR

>CYP184A(2515558380)Streptomyces sp. MspMP-M5

MALTNPSPRTGSGTAHPSPTSARRPIPGPAGLPVIGSLLDLHRDSLGAFL

RAQREHGDVVRLEAGPPGLRSVFYAVFAPEGVQQVLATQAANFRKDHPFY

EEVRQSFGNGLLTSQDADYLRQRRLVQPLFTRRRVDGYAAAVATEADAVA

ARWRSAADGVVDLVPELNRLALRTVARILFGVDTEAAVTAIHRCAPVIND

YVVRRAYTPVKIPRDWPTPRNLRNRRVTAELNALCDRIIAERRTAPGDAT

TGEPDHNDLLSLLAAAGNDEDGTLDATEVREQVLIFLLAGHETTATSMAF

TLHLLARHPDEQSRVRAEVAHVLGDRTPTAADLDRLPLLTRAFKESMRLY

PAAPVVSRRAVEATEVAGFPLPAGADVVVAPWVTHRHPDLWEDPERFDPD

RFAPEREAGRHRYAWFPFGGGPRVCIGQHFSMLESVLALATLLRSHELTA

LDQEVPVAAGITLQATGPARVRLRAL

>CYP107EB(2515558519)Streptomyces sp. MspMP-M5

MTTPPSGPNIMDPALIADPYGGFDRLREEAPLVLGQSADSTPTWYATRYE

DVRAVLGDPRFVVDPELTPGTEAVDNRNRMLEMLELPKEFHPYLSESILD

VDGDRHTRLRGLATRAFTARRVNALRPRVEAITASLLDSFGESVDLISEF

AYPLPITVICELVGIPEEDRSLWRTAGSALTSIAPGSKGAAARELIEYTH

TLVDKRRTAPTDDLISDLVKVQDEDGDRLSDVELVTMLLGLATAGHETTA

NLIGNGALALLSHPDQLEALRRNPELWPTAVDELVRWCGSILITQLRYAT

EDIDVGGQTIKAGEAVQPVVVSANRDPREFDRPECFDVTRRSTKPGDGHI

GFGLGPHYCLGAALARQECEVALRSLFDRFPRIALTTHEHTWVPVPGLRQ

LAALPLKLL

>CYP107L(2515558597)Streptomyces sp. MspMP-M5

MPEYPQTDQTRQGEQPPRASEAPQAPQGQRGAQGSQMSAPRNSPLTDPPD

SDALPVVDLPAYGEDFIADPYPYYAELRARGPVHPVRTPYDQDAWLVVGH

EAVRSALADPRLGKDWAKANLQRLAGETPLFTNMLDADPPHHTRLRKLVA

KEFTSRRVEALRPRVQQLTDELLDTMLTAPDGRADLIEALAFPLPMTVIC

ELLGVPSIDRDAFRLWSHELVSPTSPEAVAKATQDMADYLTALIETQRRE

PGDGLLSALIRTSDEDGDRLSHEELIGTAFLLLVAGHETTVNLIANGVRA

LLEHPDQLAALRADDGLLDNAVEEMLRYDGPVETATWRFASEPVEVGGTV

IPAGSPVLISLASASRDTERFAAADDFDITRDPRGHVAFGHGIHFCLGAP

LARLEGRIAIRALLDRCPNLALDTTADAPHWRSGLLMRGMDRLPVRWDG

>CYP105D(2515558674)Streptomyces sp. MspMP-M5

VTQTISFPQDRSCPYHPPANYRPLRESGPLSHVSLYDGRKIWAVTGHAAA

RTLLVDPRLSSNRQNPAFPITLKRFETARRVRTPLLGVDDPEHNAQRRIL

IPSFSLKRTAALRPRIQRIVDELLDRMLAQGPPAELVSAFALPVPSMVIC

LLLGVPYADHEFFEDCSRRLLRGRSAQESEASRLELEGYLGELIARKETE

PGDGLLDELIAERLRSGSLQREELVRLAMVLLVAGHETTANMISLGTFTL

LQHPDQLAQLRADESLMPAAVEELLRFLSIADGMLRVATDDIEIGGRTIR

AGDGVVFPTSLINRDEAAYPAPDELDVGRSARHHVAFGFGIHQCLGQNLA

RAEMEIALRSLFRRIPDLRLAVPAAEIPFKPGDTLQGMIELPLAW

>CYP180A(2515558868)Streptomyces sp. MspMP-M5

VADVPGIPDVFDPRRYAGGLPHDAYRLLRDHHPVAWQDEPEVLGWPAGPG

FWAVTRHRDVVRVLKDARTFSSHLGATQIRDPDPADLPFVRRMMLNQDPP

DHGRLRRLVSRAFTPRRVGRFETAVRERARALLTAAVRAARSGDGVCDVV

GAVTDDYALLNLADLLGVPPGDRGLMLHWTRRVIGYQDPDEGGPAATGAD

GRPLDPRSPALLQDMFAFARELAAHKRRHPGDDVLTALAADPELAVPELE

MFFFLLTVAGNDTVRSAAPGGLLALARHPDAYAALRTGAVAVGPAVEELL

RWHPPVLSFRRTAAVDTELAGRRIRAGDKVVVFHGSANYDERVFAGPGRL

DLARTPNPHVSFGDGPHVCLGAHLARLQLRVFYEEFCALLPTVTLAGPPQ

RLVSNFIHGLKALPLRMAR

>CYP107AE(2515558982)Streptomyces sp. MspMP-M5

VNNPMESAEFSRDPYPLLAALRARGPVQRVRTGKDRTTWVVTGWAEARAA

LGDGRLSKNTARYFADRPSGRDVAPAVSRSMLATDPPDHGRLRKLAMAAF

TPAAVSRLEPRVREIAERLAAALGRAAEAAGGGPVDLVEGFAAPLPIAVI

CELLAVPDADRAAVRRWSDDLFAAADPDTTDRASHALAGYMTELIAARRA

APGDDVLSGLIAARDAGDRLTEPELVSLAVLLVVAGHETTANLIGNGLLA

LLRDDALRARLRDDPGLLPKAVEEFLRFDAPVTLATFRYATEPFELGGVR

IGAGDVVLVSPGGANRDPARFDAPDTVRLDRPAPGGHLAFGHGPHHCLGA

PLARAEARIAFEVLLARFPELRPVTDERGGNEDGPGGGVTWRRTRLMRGP

AQLPALLGPFADSRNDERPSRPGA

>CYP1240B(2515559206)Streptomyces sp. MspMP-M5

VTSPYEPGAAPPPSCPGQAVPPQPEAAAPPPGCPAHGMPPQAPAPPDAYA

PVKLYGPEFAADPQRVYAQLRQYGAVAPVEIAPEVTAMLVTDYRAALDLL

NDDSTWSKDSRAWMQTVPADSPVMPMLHWRPNVFYSDGPAHVRYRDVIVD

SFKLVEPHELRARVHHAADTLIQRFGARGEADLIADYARLIPLLMFNTLF

GLPDSYSERLIAAIAGMMEGNSPEEANAANEAYTQYIMELVGAKKAQRGP

DLTSWMMDHPNDLGDEELIHNIILVMGAGNEPLANLIGNSLARMLSDDRY

YHTVSGGALTVHDAINEVLWNDPPMANYSAHFPVRDVFFHGTWLRAGQLV

MVSYAAANSQFDTTGVDGPGSGSGSHLAWAAGPHACPVKRHALLIAVTAI

ERLTAWLSDIELTVQPSELTWRNGAFHRALTALPTRFTPITPDQAGATPW

HNSERGPSSSTPPEPTSTGRTPASAH

>CYP154A(2515559207)Streptomyces sp. MspMP-M5

VRGSWLQSWIGVTNMFTAYGVDHRRLRKLIAPAFTARRTEAMVPRVKEIT

RDLLDGLAARPAGEVVDLRESFNHPLPMQVICELFGYPEGEPRRELARVV

AEIMDTTATPEQAAATQRAVAELLGSLVAAKRAAPADDLTSLLVAARDDE

GQGMTEKELLDTLLLVIGAGHETTVDLLGNAVFALLTHPEQLKLVRAGGA

SWNDVIEETLRWMPSIASLPLRFAVADVELPDGEVIRKGEALLPMYAAAG

RDPEQHGADAAEFDVRRAAQEHLAFGHGVHHCIGAPLARLEARTALPALF

ERFPDIQLAAAPEELTPAGGFIAGGLASLPVRLTA

>CYP105AC(2515559330)Streptomyces sp. MspMP-M5

MASVASVLPTERPAGSPFDPPAELAELREQRPLSRLDYPDGHVGWLVTSH

ALVREVLADPRLSIRTELRHLPVPGTPGSDRPAPPGMFTGMDAPEHTRYR

RMLTGQFTVRRMRQLTDRIQEITDQHLDALVQQAPPVDLVSALAQPVPAQ

VICELLGVPYADRARFQEDALALFRLDSAPERVTAAYRSVHEFVSELVAA

KRAAPTDDLLSGLTTGDLTDEELVNIGFVLLGAGLDTTANMLALGTFALL

SHPDQLAALRADPGVADRAVEELLRYLSIIPFTVRTALEDIELGGERIRA

GEAITVSLPAANRDATHFADPDVLDLLRPTGGHVAFGHGVHQCLGQQLAR

VELRVVFSALLTRFPTLQLACPVEDVAIRTDMLIYGVHELPVTWEA

>CYP147K(2515559570)Streptomyces sp. MspMP-M5

MTSAVQQHSASELFAQALRYENRANPYPLYEQLLQEPVVRLADGSWLATG

HREISLLLRDPRISADRLNPQQQPIRKSLLIADPPRHDQLRQAVTRQFVP

RIMGMRDHIDTLVTGLLDAHTSAGPGQLDVLGDLAYPLPVTIICELLGVP

REDERLFGGLARRLTRGLDPVETQTEEEIRELQQTRVELAEYLEGLIARH

DADGEGDLLAGLMNGQRPDGPMDAIDLRVTLGLLLIAGHETTVNLIANGT

LALLRNPGLLARLRAEPDLVTPLVEEVLRHDPPVQMSGRSTLADIDIAGT

TIPKGARIRLLLAAGNRDPRCFTDPGRFLPDRADNAHLGFGGGIHYCIGA

ALARAEAQIALTALARRLDSPRLVADPPPYRENAILRGPERLPVAFARLL

PDPREQ

>CYP125A(2515560074)Streptomyces sp. MspMP-M5

MHCPALPEGFDFTDPDVYQSRVPLPEFARLRRTAPVWWNAQPHGIAGFDD

DGYWVVTRHPDVKEVSTKPEVFSANLNTSIIRFNPTISRDQIEVQKLIML

NMDPPEHTRVRQIVQRGFTPRAIRALEEALRRRAGHIVEEARHKGSGDFV

TDVACELPLQAIAELIGVPQEDRARIFEWSNKMIAYDDPELAITEEVGTN

AAMELISYAMNLAVARKECPAKDIVSRLVAAEHEGNLGSDEFGFFVLLLA

VAGNETTRNAITHGMHAFLTHPDQWELYKRERPATAAEEIVRWATPVVSF

QRTATQDTELGGAHIRKGQRVGIFYSSANHDPSVFDRPDVFDITRDPNPH

LGFGGGGPHFCLGKSLAVLEIDLIFNALADAVPGISLAGDPRRLRSAWLN

GVKELQVRYR

>CYP105A(2515560424)Streptomyces sp. MspMP-M5

MADIATTPQATQAPAFPSDRTCPYQLPPGYAQLRDAPGPLHRVTLYDGRQ

AWVVTKHEAARKLLADPRLSSDRADANFPATSPRFEIARESRPAFIGMDP

PEHGVARRMTISEFTVKRIKGMRPEVEEIVHGFLDEMLAAGSPADLVSRF

ALPVPSMVICRLLGVPYADHDFFQDASRRLVQSTDVASAAAARDDLRHYM

DGLITAFQAEPGSGLVGSLVAEQLANGEIDREELISTALLLLVAGHETTA

SMTSLSAITLLAHPEQYAALRDDRSLVPGAVEELLRYLAIADIAGGRVAT

ADIEIEGQLIRAGEGVLVINSIANRDGTVYEDPDAFDVRRSARHHLSFGF

GVHQCLGQNLARLELEVILNALMDRVPTLRLATPVDQLKLRPGTTIQGVN

ELLVAW

>CYP1004B2(2515560948)Streptomyces sp. MspMP-M5

MTGAASAAKTHNRSALSWLPDPVGGRRIACMVLRELLPRFDPLAPDVLDD

PYPAYARLRATAPLCRMGPGSYGVTRHKDVATLLKDRRLGSEFPEAYHRA

SAGDGAAGAFFRRIVLYRDPPDHTRLRRLLGQAFSPRLVRSLEARIGGLV

DRLLEPARDTGRFDAVTDLAFPLPVMVVCELMGLSAADRDLIRPRAVDLG

KAFAAVVPEEDRSAADAAVTWLREYLDEVLAERARRPGEDLLSRMLAAEE

AGATLSHAEIVDNAVFSFFAGFETTMNLLATGCAALLEHPGELARLRADR

SQLPSAVEEFLRYDAPIQGTARLVREPVTVGDRTLRPGRVLVLLLGSANR

DGEVFRDPDRLDIGRDPNPQVSFGGGMHHCLGAVLARLEGRVVFGRLLDT

FAALEPDGPALRRTDSSFRAYGTVPVAVRPV

>CYP125G3(2515560967)Streptomyces sp. MspMP-M5

MTRPAPLADRTLLADPATYARGVPRAEFRRRLRHAPVDWVEERPLDRRDG

DRSRTEQGSGYWAVTRHASVTAASRDTRTFSSAARGAFLADPRTPEQLHR

TRQLLVSMDAPEHTRARRLIGNAFGPPAVRRLRGSIWEAAVKLVQRCRAR

GEFDLVSDLAAPLPLTVLAEVLGIPPEDRPLLLRWSNNLVGFDDPEYGGG

DVEVYRSIFTEAFGYAADVARQRRRAPADDIVSVLATAEADGERLSDAEF

GQLWLLLVVAGNETTRHAISGGVQALLDHPDQLERLAADPGLLPSATEEI

LRWTTPIMQFRRTLTRDATLDGTLMREGDKAVLYYIAANQDDTVFTAPER

FDITRDPNPHLAFGTGPHFCLGAALAREEIAVLLGALHPHLRRLRRAGEP

VRLESNFMNGLKYFPVRFDD

>CYP1694B1(2515561183)Streptomyces sp. MspMP-M5

MTQPVSAAGPAGPHSPADGSTEAGVLPATPDGHTAVRPRRASRGASLRFA

LTHTLPAFVRGVPSPRPAVIRLLGAAGQPRWSAATLRALRAGHDGAPVWI

GGPSGDLLVLLDPADVQEFFARPVRELALDAVDKTKMLTVFEPTGVICSH

GELRDRRRVLNDQVLARHEPVHPSWDAFRTIVAEECTRLTTGPALPFARL

RHTVQRISRRITLGDQAAGDEELHRWLLTLRKEGNWGAVRRGPTAANQRL

YEAAATRLHAYAPHAPAATLLGRVRATACDADVDAVGQAHHWLLALDSVA

AIVARTLLLLACHPAEQAALHETPHALDTTRLGACALESLRLYPIVPDLL

RILRTDTTWRGMPCPAGTHVLVPIGFLQRDSDVVPGGSLFIPSRWLAEGA

EHDPRMAPFGHGEGRCPGDRLGLMVATEVCAQLLREHRVTAGRPRLDPHR

PLPDTLESSGIHLTLARRRPHATADHS

>CYP107U(2515561237)Streptomyces sp. MspMP-M5

VQHHKPDARPETRPGPHPEGTPAPPAGCPMAAPAPAAPTPALFSWQFAAD

PYPAYAWLREHAPVHRTRLPSGVEAWLVTRYVDARQALADARLSKNPVHH

SENAHGKGKTGIPGERGANLMTHLLNIDPPDHTRLRRLVSKAFTPRRVAA

FAPRIQELTDQLIDAMIEKQHGAERGSADLIHEFAFPLPIYAICDLLGVP

PEDQDDFRDWAGMMIRHGGGPRGGVARSVKKMRAYLAELIHRKRAALQDG

APGDDLISGLIRASDHGEHLTENEAAAMCFVLLFAGFETTVNLIGNGTYA

LLRHPAQRELLQKSIAAGDEELLATAVEELLRYDGPVELATWRYATRELT

LGGQRIAEGDPVLVVLAAADRDPARFDEPDVLDLTRRDNPHLGYGHGIHY

CLGAPLARLEGQTALATLLTRLPDLRLAAEPEDLRWRGGLIMRGLRTLPV

EFTPESAPDSSRPGS

>CYP105BA(2515561301)Streptomyces sp. MspMP-M5

MTDTTTAVTNGLPTLRERPLDPPGTLRAGGAIRRMTFPDGYQGWLVTGYR

QGRQILSDKRFSSHASHKHLAFPSNRPSDLEADIPGLFEHLDPPDHTRFR

KRLAGQFTLRRMRLLATRIEEITAQYTDAMLRTGPPADLVTDYSVPVSSQ

VICELLGVPVAERDRFVGNSENLLRLDIEPQKVQASLKDLVDLTGELLMR

KKSDPADDVLSVLVTGDDITIEESVGATLLLLVAGHETTANMLSLGTYAL

LNNPDQMALLRANEALIDSAVEELLRFLTIVHVGVQRSPTEDVEIDGVTL

GKGETVLIHLPTVNRDPEQFTDPDRLDITRGGHSHLTFSHGIHQCLGQQL

ARLELRIGYTALLRRFPDLRIAGNPDEIPMRSDMTVYGVHRLPVTW

>CYP147B(2515561331)Streptomyces sp. MspMP-M5

MSTETLLERINDYASRPDPYPLYAELREHGVARQNDGSYLVGTYHEIAAL

LHDPRISSDVRHRTAPDASVRTDGLPPSFIGVDDPEHDRLRRLTMRPFGP

PHSPGRVDALHDDIEEIARELIDGFADRDRIDLVDDFAYPLPVTVICRLL

GVPREDMPPIRAWTNTIIASLDHSPGDDPEEKRRAGLEARMAMGGYLGDL

ADRRREHPADDMISALVHDDGPEGRLSRPELMTTLTLLMIAGHETTVNLI

TNGMLTLLRHPEELARLRREPELMPSAVEELLRYEPPVQMLPQRTPLTDV

AVGGVTVPKGAPLFLILASGNRDPLRFDDPDRFDPARRDNQHFGFGSGVH

NCFGAPLARTETQVALATLLHRLDAPRLVEDSPPYRHSPILRGPRHLLIG

KDTH

>CYP107EA(2515561371)Streptomyces sp. MspMP-M5

MTPPTTRPSQDNHPHTHEPSPTTPHPPSELRPFDAEFFRDPYPVYARLRK

LGPVLPVALPDGARAWLVTREEDVRAAFTDRRLSVNKARSRNGYQGFSLP

PALDANLLNTDPDDHLRLRRLVSKGFTPRHIEQLRERVVTAAEHYADGLA

ARLAECGEADLLAEFANPLPLVVIGHLLGVPEADGRTFSRWVAAMFAPDH

PGHTAEAIAHIHRYLLGLIGTRRARPGDDLLSSLIAARDEGDRLSEDELV

SLAFLLLMAGTENVQHLIAGGVLALLRHPEQLAELRRRPELMPEVVEELL

RYAHPNQWAIRRFPIEAVEIAGTRIPAGDTVLLGLASAHRDPDRYPEPDR

FDIHRADKAHLALGHGVHYCLGAALARMEISVALGTLLDRFSDLELALPV

GQLEWRASFRSHALRRLPVSVG

>CYP157C(2515561389)Streptomyces sp. MspMP-M5

LTSFHTDQPGTTSASAPPPECPAHARSTDGIARLFGPEVVEDAPGFFERL

RAEHGPVAPVLVDGDLPAWLVLGYRENLEVLRTPSRFSHDSRLWHCFKEN

RVAPDSPLMPALAWQPVCLFMDGEEHERFRVALTESMGRLDRRGIRRCVT

RSAHQLIDGFAADGQADLVSQFAEQLPLRVVAQLLGMPEEEGPRLVEATR

DLLRGSETAFQSNEYLMAALNRLVAAKRGAPATDFTSWLMTHSAKLTEEE

VAQHLRLVVIAGNENTTNLTANTLRMMLTDPRFRASLAGGSMTLPDALEQ

MLWDEPPTSVAPARWATGDTELGGQTVKAGDMLLLGLSAGNADPAIRPDL

SVPMHGNRSHLAFTGGPHECPGQDIGRAIVDTGIDVLLMRLPDIELAVPE

NELKWVSHWIARHLTALPVKFAPRTEKRSLAADSASASADEVQSPPSLES

STASGRETVEGTAASPSGVGAPRAPWSWWNQVKRWLTRK

>CYP113K(2515561647)Streptomyces sp. MspMP-M5

VTSKELSASPTVFDELIGRWVALQAAGPVRHDERQGVWQVLDHRSVAAVL

ADPATYSSDLSGLTPTQPDFETFREGNFVGMDPPRHRTLRTLVSQAFTPR

VVAGLEPRIHTLTTELLDAVADRDRFDVVEALTYPLPVIVIGELLGVPAE

DRGLFQEWAAVLFGGDDLGDAPDMADLERALNALAPTVREMNGYLLDHIR

RHRRAPGDGLTGRLLAAEADGVRLTDQEIVGFVALLLVAGHITTTALLGN

AVLCLDAHPDAAALLRAEPGRLPAALEEVLRWLPPFPELGRRTTREVEIG

GHTLPADTLVMPHLAAANRDPARFAEPDRFDVTRHPNPHLTFGHGIHFCF

GAPLARLEARIALRLLLDRFSDLAVPDDRDVVLQNPAVIVSPRHLPLEVR

RS

>CYP105D(2515561779)Streptomyces sp. MspMP-M5

MPDVITFPQDRTCPHQPPEGYRQLREQRPLARVALYDGRVVWAVTGEAEA

RRLLCDARLSSDNTHPSFPSLAARFADLQGMSLPLLTVDDPEHRRQRQRL

IPYFGVRRIAALRETVQRVVDRHLDAMVRQGSPADLVSAFALPVSSTVIC

SLLGVPYADHDFFEERTRWLMRGATAQQTRDGYAELCAYLEALVDGKESR

SASRTEAGKEVVHGDHGLLDELVEGCGRGGGPDRDELVALALVLLIAGHE

STAHSIATSVLMLLEHPRQLAALRADESLFPSAVEELLRYLSTLDGLVRV

AVEDMETDVAVIRAGEGVVFPLAVLNRDPAVHPRPDEVDVRRADRRHLAF

GFGVHQCLGQHLARLELEVALRTLLARLPELRLAAPAGSLVCTPGDASFQ

GVTALPVTW

>CYP107DW(2515561853)Streptomyces sp. MspMP-M5

MSEAIDLAELAATAGLEQELARLAADHGIIRTRQLSGQETWTVLSAPLTR

ELLSDPRLSNDVHTHAPHGALVPGLQVMLLEQDDPGHARYRRLVTAAFAS

KAVRQLEPRIVEITRQLLEKLGDSGTVDFIDAFTYPMPLEVICDLLGVPH

EDRDPFRKWAMDISAAPSLEAMQASAGELFAYCIGLIGAKRARPAEDLLG

ELIAARFEDGSGLTDEELSSFAAVLLIAGHDTVTNLLANALHELLTHPEQ

LAALRADRSLVGAAVEEALRFRGSAMTTVNRVALEDIEAGGVTIRKGELV

RFLLNAANRDAEVRADGHTFDLGRATAQHVAFGMGPHFCLGQRLARQEAT

IALTEILDRFPTLELGVPADEVRWLASDAIRGLEELPLRYARAAE

>CYP107MP2(2515562624)Streptomyces sp. MspMP-M5

VRAKQPRWPTGYWSSAGQAWGRAEASGSGVVGFAQLRLARSDHDPTAVAM

CLRNHERVRLDPAAPAFSYDPYPYYGRLRGQGPAARVELANGTHAWLVTG

YEHSRAVLADPRFSNVPPQRAGRPKPSSPAQRARACLAQHMLNADAPDHT

RLRRLTAAAFAPPRVDALRPRIEQLAAGLVTELTGRLGAGETVDLVDSLA

FPLPILVISEVLGVPEADRAGLREWTYRVGSPADALPQGAVDEAWVLLHT

YVTSLIAEKRQTPGEDVFSALVHNSAEDGLDDGELLAMAFLLLFAGYETT

MNLLASASLRLLTHPGELTAARRAPGSRWAAVVEETLRYDSPLEGATWRR

AAETVDLGGGVEVPAGDSVLVVLAAANRDPAHFPDPDEFRPARHLAGADG

KRAAPHTAFGHGPHFCVGSRLARMEAAIALPLLFDALPDLRLAADPKDLP

YRPGLLVRGPRRLPVTTAQAT

>CYP251G(2515562711)Streptomyces sp. MspMP-M5

MSRTDTEPTGARRAAVAPGRVPLVGHAAALHRDALRFLCGLRHHGPVTKI

YIGPRPVHVVNSPEIIRELLTLQARDFDKGAMFDALRVPLGDGLITTAGD

RHRRHRRLMQPAFHHERIARYARTMAERSQARAADWAPGTTRDLVPEIHR

LTLDILLRTLFADPQDPALDAAVKGWLTVKYHSMRLALSPLHAWAERLPL

LPGWRPPDPGPLRRLVDVQLRIIESYRADGRDRGDLLSMLLLAGGPEGAL

TDAEVTDELITLFLAGTGTVSASLAWALHEISRRPGVQRRIHDELDTVLD

GRPPGFEDLPALVYTRQVLTEVLRLYPPSWLLMRRAVRPVTLGGVRLAPG

DEVFFSPYAVHRDPRLYEDPERFAPDRWPSDAAAKAPRHTFLPFGAGSRL

CIGEDYAWAELTLAVAAFTAQRYLEPADTTPVRPLVGTVLRPDRLPLTAR

ARPS

>CYP156B(2536362545)Streptomyces coelicoflavus ZG0656

MNAHDTAPVPPPGCPAHGSGARVPLHGPEFAADPQAYYEHLRHYGAAAPV

ELAPGVEATLVTDYAAALQLLQDSGTFRKDARRWRAFNEGRISPDSPVAP

LLAYRPNCMFADGADHLRLRQAVTDSMARVDTRRLSRSTEQISGYLISQF

ATRGSADLLGDYAKQLPLFVFNELFGCPADIGDRVLFGISGMFDGVNAEK

ATAVLFQAVGELVALKRRKPGDDVTSWLMRHEAGLDDEEMVHQLALLLGA

GAEPLRNLLGNTLHRLLTHERYAREGGLIDEALDDTLWENPPMANYAPHY

PAADTELAGQQLRAGDLVLVSFAAANTGPALSASRQAGSNRAHLAWSAGP

HACPSKEPARHITVTAVEHLLNELPDVELAVPEDSLTWRPGPFNRALATL

PARFTPVRSARRAEPGQAAAAEREQPANTARPAEHKGMWSQFLNWLTR

>CYP158A(2536362654)Streptomyces coelicoflavus ZG0656

MSEETMSRTVPPVRDWPAVDLPGSDFDPVLTELMREGPVTRISLPNGEGW

AWLVTRHDDVRLVTNDPRFGREAVMDRQVTRLAPHFIPARGAVGFLDPPD

HTRLRRSVAAAFTARGVERVRERSRGMLDELVDAMLKAGPPADLTEAVLS

PFPIAVICELMGVPATDRHAMHTWTQLILSSSHGAEVSERAKNDMNTYFS

DLIGLRSDSTGEDVTSLLGAAVGREEITLAEAVGLAVLLQIGGEAVTNNS

GQMFHLLLSRPELAERLRSEPEIRPRAIDELLRWIPHRNAVGLSRIALED

VDVKGVRIRAGDAVYVSYLAANRDPEVFPDPDTVDFDRSPNPHVSFGFGP

HYCPGGMLARLESELLVDAVLDRVPGLKLSVAPEDVPFKKGALIRGPEAL

PVTW

>CYP158A(2536363216)Streptomyces coelicoflavus ZG0656

MTQETTTLTGQTPPPVRDWPALDLDGPEFDPVLADLMREGPLTRVRLPHG

EGWAWLATRYDDVKAITNDPRFGRAEVTQRQITRLAPHFKPRPGSLAFAD

QPDHNRLRRAVAGAFTVGATKRLRPRAQEILDGLVDGMLAEGPAADLVER

VLEPFPIAVVSEVMGVPAADRERVHSWTRQIISTSGGAEAAERAKQGLYG

WITDTVRARADSDGDDVYSMLGAAVGRGEVGETEAVGLAGPLQIGGEAVT

HNVGQMLYLLLTRPELMARMRERPEARGTALDELLRWISHRTSVGLARIA

LEDVEVHGTRIAAGEPVYVSYLAANRDPDVFPDPDRIDLDREPNPHLAYG

NGHHFCTGAVLARMQTELLVDTLLDRLPGLRLAVPADQVAWRRKTMIRGP

RTLPCTW

>CYP157B(2536363398)Streptomyces coelicoflavus ZG0656

MTDLDPAPRPVAAPGCPAHPDAVPLAGLEYQQTPSDLYRGLRAEHGAVAP

VLLDGGIPAWLVLGYPEVSYVTSHDDLFARDSRRWNQWGSIPPDWPLLPY

VGHQPSVLFTEGEEHRRRAGVITQALAGIDQFELARDCRHLADRLISAFA

GSGRAELMSGYAHPLPMLAAVRVCGMPHNGVETRQLVEDLRISLDAAEGD

DPVAAYTRVGERIHQLVRHKRDRPGPDVTSRMLTHPAGLTDEEIVQDLIS

VIAAAQQPTANWIGNTLRLLLTDERFALNVSGGRLSVGEALNEVLWLDTP

TQNFIGRWAVNDTQLGGRQIRAGDCLVLGLAAANTDPQLWPEAHVGAENS

AHLSFSNGEHRCPYPAPLLADVIARTGVETLLERLPDLVLAVEPGELTWR

PSIWMRGLTTLPAVFTPVVA

>CYP159A(2536363399)Streptomyces coelicoflavus ZG0656

MSTAQQVPDILSPEFAANPYPAYRTMRDSAPLIRHEATQSWIVSRYEDVE

RVFKDRGGQFTTENYDWQIEPVHGRTILQLSGREHAVRRALVAPAFRGAD

LQERFLPVIERNSRELIDAFRHTGRADLVADYATRFPVNVIADMLGLDKA

DHDRFHGWYTSVIAFLGNLSGDQQVAAAGARTRVEFAEYMFPIIRERREK

PGDDLLSTLCAAEVDGVRMSDEDIKAFCSLLLAAGGETTDKAIAGIFANL

LAHPDQLAAVREDRSLIPRAFAETLRYTPPVHMIMRQTATDVTLSGGTIP

AGATVTCLIGAANRDESRYRDPDRFDIMRDDLTTTTAFSAAADHLAFALG

RHFCVGALLAKAEVETGVGQLLDALPGLRTEDGFEVVERGVFTRGPQSLP

VRFTPAA

>CYP152D(2536365277)Streptomyces coelicoflavus ZG0656

MDRTPALLVEGYAWLPDRMRDSTRSVLRTRLLGRPALAVRGPDAVRFFYD

ESHVHRHGAIPAPVLDTLFGQGAVHTLDGVAHRTRKELFLPLLEAGRVAR

LTAHVTEAWDEAVRSWSGRERVVLFDEAAVVLTRGVCDWAGLPPRAVDPQ

TLARDLVAMVDGFATPGPRHLRARRARTRQETRMAGLIEEVRSGEVAAPE

DSVLERVARHRDTGQGPLDPRTAAVELLNVLRPTVAVSWFVAFAAHALHR

WPAHRERLRAGDAAFATAFAHEVRRFYPFAPFLGGRTVTELTWHGESVPA

GGILLLDVYGQHHDEELWGDPYTFRPERFLDRPPGPDELIPQGGGDPAAG

HRCPGERVTVGLLEALAVRLARLECTVPAQDLRIPLRRVPTRPRSGFVVT

GVRAP

>CYP155A(2536365312)Streptomyces coelicoflavus ZG0656

MVQDGRVSARKADRGRAGPVCPVGRAADGTWQVHDFAVARALLRGPGTVQ

AGLGVETVEKLPPRVRRPVLYRDGPEHREHRRQTARYFTPRRVDEHYREP

MVRIAEEQLAALRSAGEAPLSDLAFGLAIGVVSEVVGLRHSRPGIRRRLE

RFFPEEFGEPGLTSARGLYWLVRQNTNWLRIHLADVRPAIRAHRRREHDD

LISHLLAEGCSDVEILGECLTFAAAGMVTTREFVCLAAWHLFSDAELLGH

YRSADEAGRLAVLQELLRLEPVIGRLRRRATEPVELPCPGGPVTVCPGEE

VEVHLDDANSDPKAVGADPLAVRPERAETVGAGLSFGDGPHRCPGAHIAL

LETDVFLSRLFALDGVRMSAAPRVTFQEAIDGYEIRGLTVALPRAGHG

>CYP105N(2536366986)Streptomyces coelicoflavus ZG0656

MNSPETSTASHAPGPLGTDAAPESAAPPRDFPVQRGCPFAAPAEYAALRT

HDPVARVTLPTKKEAWVVTRYDDVRELLSDPRVSADIRRPGFPALGEGEQ

EAGARFRPFIRTDAPEHTKYRRMLLPVFTVRRVRAMRPAVQARVDEILDD

MLAAGGPADLVPAYANAVSTSVICELLGVPRENLEYFRDVTRISGSRNST

AEQVSEALGGLFGLLAELVGKRREEPRDDLISKLVTEQLVPGHVTTEQLL

STLGITINAGRETTTSMIALSTLLLLDRPELMEELRRDPSLMPAAVDELL

RVLSVADSIPLRVAAEDIDLSGRTIPADDGVIALLAGANHDPEQFEEPER

VDFHRTDNHHVAFGYGVHQCVGQHLARLELEVALETLIRRVPTLRLAGDR

DDVVVKHDSATFGLEELRVTW

>CYP102B(2536367692)Streptomyces coelicoflavus ZG0656

MAQTAREPARDGLPKGFRSAELGWPELHRIPHPPYRLPLLGDVVGASRRT

PMQDSLRYARRLGPIFRRRVFGKEFVFVWGASLAADLADETRFAKHVGLG

VANLRPVAGDGLFTAYNHEPNWQLAHDVLAPGFSREAMAGYHVMMLDVAA

RLTGHWDLAEASGRAVDVPGDMTKLTLETIARTGFGHDFGSFERARPHPF

VTAMVGTLGYAQRRNTVPAPLAPWLLRDASRRNAADIAYLNRTVDDLVRE

RRSTGGDGGRGGDGDLLDRMLETAHPRTGERLSPENVRRQVITFLVAGHE

TTSGALSFALHYLAQHPDVAARARAEVDRVWGDTEAPGYEQVAKLRHVRR

VLDESLRLWPTAPAFAREAREDTLLGGAYPMRPGAWALVLTGMLHRDPEV

WGPDAERFDPDRFDAKAVRARAPHTFKPFGTGARACIGRQFALHEATLVL

GLLLRRYELRPDPGYRLRVTERLTLMPEGLRLHLDRRTAAPASSPPHPDG

AGAAGEQDAGNAGSASRCPVHRAGD

>CYP105D(2536367744)Streptomyces coelicoflavus ZG0656

MTDTDTTTNTHPAAPVAFPQDRTCPYQPPAAYDPLRAARPLARVTLFDGR

PAWLVTGHAAARRLLADQRLSTDRTRDGFPATSARLAAVRGRRTALLGVD

DPEHRTQRRMVLPEFTLRRAAALRPHIRRIVGERLDAMVAQGPPADLVTA

FALPVPSMVICALLGVPYADHDFFEEQSRRLLRGPLPADTMDARDRLEEY

LGELVDRKRTAPGDGLLDDLVRRQRDEGGTDREQLVAFAVILLVAGHETT

ANMISLGTYTLLTNPGALAELRADPALLPGAVEELMRVLSIADGLLRMAT

EDIEVDGQTVRAGDAVVFSTSVINRDESVYPDPDALDWHRPARHHVAFGF

GIHQCLGQNLARAELEIALHTLFDRLPTLRLAAPAGEIPFKPGDTIQGML

ELPVTW

>CYP107P(2536368343)Streptomyces coelicoflavus ZG0656

MTAASDSATGRASEPASGLAFAPWNPAFVADPYPAFAELRARGRVLYYEP

SDQWLVPHHADVSALLRDRRLGRTYQHRFSHEDFGRTPPPPEQEPFHTLN

DHGMLDLEPPDHTRIRRLVSKAFTPRTVERLKPYVHGLADDLVARLVAAG

GGDLLTDVAEPLPVAVIAEMLGIPESDRAPLRPWSADICGMYELNPSEET

AAKAVRASVDFSEYLRTLIAARRKDPGDDLISGLIAAHDEDDDRLTEQEM

ISTCVLLLNAGHEATVNATTNGWLALFRHPGQLAALRADHSLVPSAVEEL

MRYDTPLQLFERWVLDEIEIDGTTLPRGAEVAMLFGSANHDPAVFTDPER

LDLTRRDNPHISFSAGIHYCIGAPLARIELAASMTSLLERAPGLRLAAEP

ERRPNFVMRGLTELRVEL

>CYP107U(2536368696)Streptomyces coelicoflavus ZG0656

MTGNPPVPGSASGPVPGSASGPAPGPASGPVPELFTWEFASDPYPAYAWL

REHAPVHRTRLPSGVEAWLVTRYADAKQALADPRLSKNPAHHDEPAHAKG

KTGIPGERKAELMTHLLNIDPPDHTRLRRLVSKAFTPRRVAEFAPRVQEL

ADGLIDRFAATGSADLIHEFAFPLPIYAICDLLGVPREDQDDFRDWAGMM

IRHGGGPRGGVARSVKKMRGYLADLIHRKRAALPPEPAPGEDLISALIRA

SDHGEHLTENEAAAMAFILLFAGFETTVNLIGNGTYALLTHPEQRERLQT

SLAAGERGLLETGVEELLRYDGPVELATWRFATRPLTIGGQEVAAGDPVL

VVLAAADRDPERFTDPDTLDLARRDSQHLGYGHGIHYCLGAPLARLEGQT

ALATLLTRLPDLRLAADPAELRWRGGLIMRGLRTLPVSFTPPVSSAGNGP

SPTQK

>CYP156A(2536368844)Streptomyces coelicoflavus ZG0656

MTLPSAEPAPPGPPGRIALYAPEFAADPHAAYRSMRRTHGPLVPVDLAPG

VPATLVIGYYQARRILNDPLHFPADPRAWEKLIPATCPVRPMMEWRPNAL

RSGGAEHARYRSANTHAIDHVDQHGLRALVEKVADGAIDGFRATGSADLL

TQYSFPIAFSVLSALLGCPDEIGQRIADGMAKIFDTTNAEQGNVILARAV

SDLVALRRAHPGDDITSRLALHPVELTDEEMSHQLVTLYGAGIEPMTNLI

SNTVLKILTDEEFSADLHAGLSTVRDALDAVLYTDPPMANYCISYPPYPI

DVEGVLLPADQPVVISMAAANNDPALTEGVPAGRLGGNRAHLAWSAGPHT

CPARSHAYLIAETAVTHLLDALPETDLARPLAELAWRPGPFHRALESLPV

TFPAAQPAAL

>CYP154A(2536368845)Streptomyces coelicoflavus ZG0656

MATQQPALVLDPTGAGHHAEHRTLREGGPATRVDVLGVEAWSVSDPALLK

QLLTSPDVSKDARAHWPAFGEVVTTWPLALWVAVENMFTAYGPNHRKLRR

LVAPAFSARRIAAMRPAVEGMVTGLVDRLAALPAGEPVDLRQELAYPLPI

AVIGHLMGVPEERRDGFRALVDGVFDTTLDQAEAQANTARLYGVLDELIA

AKRATPGDDMTSLLIAARDDEGDGDRLTPEELRDTLLLMISAGYETTVNV

IDQAAHTLLTRPDQLALVRKGEVTWGDVVEETLRHEPAVKHLPLRFAVTD

IPLPDGRTIARGEAILASYAAANRHPGWHEDADTFDATRTVKEHLAFGHG

VHFCLGAPLARMEVTLALESLFGRFPDIRLADPAEELPPVPSLISNGHQR

LPVLLHAG

>CYP157C(2536369434)Streptomyces coelicoflavus ZG0656

MTPERHSPTGTGEPLLEPPPGCPAHGLGPGGLHRLHEADDLEELYEKLRE

RHGPVAPALLHDDVPMWVVLGHAENLHMVSTPSQFCRDSRIWTPLNEGTV

KPDHPLMPHIAWQPICSHAEGDEHKRLRGAVTSAMSDLDFRELRRHIKRY

TQRVVNRFSEEGRADLVSQFAEHLPMGVMCHLLGMPEEYNDRLVEAARDT

LKGTDTAIASHAYVMEALGRLTAARRADPADDIAGRLVTHPAGLSDDEVR

EHLRVVLLAAYEATVNLIANVMRVVLTDPGFRAQLSGGQMTVPQAVEQSL

WDEPPFSTVFAYFAKQETELGGQRIRAGDGLLLGIAPGNVDPRIRPDLSA

DMMGNRAHLAFGGGPHECPGQDIGRAIADAGIDALLMRLPDIQLDCDEDE

LRWRSSIASRHLVELPVRFEPRAQQDIRQQPSHAPAPERRAPWHVGMPKP

EQRTQPRLPSHPPQPVSVTAAAPQQAPDPENGRPRGVWQRFLRWWRGY

>CYP184A(2536370068)Streptomyces coelicoflavus ZG0656

MTNPFDRPDTDYLVLVNDRREHSLWPSTIDVPAGWTVAFGPAGRPDCLEF

TATARTDPRAAGPCPVEHRTARPAPAPPDASDPQDAARHESSKRRDTTMT

ASTPTVSAPPPTMTGHPLFGSLMDLQKDTLGTYLKALRDHGDVVRFTVGP

PGMRAEFYGVFSADGAQQVLASSAQTFSKENRFLGELRQSFGNGLLTSMG

DEYLRQRRMLQSLFTPRQVNEYGSEITQETGSLVERWRTAPDATVDVAEE

MTGHTLRTISRILFGRKNDVDAMVPTVQRNFPLINAYAVKRAFAPVNLSR

KVPTPGNLRAAKAHRELYAVCDEIISARRAEESSGSTGRNDMLSLLARAH

DDDGNPISAEETRNQVLVFLVTGHESTATTLGLTLHLLARHPEAQARAHQ

EVDSVLSGREPVAEDLEKLPYLTRVLKETLRLYPAAPAQGRITTEDVRVG

SYTIPAGADVVVSSGVVQRRPDIWEDPEAFDPDRFLPEHEAARPRYAWFP

FGGGPRACIGQHLAMLNATLTLSVLLKNYSFTAVDTDIPLNTGITLRATG

QVRCRLTPRT

>CYP170A(2536370460)Streptomyces coelicoflavus ZG0656

MTVESVNPETRAPGAPGAPGAPELCEPPVAGGGVPLLGHGWRLARDPLAF

MSQLRDHGDVVRIKLGPKTVYAVTNPELTGALALNPDYHIAGPLWESLEG

LLGKEGVATANGPLHRRQRRTIQPAFRLDAIPAYGPIMEEEAHALTERWQ

AGRTIDATSESFRVAVRVAARCLLRGQYMDERAERLCVALATVFRGMYRR

MVVPLGPLYRLPLPANRRFNDALADLHLLVDEIIAERRASGQKPDDLLTA

LLEAKDDNGDPIGEQEIHDQVVAILTPGSETIASTIMWLLQALADHPEHA

DRIRDEVETVTGGRPVAFEDVRKLTHTGNVIVEAMRLRPAVWVLTRRTVA

ESELGGYRIPAGADIIYSPYAIQRDPKSYDDNLEFDPDRWLPERAKNVPK

YAMKPFSAGKRKCPSDHFSMAQLTLITAALATKYRFEQVAGSNDAVRVGI

TLRPHDLLVRPVAR

>CYP1005B(648861309)Streptomyces pristinaespiralis ATCC 25486

MHHDEATGLWLVSRHQDIRRVLSDPSVFLPDNAQHAVAPLPVAVLRTLAK

ARFSLPPALANNGTGSHAGLRRLVNRFFNARRVAAAVPVIERCAEELLDA

ARAGIDADGRADLFASYAQVLPCRVLMELLGVRGVTPDTLIRWSDASLEL

FWGRPAAERQLELAGLVGEFHRWLTDTVRGAASSPGVSPDSFVGALARHR

LPDGEPLDTATAVSACFFIFIAGQSTTGQLIATVLRRALCEPGLWPRLVP

EAGLAEDWVEEVLRREPPVTTWRRVTARPVELAGTRLPAGAQLLLMLMGS

GSDPAVFADPERMCPHRANVRHHLAFGAGRHRCPGASLARTEAAIALRAV

ARRLPQIRLAPEGARPQMLGLLSFRAPLDVMVERQPGC

>CYP107L(648861528)Streptomyces pristinaespiralis ATCC 25486

MADTPIVDLRELGPDFVRDPYPVYARLRAEAPVHRVLDPDGEEIWLVLGH

DTARAAFTDPRLSRDWVKSGNVGQIINTDQDQPALAHMLMSDPPDHTRLR

RLVTKGFTPRRIDALAPRIQQVTDELLDAMLAEPSRRADLIASFAFPLPM

TVICDLLGVPELDRDAFRRWSNEMVARTSPEAEAQAYEEMPAYLSELIAA

KRARPGEDLLSALIHAADADGDRLSPEELIGMSVLLLIAGHETTVNLIGN

GMRALFSHPDQLAALRADFGLLDGAIEEMLRYDGPVETCTDRLALEDVEI

GGVTIPAGSTVLITMADADRDPARFKEPDRFDIRRDARGHIAFGHGLHYC

LGAPLARLEGRIAFRTLLERCPDLAQDADEADLPWMPGLLIRGVRRLPVR

W

>CYP107L(648862103)Streptomyces pristinaespiralis ATCC 25486

MTIVDLDQYGPDFTANPYPYYARLRESGPVHRVRGADGGEFWLIVGHEEA

RAALSDPRLSKSPATIGVTMLDEQVIGPNLLVLDPPDHTRLRKLVSREFT

PRRAEALRPRVQRITDDLLDAMLPAGRADLVDALAFPLPIIVICELLGIP

AADRDAFRTWSNEVVAPTSQEAGEDAVRQLAAYLDELIEDKRSTAPTDDL

LSALVRSLPLPAEADGGAAEDDRLSTAELRALAYLLLVAGHETTVNLISN

GVRALLTHPDQLAALRADFSLLDGAVEEMLRYDGPVETTTVRFSAAPVPV

GDQVVPQGEMVLVGLAAADRDPARYEAPDRFDIRRDTRGHLAFGHGIHFC

LGAPLARLEARIAVRSLLERCPDLALDTPADTYEWLPGLLMRGVRRLPVR

W

>CYP124G(648863512)Streptomyces pristinaespiralis ATCC 25486

MEVAVARETPGRVASRPAAPAPETDLSDPTFWRLPRPARLAAFAELRARD

APVRFGERPGFYALVRHADVAEASRRPRDFTSAPGVTTPEPAPWAKALFG

NSMVNMDGPEHATLRRIVARSFTPRLLAATEDNVREVAGRIVGEMLAGRP

RDFVPAVASRMPFEVICDLMGVPGEARAEIAARIDQASENVGVQRGLRSR

LRMPGRGLRALAHMQLVMAGLARERRRRPTGDLISALVCADVDGQALNSR

QLGAFFSLLLVAGVETTRNAIAHGLSLLSEHPEQRALLESDFDRYADGAV

EEIVRHSTPIIQFRRTVAAECVLGGRGFRPGEKVVLFYASANRDEAVFED

PDVFDITRSPNPHLGYGGGGPHYCLGAHLARQEMKALFHELLTKAGGVRT

VGAPCLVDSSFDNRVRSLPFAFEAS

>CYP107P(648864221)Streptomyces pristinaespiralis ATCC 25486

MDALFEPWSPQFVADPYPAFAELRERGRVHWFEPTRQWLVPHHADVSALL

RDRRLGRTYLHRFTHEEFGRTPPPAAHEPFHRLNDHGLLDLEAPDHTRIR

RLVTKAFTPRTVQALEPVVQRLAAGLVADFRKAGGGDLLAEVAEPLPVAV

IAEMLGIPESDRPLLRPWSADICGMYELNPSQETAARAVRASTEFSAYLR

ELIAVRRERPGDDLVSALIAAHDEGDRLSEQEMISTCVLLLNAGHEATVN

TTANGWWTLFRHPGQLAALRADHSLLSTAVEELMRYDTPLQLFERWVLDD

IEIGGVVVPRGSELALLFGSANHDGTRFDRPDTLDLARADNPHVSFGAGI

HYCLGAPLARIELAASFGELLRRCPDMRLAAEPRFKGGFVIRGLEELLVE

L

>CYP154U(648864933)Streptomyces pristinaespiralis ATCC 25486

MTVSPRVAVDPFGADIIAECAKLRSLGPIVPVELPGGIPAWAPTGHDTLK

ALILDPEVSKDPRRHWSLFPEIGEHPEWGWILGWVGVVNMLSTYGSDHTR

LRKLVAPSFTARRTEAMRDRVEAITEELLTELDAAGANGEVVDVKAAFAH

PLPMRMICELFGVPEHLREATGRLIAALMDTSDPSPEQAAWVQEQIGTVL

GSLIGYKAEHPGDDMTTELIRVRDEDGDRLSDEELLYTLLLVIGAGFETT

VNLIGNAVVALLEHPEQLAAVRAGAISWEQVIEEVLRVRPSIASLPLRFA

VSDITVGGVTVPAGDAILTTYAAAGNDPARYGEDAAAFDATRAADDHLSF

GIGVHRCIGAPLARMEAMTALPALFDRYPGLTAAFESDELKQVPSFIAYG

WQEIPVRLQG

>CYP156H(648864934)Streptomyces pristinaespiralis ATCC 25486

MNAYPARADAFTLSAPVRLWEEGFAADPHAYYAALRAQGPVGWAELAPGV

PAYVVTDRRAALDLLHDPATWSHDPRPWEATVPEDSPVLGMMRWRPNTLF

ADGDAHIRYRRSLTDAFDLVEPHDLRERVHHAVDVLVGRFGPAGSADLVT

EFARPLMALIFNNLFGLPDSESGRLDAALGAMMEGGDRAAEGEAEYGRYV

LELIAAKSVRRGPDITSRLLDHPLALTPEEVTWQVFLTLGAGHEPSANLV

SNALSRILGNADYYSTLTSGSRPVMDAVLEVLRYETPLSNYGIHFARESV

SFHGVWVQAAVPVVVSYGALGHFAEQDFAGTHHRHDASHLSFSAGEHACP

VKQPALLIATEAIERLTQWLPDLQPVLPRERLSWRPGPFHRSLTALPVRF

TPHSPDQAGDRS

>CYP107U(648865109)Streptomyces pristinaespiralis ATCC 25486

MNDPTDRNAAPELFTWEFATDPYPAYAWLREHSPVHRTRLPSGVEAWLVT

RYADAKQALADQRLSKNPAHHDEPAHAKGKTGIPGERKAELMTHLLNIDP

PDHTRLRRLVSKAFTPRRVAEFAPRVQELTDRLIDSFAQKGEADLIHEFA

FPLPIYAICDMLGVPREDQDDFRDWAGMMIRHGGGPRGGVARSVKKMRGY

LAELIHRKREEPGDDLISGLIKASDHGEHLTENEAAAMAFILLFAGFETT

VNLIGNGVYALLRNPGQRERLQASLAAGESELLATGVEELLRYDGPVELA

TWRFATEPLTVGGQRIGAGDPVLVVLAAADRDPERFDAPDTLDLSRRDNQ

HLGYGHGIHYCLGAPLARLEGQTALGTLLRRMPDLRLAGDPADLRWRGGL

IMRGLRTLPVEFTPQTN

>CYP125A(648865800)Streptomyces pristinaespiralis ATCC 25486

MSCPHLPPGFDFTDPDLLQARVPHPEFAEMRRTAPVWWCEQPAGISGFDD

EGYWVVTRHADVRHVSTHPELFSSWTNTAVIRFNESISRDQIEVQKLIML

NMDPPEHTRVRQIVQRGFTPRAVRSLEAALRRRARSIVETARSGARPDGS

FDFVTNIAVELPLQAIAELIGVPQEDRSKIFDWSNKMAAYDDPEYAITEE

VGTEAAMEIVSYAMNLAAARKECPAGDIVSRLVAAEDEGNLSSDEFGFFV

ILLAVAGNETTRNAISHGMHAFLTHPEQWELYKRLRPDTTAEEIVRWATP

VVSFQRTATQDVVLGGARIKKGDRVGLFYSSANNDPTVFDHPERFDITRD

PNPHLGFGGGGPHFCLGKSLAVMEINLIFDAIADALPDLRLAGDPRRLRS

AWLNGIKELRVTTG

>CYP159A(648866863)Streptomyces pristinaespiralis ATCC 25486

MATAQHIPDILSPEFAADPYPAYRSMREHAPLLWHEPTGSYLLSRYEDVE

RAFKDKESVFTTENYDWQLEPVHGRTIVQLSGREHAVRRALVAPAFRGSD

LRDKFLPVIESNSRELIDAFRHKGEVDLVDSFATRFPVNVIADMLGLDKA

DHDRFHGWYTSVVAYFGNLARDPDVAAAGERTRVEFTEYMIPIIQERREN

LGEDLLSTLCAAEVDGVRMSDEDIKAFCSLLLAAGGETTDKAIAGMFANL

LVHPDQMAAVREDRSLIDRALAETLRFTPPVHMIMRQTAAEVTVSGGTIP

AGATVTCLIGAANRDEERYRDPDRFDIFRDDLTATTAFSAAADHLAFALG

RHFCVGALLARAEVRTGVGQLLDAMPDLRLADGFVPREQGVFTRGPRSLP

VRFTPAGG

>CYP157B(648866865)Streptomyces pristinaespiralis ATCC 25486

MNDPIGTAAHVPHPPGCPAHEEAVRLSGPEYRQSPSELYRSLRGRYGSVA

PVLLDADIPAWLVLGYTEVTYVTAHDELFARDSRRWNQWGAVPPDWPLLP

FVGHQPSVLFTEGAEHQRRARVVTEALEAVDQFELAHWCREIAEQLINAF

AGSGRAELMTAYAHALPMRAAVRMCGMPAHGADTEGLVRDLRISLDAAGN

DAPVAAYVRVQERIQRLVEDKRGAPGPDVTSRMLAHPAALTDEEAVQDLI

VVMAATQQPTANWICNTLRLLLTDDRFALNVSGGRLSVGQALNEVLWLDT

PTQNVIGRWAVRDTTLGGRRIRAGDCLMLGLAAANTDPRIWPEGHISAEN

SAHLSFGNGEHRCPYPAPLLADVIARTAVETLLERLPDVVLAVDPDELTW

RPSVWMRGLTSLPVSFTPVVDLGEYRNGSPR

>CYP251A(648867378)Streptomyces pristinaespiralis ATCC 25486

MPSAGGTVSKGKPVSLAAIPRAKGSIPGLGHLPRLARDPLAVLRSLHAEG

PVLRLDVGPVPVVVVTSAAAVNDLMVKQARSFVKGRLFDRVRPLVGNGLA

NSDEPQHMRHRRLMQPMFYKERLAGYAEVMSERADRLAGSWTADQKFDVN

EVMTTFAIETLAATLFSADIGRSAVDAVREDLPIILRNMILRALAPQFTD

SWPIWKKFDTAAARMRAVIDEVITATRASAPAGRTDLLSLLLSARDDTGE

GLTDEQVRDELTTMLFAGSETVASTLSWALHHLAQHPDVEQQLLAEIDQV

VGDGKVTFAHVTQLPSITRVLDEAIRLHGVVTLMRRTTEPVSIGGYELPA

ETEVLMSLYALHRNPDLYPDPDRFDPDRWLPEQVAARPREHVVPFGAGNR

KCIGDRYAWMEATIALATILPRWKLRPVPGSKSPREATAAMAHPTRMPMV

VLPR

>CYP157C(648867379)Streptomyces pristinaespiralis ATCC 25486

MSRHPSLPGEGLVPPPGCPAHGMNDAGLYRLPEAQNPSALYEQLRQQYGP

VAPVLIHGDLPAWIVLGYRENLDVMRTPSRFSRDSRRWTAFKENRVAADS

PLIPMVAWQPLCVFADGEEHARQRAAVVDGLAQFNRRGMRRYVTHYTREL

VAGFAASGTADLVRDYAEPLPMLVVSRLLGIDPQDGPALVEPTLDLMRGS

ATAAASNQIVTQTLEELVARKKLHPGNDLASRLISHESRLTDTEIVEHLR

LVLVAAHQGTVNLIAHTLRLVLTDRRFRGHLSGGQMTLPDALEQVMWDNP

SIGIVPGRWATRDTMLGGQHIKEGDMLLLCISAANVDPEQRPDLSVPMRG

NRSHLALSSGPHECPGGDIGRAIADTGIDELVALLPDLRLAVPETEIGIT

ANWLTSRPSSLPVRFSRPRSLGGTEVTAPEPATGAQPLPVAASAPPAVPA

HTPSIPKQSWWRSLLGR

>CYP152D(648867483)Streptomyces pristinaespiralis ATCC 25486

MELREILRPDATLPALLEGYAWLPDLVRRSGGAPVRTRLMGRPAIALHGL

DAVPFFYDEQHIARRSALPGPVLDTLFGRGAVHTLDGPAHRARKALFVSL

LKDPEGVDGLAGRAVKEWETAVAEWAHRPRIELFTEASVLITRAVCDWAG

VPRGEDDGRATARDLVAMVDGFATAGPRHRRARSARTRQEARLRALIEDI

RGSGTEHSGSALHAVALHRDDDGLLDAHTAAVEVLNIIRPTAAVAWFMAF

AAHAMHRWPRQRELLREGGPADAEAFAHEVRRFYPFVPFLGGVAVRDLEW

RGERIAEGTLVLLDVYGHHHDPAIWPDPYVFEPARFTDGGPAGSDLLIPQ

GGGDTATGHRCPGEDVTVALLKALAPRLARLEHGVPPQDLSIPLRRVPTR

PVDGLPAGRPAGAHRLNGSGAADPGVRSDARRARSSVALGRGAGQRRRDV

LQRFAFGRDGEEDGHQAGGDHQARADAEREDGVGDDAARHLGVGDGESTA

LRGEPAGDVADAGQRAAPRRGVLPHSQSRSADSRGRLSRRSRV

>CYP154B(648867629)Streptomyces pristinaespiralis ATCC 25486

MEIDTDVDVDVDVDVESGGESVGCPYALDVTGRDLAGEAAMLRERGPAAR

VMLPGGVRAWAVTGHEPLKRLLTDRRVSKDAGRHWPAFVEGRITPQWPLY

HWVSAPTMLFAYGEEHTRLRRLVAGAFTARRSRELAPRIERITAALLDEM

TPTAPTGADGGEGVVDLRTAFAKLLPMRVICELFGVEGPDRQALCAAIDT

TLGTSVPADEMARAQDRVTELLAALVARRRAEPGPDLTSALIAARDGGER

LSEQELISTLNLMIGAGQETTSNLISNAVAALLSRPAQLEHLRAGRADWP

DVIAETLRTHNPAAYIPLRYAVEDIDLDGVLIRKGDVIIVSFAAAGLDPA

RHGKDADTFDLLRPHGESVAFGHGVHYCLGAPLARLEATIALSALFARFP

GIRLARPVGDLQPLESFIISGYRTLPVHLGPPTTT

>CYP113C(648867638)Streptomyces pristinaespiralis ATCC 25486

MPPTPRPTTDDGGRELLAWLREMRHHHPVHEDEYGAFHVFRHADVLTVAS

DPGVYSSQLSRLRPGSQALSEQILSVIDPPMHRTLRRLVSQAFTPRTVAD

LEPRVTELAGQLLDAVDGDTFDLVADFAYPLPVIVIAELLGVPPADRTLF

RSWSDRMLQMQVADPADMQFGDDADEDYQRLVKEPMRAMHAYLHDHVTDR

RARPANDLISALVAARVEGERLTDEQIVEFGALLLMAGHVSTSMLLGNTV

LCLKDHPRAEAAARADRSLIPALIEEVLRLRPPITVMARVTTKDTVLAGT

TIPAGRMVVPSLLSANHDEQVFTDPDHLDLAREGRQIAFGHGIHYCLGAP

LARLEGRIALEALFDRFPDFSPTDGAKLRYHRDGLFGVKNLPLTVRRG

>CYP107EH(648867672)Streptomyces pristinaespiralis ATCC 25486

MTTTAPRPAAPAPATAGHTAPEPAAAGQADTCPAAAGHAPAFPFDTEPGP

PLEVPQEFTRLRDRCPVQRVRLPYGGEGYLVSRYADVKQVLADRRFSRAA

TVGKDVPRTVREVTAPGHLLAMDPPEHTRLRRLVAAAFTERSIQRRRPRI

QQIVDDLVDGLRARAAGGEAVDLVAHFSMPLPMTVICELLGVPPGDRHYF

QRVAEVAFSNGAVDPQEMQQVGEAFGTYLTGHIARLRERPGDDLMSQMIA

ARDEDQDRLTEAELISLAGTLLLAGYETTAAEISNFVYTLLEHDRWSWLV

DHPEHLDTAVEELLRYIALGGGDVLPRLATEDIDVGGTVLPEGASVIAAM

ISANRDGHVFPDPDVLDLERSPNPHVAFGHGPHHCTGAQLARLELRVALA

SLLRAFPALRLAVPAAQVPWRQGSLVRGPRQLLLRW

>CYP154A(648867808)Streptomyces pristinaespiralis ATCC 25486

MPAAPGAATATAPPGPRPFVLDPAGRDLHGEAARLRALGPAVLVELPAGI

RAWSINTHDLLKTLLTDDRVSKDPRRHWPAWQRGEHHDTWIRTWVGVHNM

DTAYGPEHRRLRKLVAPTFTGRRTRAMLPRVRHAADRLLDALADTAPGDI

VDLRAAYAHPLPMQVICDLFGVPETSRPRLARLMARAMDTTLTPDEAEQT

VHEVDDALTALVAHRRSHPGDDLTSALVAARDDDGSHLSERELLDTLLLV

IGAGNETTVNLIGNAVHALLTHPDQLRLVLDGTVSWHDAIEETLRWAPSV

ANVPLRYAVEDIALPGGPTIRKGEAILAAYAAAGRDPDRHGPTADRFDIT

RPGRGAAEHLAFGHGVHFCPGAPLARTEAAVALPALFDRFPGIHLAAAPG

GPAPTEGFIAYGHHTLPVRLTALHPAARNPRLRLEEPGAARNGSV

>CYP1047A(2526251739)Streptomyces sp. LaPpAH-201

MSADTGAREQGRQGEVPVVVPGPKGVPLLGSLPEFGKDPLAFFERLRGHG

DVVSWNFGGKPSLFIGDPDLVGELLREVESTFDQPDLGVAFRAVLGNGVT

VARGRDWRRKRSLVQPSVRPKQVKSYAATMASCAVDTADGWRDGQRIDIK

REMAALTQRIAVRTIFGTDAEGDVEAIGRAMDIAQREIGAEFSGIGAVLP

DWVPTPGRRRVQKAAAVIDREVGRVVAAHREDGERPDLLSRLLTAQDETG

NRLSEEEIRDETVTLYIGGHETTSSTLVWAWYLLARNPRVRAALDEELDR

VLGDREPGFDDFARLPYAQAVVQETLRLYPILWLLTGIAKEGASLGGLPV

APGTRVWTSQWAVHRDPRWYGDAEVFRPERWLEGAEESIPEYAWFPFGGG

PRVCIGARFATVEAVLILAVLGRRYDLDVDPGEIRPMTTLTLQPDRDMLA

TVRARGGGA

>CYP157C(2526251744)Streptomyces sp. LaPpAH-201

MTTPKAPHSFPGTPAGPPPGCPAHGADPVGPFGAGGLRRLYGPEAERDAP

GLFEKLRAEHGPVAPALLHDDVPIWVVLGHSENLHMLRTHSVYTRNSRRW

RLVQDGTLGPDYPLAPLFAWQPICSFAEGAERERLRGAVNNAMQQIDYRG

VRRAINRHSNRLVNEFGQDGRADLVSQFTDHLPMLVMLDVLGLPEEYNEQ

MVDAARDMLQGTETANASNAAIMGILERHVARRRAQPDDDFTSSLLEDEA

RLTDDEVAQHLRLVLIAAYEATSNLTANVLRMVLTDPRFRAQLNGGQMTV

PEAVEQTLWDQPPFSNMLGYFAVQDAELGGQQIRKGDALLLNIAAANVDP

VVRPDLEANMQGNRAHLAFGGGVYECPGDDLGRAIADTGVDALLMRLPDV

ELAVPETDLHWTNSLISSHLKELPVVFSPRQPLELSSSPGQTGASRTDWE

ISSPAPRPPAAGPVPPQAAPHVPAPPAADGPVATVPAQRRGAWRRLVEWV

RGG

>CYP105DT1(2526251950)Streptomyces sp. LaPpAH-201

MSRPSAVGLPTGTEPLPDFPQLRQCPYQPPPGYRDLRLTEGPVVRARLYD

GRPTWVVLGHAVARELLIDPRLSSDWSRPDFPSPSPRRKAIQKATILVGM

DPPEHSAYRRKLIPAFSVRRTRELGESIHRRAGELVDAILQRGTGEILHD

LALPLSSHTICGILGVPYEDHAYFEEQSALLVSPQVTEEDATGALMNLRS

YLTGLVERKEKEPEPGDGLLDTLVHKDLAEGSLSRDDVIRLGIILLMAGH

ETTASMITLSTFTLLQNPAQLAAFRDDPAGAGLAVEELLRYLSIGDVAMR

IAAEDITVGDAHIRAGDSVMLSTAEVNRDPDAFDEPDALELSRGARHHLA

FGYGVHQCIGQNLARAEMESALTLLFGRIPGLRLAVPAEEVEIKPAQSGV

QGIYDLPVTW

>CYP107L(2526253693)Streptomyces sp. LaPpAH-201

MTDVVDLAAYGERFTADPYPVYAELRERGPVHRVRLPRADGPVEAWLVVG

YEEAREALADPRLSKDPTTLGITIPEGELIGRHMLVADPPEHTRLRRLVA

REFTARRVQALAPLVQRITDELLDAMVPLGRADLVQSFAFPLPLTVISEL

LGVPVADRAAFRRMSGEVVAPSGKVPPEANLAELGTFLDALIEEKRRSGT

TGDLVGDLIRTADDEGDQLSSSELRAMAYLLLIAGHETTVNLIASGVHTL

LRHPDQLAALRADPTLTEGAVEEILRYEGPIETATWRHTAEEVEFGGVRM

GKNEPVLVSLASAGHDPARFPDPERFDIRRRTQGHLAFGHGLHFCLGAPL

ARLEGRIALTSLLARCPELAADGEPSGWAPGLLIRGVQDLPLRW

>CYP107BX(25262540480)Streptomyces sp. LaPpAH-201

MSAATPAPAGAVPPLAPLHRRAPAEPGPPRPCTLPDGSPGWLVDRYADVR

QVLSDSRFGRAGLYLQDGPSRSQAAGLVDDPELMFNQDGVEHLRLRRTLR

RAFTPRAVARWRPWIASIVDQLLDDLSARSGPVDAVAEFTLPLPVAVISR

LMGLDASVRGRMRHWSEHAFSDGTRPKDEVDAALAEFTAFGARLLAQRRR

APGDDLVSSLVRAADAEGGIPEDRLVSLVCGLVAGGHDSTMTMLGNSLLY

LLAERPEEWPRLADEPSAELAAARLIHLIPLGDDPGSTRCATEDAEVGGV

LIPAGAVVLADSTTANRDPSVFPAAQTEALFTPLPAPTLAFGAGPHYCLG

AWLARLELHLALHRLAVRFPGLRLAEPEKPVRWRPAGTSRSPERLPVTW

>CYP154D(2526254308)Streptomyces sp. LaPpAH-201

MTTDPAPAQDPPPAPGTYRMRPGGGCPHADNAALLARGPVARVVLPGEIA

GMAVLGHAELREFLAHPDVAKDPRHFTALREGGVPDGWPLRTFATVPGMT

TADGADHRRLRGPVAAAFTPSRVEALRPRITTLVDTLLDEVEEAARADGT

ADLRRLFALPLPMAVIGELLGVDPAHHERLHALSERVVATGAGPEQTLAA

NRALFAVLGEVAAARAADPGDDLTSALIAARDEGGGRLDDAEVIGTLLLM

VVAGHETTLNLVTNAVRALCAHRDQLRLVLDGRATWAQAVEETLRWDSPV

SAFPFRYATRDLTLGSTVIPAGLPVLAGYSAAGRDRAAHGPDADRFDVTR

AGPRHLSLGHGPHYCLGAPLARLEATVALERLFGRFPGLTLAEPEETLPP

AAGFVGNSVRRLPVRLR

>CYP170B(2526254886)Streptomyces sp. LaPpAH-201

MSAESTTERPAAPGPRTPPLVAGSVPVLGHAPSLVRDPLAFLTGLRDHGD

LVRVKLGPKTAYAVCAPELVGALLRNSQEFQVGGPLWENLEVLLGKGVAT

SNGADHRRQRRMMQPAFRPERIASYATVMEEEARAMADRWQDGQVVDIGA

ETFSCAVRVVARSLLEVDSIGAKADRISASLHTVFSGLYRRMILSFGPLH

RLPTPANRRFERALADLHHLVDEVIAERHAQGKGADDLLDILLTSEDGEG

RPLGDQEIHDHLVSLIVAGAENVASTLAWCFQLLTEHPAEEQRVAEEALS

VAPDRPVNFGDLGSLDHTRNVIVESMRIRPAAWIFTRRAVADTELGGYRI

PAGADIIYSAYAMQHDPRSFDRPDVFDPDRWIPERAEKVPQYAMMPFSTG

NRKCPGDHFSMVEATLMLATVLPRWRLVPVPETDPAPRIGITLQPKRAVF

RVESR

>CYP107AM(2526255482)Streptomyces sp. LaPpAH-201

MARSADTPPLDYPLTSKAALEPPEEWAGLREGCPVARVTLPSGDEATLLT

RYDDVRAALSDPRLSREGLASPDAARVSAGDTAGIFASPMARALNDEGHE

RWRRMVGRWFTARRMSALRPGMEELAGRLIGRMREHGGPADLVTHLAFPL

PVLVICSMLGVPESDRDAFKGWSDTFLNTTRYTKAETEAAHRDFAAYMSG

LVDAKRAAPGDDLLSHLLAGADSEGEPMSEAGLVATGQALLLAGHETTAG

FIAMMTAHLLSDRTRWERLVADPSLVRRSVEELLRFDPNGSGFGMLRYVH

EDTEFSGGTVPRGTTVVCSMAAPNRDERAWQDAAAMDLDRSPNPHLAFGV

GPHSCLGQPLARTELQAVLTVLLRELPTLRLAVDPRELRRHEGLLTSPLR

ELPVTW

>CYP107F(2526255585)Streptomyces sp. LaPpAH-201

MSTEAVPAAPDSAEVPTCPFDFAQGLDFDPALLAMLRSEERVARVRMPYG

EGDAWLVTRYEDVRTVTTDRRFSRNAVTGKDFPRMTPEPIVQTGAINLMD

PPESSRLRRLVRQSFAPRHLERMRGRTQSVVDGLLDAMAESGSPTDLFAH

LALPLPLVTICEVLDIPEADRHWLRAHAMTMMNMKPAGKEAAVRAKGELR

EYFARLTAERRRSPGADMISTLATAREGGEMLGEDELTVMAMVLLITGQD

TTTYQLGNISYLLLTRDDVRAQLRREPDSLPRVLEELLRHIPFRKGVGIP

RIATEDVELGGALIRAGDTVHVSYLTANRDGEKFERPDEIDLDRPSVPHM

TFGWGSHHCLGSPLAVMELEIALSTLLRRFPDLALAVEPGEVEWNATSIW

RYPLALPVTW

>CYP105H(2526255657)Streptomyces sp. LaPpAH-201

MTTSPGPTVVDFPRRTPREPLPLSQYAEHRKQNGLVQTHLPNGRPIWLVT

RHEDVRAVLTHPRISANPDNEGFPNVGETMGVPKQEQIPGWFVGLDSPEH

DRFRKVLIPEFTVRRVRELRPAIERTVDERIDAMLAGGNTADLVNDFALP

VPSLVISALLGVPSADRDFFESRTRTLVAIRTSTDEERAEATRQLLRYIN

RLIVIKKKWRGEDLISRLLSTGKLSDEELSGVLLLLLIAGHETTANNIGL

GVVTLLSHREWIGDDRLVEELLRLHSVADMVALRVAVDDVEIAGQTIRKG

EGIVPLLASANHDTEAFGCPHAFNPERTERRHVAFGYGVHQCLGQNLVRV

EMEIAYRKLFERIPELRLAVPEDQLAYKYDGILFGLHELPVRW

>CYP105AK(2526255947)Streptomyces sp. LaPpAH-201

MPIATPIRTVRTARSSIAGWLTRRYLSRLRRKGTTLDLNALSKLPEPALL

PLRRNGLDPVPEIGALRDREPVSRLPVPAVPVWLVTGYDEAKEVLGDARA

FSNDFAHLVGTNGVAEHHEPGGLGFADPPDHTRLRRLLTPEFTMRRLNRL

TPLIHSIIEERLDALEAAADADGRVDLVEHFALPVPALVICELLGVPYEE

RDAFQQFSVARFDVLGGLGASFGAISQSREYLRGVIAQQRREPGDGLLGM

IIREHGDAVTDEELTGLADGVLTGGLETTASMLALGTLVMLQDRTHFTAI

REAEDPGAVATPFVDELLRHLTVVQTAFPRFARENTVVGGQAISAGDIVI

VSLSAADRDKRLGPEMDAFDPSRPPASSHLAFGYGIHRCVGAELGRMELR

AAFPLLVERFPALRLAVEPQELEFRKLSIVYGVDSLPVRLK

>CYP146A(2526256485)Streptomyces sp. LaPpAH-201

MTIDLANPDLYTTDARFEMWEEYIRSDAKVWSDPGISPSGFWSVFSHRDV

SAVLSPKAPFTSEYGMMIGFDAEHADHSGGRMLVVSEGNWHSVLKRLIGP

FLSRLRAPELRSVLHEEVREIVERLRSQETTDIALTVGPRLPAAIVCEVI

GVPLSEREHLIQLTNHAFGGEESSFDKMTPAEAHTEILFYFHELIERRQK

EPGTDLVSALLADGRLSTEDVLINCDNVLIGGNETTRHSITGAFHAFQAF

PGALDTLRADPELADRAVEEVVRWTSPAAHVLRVATEDCEIGGRQIREGQ

AVVAWLAAANRDERLFAHPHRFVVDRAPNRHLGFGTGPHHCLGAALARLE

LKELLTQLAAEAKAVEPREEVRWMRSNLVQGYSGLEVSMRWR

>CYP157J(2526256658)Streptomyces sp. LaPpAH-201

MTAAPPPGCPAHASTGLVPLTAATGAPDHREVYRRLRAEWGDVARVELEP

GVPAWLVMGYREMLTLTRNEQLFSRDARNWRDLREGVVSPDSGLLPMMGW

RANVIGADGPEHRRLRAPLDDGVARIDQRRARRQVEALCEELIAEFAARG

SADLVGEYATIVPMLALASLFGLDSAGGHELLRALIALFGSADDSQAGNR

QFEEILLDTLRERRARPADDLTTAFLDHPDLHNEAEHLQSVVVMISAGNE

TVTAWIAHTLRLLLCDPRFASRLHGGRLGIDDALDEALWRDPPMNNMPAR

YALRDTELGGHRIRQGDCLILGIAAANDDPLIRPEDELTEPGNRAHLAFS

AGPHVCPAQVPARLITRTAVKTVLHRLPGLRLTVPAEEIGWRPSPWTRVP

TALPVAFPPAPRPSKENR

>CYP154T(2526256659)Streptomyces sp. LaPpAH-201

MTTTPDAAVPEITLDPYGSDHHGEAARLRALGPVVRVRLPGDVRAWAVTR

HDLLAELVADPRMSKDWRNWNAIRRGEIEDGWPLIGMVKVTNMVTADGQE

HRRLRKLVMQTFTPRRVAELRPRVEAIVAGLLDELPSHADADGVVDLRRH

YANPVPMRVICELFGVPGHEQPRLKELMDNIFRSDLGPEEVTASQVEQYQ

LLARVVATRRAEPGPDLTSALIAAREADPDALSEEELVGTLLLMLSAGQE

TTLSLVTNAVRALLTHPDQRALAEAGGEEVWEAVVEETLRWDAPIGNFPF

RYPLEDVEIAGVPIGRGEAIMAPYSAVGRDRAQHGEDADRFDLTREQNRH

LAFSHGPHFCLGAPLARLEAALAIPAVFARYPGLSLAVDPATLTPVPSMF

SNSSATLPVRLAHDT

>CYP107U(2526256828)Streptomyces sp. LaPpAH-201

MSETQAAGRPELFGWEFAADPYPAYAWLRTHEPVHRTRLPSGVEAWLVTR

YADARQTLADPRLSKNPVHHAADEAGRSRTGIPGERSAGLMTHLLNIDPP

DHTRLRRLVSKAFTPRRVAQFAPRVQELTDGLIDGFAGRGEADLIHEFAF

PLPIYAICDLLGVPREDQDDFRDWAGMMIRHGGGPRGGVARSVKKMRNYL

AELIHRKRADLGDDLISGLIRASDHGEHLTEEEAAAMCFVLLFAGFETTV

NLIGNGTYALLRNPAERARLQRALAEGDEALLDTGIEELLRYDGPVEMAT

WRYATEPLRIGGADIAAGDPVLVVLAAADRDPARFDGPDRLDLARTDNQH

LGYGHGIHYCIGAPLARLEGKAALATLLTRLPDLRLGTEPEELRWRGGLI

MRGLRTLPVQFTPEPDQGAKKV

>CYP157A(2526257112)Streptomyces sp. LaPpAH-201

MTPPDTPSATTDAPEAAAPAAPATGGCPVAHGAGGAEPAPLLLGGDRFQS

DPIGLYRDLRRDHGPVAPVVLPGKLPAWLVIGYRELHQVTSDPVLYSRDS

DLWNQWDRVPENWPLLPMIGKQESILYTVGERHRRRAAVMEGGLEAVEAH

ELRATTERLADSLIDDFCGSGDADVIADYAMMLPALVLFQLFGLPESEGR

GLAHAINDMINGGERALAGQQHIRECVGRMVADLHVHPRDHVTSRMLRLG

GHLPGEDRAVITDEFSAAEIIQDVLVMVVAGHQPTADWIGNTLRLMLTDT

RFAASLFGGRHSIAEAMNEVLWEDTPSQNIAGRWATRDTHIGGRRIREGD

LLILSFAGANYDPLVRTDQSALTGGNNAFFSFGHGEHRCPFPAREIAEIV

ARTAIEVILDRLPDIDLAVAAEELTRRPSPWLRGLTRLPVRFTPVPAG

>CYP154C(2526257113)Streptomyces sp. LaPpAH-201

MSLSETSATQTPATPPPGCPAHAHGAVDPEAYIALDPLVRDLDGESARLR

AAGPLAKVMLIGDVPVYSVTHHAEARKLLTDSRLVKDINHWNAWNNGEIP

ADWPLMGLANPGRSMLTADGPEHRRLRNFVAQALTVRRVQKLRPGIEALC

ARMLDTMEQAADENGVVDLKAHYAHPVPMTVITELFGMPSHHIPRLKELF

DIFFSTVVPREQVPPMMAELDDIFQAFVQSKRDEPGDDLTSGLLEAAADG

DTLTNEEIVNTLKIIVTAGHETTISLIVNAVRALSAHPEQRARVLAGEIP

WSQVIEETLRYNTPTSHLLIRFPTEDIEVGDQILPKGEGLIVSFSAIGRD

ENQHGPTAGAFDATRDPIRHIAFGHGPHVCPGASLSRVEAEVALPALYAR

FPELTLAVDDSELRNKPILTQNDLFDLPVRLHG

>CYP154A(2526257143)Streptomyces sp. LaPpAH-201

MPSSAPPEQEPLVLDPTGADPHAEHRALHARGPATRVDVLGVPAWSVSDP

VLLRALLTSPDVSKDGRAHWPAFAETVQSWPLALWIAVRNMFTSYGADHR

RLRRIIAPSFSARRVAAVRPVVEWITAGLLDDLAALPPDAPVDLREKLAY

PLPVAVIGHLMGVPEERYAHFRAVVDGVFDTTLGREEAAANTGALYVVLD

ELIATRRAEPGDDLTSLLLATPDEEGAEPALSHEELRDTLLLVISAGYET

TVNVIDQAVHALLTVDGQLDLVRSGQVGWGDVVEETLRHESAVKHLPLRY

AVRDIPLPDGRTIARGEAILASYAAANRYPGWHDDADVFDAGRLSKEHLA

FGHGVHFCVGAPLARLEVEVCLRLLFARFPGLALDVPAEGLPPFPSLISN

GHRNLPVWLHGGALGEGPG

>CYP156B(2526257164)Streptomyces sp. LaPpAH-201

MESHPPYASATGRCPMHDANFAADPQALYEQLREQGPAGPVELAPGVDAT

LVVGYETALRVLQNPTSFPRDARRWAALNEGRVPMDSPVLPMMAYRPNAL

FTDGAVHLRLRKAVTDSLAKLNITRIRRDVEPIADYLIDQFSERGRADLL

AEYAKLLPLLLFNKLFGCPADIGDTLTTEMSAMFDGKDPVRTKARLDACL

MQLIAIKRREPADDVISWLIQHPAGLTDEELKDQLVMLMGAGIEPERNLI

GNALLELLSGGAGGRGAGMMVEEAVDHVLWHRTPIANYAAHYPAQDTDFG

NGVIAPAGSPVLISFAGANSDPALAEARRSAGRGAHLAWGAGSHACPAKD

PAQVIAVTAVEKILNALPDLTLGVPEEDLQWRPGPFHRALVSLPVAFSPT

PATRMAAALQNRGIPQQQAAPATPQPQHTAPASSDGGGRRKGFWSSFLDI

FRV

>CYP107CJ(2546572351)Streptomyces albulus CCRC 11814

MTIQEPPLPAAEQKPRPQPLPDPVPLTGCPYKSNPYPLYERMREAGPVHR

VLFPSGVQAWLVTGYDAAHAALNDDRLGKNHDRGNERWRARASIMPEPQH

SQLQVHLLHQDPPRHTRMRRFVTDAFTPRRIESLRPRFQELADALIDALP

ESGPADLVAGFAAHFPFQVLAEVIGLPHHLAARFDRDWGKVVQPVGPTDP

GRPLYEARLHGLQSYIAEVVAHKREHWDDDLLSRLVVARDRRELSQEELD

SMIFQLLVAGQEPVTNQITTALIALFRHPDQLARLRDEPDLLPRAVEELL

RYDSAFELTTWRFLDQDDNLHGTDIPAGDSVIVSLCAANRDPRRFPDPDS

LDLDRTPNPHLAFGHGIHFCPGAALARAELQVALDALLTRLPGLHLAIRD

EDIEWIPAVLGRGTNHLPVGYDRRR

>CYP107EB(2546572472)Streptomyces albulus CCRC 11814

MTTPSSGPNIMDPALIADPYGGFDRLREEAPLVLGRSADSTPTWYATRYD

DVRAVLADPRFVVDPELTPGTEAVDNRNRMLDMLELPKEFHPYLSESILD

VDGDRHTRLRGLATRAFTARRVNALRPRVEAITASLLDGFGESVELISEF

AYPLPIAVICELVGIPEEDRGLWRTAGSALTSIAPGSKGAAAHELIAYTH

ALVDQRRAAPADDLISDLVKVQDEDGDRLSDVELVTMLLGLATAGHETTA

NLVGNGALALLSHPDQLEALRRTPELWPTAVDELVRSCGSILITQLRYAT

EDIDVGGQTIKAGEAVQPVVVSANRDPREFSRPECLDVTRRSVKPGDGHV

GFGLGAHYCLGAALARQECEVALRGLFDRFPRVALTTDEHTWVPVPGLRQ

LASLPLTLY

>CYP163B(2546572543)Streptomyces albulus CCRC 11814

MTVTTPALDVVDLGDPATFADHDLDAFWRTLRDTYPVYWNPPLDGRRGFW

VLSRYDDIMAAYRDDVHFTSERGNVLVTLLGGGDAGAGRMLAVTDGHRHH

ELRKILQRVLSPRVLSEVAAAVRVNTRQLIREAVEAGGCDFAEQIASRIP

MTTISNLLGVPEQDRDHLLAQTKAALSTDAEDVDEVDSEMARNEILMYFM

DMVEERRESPGDDVISMLVASSIDGVPLSDEDIVLNCYSLIIGGDETSRL

TMIDGVHTLAAQPEQWRRLKHGEVAIDTAVDEVLRWASPTMHFGRSVVGE

TELHGVQLRPGEIVTLWHASGNRDERIFDRPGTFDLGRTPNKHLAFGYGP

HFCIGSYLAKVEIAELLMALRDFTTGFETTGEALRIRSNFLTGFSSLPVR

WRPDHSGMKEVD

>CYP105AA(2546572553)Streptomyces albulus CCRC 11814

MRQTVPVPHGLPMDRDANPFDPPRALTRLRAARPVSPLVFPDGHEGWLVT

GYEAVRQLMADTRFSSRLDLGVVHVPYETPGMPVPTEPSPQIPGVFIAMD

PPDHTRLRRRLTGAFTVKRMKQLEEHIVEVTERQLDAMARLAPPVDLVRE

FALPVPSLVICELLGVPYEDRDTFQSNSAKFLVKEQTLEEKMAAYGALTT

YLAELVTSKRAAPGEDILSDLARHDDLTVEELTGIAFLLLLAGHETTANM

LALGTFALLEHPDQLAELRADPDLLPDAVEELLRYLAVGDVFYRYATEDI

ELGGETIGKGSTVVVSLLAANHDPRRFEHPDTLDVHRKARGHLSFGHGVH

QCLGQQLARVEMRAGFAGLLRRFPTLELAIPANEVKLRTDMNIYGVHELP

VTWTETAG

>CYP154A(2546572616)Streptomyces albulus CCRC 11814

MPQQSPHVLDPAGRARRTEDASLRARGPMARVDVLGEEAWAVTDPELLRS

LLLDDRVSKDPHRHWDRFPDRTGDWPLNLWVAVENMFTAYGAEHRRLRRL

IAPAFAARTITALEPDIERFTRELLDDLATARPGEAVDLRERFAAPLPIR

VITHLMGLPAHLLADFRRAVNGVFATDVTAEAAAANARDLYAALDALLAL

KQERPGDDLTTRLLQARDPEGQGHGLTPQEVRDTLLLVISAGYETTVNLI

DQALVALLSHLEHLAAARSGALPWGDVVEETLRWQAPVPLLPMRFATTDI

PVADGIVIRKGQAILAAYSAANRHPALHGPDADRFDPTRPDKTHLSFGHG

VHLCLGATLARLEATTALRMLTARFPELRLAVPADALVPLPSFLTNGHVN

LPALVGPEAGA

>CYP107EC(2546572624)Streptomyces albulus CCRC 11814

MTNPLFKANPHDFYAGLRESGPAHMVHLPGYGDVWLVTGYADARAALADP

GLSKAPANVPASLRNAMVEAGERDGFQLMAHMLNSDPPDHTRLRKLVVRT

FTARRVRALRPRIQEITDALLDEVVAKGSADLMDDLAFPLPITVICELLG

VPAEDRDDFRRWTSILVSEDEALRPQLQDTFAQLNAYLTALVRHKHAAPD

DGLLSALVSVAEDGDSLDDDEVVWMAFLLLVAGHETTENLIGNGMLALLS

NPEQLAALRADASLLPTAVEEMLRYEGPVETTTWRFTTRPVEIGGVTIPA

DQTVVVVLASANRDPSRFADPGRFDISREDNQHVAFGHGIHYCLGAPLAR

LEGQIAIGTVLRRLPDVRLAVDEGDLQWRLGIVMRGLHNLPVRFTPSGPE

H

>CYP147F(2546572629)Streptomyces albulus CCRC 11814

MTSVALLRQVLDYANRPDPYPLYAELRRTPVVRDEAGPYLVSTYWAVKGL

LHDPRISSDARNLTPEAAEATGQEQDPNLPPSFLRLDPPEHDRLRRLAMR

PFGPPHTPRRVFEMHGELAGIVTGLIDGLRGRDAIDLVDDVSYPFPVTVI

CRILGVPREDETHFHEWADTIAAGLDPVGTPEERAAKTQDVQQARRDLAM

YLAGLIEERRRSPRDDMLSALATEHGPDGQMSPVEMITTSVLLLIAGHET

TVNLITNGMLTLLRHPDVLQRLRTEPALAGPLVEELLRFEPPVQMLPQRT

TLAEIEVAGTVIPKGAAVYLMVASGNRDPQRFTDPDRFVPDRPDNQHLGF

GHGLHSCFGAPLARLEAQLALTELVRRLDAPQLVEDPPPYRQNAVLRGPR

HLPLTIGGIRD

>CYP1064A(2546572644)Streptomyces albulus CCRC 11814

MTSGTGRQTGGAARALPGPEPRADGGAGAIAAAGGLHAYQLRLHDAYGPI

VRFQLPGTELAVSISDPVLLEATAGLDERPVRPFAFLEPLCEAGNLQVLP

AAEHGPWRHLLLSVLAGRPSHERHFGQFTALTTALADRWAEQADRQPVAL

QRDLTALSLRMICAYAFGGEVTDPEGVVTAFEEVLTEHLGRLYEVPGAVG

PAERAERAERAAAALARLRATVDRVVAAHRRAGRTDRSDLIGALVAAGER

PARIRDTVMMIMLAAHHTTGVAVSWTLYLLGRHPEVAARVTEEVDRVLGD

RAAPEYADLRRLTYLGMTLKEAMRRYPPGPYGARETAEDLAVGDYLIPAG

ATVFYPFWAVHMNPKYWPEPEKFVPERFTPEAVAGRPRLAHVPFGFGPRS

CEGAALAVVEAELVLAVLLKRFRFRPVAGDEVTPVERFVLWAADDIRMLV

SPRTPG

>CYP156B(2546572959)Streptomyces albulus CCRC 11814

MYEQLRKHGPTAPVELSPGVEAELVTDYATALQILQNPDSFARDPRRWRA

LNEGRVPLDSPVLPMMMYRPNSMFSDGATHLRLRQVVTDSLAKVDMHRVA

RHVDRVATYLVDQFSIRGKADLIGDFAQVVPLLVFNDLFGCPAELGDRLI

VAISSLFDGIDVERANEEMAGALFELVALKRALPDEDMTSWMMQHPAKLS

DEEMVHQLALLIGAGTEPVQNVIGSSLLLLLSEDQFADGQHGGGVLVEDA

INEVLWNSPPIANYATHYPVRDVEVSGSKLPAGAPVLISFAAANADPTLS

ATRQTFSKRAHLAWGAGPHACPAKDPALLISVRAIEKLLNTLPDIELGVP

QETLTWRPGPFHRALNALPSRFTPIRSDRRSSAGYAAQDAAQPGGGAQDQ

GRRKRDGWWSGFLNWWKV

>CYP1035A(2546572960)Streptomyces albulus CCRC 11814

MPFYTELQSMGEMVPAPWGGHLATSYRLCHQVLRSRDWRVPDSGWRAAQA

DAVRWHAPASQQMGATMPMLNPPHHTQMRRPLGNVFDRAALQQMERSVER

NAEFLVDTFFEQLSGGTADFCALVGDELPVITVGEWLGLPSADFAVLRSL

THDQVHTQELFPTPSQLAISDSATRNLRKYFTDLIRERRKAPGDDPVSRW

LRMWDEFEPDQDAADEAVYALALFMLLAALETTSHLLTTMMWLLLEHPRQ

VDWLRTHPEHIPGAIDETLRYDAPIHMISRIAPEDTELAGVPVREGEMVQ

LMVGAAHHDPEQYAAPEVFDVRRKAPHLSFGGGIHYCLGNALARMEATCV

LTSLLRRLPVQGLRVAGTPTWAPRVAFRRLMELPVVRT

>CYP130A(2546573101)Streptomyces albulus CCRC 11814

MYAALRDRDPLHHVDDGDYWVLSRYADVLAAARDTERFSSAGGLTFTYGE

RERLGITDAAPMVMLDPPEHTDFRRLITRGYTPRRVAAIEPDVRAFVRDR

LDRIAGLGAGCDIVAELFKPLPSFVVGRYLGVPEADRGRFDGWTHAIVEA

NALGDPLAAVEAVGGLFGYFTELVARRRAEPADDTVSDLVRLLPDDDTAL

LRILGFAFTMVAGGNDTTTGLLGGAAELLTADPDRRRALLDSPARLPAAV

EELLRLTSPVQCLARTVTADTTLHGRTVPAGRKVLLLYGAANRDPRAFGP

DADRLDLTRDGPQHLAFTHGPHHCLGAAAARLVARVALAELLARFPDFAV

DAAGGTFADGHYVRRYATLPFVTGRG

>CYP155A(2546573337)Streptomyces albulus CCRC 11814

MRGYDEARAVLRSTTTVQAGLGIESVEGLPSRIRRPVLYRDGPEHREHRR

QTARFFTPRHVDQHYRDVMSRATRTQLDRLTANGRATLSDLAFNVAIEVA

CAVIGLTESRPGIKQRLERFFPEEFGTPGLRSLRGIYWTIRQSTNWLRVH

LGDVRPAVRARRRRRRDDLISHLLDEGCSTSEILGECLTFAAAGMVTTRE

FISAAAWHLFSDTELLTHYQAVDEAGRIAVLNEILRIDPVIGRLSRRTTG

PLDVPRNGDDPLTVPAGERIDILLDHTNLDERTVGPRPQRIRPGRTMREG

ARSPGLSFGDGPHKCPGTHLALLETDIFLTSLFALPGLRMATPPTIGFID

GIASYELRHCVVEVDTVP

>CYP107Z(2546573355)Streptomyces albulus CCRC 11814

MTELTDSPFSEFVGKHPGEPNVMEPALLTDPFAGYGALREQGPVVRGRFV

DDTPVWFITRFEEAREVLRDQRFANSPAHSAGGGSADTPIDRLLEIMGLP

EHYRAYLSGTILNMDAPDHTRLRRLVSRAFTARKITDLRPRVADIAEDAL

RRLPEHAVDGVVDLIPHFAYPLPITVICELVGIPEADRPQWREWSTHLVS

LRPELHPETFPEMIDHIHALIRERRTALTDDLLSELIRVHDDDGSRLSDV

EMVTLVLTLVLAGHETTAHLITNGVAALLTHPDQLQLLKSEPALLPRAVH

ELMRWCGPVHLTQMRYATEDVELAGVRIKKGEAVTPVLVAANHDPRHFAD

PDRLDLTRQPAGRAENHVGFGHGMHYCLGATLARQEAEVAFGKLLAHYPD

VALAVAPEDLQRVPLPGSWRLASLPLRLN

>CYP107L(2546573988)Streptomyces albulus CCRC 11814

MNAKTTHGAREETVVDLAAYGAQFVENPYPVYAELRAKGPVHRVRVPGQE

RDFWLVVRNEEGRRILADERLSKDWRSQGVWPADALPINENMVESDPPKH

TRLRALVTRAFTARRIEALAPRVHTLTADLLDAMSKAPNGRADLVAALAF

PLSMTVICELLGVPDLDRQSFRQWTNEIVASSSPEATAEAVRAVNAYLTG

LIEQKRAEPRDDLLSALLRTTDEEGDRLSPEEVVGMAFLLLAAGHETTVG

LISNTVLALLRHPDQLALLKADFSLIGNAVEETLRYDSPTENSTYRFATE

AMDFCGARFEKGDPVLVSLAAAGRDGERFEDGERFDITRSARGHLSFGHG

IHYCLGAPLARLEAGVAVRALLERCPDLRLDTSEPLVYIPGMLVRGVRRL

PVRWTG

>CYP125A(2546574557)Streptomyces albulus CCRC 11814

MHCPALPEGFDFTDPDVYQSRVPLPEFARLRQTAPVWWNAQPHGIAGFDD

DGYWVVTRHQDVKEVSTKPEVFSANLNTSIIRFNQGISRDQIEVQKLIML

NMDPPEHTRVRQIVQRGFTPRAVRALEAALRRRAEQIVEEARHKGSGDFV

TDVACELPLQAIAELIGIPQEDRARIFDWSNKMIAYDDPELAITEEVGAN

AAMELISYAMNLAAARKECPAQDIVSRLVAAEHDGNLGSDEFGFFVLLLA

VAGNETTRNAITHGMHAFLTHPDQWELYKRERPATAAEEIVRWATPVVSF

QRTATQDTELGGARIKKGQRVGIFYSSANHDPEVFDRPEVFDITRDPNPH

LGFGGGGPHFCLGKSLAVLEINLIFNALADAVPGISLAGDPRRLRSAWLN

GVKELQVHYR

>CYP147F(2546574923)Streptomyces albulus CCRC 11814

MTLGTLPAQITDYANRADPYPLYAELRRTPVRRESDGTYLVSTYYEVRSL

ANDPRLSNDTSHRSPGYARIGQPDEETGLPPSFIFTDPPLHDRLRDTINR

PFGPPHSPRFLDDLRGDLAKVVTELLDAFEGKDEVDIVEDFSYPLPVTAI

CKVLGVPREDEPRFHGWADALASSLDPQAGEDGLEKAQRARQELGSYLTD

LIETKRRHPGPGMLSALAPEMTPADLEATAVLLLVAGHETTVNAITNTTL

TLLRHPDVLARFQREPALAVPLIEEVLRYEPPVQFVPWTTALADIDVADT

TIPEGSPVWLMLAAANRDPKRFPDPDRFDPDRKDNEHLGFYTGIHYCFGA

PLARMELHVAVPELFRRVTFSRLLEDPPPYRANAVLRGPRHLPVAIEGLT

A

>CYP147F(2546575003)Streptomyces albulus CCRC 11814

MTTTQTPDTLRRILDYSSRADPYPLYAELRETPVARQEDGSFVISTYREI

SDILHNPHLSSDTRNLSCPTEGMQAEGTPAFINLDPPEHDRLRRLAMRHF

GPPHTPGLVTGMEGALTTAVGHLIDDFAGKERIDVVDDFAYPFPVAVICH

LLGVPREDEPRFHRWVDDLINSIDYNPKTDPKEKLDKGVQARKDLRQYLG

GLLEQRHGHPGDDLLSRLANDDGPDGRMTDEEIVATANLLLIAGHETTVN

LITNGMLTLLRHPHVLQRLCKEPDLVVPLVEELLRYEPPVHIIPWRAAYS

DITVAGTTIPKGAQIMLMLASGSRDPNRFHDPDRFDPDRRDNQHLGFGSG

IHLCFGGPLARREAQIALGQLVRHLDRPRLVADPPPYRRSPVLRGPIHLY

VEQGAA

>CYP105D(2546575778)Streptomyces albulus CCRC 11814

MTGTIPFPQDRSCPYHPPTNYRPLRESGPLSHVSFYNGRKVWAVTGHAEA

RTLLVDPRLSSDRQNPAFPIPVERFEAVRRVRTPLIGVDDPEHNTQRRML

IPSFSVKRTAALRPQIQQIVDGLLDRMLEQGPPAELVSAFALPVPSMVIC

SLLGVPYADHEFFEDRSRRILRGGTAEESEQARRELEGYLADLMARKETD

PGDGLLDELIAERLRAGTLQHQELVRLAMVLLVAGHETTANMISLGTFAL

LEHPDQLAQLRSDESLMPGAVEELLRFLSIADGMLRVATADIEIAGHTIR

TGDGVVFSTSLINRDATAYPSPDELHVDRSARHHVAFGFGIHQCLGQNLA

RAELEIALRSLFRRVPDLRLAVPAAEIPFKPGETLQGMIELPLIW

>CYP157C(2546575851)Streptomyces albulus CCRC 11814

MTAFPSHQPGTTSASGPPQGCPAHARAGGTDHLARLFGPEVAHDAPGFFE

RLRREHGPVAPVLVDGDLPAWLVLGYRENLDVLRTPTRFSHDSRIWHCFR

EGRVPADSPLMPALAWQPVCLFMDGTEHERLRLAINESMARFDRRGIRRC

VTRSANQLIDAFVADGRADLVRQFAEQLPMLVLTQLLGMPDEAGPRLVEA

TRDLLKGSETAVESNAFLMAALEQLVARKRDAPGPDFTSWLMSHPTRLTD

EEVAQHLRIVALAANENTTNLTANTLRMVLTDPRFRASLTGGSMTLPDAL

EQMLWDEPPTSVLPARWATGDTELGGQSVRAGDMLLLGLAPGNGDPVIRP

DRSVPMHGNRSHLAFSSGPHECPGQDIGRAIVDTGIDVLLMRLPDIDLAV

PEGDLTWVSHWIARHLRALPVKFTPGVPASADASGDGADGPHDLRESAGG

GPLVAGGVDASTPPAGAPRSRPSWWARLTRWLSGQ

>CYP107X(2546576045)Streptomyces albulus CCRC 11814

MQNTAETGPAAPLDTTPLLDAPYAAYAALREAGPVHRITGTDGQPAWLVT

RYDDVRSALADPRLSLDRRHATPGNYRGFSLPPALDANLLNMDPPDHTRI

RRLVVKAFTPGRIEALRAPVQRIADELLDAMAARGRAELVTDYAGPLPIT

VICDLLGIPVERRRDFLAWSDALITPDPSRPQAMKEAIGAMLEFYTGLIA

AKRAEPGDDLLSDLIAVRDDTADDPAGDRLSEDELTSLAFLILFAGYENT

VQLIGNAVLALLDHPERLAALRRNPAELAPAVEEFLRYDTPASLAIRRFP

VEDLDIGGVRIPAGESVLLSIASANRDPERFPDPDRLDPTRELSGHLALG

HGIHYCLGAPLARMEAEIAIGALISRFPELRLDVARDEVRHRRTIRARGL

ISLPVAW

>CYP159A(2546576178)Streptomyces albulus CCRC 11814

MTSIPQAPDILSAQFATDPYPAYRVLREHYPLLHHEGTGSYLLSRYDDVE

RAFREPVFTSDNYIWQLEPAHGGRTLPQLSGREHAVRRALVAPAFRGREL

RETFQPLIERNARELIDTFRDDDEVDLVAQFATRLPINVIVDMLGLDRAD

HDRFHDWYTAVVGFIANLAQDPAIADAGRRAGEELAAYLHPIIQERRAAP

GDDLLSRLCTAEVEGVRMTDQDITAFVSLLLSAGGETTDKAIALVVRNLL

AHPEQLAAVRADRSLVPAAFAETLRHTPPVQMIMRQPAEDVTVSGGTVPA

GATVTCLIGAANRDADRYAHPDAFDILRSDLTPDTAFSAAARHIAFGLGR

HFCVGALLAKAEVETSVNQLLDAFPDLAFADGVAPPDAGVFTRGPRQLRL

RLRDRER

>CYP159A(2546576207)Streptomyces albulus CCRC 11814

MNREAPDILSPEFAADPYSAYRVLRDDFPLLHHEATQSYVISRHEDVERA

FKDPVFTTDNYDWQLEPVHGRTILQMSGREHAVRRALVAPAFRGNTLEQS

FLPVIERNARELIDAFRGTGSADLVADFATRFPVNVIADMLGLDRADHDR

FHRWYTAIIGFLGNLAQDPDVTEAGLRTRDEFAAYLLPIIRERRTAPGDD

LLSTLCAAEIDGTRMSDEDIKAFCSLLLTAGGETTDKAISSLVHNLLRHP

EQLAAVRADHALIPRAFAETLRHTPPVHMIMRQAAEDVTIGGGLVPKGAT

LTCLIGAAGRDERRYTDPDVFDIHRTDLSTTTAFSAAADHLAFALGRHFC

VGALLAKAEVEVGVGQLLDALPDLTLAPDAHPREQGVYTRGLAALPVRFT

PAG

>CYP157B(2546576208)Streptomyces albulus CCRC 11814

MNDPQHSTTGGCPLRAGASGTDAVPLYGAGLSGDPARLYREMRARHGTVA

PILLDGDVPGWFVLGYRELHQVTSNPELFARDSRRWHAWDQIPADWPLLP

FVGHQPSVMFAEGPEHRRRAGAISDALTAIDQFELRQICERLADGLIDAF

AGSGEADLMARYAARLPLLVIAELFGFPHTEVPELAADIAASLNEDEGAI

AAHQRVAERMQRLVKAKRSAPGADVPSRLLAHPAGLAEGEVVIDLLVVMA

AAQQPTANWIGNTLRLMLTDDRFALSLSGGRRSVGQALNEVLWHDTPTQN

FIGRWAVRDTQLGGRRIRTGDLVILGLAAANTDPQVRPDFSDAGSDGNQA

HMSFSHGEHSCPYPAPEIAEVIAKAAVEVLLDRLPDVMLAVPTEDLVWHP

SLWMRGLVSLPVEFTPAYTPIPAMGTASARRS

>CYP163C(2546576313)Streptomyces albulus CCRC 11814

MTAMTSRPEPAPDALGDVDLADPLLHARHDLGPLWRRLRTEAPVHWQPES

GTRPGFWVISRHADVVGVLNDPQTFTSERGNVLDTLLAGGDSAAGKMLAV

TDGGPHKALRSALLKPFSPRALDVVVDSVRRGTRALVEEAVARGTVDFAA

DVAAHIPLAAICDLLGVPLADRRQIIELTSSALSSADGVPTEEATWSSRN

GLLFYFSELAAARREKPLDDVISLLVTKEIDGRPLSHEEIVFNCYSIIMG

GHETTRFAMVGGVQALMERPEQWHALKTGQVSTASAVEEVLRWTTPALHS

GRTATQDVFLGGQFVEEGDIVTAWMASANGDERVFDRPDTFDLARTPNKH

LSFAHGSHFCLGAFLARAELAALLESLCDLVAVAEPAGAPGRVFSNFLSG

FSSLPVTLVPEGRQAT

>CYP1192A(2546576388)Streptomyces albulus CCRC 11814

MRLRERPAGYDRARHGVHLLRTPEVIADPSVYIDAIAELGPLFFDEVGGM

WVCSGYAEAVEILRDHRTFSSVREHDQDAFQELGLHASASLSTMVHEQML

FMDPPQHKAIRSALAEQFTGTRVRSRENDLRHIAARALEELPRAGVLDLV

ADFAAKLPSALVAQLLGMPGREAELTRWAEAYERLLGSLSALPAAPDREV

DAVLTDALSVLQNEARSRLHAPGDDVISSLTAPLVDRSPTGEELFAVAAN

CIVLVGGGYQTLTHLVTSALLALHDDPSLEKQLRELPELIPPAVAEFMRI

NGSSQYVARKATTDVKIQGTLITGGESVLVHLAAANLDPRTFSAPRALDL

TRHGPKHLGFGSGRHTCPGAGYAERLAGFAIEGFLAKYPSYAPESEPQAF

SWGLHGNTRCLEHARVRVDAEVIPAATVDIPAAETHDSTGGNGLSLPPAA

TTAAACWHEVFERQALLTPDAPAVQAPDDLISYRELDHWANALAHRLRHQ

GAQPGALVGIVMERSVEFVLTVLAVAKTGAAFLLADISCPRERLRTMLVE

AEARLVVTDGSLPSSAFPVQIVGVGAKDFRPDAPLTGVSPGDTAYVVFTS

GSTGAPKAIAISHEATVNLHLAQHQIFGLEPRDRVLQFLSPNFDGCIADL

TLALLSGAALIVAPSNQLTVGPPLVRLLASQRVTTAILTPSVWMTLPDQP

LPELRIAAAAGERLPAAWARRWAAPGRRLLNLYGPAETAVLATWHECSPS

EDPPPIGRPVANKRAYLLDHHLRNVPPGQRGELWLGGLGVGRYLNQPDLM

EERFIRNPHTTTDPASLLYRTGDICRQRPDGTLEYIGRRDRQVKIRGQRV

ELDEVERVLESAPGVTACAVHEQDGRIEALAVPAGPQLDETAIRTYLASR

LHSAMLPSVFTTVTELPRTVNGKADHRHEPPDEPTVQSSRPSGPALPPDD

HERRLSRITWEVAQNFAQVLNLPLRQVQADSDFFTAGGDSITMAAFLARL

ESLAGAPVDTAALITAPTPEQITTLLLNAGAPK

>CYP105B(2546576450)Streptomyces albulus CCRC 11814

MPHTTIPDFPLDRAPGCPFDPPPHYAALRAQAPLVRVRIWDGQTPWLVTR

HEDQRAVLADPRFSADPSRPGFPAPTAGFKAQGREEVQALSMQDDPEHAR

QRRMLIGRFTVKQVTAMTPRLVQIIDDLLDRMEAAGPPTDLVAAFALPMP

SLVISELLGVPQQDHALFQRTAGTLISRESTVQEFAAARTELADFLGDLI

RRKDDDPGDDLLSSLVVTRMRTGELTPALLVETAMTLLVAGHETTTNQLA

LGTLVLLRNPDQLAVVRDSDDPARVASAVEELLRYLSITQNGLSRVATED

VEIAGQLVRAGEGVIVPNASGNRDAAAFRDPDRFDVGRPDVRGHLAFGYG

THQCLGQNLARKELQLAYPALLRRFPGLRTTLPDEDIRFKHDMIAYGVHE

LPVTW

>CYP107DW(2546576558)Streptomyces albulus CCRC 11814

MSEVIDLAELAATADLERELARLAAQHGIIRTRQLNQQETWTVLGAGLTR

ELLSDPRLSNDVHTHAPHGALVPGLQVMLLEQDDPGHARYRRLVSAAFAS

KAVRQLEPRIVEISRQLLDKLGDSGTADFIDAFTYPMPLEVICDLLGVPG

EDRDPFRKWAMDISAAPSLEAMQTSAGELFAYCIGLIGAKREQPTEDLLS

ELIAARFEDGTGLSDEELSSFAAVLLIAGHDTVTNLLANALHDLLTHPEQ

LAALRADRSLVNQAVEEALRFRGSAMTTVNRVALEDIEAGGVTIRKGELV

RFLLNAANRDVEVREDGHAFDIGRATAQHVAFGMGPHFCLGQRLARQEAT

IALNEILDRFPKLELGVSRAEVRWLASDAIRGLEELPLRYARETA

>CYP147B(2546576832)Streptomyces albulus CCRC 11814

MSTETLLERINAYTSRPDPYPLYTELREHGVARQDDGSYLVGTYHEIAAL

LHDPRISSDVRHRTHPDLRDRPEDLPPSFIAVDDPEHDRLRRLAMRHFGP

PHSPGRIDALHDDIVDAARELVDGLRGRDRIDLVDDFAYPLPVTVICRLL

GVPREDMPEIRGWTNTIIASLDRSPDEDPDGRLRAAAQARVSMAGYLGAL

AQRRRERPADDMISALVHDDGPEGRLTEAELTTTLTLLTIAGHETTVNLI

TNGMLTLLRRPEALERLRREPELMPSAVEELLRYEPPVQMLPQRTPLTDV

EVGGVTIPQGVPLFLVLASGNRDPLRFEDADHFDPARRDNQHFGFGSGVH

NCFGAPLARLETQVALTALLHGLDAPRLVEDPPPYRHSPILRGPRHLLVT

GDVAAGAGA

>CYP163D(2546576860)Streptomyces albulus CCRC 11814

MCVTRGALAAHVDDMAERLELVPEDRVLWFAAPHVDVAWEQALTPLRVGA

TVVTRGPGVPTFGELADLVERHAVTVANLPGGYWNGWALALTEQQRTQRR

ALRLMISGSERMSARAAVNWQRILPDVPLLNAYGPTEAVITSTLFRVPAG

LAARDEIPIGTACGSRQLQVLNAELAPVVRGQVGELYVGGGPLAREYLGR

PSMTAARFVPDPYADSRGAVMYRTGDLVRENADGDLEFVGRIDDQVKVRG

FRVEPAEVRLALERHPAVRHCAVLGRTAPGGPTSLVADPAFWAGDDSLPI

LRELRQRAPLWHLESATEGPLWCVLSHELAGEVLGDAARFSSERGSLLGT

GRDRAPAGAGKMMALTDPPRATGTCGTWCCRIRARLPRRAAAPPRGARLT

RPCPAGGCPPAHPGSPLPGLRCGGELRSCLASVTIGKGVSWAKACFRRGS

VW

>CYP180A(2546577114)Streptomyces albulus CCRC 11814

MRRPGGRRRAGGRVTGSTGGAGREPARSPDVPDVFDPRRYAAGLPHDAYR

LLRDHHPVAWQDEPEVLGWPAGPGFWAVTRHRDVVRVLKNARTFSSRLGA

TQIRDPDPADLPFIRRMMLNQDPPQHNRLRRLVSRAFTPRRVDRFEDAVR

DRARTLLASAVDAARAADGVCDLVGTVTDDYALRNLADLLGVPPAERGLL

LDWTRRVIGYQDPDEAGPAVTGASGRPVDPRSPAMLRDMFAFARELAAHK

RRHPGDDLMTVLAADRELAVPELEMFFFLLTVAGNDTVRSAAPGGLLALA

GHPDAYAALRTGAVPMGPAVEEMLRWHPPVLSFRRTAAVDTELAGRRIRA

GEKVVVFHASANHDERVFAEPGRLDLGRAPNPHVSFGDGPHVCLGAHLAR

LQLRVLYEETCALLPAVAVAGPPPRLVSNFINGLKSLPLRVAT

>CYP180A(2546577173)Streptomyces albulus CCRC 11814

MPQRSTASRGGRQVRFAPRIDALLRQHTGQDLFRLEPTTVGVGGADLMDA

LLRSRPANAEERPTFKPVLGRHVSRADAATYMQAVAADVRKALQRPLEAP

VDLTGPWPQVPHAYLRDLVFGRELLRFRVLVDRRLELTPKLTWSAVTSGA

ALLRRPDSTEPLSKLAALVLGATGFPDRRYAMYLYRRVAAPICFTVAALV

TNAVWLGAPFDDSVPNRHLLAEALRLLPPSWNILRMASPEFTVLDTRIGP

ADDVLLLPLLSHRDPALWEEPDAFRPERWADLDPDDHPGYLPFGHANERC

WGRHMVLPLAERLLDLVRRDGLTVRPAQTVGRVELDGLMEVAEVRVMRG

>CYP107EA(2546577239)Streptomyces albulus CCRC 11814

MPSAIIAVGPFPAPGQDHADRSHADGSADPSTTAPQTPEPVTMNRPATEL

PPRSAAPGRPDPASAADVHRPFDAGFFRDPYPVYARLRTLGPVLKVVLPD

GSHAWLVTREEHVRAAFTDPRLSVNKARSRNGYQGFSLPPALDANLLNID

PDDHLRLRRLVSRGFTPRHVERLRDRVGTAAAHYADRLAERLAEHGTADL

LAEFANPLPLVVIGHLLDVPEADGRAFSRWVAAMHAPARPGDTAEAIEHI

HRYLLELIRARRAAPGDDLLSSLIAARDADDRLSEDELVSLAFLLLMAGT

ENVQHLISGGVLTLLRHPEHLAALRSRPELMPDAVEELLRHAHPNQMAIR

RFPTTSVEIAGVRIPAGDTVLLGLASAHRDPDRYPEPELFDIHRADKSHL

ALGHGLHYCLGASLARMEIGVALGTLLDRFPGLRLAVSDGELEWRQSFRS

HALRRLPVAVSAPAG

>CYP161A(2546577346)Streptomyces albulus CCRC 11814

MSTPTAPPSLAAEARTVLRLSPLLRDLQSRAPVCKVRTPAGDEGWLVTRH

SELKQLLHDERLARAHTDPANAPRYVRNPLLDLLVTDDVDGARAVHAEMR

ALLTPQFSARRVLDLAPKVEALAEQALAHLTAQGPPADLHDHFSMPFSLS

VLCTLIGVPAAEQGQLIAALAKLGEIDDPPRVQEAQDELFGLLSGLARRK

RTEPEDDVISRLSRKVPSDDRIGPIVAGLLFAGLDSVASHIDLGTVLFTQ

YPDQLAAALADEQLMRSGVEEILRSAKAGGSVLPRYATTDVPVGDVTIRT

GDLVLLDFTLVNFDRTVFDEPELFDIRRAPNPHLTFGHGMWHCIGAPLAR

VQLRTAYTLLFTRLPGLRLARPIEELGYSSGQLSAGLRQLSVTW

>CYP105H(2546577348)Streptomyces albulus CCRC 11814

MAFPLRQPGRPFPPPEYAQYRAGPGPVRTELPSGTVWLVTRHEDVRAVLT

DPRISADPSRPGFPKASRTGGAPSQYEVPGWFVAMDPPEHGRFRKTLIPE

FTVRKVRELRPAIQQIVDERIDAMLAAGTSADLVESFALPVPSLVISSLL

GVPRADRDFFEDRTRVLVRLSSTDEERDKATQALLRYLGRLIQIKQRRPG

DDLISRLIAAGTLSRQELSGVSMLLLIAGHETTANNIGLGVVQLLANPQW

IGDDRIVEELLRYYSVADLVAFRVAVEDVEVGGRLIRAGEGIVPLLAAAN

HDDTVFAAPREFDPERSARSHVAFGYGVHQCLGQNLVRVEMEIAYRTLFA

RMPSLALAVPVEELSLKYDGVLFGLHELPVTWK

>CYP107U(2546577504)Streptomyces albulus CCRC 11814

MQQTSDARQENRSDSHPEARPEARPAGDPPTGCPAAASATAAGPSPALFS

WEFAADPYPAYAWLREHAPVHRTRLPSGVEAWLVTRYPDARQALADARLS

KNPVHHSEAAHGKGKTGIPGERGANLMTHLLNIDPPDHTRLRRLVSKAFT

PRRVAAFAPRIQELTDRLIDAMIEKPHGGQRGSADLIHEFAFPLPIYAIC

DLLGVPPEDQDDFRDWAGMMIRHGGGPRGGVARSVKKMRAYLAELIHRKR

ADLGDDLISGLIRASDHGEHLTENEAAAMAFILLFAGFETTVNLIGNGAY

TLLRHPAQRELLQKSLAAGDTELLGTAVEELLRYDGPVELATWRYATRAL

TLGGQRIAEGDPVLVVLAAADRDPARFDEPDVLDLTRRDNPHLGYGHGIH

YCLGAPLARLEGQTALATLLTRLPDFRLAVEPDDLRWRGGLIMRGLRTLP

VEFTPEPSRPEAIP

>CYP1190A(2546577771)Streptomyces albulus CCRC 11814

MTTTDAHPPTSRTAAEGPPAFPFDDWGQRISPAYARLREAPAPACRVVTV

TGDQVWLVTRYGLARRLLADPRLSLTAALEADAPRQEPLRPRATGARGDG

MATLQERGLRGILADALSPRAIRAHHAWTRLRARALFDELSEQGPPADLQ

QGLARPLTFAVARRVLLGELTEDEGQVLNAWCDTVLVWRDRTRDEIQAAL

DAMYGFFLRRAPELAAAPGSDVVKRAAAACTRDGGRLGADGLAEVANLML

IAGYRTAASFVANALVMMLSHPTALAALRDRPALLPSMVEEVLRHTPMST

GGVKRVATDDVPLDGLTIKAGECVLVSLESGNHDPHAYPEPDRFAPDRFA

ADRGPVDGTSSEAGRPRSRPHLGFGHGKHHCPGNALARMQIAVVLQTLAD

HTPALRLAVPAGELRWRPDVAFRIPETIPVTW

>CYP154D(2546577805)Streptomyces albulus CCRC 11814

MEDIAPVGPPHRMDPSGGCPHADNARLLARGAVAPVVLPGEIEGMAVLGH

AALKEFLGHPDVAKDARHFTALSEGRIPEGWPLRTFATVRSMTTADGEDH

RRLRSLVSRSFTARRVAELQPRVEELTDSLLDDLAGAARAGGGVADLRRH

FALPLPMGVISELLGVDLAHRDRLHELSVEVVTTDIGSQRAIAANHEFAA

VIGEFVAAKARHPGDDLTSALIAARDDDGDQLSGPELIGTLLLMIVAGHD

TTLNLITNAVRALCGHRDQLELALSERVTWGDVVEETLRWDAPVSYFPFR

YPVRDLTLHGTVIPKGTPVLAGYSAAGRDPAAHGPDADRFDVTRPGRPDA

VRHLSLGHGAHYCLGAPLARLEAETALQRLFRRFPDLELAVPEDALPRHA

GFVANSVGSLPVRLWPS

>CYP107E(2546577913)Streptomyces albulus CCRC 11814

MTTAKTAPLSYPFNIAESLDLSAEYEKARNRPGLLKVQMTYGEPAWLVTR

YAEARFVLGDQRFSRAEGIRHDEPRQSEGSRNSGILSMDPPDHTRLRTLV

AKAFTVRQVEKLRPQVKELTRELLDELEAAGPPADLVDRYALPIPVAVIC

RLLGVPTEDRPKFRTWSDAALSTSSLTAEEFDANREELRAYMGNLIEQHR

REPQDDLMTALIDARDVNDRLTELELVDLCVGILVAGHETTATQIPNFVL

ALLDHPDQLAVLREQPDLIGGAVEELLRFVPLGSGAGQPRYATEDIDVGG

TLVRAGEPVLVAMGAANRDALRFDGPGKLDIRRTGNQHLGFGHGVHHCLG

APLARLELQEALSALITRFPGLHVAGDVEWKTEMLVRGPRVLPVGW

>CYP105AC(2546577922)Streptomyces albulus CCRC 11814

MRYPDGHLGWLATGHSVVRAVAADPRFSSRYELAHLPFPGMADVTLPPAP

VGDLTGIDPPHHTRYRRLLMGKFTVRRMRELTSRVEQITAEHLDAMERQG

PPVDLVAAFAHPVPALMICELLGVPYADRDRFQHHAMAASGTGGSLEDQY

AAMAALQEFVREQVQVKRARPTDDLLSDLTTSDLSDEELAGIGSFLLGAG

LDTTSNMIGLGTFALLSHPEQADALRADPGLADQAVEELMRYLTITHTGI

RVALEDVALDGQLIRAGESVTLAVQAANRDPARFPDPDRLDLRRRATGHL

AFGHGIHQCLGQQLARVEMRVAFPALLTRFPSLRLAIPPAEVPLRTDLTM

HGVQRLPVAWDA

>CYP161A(2546577958)Streptomyces albulus CCRC 11814

MSSPHRDLPSLDLETPALLRVSPLLRDLQERGPVCRVRTPAGDEGWLVTR

HSVLKQLLNDERIGHSHPDPANAAQYVRNPFLDLMIADTDAETARRTHTE

SRRLLAPMFSARRVREMEPRVAAVVDAVLDDFTAQEPPGDLHGGVSVPVA

RTVLCDIIGVPPQNREHLTALLSQTAVLGDREGVQRTQRDLYAFVGGLVE

HKRGEPGQDIITRLTEGGLSDERVTHLAVGLLFAGLDSVVTIMDHGVVLL

ATHPEQRAAALADPDVMTHAVEEVLRAAKAGGSILPRYATEDLTVGGETI

RAGDLVLFDFSLPNFDERAFDEPERFDVTRSPNQHLTFAHGMWHCIGAPL

ARIELNTVFTQLFTRLPDLRLALAAGELAENEGRLSGGLSELPVTW

>CYP105H(2546577963)Streptomyces albulus CCRC 11814

MTHSDPVVVDFPTRKPGVPFPPPDYDGYRDHEGLVLSRLPNGARAWLVTR

HEDVRAVLTDSRISSNPSHKGFPNVGTVGVPTQEQIPGWFVGLDSPEHDR

FRKALIPEFTVRRIRGLRPAIERTVEERLDAMLAAGNSADLVADYALPVP

SLVISTLLGVPPSDRDFFESRTRTLVSLRASTDAQRETAVKELLRYIKRL

VGIKAKWPGDDLISRLLAAGSIAPHELSGVLMLLLIAGHETTANNIALGV

VTLLRNPQWIGDDRAVEETLRFHSVADIVSLRVAVEDVEIGGQLIKAGDG

IVPLIAAANHDTSAFECPHMFDPSRSARHHVAFGYGIHQCLGQNLVRVEM

EIAYRKLFERIPDIRLAVPDEGLSIKYDGVLYGLEQLPVRW

>CYP107AE(2546578180)Streptomyces albulus CCRC 11814

MNDPMESAEFSRDPYPLLAALRARGPVQRVRTGKGRTTWVVTGWAEARAA

LADRRLSKDTARYFANRPSGRDLAPAVSQSMLATDPPDHGRLRKLAMAAF

TPAAVGRLEPRIREIAEGLAAELGRSAGAAGGPVDLVEGFAVPLPIAVIC

ELLAVPEADRAAVRRWSDDLFAAADPGTADRASHALAGYVAELIAARRAA

PGDDVLSGLIAARDAGDRLSERELVSLAVLLVVAGHETTTHLIGNGTLAL

LRDDALHARLRDDPGLLPAAVEEFLRYEAPVTLATFRYATEAFDLGGVRI

GAGDVVLVSPGGANRDPARFDEPDAVRLARSGAGGHLSFGHGPHHCLGAP

LARAEARIAFEVLLARFPGLRLAAGGGQGAGGSDGPDGVTWRRTRLMRGP

AQLPVLLGPLREAPEGRG

>CYP107B(2546578276)Streptomyces albulus CCRC 11814

MSNSSLPHADAFTAESLRDPHALYAKMRDEAPVQKVVLPQGLAVWLLTRY

DDVRAALSDPRLRSDKSDVDGVLRNHLVSQEARESWVDELSGNLLNTDPP

DHTRLRRLVNRAFTPRTVAAMRSRIEEVTDELLDALPRGSEVDLLASFAL

PLPIIVICDLLGVPPEDRGVFTDWSNALLSSADAAETAEAGQKMFAFLGA

LLAEKRARPADDLLSGLVQVRDEEDRLSEEELISMALLLLVAGNESTVNL

IGNSVLALLRHPDQLAALRADPALLPGAIEEFLRYDGPINTATFRSTAEP

VTFSGVTIPAGELVVVSLLAANRDAGRFADPDRLDVTRPAGGHLGFGHGV

HFCLGAPLARMEGEIALGRLLARFPDIKPALALDELTYRFSTIIHGLEKL

PVIV

>CYP105AK(2546578304)Streptomyces albulus CCRC 11814

MSAMTTLRSRIFAWAGRLYLARTRKKGFDLSRMSFLPDSVLMPLRRDGLD

PVPDLADVREREPISKLPVPIASNVWLVTGYDEVKAVLGKADAFSSDFTN

LIGKAGAGAEQNPGGLGFADPPVHTRLRRLLTPEFTMRRLGRLTPRIHDI

VEERLDAMERAGRNGDPVDLVAHFALPIPSLVICELLGVPYEDRADFERL

SAARFDLFSGANASFGAISESLSYFRDVVKKQRENPGDGLLGMIVREHGD

SVSDEELAGLADGVLTGGFETTASMLALGALVLLQDPQHFAALKDGDDVV

DRYVEELLRYLTVVQVAFPRFAREELEIGGVQIAAGDVVLCSLSGADRDG

ELGPEMEQFDPHRAKVPSHLAFGYGIHRCVGAELARMELRAAYPALVRRF

PTMRLATRPEDLAFRKLSIVYGLDSLPVRLDA

>CYP105B(2546578538)Streptomyces albulus CCRC 11814

MTTAERTAPPDALTVPASRAPGCPFDPAPDVTEAARTEPVTRATLWDGSS

CWLVTRHQDVRAVLGDPRFSADAHRTGFPFLTAGGREIIGTNPTFLRMDD

PEHARLRRMLTADFIVKKVEAMRPEVQRLADGLVDRMTTGRTSADLVTDF

ALPLPSLVICLLLGVPYEDHAFFQERSRVLLTLRSTPEEVRAAQDELLEY

LARLARTKRERPDDAIISRLVARGELDDTQIATMGRLLLVAGHETTANMT

ALSTLVLLRNPDQLARLRAEPALVKGAVEELLRYLTIVHNGVPRIATEDV

VIGGRTIAAGEGVLCMISSANRDAEVFPGGDDLDVARDARRHVAFGFGVH

QCLGQPLARVELQIAIETLLRRLPDLRLAVPHEEIPFRGDMAIYGVHSLP

IAW

>CYP105B(2546578824)Streptomyces albulus CCRC 11814

MTEMIDTTVPAAPAPLPVEPPSGCPFDPPAEFGVLRTEEPISKISLPDGS

WAWLATRYADIRAILGDTRFSSDTTLHGYPLSGMTGGGNQQNRGFIRMDP

PEHTRLRRMVTREFMVKRVEALRPEIQRLTDELCDAMERRAGKPVDLIEA

LALPVPSLVISLLLGVPYDDHDVFQRLTGKLLSRTIAEPEREAARGELRE

YLDALVTAKEKEPGDDILGRLIVEQQRTGEITHDDVAAFAALLLIAGHET

TANMIGLSALTLMQDPDSAERLRQDPTLIRGAVEELLRFHSIIRNGPRRV

ATTDIEIDGQLIRAGEGVVVAVPSANRDATVFADPDRLDVGRANAQHHVA

FGYGIHQCLGQALARVELQVVIATLLRRFPAMRPAVPVEEIPFRTDMAIY

GCHALPVTW

>CYP102G(2546578842)Streptomyces albulus CCRC 11814

MTDTIAPQTGDRASAVGVPVADLTATGISSTPLQQAMDLARIHGPAYVRK

FGARETLFLSSVDLVTEVSDETRFAKGVSVVLENVREFAGDGLFTAYNDE

PNWAKAHELLMPAFALGSMRTYHPAMLKVARRVTASWDRRMAEGRPVAVA

EDMTRMTLDTIGLAGFGFDFESFSRDTPHPFVEAMVRCLEWSMTKFARQP

DADHTAADAAFRADADYLASVVDEVIAARAASGERRDDDLLGLMLAAGEG

EAAHQGPALDLANIRNQVITFLIAGHETTSGALSFALYHLLKDPVALRLV

QREADELWGDEADPDPTFEDIGKLAFTRQVLNETLRLWPTAAAFTRQART

DTVLGGRYPVAAGSLVTVLTPMLHRDPVWGDNPEAFDPFRFTPEAEAARS

PHAYKPFGTGERACIGRQFALHEATMLLASLVHRYRLVDHADYRLRVKET

LTLKPDGFTLALAPRTPADRAAVRSALAVLPGGPAGAAAGDATDAAADEG

LPTRVRQGTGLLLLHGTNYGTCREFAERLADEATALGFATEVAPLNAHAG

SLPTDRPVVLVAASYNGQPTDDAAAFTAWLGTAPEGAAAGVHYAVLGVGD

RNWAATYQQVPTFLDDRLAALGGERMLPRAEADASGELAGAVRKFGAALR

TELLIRHGDPASIGDRGADGADTGYAVTALTGGPLDALTARHDLVPMTVT

EAYDLTADGWSRPKRFLRLALPDGVTYRTADHLAVLPVNTPQAVARTAAA

LGVDPDSVLALRPPTGRPVRDTLPIDRPLTVRQLLTHHLELGMRPTSEQR

ALLAAHNPCPPERHALENLPDDDPRSLVELIEAHPALRGALPWPVVLELL

PPLRTRHYSLSSSPAADPRHADLMVSLLPGGTGSTYLHAVRPGDTVLARV

QPCREAFRLDPDDDTPVILVAAGTGLAPFRGAVADRVAAGRTTPARLYFG

CDDPDGDYLHAAEFAAAERAGAVAVRPVFSARPENGHRFVQHRIAAEATE

VWELLRAGARVYVCGDGSRMAPGVRDAFRAVHRERTGASEQESQAWLREL

TAAGRYVEDVYAAG

>CYP1060A(2546578934)Streptomyces albulus CCRC 11814

MRGLRPQAVAARIGEIAAGTDRIADQWPTGRDVEILPLVRPVLAEIGVRY

LFSEDAPVLLPFAWQLFVAREVLVRPSRWVWPRWVPTPARRFRTRRQVAF

TNALRPIIRRRRTSQRLGDDVLGQMLQPSSRYGPLAEEAVLDTLPGITVA

TFETPSRAAGWILLHLARYPQAADRVAAEAALLPASPASTTSTHFDNLHY

TQALVREVLRLHPPSWLLTRRAPRRTQLADYTIDAGSTVLVCPYTAHRDA

REHPEPDRFRPERWLDDAGSPTKPGVFLAFGTGPHGCEGAALAMAMLTLM

TAQTARRYHLSEPPGAEPGYRITTFEGLATAGLCLRATLRG

>CYP147F(2546578937)Streptomyces albulus CCRC 11814

MISGDNLRAFKRDGRIVVVGASLAGLRAAEALRGGGFTGSLTMIGDELGE

PYDRPPLSKQVLTGWVPADNTTLPRRRDVDAEWLLGVPASGLDLTTNHVL

LADGREVPFDRVLISTGVRARPWSVESEAALDGVFVVRTREHAEGLRRAL

AARPSRVLVIGAGFTGSEIASVCRERDISVTVAELAPAPLVGALGAMIGE

VAADMQRAHGVDLRCGVEVTQLEGDARGRFRRAHFDDGSAVDADVAVVAL

GSIRNTEWLRDSGLATDGWGITCDTSCRALDVNGRVTDDVFAAGDVARCP

NPLYEHRLISLEHWANAVEQAEIAAHNMVSAQADLRPHLSVPVFWSIQFG

VNIKSVGVPTFADEVVVTQGSLEDRRFVTAYGYRGRVTAAVSFNNGKRLH

HYRRLIELAAPFPPPCPTPDQPADRKPVPVDLPGPALLAQGATAVVTGHD

LGERRVTAAPQHRQEQGRTTTTGTPGTLQRIFDYSARADPYPLYAELRTT

PVIRQEDGSYVVSTYREITDVLNDPHLSSDLRNLSRPMPPADEGATSSFI

RMDSPEHDRLRRMAMRHFGPPHAPGLVTGLEGFLTATVGSLIDNLAGKEQ

IDVVDDFASPFSVTVTCDLLGVPREDEPRFHVWVNDLMNSIDYNPETDPK

EKLDKGVRARKDVRQCLGELVERSHSRPGDGLLSQLANDDGPDGRMTDAE

IVATARLLLIAGHETIVNLITNGMLTLLRHPPVFQRLCGEPDLIVPLVEE

LLRYEPPVHIIPWRVAYSDITVADTLIPKGSQIMLMLASGNRDPNRFHEP

DRFDPDRRDNQHLGFGSGIHLCFGGPLARRETQIALTELVHRLDHPRLVA

DPPPYRRSPVLRGPLHLDIEQGCG

>CYP251G(2546579342)Streptomyces albulus CCRC 11814

MTGAGVPGDDPRGGSPRGAAGVPGRVPLVGHAVPLRRDPLRFLCGLRQRG

PVTKIYIGPRPVHVVNSSDQVRELLTVQARSFDKGAMFDALRVPLGDGLI

TAAGDRHLRHRRLVQPAFHHERIARYARTMAERSLARSADWAPGTTRDLV

PDINRLTLDVLLRTLFAAPHEPGLDAAVQDWLTVKYHSMRLALSPLHAWA

ERVPLLPGWRPPDAGPLRRLVDVQLRIIDGYRADGRDRGDLLSMLLLAGG

PEGALTDAEVTDELITLFLAGTGTVSASLAWALHEISRRPDVQRRIHDEL

DTVLAGRPPACEDLPALVYTRQVLTEVLRLHPPSWLLMRRAVRPVTLGGV

RLAPGDEVFFSPYALHRDPHLYEDPEDFAPDRWPADAAAKAPRHTFLPFG

AGSRLCIGEDYAWTELTLAVAAFTAGRRLEPAGTAPVRALVGTVLRPDRL

PLTARPRPA

>CYP1191A(2546579504)Streptomyces albulus CCRC 11814

MLACPGLHHEQAEHTYFAARHSDVHHGLRHPDLAVGFPFRATRQLFGPTA

IDLDAPRHRPARQQVSWFTTRHMPTWNRTAVIPVIDDLIERAATDTPLDV

IKTFAEPLPTRVICRILGLPDHEWPWVWKQLRPVIGHIADPRTGMQAALA

SRDILADRLRHAVRTGVPHGSLLQRLCANAPSDSAVDRTAEPIRTALLLL

AAGTETTAAAVGNLLWCLQRRPHTWDEVAAGTIPAEAVVTESLRLHPPLH

STVRFARRDLTLGDTAIPKGARVQLLLAAANRDPARIGASPSWDPHRPPQ

AHHAFGGGPHACVGAQLALTEMKFLLTALTQRFELAGPTHPAGRFRAGPF

HYPTELPVRLIPRTTPA

>CYP1240B(2546579532)Streptomyces albulus CCRC 11814

MTSPYEPGAAPPPSACPGQAVPPSEAAAPPPGCPAHAGPQRAAAHPEAHA

PVKLYGPDFAADPHRIYARLRQYGAVAPVEIAPEVPALLVTDYRAALELL

NDDATWSKDSRAWMQTVPADSPVMPMLHWRPNVFYSDGPAHVRYRDAIVD

SFKLVEPHELRARVHHAADTLIRRFGDRGEADLIADYARLIPLLMFNTLF

GLPDSYSDRLIAAIAGMMEGNSPEEATAANEAYTQYIMELVGAKKAQRGP

DLTSWMMDHANDLSDEELIHNIILVMGAGNEPLANLIGNALARMLSDDRY

YNTVSGGALTVHDAINEVLWNDPPMANYSAHFPVRDVFFHGTWVRAGQLV

MVSYAAANSQFDSTGAHGPESGSGSHLAWAAGPHACPVKRHALLIAITAI

ERLTAWLSDIELAVAPAELTWRNGAFHRALAALPARFTPITPDQAGATPW

QNSDRSPSSSTRPAPTSTAKETASAH

>CYP154A(2546579533)Streptomyces albulus CCRC 11814

MAEQRPQPVVIDPAGTDIHGEGDRLRALGPAARIELPGGIQAWGVTGHAL

LKELLTDDRISKNPRDHWPEWQRPEIRGSWLQSWIGVTNMFTAYGADHRR

LRKLIAPAFTARRTDAMVPRVTQIVGDLLDGLAARPAGEVVDLRESFNHP

LPMQVICELFGYPEGAPRGELARVVSEIMDTTATPEQATATQAAVAELLG

SLVATKRAQPADDLTSLLVAARDDEGQGMTERELLDTLLLVIGAGHETTV

DLLGNAVFALLTHPEQLKLVRDGAVSWHDVIEETLRWTPSIASLPLRFAV

QDVALPHGEVIRKGEALLPMYAAAGRDPEQHGPAAATFDVQRASQDHLAF

GHGVHHCIGAPLARLEARTALPALFERFPDMQLAVPAEELQPAGGFIAGG

LASLPVRLTA

>CYP107EA(2546579539)Streptomyces albulus CCRC 11814

MIDQRGLLKPFRPEFFANPYAAYARLREDSPVCRVELPDGTPAWLVLREA

DVRSALADTRLSVDRSCSRNSGYKGFSLPPALDANLLNLDGDTHIRLRRL

VMRAFTYRRIGDMRNDVIKAAERLSDKLDSSSTCDLVTDFATPLPLQVIG

DMFDVPEEHRRPFAAWVGTMFALERPQQVRDSIDNIHQFLLRLVAERRRE

PGQDLLSALIAARDDHDRLTEDELVSLAFLLLSAGVQNVQHLISNGIHTL

LQHPEQLAELRSEPSLLTSAVEELMRFAHPNQMSIRRFPTEPVQLGGVTI

PAGDTVMLCVASANRDPARYPDPDTFDIRREDKSHLALGHGVHFCLGASL

ARMETEATIGTLLRRFPNLSYAAPVEELQWRSTFRSRSLKALPLRLK

>CYP1189A(2546579760)Streptomyces albulus CCRC 11814

MTTTEQIAAFADVPGPQGSGVAGVAPEFLRDPIAVLARAHRDHGDLVAFP

FGPRKGPLGKVVVAAYHPDAARQVLTETERTIGRGPSSTQVLDDMIGRNL

MTTDGAEWRRQRRTLQPLFTPKRVAQYTDLMAAEAARIVAEDVPAGTTAD

RVDLHRLMLRYSLRVVGRALFSGDIDYTAPELHKLIPLTNELIIGRTTQL

LKPPLALPTPRNRAFLRTKAQLYALIDRILARSDAEAGGAERDDIVTRLR

TARDPETGAGLSDAEIRDQTLLMMMAGHETTATALTFALHLLGRHAEVQQ

AVADEALEYTRGGGTPAEFAQQRESLARASLLESMRLYPPVYMTEHLATA

DIVLGGYRVPAGTAVFLSPWVTHRHPEFWPDPERFDPHRFVGEHDRPRYA

YLPFGGGPHVCIGEHFALLAATVLLEAVVRKFRIESLEESISYQQTGNLR

PDKPVWAALTAR

>CYP1189A(2546579761)Streptomyces albulus CCRC 11814

MSTTAQLRPFHDIPGPKGHALAGLLPDFNADPLGFLTRGFQEHGDLVAYR

FGPRKGPLGKTILAVYHPDLVHQLLMDTERTFGRDTDGFRATYELVGRGL

MTTEGPYWRRRRHILQPLFTPKRVARYTELMAAEAERIIAEHEQFEGTEI

DLHQAMMRYSLRVVGRALFGGDLDDAEAELHALIPDANRGIMARTTQIPK

LPLKFPSPTNRMFVRTRDSLYDLIQRVIERSGSGDAASSEDNIVSRLREA

RDPESDEPLTEQEVRNEALLLFMAGHETTAMGLTFGLHQIGRHGDVQKAI

AAEIDAHHAAGGTGAEYAQNRDTLGRAALNEGLRLFPSVHMTERVANEDL

ELNGYHVPKGTSVFLVPWVTHRHPEFWPDPERFDPDRFVGKQADRPRYAY

FPFGGGPRVCIGEHFALLASSILMEALLRKYQITSHDEQISMKVLNSIRP

DRDVRTTFTRR

>CYP105A(2546579869)Streptomyces albulus CCRC 11814

MTDTATTPQTTDAPAFPSNRSCPYQLPDGYAQLRDTPGPLHRVTLYDGRQ

AWVVTKHEVARKLLGDPRLSSNRADTNFPATSPRFEAIRERPQAFIGLDP

PEHGTRRRMTISEFTVKRIKNMRPEVEEIVHGFLDEMLAAGPTADLVSQF

ALPVPSMVICRLLGVPYADHEFFQDASSRLVQSTDAQSALTARNDLAGYL

DGLITQFQTESGAGLVGALVADQLANGEIDREELISTAMLLLIAGHETTA

SMTSLSVITLLDHPEQFAALRADRSLVPGAVEELLRYLAIADTAGGRVAT

ADIEVEGQTIRAGEGVIVVNSIANRDGTVYEDPDALDIHRSARHHLAFGF

GVHQCLGQNLARLELEVILNALMDRVPTLRLAVPVEQLVLRPGTTIQGVN

ELPVTW

>CYP105B(2546579873)Streptomyces albulus CCRC 11814

MPDHAVTVPTRTPHTPLERRSSVPDATGPTPTPTGSAEPHHPSDVPEFPM

PRAAGCPFDPPPTLTAQQQQGPLTKVRLWDGSTPWLVTRYADQRALLADP

RVSADVTRPGYPSAAPVSGNTIGFILMDDPEHARQRRMVTAPFAVKRVEA

LRPRVQQIVDERIEALLGGPRPVDLVEAFALPVPSLVICELLGVPYADHD

FFQENSRILINRNVTPEERTTAHGRLSDYLDDLVGEKLARPTDDLLSELA

QRVADGELTRLDAARMGVLLLIAGHETTANMIALGTLALLEHPGQLAALR

ASDDPKHVANAVEELLRYLHITHSGRRRVATADIELAGRTIRAGDGLIFP

NDIANRDPDAFPDPDRLDLQRAARHHVAFGFGVHQCLGQTLARLELQVVY

GTLYRRIPTLRLAVPLADVPFKHDGSVYGVYELPVTW

>CYP107AM(2546579875)Streptomyces albulus CCRC 11814

MVSHDDGTPAPAYPLTAPGALEAPAEWRELRTTCPVAPVTLPSGDRAALL

TRYDDVKQVLSDPRCTRQLDAEGAARISADPSGGVFNSAMAASLNGAGQQ

RWRRMLTKWFTAKRMNALRPAIEAMAEQLVDEMVDRGHPADLKASVGFPL

PVWVICDLLGVPAADRDRFSRWSDMLLNLTRYGRDEIDTAQRDFHAYLTE

HLEAKRAEPGEDLLSSLITATDADGGRLTDDQLAATGQALLIAGHETTAN

MIGKMMALLLADRRRWQQLVADPALVRTAVEEVLRYDANAGFGMPRYVTQ

DIDVAGTVLPRGATLVCSMAAANRDGAVFAAADDLRLERSPNPHLAFGAG

PHSCLGQALARTELQVVLDVLLRRLPSLELAVPVSELRRIEGLVVGGLCD

VPVRW

>CYP140C(2546579899)Streptomyces albulus CCRC 11814

MSDLPYAQRLFARDVRWALGHALPRLATDRAARQGDLHGQLVALSRSPRP

HGDTADVERRLMDRIRAEGPVHRSRFGFVTASHPAVREVLSSNDFRTGAL

PVTTGPLGRLAAWAGADAPVGPLKPPSLLVTEPPDHTRYRKLVTRVFSVR

AVEQLRTRAEEIAEELLDDLQRRPPGADDVDLVSAYCGLLPVTVIAEILG

VPHAERHRVLRFGTGAAPSLDFGLPRRRFLAVERSLRDFDAWLAQHIERI

RRQPGANLLSQLVTARDDDGRGLTGTELRATAGLVLAAGFETTVNLLGNG

IALLDRHPDQRAALHDDPTLWPNAVDEMLRFDPPVFLTGRAATRDTSIGG

RPVPRGALVTLLLAGANRDAALFTAPHRFDVTRPNAKEHLSFSGGRHYCL

GAALARMEAEVGLQALHRRFPHLTLHAGARRRTTRILRGYVHLPARLGTP

VPV

>CYP107F(2546580023)Streptomyces albulus CCRC 11814

MAKEADPTVWNCPFDYAEALEFDPTLRRIMTEEPVARIRLPYGEGEAWLV

TKYDDVRTVTTDRRFSRHAIVGRDFPRMTPEPIVQDEAINVMDPPASSRL

RSLVAKAFAPKQVERMRSRTQHVVDELLDRMVENGAPGDLMENLASPLPL

TTICEVLDIPEGERAQLRGYARTMMNTSLANKDNAIRAKADMREYFTELT

ARRRRDPGDDLISALATARVGDEVLDAKELTVMAMVLLITGQDTTTYQIG

NLSYTLLTRPKDLAMLRERPEALPQAMEEMLRFIPFRKGVGIPRVALEDV

ELSGVTIRAGDIVHVSYLTANRDSEKFERPDELDLSREATGHMTFGWGAH

HCLGAPLALTELQVALSTLLQRFPDLKLAKPAEELRWNTTAIWRYPLALP

VVW

>CYP163B(2546580311)Streptomyces albulus CCRC 11814

MTPSLPDDISSVDLTDPKTFEYYDLRDYWQQLRNTRPLYWHPPTASGPGF

WVVSRHADVMALYRDNKRLTSERGNVLVTLLAGGDSAGGKMLAVTDGERH

RDLRNVMLKAFSPQALRPIVDQVRVNTTRLVVEAVRRGECDFAADVAERI

PMNTISDLLGVPAEDRDALLSLTKSALSSDEEDHSANDAWLARNEILVYF

SDLVAERRAEPTDDIISVLANSTVNGEPLSEELIVLNCYSLIIGGDETSR

LSMIESVRALAQHPEQWQLLRDEKVLLESATEEILRWATPAMHFGRRSVT

DFELHGQVIAAGDIVTLWNSSANRDERVFGDPYVFDLNRSPNKHITFGYG

PHFCLGAYLGRAEIRAILDALRTFSTAFEINGRPQAIHSNFLSGLCSLPV

RFYPDDAALDAYLDRNRVTG

>CYP184A(2546580343)Streptomyces albulus CCRC 11814

MSLTNSPTPADPEAERASRQPLPTAIPGPAGLPVIGSLLDLRRDSLGAFL

KAQREHGDVVRLEAGPPGLRSVFYAVFAPEGVQQVLGTQAANFRKDHPLY

EEVRQSFGNGLLTSQDDDYLRQRRLVQPLFTKRRVDGYATAVTTEADAVT

ARWRSVEGDVVDLVAEMNRLALRTVARILFGLDVEAAVETIHRCAPVIND

YVVRRAYTPLKVPRDWPTPRNLTHRKVTTELNALCDRIIAERRTASATGT

TVTPDHNDLLSLLVAAGNEEDGTLDATEVREQVLIFLLAGHETTATSMAF

ALHLLARHPAEQTRIRAELTRVLGDRTPTAADLDRLPRLTQAFKEAMRLY

PAAAVVGRRAVEATEVAGHRLPAGADVVVAPWVTHRHPGLWEDPERFDPD

RFAPEREAERHRYAWFPFGGGPRACIGQHFSMLESVLALATLLRSHELTA

VDQDVPVAAGITLQATGPARVRLRAL

>CYP122A(2546580560)Streptomyces albulus CCRC 11814

MPENPQAPQDGRTPRASEDTTNPPAPRGSDIYTSPEARASAPAPAPGAPP

LVDLRAYGQDFVTNPYPYYAKLRAQGPVHPVLTPYDQAAWLVVGHEAVRT

ALADPRLGKDWSSANLPRTDGDGVPLFTNMLDVDPPHHTRLRKLVAKEFT

SRRVEALRPRVQRITDELLDAMLTASDDRADLIEALAFPLPMTVICELLG

VPSMDRDAFRGWSHELVSPTSPEAAQQAVEAMSGYLTTLIASLRREAGDG

LLADLIRTSDEDGDRLSPEEVIGTAFLLLVAGHETTVNLIANGVRALLEH

PAQLAALRADDSLLDNAVEEMLRYDGPVETATWRCAAEPVELGGTVIPAG

SAVLISLASASRDPERFADADDFDLTRDPRGHAAFGHGIHFCLGAPLARL

EGRIALRSLLDRCPGLALDDAAEPATWRPGLLMRGTDRLPVRWDR

>CYP183J(645407868)Streptomyces viridochromogenes DSM 40736

MTAESPPIPRAAGSLPFLGHMIPLIRDNLGFIASLRRDYGPVVEIILQPR

HRTVIVQDPELIQVMLKDLSPHLDKGRFFEKMGQLLGDSVVTAAGKVHRD

KRHQLQPAFARDEIARYVDKMRDEVTAAVESWKPGQAFDVREAMVKLSLD

MLAKTVLSDSLDDGAFRRVRQDLSVVMAGVGARVMMPDWVEKLPLPANRR

FNRARDAVRATIEDAVDKLQAERGATGDHGADDMLSMLLRANMTRHQICS

EILTLAVAGTETTASVLSWTLYELTRHPDIEARVLAELDEVLAGRPVTFE

DVVRVKGEARLPYLQRVLDEVLRLHHTGLLVTRRTLTETRLGEWTLPAGT

ELAYCQHALHRDPDLFPDPLSFNPDRWLDTKQPPPSGAYLPFGHGKHKCI

GDFYARAELTTAVATILRRRRFDLAPGQTVRPVGGATVRPGKMVMTVQAR

EAPGRA

>CYP154AR1(645407871)Streptomyces viridochromogenes DSM 40736

MPIHEVTPAPETALSLARPVATLDPNALDVPAEGEALRRLGHGLVPVALP

GAPGQPTVRAWAVTDHDVAKAILDSKDFAKSPEAWEAYPEQLPEPFPLLQ

VITAPLLSNDGSDHRRLRSLISKAFTARRVDALRGRVEEIAQELLVDIPT

SERVDLRRHYARPLPIRVISELFGLKDLGDRERLADACDALLDSHVTKEQ

AGTAHTVIHEVISGLIERKRHQQGDDLTSALIRARDDEDRLSHQELHEML

FLLLIAGMETTQNLIVNGALALLDHPAELSLLLAGVASYATAVSETLRYD

SPLNTLMFYFAVRDVTIGEVTVCKGEAVLFCLTAIGRDPHTAPDPDVFRL

DRPPAQMRHLSFSYGPHYCLGAQLAQMEAEIALRVLFTQRTVTTQESRAS

LPRMASLSSQSAACLPVQLADWLAA

>CYP1416B1(645407872)Streptomyces viridochromogenes DSM 40736

MTYSPHSVGYQSVALPLPTCPEHPGRVQLSGLSLEPTVYDRMREAQGNVV

EVELEDHVPAYLVIGSEEFREACRRTDRFTPDSRAWSEWGQVKPGWPWKP

QVAYLQGSARFATGDEHWQLRNLLSSGLEKVSTAQLRAFTVREADRLIDR

FCQSGKADLVADYAIPLPLLVMLRLVGLPHDAGQRLLMLMPRLLEGGEGA

QAANDEINSILDELVAARRDQPANDYTSWLIHHSSSADAQQLSDAKVRNL

VWTTVLAGFGGCANWISNVMEQLVRKTRFQALLAAGEATVDEIMVETCWD

NPPVANVMGPFALTDSVLGGRLIPRGAMLIRGIAASNTDPTLSGDRHAWT

LGNESHNGWGAGPHECPAQRTAKIIVRTAVKAFLARCWAPVLENPEKPPK

RSSFIVRALDELPVSFAPSVPVLARPTPDQTALGDAGWNSRSLFPRQMLD

ELQCPFTR

>CYP156H(645408099)Streptomyces viridochromogenes DSM 40736

MTHPSPAEQAFSLAAPVRLWEDGFARDPHPYYAALRAQGPVGWAELAPGV

PAYVVTDRRAALDLLHDTETFSHDPRPWEATVPDDSPVLGMMRWRPNTLF

ADGAAHIRYRTALLDAFDLVEPHDLRAGVHRAVHLLVGRFGPRGEADLVG

EFTRPLMALVFNSLFGLPESASDRLDAALGKLIEGGAQAAQGEAEYAGYV

LELIAAKSERRGNDLPSWLLDHPAGLTPEEVTWQVFLTLGAGHEPTANLV

SNALSRILGNPAYYSTLTSGARPVTDAVLEVLHHETPLANYGIHYARTPV

TFHGAWIRAAVPVVISYGALAQAAEQERGGDRHPGDASHLSWSAGPHACP

VKQHTLLIATEAIERLTQWLPDLEPVLPRERLTWRPGPFHRSLTALPVRF

SPRSPDQPGGRP

>CYP154U(645408100)Streptomyces viridochromogenes DSM 40736

MTVTDRIALDPFGADIAAESARLRALGPIVPVELPGGIPAWAPTRYDTLK

ELILDPRVSKDPRLHWRLWPEVGEHPSWGWILGWVGVVNMLSAYGADHTR

LRKLVAPSFTHRRTEVMRPRVEAITAELLDALDGTDDEVVDVKEAFAHPL

PMRMICELFGVPDELRKDTGRMIAAIMDTTDPSPEHAASVQRQIGTVLPA

LVAHKAAHPGDDMTTELIRVRDEDGDRLSDEELLHTLLLVIGAGYETTVN

LIGNAVVALLRRPEQLAAVRSGEIGWDAVVDETLRAHPSIASLPLRFAVS

DLTVGDVTVPAGDAIITTYAAAGLDPEHYGPEADVFDATRGSDDHLAFGI

GVHRCIGAPLARLEALTALPALFDRFPGLSLAVGEEGLRQVPSFVAYGWQ

EIPVRLRD

>CYP161H10(645408758)Streptomyces viridochromogenes DSM 40736

MSVATERQQLPFPRPNVLDLAPFYDVLRREAPVTPVTTPAGDPAWLVTRF

EEVRDLLGDRRFGRSHPEPERASRLTSAAVLDGPTGNYETEEADHTRMRR

LLTPAFSAKRMRMLSDHVQHLVDRCVDELIAGHAASPDRVVDLQSGLAFP

LPVAVICKLLGVPESDRERFASLSERMADYAIGDAAHQARDEFNEYMTAL

AETKRARPGEDVISDLVQAQSADADYDYADMIRLCVGLLFAGHETTVNRI

GLGTLFLLTRLDQWEALTADPDGRVNATVEEIMRLGAPGDLGLLRYAHTD

VDIAGVTIRRGDAVVLSINSANRDASVYRDAETFDPDRSERTHLGFGHGV

HFCIGASLARVELRIVFATLARRLPGLRLAKGLDELEVRTTLTGGVTELP

VTW

>CYP183X(645408809)Streptomyces viridochromogenes DSM 40736

MACHPDLVHQVLRDPDTFDKGGPLYDRLRTLLGDGVGTCRQRDHRRQRRL

LQPGFRKARVADQVRMMGEEVESVCRTWRDGQAVDISAAMLDLSTRLVSR

VLFSDSLDPATALEMRRCLATVTRGMFVRTVLPVDALFRIPTPANRRYRH

ASEGLRAIVDAAVAERRRGSAHDDRDDLLGTLLAAARGDGGETVISDEEV

HGQVITLLFAGAESTALCLSSALALVALHPEQEDRLRSEADAVLASGRPP

GPDELPLLEHTRRVLTETLRHRPPGWLFTRVATKDTELAGHRLPQGATVM

YSPYLLHHDPALFPDPDRFLPDRWLPGRTDAVHSAAMMPFGAGRRKCLGE

TLALAEATVAIAFIARHWRLRHLPDYEERLRPAASLGPRGLFMSCERRAS

RQVDAPWQVDSRAPRKAAAPVVHNSRTAGGKDVHDT

>CYP182B(645408813)Streptomyces viridochromogenes DSM 40736

MSEGGKTMESQWGDGGSVDLDDTRLETLDPRPLLTRDYETRPALVYERLR

QQHGPVAPVDLLGVPAWLVLGYRESLQVLQDDDGWPKGLENWRARTEGRV

PADWPLGPSLEVNHILIQGGPGYRPLRTAWDVALKPFQDPRHPQAKRLKA

AVTAYADELITLVGQGGRTGVADLSAQFSRPLPLMVASHLLGFPGSQGDD

ALMDMWRVLDAGPDAEAALERLLATLAELAALKLQKPGDDFPSYLLAAYP

DLSLDHLARELFMLLGMTSDHVGILISNTVVEVISGEGSVRASLSAGMVR

ESMNRVVMRKPPLVNFVPRFAARDTPLGNYTIRAGDPVWVSPGAAHADPL

FADHVASGTTISTRAHLSWGAGPRQCPARELASTVAAAGVGRLFERFEHL

DLALPADQLPWRSSPFMRGLRSLPVRYELAATPAGPPVGGLAPEAAGEVL

PDPSARQRSSLWRYLTGLIRGGR

>CYP157C(645409354)Streptomyces viridochromogenes DSM 40736

MTPESHAPTGTDASRTGPPPGCPAHGLGPGGLHRLHESEDLGALYEQLRD

EHGPVAPVLLHDDVPMWIVLGHAENLHMVRSPSQFCRDSRIWNPLKEGLV

KPDHPLMPHIAWQPIASHAEGDEHKRLRGAVMGAISTIDFRSLRRYINRS

TQAIVNRFCEKGEADLVSQFTEHLPMAVMCEILGMPDAYNDRLVEAARDA

LKGTETAIASHSYVMDALSRLTARRRAQPEEDLASYLITHPARLSDDEVR

EHLRLVLFAAYEATTNLLSNVLLTVLIDPRFRAQLNGGQMTVPEAVEQSL

WNEPPFSTVFAYFAKQETELGGQRIRRGDGLLFAPLPANVDPRVRPDLSA

SMQGNRSHLAFGGGPHECPGQDIGRSIADVGVDALLMRLPDVELDCGEEE

LRWTASIASRHLVSLPVQFLPKPQQDVTETPNHNHVPAQRSDWQIGSVQP

RLQPAPAAPQPQPQPQPAPAHPHPQSPVAEPVRRKGVWPRFLRWWRGY

>CYP125A(645410159)Streptomyces viridochromogenes DSM 40736

MPCPALPDGFDFTDPDLLHHRVPLPEFAELRRVEPVCWVPQPAGLAGFQD

EGYWAVTRHADVKYVSTHPELFSSYLNTAIIRFNEHIERDAIDAQRFILL

NMDPPEHTRVRQIVQRGFTPRAIRALEERLRNRAHAIVEKARAHTEPFDF

VTQVACELPLQAIAELIGVPQEDRDKIFDWSNKMIAYDDPEYAITEEVGA

ESATEIIAYAMNMAADRKQCPAHDIVTQLVAAEDEGNLNSDEFGFFVLML

AVAGNETTRNAITHGMHAFLTHPGQWELYKRERPATAAEEIVRWATPVAA

FQRTATEDTELGGKRIRAGDRVGLFYASANHDPEVFDEPDGFDVTRDPNP

HLGFGGGGPHYCLGKSLAVLEIDLIFNAVADAMPGLRLVEDPRRLRSAWI

NGVKELRVSTG

>CYP1215A(645410299)Streptomyces viridochromogenes DSM 40736

MHTHASPASLLSALRPRTQERLYDFYKELRSADDLFWDPRLDAWIGTSHA

VVSSAAGDPRFSSVRYPDIEAVSEELRPLARVLSRQMLYSDAPDHSRLRA

LLSRAFTPRAVATLRDRIAGAVEQIITRAAPTGRLDIVADLARPLPLTVI

CDLLDVPEQDRPALSSWSDPIAAAVGSSRLDADDSRAASQSMARMLAYLR

ELLTRQDSPPAPHTLRALLTTRAESADQDMDELLANCALLLIAGHETTTH

FIGNAALALLRHPQAADQLRRRTDLIPAAVEELLRYDSPVQLMLRRARHD

LDLAGRSIAAGQVVLLVCGAANRDPAAFPDPDVLDFERPGGRHVAFGYGP

HFCLGAALARLEGAVVLEALLTRLPDLRLDGTAPQWQRSLNFRGLTRLNV

AFTPVFG

>CYP107U(645411069)Streptomyces viridochromogenes DSM 40736

MTDQPQPSTPAAPDLFTWEFASDPYPAYAWLREHSPVHRTRLPSGVEAWL

VTRYADAKQTLADHRLSKNPAHHAEPAHAKGKTGIPGERKAELMTHLLNI

DPPDHTRLRRLVSKAFTPRRVAEFAPRVQELTDRLIDGFAQKGSADLIHE

FAFPLPIYAICDLLGVPREDQDDFRDWAGMMIRHQGGPRGGVARSVKKMR

GYLADLIHRKREALPAEPAPGEDLISGLIRASDHGEHLTENEAAAMAFIL

LFAGFETTVNLIGNGTYALLTHPEQRERLQRSLAAHDTALLETGVEELLR

YDGPVELATWRFATEPLTIGGQRIAAGDPVLVVLAAADRDPERFADPDVL

DLSRRDNQHLGYGHGIHYCLGAPLARLEGQTALATLLTRLPDLRLATDPA

ELRWRGGLIMRGLRTLPVEFAAAKSDETSGL

>CYP154K(645411881)Streptomyces viridochromogenes DSM 40736

MAQQACPFLTIEPSGQDLYGEISRIRDQGPAVEIELPGGVRAWWINGLEL

NKRLLAGPETSKDAFQHWPAWINGDISRTWPLAIWVSVRNMVTAYGSDHS

RLRKPMAAAFTKRRVDALLPRVQEIVDRALDDLERIPAGEVVDLRAAFAA

PVPHEVVCELFGVPRGEDGPRRALYRIIDSFFDTAISLEDAQANAVDLYG

TLTAFLQDKREHPADDLTTALIAARDEGSLSEQELMDNLILLLTAGFETT

VNLIDNTVHSLLAHPDQLELVRSGRVTWEDAIEESLRFEAPGAMSGLRYA

VQDIEIEPGLTIPKGDPLVVSFAGAGRDPERHGPDAERFDVTRATSRDHV

SFGHGVHHCLGRPLAMAEATAALTSLFDRFPRLALADPARPPKRLRSIIS

TGHQELPVLLHGRNPGPDLGPRAEEAGAIRKLSELRGTSGTKVPRA

>CYP107P(645412145)Streptomyces viridochromogenes DSM 40736

MAAFDPWDPAFLADPYPAYAELRAQGRVHYFEPTDQWLVPHHADVSALLR

DRRLGRTYQHRFTHEEFGRTAPPPEHEPFHTLNDHGMLDLEPPDHTRIRR

LVSKAFTPRTVERLKPYVRGLADELASALVAAGGGDLLKDVAEPLPVAVI

AEMLGIPASDRAPLRPWSAEICGMYELNPSEETAARAVRASTEFSDYLRE

LIAARREEPGDDLISGLIEAHDEGDRLTEQEMISTAVLLLNAGHEATVNA

TVNGWYALFRDEAQLSALRADHSLVPAAVEELMRYDTPLQLFERWVLDEI

EIAGTTIPRGAEVALLFGSANHDPTVFKNPDRLDLTRPDNPHISFGAGIH

YCIGAPLARIELAASMTALLEKAPALRLAQEPERKPNFVIRGLEGLSVAV

>CYP113R2(645412262)Streptomyces viridochromogenes DSM 40736

MLDTQPVYRDPEKGWQVFGYSDISRILADTTTFSSDTARAFNPPQPDLDF

FDMGNLVTTDPPRHRQLRSVISSVFTARAVTGLTPRIEKITNALLDGVEG

ADRFDLIDSLAYALPINVILELLGLPVEDEPLFRVWGEALGTVDAATVPP

EQLENEVAPAVREMNAYLLEHVRRLRKNPTDDVLSRLAGAKVDGTYLDDG

EIVGVTGLTMFAGHATTMALIGNAVLLFDRHPEVAGAVRADRSLLPGALE

EVLRLRPPFPRLARITTADTELGGHTIPAGSLVTPWIGAANRDGSRFPDP

DRFDIHRTTSHLVFGQGIHFCLGAPLARLEAKIALNILMDRYRDIAVDET

GSLEFENPRQLISPTRLPVRVSG

>CYP170A(645413209)Streptomyces viridochromogenes DSM 40736

MPFLGHGWKLVRDPLEFMSRLRDHGDVVRLKLGPKTVYAVTTPELTGALA

LSNDFIIAGPLWESLEGLLGKEGVATANGPVHRRQRRTIQPAFRLDAIPA

YGPIMEEEAHALTERWKPGETIDCTSESFRVAVRVAARCLLRGDYMDERA

ERLCVALATVFRGMYRRMVVPLGPLYMLPLPANREFNRALADLHLLIDEI

VAERRASGQKPDDLLTALLEAKDDNGDPIGEQEIHDQVVAILTPGSETVA

STIMWLLHMLAEHPEHADKVREEVEAVTGGRPVAFQDVRNLRHTNNVVVE

AMRLRPAVWILTRRAVRDTELGGYRIPAGADIVYSPYAIQRDAKSYERNL

EFDPDRWLPDRVKDIPKHAMSPFSTGNRKCPSDHFSMAQLTLITAALATS

YRFEQVAGSNAATRVGITLRPHDLRLRPVRR

>CYP107AH(645413387)Streptomyces viridochromogenes DSM 40736

MTQAFASEGMSSEEAAAAASSCSREFRANPHPVYAALREAAPVCPLSPPH

GVDTYLITRYDDARAALADPRLSKDMYGAIDAYHRIFGDSSIALDDNMLF

SDPPKHTRLRRIVGATFTPKRVESLREQVQKITDGLLDRCPASEPVNLLT

EFCFPLPLHVICELLGVPENERKQAQEWSATVAQTGFGPEARQKLEIAEG

NLRDYLVDLIARKRREPDGALLSALVTAQDQDGALTDGELVSTAWVLLFA

GHKSTAYQIGNAVYHLLSRPEQKRLAMTDPEAMAAAVEEIFRFETSVENS

TFRYAKEDIKIRDVLIPKGALVQISLTSANRDPEMFPEPDRMDIERPNVQ

ATHLAFGLGPHYCIGAPLARLEMQIALGTLFGRHPDMALAVAPEDARWLT

VPFPAFRGLAELPVVLDPS

>CYP229K2(645413872)Streptomyces viridochromogenes DSM 40736

MLGHALRLARRPHQFLSTLSALGPVVRIKIGGYLGYVVTEPGLIRKAFVT

HSDQGILAERTRPLTGEGVIVLQGQRHRQERRLIAPAFAKARIADYASVM

ARQGAECAQSWRDGQEILLNEEMHELAVRTMAATLFQGTLGAETAGHIHR

LLPPVMTLLVRRGTRPAWTDRLPLPSNRRFEVMLAELKAVTRTIITSRRA

ELAADPEHDPGDLLGALLQARDDESGAMLSDTQVHDELLNFLVAGTQAAA

ATLAWIFHELSANPNVEAALHAELDAVLTNGRPAEFADLERLDVTKRVVT

ESLRKYSPWLTLRQITEPTTLGDVEIPGGVLLFACPIAVHRDPAFHPDPM

RFDPDRWLPQNRARMSPDTYIPFGMGARQCPGNVFALTQITLQIATIAAR

WRFRTIPGSEVKEVAIGAFIQPTRMPMRAEARF

>CYP154A(645414126)Streptomyces viridochromogenes DSM 40736

FCYPLPIQVIGELLGLPEELGPELRAVVDGVFHTSADAAEVTDIYARFYA

VLGELVAAKRASPGDDLTSALIAAHEEESGTRLSEQELLDTLMLVISAGH

ETTVNLLDNAVHALLTHPDQLAHVRDGRATWDDVIEETLRAEAPVASLPL

RYAVEDLPLGELGGPDGAVIRKGEAILASYAAAGRDPEQHGPDAARFDVT

RAAKDHLAFGYGVHHCLGAPLGRLEARVALPALFERFPGLALAVPSDELR

PVDSFISHGHRSLPVLTG

>CYP105AC(645414281)Streptomyces viridochromogenes DSM 40736

MMMSDSLHTVTTLPTTRRAGCPFDPPAELLEAREHGPISRFTHPGGKPGW

LITGYDLVRSVLADSRFSSRKELMNVVDFELPPAPPGEFLLMDDPQHRRY

RKPLAGKFTVRRMRLLTERIEQITADCLDAMERTGPPADLVTAFAKPIPT

IVICELLGVPYEDRGSFQEQIDTFMGGETSDEELIAAYTATQEYLAKLVA

AKRAHPTDDVLSELTESDLNDEELKGISLVLLAAGFDTTANMLSLGTFAL

LQNPAQLAALRADPELTDQAVEELLRYLSVAKTFMRTALEDIELGGQTIE

AGTTVVLSYHTANRDPERFADPHVLDLRRQDTGGHLAFSHGIHQCLGQQL

ARVEMRVAFRALIDRFPTLRLAVPAGEVGLRPETADIYGVKSLPVTWDAT

S

>CYP102B(645415153)Streptomyces viridochromogenes DSM 40736

MTEAPIRAGMPKGFRSAELGWPELDRIPHPPHRIPLLGDVVGVNRRTPLQ

DSLRYARRLGPVFRRKAFGKEFVFVWGARHTADLADESRFAKHVGLGIAN

LRPVAGDGLFTAYNHEPNWQLAHDVLAPGFSREAMAGYHPMMLDVARRLT

EHWDRAAGAGRTVDVPGDMTKLTLETIARTGFGHDFGSFERTRPHPFVTA

MIGTLTYAQRLNTVPFPLAPLLLRSATRRNAQDIAHLNRTVDELVRARRT

DGGGQGDLLDRMLETAHPVTGERLAPQNVRRQVITFLVAGHETTSGALSF

ALHYLSRHPDVAARARAEVDRVWADAAEPAYEQVARLRYVRRVLDESLRL

WPTAPAFAREAREDTVLAGEHPMRRGAWALVLTPMLHRDPEVWGADAERF

DPDRFDARAVRSRAPHTFKPFGTGARACIGRQFALHEATLVLGLLLRRYE

LRPDPAYRLRVTERLTLMPDGLRLRPERRSAVREPASELRCPVPGAGD

>CYP102G(645415160)Streptomyces viridochromogenes DSM 40736

MRPTAQSPRPADSLPGVPVADITATGPGGAPIQQAMDLMREHGPVFVRRL

YGRDTLFVGDLDLVAELADEQRFAKHIGPGLENVREFAADGLFTAYNDEP

NWAKAHDILMPAFALGSMRTYHPVMLRVAQRLIASWDRDARAGQPVDVPG

DMTRMTLDTIGLAGFGYDFGSFERAEPHPFVESMVRCLEWSMTRLARVPG

KDYSAADAAFREDSAYLAQVVDEVIAARTGTDQSQADDLLGLMLTAEHPD

DGSTLDTANIRNQVITFLIAGHETTSGAMSFALYYLAKHPTALQLVQREV

DELWGDTADVEPTYDEVGRLTYTRQVLNEALRLWPTAAVFSREAREDTLL

GGRIPLRAGQAALVLTPMLHRQPVWGDNPELFDPSRFTAEAEAERPVHAF

KPFGTGERACIGRQFALHEATMLLAMLVHRYRLRDHADYRLDVKETLTLK

PEGFTLALAPRTCADRVHTPLPGAIQAQDAESAEPQGLPARVRPGTAALF

LHGSNYGTCRDFAAQLADEAAAVGCETEVAPLDAYAGGLPTDRPVVITAA

SYNGRPTDDATAFAARLEETHDLSGVTYAVLGVGDRNWAATYQHVPTRID

ERLAESGATRLLDRAAADASGDLTGTVREFTAALRTALLQQYGDPDATAP

DTGEPTAAYEVRTLTGGPLDALAERHGLVPMRVTEAHDLTAPGYARRKRF

VRVALPEGVTYRTADHLTVLPANDPGLVTRAATALGVDLDEVLDIRATRP

RRDGIAVDRPVTVRQLLTHHVELQERPSTGQLAALAAANPCPPERMALAA

LSDDPRTLVEIIEDHPALRGALDWPRLLDLLTPLRPRHYSISSSPAVDPG

HADLMVSLLEAPARSGKGVYRGTGSGHLNTVEPGDIVYARVQPCREAFRI

DAADASAPVVMIAAGTGLAPFRGAIADRTAALATGARTGPGPLL

>CYP157K(645415187)Streptomyces viridochromogenes DSM 40736

MNDQPESPLQAPRGCPVAHHAAELTRLYGPEAATDPGGIYERLRKEHGSV

APVLLEGDVPAWLVLGYRDTRRVLDNPRQFSRDARIWRDWTQGRVEESSP

LIPMLGWRPDCVSQDGEPHRRLRSAVTDGLQAAAVRGIRRHATHFANKQI

DAFAATGRADLVTDYAEYLPMLVLSRILGLPEAEGRDLVESCAQVLKGGE

DALAHNDRIVAILGALAERKRTEPGGDFTTALLGHAAGLDEEEVVSHLRL

VLIAAHTTTSNLLARVLQLILTDTARLSGLISGQLNISGVVEEVMWNSPP

LAVLPGRFAAGDLELGGHQIQEGDLLVLGLAAGNVDPEIRPDTGVSVQGN

QAHLAFSSGPHECPGQGIGQSIIEIAVDVLLHRLPGLTLAVPPDELTSTA

STWESRLDSLPVEFPA

>CYP105D(645415196)Streptomyces viridochromogenes DSM 40736

MTDTTAPVAFPQSRTCPYHPPAAYEPLRTERPLTRISLFDGREVWLVSGH

ATARALLADPRLSSNRDRPGFPSPTARFAGVRNRRTALLGVDDPEHRVQR

RMVVGDFTLKRAAELRPRIQQIVDERLDAMIAQGPPADLVSAFALPVPSM

VICALLGVPYSDHDFFEAQSRRLLRGPETADVLDARDRLEEYFGELIDRK

REEPGTGLLDDLVQRQLSDGALDREGLIAMALILLVAGHETTANMISLGT

FTLLQHPDRLAELRADPGLVPAAVEELMRMLSIADGLLRLALEDIDVAGT

TIRKGDGVVFATSVINRDETVYPDPDTLDFNRSARHHVAFGFGIHQCLGQ

NLARAEMEIALHTLFDRLPTLRLAAPAEEIPFKPGDTIQGMLELPVTW

>CYP107BT5(2515928940)Streptomyces sp. LaPpAH-95

MTSTPTPAGVRDIAGPAPVRDLGAALLTPEARRDPYPLYARMRREDPVHR

SPQGVWYLTRYADVEAALGDLRLSNDRDRMTRAYTALGGDLKEFSRLTDR

LGRVMSNTDPPDHARLRKLANRAFTARRVEALRDRVQHLVDRLIDAAVAA

GPEMELIEAVASPLPMSVVCELFGIPEADRPRVKDWFRRFGRLSEDLAKS

ETAIDQYEDYLSGLIRQRRREPGDDLISALVATQTHDDRLTDSELLSTCF

VLITAGDETTTHLITNAVHALLRHPDQLARLREDPGLMRGAVEELTRYDT

VTQAIVRVVAQDLEIGGRILCEGELVYLFLGATNRDPERFEDPDRLDLTR

PGNRHLGFGHGPHFCLGGPLARLQTEVAVGTLVRRLPGLRPADGAELSWR

PNPLQRRLNTLPLTY

>CYP157C(2515929332)Streptomyces sp. LaPpAH-95

VTSPFPYEHGQVPPPECPAHGMSVGPGGLRRLYGPEAEQDPHGLYEKLRA

EHGTVAPVLLHGDVPAWLVLGHSENMHMTRTPSQFSRDSRRWRALQDGSV

APDHPLAPIFTWQPICVFADGATHERQRGAVTDSMARIDTRGVRRHVNRY

SNRLVNGFCREGRTDLVAEFAERLPMMVMCAILGMPEEYNDRMVEAARDM

IKGTATAVQSNAYVVGALTRLVERRRREPADDFATWLVEHPANMNDQEVA

EHLRLILIASYEATANLIANVLRMVLTDPRFRARLSGGHMTVPEAVEQTL

WDEPPFTAVFGRWAVGDTELGGKHIKAGDALIVGIAPANTDPVVRPDLTA

NMEGNRSHLAFSGGPHECPGQDIGRAIADVGVDALLMRLPDLELAVDERE

LQWVGNFMSRHLVDLPAEFAPRSPQDDREPPAMGRPNAARQDWEVQSAVR

HTAPTPVRAPLDGAHAPSAAAPAGATATVDGSADALSGRVPRQRKPGGPA

RLWRVVTRWWNGD

>CYP154C(2515929361)Streptomyces sp. LaPpAH-95

VTCPHAARAAADPGATVVIDPMVQDLDGETARLREAGPLARIELLGVPVW

TLTRHADARRLLVDPRLVKDIDAWGLWQSGEVTHAWPLIGMIDAGRSMFT

VDGAEHRRLRTKTSQALTPRRLEAIRPDIEKFTAELLDALEEGGRDGAVV

DLKSVFAQPLPMRVVGMLMGVDPAENAMLTRQYKKFFSMLTPQDERLALL

ADLDVFYAGLVREKTAHPTDDLTSALILAEEGGEPLTEEEVVGNLKAMVA

AGHETTIGLILNAVRALLAHPDQLRMVLDGKVSWDTVIEETLRWDTPTTH

LLMRFATEDIQVGDSVIAKGEGVVISYRVIGRDPEQHGPDADAFDITRST

PIRHMTFGHGPHICPGAALSRVEAGIALPALFARFPELRLAIPDAELRNL

PVMTQNDMESFPVLLAG

>CYP157A(2515929362)Streptomyces sp. LaPpAH-95

MTRPSPAESAPVSPDAPSGCPAHAGAVPLWGPGFQAEPQSVYRAMRREHG

PVVPVELLGGFPAWLVIGYRELHRVTSDGELFPRDVGLWNQWPNVPDDWP

LLPMVGRPLPSIYFSAGKEHLRHARMVTPALEGADPFRIRQHCEQLADRL

IDAVCSRGTADLVAEFCEPLPVLVLARLVGFPDEEGADIARVLKDLADGG

PEAQSAHLRFGEHMARLLAAKRAEPGDDVTSRMLDFPEEFTDEEYTLDLM

AITAAGHLPTADWISNSVRLMLTEDEFADSMAGGRRSIGEAMNEVLWEDT

PTQILAGRWAARDTQLGGQRIKAGDMLLLGLGAANTDPLIRQGVSGGPGP

AQGGNGAHLAFSHGEYRCPFPAQEIAEIIARTGIEVLLDRLPDLRLDVAV

EDLVRRPSPFLRGNTTLPVRFTPVRTTGEFS

>CYP1813A(2515929580)Streptomyces sp. LaPpAH-95

MSHATTHEFSQYAEVLAALADPALVPPAPGPFEGPPGASVAWLRATVARF

ASGEPHRRRRALVEAELARIAPADVHRAASASASGEGDLRTRVVRGLAAA

LDMPEPDRVAREVAVVADAYFGEDGGAEADRAVARLVGLLAPGPADEAGL

EAVANRIGLLAQACAATAALAGSVEAAGPGAPTARVLRDDPPVRVMRRVA

ARATRVAGREIAEGDAVVLDLVAARQGHPVPLTFGAPPRACPGRAHALAL

AEGLLGRPMTPFARLHHRGEAFLLPNAWDYASAAALAAEGFGAVGTTSLG

VAAGLGLPDGAAATKEATVNLARRLGRGPFLFSVDAEGGFSDDPAEVAAF

ARRLYEAGAAGINLEDGRADGTLAPMGLHAAKIAAVKEAVPGLFVNARTD

TYWLGLDRERTAGRLAVYEQAGADGVFVPGLSDRAGIAALTAALVTPLNV

LYNPAGPGIAELGALGVRRVSLGSLLYREALTGAVSTAATIRDGEPVRGG

ALSYAEVQALAPGDGSGGGGGDDVHDG

> CYP107L (2515929586)Streptomyces sp. LaPpAH-95

MSEVVVDLRGLADFTADPYPYYERMRATGPVHLVRTDEFDSIWLVVGYDE

GRAVLADQRFGKDWRALPGETGGDPINANMLETDAPDHTRLRKLVARAFT

PRRIEALRPRVEEITGELLDLMVPEGRADLVDALAFPLPMTVICELLGVP

DLDRAAFRKLSNGIVAPVNAEEEGEAVRAMGGYLAELIGDKRRSPGDDLL

SALIDARHEDDDALSPDELVGMAFLLLVAGHETTVNLISNGVRALLAHPD

QLAALRADLSLLDGAVEEMLRYDGPVETATLRFAREPVEVGSRTIGTGDA

VLVSLAGSDRDPARYPEPDRFDIRRDSRGHLAFGHGIHFCLGAPLARMEG

RIAIRALLERCPGLEADPEAAPFDWLPGTLIRGVRRLPVRW

>CYP159A(2515929763)Streptomyces sp. LaPpAH-95

MQADAPRDIPAPTTSPEPRAHATRSARSRTTGRACTGPSLLARGAARDPY

RFYRMLREQYPLSYDAPLGAWLVSRYDDVTTALADPRFTGFPQDGAPRGG

PAPLGLCHGSPLCLPADRYTVVTGTVPDGLAERVERTAFVLARRIARSSR

ADLVEEFCRWLPAAPAGDGPCGRDTALRRTVLASLLANLLDDPDLLAALR

VEPALTGRAWTESLRRDPPVQVVLRRTVTEVALSGGTLPARAPVACLVGA

AARDPGRFAAPDAFDPFRPDQDRALTGPAGCPAVALSRLEAEQGLRALLD

AMPRLRWAEGFRPAATGLLTRGPRSLIVRPG

>CYP105D(2515930508)Streptomyces sp. LaPpAH-95

MTETQSEPQPRATQTTAFPQDRTCPYHPPTGYPSESRGQRSVSRVRLFDG

RTVWLVTGHAEARALLADPRLSSDRTHPDFPLFAPRLTASRQRRVELIGV

DDPEHNVQRRMLIPSFTVRRAAALRPRIQETVDRLLDAMVAQGPPAELVE

AFALPVPSMVICALLGVPYADHEFFEDRSRKLLRGPGPEDVVAARDDLDA

YFGELIERKRRDPGDGLLDELIAERLETGQVERDELVRLAQILLVAGHET

TANMISLGTFTLLRHPDQLARFTGGGAEAVPAAVEELLRFLSIADGLSRM

AVEDIEIGGETLRAGDGVLLSTAVINRDASVFETPDELDLGRGARGHVAF

GFGIHQCLGQNLARAELEIALPALFRRLPGLRLAVDPEEVPFKPGDTVQG

LLELPVTW

>CYP157A(2515930613)Streptomyces sp. LaPpAH-95

VTECPHAAGAVPLSGPRFQGDPVRLYREIRRDHGAVAPVVLDGGLPAWLV

LDYRELHQVTSDPVLFSRDSDLWNQWENVPADWPLLPMIGRKQPSILYTV

GPRHAERAAMIGNALEAIDSFALKRFAEEFADELIDRFCSKGSTDIIAEY

AMLLPARVLARMYGVSDADGDALVGSINDMIDGRERALAGQQHLAATMLK

LLADKHAEPGDDVATRMLADPGGFTDEEVMQDLMVMMAAGHQPTADWMGN

SLRLMLTDDRFAASLSGGRHSVAEAMNEVLWEDTPTQNVAGRWAARDTHL

GGRHVRAGDLLLLGIAAANSDPQIRVDGSALTGGNSAFLSFGHGEHRCPF

PAQETAEVIARTGIEVLLDRLPDIDLDVPAGQLTRRPSPWLRGMTSLPVV

FTPTPALGGRI

>CYP154C(2515930614)Streptomyces sp. LaPpAH-95

MTRIALDPFVADLDGESAALRAAGPLAEVELPGGVHCYAVTHHAEARQLL

TDARIVKDINVWGAWQRGEIPMDWPLIGLANPGRSMLTVDGADHRRLRTL

VAQALTVKRVERLRAGIEALTTASLDRLAALPEGERVDLKAEFAYPLPMN

VISELMGVDAADHPRLKELFEKFFSTQTPPEEVPQMMADLGALFTKIVDA

KRAEPGDDLTSALIEASEDGDHLSNEEIVNTLQLIIAAGHETTISLIVNA

VVALETHPEQRKQVLSGEVPWENVIEETLRWNTPTSHVLIRFATEDVPVG

DKVLPKGEALIVSFGALGRDEKQHGPTAGEFDVTRSPNRHIAFGHGPHVC

PGAALSRLEAGVALPALYERFPELELAVPPTELRNKPIVTQNDLFELPVE

LS

>CYP107P(2515931268)Streptomyces sp. LaPpAH-95

MDQPSTSPAPFDPWSAAFVADPYPAYAELRATGRAHYFEATDQWLVPHYA

DVSALLRDRRLGRTYLHRFTHEEFGRTPPPPEHEPFETLNGQGLLDLEPP

DHTRIRRLVSKAFTPRTVEQLVPTVQRLAAGLVDDFVEQGGGDLLAAVAE

PLPVAVIAEMLGIPESDRAPLRPWSAAICGMFELNPSEDTARAAVRASLE

FSAYLRELITERRAHPGTDLISALVAAHDEGERLTEQEMVSTCVLLLNAG

HEATVNTTVNGWWTLLRHPEQLAALRADHGLLPTALEELMRYDTPLQMFE

RWVLDDIEIDGTVIPRGSEVALLFGSANRDPARFAAPDTLDLARRENPHI

TFGAGIHFCLGAPLARVELAASFGELLRKAPGLRLAAEPEWNPGYVIRGL

KELRVEV

>CYP107CD(2515932229)Streptomyces sp. LaPpAH-95

MTTTDSGNSLSSCPYHASGFTSERLELDPAYARLRSEEPVTRVTMPHGGD

AWLVTRYADVRAALSDPRLSRAAAVGKDVPRSSPLIQQADSLLSMDPPEH

TRLRRLAAKAFTARQVAAVRPRVQAVIDSLVDALEESGPPADLATGLAWP

MGITVICETFGIPEEDRGRFRDWTDAMMALTVADPATIQAARDSLDGYLR

ELIARRIAEPGDDLLSRLVTSRESGDRLTESELGVFAVDLLTAGHETTAN

QTGNFLYTLLSRPRLWESLVKDPDLVPPAVEELLRYTPLATSVAPFSRIA

VEDFELAGQLVRAGDAVVAQTDSANRDGTVFENPEELDFHRENNPHVAFG

HGAHHCPAAPLARLELQTVIATLARRLPGLRLAVPADEVEWNTNRVMRGV

TRLPVAW

>CYP107U(2515933290)Streptomyces sp. LaPpAH-95

VNDSPAERPAEAASVPAGCPAHTSSAAVPPAPELFTWEFATDPYPAYAWL

REHSPVHRTTLPSGVEAWLVTRYADARQALADSRLSKNPAHHAEPAHAKG

KTGIPGERKAELMTHLLNIDPPDHTRLRRLVSKAFTPRRIAQFAPRVQEL

TDRLIDGFVEKGEADLIHEFAFPLPIYAICDLLGVPREDQDDFRDWAGMM

IRHGGGPRGGVARSVKKMRAYLLELIHRKREDLGDDLISGLIRASDHGEH

LTENEAAAMAFILLFAGFETTVNLIGNGVHTLLNHPAERERLQRALAAGD

SGLLATGIEELLRYDGPVELATWRFATEPLTIGGQRVAEGDPVLVVLAAA

DRDPERFAGPDTLDLSRSDNQHLGYGHGIHYCLGAPLARLEGQAALATLL

TRLPDLRLAGESGDLRWRGGLIMRGLRTLPVEFTPGARTEGSDTPSTL

>CYP105AK(2515934430)Streptomyces sp. LaPpAH-95

LSTTTFRSRITARLGRGYLARIQKHGFGSTTMKLLPEQLLMPLRRDGLDP

VRRLAETRAGAPVSRVALPFGMDAWVVTGYEESKAVLGSAEGFSTDFAHL

AGNAGIAAEHSPGGLGFSDPPVHTRLRRILTPEFTMRRLRRLTPRIDAIV

AERLDAMAAAPGPVDLVHAFALPVPSLTICELLGVPYEDRDDFQKLAMDR

FDLFQGATAPFGAMSESLDYFRGVVRDQRRAPGDGLLGMIVREHGDSVDD

EELAGLADGVLTGGFETTASTIALGALVLLRNPEVADRLRTDDAVTAPFV

EEVLRYLSAVQMAFPRFAREDIEVAGVVIPRGDMVLCSLSGANRDPAYVG

DDGRFDVDRVTPAGHLAFGYGIHRCIGAELARMELRSAFPALVRRFPAMR

LAVPPQDLAFRKLSIVYGVESLPARLG

>CYP105DW1(2515934489)Streptomyces sp. LaPpAH-95

MSDLVDACPHAQPRPFPLERTGCPLDPAPEYAVLRETEPVSRVKLKFNGR

EAWLLTRYEDVRQMLVDPRFSSNMADPGYPLQFHFPMELLGKVKPALLHM

DPPEHTAHRMMLMPELSVKRVEAMRPRTQEIVDECIDAMLEQGGPVDLVS

MLSMPVPSIGMCELTGVPHESRDLFHRWVTLLVTQGSAEEHASANAEVEV

LLYELIAERQKNPGDDLISSLLQRNDQKKELEPSDISALVRAMIAAGHES

TVNGITIGTLVLLQHPEQADWLRAHPELSGQAVDELSRYSSISDHGTVRV

ALEDAEIGGQLIRKGEGVICSLSASNHDPAVFKDPNTLDLTRREARHNVA

FGFGRHQCAGQMLVRMQLEVVFTTLLRRIPGLRLDAALDELPFKANALID

GVHELPVTW

>CYP154D(2515934649)Streptomyces sp. LaPpAH-95

MDPTGGCPHAANARLLERGSVVPVVLPGEVRGMAVLGHEALKDFLAHPDV

AKDARHFVALRAGEIPDGWPLKTFATVQGMTTADGDDHRRLRALMGRAFT

ARRVEELRPSVAELTGRLLDGLEAAADGDGVADLRTHFALPLPMGVICEL

LGVDEVHHDRLHALSSQIVATDIGPAEAMAANREMGEILSAVAAARAADP

GGDLTSAMIAAREEDGDRFSPHELIGTLMLTVIAGHETTMNLITNAVRAL

CAHRDQLDLVLSGGATWSDVVEETLRWDSPVSYFPFRYPTHDLTLDGTLI

PRGTPVLAGYSAAGRDPRAHGPDAAAFDLTRTGPRHLSLGHGPHYCLGTP

LARLEATTALEQLFTRFPALDLAVPEPELPRVRSFVGNSVRRLPVRLRS

>CYP154A(2515934671)Streptomyces sp. LaPpAH-95

MSEQPIIVLDPAGTDRHAEYRALRERGSATRVDVLGVTAWSVSDPVLLKE

LLTSSQVSKNARAHWPAFAETVATWPLALWVAVNNMFTAYGGDHRRLRRM

IAPAFSARRIQDLRVSVEKVVSELLDGLADRPAGEPVDLRLELAYPLPIA

VIGRLMGVPEDQLDGFRAKVDGVFDTTLTVEEATANTAALYGALDQLIAA

KRAQPGDDMTSLLLATRDEEGDGSRLSDEELRDTLLLMISAGYDTTVNVI

DQAITALLTHPEQLAHLREGRADWNDVVEETLRHEPAVKHIPMRFAVSDI

PLPDGQKIAQGEAILVSFAPANRHPDWHGETADAFDVTRTTKDHLAFGHG

VHFCLGAPLARLEIATALRQLFERFPDVSLAVPAEDLLPLPSLISNGHQA

LPVHLRPTAG

>CYP156A(2515934672)Streptomyces sp. LaPpAH-95

MTSPSITLPSAPSGRVALYDPAYAADPHAAYEQMRAVYGPLVPVELSPGI

PATLVIGYFQARRILNDPLYFPHDPRVWQEQVPASCPVRPMMEWRPNALR

SSGTAHARYRSANTTAIDAVDQHELRARVEKLAEAAIGDFITAGRADVLT

QYAWPIAFRVLSSLLGCPDEIGARIADGMSRIFEATDAQRGNEILGQAVA

DLVELRRRHPADDITSRLIGHAAQLTDEEMSHQLVTLYGAGIEPLTNLIA

NTQLKILTDEEFSAGLHAGHSTVRDALDTVLYTDPPMANYCITYPPYPAD

IDGVLLPAHQPVLISMAGCNNDPAIAEASGGIAGNRAHLAWSIGPHTCPA

RSHAYLIAETAVTYLLDALPEMDLAVPAAELRWRPGPFHRALESLPIVFH

ASH

>CYP1046A(2515934994)Streptomyces sp. LaPpAH-95

MSKETDPGACPVSREEPVSREEPGVAHSRVLRFWLDPANLAVRLGQAGPV

VRTRTGPAVAFQVNDPSLIRKVGCGEDIFQFWNADPSLRGFAENGFPGAE

GQAHRDRRALMKPALAAPRLTALGPSVRDSTERLIADLPTDRPVDIPVEM

SRLVAGLVIETVLNSAISPDTLTALAHARSVLSARVFWTYALSRWPWVPV

PRRRASRRAFATLNEAAREVYARHRPDADGNDVVSVLKRASEDRPDVALH

DVRALLFAGIEATASTLSWACYELGRHPDHQAAIRAEGDAVLGTDAPADT

IRPGLLPFTAGFVSEVTRLHGIPFLVRRPRHATHVGDQPVPAGALVTLPL

GALRRDPARYARPDEFDPLRWSPEARPPLSPAALLPYGIGPRYCPGAAAS

DILVPVALASLVRSRTLRTASTGRSVRVSLDLTPTPKGLAMVATPREPRP

PAPGQPPHEQAAPVRRH

>CYP156G(2515935066)Streptomyces sp. LaPpAH-95

MTTYAPPPQPLALFGPAFAADPQGHYRALRAHGPLAPVRIAPDVEALLVT

DYQAAIDVLRDTHTFSKDPRTWQATIPPDSPVLPVLGHRPTALFSDGAVH

ARYREAINDSLALIEPHVLRAEVARVARQLIGRFAATGSGDLIAQYARRL

PMHIFVTWFGADPKDGERIVDGIAGMMNSAADAAAAYADLVDVVTRLVAD

RRARPRRDLTSYFLAHPAGLDNDETVRQITLVMSAGNDPMTNLIGNATLH

MLTDERYAGSLHGGAMTAHEAINEVLWRDPPIANLAAHYPRHDTEFHGVR

LRAGQLLLVSYAAANSQSPPPVSDDGLRSGASAHLAWSAGPHRCPAKQPA

LLIAMTAIEQLTSLLCDARLAVAPDELLWRPGPFHRALAHLPIRFTPLDT

TAAPTGAITVAEPADVIPAAAITI

>CYP154M12(2515935094)Streptomyces sp. LaPpAH-95

MGKCPIVLDRLGKDIHAEARALRAQGPVTRVELPGGYFGWSVTDYAVAKK

MLLDPRFPKNAKKYWPPLVNGEVSMDWEMITWVMMENINTRDGEEHDRLR

KLVSHAFAPRQVEASRPLIEKVIGELLDDLETTEPGEAVDLKGRFTYALP

AWVICDMFGVPVEARKRMLQGAVANSKTTITPEEAEENLRQWHEAMAELV

ATKRAKPGDDLTSLLIQAREEDDALTDDELLGSLHVLLGAGSETLGNVMA

HAVVDLLSHPDQLELVTSGKADWATAFAETVRKDAAVAQLPFRYAAEDVE

IGGVRIEKGDLVMIAFAGVGRDPKVHGETADAFDILREDKTNLSFGHGPH

ACLGRSLATLQATLALPALFERFPNMRLATAPEDIPPQGTFIMNGYATLP

VVVH

>CYP208A(2515935127)Streptomyces sp. LaPpAH-95

VTATRAEATGIRRVPPGPSRLATFKLLNMLLRDRLALMRTAADGYGDAVR

VAIGPKTLYFFNHPDYAKYVLADNPANYHKGIGLHQARRALGDGLLTSEG

ELWREQRRVIQPAFQAKRIAAQAEVVAEEAARMVGRLRSRTGTGPVDITG

EMTELTLGVLGRTLLDEDLSGHTSIGHDFEAVQDQAMFEVVTLGAVPQFL

PLPQQLRFRAARARLQKVTEKLAAERASRSGGDTDDVLSRLLESVHAEQD

PQVARRRLRDELVTLLLAGHETTASTLSWAFHLIDQDPEVAERLRDEARS

VLGDRLPEYEDLHRLTYTSMVVEETMRLYPPVWMLSRIAQAPDVIAGYGI

PRGADVVVCPYTLHRHPEFWERPERFDPERFDRRRRAQRPRYAYIPFGAG

PRFCVGNHLGMMEAVFVIAMVMRELRLVLPKGASVVPEPMLSLRVRGGLR

MSVRKA

>CYP157J(2515935329)Streptomyces sp. LaPpAH-95

MSLTPPPACPAHDAQSTGLMPLSAAVGSADPQAAYRRLRSEWGNVAKVEL

EPGVPAWLVLGYRELLTITRQEQLFSRDARNWRDLNNGLVPLHSGLLPMM

AWRANVIGADGPEHRRLRMPLDNAVAKMDQRRVRREVEALCTELIAEFSP

RGRADLVNEYATIVPMLSLASLFGLDTTEGRELLDALIALFGSADNSQSG

NRNFEQIILDTVRERQRNPTSDMTTAFISDPNLRNEAEHLQSVVVMISAG

NETVTAWISHTLRLMLTDPRFAARLRGGRLGIDDALDEALWRDPPMNNMP

ARYALHDTELGGRVIRRGDCLILGLCGANDDPVIRPGNDEESWGNRAHLA

FSAGPHVCPAQVPARLITRTAVQTALHLLPDMRLSIPAEEVTWRPSPWTR

VPMSLPAEFSASRIGADIRR

>CYP154T(2515935330)Streptomyces sp. LaPpAH-95

MTRTDSSEGHIPVVALDPRGTDHHAEAARLRSLGPVVRVRLPGDVMAWSV

TEHALLNDMVADPRFSKDWRNWSAIVRGEIEDGWPLIGMVKVTNMVTSDG

QEHRRLRKLVTQTFTPRRVQEMRPRIEQIIDDLLDALPGRVDADGSVDLR

RHFAHPVPMQVICELFGVPSHERQRLQELMDNIFRSDLPPAEVTASQIEQ

YQLLGRVVEARRGDPGDDLTSALIAAREADPDALSEEELVGTLLLMLSAG

QETTLSLITNAVRALLTHPDQRALATGGDASVWADVVEETLRWDAPIGNF

PFRYPLEDVEIAGVRIPKGEAIMAPYSAVGRDRRQHGSDADRFDIAREQR

RHLAFSHGPHFCLGAPLARLEAGLAIPAVFRRYPDLSLAVDPGALVPVPS

LFSNSSSTLPVRLGN

>CYP156C(2585311798)Streptomyces mirabilis YR139

VTSAPPPGCPHHQPLCGPDFAADPAAVYATLRAHGSTAPVELAPGVRATL

VTGYEAALHVLRSPETCSKDPRRRRDLADGTVAPDSPVVPMMMYRPNALF

TDGEEHRRLRGAITDGLARIEPNTLRGCVERSADTLTDLIANSLRLLLSD

DRFAGSLGGGSLPVEDALDEVRWTDPPMANYAVHYPVRDVVYEGAPATRS

C

>CYP154A(2585311800)Streptomyces mirabilis YR139

MAQQDSIVIDPSGRDIHGEAARIRARGPVTRVELPGGVEAWAVSSADLLR

RLLTDPRVSKDPRRHWPLWINGEISPEWPLFTWVAVQNMFTAYGGEHKRL

RALVSKAFTARRTAALQPRIEEITKELLDRLDEAGRRGEAVDLREEFCYP

LPIRVISELFGLPEDKGAELRAVVDGVFNTAATPEEVTDIYARMYAVLGE

LVALKRKSPGDDLTSALIAARDDEGDTRLSEQELLDTLMLMISAGHETTV

NLIDNAAHALLTHPEQLAHVRAGRAGWDDVVEETLRVRAPVASLPLRYAV

EDLVMGELGGPDGVVIARGEAILAAYAAAGRNPEEYGADADRFDVTRLGK

EHLAFGHGVHFCLGAPLGRMEARIALPALFERFPALELAVPDEELTPVAS

FISNGHRSLPVRLNSDG

>CYP105B74(2585312326)Streptomyces mirabilis YR139

MTESVAGTDVAEELPEYPSPRSGECPFAPPPGLLKLHDTGKALVRTKTWD

GTTPWVVTTHAAQRQLLTDPRLSANIGAPGYPHTTEAMKAHAAEIQPSIN

NTDGAEHTRWRRMLTSSFTRHRMEKIRPEIQRITDDLIDEMLAGPNPTDL

NEALSLPLPSLMICALLGVPYEDHEFFQEHAGVTNARFKTPEKAAESTAT

VRRYISGLIEAKMDDPGEDVLSDLGARVKEGDLSMEEAAPLGHILLVAGH

DTSANMITLGTALLLQNPGQLTALREHSDDPKFVTSTVEELLRYLTIPHL

LARRAVVEDIEIDGETIRAGEGVIASLPAANWDPQAFPEPEKLDLTRAAA

HHHAFGWGPHQCVGQQLARIELHVVFSTLFRRVPTLRLAVDVSELKFKED

SQAYGIYNLPVTW

>CYP1027G3(2585312335)Streptomyces mirabilis YR139

MSTTLEPDGSYQGVPAYHFDNRLDGPVLSHQERWDVMAGTHSGFRSTVAR

GYWVVTEGEAVQKALQDWQTFSNTSVTALDPDPRFLWIPEMLDPPQHTAW

RRLLGSAFSPKTVAAREPEMRQVASGLIDELKDSGGTDVLDAFARRFPTL

IFMRLMGLPEEDLARFLGWIHDLLHLSYDEDPDGNRQLSAMHAVSDYFEE

QIALRREAPRDDLLSRAMTWTIDGEPIGDRDMHAFCVLLFQAGFDTVPIS

IGWALYHFATHPEQRAEIVADPSLIPTAVEEILRIYSFVVPARKATRDVE

IGGCPVSAGEMVMLPLAVSNRDPERFHHPLEVDLRRKTTNHIAFGSGPHR

CLGSHLARLELNVAIEEWHRRIPDYRVTPGQHLEAHGNMYGIKQLRLAW

>CYP107P(2585312875)Streptomyces mirabilis YR139

MAAFDPWDPAFVADPYPAYAELRAQGRVHYYEPSDQWLVPHHADVSALLR

DRRLGRTYQHRFTHEDFGRTAPPAEHEPFHVLNDHGMLDLEPPDHTRIRR

LVSKAFTPRTVEQLKPYVEGLAGELVAGLMEAGGGDLLTDVAEPLPVAVI

AEMLGIPESDRAQLRPWSADICGMYELSPSEETATRAVRASVEFSDYLRE

LIAARREEPGEDLISGLIAAYDEGDRLTEQEMISTCVLLLNAGHEATVNA

TVNGWWALFRNPDQLASLRADHSLIPSAVEELMRYDTPLQLFERWVLDEI

EIDGTVIPRGAEIAMLFGAANHDPAVFAAPEKLDLTRKDNPHISFSAGIH

YCIGAPLARIELAASMTALLEKAPTLTLAAEPKRKPNFVIRGLEGLQVEP

A

>CYP125A(2585313280)Streptomyces mirabilis YR139

MPCPALPDGFDFTDPDLLHRRVPLPEFAELRAAEPVRWIPQPLNLAGFQD

EGYWAVTRHADVRYVSTHPELFSSTLNTAIIRFNDHIERDAIDAQRLILL

NMDPPEHTRVRQIVQRGFTPRAIRALEERLRDRAQAICAGARARSGPFDF

VTEVACELPLQAIAELIGIPQDDRTKIFEWSNKMIAYDDPEYAITEEVGA

QSAAELIAYAMNMAAERKQCPAHDIVSTLVAAEDEGNLSSDEFGFFVLML

SVAGNETTRNAITHGMHAFLTHPEQWELYKRERPSTAAEEIVRWATPVNS

FQRTATQDIELGGKLIRKGDRVGIFYASANHDPEVFENPDVFDVMRDPNP

HLGFGGGGPHYCLGKSLAVLEIDLIFNAIADAMPGLKLTGDPRRLRSAWI

NGVKELQVTTG

>CYP1859A2(2585313356)Streptomyces mirabilis YR139

MDDKTQTHNPALPEFWLQKDIEAAFAEMRREKAVHWHEEPPSDWFPEGGR

GFWSVVRFEDVVEVSRDQETFTSDHGTEIVDMTPEMVHIFGGMLNMSGAA

HAHHRAIVNRVLTPRTVEALSDTIRAHARRCIDRVAAQDGCDFMQDMVGD

FPAQIICELLGVPVDDRARLVALTGTALSAYGTAEAYNAMLEIIAYAESL

VALAREQVSDSASFLRKLLTAQVDGQHMSDHEIAVFFALLVTAGIETTAT

SIGQGMYALSLHPEQRRRWQSDFQQLAPRAVEEIVRWVTPVRYFRRTATR

DTELAGQPIAKGDKVVMWYTSANRDESVIERAGELDFARTEERHVAFGGG

GPHFCLGAVLARKEMTIFFEELFARLPDIEVNGEPERLHSNFVNGLAALP

VRYTPA

>CYP157A(2585313506)Streptomyces mirabilis YR139

VTAPVPLSGPRFQTEPARLYREMRREHGAVSPVVLDGDVPAWLVLGYREL

HQVTGDPVLFSRDSDLWNQWDRIPDDWPLLPMIGRKQPSILYTVGERHRE

RAAMISDALEAVDPSELRAYAEKFADELIDAICAEGETDIVTDYAMLLPV

RVLARLFGFSDEQGPGLVTALNDMIDGRERAIAGQTHLATSMAQLLADRK

AEPADDVVSRMLANKSGFGDEEIAQDLMVMMAAGHQPTADWIGNSLRLML

TDERFAASLFGGRNSVAEAMNEVLWEDTPTQNVAGRWASRDTQLGGRRIR

AGDLLLLGLQGANSDPQVRTDGSSLTGGNNAHFSFGHGEHRCPFPAQEVA

EVIARTGIEVVLDRLPDIDLAVPAESLTRRPSPWLRGLAQLPVRFTPVPA

L

>CYP154C(2585313507)Streptomyces mirabilis YR139

MTSGIEESRIVLDPFVGDLDGESARLREAGPLAAVELPGGVPVWAVTHHA

EAKQLLTDPRLVKDINVWGAWQRGEIPPDWPLIGLANPGRSMLTVDGADH

RRMRTLVAQALTPRRVEQMRERITKLTDGLLDTLEGDVVDLKADFAYPLP

MYVIADLMGIAEDKLPRLKELFEKFFSTQTPPAEVIATLTELAGIMAETV

AAKRAEPGDDLTSALILASEDGDHLTDEEIVSTLQLMVAAGHETTISLIV

NAVVNLSAHPVQRALVLAGEADWSSVIEETLRHSTPTSHVLIRFATEDVQ

VGDKVLPAGDALIVSYAAIGRDEQAHGPTAGDFDITRTSENRHISFGHGP

HVCPGAALSRLEAGVALPALYERFPSLDLAVPVTELRNKPVVTQNDLFEL

PVRLTPLG

>CYP188A(2585313794)Streptomyces mirabilis YR139

MTTEDAAAAVDDDGRKQHQVHFDRHTPDYRHHFEEITHELHGTCPIAWTQ

THGGHWVASGNREVFELARSAEFLSNDHDVKGERRGYKGISIPPPPRARE

AQGGFLEMDPPDQRHYRQTLNPYLSPAAIQRWIPFVDEVVRAGLDEKIES

GSIDFVDDLANIVPAVLTLAMMGIPLEHWTIYNEPAHASVYTPPNSPDMP

RVLELSRKMGQHLMTQLMEIRNKPRPGLVDALVRADISGRNPDDGELLGV

LALLIGGGFDTTTALTAHALEWLSQNPEERARLSRDRATLLDSATEEFLR

FYTPAPGDGRTFSADCEIAGTEFKEGERLWLSWAMANRDPSVFPDPDRID

MERKGNRHSSFGLGIHRCIGSNVARTVFKRMMTAVLDRMPDYRCDPEGAV

HYETIGVIQGMRHLPATFTPGERLGAGLDETLAALQKVCDEQRLAEPVTV

RTSKARIGT

>CYP150A(2585313799)Streptomyces mirabilis YR139

VHTVKDFEAIDFFQDDEVVADPYPYFEALRGQCPVHRESHHDVMMVTGYD

EAVQVFNDAETFSSCVSVTGPFPGFPVPLEGDDVSDLIEQYRDQLPMSDQ

LPTLDPPVHTDHRGLLMRLITPRRLKENEALMWHLADARLDGYLAGGEGD

FISGFAGPFTLAVIADLLGVPDEDRDAFVEGLQHRPHSDAGIGSTDGEVA

YSPLEYLYGQFSAYVEERRGEPRDDVITGLATATFADGSTPDVADVARVA

TNLFAAGQETTVRLLGNALKLIAEDPDLQHLLRTERDRIPNFIEETLRTE

SPIKGDFRLSRVPATVGGVDIPAGTTLMVLNGAANRDPRHFEDPETFDPA

RANARHHLAFGRGVHTCPGAPLARAEARVAIERILDRTADITISERVHGP

ADARTYRYLPTYILRGLTHLNLEFTPAEESAR

>CYP154U(2585313868)Streptomyces mirabilis YR139

VTVTDRIALDPFGADIPAESARLRALGPIVPVELPGGIPAWAPTGYDTLK

ELILDPQVSKDPRLHWKLWPEIGEHPSWGWILGWVGVVNMLSTYGTDHAR

LRRLVAPSFTHRRTEAMRARVEAITTELLDALEAADGDVADIRERLAHPL

PMLIICELFGVPDELRDDTARLIAAIMDTSDPTPEHAAFVQQQIGTVLPA

LIAHRSEHPGDDLTTELIRVRDEDGDRLSDEELLYTLLLVIGAGFETTVN

LIGNAVVALLRRPEQLAAVRSGEITWDAVVDETLRVHPSIASLPLRFAVS

DLKVGDITVPAGDAIITTYAAAGVDPAHYGPDADVFDAARGADDHMSFGI

GVHRCIGAPLARAEALTALPAFFERFPDVRLAVDADELRQVPSFIAFGWR

EVPVRLRG

>CYP107X(2585314260)Streptomyces mirabilis YR139

MDLADGLLDHPYDVYRRLRDSAPVHRIAGPDGTPAWLVTRYDDVRAALSD

PRLSLDKRHATAGTYKGFSLPPALDANLLNMDPPDQTRIRRLVGRAFTPR

RIEQLRTPIRRTADQLLDALGPDGDTDLIASYAAPLPITVICDLLGVPDS

HRRDFRVWTDALVAPAPGARPEAGKEAVVAMLGFFTGLLADKRKEPADDL

LSDLIAVRDEGDRLSEDELMSLAFLILFAGYENTVQLIGNAVLALLRHPE

QLAALREDPTRLPAAVEEFLRYEGPALLAIRRFPVEDVTIGGVTVPAGET

VWVSLSAANRDPGRFPDPDRLDLGRDTSGHLALGHGIHYCLGAPLARAET

EIALSALLERFPHLALGEGELRWRRSLRARGLVALPVTYAKSSV

>CYP105B(2585314731)Streptomyces mirabilis YR139

MAEAETELAAPDAEIFDYPGARDSACPFAPPAGLRALNDKAPVSRGRIWD

GSTPWLVTGHAAQRVILSDPRVSSNDKQPGFPHPNQAMAENSPHHPLTIF

NADGADHTRIRRMMTAPFTRNRMEKLRPEIQRFTDELIDKMLEGPKPADL

NTALSLPLPSLMICALLGVPYEDHEFFQEHASLATRSDKTAEQDRAANEA

LGGYLAGLLQVKMEEPTDDVISDFAGRIKAGEVTLPEAVMLCMILLIAGH

ETSASMITLGTALLLENPEQLTLLRETDDPKVVANAVEEILRYLTIPALG

QRRIAGEDIEIEGVTIKAGEGIVVPLPAGNWDPEAFPEPEKFDISREARH

HHAFAWGIHQCLGQQLARIELQVVYGTLYRRVPTLRLAVGRDELRFRRDD

ALAYGIHELPVTW

>CYP147F(2585314755)Streptomyces mirabilis YR139

MTQSPLHQILDHANRANPYPIYEELRKTPVHHEDDGPYVISTYYEIRSLL

HDPRISSDARNLASTTGDPLAEPTQEESALPPGFLRLDPPEHDRLRRMTN

RPFGPPHSPHRVDGMRGELHGIVSGLIDGIGDPGRIDLVEQFSYPFPVTV

ICRLLGVPREDEARFHTWADTIAASLDPDPNADPAERGKGSHEARMQLGM

YLAGLIEERRKKPGEDMLSQLAAAKGEDGPMTTMELLSTAALLLIAGHET

TVNLITNGMLTLLRNPDVLQRLRADARLAVPIVEELLRFEPPVQLVPQRT

TLADVEVRGVTIPKGASLWLVLASGNRDPQRFEDPDHFDPDRRDIQHLGL

GSGIHSCFGAPLARLEAQNALAELARRLENPRLLEDPPPYRQNAVLRGPR

HLHISCDGIRP

>CYP107L(2585314832)Streptomyces mirabilis YR139

VTEVIDLGQFGDGFRSDPHPVYAMLREQGPVHRVSLPPLDAQNEVWLVVG

YEEARSALADPRLSKDGSKIGMTFLDEDLIGRYLLVADPPQHTRLRGLIT

RAFTMRRVEELRPRIQEITDELLDAMLPRGRADLMESFAYPLPITVISEL

LGVPEIDRTEFRTLSTEAVAPTSAESEYDAFVRLADYLRDLIEDKRCAGP

GGDLLSDLIRTTAEDGDRLSPQELRGMAFILLVAGHETTVNLIGTGVHTL

LTHPDQLAALRADMTLIDRAVEEMLRYEGPVENATFRYAAEPLEIGGTRI

EKGEPVMVGLTAADRDEAHCPDPDRFDIRRDTRGHLAFGHGIHYCLGAPL

ARLEARTAIRTLLERAPALALDGPPGEWLPGMLMRGVRSLPVRW

>CYP102B(2585315080)Streptomyces mirabilis YR139

MAETTTATAPPKGFRDAEQGWPRLDRIPHPPRRVPLLGDVLGADRRTPVQ

DSLRYARQLGPIFRRKAFGKEFVFVWGCDLVADMADESRFAKHVGLGVAN

LRPVAGDGLFTAYNHESNWQLAHDVLAPGFSREAMEGYHPMMLAVAERLT

DHWDRELAAGRAVDVPGDMTKLTLETIARTGFGHDFASFERARPHPFVTA

MVGTLSYAQRLNTVPSPLAPLLLRTATRRNEADIAYLNRTVDDLVRTRRT

ATGDGDLLDRMLETSHPDTGERLSPENVRRQVITFLIAGHETTSGALSFA

LHYLSSHPDIAARARAEVDRVWGDTEVPGYDQVAKLRYVRRVLDESLRLW

PTAPAYAREATQDTVLAGVHPMRRGAWALVLTPMLHRDPEVWGADAERFE

PDRFDAKAVRSRAPHTFKPFGTGARACIGRQFALHEATLVLGLLLRRYEL

RPDPAYRLRVAERLTLMPEGLRLRLERRTAAAPAPVAVPDDSPSELRCPV

QGAGE

>CYP107BX(2585315108)Streptomyces mirabilis YR139

MTADSTSRPLRPMHLMTYPEPGPPRLATLATGTPVWLVTRYAEVRQVLMD

PRFDRRSLHAEGAPPLLVVPNILDDPNGLLNQDGPGHQRLRGTVQRAFTP

RAIARWRPWVASVVESLLDAFAGQPMPADIIEGFTRPLPVSVMCRLMGLD

HVDRERIRHWADHALSGGAHSAEQVVAAMTEFGAFAVELIGERRAKPGDD

LVSGLVAAGDTLGIEDRQLVSLVCGLVVAGHETTMSALGNVVVYLLTDGR

AAWPPLAEDEEAAATAAEQLLRTVPLSEGRVLPGLIRRAVEDVEIGGVTI

PAGSVVAVQTNSASRDPDVFPPGPPDVTAQLPAPSVIFGAGPHHCLGSWL

ARLELELALHRLAVRFPHLRAEFTPETIEWRVGQMTRSPLRLPVSW

>CYP157K(2585315123)Streptomyces mirabilis YR139

MNDQTLNGPDAPRGCPVAHGSADAVTRLYGPEAATDPHGIYERLRKEHGS

VAPVLLEGDVPAWLVLGYRDNRRVLDNPRQFTRDARIWRDWREGRIDNTS

PLIPMVGWRPDCVSQDGEPHRRLRGAVTDNLLAAAGRGIRRHATHFANKQ

IDAFADTGRADLVADYAEYLPMLVLTRLLGLAEAEGRRLVESCAQVLKGG

EDALMHNDIIIQSLGELTARKRAEPGSDFTTGLLGHPARLDDEEVVSHLR

LVLIAAHTTTSNLLARVLQLVLTDAARLSGLVSGQLNISSVVEEVMWDTP

PLAVLPGRFAAADLELGGHHIQEGELLVLGLAAGNLDPEVRPDSAVAVQG

NQSHLAFSAGPHECPGQNIGQSIIEIGVDVLLHRLPGLRLVVPSEELSST

ASTWESRLDSLPVEFSV

>CYP105D(2585315134)Streptomyces mirabilis YR139

MTELTEPTAPTEPVAFPQDRTCPYHPPTGYDPLRAERPLARITLYDGRPV

WLVTGHAAARALLADQRLSTDRTQPGFPATTARFAAVRNRTTALLGVDDP

KHRTQRRMMIPGFTLRRAVELRPSIQRIVDERLDAMIAQGPPAELVSAFA

LPVPSMVICALLGVPYADHEFFEAQSRRLLRGPAPEDTQDARDQLEAYLG

ESIDRKRKEPGDGLLDDLVRDQLSAGQLDRAELISLAVILLVAGHETTAN

MISLGTFTLLRHPERLAELREDPALLPDAVEELMRMLSIADGLMRMATED

IELAGTTIRAGDGVIFSTSVINRDQDVYTAPDSLDWHRPTRHHVAFGFGI

HQCLGQNLARAELEIALHSLFDRLPTLRLAAPADEIPFKPGDTIQGMLEL

PVTW

>CYP154D(2585315239)Streptomyces mirabilis YR139

LDTSPDAPHRMDPSGGCPHADNARLLGRGAVAPVLLPGDIEGMAVLGHEA

LKEFLAHSEVAKNARHFTALREGRIAKGWPLMTFATVQGMTTADGDDHRR

LRSLASRAFTPRRVEELRPRIEELTASLLDDLGRAAELGGGVADLRRYFA

LPLPMGVICELLGVDAEFRDRLRHLSHQVVATDIGPEEAVAANRDLVAVL

ATIAAARAKQPGDDLTSALIAARDEDGDRLSEEELIGTLVLMIIAGHETT

LNLITNAVRALCGHRDQLDLVRKGEASWGDVVEETLRWDAPVSYFPFRYA

VRDLTVDGTVVPEGTPVLAGYSAAGRDPAAHGPDADRFDVTRPGRPGAVR

NLSLGHGAHYCLGAPLARMEATIALERLFARFPGLDLAVPEDGLSRNSSF

VGNSVRTLPVSLR

>CYP1457B(2585315272)Streptomyces mirabilis YR139

MESSATRQGYGEHARTPRGIVPRVRDPLALLTAEFDGVHDVWRSAAGTVC

VAGPETARAVLGNRHAAVAETSDFYRTRHGVFGPRSAQIRIGRSARALMR

RHLDARRTELPRLVGERLAPRSVWPDAGNLLVLQHLRDVLLHREASAQLH

VTVEEIVTRAVLAGARRRHSPPARLLFRRRALSALHGEIRARQRQWSGSA

EPRDLLDVVVDGSGPGADPRELAEIYLSFLFAAVGSVGFALGWSVYLLGT

HPHCLAMEPSWTVREALRLWPVAWLFARTPSRSQELGGTTVTPRDQLAVC

TYLVHRHPGHWERPDEFVPQRWAAAAPDAAYLPFGHGPHTCAGATVTMRL

LEDLVGLITRDWRLSVTQDGDGPQVGPALAPPRFTAVLSDHSGCPGRR

>CYP1453B(2585315273)Streptomyces mirabilis YR139

MTTPQHPEDFLRFMRMRSATEDGVFWVDDKRLGVFEPEAARRVSATNWHR

FVMHDRLIDMLLRRRSPEVRWSQVRSAWLTQLHALATAEHHGRLIDRMER

IIDERLGEDVDLVMLTQDVALRSMLPVALSGLASGEADLVRRDLEMKLLR

LISPEPGGTWHHLRFIVVQMRSGLVVRRVLRQRARGTRKREPDLADPFVD

LLPELGMDRALDVVTAVLTALGGPPGTAAASLLYEFARHPEWQRRLADEL

CAVDPVEFRTAPTSAAPVTHRFVKEVLRLWSPPLLLVRRNHFPFDFGKTR

LEPGDWYLLSPHLIHRDERVWKQPDVFDPDRFLPGAPHGPADRTCYVPFG

WAPKKCVGANIGTVQLMALCHLLCTRYRLTVDRPEDLTMALRFAPVPENF

HGRLALR

>CYP157C(2585315600)Streptomyces mirabilis YR139

VTPEHHSMTGTDDLLGPPPGCPAHGLGPGGLHRLYGPEAADLGALYEKLR

AEHGTVAPVLLHDDVPMWVVLGHTENLHMVRTPSQFCRDSRMWTPLLEGM

VKPDHPLMPHIAWQPIASHAEGDEHLRLRGAVTGAMSTIDHRGIRRHINR

YTQILVNGFCETGRAELVGQFAEHLPMAVLCEILGMPDEYNERMVEAARD

ALKGTETAIASHQYVMDALGRLTTRRRARPEEDFTSHLITHPAGLTDDEV

REHLRLVLFAAYEATTNLLANALRMVLTDPRFRAQLNGGQMTVPEAVEQS

LWDEPPFSTVFAYFAKQDTELGGQRIRKGDGLLFAPAPGNVDPRVRPDLK

ANMMGNRSHLAFGGGPHECPGQDIGRAIADTGVDALLMRLPDIELDCDED

ELSWTASIASRHLVELPSQFAPKPQQDVSQKPGHGPVPQQRAAWHIGTPP

PPPQPAAPVAPEPRPAAEPQPAPVPEPVRPLGAWGRFLRWWRGY

>CYP179A(2585316548)Streptomyces mirabilis YR139

VDREPHSGLDELQRDPYPHYERARRADGLTFVAELDAWLVARDADVREVL

RRPEDFSSANSLQPDVLPSPAAFAVLGGGFGGRPVVVTADGSLHQRLRAP

VVRGLSPARVAAVLPYAAERVAALVDTFAKDGHVELMSAYAMRLPGEVIG

RIVGLDPADVPAVVHGGHRAEELLFRPLAEDEQIAAAEDVVAMQRILERY

VRERHAHPREDLCTELITSVTDGDLTLDHRHEVVAHLQNLLIAGHLTTTA

LIGTTVLHLLGHPRQWELLCAEPERIPAAIEEAARYDTALQGFRRVTTRP

VTLAGTELPAGAALFVAFGAANRDGLRHPRPDEFDITRTPGRHLAFGFGV

HACPGAQLAREQLRLTLEELTRRLPGLRRAEDQPVRMRPTMIHRSPYALH

LTW

>CYP1199A(2585316785)Streptomyces mirabilis YR139

MPLSSPPRARTTVFAPRLAALLRDHLGQDVFRLEPDTVGVAGPEVTDRIL

AARRATDTERPTFKPLHGRSISRNEGASVIRTVGADVREALKRPLPEDVD

LTGPWPLTGHLLLRDLILGQDPYRLRMLMSRNLELTTKLTWSVIAVGAAL

PGWPRPGERLTGLAARAAEATGYHDRRYAMGIYRRAAAPVCFTISTLVAN

ALWLGAPFDDATPNRDIIHESLRLLPPSWNILRNASPEYPEIDGRIGAQD

DILLLPLLSHRDPALWEAPDEFRPERWNDLDPDTAPGYLPFGHSSERCWG

RHMVMPLAELLLDRVRAAGLVVDPGQRVGKVPLLGLLGVEEVRLTRPV

>CYP1417A(2585317103)Streptomyces mirabilis YR139

VTASPATPAPVPTAPGGLPLIGHAHHLARTPLPFITSLRDHGSVVRICLG

PTPAYVVTDPVLTRTVLVTEAAHFAKGGKIFDVLRGVFGDGLATVADGDT

HLRNRRLMQPMFNKAHIATRGNAMIDQVRSMVGAWEEDQLRNVFADMNDV

TLAAFLVALFGMDLPANLQREFTVLMPMIMKGAIRQTILPPWVTRLPLPA

NRAHTHRVARLRALIDRAIDHHSGRSAGTPPATGHDPAEAAGCPHKAAAD

NEQAGLFETLLTAGDQFTRRQLQDEAITLMNAAIETTATTLAWTLYELTQ

HPEIEQRLRAELAAVCTGRPLRQEDLDRLPYARQVLQEAVRKYGPAWMVT

RTATRDIDLGGHHIPQGADILWSPYLHQNDPAHFPEPATFDPDRWPPDRV

PTTRDSLFAFGDGRRKCIGENFAWTEMQIILATILQTWPRHRLVSRPPRP

QTAITVKPDKLAIAFGTS

>CYP107BU(2585317433)Streptomyces mirabilis YR139

MSVVGSFPLGAATTLAELARDPHPRLALLRAHEPVSWLPELDGWLVTRRD

LALSVMRDAETFTVDDPRFSTAQVVGPSMLSLDGDQHARHREPFTAPFRP

REVRDGFASFIERETDRLVTALEPAGTGELRRAFAGPLAVSVVTEALGLV

GTTAATVLAWYDSIVRAVSDITAGHEAGPAGAAAYAQLRAAVEATVTDRG

GASLLGSAVGPLTLPEVASNAAVLMFGGIETTEAMITNALLHLLQRPDQL

ALVRADFDLLDGAIEESLRLEPGAAVVDRYATRDTTLGPATIRRGELVTV

SLSGANRDPAVFPDPDRFDVRRENARLQLAFAHGPHYCLAAHLARLETRI

ALQCLLERLPALRLDPDHPATPHGLVFRKPPTLHVLWDRANASS

>CYP102G(2585318040)Streptomyces mirabilis YR139

MKEAKELGPLFKLRIFGNEINFASGLDLVTELADDSRFRKNVHPDLVILR

EIGGDGLFTAFNDEPNWRKAHDILMPAFSLGAMRGYHSTMLQVARELIGK

WDRAAGAEPVDVAADMTSLTFDTIGLCAFGYDFESFSREEPHPFVTALSR

ALGFAQAKGESIPGTELFKWKRAERFQADVTLMKDLVDDVIRQRRASGDQ

STDDLLGRMLHVHDAVTGEPLDDVNIRYQAITFLIAGLQVSHAGLVIDHA

DQRLREVCVSAV

>CYP183X(2585318351)Streptomyces mirabilis YR139

MSGVVPQRERVWTTGTAPGIFPFLGHAITLFRRPLAFLTSLPAHGDLVEI

RLGPQRAWMACHPELVHQILMDPRTYDKGGPLYDKLRLLMGDGLVTCGQE

AHRRQRRLVQPSFRPSRIADYAETMGEEAELECRQWQAGEFDLSGALMAL

TTRVTSRVLLSDSLDASTAAEVRDCLAVIVRGLFVRMVAPIEVLFRIPTP

ANRRYRRALNRLHTIIDSAIEERRPGAPRNDLLGTLLGTGGGNGGTSPLT

EQEVHDQLITLLLTGVEAPAMCLASAFRLLAAHPEVERRVHREVDAVLAG

RRRPRPDDLPHLVYTRCVVDETLRMYPPGWLFTRITTTDTNLAGRPLPRG

TTVLYSPYLLHHDPASFPDPDRFVPERWLPGQEATVPSGAMLPFAAGNRK

CMGDTFATAEATLAIAAIASHWRLRSLPGSAEQPVPAVTLGHRSLVMICE

PRPRTPVGEPSREGSVGPPVQDARQARQNSRTAGDEGVCDA

>CYP163H(2585318528)Streptomyces mirabilis YR139

MPNKGIFADDLNSVDLTDPKTFLHTDMEDFWRRVRADQPVYLHPATDRGS

EFWVVSRHSDVMAVLQDPERFSSQPGNMMSSLHKPGGDPAAGKILALTDA

PRHNAMRTILLKAFSPRIRKGVTDKLQQRVDHVLGHAVGTGTFDFAKEVA

EVVPMGTICDLLGFPSTDHKYVLDLSRETVSADDEGQTEEDVWLSRNELL

IYCQEIIEARRESPQDDLISAMATCRINGEPMTDDEVVVNCYGFILAGDH

TSRLAMVGALLAFSQHPDQWLALKEGRVSIASAVEEIVRWTTPAMHIGRT

ATAEVTIGGQTIREGDHVILWNTSANRDESVFSDPHSFDLARTPNKHLGF

GFGPHFCFGSYLGRAEITAVLKTLISRVDRIELMGDPKALYSTFLRGYSS

LPVSLIPSRFTAK

>CYP170A(2585318623)Streptomyces mirabilis YR139

MTVESVKPEAEARPASELRVPPLAGGGVPLLGHGWKMARDPLAFMAQLRD

HGDVVRLKLGPKTVYAVTAPHLTGAMALSPDYIIAGPLWESLEGLLGKHG

VATANGPTHRRQRRTIQPAFRLDAIPAYGPIMEEEAHALTERWRRGETID

CTSESFRVAVRIAARCLLRGDFMDERAERLCVALATVFRGMYRRMVVPAG

PLYRLPLPANLEFNRALADLHLLVDEIVAERRASGQKPDDLLTALLEAKD

ENGEPIGEQEIHDQVVAILTPGSETVASTIMWLLQVLTEHPEQADKVRAE

VESVTGGRPVAFEDVRQLTHTNNVVVEAMRLHPAVWILTRRAVRDTDLGG

YRIPAGADIVYSPYAIQRDARSYARNLDFDPDRWLPERVKDVPKYAMSPF

SVGNRKCPSDHFSMAQLSLITAAMATAYRFEQVAGSSDTTRVGITLRPHR

LLLRPVPR

>CYP107U(2585318837)Streptomyces mirabilis YR139

VTDQPTPTSTAPELFTWEFATDPYPAYAWLREHAPVHRTKLPSGVDAWLV

TRYADAKQALADPRLSKNPAHHDEPAHAKGKTGIPGERKAELMTHLLNID

PPDHTRLRRLVSKAFTPRRVAEFAPRVQELTDHLIDQFAANGSADLIHEF

AFPLPIYAICDLLGVPPEDQDDFRDWAGMMIRHGGGPRGGVARSVKKMRG

YLAELIHRKREALPDTPDPLPGEDLISGLIRASDHGEHLTENEAAAMAFI

LLFAGFETTVNLIGNGTYALLTHPEQRTRLEKSLAAGERELLETGVEELL

RYDGPVELATWRFATRPLSIGGQDIAPGDPVLVVLAAADRDPERFADPDA

LDLGRRDNQHLGYGHGIHYCLGAPLARLEGQTALATLLTRLPDLQLAVDP

ADLRWRGGLIMRGLRTLPVEFAPVR

>CYP182A(2585319304)Streptomyces mirabilis YR139

MEAHSRTPAPAEHPALGSLSVEPLLTSAFDADPGAVYARLRRTYGPVAPV

GLMGVPAWLVLDYREVLEVLRNESLWRRDIRYWRARAEGRLPRDWPLLAG

YEVRQTMFMDEEEHRAARHTLHTALQPFQEARSLQGWELRSTVASYADEL

VAMLAAESGSVGFADLSAQYTRPLLLMVTTKLFGCPVELGDELVMDLWRM

LDGGPDAGPATARASAAMTRLAAHRRARPGDDLTSYMLLADPGLSDEQLG

RELFMNAVYLNDITGNMVLNTLLEVLRGNATVRRSLSAGQLGETVNRAAL

ANPPVANMCFRFAARDVPLGNFWIRAGDVVSPSAAAAHQDLLAIGSSQLV

GSAVSTRAHLGWGAGQHQCPAAARELGGVIVATAVGRIFDHFSQAELTLP

PDQLPWRSGPVVRGLRLLPVRYEMNRPSARPPVPRPRQDHHPTGTTHASD

GQAKRLLSALRRLMFGGKKDGRDG

>CYP1238A4(2585319916)Streptomyces mirabilis YR139

VLCLTGVPVTQTAGIPARPPGPPSLRTAARDPYPLYRTLRTHFPLVYDEP

YGAWLVSRYDDVRAALADPRLVAVPSGGRPTGSDTVALLAPALRGPALAA

LTAAVQRSAYVLARRLSARAEADLVAEFCDWLPTATAVAALGLPYEETAR

VHRWCRTGLDHLGGDDPELAAFLRPLTARRRAHPGSDLLSVLCTARAGGR

PLPDETVTGLVGALLGAAGETTARALASFLANLLDHPRQLDVIRARPELT

ADAWAESLRRDPPLHVVLRRAAEPVGRIPAGATVACLLGAAGRDPARFTD

PDRYDAFRTDRGPLAYGTGSRTCPGALLARLTAEHGLHALLTALPALRRA

PGARPAPEDPIRRAPGALRVRTR

>CYP180A(2585319936)Streptomyces mirabilis YR139

VTVHEAPPVPDVFDPRRYADGVPYGAYRVLRDHHPVAWQDEPEVLGWPAG

PGFWAVTRHADVVRVLKDSATYSSYLGATQIRDPERDDLPFIRRMMLNQD

PPGHGRLRRTVSRAFTPGRIERFTQTARARARELFSRAAESARAADGTVD

LVAAVTDDYALLNLTDLLGVPEGERALLLNWTRRVIGYQDPDEAGSPVRD

ADGRPVNPRSPAMLGDMFAYARQLAAHKRRYPADDVMTTLAHEAELADAE

LEMFFFLLTVAGNDTVRSAAPGGLLALAEHPQSYERLVAGKLELAPAVDE

LLRWYPPVLSFRRTAARDTELAGRRIRAGDKVVVLHASANRDERVFADPD

RLDLSRAPNPHVSFGDGPHVCLGAHFARLQLRVLYAEALRAFPTAPRVAG

PPERLVSNFINGLKSLPMRVT

>CYP1232C1(2585320586)Streptomyces mirabilis YR139

MSTLRTAAPGAPVVELDLAELRRDPYPAYTELRRTAPLAWVPSVGRHLLT

RYEDIVHAEKLPEVFSSREDGSLLLRTVGPNMLREDDPEHRRLRAAAEPP

TRPRQVRDLWAGSFERTAHELLDRIAGRGEADLIADFAEPLVATNLALVL

GLRNASADDIADWSRAMMAGNGNYADDPDIWLRAELATRAIEEAVAERAE

AVRREPDGSVISSMVHAAEPVDLAEIQNNIKVIIGGGVNEPRDVFGVGAQ

ALLQRPEALEKVLRDPAAWKRVFEETARWVSPIGMYPRQLTEDHEIAGVT

LPAGSRVALVIASGNRDESVFERADEFDLDRPHRPHLAFGGGPHFCMGAW

VARHEVSNLAWPLVFSRLKGLRLVEGATASDSGSAVGRMDGWVFRGLTSL

NVTWQAP

>CYP1232G2(2585320606)Streptomyces mirabilis YR139

VATTVSQPAEHIRLAELWRDPYPAYRYLREHDPVAWVPEAGRYLVTRFED

IMHVERHPETYLSAENPSLMTRSIGETLLRQDGAAHQRLRRAAEAPLRPR

IVKEHWLPAFRRNAEDVLGAVVGRGECDLFREVAGPLAARNLADLLGLRG

VGDADMQEWSQAIIDAGGNYGEDPDIWARCARAVADLDVAMDEVLPVLRR

DPDHSVISAMLHAGDPLTEDEIRTNVRVFLGGGLNEPRDSMAAAAYALLT

HDDQREDVEAEPALWRRVFEEAVRWVSPIGMYPRRVAERTELGGRVLEPG

DKLGVVIASGNRDERVFDRPDAFDIHRAETSHVGFGGGPHFCLGTWVSRA

AVGQIGLPLLFRTLSGLRLTETEVPFGGWVFRGPLRLTAAWDT

>CYP247A(2585321192)Streptomyces mirabilis YR139

VRLTPGAGRDIDLDGVDLFDLDLYTSGDPHPIWDVMRAKAPLHRQVLPDG

REFWSVTRYEDVRRVLSDHKEFTSERGTVPTHLGEDDAAAGVLMTSTDPP

RHTEVRRPLGSKLTARAVKSWEDRIRGTIVRFLEPALDGEVFDLAEKALL

LPAMVTGPLLGIPEKDWEELVQLTAMVTAPSDPHFLVGNEAATLAISHHE

LVTYVREWVRTRRATGGEDDSLLQHLMSVRPGGAPLTDEEIALDGYSILL

GANVTTPHTVSGTVQALIERPEQFEKAQADPSLVPNLVEEGLRWTSAACN

FMRYAVNDTRIGGGTIPARGAVVAWIGSANRDESQFPDPHQFDITRSNAK

RQAAFGFGPHFCIGAPLARMTLRVFFEELLQRFGSIDLAGEPQHLRSYFI

AGMTHLPIVGQKRKTP

>CYP154B(2585321658)Streptomyces mirabilis YR139

MEITACPYALDVSGRDHAGQAAELQKRGPLAQVELPGGVVVWAATAHRYV

KRLVADARVSRDARHWPAFTGGQVTEDWPLYYWVAAQNMFFADGERHARL

RRLVAGAFTARRTEALRPQVEQITAELLDATAAAAAAAGGRVDLHTSFSR

VLPMRVICELFGVPRDTRESLCEELGTTVSTVATAQETTASQMKVFALLG

DLVALKRSQPGDDLTSALIEVRDHGEGLTEDELVGTLNLMIVAGLETTSI

VIENAVVALLSRPEQLGHLRSGRAGWDDVVAETMRVHNPVAFSPLRYAVE

EIDLDGVVIGKGDPILVNFSAPGLDPQLYGDDAAAFDVLRTDRRDSLGFG

HGVHRCLGAPLARLEATVALAALFGRFPRLALARPAEELQPRGSFIVNGY

RELPVLLG

>CYP107Q(2585321850)Streptomyces mirabilis YR139

MTVTAETTADPVELLSPELVADPFAGYARLREQAPTLTGAMLGGPPMWLV

TRYDDVRRVLTDPRFLNNPASVPNAPAVRTTIMKQLNIPSDLVDYLDDKL

MQIDGDHHRRLRSLVSRAFTARRVAQLRPRVEKITAELLDRLAEEGRDGT

PVDLMESFCYPLPFTVICELVGIDEADREPWYEWGNCLGSPTEHRERIPA

VMRECVDHMLEVIARRRAEPRDDLITALVQAQEDNGDRLSEQEMVTLVFT

LVITGHETTTQLLATAVLALLDNPDQLALLREDSSRWPQAVHELMRLGPT

QWGMPRYPSQDVELGGTTIPAGSTILPLILPANTDPRQFEDPERLDIGRD

HGNGQNHLGFGRGAHYCLGRPLALQEAEVALHALFTRFPDLSLAVEREQI

SWVLRPGVTRCEQLPLRLA

>CYP166A(2585322150)Streptomyces mirabilis YR139

MSDAISFEEPWARTHQFDPPEIFDALRRKRPLARMAYPDGHVGWIVSGYE

LARKVLSDPRFSHSTEIGHFPVTHRGQVMPNHPRIPGMFIHMDPPDHTRY

RRLLTGEFTARRANQLTARAKAVAAEQIAVMREHGSPADLVANFAKPLSL

RILSELVGLPYSEVDRYGHAPTLLHDSDADPQQVAAAMGQAGAFFAEVVE

LRRKEPEDDLISRLIADGQLTTEELCNIVTLLLFAGYETTESALAVGVFA

LLHHTDQLAALRADPSRLDAAIEELLRYLTVNQYDTYRTALEDIELNGET

VKKGDSVTVSLPAANRDPAKFTCPADLDIGRETSGHVAFGFGIHQCLGQN

LARVELRAGFSALLDAFPDLCLAVDFEEVPLRLKGSVFAVKSLPVSW

>CYP158B2(2551890196)Streptomyces sp. AA1529

MAQCPLYDVPADPPELEFDPFLRAALESPPSRIRLPHGEGDCWLVTRYDD

VRFVTSDPRFSRDIVGRPTPRMTRHLIPLDRAVSFVDPPGHARVRSVVAP

VFGSGAVERLRPRVRALVAELVDGMLAAGPPADVVRYVVSPLPLAVVGEL

LGVPPEDRTQVRDWAVTLLTRASDDAAAERAQQVKAAARRYFRELAERRR

SRPAGDLMTRMVAAVDAGRIDEEELLALATLIGLNGWHAVRNNVSNMVYL

LLTRPELRERLRAEPESVPRAVEELLRWIPHKHGVGQPRIATEDVEVGGV

LVRRGEVVQVSYVAAGRDPRRFPDPDALDIDRQGPPHLAFGHGPHHCVAP

LLARMEAEELLSALLRRLPGLRPAVPAGEIAWQGSVLIRGPVGLPVTW

>CYP107MK1(2551890365)Streptomyces sp. AA1529

MTTLTGPGREVLDMSFFQDPYAVYDRLRVEGPVHAMRTPDGIDVWVITRY

EEAKEALSDARFTKDFSGVPDIFAQQSTGVSAHEGYTDILTKHMLFADPP

DHTRLRELAAKAFTRRRVEALRPEIQQIVDGLLDTISAAGEVDLLRSLSF

PMSMAVTCRLLGIPPADEDAFRTWTETIFTTNDPARMNEAVRGVDQYLVD

VIEAKRADPADDLITALINAREDGDRLSDRELVSTCALMFVAGYETTASM

VGNAVLALLKNPDKLAELKADPSLMPNAVDELLRFDGPVNVATLRSTATD

VTVAGTTIPKGQFVLVSLIAANRDGEKFPEPARLDFTRPLGGSLAFGHGI

HYCLGAPLSRVEIEVALSRLLARFPDLSLAREPETLQWRNSLLVRGLSTL

PIRLH

>CYP1031A(2551891318)Streptomyces sp. AA1529

MSTVPQDQPLIYYPFTPEFMEDPYPHYAELRRQVPVHEHPGGFWMLSRYA

DVDALLRSKHSVDQRHVAPGPFRDAYARAGVSEKPRLKGLALLDLDPPDH

TRLRKLVTKAFTVRAINGLEPMVRELVDEALDGIAAAGGGDLVEELAFPL

PFTVMTRMLGMPPIDTKHLRMLTSLLMRSVELTTDPEVMRVIEAADAEIF

EVVGDAVSRKRDDPGDDLLTALIAAEDDGDTLSHDELVAQVTTLFVAGYE

TTVNLISGGTLALLRNPDQLELLRARPGIGENAVEEMLRYDPPTHSSRRI

TLEPYHVGGYEIPAGSMVLANLAAANRDEEFFGPDAEELRLERENARKQL

SFGGGMHYCLGGALARIEGRVAIGELVRRFPGLAMDGPVEWNGLLTLRGA

ERLPIRV

>CYP2378A3(2551892216)Streptomyces sp. AA1529

MPAAPSSDVPLFDDRALADPYPLYAQLRETGPAVYLTRYGVWAIPRHREA

EAILHTPDTYGSEGGVALTEPANTRILAGTVLASDAEQHIRLRRVLSAQL

APRTMRRLVAVVTARAERLVEEHVADGGFDAAALARHMVCDTVMELMGLP

EETRAYLLTGAAATFDVFGPDNERYQRALPVASRMVAFLHEAVTRDTVAP

ESWMGAIFQAVDDGRIEEKDAIPLASAYTAASMDTTILGITEAIAQLARH

PQQFAWIRQDPTCATPAFHEALRLEAPIQGFGRMVTETADVDDTRLEAGE

QVWLLYGSAGRDRLAWGPDADVFNVRRRRADRHLAFGGGPHLCAGIPLAE

LQARAVLRALAAQCTNLTTAGEPDRVLNNLLRGWERMPLAVKRASATPAM

RAQADHP

>CYP1192A(2551892378)Streptomyces sp. AA1529

MHLPERPAGYDRARHGVHLLRSPEVIADPSVYIDAIAELGPLFYDEVGSV

WVCSGYAQAVEILRDHSRFSSVREYDHAALRERGLHTSAALSAMVHEQML

FLDPPQHSAVRSTLAGQFSGKRIKSRESDLRAIADRALEGLPAEGVLDLV

DDFAAKLPATLVAYLLGMPGREDDLTRWAEAYERLLGNLSALQAPSDPQV

ERDLTEALSVLQQEAQDRLRAPGTDVISSLTTPLADRTPSEEELFAIAAN

CIVLVGGGYQTLPHLVTSALLALHDDPACQQKLRAQPERIPSAVTEIMRL

NGSSQYVARRATADVTIHGTRIATGDNVLVHLAAANLDPQTFTAPGTLDL

DRSAPKHLGFGTGRHACPGAGYAERLAGFAIEGFLTKYPAYAPEPGPDAL

SWGLHGNTRCLDHAHIRVPAAAPASAADPAAEVNTADHAVDEHARLDGCS

SLRPPSDPPPAHCWHEVFEQQARLTPEVPAVQGPHAAVTYRELDQRANAL

AHRLRHLGAQPGAVVAVVMERSVEFALAVLAVAKSGAAFLLADITCPHER

LRTMLTDADACLAISDGTVPASIFPAPVISTDAPDPRPDAPLTGVSAGDS

AYVVFTSGTTGAPKGIGISYEATVNLSLAQREIFKLCPGDRVLQFLSPNF

DGCFADLTLALLSGATLIVAPADQLTVGPPLVRLLTSQKVTTVILTPSVW

SALPDQPLPDLRIAAAAGERLPAAWVRRWAAPGRRLLNLYGPAETAVLAT

WHECTPGEERPPIGRPVANKQVHLLDEALQRVAPGQEGELWLGGVGVGRY

LNQPDLMEERFIRDPHTTTGPSSLLYRTGDICRQRPDGALDYVGRRDRQV

KIRGQRLELDEVERVLESAPGVADCHVHENDGRLHALVVPTGTDLDEESV

RTHLAGHLHSAMRPHTLTAVAELPRTQNDKADHSTTASAGSASHHSATAA

PHRRHSRLTWQVAQHFAQSLDIPLRQVQADSDFFTSGGDSLTLAAFLQRL

ESLTDAPVDTSALINAPTPEQIASLLLAEGTPA

>CYP107MH2(2551892389)Streptomyces sp. AA1529

MNDARTPRTRTPATVDLRPHAEALLRDPNPVYARLLEQGPVHRAVQPDGA

EAWLVLGYPEAKEALAHPALSSDPAQADRNWRARYLGDPDTTEFPHGRNM

LNSDPPEHTRLRKPTTQAFTPRRIEGLRSTVEEHTERLIRALPETKPFDV

IDDFAAPLTLSMICTVLGVPDLDHGRIREWADRVSFPKIPQDAITARQDL

IPYFDELIRTKRQEPGTGLFDALLETADTDLLSHEELRATAFLLLLAGHE

TTISLLSNAVLCLLTHPEQLALLRRNPDLANQAVHETLRFAPPAPSPFPR

FAAEDLTLDSTEIPGDGSHVKIMISSAQRDPREFPEPDVFDISRPPGYLL

AFGHSIHGCPGRSLAHLEASIALPALLERFPDLSLATTPDRLNWRVGPLL

RALDHLPCQAHAAERRPTKPTRAA

>CYP157C(2551892403)Streptomyces sp. AA1529

MTAPSSDPAPHAPGTPPPGCPAHAGGQPVGRRLYTEDAIRNPRAVYEELR

EQHGAVAPVLLHGDLPAWLVLGYRENLKALQTPSVFSRDSRYWRDMEEGR

VPSDHPLIPITAWQPLCVFVDGEKHRRLRGAVTESLDAFESRGIRLHVTQ

HTDRLVDEFAEAGQADLVGQFAAQLPLRVMTQLYGMSDEHGGEYGPAFIE

AVQDALRGTATSAASNDFVTRTLDDLVARKQDTPAHDLPSMLLAHPAGLS

TDEVREHLRLILTAANETTVNLLANTLHMVLTDSRFRADLAGGHMTLPDG

LEQVMWDEPPMMTLLGRWAARDTALGEQRIRAGDLLLLGLAAGNVDSAVR

DEDTPVIGNRSHLAFSRGPHECPGQQIARAIAESAIDALLRRLPDLRLAV

PEEELRWSSALMYRNLAALPVEFSPVSRKLPVLDADAAGTLRAKLPSAVA

AAPAAQGPGAGAGGPAAGRKRAPWWKPWAHQS

>CYP1419A(2551892673)Streptomyces sp. AA1529

MRGPGARARTPGARTARRRDRRVYTRSHPVLFALLAATRRRPVVRLGRTV

LVHGTDACRQALTRLPLDRTAAGTTGGAARELSGGGALFDEEGRGHRGAR

RSLAEDLGTAGTERLRPVWQEILARRLAPLADGREVETAALARELAGATV

CALLGTAGDPAAVARAAADAAAAAVRDHLPGPRRPGTARAAARATARLEE

LLRERTGGAAEGAGAADGAGTPDGAGTPEGTGAALRAVLAVAAVATTAAA

LPRAVAWCADAGLWEQAADPAARETLVDELLRVTAPSPVLPRRAAAPGTL

DGCPVRSGDRLVLVARHAVGAHRDAPGCPAPAPAAVARLVFGAGPHTCPG

ARLARTQLADLLAALAPYRPVVVRARADRRAALPGWRTLTVRPGPAPSTP

RRRMHRDEEGSTHPNGVHGGNGVHGGNDRNGEAP

>CYP107U(2551892707)Streptomyces sp. AA1529

MPPLFDRQFALDPYPAYAWLREHAPVRRTRLPSGVEAWLVTRYADAREAL

ADPRLSKDPRRHGEQDTHGKGKVGIPGERSANLMTHLLNIDPPDHTRLRR

LVSTAFTPRRVAAFAPRVRELADGLIDSFAPRGEADLIHEYAFPLPIYAI

CDMLGVPQEDQEDFRTWAGMMIHQPGSPRGGVGRAVKRMRGYLAELIHRK

RAEADTAEGGGDLISGLIRASDRGEHLTENEAAAMAFILLFAGFETTVNL

IGNGTFALLRHPEQRAVLQRAVAEGDEKLLGGAVEELLRYDGPVELATWR

FATRDLVLGGQRVREGEAVLVVLAAADRDPAKFAAPDTLDLVRQDNQHLG

YGHGIHYCLGAPLARLEGRTALAALLHRLPDLRLAADPGTLHRRGGLIMR

GLRTLPVEFTPAEADDLRGEPV

>CYP107L(2551892916)Streptomyces sp. AA1529

MADRSVLDLSPDTDGIVDLAVLGDDFVRDPYPVYAALRRRGTVHHVRTPE

GALGWLVVGHDAARAALNDPRLSKDWANASPEAGTLSISPGTHMLISDPP

DHTRLRKLVSRQFTPRRIAALEPRIRELTAGLLDRMLAHPDGRADLVDSL

AFPLPIAVICELLGVPDLERERFSAWSDHVLSAAPEEEKRGSAEALGGYL

AELLERQRARPGEDLLSLLIRTSDEDGDRLSTGELHGMAWLLLVAGYETT

VGLIANGALALLCHPDQLAELRADPALLDGAVEEMLRYDGPVETSTYRFT

REPLEIDGTLIPGDGRLVLPVLADADRDPARFPEGDRFDIHRESGGHLAF

GHGIHFCLGAPLARLEARIAFRMLLERTERLELDASPAALEWRQGTVLRG

VKSVPVRFG

>CYP1251C1(2551893008)Streptomyces sp. AA1529

MTGAAAHTRRDPLYDPLAPEVIAAPHAAYRRLREHRRVYWHAQLDSWVLT

GHAECRRVLGDTAAFGSDFRRVGEAVPDAQLSVQSLDPPAHSAVRHLLVS

ALHVRSHTAVTDTAARLAAGRLDALRGAGSVDLVSGFARPLALHTMCDFL

GVAPPDGPGFEEMSNAIVRSMDAGLDPARAEPGTRARAELSRLVGEWLET

AGEDGFLGAARAARAQAPEVSAAVLANSLRAVLHAGYESVSRLLGNALAR

LVDDPALLGRAMARNALDPLVDELLRLDGPVQADARVCVADSELGGRRIR

PGEVVVLLLGAADRDPEVFADPDAVDLNRRRGAHLAFGRGAHACLGAGLA

TLQLRAVLSALHTAGIGFRAQGPAQYEPTATLRGLRALPVAVSEPAAAC

>CYP107AE(2551893291)Streptomyces sp. AA1529

VQESASSRQETHGQGGSPRRDEPVRAVRSGPAGRTGHLVTGYTEARQALG

DPRLSKDTAAFFANAPARRRLHPAVTRSMLASDPPRHSRLRKLVTGAFTT

GAVAALRPFVEQVTAALLRRWHSPGPVDAVEELAMPLPVAVICELLGVPA

SDRADVRRWSADLFAAGRPEVIDAASHTLADYMAGLVAEKRIRSGDGLLD

RLIAARDGADRLSEEELVSLGVLLLVAGHETTTHFIGSSLLALLQHPAEL

ALLRRAPHRVRDVLDELLRHSSPVSTATFRFTTQPSTLGGTDLPPGCPVQ

VAIGTANRDPGRYPSPDRLDLARDASGHLAFGHGIHRCLGAPLARLEGEV

ALRMVLTRFPDIRPAVDPAELEWRPTRLVRGLAALPVLV

>CYP157A(2551893641)Streptomyces sp. AA1529

VTPPQHPGAASAPPPGCPARPDSGAVPLLGPRFQNDDRARLYREMYQQHG

PLAPVLLPGDIPCWLVLGYRELHRVTSEPALFSRDSGLWNQWPNIPPDWP

LLPMVGKQPSILYTVGERHRRRAALVGDALAAVDPFELRNHAERFADRLI

DGICGRGSADLIAEYAKVLPALVLARLFGFSDEAGAELVPSINALVDGGP

DALRGRDRVQAAMHSLLAARRTAPGAGVASRMLRHGHAPEFTFEEILEDM

VVNIVAGHQPTADWIGNSLRLMLTDERFAASLSGGRHSVAEAMNEVLWED

TPTQNIAGRWTTRDTRLAGRHIPAGDLLMLSFAAANGDPRIRPDRTVLTG

GNSAFLSFGHGEHRCPHPAQDLAEGIARTGIEVLLDRLPDLDLAVSPQAL

VWRASPFLRGLSALPARFTPTPTSTTGGPR

>CYP154C(2551893642)Streptomyces sp. AA1529

MTPCPVTGTGPDSGAGRAAATDPVIVLDPLVRDLAGEGARLRAAGPLAPV

ELPGGVRVWAVTRHAAARKLLTDSRLVKNIEHWAAYRRGEIPPTWPLIGL

ADPGPSMLTFDGPEHRRLRALTAQALTPRRVAALRPRIEEITHTLLDGLE

AAADAEGRVDLKSAFAYPLPMAVIGDLLGIDTTRIPRLRTLFDGFFSSVT

PAEEVPAIIAELGSIFGAEVARKRESPGDDLTSALLAAAEDGDSLSDEEV

VATVQVLITAGHETTISLLVSAVRALLTRPEQLALLRSGEVGWEAAVEET

LRWDAPTTHVLIRFATEDLEVGGTVVARGDAVIISYGAIGRDEEQHGPDA

ERFDLTRTPTRHLSFGHGPHVCPGAPLSRLEALVALPALFDRFPGLRPAV

DPAELRNKPAITQNELHELPVRLR

>CYP183A(2551894103)Streptomyces sp. AA1529

MTEESTFSAGTAPGAFPVVGHALQMMRHPVNFMTSLSAHGDLVEIKIGPT

RAYVPTHPELLRHVLTNDRIFDKGGVFYDRARDIAGNGLVTCPFADHRRQ

RRLMQSAFTRGQLKRYSEAMHAEIEATASRWQDGMVVDAFQEMYGMALRT

VGRTLYSTPVSPELAAQVERSFDVVLNGLFRQMFLPASVRRMPLPSNRRY

RSNLDFLHATTQRLIDDYRADGTERDDLLAALLASRDDDGGRLADREIHD

QVITVMAAGTETVAGTLTWVFYLLSQHPEIEAALYEEMDTVLEGRAPQWD

DLPKLSLTDRIISETLRLHPPAWLFTRLTAAPTELAGRRLPAGSTVVFSP

AAVAQYADAFDDPATFDPDRWLPDRVAPASRHAYVPFGTGARKCIGDLYA

RTEAALGLATILGRWRVTCEPGMDIRPVPLATVYHPRRLRLRLDARTPRR

TAAAVPAPAGGDPT

>CYP161C(2551894111)Streptomyces sp. AA1529

MEELPTLPFDNPSVLGVAPRMRTLQQEGPITPVRAAGEDAWLVTRYDEVR

ALLADRRLGLSNPYPARQAKTTARNTMMALMAGDDYETEAADHPQLRELL

VPRFSTRRMRMMKSRIEQHVDELLDQLAAGPQPADLHRALSFPLPTMVVC

DLLGVPWADRERFGQWARGTFDQSDDGRHSAHTFQQVVEYTTELVARKRT

EPGDDILSELIAAKDGSLSDAYIAQLGNAVLLFGYETTIVRIDLGTLLML

RNPEQRALLADKPELAPGAVEEILRLAVGGKGSNALVPRYAHSDITVGDT

VIRTGEAVLLAIGAANVDGHAYPEPDLMDITRDRPRPHLSFGHGTRHCVG

RVLARIELTAVFERLFRRLPELRLAVPEEELRWQEHRITGGFDEIPVTF

>CYP1237A4(2551894200)Streptomyces sp. AA1529

VSTQTPPRYPFAWTPPMQVPEALRHVHSSSAMEVTLPSGDVATLVTRYKD

VRALFADKRLSRNIARPDCARISADNDLFMDPEIDPDPPKHTQVRSLVTK

AFTARRIEALRPYVQRVADELLDEMAAGPRPVELNEALAFPLPIMVICKL

LGVPAEDRDQFREYVDGFLSVTKLPPEEVGQCRQNLWKYLGDLIDAKREK

PGDDLVSELIRVRDEEDNRLNDHELHFWCQGLLIAGYVTTASQIGTGTAV

LLHHPELVREIQADWSLVPSTVEELLRTQIMGSSVGTMRYAVDDIPLSDG

SVIKKGTSVLLSEEGANMDPEVFDQPFELDIRRQENHHMTFGAGIHYCVG

AALARMELQVATESLLRRFPDIRLAAPAEELPRALGGFMEGFTEIPVEW

>CYP107A(2551894254)Streptomyces sp. AA1529

MAQDSATPTPVRPYPPPRRSATEVPEDYALLRAEEPVAAVTLPSGDPGHL

VSRYDDVRALLADPRCSRAATVAPEAPKLTAVPFDAGGLFTMDPPEHTRL

RGLVARAFTPRRVERMRPRLEELAGELADAMAAGNGAGGGPTDLNTAFAF

PFPMAVICELLGVPFADRERFRTWSDAVLSLTAHPPEEMLRHKQALLAYL

ADLVADKRRAPGTDLLSALVGVRDEEGRPDERELLVLAMTLLVAGHETTA

GVLGTSVLTLLRQPERLGLVPDGEEATAALVEELLRLNPVGDGGPLRVTT

EPVEVAGRVLPPNSAVIASVCSANRDGSRYTDPDRLDPDRTGDPAQPPHL

AFGHGPHYCLGAPLARAELAVALRVLAGRFPALRPAVPVESVTMHRGLLV

NRLTELPVRWD

>CYP154C(2551894301)Streptomyces sp. AA1529

MNGDAQSPASEPRTAGAAPAGCPAADGGDAPGTAGCPLTIDPLVGSLAAE

TEALRTAGPLARIDLMGAEVWTVTTHAEARRLLTDTRLVKDITRWNLWRD

GEIDENWPLIGMVDAGRSMFTVDGAEHRRLRVKTAQALTPRRLEELRPVV

ERVTARLLDAMASDAAEAPDEPIDLKSAFAQPLPMSVVCSLMGVDPALEP

RLHHLYEAFFSTLTPQQERLAVLRELDELYHDMVRHKTASPGDDLTSALI

LADEGGEPLTAEEVRGNLEAMVAAGHETTVTLILCAVRALLTHPDQLRLV

LDGQVGWDAVIEETLRWESPSTHLLLRFATSDIEVGTGGAVIREGEGVAM

SYRAIGRDRAQHGQDADRFDVRRPAPIRHLAFGHGPHICPGAGLSRLEAS

VALPALFERFPALSAVAAAETRHRPVLTQNELECLPVRLGVG

>CYP157A(2551894302)Streptomyces sp. AA1529

LTSQPQYYDPAHGPPASGAPPAEPRGCPAAAGGRAAPGAVPLMGPRFHTD

LEAVYRDMRRDHGPVVPVTLPGDVPAWLVIGYRELHHVTGDPVLFPRDQG

LWNQWKNLPEDWPLRPMIGTPQPSVYFTVGQEHRRHLAMVQQALEGVNHV

ELRGTTEEIADRLIDSFCGVGESEIVSAYAKPLPLLTLARILGFPDSDGP

ELIRSLTDMADGGPHALAGFEHALSLMRRLVARKHAAPGADVLSAMLAHP

AEFTDEAYVLNLMAVTSAGFLPTADWIGNSVRLMLTDDRFAASLSGGRHT

VPEAMNEVLWEDTPTQILAGRWAARDTRLAGRAVARGDLLLLGLAGANRD

PAAHAPHGADRPRERNFAHFAFSHGEYQCPFPAQEIAEVIARTGIEVLLD

RLPDLDLAVAPRTLARRPSPFLRGMTVLPVRFAPTPPYGGR

>CYP154A(2551894810)Streptomyces sp. AA1529

VSVPELLTLDPTGSDPHGEHQALHARGPATRVDLLGVTAWSVSDPVLLKK

LLTGPDVSKDARQHWPQFAETVPTWPLALWVAVENMFTSFGSDHRRLRRM

VAPSFSARRVAALGERVESIVDDLLDVLARTPEGEAADVRELFAYPLPIQ

VICHLMGVPAERRAGFRQLVDNVFATTLTPEEAAANTASLYGVLDELIAE

KREQPGEDMTSLLIDARDEEGDGSALSEAELRDTLLLMISAGYETTVNLL

DQAITVLLTHPEQLALVREGRANWADVVEETLRFEPAVKHLPLRFAVRDI

PLPDGQVIAKGEPVLASYAAANRHPDWHGESADTFDLTRTDPGPEHLAFG

HGVHFCLGAPLARLEGITALEKFFARFPDARLGVPEEELRPVPSLISNGH

QTLPVVLRPRSAAD

>CYP1240B(2551895332)Streptomyces sp. AA1529

LSTVPAPGHPSSGQMPPPLSRPNHEVTPLYGEEFAADPYAVYDRLRKYGA

LAPVEIAPGVGAMLVIDYRAALDLLHDSATWSKDSSIWLDSVPEDSAVMP

MLRGRPNALFTDGETHARYRKVISDSFGRQEPHEMRRDVQEVADNLIANF

ARDGEADLIGQFARLLPLLYFNRAFGMPDEESELLIQGIMGMFDSKTPEE

AAAADAAYTRYVTELTQLKQRQPGQDLTSWFMQHPAGLSAEEVVQQIVLT

LAASYEPLSNLIGNSLSRMLVDDRYYGNLSGGALTARDALHDVLRNEPPM

ANYSAHYPRRDVYFHGVWLRAGQLVLVSYAAASTQSGRSAPGESTGAAGS

GGGAHLAWAAGPHACPAQQPALLIATTAIERLTAWLSDIELTVRYDELSW

RPGPFQRGLVSLPARFSPVSPDQAGVPR

>CYP180A(2551895399)Streptomyces sp. AA1529

VSGAVPEPGVPGPASAAASPEPAPQPAPGSAAPAPGSAAPAPDRDVFDPR

RYARGLPHEVFRALRDGEPVAWQPEYEVLGWPAGPGFWAVTRHADVLRVL

RSPGDFSSYEGATQIRDPAPEDLPFIRRMMLNQDPPAHNRLRTLVSRAFT

RRRVERFEAQVRQRARRLLTGALRQARERGSCDLVAAVTDDFALLNLADL

LGVPTGDRGLLLEWTERIIGYQDPDQARTVLGPDGRPADPRSPAMLQEMF

DYARRLASHKRAHPADDVMTALAHGLTGAELEMFFFLLTVAGNDTVRSAA

PGGLLALAQHPRAQCTLRELVRAGQVRAQAVAVEELLRWHTPVLSFRRTA

VRDLELSGRRIGAGEKVVVFHASANYDERVFDAPHTLDLARAHNPHVAFG

AGPHVCMGAHFARLQLRVLHQEAARLLPPYELLQQGEGPGRCAG

>CYP107R2(2551895506)Streptomyces sp. AA1529

VTTHSRQVRDFPFETPARLSMEPLFAALRESEPLSRVRLPYGGEAWLVTR

YEDIRTVLGDPRFGRAGTLHERAPRIQPDPAGEGVLMSLDPPDHTRLRKT

VAGVFTKRRVEELRPGTERIARELLDAMEAAGPPADLVTSFALPLSVTVI

CDLLGVPRQDREKLRSWSDALLSTTACTPAEMAASTEALADYFATLVRQR

RQEPAGDLLGALVDICDSRQGRLDEEELVLLTRDLLIAGHETTASQLANC

TYLLLQQPDGAARPDADGRMPMAAVEELLRFIPLGSGSFRARVATEEVEL

CGRRVRPGETVFAPTVAANWDPGVFSDPGTLDWERSPNPHLAFGHGVHHC

LGAQLARMELQVALGALLDRLPGLRPAVGEGEIEWKTGMQVRGPRTLPVR

W

>CYP157K(2551895579)Streptomyces sp. AA1529

MNAQPSDGTRLEAPLPPAGCPVGHTGAATRLYAAESTADPYATYSRLRKQ

YGPVAPVLLEGDVRAWLVLGYRENRRALDNPGQFSRDSRRWRDWQEGRIE

EMSPLIPMLGWRPDCLSQDGEPHQRLRGAVTDSMATVANRGIRRHVVHFA

HKQIDAFAHTGRADLVPQFSEHLPMLVLTRLFGLPESDGEQLVASCARMM

RGGDGAVEHNERIMRVLAGLTAHKRAEPGPDFATGLIEHPACLDDDEVQN

HLRLVLIAAHTTTSNLLARVLQAVLSSSSHLADLVSGQLTVTALVEETMW

NAPPLAVLPGRFATADCELGGQQVEAGDLLILGLGPGNLDPEIRPRTGTA

VRGNQAHLAFSSGPHECPGQAIGQAIIETAVEVLVHRLPGLRPAVDPAEL

TDTASTWETRLDHLPVTFPV

>CYP163B(2551895680)Streptomyces sp. AA1529

MSSLITPDSGALDTLDLADPRTHAEYDLTQVWQRLRKEQPFYKHRPVGGS

EGFWVVTRYEDVSTLYRDTARFTSEKGNVLTTLLMSGDSAAGMMAAVTDG

PRHNDLRRILLKAFSPRVLTGVVENIHAAARQLLLDAVAREECDFARDVA

AHIPLNAICDLMAVPQADRPFVLECTEAALGSDGAAQSPAVAWQARNDIL

AYFEKLATERRKNPGTDAVSMLATGTLEGKPLTMDEIVVNCYSLILGGDE

TSRLSMTGAVAAFLEHPDQWRALLKGEVEVSTAVEEVLRWTTPAIHFGRS

ALEDVPVGDRGQEIKAGDIVTLWNNSANMDEEVFADPGRFDLARTPNKHI

AFGYGPHFCLGAYLGRAEIAAMLTALRDIVATIEPNGEPRRVYSNLLSGM

TSLPVVLRPA

>CYP159A7(2585296949)Streptomyces atratus OK008

MQADAPDGIPPHTSAPDLGRRADRTARPGTGPSLVAPGAAHDPYRLYRVL

REEYPLSYDAPLGAWLLSRHADVSTALTDPRFTGFPHDGAPRGGPAPRGL

CHGSMLCLPRPQDIARPTATVPRHLTERVERTAYVLARRIAGWQQADLVE

EFCRWLPVGAAAPGASPDPAQAPGNPTAPCAGRTGLRETALASFLANLLD

DPDLLAALRIETALAHRAWTESLRRDPPVQIVLRRTVAEVRVSGGTLLAG

APVACLIGAANRDPERYAAPDLFDPFRTDQGRSVAGPESCPAVLLGRLEA

EQGVRALLDAMPRLRWADGFRPCSTGLLTRGPQTLLVQPG

>CYP147B(2585297118)Streptomyces atratus OK008

MTADTLLERITDYANRPDPYPLYAELREAGPVVRQTDGSYLVGTYHEITA

LLHDPRISTDPRNRPTPPSGPSEEVPQPPFLRLDDPEHHRLRTLAMRPFG

PPHSPGRIDAMRDQIADITKELLEALRDRKHIDIVDDFAYPLPVTVICRL

LGVPREDEPLFRQWSDVLVATADVRPEENTTEQDRAGRQARAEMGQYLLN

LAEQRRGRPSDDMLSAFINEPDPARRLTREELAETAILLFIAGHETTVNL

ITNGVLTLLRHPGQLDRLRRDPDLLPGAVEELLRYEPPVHMRERIPLADI

EVTGASIPKGSLVVLALASGNRDPKRFHEPDRFDPGRTDNEHFGFGSGIH

LCYGAPLARIEAQTALGALIPHLNAATLVQDPPPYRQNAMLRGPRHLLLE

VQQPI

>CYP107P(2585297897)Streptomyces atratus OK008

MHPSSQPVATASFDPWSPAFVADPYPAYAALRAAGRVHRFEPTNQWLVPH

YADVSALLRDRRLGRTYLHRFTHEEFGRTPPPAAHEPFTTLNGQGILDLE

APDHTRIRRLVSKAFTPRTVEQLVPTVQRLAAELVDSFVEADGGDLLAAV

AEPLPVAVIAEMLGIPESDRGPLRPWSAAICGMFELNPSEETASAAVRAS

VEFSAYLRELIAERRKNPGMDLISALIAAHDEGERLSEQEMISTCVLLLN

AGHEATVNTTVNGWWTLLRHPEQLAALRADHGLLPTAVEELMRYDTPLQM

FERWVLDDIEIDGTVIPRGSEVALLFGSANRDPARFANPDTLDLSRQENP

HITFGAGIHFCLGAPLARVELAASFGELLRRAPRMRLAAEPEWNPGYVIR

GLKELRVEL

>CYP157A(2585298291)Streptomyces atratus OK008

MTRLSSAVPAPGPETPTGGCPVRHGSAAVPLSGPEFHTEPQALYRTMRRD

HGPVVPVELPGGFPAWLVIGYRELHQVTSDGELFPRDVGLWNQWESIPED

WPLLPMVGRPMPSIYFTAGAEHRRHADMVGPALEGADPFEIRRHCEELAD

RLVDSVCTRGAADLVAEFAEPLPVLVLARLIGFPDAEGADIAQVLKDLAD

GGPEAQRAHVRFGEHMQRLVAAKRAAPGDDVTSRMLAYHEPFTDEEYVLD

LMAITAAGHLTTADWISNSLRLMLTDDLFADSLAGGRHSVAEAMNEVLWE

ESPTQILAGRWASRDTRLAGRDIRAGDMLLLGLGAANSDPHVRQRIATEG

RSGQGGNSAHLAFSYGEYRCPFPAQEIAETIARTGIEVLLDRLPDLALAV

PAQTLVRRQSAFLRGMTSLPVRFTPVRMTGDLS

>CYP154C(2585298292)Streptomyces atratus OK008

LNCPHAAAHEATTGGGTVVIDPMVQDLDGETARLRDAGPLARIELLGVPA

WTVTRHAEARQLLVDPRLVKDLDAWGLWRSGAVTHAWPLIGMIDAGRSMF

TVDGAEHRRLRTKTSQAITPRRLEAIRPAIEKFTEELLDALAEQGKDGVV

DLKSVFAQPLPMRVVGMLMGVDEAEHPMLTKRYKAFFSMLTPQEERLALL

AELDVFYAALVREKTAQPTDDLTSALILADEGGEPLTEEEVVGNLKAMVA

AGHETTIGLILNAVRALLAHPDQLGMVLDGEIPWETVIEETLRWDTPTTH

LLMRFATEDIQVGDQVIAQGEGVVISYRVIGRDVEQHGPDADAFDITRPT

PIRHMTFGHGPHICPGAALSRVEAGIALPALFGRFPGLRLAVPDGELRRL

PVMTQNDMESFPVLLNG

>CYP1047A(2585298303)Streptomyces atratus OK008

MSTETGPADDAEPCGHVPVPGPKGLPFLGNLPQFGKNPLEFFERLRGYGD

MVRWRFGPNPCVFLADPECIGELLTETERTFDQPALGIAFRTVMGNGVVV

ARGPDWRRKRSLVQPSVRPKQVRSYAATMAGSTVELADTWSGGERIDIKR

EMAALTQKIAVRTIFGVDTPADAEAMGRAMDVAQQEIGKEFSGIGALLPD

WVPTPGRARIKKAAAVIDAEVGRVVARHRDGDGERPDLLSRLLTAVDETG

AHLTDEEIRDETVTLYIGGHETTSSTLVWAWYLLSRNPQTRAALTEELDR

VLGDREPEFDDYARLTYTQAVVKETLRLYPTIWLVTGVAKDGARIGGVDM

PKGTRVWTSQWATHRDERWFPEPEAFRPERWDADNGDDIAEYAWFPFGGG

PRVCLGTRFAMVEAVLILAVLARRFELDVDPGDVNPVPTLTLQPDRDVRA

TVRAR

>CYP107E(2585299219)Streptomyces atratus OK008

VPKADITPLTYPFNTPDGLQLADEYERVRDRPGLLRVQMEYGEPAWLVTR

YADARLVLGDPRFSRAAGASHDEPRQSEGRRDGGILGMDPPEHTRLRSLV

AKAFTVRQVEKLRPRVRELTASLLDGLEAAGPPADLVDLYALPLPVAVIC

QMLGVPAEDRPRFRVWSDAALSTSSLTAEELRANREELRAYMAELIDGHR

RSPQDDLMTALITARDGSDRLSELELVDLCVGILVAGHETTASQIPNFVL

TLLDHPDQLARLRAEPDLVASAVEELLRFVPLGSGAGQPRYATEDIEIGG

TLVRAGSPVLVAMGAANRDALRFTAPGVLDIAREGNQHLGFGHGVHHCLG

APLARLELQEALIALITRFPGLRSAGDVTWKSEMLVRGPRVMPVGW

>CYP107U(2585300210)Streptomyces atratus OK008

VNDSPADSPADSPADSPADAPELFTWEFATDPYPAYAWLRDHSPVHRTAL

PSGVEAWLVTRYEDARQALADTRLSKNPAHHDEPAHAKGKTGIPGERKAE

LMTHLLNIDPPDHTRLRRLVSKAFTPRRVAEFAPRVQELTDRLIDNFIEE

GSADLIHDFAFPLPIYAICDLLGVPREDQDDFRDWAGMMIRHGGGPRGGV

ARSVKKMRGYLAELIHRKRENPGEDLISGLIRASDHGEHLTENEAAAMAF

ILLFAGFETTVNLIGNGVYALLRNPEQRERLQRSLDAGETGLLETGIEEL

LRYDGPVELATWRFATEPLTLGGRRIAAGDPVLVVLAAADRDPARFADPD

RLDLARRDNQHLGYGHGIHYCLGAPLARLEGQVALATLLRRLPDLRLAVE

PADMRWRGGLIMRGLRTLPVEFGPGRRSGESDDLSIL

>CYP107F(2585300550)Streptomyces atratus OK008

MEPASTPRNCPFDYAEALEFDPLLKQLQNDEPVARIQLPHGDGGAWLVTG

YDDVRTVTTDRRFSRSAIIGRNFPRMTPEPIVQDEAINVMDPPASSRLRS

LISKGFAPRHMERMRVRTQHVVDELLDRMEEHGSPADLFAHLADPLPLTT

ICEVLDIPEGDDAQLRAHARTMMNVGIDNRDNAVRAKADLRAYFAELTAH

RRENPGDDLISALAAARDGDELLSDQELTVMAMVLLITGQDTTTYEIGNI

AYTLLTRPKELAMLRARPEMLPQAIEELLRFIPFRKGVGIPRVATEDVEL

SGVKIRAGDIVHVSYLTANRDGRKFDRPDELDLERTGPSHMTFGWGGHHC

LGAPLAATELEVALGTLLKRFPDLRLAKPAEEVAWNKTSIWRYPLALPVA

W

>CYP1060A(2585300673)Streptomyces atratus OK008

MGRMNEPPRLPEGSFAAWSRDRLALARRGADECGDVWQLEPGVYVAAAAG

PCEAVLRRAHDFPKVSSPLFPPLKRSSGAPTSEERAHAHAARMRGLRPQA

VAARIGEIAPGTARFVDQWPTGQDVEILPPVRHALAEIGVRYLFSEDAPT

LLPFASQLFVAREVLVRPSRWVWPRWIPTPARRFRTRQQVAFTNALQPIV

RRRRSSGRLGDDVLGQMLQPSSRYGLLPEEAILDTLPGITVATFETPSRA

AGWILLHLARHPQTAGRVAAEAALLPADPAATTSTHLDSLQYTQALVREV

LRLYPPSWLLTRRATRQTQLADYTIDAGSTVLVCPYTAHRDAREHSEPDQ

FQPERWLDDSGSPAKSGVFLSFGTGPHGCEGAALAMAMLTLLTAQTARCY

HLSEPPGPEPSYQVATFEGLATVGLRLRATVRS

>CYP156B(2585300962)Streptomyces atratus OK008

MQQPREPQEIPPGCPAHGNVQMYGPSFGADPESHYTQLRSYGPSAPVDIA

PDVQAELVTSYDAALYVLQNPASFVRDSRRWNALNEGRVPADSPALPMMG

YRPNALFSDGAAHARLRQAVTDSLATVNEHQLIRQTQQSANYLISQFSTD

IRGQAELMAEYAQPLPLLVFSNLFGCPPEIGDRVIAGISGIFEGTPGADE

VLGGALTELIALKRRRPAADLTTRLMEHPAQLSDEEVLHQLVTLLSGGTA

PLAAAIGTSSALYLGEDWQVGLPVEDAVSQTLWNYAPIANYAAHYPTHDV

ELGGRVIRANDPVLISFAAANTDPKLTEHREQLSAKAHLAFGAGPHACPA

KDPAFMIAVTAVEALLNRLPDVEMRVPFKALTWAPSPWSRSLVTLPIRFT

PRGVPQAAESAGSNAGQMPQQQTAASSPSTAGPSRTNAGVAPQPKGGLFS

RFLAWTRGE

>CYP1035A(2585300963)Streptomyces atratus OK008

MGVATTPSYDRRASASLFSRLRTARGQADPFPIYAELLSRGEVVPAPWGG

SVVAGFAACDQVLRSRQWLEPDRKWRERQGPGTRWNAPSSREMSNTLAAL

NPPDHTKVRRAAGTFDRATVERIGRNVNRTADQLLDAFTERIRTGEADFS

ELVCEELPVATIGDWLGLPQADWPRLRQLTHDQVFTQELLPSASQLALSD

AATAELRTYFMDLVRDRRSHPGDDPVTRWIQTWDAIEPDQDKADEAVYFL

VLFVLLAALETTATLLSTMTLRLVESPERWDMIADNPGLVPGFVEETLRY

DPPTHVISRVASQDSVLGGVEIRRGEMVHLMVGAAHRDPARHSDADRFDP

ERTPGHLAFSGGIHYCLGAPLARLEAQTLLRQLIRRLPRLTLVRPPSMAP

RVAFRRLLNLDVALA

>CYP157C(2585301860)Streptomyces atratus OK008

MGPVPPAECPAHGLGVGPGGLRRLYGPEAERDPAGLYDKLRAEHGTVAPV

LLHGDVPAWLVLGHSENLHMTRTPSQFSRDSRRWRALQDGSVAPDHPLAP

IFTWQPVCVFADGATHERQRGAVTDSMERIDHRGVRRHINRYSNRLVNEF

CRDGRVDLVSQFAEWLPMMVMCEILGMPEECNDRMVQAARDMTRGTATAV

ASNAYVVAALNRLVRRRRTEPAADFATWLVEHPASMTDTEVTEHLRLILI

AAYEATANLIANVLRMVLTDPRFRARLSGGHMTVPEAVEQILWDEPPFTA

VFGRWAVGDTELGGQRIRAGDALIVGIAPANTDPVVRPDLAADMEGNRAH

LAFSGGPHECPGQDIGRAIADVGVDALLMRLPDLELAVEESELNWIGNIM

SRHLVELPAGFAARSPQDDNEPPPMGRRRPPGEDWEVQSPDRTPAPVPAG

VAAEAGHGTVGGVRTETSHGASAGPSGRIPQQRRPVAPARLWRAVTRWWS

GY

>CYP105AK(2585302020)Streptomyces atratus OK008

MSIPSARSAIAAWVTRLYVSRLRKKGRGLDLSILSKLPDAALLPLRRDGL

DPVAEIGALRDREPVSRLPIPGMTVWLVSGYDEARQVLGDARAFSNDFAN

LVNHTGVTENHQPGGLGFCDPPDHTRLRRLLTPEFTMRRLSRLTPRIHAI

AEESLAELAKAADTDGRVDFVEHFALPVPALVICELLGVPYEERDAFQQF

SVARFDVLGGVGASFGAISQSREYLHGLVEQQRRNPGDGLLGMLIREHGD

SVTDEELTGLADGVLTGGLETTASMLALGTLVMLQDRKHFAAMRNADDPA

AVAAPFVDELLRHLTVVQTAFPRFAREDMEIGGVRISGGDIVIVSLSAAD

RDPRLGPAMDTFDPTRPPASSHLAFGYGIHRCIGAELGKMELRAAFPKLV

ARFPELRLAVRPQELEFRKLSVVYGVDSLPVHLN

>CYP159A(2585302368)Streptomyces atratus OK008

MTFAPQESHVSPPPDILPPAFVADPYGAYRTMRASAPLIHHEATDSYLAS

RYEDVERVFKDKAGEFTTATYDWQIEPVHGRTILQFSGREHVPRRALVAP

ALPAPTSRRSSCRSSNATLTPVATGSPDPGARHLPRDPFDAWFAVERSRS

GYPPTGRSAVGEGAWLPSRYAAGATTASERRIRRGRPSHRRRHRRTCPTL

GRHHPYDPRSSSE

>CYP146A3(2525013977)Streptomyces sp. PsTaAH-130

MSIDPTALDPATFRPAPVDLADPDLYTTDARFTMWGEYTRAEAKVWSDPG

VSPSGFWSVFSHRDVAAVLSPKAPFTSEYGMMIGFDPEHPDSSGGRMLVV

SEGGWHSTLKRLIGPFLSRLRAPELRSVLHREVRDIVDRLRSQDSTDIAQ

GIGPRLPAAIVCEVIGVPLSEREQLIELTNHAFGGEESSFDKMTPAEAHT

EILFYFHELIEQRRRTPGSDLVSALLEDGRLSAEDVLINCDNVLIGGNET

TRHSITGAFHAFQAFPETLDALHADPELAGSAVEEVVRWTSPAAHVLRVA

TEDCEIGGQEILKGQSVVAWLSAANRDERHFPDPHRFVPDRTPNRHLGFG

NGPHHCLGAALARLELAALLGELATEARGVEPAGEVRWMRSNLVQGYSSL

EVSMNWR

>CYP105BC(2525014084)Streptomyces sp. PsTaAH-130

MSACDQHQLAEWPMPRTCPYAPPDAYRDLREDAPTRVRIRGGDAWLVTRH

ADVRQVLNDNRFSADDQQPGFPIRIQLPPEPGVMSFSRMDDPEHGRLRRM

ALTEFTARRTRALRPEVELLVDRLLDELERRPRPVDLVTEFAVRLPALVI

ARMLGVPEQDEADFTEQSRVVLSQEAGPEETYAAFVTMTRLLDRLVTERT

ADPRDDLISRLATRYVASGELTHDELVAMARFFLVAGHETTAHQIGLSVL

SLLRDPGQLAELRADPVLFKPAVEELLRYWSISQDNQVRVAVGDIELGGA

RIRKGEGIVLAIPAANHDESVYPGAERLDIHRDAAGHLAFGFGPHLCPGA

SLARMELEVCLSRLFARFPALRLAVPVEDVRFRENTLVYGLEELPVTW

>CYP105AH(2525014085)Streptomyces sp. PsTaAH-130

MSLADHADILDWPFARAEDGSPPPILAELRQAPPRVVRIPAGAAESRLGW

LVTRYADVRQALADPRLSADETLPGAPVRIQVPPGGNPSSFLRLDDPEHA

RLRGMIQTEFTARRVKRLREPVQRLVDELLDELAAQPQPADLHAVFSRAL

PTLVIARLLGVPEEDSPFFIEKTRVTISQEDPAVSQAAFVEMSEYLAKLA

LRKLTEPGDDLMSRLAVRHHATGAITLDELVGIARLVLVAGHETTTNQIA

LNILALLRDDDLRARVAADDGALIPNFVEECLRYWSISQDAMVRLALEDM

ELGGARLEKGDAVVISVPAGNHDETVFACPHRIDPERDTSGHLQWGFGPH

YCQGAPLARLEMDLALRTLLRRFPNLRLAADPRTLFRRGTVFHGVTRLPV

TW

>CYP180A(2525014192)Streptomyces sp. PsTaAH-130

VSTGITGPVPDVFDPRRYATGVPYDAYRVLRDEHPVAWQEEPEVLGWPAG

PGFWAVTRHADVVRVLKDSAAYSSHLGATQIRDPDPADLPFVRRMMLNLD

PPHHGRPRRLVSRAFTPGRIERFAALARERARTLLAAAVERAAHGDGTVD

LVAAVTDEYALLNLTDLLGVPTSDRGLLLDWTRRVIGYQDPDEAAAPVLD

GSGKPLNPRSPALLRDMFGYAHELAAHKRRHPADDIMTTLATDPELTGPE

LEMFFFLLTVAGNDTVRGAAPGGLLALAGHPESYGLLRSGKYELTSVVEE

LLRWHPPVLTFRRTAARDTELAGRRIPAGDKVVVFHASANRDERAFADPG

RLDLARSPNPHISFGDGPHVCLGAHFARLQLRLLYAEVARALPELWPAGP

AERLVSNFINGIKSLPVRIS

>CYP107L2525014351)Streptomyces sp. PsTaAH-130

MDRRQAVDLGEFGDAFRRDPHPVYALLRERGPVHRVRIPHADEDYETWLV

VGYEEARAALADPRLSKDGTKIGVTFLDQDLIGRDLLGTDPPQHTRLRGL

VTRAFTMRRVEQLRPRVQRITDELLDEMLSPGSAGRADLIAALAYPLPLT

VICELLGVPEMDRTEFRKISTQVVAPTDPGSEREAMSRLGEYLTELIEDK

RRSGGTGDLLGDLVRTTAEDGDRLSSEELRGMAFLLLIAGHETTVNLIGN

GVLALLTHPDQLAALRADMSLIDGVVEETLRWEGPVENATFRYAAEPLEL

GGVRIEKGEHVMVGLTAAQRDGARFPAPERFDIRRDTRGHLAFGHGLHYC

LGAPLARLEGRVALSTLLERTPGLALDGPHDEWLPGMLMRGLRTLPVRW

>CYP156H(2525014377)Streptomyces sp. PsTaAH-130

VTPYPAPAESFDRSTPVRLWEDGFAADPRRYYAALRAQGPVGWAELAPGV

PAYVVTDRRAALDMLHDDATWSRDPRPWQATVPEDLPILGMMRWRPNTLF

SDGAEHIRYRTALLQAFDLVEPHDLRERVLRAVHILVSRFGPRGEADLVN

DFARPLMTLVFNSLFGFPEGEADRLNDALGALMEGGEASIQGEAEYGRYV

MDLIGAKTARRGDDLTSRLLDHPLGLTTEEVTWQVFLTLGAGHEPSTNLV

SNALSRILGNPAYYSTLTSGSRTVRDAVLEVLRHETPLTNYGVLYARESL

SFHGAWVRAAVPVVVSYGALALFADEEHGGARHPRDASHLSFGAGPHTCP

VKHEGLLIATEAIERLTQWLPDMEPTVPRDRLTWRPGPFHRSLVSLPVRF

TPCTPDRPAGPPAPSASSQTGARA

>CYP154U(2525014378)Streptomyces sp. PsTaAH-130

MTTTPVRVPIDPFGSDIVAESARLRALGPVVPVELPGGIPAWAPTGYDAL

RTLILDPQVSKDPRRHWRLWPEVGEHPEWGWVLGWIGVVNMLSTYGADHT

RLRRLVAPSFTARRTEALRGRVDSVAAELLDKMAESGAGGAVVDLKEAFA

QPLPMQIICELFGVPGHLRPSVAELIAAIMDTSDPSPEHADFVQRQIGTV

IPAQIAYRAENPGDDLTTELIRVRDEEGDRLSAEELLFTLLLVIGAGFET

TVNLIANAVVELLTHPEQLAAVRSGELDWDQVIDEVLRVRPSIAALPLRF

AVSDVTVGGVTIPAGDAILTTYGAAGSDPAHYGETAEAFDAARAADDHLS

FGIGVHRCIGAPLARMEARTALTALFDRFPDLAPAFTAGELAQVPSFIAY

GWQTVPVRLEG

>CYP156B(2525014427)Streptomyces sp. PsTaAH-130

MQQRPGIEPPPGCPAHGNIPLYGPRFGSDPDGHYAHLRGLGPSAPVDIAP

GVEVELVTSYDAALYILQNPASFVRDSRRWNALNEGRVPEDSPALPMLAY

RPNALMSDGAAHARLRQAVTDSLASVNELQLVRQTQASADYLISRFSSER

MGQAELMAEYAQPLPLLVFSDLFGCPPEIGDRVIAGITGLFSGTKGADQL

VAEALNELIALKRRNPGDDLTTRLMQHSSQLTDEEMLHQLITLLSGGTTP

LSAMIGTASALILGEEWQSGLPVEDAVTQVLWNYPPIANYAAHYPTHDVE

LGGRVVKANDPVLISFAAANTDPRLAEHREQLSAKAHLAFGAGPHACPAK

DPAFVIAVTAVETLLNRLPDIEVRMPFKDLSWVPAPWSRSLVALPVRFTP

RIVVPTASQRTETPAAAPGGHVTATPRPAPSGASKPKSGLFSRFLAWTRG

E

>CYP107E(2525014792)Streptomyces sp. PsTaAH-130

VTATDHAPLAYPFNSGQDLDLAEAYERARDTPGLLRVRMQYGEPAWLATR

YADARLVLGDQRFSRALSLEHDEPRASEGRRDSGILSMDPPDHTRLRSLV

AKAFTVRQVEKLRPQVRELTTSLLDAMEAAGPPVDLVDRYALPIPVAVIC

RLLGVPERDRPQFRVWSDAALSTSSLTAEEFDRNREELRAYMAELIAAHR

AEPRDDLMTALIEARDHGDRLSELELVDLCVGILVAGHETTATQIPNFVL

TLLDHPQAVERLRAEPALITQAIEELLRFVPLGSGAGQARYAKEDVEVGG

TLVRAGEPVLVAIGAANRDALRFTEAGALDLTRGGNAHLGFGHGVHHCLG

APLARLELQEAIGALVTRFPRLHLAGDITWKTEMLVRGPRVMPVGW

>CYP105D(2525014945)Streptomyces sp. PsTaAH-130

MTELTDITGPATPADPVAFPQDRTCPYHPPTGYAPLREDRPLARVTLYDG

REVWMVTGHATARALLADPRLSSDRRRGEFPSISPRFEGVRDRRVALLGQ

DDPEHQRQRRMMIPSFTLKRAGGLRPAIQRIVDDLLDTMIEQGPPADLVS

AFALPVPSMVICDVLGVPYADHEFFEEQSRRLLRGPTAEDSQGARDRLEE

YLGGLVDAKARQSEPGDGVLDDLVHQQLGQGALERADVVSLAVILLVAGH

ETTANMISLGTFTLLQNPGRLAELRADPALLPAAVEELMRVLSIVDALAR

VALEDIEIDGTTIRAGEGVFFSTSVINRDPGQYDDPDALDLHRPTRHHVS

FGFGIHQCLGQNLARAELEIALGTLLARLPGLRLAAPAEEIPFKPGDTIQ

GMLELPVTW

>CYP102B(2525014978)Streptomyces sp. PsTaAH-130

MAQTTESARTTGATGDPQRPKGFRSAELGWPELDRVPHPPRRLPLLGDVV

GVDRRKPVQDSMRFARELGPVFRRRIFNKEIVVVWGAGPAADLADESRFA

KHVGLGVANLRPVAGDGLFTAYNHEPNWQLAHDVLVPGFSRDAMEGYHGM

MLAVAGRLTDRWDRELAAGRPVDVPADMTKLTLETIARAGFGHDFGSFER

DRPHPFVSAMIGTLTYAQRLNSVPGPLAPLLLRGAARRNAADMAYLNRTV

DALVEERRGSDGGAGDLLDRMLATEHPSTGERLSARNVRRQVITFLVAGH

ETTSGALSFALYYLARHPDIAAKARAEVDRVWGDTGRPGYEQVAKLRYVR

RVLDESLRLWPTAPAFAREARRDTVLAGDHPMLRGAWALVLTPMLHRDPA

VWGENAEDFDPDRFTPQAVRTRAPHTFKPFGTGLRACIGRQFALHEATLV

LALLLRRYDLHADPAYRLEVTERLTLMPEDFRLRLSRRATPERTARPDAA

PERANGEAPGGGCPAHAEGAAGSGS

>CYP107NB1(2525015075)Streptomyces sp. PsTaAH-130

VASHVGDRNYPPIFGDDFARDPYRPLAALRARAPAHKVLTPDGTGLWITT

GYQDTRAVLDDPTMGKDADGLRAALKRPGTVHEIDRMIAFTDPPEHTRLR

RLVVRAFTTARVAALRPRIESVAERLVHRMRRADGPADLIADFAVPLPFT

VICELLGVPDEDRPGFRAAWSRLRAAASGTTAYTAAADGMAEVLFRIVRA

KRGGGTDDLLAALVNAPGDGERLTETELVAMAYALLVAGHETTANLIGNG

MFCLLTHPQQYDLIRTDAGLVPGAVHELLRYESPVYLATHRFTTVPVQLG

HVTVPAGEVVLAGLGPANRDPRRFPDPDRLDITRPSGGHLSFGHGIHRCL

GAPLALLEAEVALTVLPALRLAVPADRLDWHTTTVFRGLNHLPVTLAT

>CYP125A(2525015408)Streptomyces sp. PsTaAH-130

MHCPALPDGFDFTDPDLLHHRVPLPEFAELRRTEPVRWIPQPHGIAGFAD

AGYWAVTRHADVKYVSTHPELFSSFLNTAVIRFDEHISRDAIDVQRLILL

NMDPPEHTRVRQIVQRGFTPRSIRALEDRLRARAEAIVGAARSRTGPFDF

VTEVACELPLQAIAELIGVPQEDRAKIFDWSNKMIAYDDPEYAITEEIGA

RSAAEIISYAMNMAAERKRCPARDIVTTLVAAEDEGNLNSDEFGFFVLML

AVAGNETTRNAITHGMHAFLTHPEQWDLFKRERPATAAEEIVRWATPVNS

FQRTATQDTELGGTPIRKGDRVGLFYASANHDPAVFTDPESFDITRDPNP

HLGFGGGGPHYCLGKSLAVLEIDLIFHALADAMPDLRLVDAPRRLRSAWI

NGIKQLQVTTG

>CYP159A(2525016206)Streptomyces sp. PsTaAH-130

MSTARQVPDILSPAFAADPYPAYRLMRDTAPLLWHEATRSYLVSRYEDVE

RVFKDKDGEFTTGNYDWQLEPVHGRTILQLGGREHAVRRALVAPAFRGAD

LREKFLPVIERNSRELIDAFRHSGSADLVGDYATRFPVQVIADMLGLDRA

DHARFHRWYTSVIAFLGNLSGDPEVAAAGERTRAEFAAYMLPVIRDRREH

PGEDLLSTLCAAEVDGVRMSDEDIKAFCSLLLAAGGETTDKAVAGIFANL

LRHPGQLAAVRADRGLIPRAFAETLRYTPPVHMIMRQCATEVTLSGGTVP

AGATVTCLIGAANRDESRYRDPDRFDLFRDDLTTATAFSAAADHVAFALG

RHFCVGALLARAEVETGVGQLLDAMPDMRLADGFEPVEEGVFTRGPRSLP

VRFTPVSVL

>CYP113K(2525016221)Streptomyces sp. PsTaAH-130

VISRQPTDGQLLLEELPDRWRGLREAGPVRHDEVQGTWQVLDHENVAAVL

ADPATYSSDLSALSPTQADFETFTQGNFVGMDPPEHRKLRTLVSQAFTPR

VVQGLEPRIEAICARLLDGVADHDRFDLVDTLAYPLPIIVIAELLGIPAE

EHRLFQEWASVLFGGDQLGEAPDMADLERALEAIAPTVREMNSYMLDYIR

ARRAAPGDDLTSRLIAAEVDGVRLRDQEMVGFVALLLVAGHITTTALLGN

AIVTFDRYPDTDAALRADPLRIPTAVEEVLRWLPPFPELGRRVTRPVVLG

GHEIPADTLLMAHLGAANRDPARFDAPDVFDVTRSPNPHLTFGHGIHFCF

GAPLARLEARIALRMLHERFRMLAVPSHKDITYQNPAVIVGVRHLPVEVG

RA

>CYP105P(2525016250)Streptomyces sp. PsTaAH-130

MSEPTGAAPAVPKARSCPFLPPDGIAEVRAAAPVTRATFTSGHEAWLVSG

YEEVRTLLRDPSFSVQVPHALHTQDGIVTQKPGRGSLLWQDEPEHTADRK

LLAKEFTVRRMQALRPNIQRIVDERLDAIAAQGGTVDLVKTFANPVPAMV

ISDLFGVPVERRPEFQEIAEAMMRVDQDAAATEAAGMRLGGLLYQLVQER

RSSPGDDLISALTTTEDPDGVLDDMFLMNAAGTLLIAAHDTTACMIGLGA

ALLLDRPDQLALLREDPSLVGNAVEELLRYLTIGQFGGERVATRDVELGG

VRIAAGEQVVAHVLAADFDPAFVEDPERFDITRRPAPHLAFGFGAHQCIG

QQLARIELQIAFESLFRRFPTLRLAKPVEELRFRDDMVFYGVHELPVTW

>CYP105D(2525016251)Streptomyces sp. PsTaAH-130

MTETDIRHTGSEAPAFPQDRTCPYQPPQAYTEWRGESPLTRVTLFDGRPA

WLITGHAEGRALLADPRLSSDWGHPVFPVVVQRTEDRGGLAFPLIGVDDP

LHARQRRMLIPSFGVKRMNAIRPSLQSLVERLLDDMLAKGPVVDLVSAFA

LPVPSMAICELLGVPYDDHDFFEECSRDFVGAATSGDADAAFAKLYQYLH

GLVAKKQAEPGDGLLDELIARQLEEGGLDHNEVVMIALVLLVAGHETTVN

AIALGALTLMQHPEQIEVLLNDPAAVPGVVEELLRFTSVSDYMVRMAKED

IEVGGTTIRTGEAVLVSITLMNRDAKAYDDPDVFDARRNARHHVGFGHGI

HQCLGQNLARAELEIALGALFTRIPGLRLAVPLDEVPLKAGHDAQGPIEL

PVTW

>CYP107P(2525016469)Streptomyces sp. PsTaAH-130

MAGSSALAFDPWDPAFVADPYPAYAELRARGRVVRYAPTDQWLVPHHADV

SALLRDRRLGRTYQHRFSHEEFGKSAPPAAHEPFHTLNDHGMLDLEPPDH

TRIRRLVSKAFTPRTVERLKPYVQGLADELVAGVVRDGGGDLLADVAEPL

PVAVITEMLGIPEADRGQLRPWSAAICGMYELNPSEDTAARAVRASAEFS

DYLRELIAERRRRPGDDLISGLIAAQDGGDGAGSRLTEQEVISTAVLLLN

AGHEATVNATVNGWWALFRNPGQLAALRADHSLIPTAIEELMRYDTPLQL

FERWVLDDIEIDGTVIPRGAEIALLFGSANHDPAVFPDPARLDLSRADNP

HISFSAGIHYCIGAPLARLELTASMTSLLHRAPTLTLSNEPTRKPNFVIR

GLEGLRVEVR

>CYP1469D1(2525016917)Streptomyces sp. PsTaAH-130

MTATTPTPPQEPALPLSYPFPLGENGQLPPLIRWAQANQPVCPVAMPSGA

RMWMLTRKDDIARIFADPRFSRDLDDPATPRIAGQDITTVGRGLFSLDPP

DHTRVKSVVSGFFTRRAVGRYETLVRSHAAELLDAMDDGPNPCDLVAGYT

SLLQMLVMREVLGVPEDLWEDHRRVFPSASSITAGPQETATETEEIKAFT

ARVIAARRRLPEREDPLGALLRAAADGTISEEEMFGTAFLLFFTGSDGII

APTTTGAMILMLNPSQLRPVLDDPGLWPKAAEEVLRYFHNGVLGFPVVAR

EDVELHGVTIAAGDAVVASMQAATWDPRHVNNPEKFNVRRREDAAATFGA

GPHYCLGAQLARLYLATALRALFERHPSLRLAVAEQDIPWRNDIMFVRPV

ALPVTWSEGTPA

>CYP154A(2525017515)Streptomyces sp. PsTaAH-130

VTEEQPLVLDPTGSDHHAEHRALYARGAATPVDILGVTAWSVSDPDLLKQ

LLTRASVSKDARAHWPAFAATVPTWPLALWVAVDNMFTAYGGDHRRLRRL

IAPAFSARRIAALTPVVEALVTGILDELAALPAGEVVDLRERLAYPLPIA

VIGRMMGLSAARSSGLRTVVDRVFATTLSAEEATANTAALYGLFDELIAE

KRSSPGDDMTSLLIATRDEEGDGGCLTDAELRGTLVLMISAGYETTVNVI

DHAVTSLLTDPAQLAHVDSGRCGWDDVVEETLRHESAIKHLPMRFAVQDI

PLPDGRTIRAGEAILASYAAANRHPDRHGADADRFDATRTTKDHLAFGHG

VHFCLGAPLARLEVAAALRQLFTRFPDVRLAVPAEELRPLPTLISNGHTS

LPVRLRPVPAGGRAGT

>CYP1253B3(2525017569)Streptomyces sp. PsTaAH-130

MFTAEGKEDPYPALAELRRIAPVYYHEKLDTYFLTRFADCQQVLSDASCL

TPDLTWCETELPHWREHPAAVFFYSSLLRANGTDHVRLRRLVADRFSARR

VAVLAERIETTTAALLDRFADATRDGGAADFQELVGYPLPVAVVGDLIGV

PHTDQDRFHHYGQDASRLLEPVRTDEDWKRADEAVTALRGYFAQLVALRR

RRPADDLVSALLAVREADDGRLAESELLDMLLLVFVAGFETTTSLLGLTV

HALLSHPGQLALLREDPALVPNGVEESLRWDTPVRMTERIATRPLTVGGV

AIPQGGNITTVLAAAGRDPARHPDPDAFDVLRPDIRVLSFSAGAHYCLGA

ALARLEGAAVVRQLLTRFPRLAHARRPVRRDSISLRAFTRLPLVTSG

>CYP158A(2525017615)Streptomyces sp. PsTaAH-130

MPAATPAGGPRTLRLEQSRTTPAAPRPIVEGMTTMETSAPTGQEPPPVRD

WPALDLDGTEFDPVLAGLVREGPLTRIRLPFGEGWAWLATRYEHVKLITN

DPRFSRAEVARGRVTRLAPHFAPRPGSLAWADQPDHNRLRKSVAGAFTVG

AMKRLRPRAQEILDELVDAMVREGPPADLVERVLEPFPLYVVSEVMGVPE

TDRDQVHEWTRQIISLCGGAEAADRAKNGLYGWITEIVRARARSAGEDVY

SLLGGAVARGEIGEEEAVGLAGPLQIGGEAVTHNCGQMLYLMLTRPELMA

RVRARPGERGPVLDELLRWIPHRSTVGLARIALEDVEIAGHRIAAGEAVY

VSYLAANRDPEVFPDPDGIAPDRDPNPHVAFGNGHHYCTGAVLARLQLEL

LVDALLDRLPGLRLAVPPEEVRWRRQTMIRGPRTLPATW

>CYP147B(2525017641)Streptomyces sp. PsTaAH-130

MDPGTLLARITDYASRPDPYPLYAELREAGAVVPQADGSLLIGGYHEVAA

LLHDPRMSADPRTRGVQTAKPPFLRLDDPEHHRLRTLAMRPFGPPHTPHR

VDGMRAEIENITKELLAPFESGGRVDVVDDFAYPLPVTVICRLLGVPHED

EPLFRAWSDTLVASADVRPDASSAPRDDAERARQEMGGYLVGLAEQRRDD

PRDDLLSAFVSEPDPELRLSREELAETAVLLLIAGHETTVNLITNGVLTL

LRHPEELDRLRREPDLMPRAVEELLRYEPPVHMRERIPLADFDVAGTTLP

RGSSVFLALAAGNRDPRRFTDPDRFDPTRADNEHLGFGSGIHLCYGAPLA

RLEAQYALTALLPRLGTAELAEDPPPYRQNAMLRGPRHLTLRF

>CYP163B(2525017676)Streptomyces sp. PsTaAH-130

MVRAGAPAPATVDLGDPDTFADHDLDGFWRTLRDTAPVYWNPPAGGRRGF

WVLSRYDDIMAAYRDDLHLTSERGNVLVTLLGGGDAGAGRMLAVTDGHRH

HELRKILQRVLSPRVLSEVAAAVRANTRRLIREAVEAGGCDFAEEIASRI

PMTTISNLLGVPEQDRDYLLAQTKAALSADTEDVDEVDSEMARNEILLYF

MDMVEERREAPGDDVISMLVASSIDGVPLSDEDIVLNCYSLIIGGDETSR

LTMIDGVHSLAARPGQWRRLKDGEVAIDTAVDEVLRWASPTLHFGRTVVR

ETEVHGVRLRPGEIVTLWHASGNRDERVFERPGEFDLGRTPNKHLAFGYG

PHFCLGSYLAKVEIAELLMALRDFTTGFEQTGEALRIRSNFLTGYSTLPV

RLWPDPRAMKEAAAQ

>CYP105B(2525018149)Streptomyces sp. PsTaAH-130

MTETPTAPPAPTPAPASTAPPAYAAPRAPGCPFDPPPERHRLLAEGPLAR

ISLWDGNGAWLVTRHADQVALLRDERISADNRLPNFPSVSAGSQATRAHS

RTFISMDEPEHNEQRRRFTADFTVKRINELRPRVEAIVTGLLDAMEQAGP

PADLVRAFALPLPSLVICELLGVPYADHAFFQRVSAVMIDTRSTPEVALA

ASQELVDYLGDLVVAKERDPGDDLLSRMAVQYLRTGISTRDECAKQARLL

LVAGHETTANMIALGVCALLQHPEQLAAVRDGEPRRVVNAVEELLRYLTI

VHLGRRRVAVADVEVGGQVIRAGEGVVFATDLANRDPEVFEDPDRLDVDR

AARRHIAFGTGPHQCLGQNLARMELQVAYPALLRRFPGLRLERPLAEIPF

REDMAVYGVYELPVTW

>CYP105B(2525018179)Streptomyces sp. PsTaAH-130

MTVESVQPETSVAPAQLREPPLAGGAVPVFGHGLKLVRDPLDFMSSLRAH

GDIVRLRLGPKTVYAVTTPELTGALALSPDFKIDGPVWESLEGLLGKEGV

ATANGPLHRRQRRTMQPAFRMDAIPDYGPIMEEEAHALTERWRPGTPIDC

TSESFRVAVRMAARCLLRGEFMDERAERLSTDLATVFRGMYRRMVVPLGP

LYKLPFPANREFNRALADLHLLVDEIVAERRASGQKPDDLLTALLEAKDD

NGEPIGEQEIHDQVVAIITPGAETIASTIMWLLQALAEHPEHAEKVRAEV

ESVTGGRPVGFEHVRSLRHTNNVILEAIRLRPAVWILTRRATADTALGEY

RIPAGADIIYSPYAIQRDARSYDRHLDFDPDRWLPERAKEVPKYAMSPFS

VGNRKCPSDHFSMAQLSLITAAITAKYRFEPADDCDDSVRVGITLRPDNL

RLRPLPW

>CYP1207A(2525018464)Streptomyces sp. PsTaAH-130

MTTATPTSTATAPAPVFTERYLLSPDMIADPYGHLDALRAHAPVHWSPMH

HAWLVTGYDQVMRCLRDPAVTADRVRPLMDAVPQGAREDAERAFGILSRW

MVFNDPPQHRRLRQVFQEAFAARAVARYRTFTEKATRAVLARKAVPGRTG

DLMADVAKPLPALVFARWLGIPRTDAAAFWYWNARVADLVLGTAQEESEY

RASLQALVSLEDYLAGLVARRRAEPADDLISQVLKEGRVGESVTEEEFVG

MLTQMAFAGGETTSNLIANTVLALHTRPGQLAAVREDPALAQGAVEEALR

LDGPSKMSIRAAVSGLELDGHTLRAGDRIFLVTAAANRDPARFPDPGRFD

VRRTGATHLGFGFGAHFCIGAALARLVAVAAVETLVRDHPGLTLTDPGAL

TWQPSLLNRSLTALPVRY

>CYP157C(2525018669)Streptomyces sp. PsTaAH-130

VTPEHQPPTATFATGTPDPLSGPPPGCPAHGLGPGGLHRLYGAGAEDLDD

LYERLREQYGPVAPVLLHDDVPMWVVLGHAENLQVVRSPSQYTRDSRIWA

PLLDGRVKQDHPLMPHIAWQPICSHAEGDEHLRLRNAVTSAMATIDHRSV

RRHIGGHTQHLVNGFCERGRADLVAQFAEHLPMAVLCEILGMPEEYNDRM

VQAARDALKGTETAIQSHAYVMDALNRLTLRRRARPEDDFTSHLVLHEAG

LSDDEVREHLRLVLFAAYEATANLLANALRMVLTEPGFRARLNGGQMTVP

EAIEQSLWDEPPFSTVLGYYAKQDTELGGQRITKGDGILFAPAPGNIDPR

VRPDLAASMQGNRSHLAFGGGPHECPGQDIGRAIADVGVDALLTRLPDVQ

LACAEDDLRWRSAIASRHLVSLPVRFEPKPQQDTDMPPRAYGDPGLPADL

RLPVQRRESQGAALPAAVPPPRPAPRPPVPAAAPARGPWQRLLRWWRGE

>CYP147F(2525018839)Streptomyces sp. PsTaAH-130

MTRDSIVRRITDFANRADPYPVYRELRATPVLHEEEGGPYVISSYWDIEA

LLHDPRISSEAANLADPGEDAALLQSEETGGLPPSFLRLDPPEHDRLRRI

ANSAFGPPHRPRRIDGMRGELREIVTALIDGFGDARGIDLVDDFAYPFPV

TVICRLLGVPREDEPRFRAWVDPLVASLDPGKRQEADEEFARTAREARMQ

LGMYLAGLAEERAKEPRDDLLSDLVNSHGPDGAMTMMEVLSTSVLLLIAG

HETTVNLITNGMLTLLRHPDVLERLRREPELAPRLVEELLRYEPPVQLVP

QRTCITDIEVRGVTIPKGSRIWLVLAAGNRDPERFKDPERFDPDREDIQH

LGFGSGIHICFGAPLARLETQIALTELARRLENPRLVQDPPPYRENAVLR

GPRHLEITFDGVRP

>CYP158A(2525018902)Streptomyces sp. PsTaAH-130

MTETEEATLAEAVPPVRHWPALDLTGTDFDPVLAELMREGPVTRIQLPNG

EGWAWLVTRHDDVRMVTNDPRFGREAVMDKPVTRLAPHFIPDRGAVGFLD

PPDHTRLRRSVTAAFTARGVERVREKARGMLDEQIDQLLRDGPPADLSAA

ILSPFPIAVICELMGVPAADRHDMHEWTQLILSSAHGKEVSERAKREMSA

YFSGLIGARGGSTAEDVTSLLGAAVSAGEVTLEEAVGLAVLLQIGGEAVT

NNSGQMFYLLLTRPELAERLRREPEIRPKAIDELLRYIPHRNAVGLSRIA

LEDVEIRGVRVRAGDAVYVSYLAANRDPEVFPDPEEIDFSRCPNPHVSFG

FGPHYCPGGQLARLESELLVDALLDRIPNLQLAVPAEEVPFKKGALIRGP

EALPVMW

>CYP105B(2525018950)Streptomyces sp. PsTaAH-130

MPLRPAPALKELQSERPVSRVRIWDGSSPWMITRHEDVRALLGDPRISAD

PRRPGYPHRSAATQERDRSRSTFLTMDDPEHARLRRMVTAPFTIKKVEAL

RPSVQRIVDDLIDKMLAGPHPTDLVEAFALPLPSLVICALLGVPYEQHDF

FQRHSRVLVRRTSTREELLAASEALTDYLDGLLAAKLANPEDDVLSQLAV

QRVATGELTRRQAAEVGVLLLVAGHETTANMIALGTVALLRNPDQLALLR

GSDDPKTVASAVEELLRYLNIVHSGRRRVALEDIEVGGELIRAGDGVIFA

NDIANRDPGLFPDPDRLDLTRDARRHVAFGYGIHQCLGQPLARVELQVAY

STLYSRVPTLVLATDFDQLEFKHDGFVYGVYELPVSW

>CYP107U(2525019458)Streptomyces sp. PsTaAH-130

VTDQLPPDDTVPAAPELFTWEFAADPYPAYAWLREHAPVRRTRLPSGVEA

WLVTRYADARQALADQRLSKNPAHHDEPEHARGKTGIPGERKAELMTHLL

NIDPPDHTRLRRLVSKAFTPRRVAEFADRVQELTDDLIDRFAERGSADLI

HEFAFPLPIYAICDLLGVPREDQDDFRDWAGMMIRHGGGPRGGVARSVKK

MRGYLAELIHKKREALPAEPAPGEDLISGLIRASDHGEHLTENEAAAMAF

ILLFAGFETTVNLIGNGTYALLTHPEQRRRLQEALARGDEELLATGVEEL

LRYDGPVELATWRFATEPLTVGGQDIAPGDPVLVVLAAADRDPERFEDPD

TLDLGRRDNQHLGYGHGIHYCLGAPLARLEGQTALATLLTRLPDLRLAAE

PDELRWRGGLIMRGLRTLPVEFTPPAR

>CYP178B(2525020818)Streptomyces sp. PsTaAH-130

MHVVEDSPLYSPEFFADGDPWAYLADLRAHHPVSVHRREDGYEFHALTRY

ADIYAAYVDHQRLSSSYGTMVDGSYLPQKDSASGRMLIVTDQPAHTGIRK

PIKTSGFSRDMLAKVGRTVRRNIRDTLGELSVGDRLDFGSVVAPELPKGV

LEVLFGIGAGDAAKLLEDTRTMIGYRDEVYAGTSPLDALVDAQLDVLEFI

DELIGQRLDSGQADDMIGFLARCVADGTMPRDVAVLNGLNVAVGGNETTP

HTASLTVHTIDGHRDQWRTVADGDTGCDVATQEFLRWTSTNSYVQRLAVE

DFPVGGHVIPAGSFVTLWNMSANRDADVFDRPDEFLIDRADNKQLAFGAG

VHRCVGAPAATLEIQTFVEELAAWDKAFAVLEEPRKLRSNFMLGLTELQV

EVVDAKEFHQ

>CYP1035A(2525020922)Streptomyces sp. PsTaAH-130

MAASLPMLNPPDHTMTRKSLGNIFDRHSLARLRPFIERTADRLTERFFEE

AREGSGEFCSLVADELPIITIGQWMGLPEADHEFLRTLTHDQVHTQELFP

SRSQLALSDAATAQLRQYFTDVIRERRNDPGEDPISSWLRAWDGIEPDRA

RADAAVHSLALFMILAALETTSHVLSASVRLLVERPRTWEWLRRHPEDVP

GAVEETLRYDAPIHMISRVAPEDCEVGGVLVREGEMVQLMTGAAHHDPEQ

YADPHVFDIHRAQAHLSFGGGIHYCLGNALARLEATALLNSLLKRDARLH

ISAPPRWEPRIAFRRMTSLQLALG

>CYP206D(2525020939)Streptomyces sp. PsTaAH-130

VSPTKTLADPIDVMGRLLSFEGKQDPYPLYEQMRAHGPVVDVGGAHLFVT

GHAECARALREADLLSTDAAVQDVRLPGWREHASWSWLTKNMLFSNDPDH

ERYRRFFSSAFSARGVEAWRPLVERRAARAVERVARLAADGREVDLVAEF

SFPMAAGVIGELLGIPDEDHDSFRGDVGDITLALEPIRDMGQLTAGDAAM

ERLAGYFHDLVARRRAQPTTDLTSAFTAARDAGGELSETELVANLMLLLV

AATEAPQDLLSNMVRLALEHPAEGERLRTEADFAAGFTDETLRFEPAAQI

LNRVASRDLDFFGVKVAKGVPLTLLIAAGNRDPRRFTDPGVFDPARTGNS

PLTFSGGAHFCLGAALARMSAETAVPLLLRRLPALKLAGAATFRDQIVQR

GHGRLPVTAG

>CYP124N1(2525020975)Streptomyces sp. PsTaAH-130

LDTHHDDTTTGDPLRAEFWKQPADEIEAFFARLRAGRKPVFLPYRTSYGF

HALTRHAQITEVSRRPDLFGSAPSSSTLEDRPPAFGESAEGSLLHMDAPR

HTAMRRVVARAFNPSGLRHHDDMIAGAARRVVDELLEKTPCDFVTEAALQ

LPLRAICAIVGVPASAYEDVVRATDAITRIANTTDRTGQGAVAEAVTYFS

TLMGDLARLRRESPADDVTTTLVHATVDGRPLTDAELGAFLRILLVGGND

TTRSTLAHMLHLLTDNPGQKRLLLADLPAHLPGAVEETLRHATPGTWMRR

NVTRDTTVDGHPLNAGDRVILYYNSANRDEDVFERPHAFDIGRAPNPHLS

FGGPSPHHCLGAHLARRETSLFYRELLTRAPGIHATAPPAHTYSGLIHGI

GHLPCAR

>CYP107MK1(2524930963)Streptomyces sp. CNT318

MTTLTGPGREVLDMSFFQDPYAVYDRLRVEGPVHAMRTPDGIDVWVITRY

EEAKEALSDARFTKDFSGVPDIFAQQSTGVSAHEGYTDILTKHMLFADPP

DHTRLRELAAKAFTRRRVEALRPEIQQIVDGLLDTISAAGEVDLLRSLSF

PMSMAVTCRLLGIPPADEDAFRTWTETIFTTNDPARMNEAVRGVDQYLVD

VIEAKRADPADDLITALINAREDGDRLSDRELVSTCALMFVAGYETTASM

VGNAVLALLKNPDKLAELKADPSLMPNAVDELLRFDGPVNVATLRSTATD

VTVAGTTIPKGQFVLVSLIAANRDGEKFPEPARLDFTRPLGGSLAFGHGI

HYCLGAPLSRVEIEVALSRLLARFPDLNLAREPETLQWRNSLLVRGLSTL

PIRLH

>CYP158B2(2524931129)Streptomyces sp. CNT318

MAQCPLYDVPADPPELEFDPFLRAALESPPSRIRLPHGEGDCWLVTRYDD

VRFVTSDPRFSRDIVGRPTPRMTRHLIPLDRAVSFVDPPGHARVRSVVAP

VFGSGAVERLRPRVRALVAELVDGMLAAGPPADVVRYVVSPLPLAVVGEL

LGVPPEDRTQVRDWAVTLLTRASDDAAAERAQQVKAAARRYFRELAERRR

SRPAGDLMTRMVAAVDAGRIDEEELLALATLIGLNGWHAVRNNVSNMVYL

LLTRPELRERLRAEPESVPRAVEELLRWIPHKHGVGQPRIATEDVEVGGV

LVRRGEVVQVSYVAAGRDPRRFPDPDALDIDRQGPPHLAFGHGPHHCVAP

LLARMEAEELLSALLRRLPGLRPAVPAGEIAWQGSVLIRGPVGLPVTW

>CYP107L(2524931454)Streptomyces sp. CNT318

MADRSVLDLSPDTDGIVDLAVLGDDFVRDPYPVYAALRRRGTVHHVRTPE

GALGWLVVGHDAARAALNDPRLSKDWANASPEAGTLSISPGTHMLISDPP

DHTRLRKLVSRQFTPRRIAALEPRIRELTAGLLDRMLAHPDGRADLVDSL

AFPLPIAVICELLGVPDLERERFSAWSDHVLSAAPEEEKRGSAEALGGYL

TELLERQRARPGEDLLSLLIRTSDEDGDRLSTGELHGMAWLLLVAGYETT

VGLIANGALALLCHPDQLAELRADPALLDGAVEEMLRYDGPVETSTYRFT

REPLEIDGTLIPGDGRLVLPVLADADRDPARFPEGDRFDIHRESGGHLAF

GHGIHFCLGAPLARLEARIAFRMLLERTERLELDASPAALEWRQGTVLRG

VKSVPVRFG

>CYP154C(2524931723)Streptomyces sp. CNT318

MTPCPVTGTGPDSGAGRAAATDPVIVLDPLVRDLAGEGARLRAAGPLAPV

ELPGGVRVWAVTRHAAARKLLTDSRLVKNIEHWAAYRRGEIPPTWPLIGL

ADPGPSMLTFDGPEHRRLRTLTAQALTPRRVAALRPRIEEITHTLLDGLE

AAADAEGRVDLKSAFAYPLPMAVIGDLLGIDTTRIPRLRTLFDGFFSSVT

PAEEVPAIIAELGSLFGAEVARKRESPGDDLTSALLAAAEDGDSLSDEEV

VATVQVLITAGHETTISLLVSAVRALLTRPEQLALLRSGEVGWEAAVEET

LRWDAPTTHVLIRFATEDLEVGGTVVARGDAVIISYGAIGRDEEQHGPDA

ERFDLTRTPTRHLSFGHGPHVCPGAPLSRLEALVALPALFDRFPGLRPAV

DPAELRNKPAITQNELHELPVRLR

>CYP157A(2524931724)Streptomyces sp. CNT318

VTPPQHPGAASAPPPGCPARPDSGAVPLLGPRFQNDDRARLYREMYQQHG

PLAPVLLPGDIPCWLVLGYRELHRVTSEPALFSRDSGLWNQWPNIPPDWP

LLPMVGKQPSILYTVGERHRRRAALVGDALAAVDPFELRNHAERFADRLI

DGICGRGSADLIAEYAKVLPALVLARLFGFSDEAGAELVPSINALVDGGP

DALRGRDRVQAAMHSLLTARRTAPGAGVASRMLRHGHAPEFTFEEILEDM

VVNIVAGHQPTADWIGNSLRLMLTDERFAASLSGGRHSVAEAMNEVLWED

TPTQNIAGRWTTRDTRLAGRHIPAGDLLMLSFAAANGDPRIRPDRTVLTG

GNSAFLSFGHGEHRCPHPAQDLAEGIARTGIEVLLDRLPDLDLAVSPQAL

VWRASPFLRGLSALPARFTPTPTSTTGGPR

>CYP183A(2524932028)Streptomyces sp. CNT318

MTEESTFSAGTAPGAFPVVGHALQMMRHPVNFMTSLSAHGDLVEIKIGPT

RAYVPTHPELLRHVLTNDRIFDKGGVFYDRARDIAGNGLVTCPFADHRRQ

RRLMQSAFTRGQLKRYSEAMHAEIEATASRWQDGMVVDAFQEMYGMALRT

VGRTLYSTPVSPELAAQVERSFDVVLNGLFRQMFLPASVRRIPLPSNRRY

RSNLDFLHATTQRLIDDYRADGTERDDLLAALLASRDDDGGRLADREIHD

QVITVMAAGTETVAGTLTWVFYLLSQHPEIEAALYEEMDTVLEGRAPQWD

DLPKLSLTDRIISETLRLHPPAWLFTRLTAAPTELAGRRLPAGSTVVFSP

AAVAQYADAFDDPATFDPDRWLPDRVAPASRHAYVPFGTGARKCIGDLYA

RTEAALGLATILGRWRVTCEPGMDIRPVPLATVYHPRRLRLRLDARTPRR

TAAAVPAPAGGDPT

>CYP161C(2524932036)Streptomyces sp. CNT318

MEELPTLPFDNPSVLGVAPRMRTLQQEGPITPVRAAGEDAWLVTRYDEVR

ALLADRRLGLSNPYPARQAKTTARNTMMALMAGDDYETEAADHPQLRELL

VPRFSTRRMRMMKSRIEQHVDELLDQLAAGPQPADLHRALSFPLPTMVVC

DLLGVPWADRERFGQWARGTFDQSDDGRHSAHTFQQVVEYTTELVARKRT

EPGDDILSELIAAKDGSLSDAYIAQLGNAVLLFGYETTIVRIDLGTLLML

RNPEQRALLADKPELAPGAVEEILRLAVGGKGSNALVPRYAHSDITVGDT

VIRTGEAVLLAIGAANVDGHAYPEPDLMDITRDRPRPHLSFGHGTRHCVG

RVLARIELTAVFERLFRRLPELRLAVPEEELRWQEHRITGGFDEIPVTF

>CYP1237A4(2524932125)Streptomyces sp. CNT318

VSTQTPPRYPFAWTPPMQVPEALRHVHSSSAMEVTLPSGDVATLVTRYKD

VRALFADKRLSRNIARPDCARISADNDLFMDPEIDPDPPKHTQVRSLVTK

AFTARRIEALRPYVQRVADELLDEMAAGPRPVELNEALAFPLPIMVICKL

LGVPAEDRDQFREYVDGFLSVTKLPPEEVGQCRQNLWKYLGDLIDAKREK

PGDDLVSELIRVRDEEDNRLNDHELHFWCQGLLIAGYVTTASQIGTGTAV

LLHHPELVREIQADWSLVPSTVEELLRTQIMGSSVGTMRYAVDDIPLSDG

SVIKKGTSVLLSEEGANMDPEVFDQPFELDIRRQENHHMTFGAGIHYCVG

AALARMELQVATESLLRRFPDIRLAAPAEELPRALGGFMEGFTEIPVEW

>CYP107AL(2524932182)Streptomyces sp. CNT318

MAQDSATPTPVRPYPPPRRSATEVPEDYALLRAEEPVAAVTLPSGDPGHL

VSRYDDVRALLADPRCSRAATVAPEAPKLTAVPFDAGGLFTMDPPEHTRL

RGLVARAFTPRRVERMRPRLEELAGELADAMAAGNGAGGGPTDLNTAFAF

PFPMAVICELLGVPFADRERFRTWSDAVLSLTAHPPEEMLRHKQALLAYL

ADLVADKRRAPGTDLLSALVGVRDEEGRPDERELLVLAMTLLVAGHETTA

GVLGTSVLTLLRQPERLGLVPDGEEATAALVEELLRLNPVGDGGPLRVTT

EPVEVAGRVLPPNSAVIASVCSANRDGSRYTDPDRLDPDRTGDPAQPPHL

AFGHGPHYCLGAPLARAELAVALRVLAGRFPALRPAVPVESVTMHRGLLV

NRLTELPVRWD

>CYP154C(2524932230)Streptomyces sp. CNT318

MNGDAQSPASEPRTAGAAPAGCPAADGGDAPGTAGCPLTIDPLVGSLAAE

TEALRTAGPLARIDLMGAEVWTVTTHAEARRLLTDTRLVKDITRWNLWRD

GEIDENWPLIGMVDAGRSMFTVDGAEHRRLRVKTAQALTPRRLEELRPVV

ERVTARLLDAMASDAAEAPDEPIDLKSAFAQPLPMSVVCSLMGVDPALEP

RLHHLYEAFFSTLTPQQERLAVLRELDELYHDMVRHKTASPGDDLTSALI

LADEGGEPLTAEEVRGNLEAMVAAGHETTVTLILCAVRALLTHPDQLRLV

LDGQIGWDAVIEETLRWESPSTHLLLRFATSDIEVGTGGAVIREGEGVAM

SYRAIGRDRAQHGQDADRFDVRRPAPIRHLAFGHGPHICPGAGLSRLEAS

VALPALFERFPALSAVAAAETRHRPVLTQNELECLPVRLGVG

>CYP157A(2524932231)Streptomyces sp. CNT318

LTSQPQYYDPAHGPPASGAPPAEPRGCPAAAGGRAAPGAVPLMGPRFHTD

LEAVYRDMRRDHGPVVPVTLPGDVPAWLVIGYRELHHVTGDPVLFPRDQG

LWNQWKNLPEDWPLRPMIGTPQPSVYFTVGQEHRRHLAMVQHALEGVNHV

ELRGTTEEIADRLIDSFCGVGESEIVSAYAKPLPLLTLARILGFPDSDGP

ELIRSLTDMADGGPHALAGFEHALSLMRRLVARKHAAPGADVLSAMLAHP

AEFTDEAYVLNLMAVTSAGFLPTADWIGNSVRLMLTDDRFAASLSGGRHT

VPEAMNEVLWEDTPTQILAGRWAARDTRLAGRAVARGDLLLLGLAGANRD

PAAHAPHGADRPRERNFAHFAFSHGEYQCPFPAQEIAEVIARTGIEVLLD

RLPDLDLAVAPRTLARRPSPFLRGMTVLPVRFAPTPPYGGR

>CYP105N(2524932651)Streptomyces sp. CNT318

MRTPETSTPDRLRTDAGPNPEAPPRDFPVRRGCPFTAPAEYGALRTHDPV

ARVTLPTKKEAWVVTRYDDVRELLSDPRVSADIRRPDFPALGEGEQEAGA

RFRPFIRTDAPEHTKYRRMLLPVFTVRRVRAMRPAVQARVDEILDGMLAG

GGPVDFVSVYANAVSTSVICELLGIPRENLEFFRDVTRVSGSRTSTAEQV

SEALGGLFGLLAELVGKRREEPRDDLISKLVTEHLVPGHVTMDQLLSTLG

ITINAGRETTTSMIALSTLLLLDRPELMEELRRDPSLMPAAGEELLRVLS

VADSIPLRVAAEDIDLSGRTIPADDGVIALLAGANHDPEQFDDPERVDFH

RTDNHHVAFGYGVHQCIGQHLARLELEVALETLIRRVPTLRLAGDRDDVV

VKHDSATFGLEELRVIW

>CYP107E(2524932878)Streptomyces sp. CNT318

VTTHSRQVRDFPFETRARLSMEPLFAALRESEPLSRVRLPYGGEAWLVTR

YEDIRTVLGDPRFGRAGTLHERAPRIQPDPAGEGVLMSLDPPDHTRLRKT

VAGVFTKRRVEELRPGTERIARELLDAMEAAGPPADLVTSFALPLSVTVI

CDLLGVPREDREKLRSWSDALLSTTACTPAEMAASTEALADYFATLVRQR

RQEPAGDLLGALVDICDSRQGRLDEEELVLLTRDLLIAGHETTASQLANC

TYLLLQQPDAAARPDADGRMPMAAVEELLRFIPLGSGSFRARVATEEVEL

CGRRVRPGETVFAPTVAANWDPGVFSDPGTLDWERSPNPHLAFGHGVHHC

LGAQLARMELQVALGALLDRLPGLRPAVGEGEIEWKTGMQVRGPRTLPVR

W

>CYP157K(2524932885)Streptomyces sp. CNT318

MNAQPSDGTRLEAPLPPAGCPVGHTGAATRLYAAESTADPYATYSRLRKQ

YGPVAPVLLEGDVRAWLVLGYRENRRALDNPGQFSRDSRRWRDWQEGRIE

EMSPLIPMLGWRPDCLSQDGEPHQRLRGAVTDSMATVANRGIRRHVVHFA

HKQIDAFAHTGRADLVPQFSEHLPMLVLTRLFGLPESDGEQLVASCARMM

RGGDGAVEHNERIMRVLAGLTAHKRAEPGPDFATGLIEHPACLDDDEVQN

HLRLVLIAAHTTTSNLLARVLQAVLSSSSHLADLVSGQLTVTALVEETMW

NAPPLAVLPGRFATADCELGGQQVEAGDLLILGLGPGNLDPEIRPRTGTA

VRGNQAHLAFSSGPHECPGQAIGQAIIETAVEVLVHRLPGLRPAVDPAEL

TDTASTWETRLDHLPVTFPV

>CYP1251C1(2524933024)Streptomyces sp. CNT318

MTGAAAHTRRDPLYDPLAPEVIAAPHAAYRRLREHRRVYWHAQLDSWVLT

GHAECRRVLGDTAAFGSDFRRVGEAVPDAQLSVQSLDPPAHSAVRHLLVS

ALHVRSHTAVTDTAARLAAGRLDALRGAGSVDLVSGFARPLALHTMCDFL

GVAPPDGPGFEEMSNAIVRSMDAGLDPARAEPGTRARAELSRMVGEWLET

AGEDGFLGAARAARAQAPEVSAAVLANSLRAVLHAGYESVSRLLGNALAR

LVDDPALLGRAMARNALDPLVDELLRLDGPVQADARVCVADSELGGRRIR

PGEVVVLLLGAADRDPEVFADPDAVDLNRRRGAHLAFGRGAHACLGAGLA

TLQLRAVLSALHTAGIGFRAQGPAQYEPTATLRGLRALPVAVSEPAAAC

>CYP1192A(2524933685)Streptomyces sp. CNT318

MHLPERPAGYDRARHGVHLLRSPEVIADPSVYIDAIAELGPLFYDEVGSV

WVCSGYAQAVEILRDHSRFSSVREYDHAALRERGLHTSAALSAMVHEQML

FLDPPQHSAVRSTLAGQFSGKRIKSRESDLRAIADRALEGLPAEGVLDLV

DDFAAKLPATLVAYLLGMPGREDDLTRWAEAYERLLGNLSALQAPSAPQV

ERDLTEALSVLQQEAQDRLRAPGTDVISSLTTPWPTGRRAKKSCSPSPRT

ASSSSAAATRPCPTSSPRPCWPCTTTRPASRSCAHSPRGSRRPSPRSCGS

TDRVSTSRAGPPLT

>CYP1192A(2524933686)Streptomyces sp. CNT318

MRLNGSSQYVARRATADVTIHGTRIATGDNVLVHLAAANLDPQTFTAPGT

LDMDRSAPKHLGFGSGRHACPGAGYAERLAGFAIEGFLTKYPAYAPEPGP

DALSWGLHGNTRCLDHAHIRVPAAAPASAADPAAEVNTADHAVDEHARLD

GCSSLRPPSDPPPAHCWHEVFEQQARLTPEVPAVQGPHAAVTYRELDQRA

NALAHRLRHLGAQPGAVVAVVMERSVEFALAVLAVAKSGAAFLLADITCP

HERLRTMLTDADACLVISDGTVPASIFPAPVISTDAPDPRPDAPLTGVSA

GDSAYVVFTSGTTGAPKGIGISYEATVNLSLAQREIFKLCPGDRVLQFLS

PNFDGCFADLTLALLSGATLIVAPADQLTVGPPLVRLLKSQKVTTVILTP

SVWSALPDQPLPDLRIAAAAGERLPAAWVRRWAAPGRRLLNLYGPAETAV

LATWHECTPGEERPPIGRPVANKQVHLLDEALQRVAPGQEGELWLGGVGV

GRYLNQPDLMEERFIRDPHTTTGPSSLLYRTGDICRQRPDGALDYVGRRD

RQVKIRGQRLELDEVERVLESAPGVADCHVHENDGRLHALVVPTGTDLDE

ESVRTHLAGHLHSAMRPHTLTAVAELPRTQNDKADHSTTASAGSASHHSA

TAAPHRRHSRLTWQVAQHFAQSLDIPLRQVQADSDFFTSGGDSLTLAAFL

QRLESLTDAPVDTSALINAPTPEQIASLLLAEGTPA

>CYP107MH2(2524933697)Streptomyces sp. CNT318

MNDARTPRTRTPATVDLRPHAEALLRDPNPVYARLLEQGPVHRAVQPDGA

EAWLVLGYPEAKEALAHPALSSDPAQADRNWRARYLGDPDTTEFPHGRNM

LNSDPPEHTRLRKPTTQAFTPRRIEGLRSTVEEHTERLIRALPETKPFDV

IDDFAAPLTLSMICTVLGVPDLDHGRIREWADRVSFPKIPQDAITARQDL

IPYFDELIRTKRQEPGTGLFDALLETGDTDLLSHEELRATAFLLLLAGHE

TTISLLSNAVLCLLTHPEQLALLRRNPDLANQAVHETLRFAPPAPSPFPR

FAAEDLTLDSTEIPGDGSHVKIMISSAQRDPREFPEPDVFDISRPPGYLL

AFGHSIHGCPGRSLAHLEASIALPAFLERFPDLSLATTPDRLNWRVGPLL

RALDHLPCQAHAAERRPTKPTRAA

>CYP157C(2524933711)Streptomyces sp. CNT318

MTAPSSVPAPHAPGTPPPGCPAHAGGQPVGRRLYTEDAIRNPRAVYEELR

EQHGAVAPVLLHGDLPAWLVLGYRENLKALQTPSVFSRDSRYWRDMEEGR

VPSDHPLIPITAWQPLCVFVDGEKHRRLRGAVTESLDAFESRGIRLHVTQ

HTDRLVDEFAEAGQADLVGQFAAQLPLRVMTQLYGMSDEHGGEYGPAFIE

AVQDALRGTATSAASNDFVTRTLDDLVARKQDAPAHDLPSMLLAHPAGLS

TDEVREHLRLILTAANETTVNLLANTLHMVLTDSRFRADLAGGHMTLPDG

LEQVMWDEPPMMTLLGRWAARDTALGEQRIRAGDLLLLGLAAGNVDSAVR

DEDTPVIGNRSHLAFSRGPHECPGQQIARAIAESAIDALLRRLPDLRLAV

PEEELRWSSALMYRNLAALPVEFSPVSRKLPVLDADAAGTLRAKLPSAVA

AAPAAQGPGAGAGGPAAGRKRAPWWKPWAHQS

>CYP107AE(2524933762)Streptomyces sp. CNT318

VQESASSRQETHGQGGSPRRDEPVRAVRSGPAGRTGHLVTGYTEARQALG

DPRLSKDTAAFFANAPARRRLHPSVTRSMLASDPPRHSRLRKLVTGAFTT

GAVAALRPFVEQVTAALLRRWHSPGPVDAVEELAMPLPVAVICELLGVPA

SDRADVRRWSADLFAAGRPEVIDAASHTLADYMAGLVAEKRIRSGDGLLD

RLIAARDGADRLSEEELVSLGVLLLVAGHETTTHFIGSSLLALLQHPAEL

ALLRRAPHRVRDVLDELLRHSSPVSTATFRFTTQPSTLGGTDLPPGCPVQ

VAIGTANRDPGRYPSPDRLDLARDASGHLAFGHGIHRCLGAPLARLEGEV

ALRMVLTRFPDIRPAVDPAELEWRPTRLVRGLAALPVLV

>CYP154A(2524933887)Streptomyces sp. CNT318

VSVPELLTLDPTGSDPHGEHQALHARGPATRVDLLGVTAWSVSDPVLLKK

LLTGPDVSKDARQHWPQFAETVPTWPLALWVAVENMFTSFGSDHRRLRRM

VAPSFSARRVAALGERVESIVDDLLDVLARTPEGEAADVRELFAYPLPIQ

VICHLMGVPAERRAGFRQLVDNVFATTLTPEEAAANTASLYGVLDELIAE

KREQPGEDMTSLLIDARDEEGDGSALSEAELRDTLLLMISAGYETTVNLL

DQAITVLLTHPEQLALVREGRANWADVVEETLRFEPAVKHLPLRFAVRDI

PLPDGQVIAKGEPVLASYAAANRHPDWHGESADTFDLTRTDPGPEHLAFG

HGVHFCLGAPLARLEGITALEKFFARFPDARLGVPEEELRPVPSLISNGH

QTLPVVLRPRSAAD

>CYP1419A(2524934063)Streptomyces sp. CNT318

MRGPGARARPPGARTARRRDRRVYTRSHPVLFALLAATRRRPVVRLGRTV

LVHGTDACRQALTRLPLDRTAAGTTGGAARELSGGGALFDEEGRGHRGAR

RSLAEDLGTAGTERLRPVWQEILARRLAPLADGREVETAALARELAGATV

CALLGTAGDPAAVARAAADAAAAAVRDHLPGPRRPGTARAAARATARLEE

LLRERTGGAAEGAGAADGAGTPDGAGAPEGTGAALRAVLAVAAVATTAAA

LPRAVAWCADAGLWGQAADPAARETLVDELLRVTAPSPVLPRRAAAPGTL

DGCPVRSGDRLVLVARHAVGGHRDAPGCPAPAPAAVARLVFGAGPHTCPG

ARLARTQLADLLAALAPYRPVVVRARADRRAALPGWRTLTVRPGPAPSTP

RRRMHRNEEGSTHPNGVHGGNGVHGGNDRNGEAP

>CYP107U(2524934097)Streptomyces sp. CNT318

MPPLFDRQFALDPYPAYAWLREHAPVRRTRLPSGVEAWLVTRYADAREAL

ADPRLSKDPRRHGEQDTHGKGKVGIPGERSANLMTHLLNIDPPDHTRLRR

LVSTAFTPRRVAAFAPRVRELADGLIDSFAPRGEADLIHEYAFPLPIYAI

CDMLGVPQEDQEDFRTWAGMMIHQPGSPRGGVGRAVKRMRGYLAELIHRK

RAEADTAEGGGDLISGLIRASDHGEHLTENEAAAMAFILLFAGFETTVNL

IGNGTFALLRHPEQRAVLQRAVAEGDEKLLGGAVEELLRYDGPVELATWR

FATRDLVLGGQRVREGEAVLVVLAAADRDPAKFAAPDTLDLVRQDNQHLG

YGHGIHYCLGAPLARLEGRTALAALLHRLPDLRLAADPGTLHRRGGLIMR

GLRTLPVEFTPAEADDLRGEPV

>CYP180A(2524934175)Streptomyces sp. CNT318

VSGAVPEPGVPGPASAAASPEPAPQPAPGSAAPAPDRDVFDPRRYARGLP

HEVFRALRDGEPVAWQPEYEVLGWPAGPGFWAVTRHADVLRVLRSPGDFS

SYEGATQIRDPAPEDLPFIRRMMLNQDPPAHNRLRTLVSRAFTRRRVERF

EAQVRQRARRLLTGALRQARERGSCDLVAAVTDDFALLNLADLLGVPTGD

RGLLLEWTERIIGYQDPDQARTVLGPDGRPADPRSPAMLQEMFDYARRLA

SHKRAHPADDVMTALAHGLTGAELEMFFFLLTVAGNDTVRSAAPGGLLAL

AQHPRAQCTLRELVRAGQVRAQAVAVEELLRWHTPVLSFRRTAVRDLELS

GRRIGAGEKVVVFHASANYDERVFDAPHTLDLARAHNPHVAFGAGPHVCM

GAHFARLQLRVLHQEAARLLPPYELLQQGEGAGQVRRLVSNFIHGIKALP

VRLSGA

>CYP1240B(2524934267)Streptomyces sp. CNT318

LSTVPAPGHPSSGQMPPPLSRPNHEVTPLYGEEFAADPYAVYDRLRKYGA

LAPVEIAPGVGAMLVIDYRAALDLLHDSATWSKDSSIWLDSVPEDSAVMP

MLRGRPNALFTDGETHARYRKVISDSFGRQEPHEMRRDVQEVADNLIANF

ARDGEADLIGQFARLLPLLYFNRAFGMPDEESELLIQGIMGMFDSKTPEE

AAAADAAYTRYVTELTQLKQRQPGQDLTSWFMQHPAGLSAEEVVQQIVLT

LAASYEPLSNLIGNSLSRMLVDDRYYGNLSGGALTARDALHDVLRNEPPM

ANYSAHYPRRDVYFHGVWLRAGQLVLVSYAAASTQSGRSAPGESTGAAGS

GGGAHLAWAAGPHACPAQQPALLIATTAIERLTAWLSDIELTVRYDELSW

RPGPFQRGLVSLPARFSPVSPDQAGVPR

>CYP1031A(2524934638)Streptomyces sp. CNT318

MSTVPQDQPLIYYPFTPEFMEDPYPHYAELRRQVPVHEHPGGFWMLSRYA

DVDALLRSKHSVDQRHVAPGPFRDAYARAGVSEKPRLKGLALLDLDPPDH

TRLRKLVTKAFTVRAINGLEPMVRELVDEALDGIAAAGGGDLVEELAFPL

PFTVMTRMLGMPPIDTKHLRMLTSLLMRSVELTTDPEVMRVIEAADAEIF

EVVGDAVSRKRDDPGDDLLTALIAAEDDGDTLSHDELVAQVTTLFVAGYE

TTVNLISGGTLALLRNPDQLELLRARPGIGENAVEEMLRYDPPTHSSRRI

TLEPYHVGGYEIPAGSMVLANLAAANRDEEFFGPDAEELRLERENARKQL

SFGGGMHYCLGGALARIEGRVAIGELVRRFPGLAMDGPVEWNGLLTLRGA

ERLPIRV

>CYP163B(2524935284)Streptomyces sp. CNT318

MSSLITPDSGALDTLDLADPRTHAEYDLTQVWQRLRKEQPFYKHRPVGGS

EGFWVVTRYEDVSTLYRDTARFTSEKGNVLTTLLMGGDSAAGMMAAVTDG

PRHNDLRRILLKAFSPRVLTGVVENIHAAARQLLLDAVAREECDFARDVA

AHIPLNAICDLMAVPQADRPFVLECTEAALGSDGAAQSPAVAWQARNDIL

AYFEKLATERRKNPGTDAVSMLATGTLEGKPLTMDEIVVNCYSLILGGDE

TSRLSMTGAVAAFLEHPDQWRALLEGEVEVSTAVEEVLRWTTPAIHFGRS

ALEDVPVGDRGQEIKAGDIVTLWNNSANMDEEVFADPGRFDLARTPNKHI

AFGYGPHFCLGAYLGRAEIAAMLTALRDIVATIEPNGEPRRVYSNLLSGM

TSLPVVLRPA

>CYP157C(2516097662)Streptomyces sp. CNH099

VTTDSDPVPAAGPPDPAAGPETGAAPAPPPGCPAHSGGGRTDGPARMHGP

EAASNPYGLYERLRAEHGAVAPVLVNGDLPAWLVLGYRELLDVTRNPTRF

SRDSRNWRFLRDGQVGPDNPLLPLVTWVPMCSFVDGAEHRRLRSAVTDSM

KRFDRHGIRRHVTRFSGQLIDDFADTGRADLVGQFAEHLPMLVMTKALGM

AESYGPRLVEAARDMVKGSETAIASNDYIVQTLRDLVARKQAAPGHDFAS

WLVAHESGLGEDELVEHLRLVLIAAYSTTANLIANTLRMVLTDSRFRGNL

AGGHMTLPDALEQVLWDEPPFTAVYGRYATGDTELAGQQIKAGDLLILGL

AAGNLDPEIRPDLAVPVHGNRSHLAFSGGPHECPGQDIGRAIADTGIDTL

LARLPDLSLAVPEEELEHRTSLLSRELVELPVVFTPVRPGPRPAVGEPAA

AVVPGPAAPANGVPAPAAGERVPAAVASAAAPVAQDAAPEAPARSNGLPA

GARAAAGRRRRASLWRTLVAWWRGY

>CYP102B(2516098439)Streptomyces sp. CNH099

MAKPFLRTRPGAWPELSRIPHPPRRVPVLGDVFGFSPGTPVQDSLRMARE

LGPIFRRKIFGLEIVVVSGADLVTELADESRFTKRVVLAVHNLRDVGGDG

LFTAHNHEPNWQLGHDILAPAFTREAMERYHPVMLALARRLTAGWDAHAA

GGTAADVSADMTKLTLETIAHTGFGYDFGSFERTEQHPFIAAMVRALRHA

QGGNVPRPLSPVKNRGRGRRGAADAAYLAAVVDEVVRERTEHPDPGAQDL

LGLMLHTRHPRTGERLSPENIRHQIITFLVAGHETTSGALSFALYYLSRH

PEVTARARAEVDEVWGPTGEPSYRQVSKLRYVRRVLDESLRMWPTAPAFT

RAARTDTVLGGVHPMRAGAWAVVLIPALHRDSAAWGPDADRFDPDRFLPE

RVRARPAHVFKPFGTGERACIGRQFALHEATLVLGLLLRRYELRGDPGYR

LEIAERLTLMPRGFRLTPVRRRPAAPRPSSGVPGAAGATGATGATGAARA

AGSPG

>CYP105AC(2516099099)Streptomyces sp. CNH099

MSDTQSTSPRRLPTERPRPLDPPEELGRLRAEEPVSPLLYPDGHVGWLVT

SHAAARKVLSDQRFSARGDIKKVPFELPSGGRPWEQVPPGFFMHMDPPDH

TRYRRLLTGQFTVRRMKSLEPRIEQITEEHLDEMERQGSPTDLVPAFALP

IPSLAICELLGVPYGERQRFQDHSTTVMRIGSSGDEVAAAFAAIYGLIHE

LVQLKHKEPEDDLLSGLIATGELNDAELTGIGMLLLMAGHETTASMLSLG

TYALLRHPGEVKRLREDPELFGNAVEELMRYLTIAQFGVPRTALEDVELE

GKLIKAGDGVTVSLAAANRDPGRFEDADGFDVGRSTSGHMAFSYGIHQCL

GQQLARVEMRIAYSALFRRFPDLRLAVDADEVPLRAEATMYAASSLPVAW

>CYP285A(2516099236)Streptomyces sp. CNH099

MRLNKSAAPDEVDRHAVDLTDPLLFAHGDPHAVWEDMREHAPVHWQQVDD

TLGFWSVTRFEDADLVLRDHTLFTSQRGTMLFLLGKDDPARGRQMAATDP

PRHTRMRAPMQRALTNKRVEKYSDAVTAEVHRLLAPALGGERFDFAEAMM

ALPMATAGTMMGLPREDWPHLTRLTTMSIAPEDPEFGGPDGVDETLQGAH

RELFAYFHDILRERRHHLGDDLVSLLLDMEIDGSRLETGAVLSNCYSLIL

GANVTTPFVPTGAMAEVVGTGVWDEWRADPKLLNTGLDEALRWASPTNHF

MRYALQDVELRGEKIRAGDAVVVWLGAANRDAEAFADPFTFDIRRKPNRH

LAFGSGPHYCVGHTVARMSLKILFTELFRTFESFEFAGGIEHLHSNFVAG

IKHMPMAGRVRRDAVGRLGEPAA

>CYP146C1(2516099249)Streptomyces sp. CNH099

VTSPAYPVAELGSDEFYATTDPTALWRRFAADDAVVWTPPSTTHNGFWSV

FSHRACTAVLAADAPFTSEYGMFIGFDRQRPDAGGGRMIVVSEGAAHDRL

RRVIGGHLARATAGPLARVVARELGDFVAAAREQPVTDAAALGSALPNTV

VCELLGVPAADHERLRYLTQFAVGAPDDPRLMSPSTAHLQIMGYFADLVR

HRRRAPGDDLVSRLLGDGMSEHDVLINCDNVFAAGNATTQHSVTGAFDGL

AASPGALEVLRTDPAAVRTAVEEVLRWSTPGPHVLRVALRDTAVDGRPIP

ADTAVVSWLAAANRDARVFPDPYRFDIRRRPNRHLSFGHGLHYCTGAALA

RLELRMLLERLAEHVTAVRHAGEPERLRATKVNGYRRLPVSFEWRV

>CYP1813A(2516099459)Streptomyces sp. CNH099

MTTQRTHELDRHADVRAALADPVLVPEPPAGDGGPAGASMAWLRATVARF

SSGETHRRRRAVVTAELARLEPAALRRAAAAGPGGETRVRVVRTLAEALG

MPEPAAVAEAVTVVAGAYFGGADAGAGTGAGAAAGAGGAAGAGGAAGAGG

AAGAGGAAGADADRAVARLVALLAPEDADEVGLEAAANRIGLLVQACAAT

SALVEAADGGDAPLARVLRETPPVRIMRRVAARATRVAGRDIAAGDAVVL

DVAAAQRTHPVPLAFGAPPRVCPGRAHALALADGLLRRPTTPFAALHHRA

GAPLLLPNAWDHASAAALAARGFDAVGTTSLGVAAGLGLPDGAAATAEAT

VALARRLGRGGFLFTVDAEGGFSDDPEEVAELARRLYGAGAAGINLEDGR

PDGSLAPAGLHAAKIAAVKAAVPGLFVNARTDTHWHGRREDETAERLAGY

EQAGADGVFVPGLSDPDGIAALTATLLVPLNILYAPGGPSLPELAALGVR

RISLGSLLYRRALAAAVAAATDIRDGRPTDLDAPSYAEVQAAAAGGADGR

ADSGVAGGVRRDP

>CYP126B5(2516099729)Streptomyces sp. CNH099

MTGQQARVSTELPAVLDGFDLTDQERFTDGFPHEVFVRLREEAPVLWHPP

GTTVDGEGFWVLSRHADLKAAAEDPVFSSKGGGGREGGGTHIDDLKPGVH

AGSLINMQDDPRHRMFKDLVSPPVARQAVEARLPQLREVAARLVGQALAR

GRVNFQPAVTAPYTIECVGRVLGAPEADMPQLIEWGETLAGFEERLSGKV

NRAATETQYAMYEYSKKLIAAKRAAGGPPADDLMSVLAHRDIPADRGEEP

LSEYEREAFFCLVLIAGSEPPRNALATGVLALAQHPEQWRALRADRSLLP

GAIDEMLRWSTPTPYNRRTATRDTVFRGTEIKAGEKITFWWASGNRDGSV

FDDPTTFDIRRSPNPHLAFAHGTHSCLGEQLARIEMRVLLEELLDRVAEI

RIDGDVTWAPSNKHTVILRMPVELVPATAA

>CYP107AT5(2516099730)Streptomyces sp. CNH099

MAETSETSAAAGDAGAEGTEDPGIDPAIFSQLLPFDPFDPAFHADPYAVY

RKIREQGPVTRTPLGLVVVPGHAGVSAVLRDNRFGWGDGATVAEHFSEAP

DGTTVRPFIFMDPPDHTRIRGLVGQAFSARRVDALRARAGELVTELIDKA

VAAADGGPVDLIDAVAHPLPAILLGELIGVPPEKHERFRALSTDIAHGLD

PSLFLTPEEVTRRDSARAELYEYFGRLAEERRAAPAGDLISELVTAEDTD

GGLTGHELVVTLTLLLSAGFALTVNLIGNGMYALLQHPGQLAWLREHPED

VPAAVEEMLRYDPPGQMISRVALEDAEVEGVPVAAGEQVMLMIGAASRDP

EVYEDPDALVLSRPAGRNLGFGLGVHFCVGAPIARLAAQVAVAKLAALDL

ELETATPPRAPYIITRGLRELPVRLAAAD

>CYP107F(2516100083)Streptomyces sp. CNH099

MAQAESAPVCSFTEVEALAFDPQLRELMERPGGGALARIRMAYGEGEAWL

ATRYEDVRLVTSDRRFSRGALVGRDFPRMTPAPIVQDESINLMDPPEHAR

LRRLVAQAFTGKHVETLRPRTRRVVDGLLEAMARQGPPADVVAGLAAELP

LTTICDLLDIPDEDRAELRGNAVALMVTGKADHEAQLRAKSALRGYFQDV

CARRRRAPGNDLISELARARVDGEELADDELAVMAMVLLLTGHDTTTNQI

SNITYTLLTHPAHLTALRKDPERLPRALQELLRHIPFRKGVGIPRVATED

VEVGGVLVRAGEVVHVSYLAANRDPDTYEQPDELDLDRQGQPHMTFGWGQ

HHCIGSHLAMMELEVAIGSLLTRFPGLRLGVPAEDIRWNTSSIWRYPLAL

PVAW

>CYP105BA(2516100712)Streptomyces sp. CNH099

MTDDATCPLSNGLPTLRERPFDPPEALRAQGPVSRMTFPDGYEGWLVTSY

KLGRSILSDKRFSSAPTNKHLAFPSNRAADLEADIPGLFEHMDPPDHTRF

RRQLAGQFTLRRMRLLTPRIEEITAQYADAMLAKGPPADLVTDYAVPVSS

QVICELLGVPLDARDRFEGNSEKLLSLDVEPQEVQQVLGDLVELTGELLA

RKKAEPADDVLSVLVSGEEISLPESIGATLLLLVAGHETTANMLSLGTYA

LLSNPDQTELLRADESLMEGAVEELLRYLTVVHVGVQRTATEDVELDGLT

LRKGDTVLIHLPSVNRDPNQFPDPDRLDVTQGEIGHLTFSHGIHQCLGQQ

LARLELRIGYATLLKRFPGLRLAAQPDEIPTRSNMTVYGVHRLPVAW

>CYP154C(2516101042)Streptomyces sp. CNH099

VNCPHAAAHSGSGSGAGGPAALPEPLAIDPLVRDLAGETAQLCAAPPLTR

IELLGAPAWTVTRHAEARQLLTDARLVKDIGAWRLWQTGAVTAEWPLIGM

VNPGRSMFTVDGAEHRRLRAKTAQAITPRRLDALRPVVERVTAELLDALE

SAAAAGDGVVDLKEVFAQPLPMRVVCALMGVAESEIPRQMRLWKAFFSML

TPQDERLAVMAELDRVFTDMVRQKTAAPADDLTSALILADEGGEPLTEEE

VVGNLKSMIAAGHETTITLLLTTVRGLLTHPGQLDLVRAGTVTWETAIEE

ALRHDPPVTHLLMRFATEDIEIAGTVIEKGEGVVISYRAIGRDAGHHGAD

ADAFDVARPTPIRHMTFGHGPHICPGAALSRLEAAVALPALFARFPGLRA

AVPAGEIRNLPVLTQNDLESFPVRLA

>CYP157A(2516101043)Streptomyces sp. CNH099

MTPTPEDQAAREGSVAAEAQGAQGAGPGAEPARSAGSAGASGPAAATEAG

CPAHAGAVPLGGPRFHTDPRDLYREMRRDHGPVVPVELPGDIPAWLVIGY

RELHQVTSDPDLFVRDRGLWNQWEKIPSDWPLLPMVGQVMPSIHFTASAA

DHRRHAVMVGAALESVDPFDLRRHCEQLADRLIDSFCSRGNAELVGDYAV

PLPVLVLARLIGFPDAEGRQLAQVLNELADGGPGALKAYETFVGHMTRLV

AEKRLTPGQDVASRMLADPMHFTDEEVMLDLKDTTAAGFLPTADWIGNSI

RLMLTDDRFAAALGGGRHSVGQAMNEVLWEDTPTQILVGRWAARDTQLGG

RPVGAGDLLLLGLAAANDDPEIKQALGGTGGGYGASGYGGGGVPAQAGNS

AHFAFSYGDFRCPFPAQEIAEIIARTGIEVLLDRLPDLDLSVPARTLVRR

PSPFLRGMTALPVTFTPVRAAGGHP

>CYP1064A(2516101121)Streptomyces sp. CNH099

MPDDGIDRIPGPPAEGDGVEAIAAAGGLHTYQLRLHDEYGPVVRFPLPGV

DAAVSVADPVLLEATAEINKRPEQLFAFLEPLQESGNMQTLPPAEHVPWR

RVMLSVLAGRPAHEAHFAQFAALTEAMADRWAEQSASGPVELQKDLSKLS

LQLVCEFALGSALTSEESAGRVVAAFEDVLTDYLARLYQVDVGGTEEERQ

DRSERALAYLRTTVDQVLAAHRARGTTARADHSDLIAALVAAGEAPARIR

DTVLMTMMAAHHTTGVAISWALYLLSRHPEAADRATAELDRVLADRAAPG

YGDLRQLPYLDMVLREAMRLYPPGPYGAREATEDLRLGDYTIPAGTTVFY

PFWAVHMNPAYWPDPETFDPDRFTPEATADRHRLAYIPFGLGPRSCEGAT

LAMVEAKLVLAVLLKRFRFELAPGQTVTPIERFVLWAAEDIRMNLTPR

>CYP1043A(2516101469)Streptomyces sp. CNH099

VTARPPAAVPASWWLPRGIVALRVRIFARVNGEQTLTLPNAAYGPDVFER

VYAHPAADGRSAGAALSDLFWYWLSPGPEVHQEHLEPGPRYDDVAATTRR

ILAGDSAGHAAAAARVFARLLDTVPATRVSLVRLRDLVMPAWAEFAYELV

FREPCPPRARELITAHARDVISALKCTGLRHPRRRARLTAYLRERVAAGD

VPHALPASLTPAEQVHYLQGTFFNTAVVQLSEATAHVLLALARHRRVQDR

VAADPDDDRYLTRVLDETMRLYPLFGIAHRITTGAIPLDADTALPAGSVV

CFSYPDYHATGYDRPDEFDPDRWAGQSAKDAHHIPFGVAANRPCPAWRIS

PIVMRAAIREVLRRFRLDSTASHTRSLPNRAPCLLLPRHLPPRPRRLAAR

RALVRSRDKAEDVTRGVRQLLLGTVMVLHARRQRPAARYFAAHPAGSPPP

PGSCPVAHPEDEST

>CYP1265B1(2516102835)Streptomyces sp. CNH099

MADMTDGGTVSADEKLTEEADTLLAQLLLSPEGHKDPYPLYAELREKAPV

YKSQMGFWIVTPYELVNEVLRNQKVGRDAELFMGGRFGGEWNEHASLRRM

ASGLLWYNPPEHTRLRQAVNHAFTPRRVEAMDQTMRDLVDEYLDPLAAAG

GGDLLNEFAFPLPLATVTTLLGVPRDEAPALREPMQAFQRTFEIGLTADE

LLDADEGMEFTQKYFAELIARKRAEPQDDLISALIELEEAGQLSADELMT

FCNMLVSAGFETSTNTITGMVLQLTRHPDQLELVRENRDLVHNTVEEVLR

YDPPIQINARMTFEPLELGGAVIPPQETIIAILGSANRDPEQFPDPDRFD

ITRPNISHLSFGAGIHHCVGWAMAHKQIGIALNAMLDRFSSIEVVEEPVH

QPRVTMRGFESLKVALTSR

>CYP107U(2516103730)Streptomyces sp. CNH099

VPDAPVPELFGQEFATDPYPAYAWLREHAPVRRTTLPSGVEAWLVTRYAD

ARQALADPRLSKNPVHHADDARGKSKTGIPGERSAGLMTHLLNIDPPDHT

RLRRLVSKAFTPRRVAAFAPRVQELTDHLIDGFAGRGSADLIHEFAFPLP

IYAICDLLGIPREDQDDLRDWAGMMIRHGTGPRGGVARAVKKIRGYLAQL

IHRKRLGDGDDLVSALIRASDHGEQLTEEEVVAMCFVLLFAGFETTINLI

GNGTHALLRHPGQRAALQGALARGEHRLLETGVEELLRFDGPVEMATWRY

ATEALEIGGTAVAEGDPVLVVLAAADRDPARFDGPGTLDLARRDNKHLGY

GHGIHYCLGAPLARLEGQTALATLLTRLPDLRLAVDPGELRWRGGLIMRG

LRTLPVEFTPR

>CYP105AR2(2524582576)Streptomyces sp. CNH287

MPDPDSRPPRIPTERQNVLDPPTPLAELRKRCPVSPLSYADGHLGWLVTS

HELARKVLSDSRFSSRPELRHSAVHEVLGDGQPPEEDVPGMFVGMDPPDH

TKYRRLLTGELSVRRMRQLEPKIAGTAHELIDRMLAEHPSGSADLVTSFA

LALPSRVICELLGIPYDRWEHIQPVSEKMLRIDSTADEVKECYRIIFEFL

ADVVARKQREPADDLLGGLVESAELSAEELTSVAFQLFTAGHETTANMLS

LGVYTLLTHPAQLAALRADPALLDGAVEELLRYLTVIQFGISRGALEDVE

MGGEVVRKGQTVTVSLPAANRDPARFTDPDTFDITRPPSGGLAFGFGVHQ

CVAQQLARSEMRTGYGTLLERLPGLRLAVPAAQVPMRDGAIVYGVESLPV

AW

>CYP251D2(2524582580)Streptomyces sp. CNH287

MSTTYATAPGRAPGVGHALALMRSPLGFIQSLRGHGDLVRVDLGPMPALF

VTEPELIHTLLTDRRDLLDKGRFYDKVRPLTGDGVIHAYGAEHRRQRAMI

KPSFHREQIARYAEVMHEVTTRKTASWQAGEQIPLDKAMRDITFNILAST

MFGSAVGKEAHQELADLLPVVLEAIMWRTVSPGDLLEKLPLPMNRRFDRA

FAELRAAISRVMLEYRRSEDGERRADMLSMLLGARDEDTGEPMSDEEICN

QIVSITMAGSETAATAMAWMFYELDRHPEVERRVRAEVGEVLGQRDFELA

DAGRLPYCRTVLQEVLRVRQPILVISRRAVYDFPLGSGTVRAGTELFYSP

YAVHRDPELFPDPLRFDPDRWVARPAASLPKGAYTPFGAGPRHCIGEQFA

WAMMHAVLAIVVRRWRLVLPRGVQVREMPWATVNPQHMPMVPVPVGREES

VHGEDQRR

>CYP251B2(2524582583)Streptomyces sp. CNH287

MALATAAPPVPPMPGRLPLAGHAVQMFRDPLKFFQSVSARGGLVRIWLGT

KQAYVVSDHDMLYDILVRKAKSFEKGMQFDQARPLLGNGILLSEGDFHRR

QRLLMQPAFHHTRVAAYVEVMRESASEVFDGWRDGQEVTMYDMFYELAVR

IVIKALFSTDMVEQDVAQVYKSMPIVISGVEKRAAIPPALLDRLPTRSSR

EFHGSVARLYAMGRRIIADYRAQGRKADQDDLMTLLFAAQDSGNKEMTDK

QIQDEFMTLLTAGSETTPSAMSWSAMLLGRHPEVQRRLQEEVDTVVGERP

VTAEDLRALDYTRRVIQEALRLYPPVWALGRKCVRDVELGGHRIAAGTEV

LYSIYAVHHDPELYENPEAFDPDRWLPERAKGVPRSAFMPFGAGVRNCIG

ESFSWFETQTVLATMAQQWTVQPVTAEPVKPVAMGALVPGPLPMKVVRRA

K

>CYP1044A3(2524582584)Streptomyces sp. CNH287

MSTQPPAPSGTDHAAPLDPQSESFRTCPYAAYAEVRKTSPVVHQAALDEW

IVVGRDEIESVQRDHAHFTSRYNLDGAYPFSPEARAVLEDSLFFRVALYN

VEPPAHTRFRTLISEYFSPRNLRALEPAVRRTADRLAQGISGAGEADLLQ

EFAYPLPMTVICDLIGIPEQDRAQVKEWNNQWLALQVVPLPGEQQVHCAR

NVVEYERYVLRLLRERRDHPADDLLTVLANAAAEEDPVCAVEDAVVALRV

MIAAGHETTTNLIGNTVWHLLEDRALWEALVADSALIPAAVEEGLRFDSS

VQGAPRVATEAVKVGGTEIPAGGQVRVMFAAAGRDPEWVQDPASFRLDRQ

GPPRHLGFGHGIHFCVGAGLARLETRIALETLTGRFPSLRLAEGFTPEHL

PGGFVFRGLGALPVAWS

>CYP154A(2524582860)Streptomyces sp. CNH287

MLTSYGPEHRRLRKLMAPAFTGRRTEAMRPEVERINKQLLDALARTPPGE

AVDLRARYAFPLPMLVICGLFGIPEESRDALSDLVERIMNTNTTPAEANR

TMEDIASTFTELIATKREHPGDDLTTMLVSTRDEDGDRLTEVELQATLLL

LMSAGHETTVNLIGNAVHALLTHPEQLRLVLDGEVPWDDVIEETLRWAPS

VSSIPLRYATEDIEIGGGEGAGEGRGVTIRQGEPILATFGAAGRDPLKHG

PDAAVFDIRRKDREHLAFGHGVHFCLGAPLARIEARTALPELFARFPDIR

LAADAGEIARVPSFIANGPATLPVRLTPAD

>CYP1240B(2524582861)Streptomyces sp. CNH287

VTAPRMTSTPPARTGSLPVAAPTAHEVTPLYGAEFAADPYGVYDRLRAYG

PLAPVEIAPGVGAMLVTDYRAALELLHDPVTWSKDSRAWQETVPQDSPVL

PMMGWRPNALVNDGAVHRRYRQVITESFSLITPHELREHTRQVSETLIAR

FASNGRADLIAAFASQVPVLVFNRLFGMPDSYSDQLVTALCGMLDANTPE

EATAANEAFSAYIGELIGTKSRERGPDLTSWYMDHAAGLTPEELASEIVV

TMAAGHNATTNLIGNALARMLSDDRYYGTLSSGGLTPRDALHDVLQNDPP

MSNLSPHFPTRDVFFHGTWLRAGQLVLVSYAAANTQRGRTAPGEAASASG

SGGGAHLAWSAGPHACPVQNPALLIATTAIERLTAWLSDIELTVPHDQLV

WRHGPFHRGLVELPSRFTPITPDQAGATPWGSSPSFSTRSDATSTERPTA

SAS

>CYP1038A(2524583774)Streptomyces sp. CNH287

VETETDASYPSRPGQPVPVPEADPALVERWLSAGGELVDLLAQVRERCGG

VAAFRVGPAPAPTVLVTGPQAVQHVLAQRPDRYVKRSHRARLLVGDGVLS

ATGPAWKRQRRLLQPQFTGAGMRRHERRIEEAARCAAERWAAYARSGEPV

DVGREMHRFALDTIWRSLTGRPLDDRTERELDAVQVVATALPTLPSDAAG

ARETVAADLARIDAVAEHAIGAARSEDAAAGPEGPGLLRVLTDAAAEHPE

YTDQLIRDELVTLLAAGHETTATTLTWLYMLLDRHPEARQQALAAGPEGS

AERRAALQALVQETLRLYPAAWLLPRHATEADTLAGFAVEAGTDVLVCPY

LTHRDPQLWPDPEHFDPRRFLGPDARPGRLGAYFPFGMGPRACLGTQFAL

RESTALLELLLPAHTLDFHARPAKTSYGITVRPEGPALATLRPPHGR

>CYP107BY(2524584062)Streptomyces sp. CNH287

VTADPYPGYAWLREHDPVCPVSGPHVPGRMWLVTRYDDVRACLADRRLGS

GAPVNPDPHLPGLSNLDDPGHTRLRRLVAAAFTPAAVSRLRERTARTCAH

ALEAFAGRGRADLVAEYTRQVPVAVMHDLLGVPEAERAPAADVLEMWFGA

KFQQPRDEVKLAEVLGYVRKLVAYKRSHPGDDLPTRLIESGALTGEELEV

MVMTLIGAGHITTIQFLGTTILRLLDDPGRRAALLGGDVDWSHAINELLR

LDSPSHVAEYRYAGEDMTIADARVREGDVVLLSLAAANRDPNRFPDPGAL

DLTRDARPHLAFGHGAHTCLGSHLVKLETEIAITTLFGRLPDLTLDIPSG

EVDWEYAPTFRGPRTLPVTFTPPK

>CYP154Q(2524584791)Streptomyces sp. CNH287

MDARTPAAGCPYRLDPSARDIHAEAGQLRALGPAARVELPGGLEGWSVTD

PGLIRRLLTDPRISKDAHQHWPAYIDGEIPEQWPLRIWVDARNALTAYGS

EHTRLRRLIGPAFTARRIRALAPAVERITDTLLDELAQRQQQGPDGDAAD

GAVDLRAAFAWVLPLLVVNTLLGVPEELHGGFREKIGGLFATGISEEEAM

ANGAAVYQLLAELVALKREHPGDDVTSALIDAHDDETGTRLTEQELLDSL

LLLIGAGHETTVNLLDHMVVNLLENPEQLALVRSGEVVWSDVVEETLRHQ

APVATVILRFPVEDLHDPQSGLTFGRGEPLVINYAAAGRAPEVHGEDADL

FDVTRPERREHLAFGHGAHYCLGAELARLEARIALPALLDRFPGLALAVP

AAELVPLESFISNGHQEVPVLLGTPSGAPAGATAA

>CYP156R2(2524584792)Streptomyces sp. CNH287

MTTPPTASPGRAAAGACPYTGGAERPLPLYGPEFAADPRATYARLRALGP

IAPVEIAEGVNGMVTTTYRAALHLLRNTPDMFAKDPHHWEALRTGQIPPD

SPVLMMMGPRHNAMLLDGTAHSRLRGAITSSLELVDTHALEAGVARVADQ

LIDRFAADGHCDLITDYADALPMQVLIEMFGSPPDVGLAIVQGIAKLFDT

TQDAAEANVELEAACLALTQLKRDHPADDVTSWLVAHEARLTDAEMMHQI

LLLVGAGTSPCTNLISNALLLMIADDRFSGNVFNGVQPVGDALDHVLWED

PPVSNYAPLYALGQQSYLGVELRPGIPILISFAAANSDPALAPAADQRAG

NRAHLAFGAGVHACPVPDLARVISETAVERVLDRLPELALAVRPDQLERR

PGTFHSGWVSLPVTFPPAARTSGGN

>CYP102B(2524585247)Streptomyces sp. CNH287

MTAQQDMPGAAPAHAPEGFRRGFHGAEGDWPQLRRIPHPPRRVPVLGDVL

GAGLRTPVQDSMALGRELGPIFRRKGFGREIVFVWGAPLATDLADESRFA

KHVGLGVANLRPLAGDALFTAYNHEPNWQLAHDILAPAFSRAAMENYHPL

MLEVADRLTAHWDGHAAAGTPVDVPGDMTRLTLETIARTGFGYDFGSFER

TRPHPFLTAMVGALSFAQRRNLVPPALAPLLLRSAARRNAVDIGYLNRTV

DEVVAARRAADASGARGDLLDRMLQTAHPDTGERLTPQNIRRQVITFLAA

GHETTSGALSFALHFLARNPRVLARAQAEADRLWPGGARPAYAEVAKARY

VRQVLDESLRLWPTAPAFARAARADTELAGIHPMRRGAWALVLTTMLHRD

PAVWGADAEEFDPDRFAPKAVRARSPHVFKPFGTGARACIARQFALHEAT

LVLGLLLRRYDLHADPGYRLRVAERLTLMPAGLTLRLTRR

>CYP159A(2524585698)Streptomyces sp. CNH287

MSTVREAPDILSPRFAADPYAGYRILRDDFPLLWHEATASYLVSRYEDVR

RAFRDDVFTTRNYDWQLEPVHGRTLIQLSGREHAVRRALVAPAFRGRMLE

EKFLPVIARNARELIDPIRERGTADLVGDFATRFPINVIVDLLGLDKGEH

ARFHRWYTAIVAFFSNLAQDPEVVAAGHRTRDELAAYMIPVIRERRENPG

DDLLSTLCAAEIDGTRMSDEDIKAFVSILFAAGGETTDKAVSGLFGNLLA

HPDQLAAVREDRTLLAQALAETLRYTPPVHMIMRETAEDVELSGGVIPAG

STVNCLIGAANRDERRFRDPEVFDIFRGDLPTRSAFTAAADHLAFALGRH

FCVGALLAKAEVEIGANQLLDAMPDLSLRGGGVPAEQGLFTRGLHSLPVR

FTPVAPGTPDASAAPAARP

>CYP157A(2524586563)Streptomyces sp. CNH287

VTTQPDRDRTANTAAVPLASPRFRSEPGPLYQELRREHGPLVPVLLPAEV

PAWLVIGYRELYQVTADPVLFPRDPARWSRRDGLPADWPLRPVLSCQAPA

VQHTTGAAHHRHLALVQRALAAVSPLELARWTQESADQLIDGFGTRGDAD

LIAEYARALPLLVLARLFGLPETEAPPLGDALLDLADGGLRAPEAHGTLA

ARMRLLVAEKRARPGADLPSRMLAHPEPFTDEEYALDLLALLAAGQVPTT

DWIGNTVRLLLTGGTHSVPEAMNEVLWEDTPVPIPAGRWAARDTRLGGRS

VRAGDLLLLGLAGANTDPDVRAAAPGGAGGRTAGGRTAGGWAAGGRAGNS

AHFAFGHGEFRCPVPAREVAEVITRHGLEVLLDRLPDLELTVPAEALNRR

AAALPRGVTVLPVRFSPVPPRS

>CYP107F(2524587318)Streptomyces sp. CNH287

VDVADAVWSCPFDFAKALDFDPLLKKLLTEEPVARIRMPYGEGEAWLVTR

YDDVRTVTTDRRFSRSAVAGRDFPRMTPAPIVQSEAINLMDPPVSSRLRG

LVAKGFTPRHVARMRSRTQAVVDTLLDAMAESGGEADLIGHLAQPLPLTT

ICEVLDIPEPDRPRLRTCALTMMNIGAASKDAAVAAKAELRTYFSELSAE

RRRAPGDDLISTLATARDGADLLDEQELAVMAMVLLITGQDTTTYEIGNL

AYTVLTRPEVAGALRARPEALPQALQEMLRFIPFRKGVGIPRVATEDVEL

GGVTIRAGEVVHVSYLTANRDPAKFPRPDELDLERPATGHMTFGWGSHHC

LGAPLAEMELEVALGTLLTRFPGLRLTVPAAEVEWNTTSIWRHPLALPVA

W

>CYP163C(2524588239)Streptomyces sp. CNH287

VDTRPVLDAGRLRETDLADPLLHATHDLGPLWRHLRAEEPLYRQEETSRG

PGFWVVSSYRDTTAVLGDSETFSSERGNVLDTLLAGGDSAAGQMLAVTDG

RQHRTLRSALLKPFSPRALEVVVESVRRGTRQLVLDAVRDGKVDFAADVA

AHIPLAAICDLLGVPPGDRRRIIELTSSALSSAEGVPTEEETWTSRNGLL

LYFSELAAERRAKPYDDVVSLLVTKEIDGRTLTDEEIIFNCYSIIMGGHE

TTRFAMIGGLLALMDNPAQWKALKSGQVATASAVEEVLRWTTPALHSGRT

ATQDVFFGGQFIEEGDIVTTWLSSANRDEELFARPDEFDLSRSPNKHLTF

AYGPHFCLGAFLARAELSALLESLTELVAEAAPAGERRWVYSNFLGGLSA

LPVTLAPEPSAPLD

>CYP107U(2524588392)Streptomyces sp. CNH287

VHHEPSAPVAPPPPPLFDWEFATDPYPAYAWLREHAPVRRTELPSGVEAW

LVTRYADARQALADQRLSKNPGHHREGGAAHGKGKVGIPGERSANLMTHL

LNIDPPDHTRLRRLVSKAFTPRRVAAFEPRVQELTDRLIDAVLARRPGAG

GVGAEPGSADLIHDLAFPLPLYAICDLLGVPPEEQDDFRDWAGMMIRHDR

GPRGGVGRAVKKMRAYLAELIHRKRRDPGDDLLSGLIRASDHGEHLTENE

AAAMAFILLFAGFETTVNLIGNGTCALLRHPRQRAYWEDAVARGDEAAQT

VAVEEMLRYDGPVEMATWRFATEALELGGRRVEPGDPVLVVLAAADRDPA

RFAGPDVLDLARRDNPHLGFGHGVHYCLGAPLARLEGRIALGTLFRRLPD

LQLAVEPADLRWRGGLIMRGLRTLPVTFT

>CYP157A(2513746128)Streptomyces sp. MnatMP-M77

MTNPSSATPASTAGTGGGCPVGAGTGAVPLGGPGFLAEPRELYRSLRRDH

GPVVPVELPGGLPAWLVIGYRELHQVTSDGEMFPRDVSLWNQWENVPADW

PLLPMVGTPMPSIYFTAGAEHRRHVDMVVPALEEADPFEIRRHCEQLADR

LIDAVCTRGTADLVAEYAEPLPVLVLARLVGFPDDEGADIARVLKDLADG

GPGAQKAHLSFGEHMQRLVAAKRARPGDDVTSRMLAHPEPFTDQEYALDL

MAITAAGHLTTADWISNSTRLMLTEDQFADALSGGRHSVAEAMNEVLWED

GPTQILAGRWAARDARLGGRDIARGDMLLLGLGAANADPHIRQQVTASAV

RSGQGGNSAHLAFSHGEYRCPFPAQEIAEIIARTGIEVLLDRLPDLELAV

PATELVRRPSAFLRGTTALPVRFTPVRTTGDAL

>CYP154C(2513746129)Streptomyces sp. MnatMP-M77

VNCPHTAAAQTDPGAGTVVIDPMVQDLDGETARLRDAGVLARIDLLGVPA

WTVTRHAEARQLLLDPRLVKDIDAWGLWQSGVVTRAWPLIGMIDAGRSMF

TVDGAEHRRLRTKTSQALTPRRLEAIRPEIEKFTDELLDALDAARGEDGV

VDLKSVFAQPLPMKVVGMLMGVDESQHAMLTRQYKAFFSMLTPQEERLAL

LAELDVFYTDLVREKTARPTDDLTSALILAEEGGEPLTEEEVVGNLKAMV

AAGHETTIGLVLNAVRALLSHPDQLRMVLAGEAGWDAVIEETLRWDTPTT

HLLMRFATEDITVGDDVIRKGEGVVVSYRAIGRDVGHHGPDADAFDITRP

TRNRHMTFGHGPHICPGAALSRVEAGIALPALFTRFPGLRLAVPDEEITK

LPVMTQNDLTAFPVLLG

>CYP1047A(2513746135)Streptomyces sp. MnatMP-M77

MSTQTGPALGTPSRGHAFVPGPRGLPLVGNLPEFGKNPLAFFELLRGHGD

MVRWRFGRKRCVFLADPDLVGELLTETERTFDQPRLGIAFRTVLGNGMLV

ARGRDWRRKRSLVQPSVRPKQVTSYATTMAGCAVELADRLADGQRIDVKR

EMSALTQKIAVRTIFGVDTPADSEAMGRAMDVAQTEIGKEFAGLGALLPD

WVPTPGRTRIRKAAAVIDAEVRRVVARHRDGDEERPDLLSRLLTAVDESG

TRLSDEEIRDEAVTLYIGGHETTSTTLVWAWYLLARNPRVREALAEELDR

VLGDRDPGFGDYAQLTYAQAVVKETLRLFPAVWLITGIAKEGATIGGLPV

AEGTRVWSSQWATHRDARWFPEPEEFRPERWDAESGDAIPEYAWFPFGGG

PRVCIGTRFAMVESVLLLAVLARRFTLDVDPGEITPLTGLTLQPDRDVLA

TVRAR

>CYP157C(2513746155)Streptomyces sp. MnatMP-M77

VTTPFHHEPGTVPPPQCPAHNLDIGPGGLRRLHGPEAENDPAGLYDKLRA

EHGTVAPILLHGDVPAWLVLGHSENLHVTRTPSQFSRDSRRWRALQDGSV

APDHPLAPIFTWQPICVFADGAKHERQRGAVTDSMERIDTRGVRRHINRF

SNRLVNDFCEKGTADLVGQFAEHLPMMVVCAIFGMPEEYDERLVQAARDM

TRGTETAVASNAHIVSVLTRLVERRRAEPSPDLASWLVEHPATMTDTEVI

EHLRLIMIAAYESTANLIANVLRMVLIDPRFRARLSGGHMTVPEAVEQTL

WDEPPFTAVFGRWAVGDTELGGQQIKAGDALLVGIAPANTDPTVRPDLGA

DMGGNRAHLAFSGGPHECPGQDIGRAIADVGVDALLMRLPDLELGVGESE

LHWVGNIMSRHLVELPVKFAPGPQQKLDADPLTVMARAPRPADAWEISSP

ARQVPEPRHEAVVAQPAHAPGAAPTAEPDPAPAAPPAPEPAAAPEPAPVA

TIPQQRRPAAPARFWQAVTRWWSGY

>CYP107BY(2513746324)Streptomyces sp. MnatMP-M77

MTADPYPGYAWLREHDPVCPVGGPHVPGRMWLVTRYDDVRACLADRRLGS

RAPVDPDPHPPGLSHLDGPGHARLRRLVAAAFTPAAVARLRDRTARTCAD

AVESFAGRGHADLVAEYTREIPVAVMHDLLGVPETERAPAADVLDMWYRA

KFRQPRDEASLAELLDYVGELVAYKRTHPGDDLTTRLIDSDALTGEELEV

MVMTLIGAGHITTIQFLGTTVLHLLDHPGHRAALLRGDLDWPRAVNELLR

LDSPSHVAEYRYAGEDMTLADARVGEGDVVLLSLAAANRDPDRFPDPGTL

DLTRDARPHLAFGHGAHTCLGSHLVRLETEIAVTTLFGRLPDLALDIPGG

EVAWGYAPTFRGPLALPVTFTPSTSR

>CYP107BX(2513746411)Streptomyces sp. MnatMP-M77

LTTTDPTRPAPVPMHRLFFEEPGPPRPAELPGGDPAWLVSRYADVRQVLS

DPRFGRARLYAPEAPALSGVPDLVNNPDLMFNQDGSDHLRLRRTLRRAFT

PRAVARWRPWIAATVEGILDRLESRPQPADVVAEFALPLPVAVISRLMGL

DESAWDRVRYWSEHAFSDGTHEREQVAAALKEFSAFGAHLLAERRSTPGE

DLVSGLVTAADEEGGVPEAQLVSLVCGLVVGGHDSTMTMLGNALLYLLGE

RRETWPRLGADEEAAGLLVERLVHLVPLGDDRGSTRHAAEDVEVSGVRIP

AGAIVIADCGMANRDPDVFPPATLYDLFAPLEAPTLSFGAGPHYCLGAWL

ARTELQLALHRLAARFPELRLADPVGAVVWRTGTTSRSPRRLGVRW

>CYP154C(2513746649)Streptomyces sp. MnatMP-M77

MTRIALDPFVRDLDGESAALRAAGPLAEVELPGGVHVYAVTRHAEARALL

TDSRVVKDINVWNAWQRGEIPMDWPLIGLANPGRSMLTVDGADHRRLRTL

VAQALTVKRVERLRAGIEALTNASLDRLAAHPAGAPVDLKAEFAYPLPMN

VISELMGVDAADHPRLKELFEKFFSTQTPPEEVPQMMADLGTLFTKIVDS

KRANPGDDLTSALIAASEDGDHLSDEEIVNTLQLIIAAGHETTISLIVNV

VEALATHPEQREKVLNGEIGWDGVIEETLRWNTPTSHVLIRFATEDIKVG

DRVLPKGEGLIVSFGALGRDEEQYGPTAGDFDAGRTPNRHIAFGHGPHVC

PGAALSRLEAGIALPALYERFPELDLAVPAAELRNKPIVTQNDLHELPVK

LGCPFGHDA

>CYP157A(2513746650)Streptomyces sp. MnatMP-M77

VTTVSGCPVTHTSVPLSGPRFQSDPVQLYREMRRDHGAIAPVVLDGDVPA

WLVLGYRELHQVTGDPVLFSRDSDLWNQWDRIPDDWPLLPMIGRKQNSIL

YTVGERHSVRAMMISNALEGVDPFSLKRYAEEFADELIDRFCTKGSVDII

AEYAKLLPALVLARIYGFSDEEAHPLVGAINDMIDGRERALAGQQHLATS

MFRLLADKHAEPGDDVASRMIADPGGFTDEEVAQDLMVMMAAGHQPTADW

MGNSLRLMLTDDRFAASLSGGRHSVAEAMNEVLWEDTPTQNVAGRWAARD

THLGGRHIRAGDLLLLGLAAANGDPQVRTDGSALTGGNNAFLSFGHGEHR

CPFPAQETAEVIARTGIEVLLDRLPDVDLAVAADQLTRRPSPWLRGLTDL

PVLFTPTPAIGRPGSFGGPA

>CYP107L(2513747227)Streptomyces sp. MnatMP-M77

VLTTEPLVDLAALGEQFTRDPYPAYAALRAKGPVHRVRIPEGAEAWLVVG

YEQGRALLADQRLSKHWSRASPSLGVSKVSAGSSMLGSDAPDHTRMRKLV

AREFTPRRMEQLAPRVQEMTDGLLDAMLAAPDRTADLVEALSFPLPMAVI

CELLGVPSLDREAFRTWSGQAVSSVDPSLRASSTQAMTAYIAGLLADKRE

KPGEDLLSALIHTSDEDGDRLSGDELIGMAWLLLVAGHETTVNLITNGVH

NLLAHPDQLAALRADFTLIDNAVEEILRFEGPVETPTYRFTTDPIEVGGT

VIPGGGELVLVAMSDANRDPARYPDGSRFDITRDARGHIAFGHGIHYCLG

APLARIEARIAIRSLLERCPELRSAADPATLPWRTGILMRGPLSLPVGW

>CYP107L(2513747228)Streptomyces sp. MnatMP-M77

MAVLDLRDLPDFTTNPYPYYAKLRAEGPVHAVRTEEMEQRVWLVVGHAEA

RAALADQRLGKDWRHTGLWTESEAALSANMLELDAPHHTRLRRLVSREFT

ARRIEALRPRVTEITGELLDAMAPRGSADLVDALAFPLPMTVICELLGVP

DIDRDAFRALSNGIVTPTPEQRGADPAGAMGAYLDELIENKRRSPGDDLL

SGLIRTGEADGEGLSSAELVGMAFLLLVAGHETTVNLIANGVRALLDHPD

QLALLRADPGLLDNAVEEMLRYDGPVETATFRFARETLTIGDTEIHVDEP

VLVALASADRDPLRFRDPDTFDIRREPQGHLGFGHGVHYCLGAPLARMEA

RIAIGALLERFPGLARDPSGGELDWPPGLLMRGARGLPVRW

>CYP159A(2513747371)Streptomyces sp. MnatMP-M77

MPADRPDALPAARRRHRLAGGQPGLLAPGATTDPYRLRLYRLLRTHYPLG

YDPGLGAWLLSRYTDVALALTDPRFTGYPHDGAPRGRAPVPLGLCRGSLV

CVPPAVPYRTAEPAVERTAYVLARRIARRDRADLVADFCRWLPAGAAAVA

AGRGLSTLPRGGAPGRRAGAVPDDCAGPTALREHALASFLANMLDDPDLL

AAATAGDGAATLLGRAWAETLRRDPPVQIVLRRTRTEVAVSGGTLPADAP

VACLIGAAGRDPARFGAPDRFDPLRSDADPLLIGPAGCPAALLGGLEAEH

GLRALLAAMPGIRWADGFRPAAGGLLTRGPRTLLVRPS

>CYP159A(2513748255)Streptomyces sp. MnatMP-M77

MRCPHLPDGFDFTDPDLLQSRVPHPEFALMRETAPVWWCTQPRNISGFGD

EGYWAVTRHADVKYVSTHPELFSSNTNTAVIRFNETISRDQIDVQKLIML

NMDPPEHTRVRQIVQRGFTPRAVRSLEAALRSRARSIVGTAQASADAHGS

FDFVTDIAVELPLQAIAELIGVPQEDRSKIFDWSNKMAAYDDPEYAITEE

VGAEAAMEIVAYSMNLAAARKECPAQDIVSQLVAAEGEGNLSSDEFGFFV

ILLAVAGNETTRNAISHGMHAFLTHPEQWELYKRERPKTTAEEIVRWATP

VVSFQRTATQDVELGGQRIRKGERVGLFYSSANNDPEVFDAPEAFDITRD

PNPHLGFGGGGPHFCLGKSLAVMEIDLIFNAIADVLPDLRLLEDPRRLRS

AWLNGIKELRVTTAAA

>CYP156B(2513748298)Streptomyces sp. MnatMP-M77

MDPQPGATPYTAPAGCPMHQQRTALYGPEFAADPHRFYDAARTHGPAAPI

ELSPGVEATLIVQHEAALRVLQNPALFARDSRRWAALREGAVPMDSPVLP

MMVYRPNCLFTDGAEHLRLRKAVTESLSRLNSSRLSRDVERIADYLIDQF

IERGTADLLNEYAKLLPLLLFNQLFGCPGDIGDRLTRSMSAIFDGEDVLR

ANAELTECLMELVALKRRQPGEDITSWLIQHPAGLRDEELKDQLVVLMGA

GVEPERNLIANALLLMLAGEAPGAPERRGSGMLVEDALDDVLWNNPPIAN

YATHFPVQDIELDGVVLKAETPVLISFAAANSDPGLTDARQTLSKGAHLA

WGAGPHVCPAKSPATLIALTAIEKILNTVPDLALAVPASGVAWRPGPFHR

ALVALPVRFTPTAARRAATGAQPTAPVSAQLPDPFRNTPSAPSAAPRHAQ

EPAKKQKGWWSSFLDVFRV

>CYP107CA(2513748813)Streptomyces sp. MnatMP-M77

MTATGHEIRDYPFGPVDRLDLDPALVEICGEHPVLRVRLPFGGDGWLVTR

YADVRAVLSDPRFSRSAAAGDHVPRTVAVAPPPTSIMGMDPPDHTRLRRR

VMRAFTVRSIDALRPRIAEIVNDLVDTMTEGDGPADLAAVLTWPLPITVI

CEMLGVPRADQDRFTEWVDGLLILDDPERSADARRQLGDYLAVLIARRRA

EPTDDLLGELAADSGKDPLSEEELVGLGVSLLSAGQEATANQIGNFVYTL

LTRPALWRELVADPSIVPRAVEELSRFIPISATAGFTRVATEDLELGGQL

IRAGDAVVAELGMANRDSAVFDRPEEIDFHREQIPHVTFGYGIHHCLGAQ

LARVELRVVLETLVTRLPGLRLAVPADQLAWRTERLIRGVAALPVRW

>CYP159A(2513749578)Streptomyces sp. MnatMP-M77

MSVPAPDILSPEFERDPYRAYRLMRQDTPLMWHEATGSYIVSRYEDVERV

FKDKEGEFTTENYDWQIEPVHGRTILQLSGREHAVRRALVAPAFRGSDLR

EKFLPVIERNSRELIDGFRDAGSVDLVADYATRFPVNVIADMLGLDKSDY

ERFHGWYTAVIAFLGNLSGDAEVARAGERTRVEFAEYMLPIIRKRREAPG

DDLLSTLCTAEVDGVRMGDEDIKAFCSLLLAAGGETTDKAIAGIFTNLLR

HPEQLEAVRADRGLIPRAFAETLRYTPPVHMIMRKSATEVELSGGTVPAG

VTVTCLIGAANRDEDRYRNPDSFDIFREDLTATNAFSAAADHLAFALGRH

FCVGALLAKAEVEIGVGQLLDAMPDLRLADGFDPVERGVFTRGPQSLPVR

FTPVSG

>CYP157B(2513749579)Streptomyces sp. MnatMP-M77

MSTSSPSFGPQAPASCPVGAGAGAVRLSGASYQQTPTQLYRSLRRDHGAV

APVLLDGDVPAWLVLGYAELSYVLTHDELFARDSRRWNQWETIPPDWPLM

PFVGYQPSVLFTEGDEHRRRAGVITEALEGIDQFELARDCRRIADRLIAD

FAGSGRTELMSSYVHALPMRAVVEMCGMPVSGSDTQQLVDDLRISLDAGE

GDDPVAAYGRVGDRLRQLVEDKRAAPGADITSRMVTHGAGLTDEEIVQDL

ISVIAAAQQPTANWICNTLRLLLTDERFALNVSGGRLSVGEALNEVLWLD

TPTQNFIGRWAVRDTQLGGRHIRAGDCLVLGLAAANTDPEIWPESYVGAE

NSAHLSFSGGEHRCPYPAPLLADVMARTAVETLLEQLPDLMLAVDPTELS

WRPSIWMRGLSTLPVQFSPMAQ

>CYP107U(2513749622)Streptomyces sp. MnatMP-M77

VNDSPAPRPSEPSACPHSPAEPHGAPELFTWEFATDPYPAYAWLREHRPV

HRTALPSGVEAWLVTRYGDAREALADARLSKNPANHAESPHAKGKTGIPG

ERKAELMTHLLNIDPPDHTRLRRLVSKAFTPRRVAEFAPRVQELTDRLID

DFVEKGSADLIHDFAFPLPIYAICDLLGVPEEDQDDFRDWAGMMIRHGGG

PRGGVARSVKKMRGYLAELIHRKRENPGDDLISGLIRASDHGEHLTENEA

AAMAFILLFAGFETTVNLIGNGTYALLRHPGQRARLEASLAAGESALLAT

GLEELLRFDGPVEMATWRYATEALTLGGEEIAAGDPVLVVLAAADRDPER

FTDPDTLDLARRDNQHLGYGHGIHYCLGAPLARLEGQTALTTLLRRLPDL

RLAGEPGDLRWRGGLIMRGLRTLPVAFEPGSRTRKSDTASTL

>CYP107P(2513750063)Streptomyces sp. MnatMP-M77

MHVSFDPWSPAFVADPYPAYTALRAAGRAHWFEPTGQWLIPHHSDVSALL

RDRRLGRTYLHRFSHEEFGRTPPPAAHEPFTTLNGQGILDLEAPDHPRIR

RLISRAFTPRTVENLAPTVRRLAAELVDAFVAKGGGDLLAEVAEPLPVAV

IAEMLGVPEADRGLLRPWSAAICGMFELNPSEETAEAAVRASVDFSAYLR

GLIAERRADPGDDLVSALIAAHDEGERLTEQEMISTCVLLLNAGHEATVN

TTVNGWRTLFHHPEQLAALRADPALLPSAVEELLRYDTPLQMFERWVLDD

IDLDGQVIERGAEVALLFGSANRDPERFARPDTLDLSRQDNPHLTFGAGI

HFCLGAPLARLELAASFGELLRRAPALRMTAEPEWHPGYVIRGLKELRAE

V

>CYP107BZ(2513750212)Streptomyces sp. MnatMP-M77

MTGTQTLSKYWMLTNEFTQNPYPVLEHVRREGPVRELSFPDGGRAWVVTR

YEEAKAALADPRLSRDIHVHYRLMSRRTGRALTPPPEEANHLANLEPPRH

TPLRRAISFAFTPRRAEALRPKVERIADDLLDRLGEAPEAELIAGYADPL

PVITIAELMGVPADAWPDFLRWSTALRTHSPTDGSGALDRNVQELSAYMA

DLIARKEREPGEDLLSALIHAAPENRLTPTEILSTGFALMTGGNDTTASL

VGGVIAALLTHPRERARLLADTGRWGKSMDELIRYVSPISNALQRVTTEP

VDVGGVTIPAGEVVVVCVMSTNRDTGQFPGHPDRLDLDRVKPAHLSFGFG

IHYCSGAHLAKVITEVSARRLFERFPAARLAVDPSRLRYQQNVVVRPLEA

LPVLLRP

>CYP163B(2513750219)Streptomyces sp. MnatMP-M77

MTTATSTLHGVDLTDPQTFLDRKDDLVGLWQEFRSHSPVHWHPVEGRQVP

GFWVLSRYRDVMEVYRDNKRFTSERGNVLATLLEGGDSAAGQMLAVTDGR

RHRELRNLLLKAFSPRLLASVVEGVRRRADRLVREAVGRGSCDFAQDVAE

HIPMATIADLLGAPAADRDYLLSLTKQALSAEEAGQSAEEAVVARNELLY

YFSELAEIRREDPRDDVVSVLATATVDGKPLTEQEIVFNCYSVIIGGDET

SRLSMICAVHELMEHPDQWRRLVSGEVSVDSAVEEVLRWVTPAMHFGRRA

LTDVEIGGRTIRAGDVVTLWNSSANYDETVFDRPEEFDLARTPNKHVSFG

YGPHFCLGAYLGRGEIHALLTALRTHVAAMEPTAPARPIHSNFLHGYSSL

PVSLRPVTGDRT

>CYP1046A(2513750302)Streptomyces sp. MnatMP-M77

MSVDRTACPGQPSVPPGEGMLTHSRALRFWLDPANLAARLEQAGPVVPTR

TGPATAFQVNDPALLRKIGTDEDTFRFWGPDPSLRDFTEDGVVGLEGAAH

RKRRAVMRPAFSASRLTTLGPAAQARTRRLLAGLPADRPLDMRMEMSRLA

CGLLVSCVLNSELAPDTLSRIAAARSTLSSGMFWRYALAPWPWVPVPRRR

ACRRALAELDEAVRQLLARHQPHPDGRDLVSVLEAATPENPRVVQRDVRA

LLIAGMETSASTLAWACYELGRHPHYQQALRDEADAAPDPSRLQADQLPL

ATAFVQEVTRLHGIPFLVRRTRHQTVQGGVRIPAGAVVTLPLGALRRDRD

RYRDPDAFDPKRWLPHAEPPPAPTALLAYGLGPRYCPGAAAADAMLPVAL

ATLAGSRTLRPARPNRKIGMSLELTPTPKGLTMYATPR

>CYP107AE(2513750312)Streptomyces sp. MnatMP-M77

MNDDSEDSRSAPDLSSGCTAPRPRCPVRAVTSGSDGRESYLVTGYAEARD

ALSDVRLSKDTAAFFAGKGSRRRLHPAVAHTMLASDPPRHTRLRKLVTGA

FTSGSIAALRPSIARLTDELLDRWPAGGAVDVVAALAVPLPVMVICELLG

VPETDRPRVQRWSADLFAAGDPGRTDAASHAVAAYMTGLIASKRLHPGDS

LLDRLIAARERGDRLGEEELVSLAVLLLVAGHETTTNFLGNAVLALLRHP

AELNRLRGDPGTIPRALDELLRFDPPVSTSTFRFAAEAMSLGGTEIPAGV

PVLVDIKAANRDPERFTAPDRLDLDRDATGHLGFGHGIHRCLGAPLAKAE

AHIALHAILTRFPDIRLAVAADRLDRRRTRLISGLDSLPVLL

>CYP124G(2513750422)Streptomyces sp. MnatMP-M77

MTVPYQLSEGRVLRAADVDLADPAFWRLPRPVRLRAFALLRELEEPVLFT

PRAGTARTAGKPFHALVRHADVRTASRTPQVFASAPGVTTPEPAGWAKAL

FGNSMVNMDGPEHAALRRIISRRFTPRLLAEAEENVGRLAGRLVDELIAE

RPRDFMPSAASRLPLEVICDLMGVPAAYRARIAEQIDHASEHVGVERRGR

ARLRIPGRGLASLARMQFVMGRLARERRRHPEDDLVSALVNADIDGEALS

GRQLGAFFSLLLVAGVETTRNAIAHGLFLLDRHPEQRELLRSDFDRYIDG

AVDEIVRHSTPIIQFRRTVAEECALGGRTFLPGEKVALIYASANRDETVF

THPDRFDITRSPNPHLGYGGGGPHHCLGAHLARLEMTALFRELIARRPVM

RDLGDPDLVDSNFDNRVGSLPFTFGPTFT

>CYP162C(2513750475)Streptomyces sp. MnatMP-M77

VSGEAGGCPAGTARGPDLTDPATYRDADYFAQWRRARRDHPVVRLESPRF

GAFWSVTAHAAARQVLERPESFTSTRGMRLGGEPAAVSAASGRMLVVSDG

PAHTRLRSAHAPWFAGQAVSRLKDALRSRLDALLADLADGSPVDVPARLA

RPLPTWLVCGMLGVPEEDWEELALLAAAAFDETETSTASARRAASAGVFA

YFAELLEKRRADPGDDLVSALVHQPGGDRLTDEEILLTCDGLVNGGLGTT

RHAVSGAVLAFAAHPRQWERLRADRGLVPTAVEEILRWVSPPLHIMRTAT

EDVLLDGARIRAGERVVLWIPSCNRDESVFAEPDAFRVDRRPNPHLGLGG

GPHYCIGASLARLELRTLLRALLDHVARFESDPVLARTPSTFLHGLDRLE

VTLIPAADAAACSPAESEDP

>CYP208A(2513750504)Streptomyces sp. MnatMP-M77

MRTDPPGPPVSALPGLLRKLAVDRLEMMKDAAALGDAVRVSMGPKKLYIF

NRPDYAKHVLADNSDNYHKGIGLVQSRRVLGDGLLTSDGEVWRAQRQTVQ

PAFKPGRINRQANAVAEEGAKLVALLRAHEGGGPVDVLHEVTGLTLGVLG

RTLLDSDLSSQDTLAPSFEEVQDQAMLEMVSQGMVPGWLPLPPQARFRRA

RRELYRVADLLVADRSARMADGEPGDDALARIIEAAGRGNGPPRRVRGKL

REELVTLLLAGHETTASTLGWTLHLLERHPEVRAAVREEARSVLGERLPD

LDDLHRLTWTTKVVQEAMRLYPPVWVLPRVAQREDEVGGYTVSAKADVLI

CPYIMHRNPRLWEDPERFDPERFDPQAVASRPRYAYIPFGAGPRFCVGSN

LGMMEAVFVTALITRDLDLRTVPGHRAVAEPMLSLRMRGGLPMTVSVAG

>CYP154M(2513750516)Streptomyces sp. MnatMP-M77

VESRCPVVIDRTGQNIHAEADRLREQGPVARVELPGGVRAWSVTGYDVAL

SVLGDQRFSKDPRKHWTAYANGEIGDDFPLIGWVLMDNLTTAHGSDHSRL

RRLTAKAFTPRRVSAMRSAIEQACTELLDELAESGPGDKVDLKARFAHPL

PARVICDLVGVAPQDRAAMLRGGEVNVDTTTSPEDAAANVERWHQEMHEF

VESKRRTPGDDLTSDLIAAQEEDGSRLSPSELVGTLHLMLGTGTEPVMNL

IANAVHLLLTHPEQRAELRAGRISWDDVIEETLRAEAPVAHLPFRFPTED

VEIGGVTIPRGDPVLIAFAAAGRDPAVHGPSAGRFDPSRADKAHLAFGHG

IYRCIGMPLAREEARIVLPALFTRFPDLDLAVAPEDIEPQGSFIMNGLRT

LPVRLRSEGAGR

>CYP156A31(2513751095)Streptomyces sp. MnatMP-M77

VTTYTRAAERFALYTPEFAADPHRVYRQMRDFGPLVPVDLQPGVPATLVI

GHALAVEVLHDPVRFPADPRVWQQGVDPGCPILPMVGYRANALRSAGHEH

ARFRASNKDALDPVLLHQLGTTVARISRQAINRFAHTGEVDLLAAFVKPV

VLNSISTEMGVPPELARPIGESMAKVFEGIDAAAANELLMRSLDALIELK

RARPGQDITTRLLQHRTRLSPEELAEQCVTLFGAGYEPLKNLIANTQLRM

LSDQQFRGTVSSGMLTVRDAMDEALFTDPPMANYCLSYPPAPVNINGHVL

PAHQPVVISMAAANNDPALGDPKLRLGNRAHLAFSAGPHACPAQDASLVI

ARTALEELIDALTDLHLAVPAADIRWRPGPFHRALQSLPVRFTPIAQLPE

H

>CYP154AM1(2513751096)Streptomyces sp. MnatMP-M77

MSPTDVLVLDPKARDRVAEDRELRARGPVARVDLLGLEAWAVSHPDYLKR

LLTDNRVSKDSRRHYPDFEQTVQRWPLWLWIAVENMFTAYGTDHRALRRL

VGPAFNHRRTQAMRPVIQDITDRLLTRMLAKQEAGDLVDLRADFAHQLPM

DVIHHLMGMPREWQEAMADPVAKVFDTSLDIAAALQNGEGLKALLARFIE

RKRAHPADDLTTDLIHARDGERRLSEEQLVDTLLLVFTAGYETTVGLIVN

THRNLLTHPRQLDIVRRNPKEATVAAVQETLRRDASIAFLPLRYATEPID

LPEAGVRIGQGEPILAAYSAAGRHPDIFEQPDVFDVLRPNASGHLAFGHG

AHLCLGAPLAIAEAEIATRSVLNAFPDAVLAVEEHQLTPVESVISNSLRE

LPITRRGEKKTGEAA

>CYP107F(2513751423)Streptomyces sp. MnatMP-M77

VENTSVQNKETVRNCPFDYAHELEFDPQLRQLLTEEPVSRIRMAYGEGEA

WLVTRYEDVRTVTTDRRFSRSAVLGRDFPRMTPEPIVQAESINLMDPPAS

SRLRGLVAKSFTPRRVEQMRGGTQRVVDRLLDEMEEEGSPADFVARVSAP

LPLITICEALDIPEADRPWLRAHAMTMMNVGAAGKQDAVRAKAELRGYFQ

ELTADRRRSPGEDLISTLATARDGDELLDDDELAVMAMVLLITGQDTTTY

QLGNIAYTLLTRPDLLRSLRAEPQRLPRTLEELLRHIPFRKGVGIPRIAL

EDVELSGVLIKAGDVVHVSYLTANRDSAKFDRPDELDPDRPTIPHMTFGW

GAHHCLGAPLATMELEVAFSTLLTRFPALRLDVPPEDVSWNTTSIWRYPL

ALPVTW

>CYP105D(2513751625)Streptomyces sp. MnatMP-M77

MTESTTEPARQDPAPTAPPTQPTSTTPFPQNRDCPYHPPTGYQPLRADRP

LSRVTLFDGRPVWAVTGHALARRLLADPRLSTDRTHPDFPVPAERFANVE

RRRVALLGVDDPEHNAQRRMLIPSFSVKRIAALRPRIQETVDGLLDAMER

QGPPAELVADFALPVPSMVICALLGVPYADHEFFEGCSRRLLQGPGAADV

NEARIELEGYLGALIDRKRVEPGEGLLDELIHRDHPGGPVDREDLVSFAV

ILLVAGHETTANMISLGTFTLLNHPEQLEALRSGSTTTAAVVEELLRFLS

IAEGLQRLATEDIEVAGTTIREGEGVFFSTSLINRDTEVYENPETLDWDR

PSRHHLAFGFGVHQCLGQNLARTELDIALRTLFERLPGLRLAVPAHEIQH

KPGDTIQGLLHLPVAW

>CYP183B10(2513752686)Streptomyces sp. MnatMP-M77

MTTDVPISQAPGALPLLGHLLPLARDPLKFLTSLPAHGDLVHIRIGPMKA

LMVCDPGLTRHVLVNDRTFDKGGPAFDQGREVLGNGLPLCPHTDHRRQRR

LVQPAFHPARMPAYARLMTQQTDTITGSWTDGQTIDAYAEMQNIVAQGLV

ATMFADTLNPTAITEVLEDLGAITDGIFRRMITPPRLNTLPIPGNRRYYQ

AQSRLRQTMARATADRRSADTGHDDLLSVLLGSPDSPEDGHDQNLSDAEV

IDQLMAFFFAGIETAAATLAWALHALAQDPHIEEQLHAEADSVLAGAAAT

LDDVPRLEYTRRVLMESLRRHPPAWLVSRTTTTDTHLGTHPVRAGTAVFF

SPYLIHHRTDQYDNPEQFDPDRWSSSRHLNPPDGSFIPFGGGARKCVGDQ

FGITEGVIILATIAARWRLEPLPGRPVRPALALTLSPQRLYMRLRPRT

>CYP1469B3(2513752689)Streptomyces sp. MnatMP-M77

MAATIPDELTWPDSFPTRGHGLPPEMFDLLRRERPVAPVTFPSGHRAWLV

TRRDDITAIGSSRGFSRDLTCPGGQRIAGDDFNSVPGGIFNLDPPEHTTV

RRVVQPFFSPAAAAAIQPAIALAASDLATALGEGPNPADLHRAYAHPLAA

TLACDLMRVAPRQRRKIVPRLRSQVDYTTPAAKIDTSTRWMLDFAAEVIE

AKSADYDHRCEPRDPVEALIRAHQHGTITSEHLHATVMYLFVTSAEPVTG

PVTTGVYTLLRHGDQLARILRDPGLWPTAVAELLRLHHNGMTSMPRMALT

DTELHGVRIRAGDAVITPWVAATWDPEHYKQPERFRLDRSTHEDPAITFG

TGPHFCLGVNIARTYLHTALSTLFTHLPDLALAAKHEDIAWEPDTYLFTR

PTELPVTWS

>CYP154V(2532533423)Streptomyces zinciresistens K42

MTSSPITLDTTGRHLYAQADQLRAQGPAVRVTMPSNLTAWSVTRGDIART

LLVDPRVSKDARKSWPGYRPFAIPWLAAWVDVVSMFTSDGADHKRLKDLV

GRAFTARRIEAMRPAVEAIVTSLLDAMEALEPDQPVNLRAVFSYPVPTRV

ICDLFGVPDEQRPEMLRVIDAVLDTSASDETAAQIRDDMFAAMRTLIATK

RATPGDDMTSILLATHEAHGDRLSEDELVSTLILMIGAGSETAVSLINHA

VVELLAHPDQLAAAIADPTRWDDVTEETLRKHPPIMHLPLRYATADIDLG

EGVTIRQGDLILIGFGAHGRDPKTNPQPDHFDIDRDDRQHLAFGHGIHFC

LGAPLARLEARVALPALFARFPRLRLATDEDGLLPQPSFIGNDYRELPVV

LTTAQAGNTGAAA

>CYP156S1(2532533424)Streptomyces zinciresistens K42

MTASPRPPRLFGDAFALRPQDAYAELRAAGPVGWAEIAENVYALVVTSHR

AAITLLNDTTTYSKDSRRWAALNNGRVPPDSPVLALMAFRPSLLYADGQR

HERLRIALEECLARVDVHMLRETTQAHANWLIDRIAPRGHADLMVDFADT

LPLLIFADILGCPTQLAGRMVTACQGIISAGAAAAQAAEDFARILFELIQ

TKKQRPGADFTSWMLAHPARLTYEEMVHQLFCFVGAGTIPTAAWIASGLG

LLLEDDTYAGDLAGGTVTVRRAMEKVLWTRSPMANFSAHFARHATMLHGV

AIPEGEPVLISHAATGTDPALPPGLGYDNRSHLAWSAGPHRCPADGQASV

IAQTAIETILDRLWDLDLTDPDVPNRPGPFHQCPVSLNVSFRKQPADVLT

ATTSAGGTA

>CYP105AX(2532533687)Streptomyces zinciresistens K42

MSYPGGTPGWLVTRYAEAKELLVHKSFSARQEGIISPVPTELAYDGPAAP

GAFAKTDDPVHSKYRKLITGFFTVRRTRQLAPMIERIVHEQLDDLEKAGP

GADLVEVFTESVPSRVMCEMIGVPESERKSLQRHVETTGRLSATVPEALA

AVSAMSGFLARFVPARMDDPRDDVLGDLIRGGQLTEQELMGMTATLITGA

FDTTGNMLAMGVYALLEHPGELAKLKEDPELMGAAVEELLRFLTISHLGA

SRWALEDVEFAGRTVRKGEVVTVALPAVNRDPERYENPDQLDIGRTDHGH

LGLGHGVHQCLGQHLARTILRVGYEALFDRFPTLRLAVPADEIPMRDDFV

HYGPGSLPVTWED

>CYP170A(2532533772)Streptomyces zinciresistens K42

MTVESVSPEATPRAPSELREPPVAGGGLPLLGHGLKLARDPLAFMAQLRD

HGDVVRLRLGPKTVYAVTTPDLTGALALSGDFKIGGALWESLEGLLGKEG

VATANGPLHRRQRRTIQPAFRLDAIPAYGPIMEEEAHALVDRWKGGEPLD

CTSEAFRVAVRIAARCLLRGDYMDERAERLCAALATVFRGMYRRMVIPLG

PLYRLPLPPNREFNRALADLHLLIDEIVAERRASGQMPDDLLTALLEAED

ENGDPIGEQEIHDQVIAIVTPGSETVASTIMWLLHVLAQHPEQADKVCAE

VESVTGGRPVAFEDVRALRHTNNVILEAMRLRPAVWILTRRAVIDTSLGG

YRIPAGADIIYSPYAIQRDEKSYERNLEFDPDRWLPERAKDVPKYAMTPF

SVGNRKCPSDHFSMAQLTLVTAALAARYRFEQVPGSDDSTRIGITLRPND

LRLRPVPRDTP

>CYP107C(2532535212)Streptomyces zinciresistens K42

MTAQEPQPLTDPVPLMGCPYKADPYPLYERMRDAGPVHRVLFPSGVRAWL

VTGYDAAHAALNDDRLGKNHDRGNDRWRARASIMPEPQHSQLQVHLLHQD

PPRHTRMRGQVTDAFAPRRVDALLPRFQVLADRLVDALPDTGPADLVAGF

AAHFPFQVLAEVIGLPPELSARFDRDWGKVVQPVGPADPGRARYEARLHG

LQSYIADVVAHKRRQGDVDLLGRLTAACDQGELTPEERDSMIFQLLVAGQ

EPVTNQITTALVALFRNPRQLARLREDPALLPRAVEELLRHDSAFELTTW

RFFDRDSDLHGTPVPAGDSVIVSLCAANRDPRRFPDPDTLDLDRSPNPHL

AFGHGIHFCPGAALARAELRIALGTLLTRLPGLRLAVGDDDLAWIPAVLG

RGTRELPVAYDRRR

>CYP107CL(2532535362)Streptomyces zinciresistens K42

MQITEDFLRDPYPVYAGMRASAPVHLSDANTGRTWFVPRYADVIDVLRDD

RFSAALKAPGFINQFPPEQRAEFQPFNRSVAGWVVLQDPPAHRRLRQLMN

KGFTRQLVATLRPKVTEIAAGLIHDMAERRSGDFMTDFAQPFPAAVIATM

FGVPTADLSTFISWSDDIVLFAGSLRPTPQVARSAQHGLLSMTEFFRALL

PQRRADPGDDVISLLVSVRENGEQLTDEQVLANCAQLIVAGHETTRNLVA

NGLWTLLNHPDQLEKLKADPSLMTSAIREMMRFESPLQFVRRVAREDFTH

LGARIKAHTAW

>CYP1056B1(2532535371)Streptomyces zinciresistens K42

MRHVDFLSPELGDELWRVFARCRRERPVTWVPSVRMYCVFRHADIKTCLT

SPDFTVDYPFRVSRQVFGPTLLDFDGPRHTRLRRGLGSLLVGRDANTPFA

GPVARCVSDVLDGLAGRDDVDFVPAVARALPESVTAAFLGIPRGERDWVF

GHLRYLLAHLDGSSRDFAVATALRHEVRDLARRLLRDPHQDGRTVISRLG

ALVRTGELDLEDAVGMVLLVLAAGVETSTGVLANTMVALTRFPEWRARGR

ADEAVLGRVVREAIRWQPPQMDTVRFARTDTRLAGVPVAAGRPLKLLLGS

GNRDESVFADPDGFRPDREERAGLSFGHGPHSCLGTHLAVDLATRFFAAF

LRRFPDAAVVGTPPRIDGWTFRQPAALPMRLGGPDRTAVAATEGGHR

>CYP105B(2532535510)Streptomyces zinciresistens K42

MTTHHETAADIPDFPAPRSPACPFDPSPELRALSEHGPLTRVRSWGGTTP

WAVTGHAEQKALLSDARLSVDFALPGFPSPVDPRHSRRQSAGDLSFVGMD

DPEHTRLRRMVSGAFTIKRVEAMRASIQRMTDAFIDDILAGPKPVDLVQA

LALPLPSLVISDLLGVPYQDHDFFQTNSKTLVSAVTTGEQRQAAHAALAQ

YLDDLIGKKITAPGEDLLSKLGRHVTEGELTRRGAATMGVLLLLGGHETT

ANMISLGTLLLLRDPGQAAVIRHAENQQAVVNAVEELLRYLSIVHLGRRR

TALEDIEIGGQTIRAGEGVILLGELANRDPDVFENPDRLDLTRDSRLHQA

FGSGIHHCVGQPLARLELQILYPTLLRRIPTLRAAVPLEETRFKYDAVIY

GLHELPVTW

>CYP107U(2532535905)Streptomyces zinciresistens K42

MTDQPGPSVPGPSVPDSSAPGPSAPAPPALFTWEFATDPYPAYAWLREHA

PVHRTRLPSGVEAWLVTRYADAKQTLADQRLSKNPAHHDGPAHARGRTGI

PGERKAELMTHLLNIDPPDHTRLRRLVSKAFTPRRVAEFAPRVQELTDRL

IDGFADRGGADLIHEFAFPLPIYAICDLLGVPREDQDDFRDWAGMMIRHQ

GGPRGGVARSVKKMRGYLADLIHRKRAALPPDPAPGEDLISGLIRASDHG

EHLTENEAAAMAFILLFAGFETTVNLIGNGTHALLTHPAQRARLQRSLAA

GESALLATGVEELLRYDGPVELATWRFATEPVTIGGQRIAAGDPVLVVLA

AADRDPERFADPDVLDLGRRDNQHLGYGHGIHYCLGAPLARLEGQTALAT

LLTRLPDLQLAVEPAELRWRGGLIMRGLRTLPVEFTPNR

>CYP156B(2532535978)Streptomyces zinciresistens K42

MYGPQFAADPEATYTYLRSLGPSAPVDVAPSVQVELVTSYDAALYILQNP

ASFVRDSRRWNALNEGRVPADSPAVPMMGYRPNALFSDGAAHARLRQAVV

DSLATIDELQLRRKVQQSADYLISQFSSEPFGQAELMASYAMPLPLLVFS

DLFGCPPELGDRVIAGISGIFQGAAGADEVLGTALSELIALKHRQPGDDL

TTKLMQHPARLTDEEVLHQLVTLLSGGTAPLAAAVATSAALYLGEDAQAA

LPVEDAIAQTLWNFAPIANYAGHYPVHDVQLGARVLKAGDPVLISFAAAN

TDPKLAQHREQLSAKAHLAFGAGPHACPAKDPAFMIAVSAVESLLTGLPD

LEMRVPFKDLQWGEGPWSRSLASLPVRFTPRAATPAPSESARPAAGQQTA

TAAPHAVHAQQHAPQPAAQPRGMFSRFRAWLGGE

>CYP1035A(2532535979)Streptomyces zinciresistens K42

MVVEARTSFDRRSAVSLFARLRTAEGQADPFPFYAQLRAMGPVVAAPWGG

LLITGFDTCDRILRSREWLEPDQRWRAKQGPRTRWNAPSSQEMSNTLPAL

NAPDHTRIRRAAGTFGRAALERVGRTVSRTTDELLDSLCEQLREGEADFV

PTVSEELPVAAIGEWLGLPTADRVRLRELTHEQVFTQELLPTASQLARSD

AATAELRTYFMELVRQRRRRPGDDPVSLWIHTFDALEPDQDAADRAVYFL

ALFVLLAALETTSTLLSTMALRLLEQPLHWDLLSADPALVPAFVEETLRH

DPPTHVISRVAAADTVVEGVEVAKDAMVHLMVGAAHRDPARHPDPDRFDP

ERAKPDHLAFSNGIHYCLGAPLARLEAQTLLHHMVSRLPRLTLARRPSWA

PRVAFRRLENLDVALA

>CYP107AH(2532536289)Streptomyces zinciresistens K42

MTQAFASEESEALSFEEAAAAASSCSREFRADPHPVYATLREAAPVCPMS

PPHGVETYLITRYDDARAALADPRFSKDMYGALDAYHRIFGDSSIALDDN

MLFSDPPKHTRLRRIVGSTFTPKRVEALRPRVEQITAGLLDRCPTSAPVD

LLPEFCFPLPLQVICELLGVPENERQKAQEWSATVAKTGFGPEARAKLEI

AEGNLREYLVELCARKRREPDDGLLSALVTAKDQEGALTDHELVSTAWVL

LFAGHKTTAYQIGNTVHHLLGRPEQKAMALKDASALNAAIEEVLRFETSV

ENATFRHATEDVVLRDTLIPKGALVQISLAGANRDPEMFAEPNEMDVRRP

NVQATHLAFGFGPHYCVGAPLARLEMQTALTTLFARFPRVARAGDPGEAR

WMTVPFPAFRGLAELPVVLDPS

>CYP159A(2532537193)Streptomyces zinciresistens K42

MSTAPQAPDILSPEFAADPYPAYAVMREKEPLIWHEATKSYVISRYEDVE

RVFKDKKSEFTTDNYDWQLEPVHGKTILQLSGREHAVRRALVAPAFRGSD

LQEKFLPVIDRNSRDLIDAFRHTGSADIVTDYATRFPVNVIADMLGLDKA

DHARFHGWYTAVIAFLGNLAGDPEVTAAGERTRVEFAEYMIPIIRDRRDN

LGDDLLSTLCAAEVDGVRMSDEDIKAFCSLLLAAGGETTDKAIASIMANL

LLHPEQLAAVREDRGLIAAAFAETLRHTPPVHMIMRQAATDVEVTGGTIP

QGATVTCLIGAANRDERRYRDADRFDIFRDDLTTTSAFSAAADHLAFALG

RHFCVGALLAKAEVETGINQLLDAMPDIRLADGFDPVEQGVFTRGPQSLP

VRFTPAAG

>CYP159A(2532537194)Streptomyces zinciresistens K42

MSDHSGAGSAGAPPPGCPAHASAVPLGGLQYQQTPSELYRALRREHGAVA

PVLLDDDVPAWLVLGYPEVCYVTSHDELFARDSRRWNQWPNIPETWPLLP

FVGYQPSVLFTEGAEHQRRAGVITQALEGVDQFELARECQAIAEQLITLF

AGSGEAELMSTYAHALPARAVLWMCGMPQGSEDTEQLVDDLRISLDAGEG

DDPVAAYMRVGARLTQLVKEKRESPGPDVTSRMLLDAAALTDEEVVQDLI

SVIAAAQQPTANWICNTLRLLLTDERFAVNVSGGRVSVGDALNEVLWLDT

PTQNFIGRWAVRDTQLGGRHIREGDCLVLGLAAANTDPQIWPESHVGAEN

SAHLSFSNGEHRCPYPAPLLADVMARTAVETLMERLPDLVLAVETAELTW

RPSIWMRGLSTLPVRFTPSVH

>CYP107P(2532537905)Streptomyces zinciresistens K42

MAAPSDLAFDPWDPAFLADPYPAYAELRAAGRVHWFAPTRQWLVPHHADV

SALLRDRRLGRTYQHRFGHEDFGRTAPPPEHEPFHVLNDHGMLDLEPPDH

TRIRRLVSKAFTPRTVESLKPYVRRLAGDLVAALVEKGGGDLLADVAEPL

PVAVIAQMLGIPEADRAQLRPWSADICGMYELNPSRDAADRAVRASVEFS

EYLLELIAARRRAPGEDLISGLIAAHDEGDRLTEQEMVSTAVLLLNAGHE

ATVNATVNGWWALFRNPGELAALRADHSLIPSAIEELMRYDTPLQMFERW

VLDEIEIDGTTIPRGAEVALLFGSANHDPEVFRAPAALDLARADNPHISF

SAGIHYCIGAPLARVELAASMAALLEQAPTLRLTAAPERKPNFVIRGLEG

LAVEL

>CYP1194A(2532538614)Streptomyces zinciresistens K42

MTAEAPPRERRSPSRPRRLPMASGGGLLGHAAALQADAIGTLHRLASASE

GVMGFRIGGTVAATVSTPAAARDVLIENADDFGRGRRQTRALTPLMGDGL

LTSEGELHRRQRRLVLPHFSPRRIPRHAQAVVATAESLAGRWRAGVDVDL

VAEMNTLTMDIVSRLLFTASSSDNHALAEAITEAFAWEMHAITSPVALPL

WVPVPRNLRARRAMTGIRAWIARFVRERQEAAAAGAEVPEDILGDLLASR

YEDGTAMSEELLLDEVLTAWGAAQETSADAQAWTLYLLARHPDVLERVHH

EIGTVLGERTVTFEDLPRLPYCLQVFKEAMRLYPPAAVIPRQAVRDTVVG

GHRVKAGTMIFLNAYSLHRNPQVFADPERFDPDRFARERERTLPKGAYLP

FGTGGNVCPGSHLAMMEGHLLTVVLHQRLAFDLLPQGAEVTPELLVNLRP

SPGVRARVVAR

>CYP125A(2532538689)Streptomyces zinciresistens K42

MPCPALPDGFDFTDPDVLHHRVPLPEFAALRRTEPVRWIPQPGNVAGFQD

AGYWAVTRHADVKYVSTHPELFSSSLNTAIIRFNEHIERDAIDAQRLILL

NMDPPEHTRVRQIVQRVFTPRAIRALEERLRTRALTIAANARDRSGPFDF

VTEVACELPLQAIAELIGIPQDDRARIFDWSNKMIAYDDPEYAITEEVGA

ESATELIAYAMNMAADRKACPAKDIVTTLVAAEDEGSLGSDEFGFFVLML

AVAGNETTRNAITHGMHAFLTHPGQWELYKRERPSTAAEEIVRWATPVVS

FQRTATQDTELGGRRIGKGERVGIFYASANHDPEVFDEPDAFDITRDPNP

HLGFGGGGPHYCLGKSLAVLEIDLIFNAVADALPGLRLTGDPRRLRSAWI

NGVKELRVSAG

>CYP105D(2532540480)Streptomyces zinciresistens K42

MTDLTESPTVAFPQSRGCPYHPPAAYDSLREERPLARVRLYDGRPVWMVT

GHALARTLLADPRLSTDPQRPGFPATSERVARIRRRRTALLGVDDPEHRV

QRRMMIPGFTLKRATALRPRIQRIVDERIDAMIAQGPPAELVGAFALPVP

SMVICALLGVPYDDHEFFEGQSRRLLRGPTADDSMDARARMEAYFEELID

SKERQDTPGDGVLDELVHRQLRDGDLDREGVIALAVILLVAGHETTANMI

SLGTFTLLRHPARLAELRADPALLPAAVEELMRMLSIADGLVRVAAEDME

VAGETIRAGDGVLFSTSVINRDEAVYPEPDTLDWHRPARHHLAFGFGIHQ

CLGQNLARAEMEIALRTLFARLPHLRLAVPAEDIPFKPGDTIQGMLELPV

TW

>CYP102B(2532540579)Streptomyces zinciresistens K42

MAQTRTGDGLPKGFRSAEHGWPQLSRIPHPPYRVPLLGDVVGAGRRTPLQ

DSLRFARKLGPIFRRRAFGKEFVFVWGAGLAADIADESRFAKHVGLGVAN

LRPVAGDGLFTAYNHEPNWQLAHDVLAPGFSREAMAGYHPMMLAVAERLT

DHWDLAAARGQSVDVPGDMTKLTLETIARTGFGHDFGSFERSRPHPFVTA

MVGTLSHAQRLNTVPAAFLPRRAARRNQADIDHLDRTVDELIRARTAGGP

GDGDLLDRMLDTAHPVTGERLSARNVRRQVITFLVAGHETTSGALSFALH

YLSRHPEVAARARAEVDRVWGGTAAPGYDQVARLRYVRRVLDEALRLWPT

APAFAREAREDTVLGGVHPMRRGAWALVLTAMTHRDPRVWGPDAERFDPD

RFDPRAVRGRTPHAFKPFGTGARACIGRQFALHEATLVLGLLLRRYDFAG

DPGYRLRVAERLTLMPQGLRLRPARRAPAAAAPSGADAPGP

>CYP125A(2515898800)Streptomyces sp. So1WspMP-so12th

MRCPHLPEGFDFTDPDLLQARVPHPEFALMRQTAPVWWCAQPANISGFGD

EGYWAVTRHADVKYVSTHPELFSSNTNTAVIRFNETISRDQIEVQKLIML

NMDPPEHTRVRQIVQRGFTPRAVRSLEQALRNRARSIVETAHAAAAGTGG

SFDFVTDIAVELPLQAIAELIGVPQEDRSKIFDWSNKMAAYDDPEYAITE

EVGAEAAMEIVAYSMHLAAARKECPAQDIVTQLVAAEGEGNLSSDEFGFF

VILLAVAGNETTRNAISHGMHAFLTHPEQWELYKRERPRTTAEEIVRWAT

PVVSFQRTATQDVELGGQLVREGERVGLFYSSANNDPEVFEAPEAFDITR

DPNPHLGFGGGGPHFCLGKSLAVMEIDLIFNAIADVMPGLRLLEDPRRLR

SAWLNGIKQLQVSTPVG

>CYP1035A(2515899455)Streptomyces sp. So1WspMP-so12th

VSITGGTSRTPDGRRAVIGLLRRLNSPEGQAEPYGILAELRTMGDVVRAP

WNGYLVTGFDACSQVLRGRNWQAPDFAWQERQDDAKRWDAIATREMTSTL

ARLNAPEHTCQRRSLGNLFDRSTIERLTPDVERHADRLLDELADKLRWGE

ADFVSTVSEQLPIHTIGSWLGLPPEDYPHILEITHNQVYAQELLPTSSEL

AVSAKATAQLRAYFTDLVARRRAEPRHDVLTGWIHTWDALEPDRERADEI

LYRLTMFVTIASLETTATLLTSMVSLLSAEPGRWAWLRSCPEHIDAAVDE

VLRYDPPIHINTRIAAEDTVLAGVPIEKDSMVHVLYGAANHDPRRNPDPG

AFDILRGGSHLTFGGGVHYCLGAALAKLEARTLLARMLDRFPALRTVSPP

VHAPRMVFRRVTSLGVAL

>CYP156B(2515899456)Streptomyces sp. So1WspMP-so12th

MDPSPGATPYSAPAGCPMHQQQTSLYGPEFAADPHRVYDTFRAHGPAAPI

ELAPGVDATLIVQHEAALRVLQNPALFARDSRRWAALREGAIPMDSPVLP

MMMYRPNCLFTDGAEHLRLRKAVTESLARLNSSRVSRDVERIADYLIDQF

IERGSADLLNEYAKLLPLLLFNQLFGCPGDIGDRLTRSMSAIFDGEDVLR

ANAELTECLMELVALKRRQPGDDITSWLIQHPAGLRDEELKDQLVMLMGA

GVEPERNLIANALLLMLSGEQPGGHERRGSGMLVEDALDDVLWNNPPIAN

YATHYPVRDIELDGVVLKAETPVLISFAAANSDPSLTDARQTLSKGAHLA

WGAGPHVCPAKSPATLIALTAIEKILNTVPDLALAVPASGVAWRPGPFHR

ALVALPVRFTPTAARRTPAAQPPAPTSAQLPDPYRNAPPQPGAAARHPAE

PAKKQKGWWSSFLDVFRV

>CYP107P(2515899591)Streptomyces sp. So1WspMP-so12th

MHVSFDPWSPAFVADPYPAYAALRAAGRAHWFEPTGQWLIPHHSDVSALL

RDRRLGRTYLHRFTHEEFGRTPPPAAHEPFTTLNGQGLLDLEAPDHPRIR

RLVSKAFTPRTVENLAPTVRRLAAGLVDAFVARGGGDLLAEVAEPLPVAV

IAEMLGVPEEDRGPLRPWSAAICGMFELNPSEETAKAAVRASEEFSAYLR

GLIAERRTAPGDDLISALIAAHDEGERLTEQEMVSTCVLLLNAGHEATVN

TTVNGWRTLFHHPEQLAALRAAPASLPTAVEELLRYDTPLQMFERWVLDD

IEVDGQVIGRGAEVALLFGSANRDPERFAEPDRLDLGRVDNPHITFGAGI

HYCLGAPLARLELEASFGELLRKAPAMRMVAEPEWQPGYVIRGLKELVVE

V

>CYP1046A(2515900723)Streptomyces sp. So1WspMP-so12th

MIEEGGPGGCPASREEPRVAHSRTLRFWLDPANIAVRLGQAGPVARTKAG

PAVAFQVNDPSLLRKVGCGEDTFQAWSADPCLRDFTGNGFAGSEGQPHRD

RRALMKPALAAPRLTALGPSVRDSADRLIADLPTDRPLDIPFEMSRLVAG

LVIETVLNSAISPDVLTRLARARSVLSAGVFWRYALSPWPWVPTPRRRAF

RRALATLDEAAREVCAGHRPDADGNDVVSLLKRSADDRPDTALHDVRALL

FAGIEATASTFAWACYELGRHPGHQTAIRAEADAVLGTGAPADAVQPRLL

PRTAGFVSEVTRLHGIPFLVRRPRRPTYVGDQQVPAGALVTLPLGALRRD

PARYAQPEEFDPLRWTPEARPPLSPAALFPYGLGPRYCPGAAASEIMVPV

ALASLVRSRMLRTAPPGGTVRVSLELTPMPKGLTMVAAPR

>CYP157A(2515900754)Streptomyces sp. So1WspMP-so12th

VTTTSDTTGPTSSGPISSGPATASGTAASDPTAATGRTATTGTADSGATA

SATATGTTTSGATSPATTPSGCPVAHGSVPLSGPRFQSDPVQLYRDMRRD

HGAVAPVVLDGGVPAWLVLGYRELHQVTGDPVLFSRDSELWNQWDRIPAD

WPLLPMIGRGQNSILYTVGERHSVRAMMISNALEGVDPFSLKRYAEEFAD

ELIDRFCTKGAVDIIAEYAKLLPALVLARIYGFSDEEAYPLVGAINDMID

GRERALAGQQHLATSMFRLLADKHAEPGDDVASRMLADTGGFTDEEVAQD

LMVMMAAGHQPTADWMGNSLRLMLTDERFAASLSGGRHSVAEAMNEVLWE

DTPTQNVAGRWASRDTHLGGRHVRAGDLLLLGIAAANGDPQVRTHASALT

GGNNAFLSFGHGEHRCPFPAQETSEVIARTGIEVLLDRLPDVDLAVPAEQ

LTRRPSPWLRGLTDLPVLFTPTPALGRPGSHGGPA

>CYP154C(2515900755)Streptomyces sp. So1WspMP-so12th

MTRIALDPFVSDLDGESAALRAAGPLAEVELPGGVHVYAVTHHAEARALL

TDSRVVKDIDVWNAWRRGEIPMDWPLIGLANPGRSMLTVDGADHRRLRTL

VAQALTVRRVERLREGIEALTNASLEKLAAHPAGEPVDLKAEFAYPLPMN

VISELMGVDAADHPRLKELFEKFFSTQTPPEEVPQMMADLGTLFTKIVND

KRANPGDDLTSALIAASENGDHLTDEEIVNTLQLIIAAGHETTISLIVNV

VEALQTHPEQRKKVVNGEIGWEGVIEETLRWNTPTSHVLIRFATEDIEVG

DRVLPKGEALIVSFAALGRDERQYGPTAGEFDATRTPNRHIAFGHGPHVC

PGAALSRLEAGIALPALYERFPELELAVPASELRNKPIVTQNDLYELPVK

LGCPFGHEG

>CYP105D(2515900887)Streptomyces sp. So1WspMP-so12th

MTESTADPTTRRAPGPTAPATAAAVGPAPATPFPQDRGCPYHPPAGYAPL

REDRPLSRVALFDGRPVWAVTGHALARRLLADPRLSTDRTHPDFPAPAPR

FANANRRRVALLGVDDPEHNTQRRMLIPAFSVKRINALRPRIQETVDRLL

DAMERQGPPAELVSAFALPVPSMVICALLGVPYADHEFFEERSRRLLRGP

GADDVDRALDELEEYLGALIDRKRTDPGDGLLDELIHRDHPGGPVDREEL

VSFAVILLIAGHETTANMISLGTFTLLRHPEQLAALRAGGTTTAGAVEEL

LRFLSIADGLQRLATGDIEVPDAGVTIRKGEGVLFSTSLINRDDDVFPRP

ETLDWERPARHHLAFGFGVHQCLGQNLARAELDIAMRSLFERLPGLRLAV

PAQEIPHKPGDTIQGMLELPVAW

>CYP107F(2515901213)Streptomyces sp. So1WspMP-so12th

VENTSVQNAPAQDKETVRSCPFDFAERLEFDPQLKELLTEEPVSRIRMAY

GEGEAWLVTRYEDVRTVTTDRRFSRGAVLGRDFPRMTPEPIVQAESINLM

DPPASSRLRSLVAKSFTPRRVEQMRPGTQRVVDRLLDEMEAEGSPADFVA

RVSSPLPLITICEALGIPEADRPWLRAHAMTMMSVGAAGKEDAVRAKAEL

RGYFTELTAERRRSPGQDLISTLATARDGDELLDDKELAVMAMVLLITGQ

DTTTYELGNIAYTLLTRPGLLGTLRAEPGRLPRTIDELLRYIPFRKGVGI

PRIALEDVELGGVTIRAGDVVHVSYLTANRDGAKFDRPDELDPDRPSIPH

MTFGWGAHHCLGAPLATMELEVAFTTLLARFPGLRLDAAPEDIRWNTTSI

WRYPLALPVTW

>CYP159A(2515901590)Streptomyces sp. So1WspMP-so12th

MTVRTPDILSPEFERDPYRAYRRMRDDEPLIWHEATNSYIISRYEDVERV

FKDKKGEFSTENYDWQIEPVHGRTILQLSGREHAVRRALVAPAFRGSDLR

DTFLPVIERNSRELIDRFRTTGSVDLVTDYASRFPVNVIADMLGLDKSDY

DRFHGWYTAVIAFLGNLSGDQEVIRAGERTRVEFAEYMLPIIRERREAPG

NDLLSVLCTAEVDGVRMSDEDIKAFCSLLLAAGGETTDKAIAAIFANLLR

HPDQLAAVRADRELISRAFAETLRYTPPVHMIMRQSTTEVALSGGTVPAG

ATVTCLIGAANRDGNRYRDPDSFDIFREDLAATNAFSAAADHLAFALGRH

FCVGALLAKAEVEIGVGQLLDAMPDIRLADGFDPVEHGVFTRGPQSLPVR

FTPISG

>CYP157B(2515901591)Streptomyces sp. So1WspMP-so12th

MSTSSPAFDPATAPGCPAGRGAVRLSGTSYQQTPTELYRSLRSEHGAVAP

VLLDADIPAWLVLGYAELSYVTEHDELFARDSRRWNQWENIPADWPLLPY

VGYQPSVLFTEGDEHRRRAGVITEALETVDQFELARDCRHIADQLISAFA

GSGRTELMSQYVHALPMRAVVQLCGMPVSGDDTAQLVDDLRVSLDAGEGE

DPVAAYGRVGDRLRQLVQERRAAPGPDVASRLVTHPEGLTDEQIVQDLIS

VIAAAQQPTANWICNTLRLLLTDERFALNVSGGRLSVGEALNEVLWLDTP

TQNFIGRWAVRDIQLGGRHIRAGDCLVLGIAAANTDPEIWPESYVGAENA

AHLSFSNGEHRCPYPAPLLADVMARTAVETLLERLPDLMLAVEPEALRWR

PSVWMRGLSELPVVFSPVSQ

>CYP107U(2515901633)Streptomyces sp. So1WspMP-so12th

VNDTPACPRTAAPESAPELFTWEFATDPYPAYAWLREHSPVHRTALPSGV

EAWLVTRYGDAKQALADARLSKDPANHAGSAAAKGKTGIPGERKAELMTH

LLNIDPPDHTRLRRLVSKAFTPRRVAEFAPRVQELTDRLIDDFVEKGEAD

LIHDFAFPLPIYAICDLLGVPREDQDDFRDWAGMMIRHGGGPRGGVARSV

KKMRGYLAELIHRKRENPGDDLISGLIRASDHGEHLTENEAAAMAFILLF

AGFETTVNLIGNGTYALLRHPEQRARLEESLAAGESALLATGIEELLRFD

GPVELATWRYATEALTLGGQEIAAGDPVLVVLAAADRDPERFTDPDTLDL

SRSDNQHLGYGHGIHYCLGAPLARLEGQAAVATLLKRLPGLQLAAEPADL

RWRGGLIMRGLRTLPVQFEPGRRLGEGDTLSPL

>CYP124G(2515902001)Streptomyces sp. So1WspMP-so12th

MTVTDQVSDDRAAHRDLADPAFWRLPRPERLAAFARLREREAPVLFTPRP

GTARTSGKPFYALVRHADVLTASRTPKVFASAPGATTPEPAGWAKALFGN

SMVNMDGAEHAALRRIISRRFTPRLLAATEENIGRLAGRLVDEMIAERPT

DFMPSAASRLPLEVICDLMGIPAEHRPRIAAQIDHASEQVGVERRGRLRI

PGQGTASLAMMQLVMARLARERRRRPQDDLVSALVRADVDGEGLSSRELG

AFFSLLLVAGVETTRNAIAHGVSLLDRHPEQRELLRSDFDRYIGGAVEEI

VRHSTPIIQFRRTVVSEFELGGRTFLPGEKVALLYASANRDEAVFTRPDL

FDITRSPNPHLGYGGGGPHHCLGAHLARLEMTALFRELLTRRTVIRRTGE

PGLVDSNFDNRVGSLPLSMGPTVT

>CYP159A(2515902259)Streptomyces sp. So1WspMP-so12th

MPADRSEALPTARRPHRLAGGQPGILEPGASTDPYRLRLYRVLRTDFPLG

HDPGLGAWLLSRYEDVVLALTDPRFTGYPHDGAPRGPVPAPLGLCRGSLV

CTPLPQGGTAPAGRETPDPRPETRDPRAETRDLRAESPDPRAASAGPVPA

SAPAPAVERAAYVLARRIAGRDQADLVGEFCRWLPAGAGAAAGLSSPAPA

ASPRPGGGSDDCLRQAGLRERALASFLANMLDDPDLLAAVAGGGAGAGML

VRRAWAETLRRDPPVQIVLRRTRTEVRVSGGTLPAGEAVACLIGAAGRDP

ARFAAPDRFDPLRTDADPLFSGPAGCPAVLLGLLEAEHGLRALLTAMPGI

RWAEGFRPTASGVLTRGPRTLLVRPS

>CYP107L(2515902407)Streptomyces sp. So1WspMP-so12th

MTMLDLRELPDFTANPYPYYAKLRAEGPVHTVRTEQMERIWLIVGYEEAR

AALADHRFGKDWRTGGRWADEVNPISSNMLELDAPHHTRLRRLVAREFTP

RRIEALRPRVTEITTGLLDAMVPAGSADLVDALAFPLPMTVICELLGVPD

IDRDAFRALSSAIVTPTPAQRESADPVGAMSDYLVQLIREKRNSPGDDLM

SALIRTRDEGGDGLSGEELVGMAFVLLVAGHETTVNLISNGVRALLDHPD

QLALLRADPGLLEGAVEEMLRYDGPVETATFRFTREEITVGSTVIPYDEP

VLVALASGGRDPEKFTDPDTFDIRRAPQGHLAFGHGAHYCLGAPLARMEA

KIAIGALLERCPELARDPAGGEPEWLPGLLMRGVRRLPVRW

>CYP157A(2515903219)Streptomyces sp. So1WspMP-so12th

MTNPSPATPAPSAGGGGGCPANFGTGAIPLSGPGFHTEPHALYRSMRRDH

GPVVPVELPGGFPAWLVIGYRELHQVTSDGELFPRDVSLWNQWENIPADW

PLLPMVGTPMPSIYFTAGAEHRRHADMVVPALEEADPFEIRQHCEQLADR

LIDAVCSRGTADLVAEFAEPLPVLVLARLVGFPDDEGADIARVLKDLADG

GPDAQKAHLRFGEHMHRLVATKRAHPGNDVTSRMLAHPEPFTDEEYALDL

MAVTAAGHLPTADWISNSTRLMLTEDQFADALSGGRHSVAEAMNEVLWED

APTQILAGRWAARDARLGGQNIVRGDMLLLGLGAANGDPHIRQQVTDLLV

RSGQGGNNAHMAFSHGEYRCPFPAQEIAEIIARTGIEVLLDRLPDLELAV

PATELVRRPSAFLRGTTSLPVRFTPVRTTGDAL

>CYP154C(2515903220)Streptomyces sp. So1WspMP-so12th

VNCPHAAAAPAAEAGRGAGAVVIDPMVQDLDGETARLREAGVLARIELLG

VPAWTVTRHAEARQLLVDQRLVKDIEAWELWRTGVVTRAWPLIGMIDAGR

SMFTVDGAEHRRLRTKTSQALTPRRLEAIRPAIEKFTDELLDNLEAARGE

DGVVDLKAVFAQPLPMKVVGMLMGVDESEHPMLTRQYKAFFSMLTPQAER

LKLLADLDVFYADLVREKTARPTDDLTSALILAEEGGEPLTEEEVVGNLK

AMVAAGHETTIGLILNAVRALLAHPDQLARVLAGEVGWDAVVEETLRWDT

PTTHLLMRFATEDITVGDTVIRKGEGVVISYRAIGRDTGQHGEDADAFDI

TRPTRNRHMTFGHGPHICPGAALSRVEAGIALPALFGRFPGLRLAVPDAE

ITKLPVMTQNDMAAFPVLLG

>CYP1047A(2515903227)Streptomyces sp. So1WspMP-so12th

MSTQTGPALGTTSTGPVPVPGPRGLPLLGNLPQFGKNPLAFFELLRGHGD

LVRWRFGRNRCLFVSDPAHIGELLTETERTFDQPSLGIAFRTVMGDGIIV

ARGREWRRKRSLVQPSVRPRQVTSYAATMAASAVELADSWSDGERVDIKR

EMAALTQRIAVRTIFGVDTPADSEAMGRAMDVAQAEIGKEFAGIGALLPD

WVPTPGRARIRKAAAVIDAEVARVVARHRDGETERPDLLSRLLTAVDESG

ERLSDEEIRDETVTLYIGGHETTSSTLVWAWYLLSRNPRVRAALAEELDR

VLGDREPGIDDYARLPYAQAVVKETLRLYPTIWLITGVAKEGARLGGMPV

EEGTRVWSSQWSTHRDPRWFPEPEEFRPERWDPEEGDEIAEYAWFPFGGG

PRVCLGTRFAMVEAVLVLAVLARRFVLDVDPGTVEPVPSLTLQPDRDVLA

TVRTR

>CYP157C(2515903245)Streptomyces sp. So1WspMP-so12th

VTTPFHHEPGAVPPPQCPAHNLGTGPGGLRRLYGPEAENDPAGLYDKLRA

EHGTVAPVLLHGDVPAWLVLGHSENLHLTRTPSQFSRDSRRWRALQDGSV

APDHPLAPIFTWQPICVFADGAKHERQRGAVTDSMERIDTRGVRRHINRF

SNRLVNDFCEKGSADLVGQFAEHLPMMVMCAIFGMPEEYDERLVQAARDM

TRGTATAVASNAHVVAVLTRLVERRRAEPAPDFATWLVEHPATMTDVEVV

EHLRLILIAAYESTANLIANVLRMVLTDPRFRARLSGGHMTVPEAVEQTL

WDEPPFTAVFGRWAVGDTELGGQQIKAGDALLVGIAPANTDPAVRPDLAA

DMGGNRAHLAFSGGPHECPGQDIGRAIADVGVDALLMRLPDLQLGVEEAE

LRWVGNIMSRHLVELPVKFAPSPQQKLDADPLSVMARPARPAGDWEISSP

ARPVPEPAHSLAGTQPAHAPGGAPLPRPAPPAEGAGGQPAPGTAVVPAQR

RPAAPARLWQAVARWWKGY

>CYP154D(2515903377)Streptomyces sp. So1WspMP-so12th

LKPSPQNPTADRPHRLDPAGGCPHAANARLLAQGAVTPVVLPGEVEGMAV

LGHDALKEFLAHPDVAKNAQHFTALQAGEIADGWPLKTFATVQGMTTADG

DDHRRLRSLMGKAFTARRVEELRPSVEELTSRLLNGLEAAASEDGVVDLR

AHFALPLPMGVICELLGVDEAHQDRLHDLSNKIVATDIGPAEAMAANREM

VEVLSAVAAARTADPGDDLTSAMIAAREESGDRFSPQELIGTLMLTIIAG

HETTLNLITNAVRALCTHRDQLGLVLSGGASWADVVEETLRWDSPVSYFP

FRYPTRDLTLDGTVIPQGTPVLAGYSAAGRDTLAHGPDADRFDITRTGTS

RHLSLGHGAHYCMGAPLARLESTTALEQLFTRFPALDLAMPDAELPRHAS

FVGNSVRKLPVRLKG

>CYP157F(2515903378)Streptomyces sp. So1WspMP-so12th

MSTPHPTAGPAARCPVTGAPAKAAALYGPGLDGNTMPALYEELRGTHGPV

APVSLAPGIDAWLVLGHRELLRLTREEQDFSHDPRRWSLLREGRVPADSP

ILPMVGWRPALLFADGQQHRRMRGAVSDALAGINGHELRRSVRTTAEALI

AGFADDGEADLVASYARMLPLRVIAGLLGVDDRTGRELVDAVGGLASGAS

GAAEASRRMGAILLSLIEEKRRVRGEDIVSALLHHPARLTDEEVLHNLVV

MFVAGNQTTVNWIATTLRILLCDPALRSSLSGGHLSVDDALDLVLWRFPP

TQNFPARYATRDMRFGEQDIRAGDMLILGLAAANADPDVLPADGAPVVGN

RSHLAFGAGPHTCPAQDPARLITRTAVDTIRHRLPDLELAVPENELKWVN

SPWSKGLGTLPVRFTSPQLPQLPVPSQAIEAPATPWPYRDGTRPQARPH

>CYP107BX(2515903921)Streptomyces sp. So1WspMP-so12th

MTTTDPSLIPLHRLRFEEPGPPRPGELPTGAPAWLVSRYADVRQVLSDPR

FGRSRLYAEDAPALSGVPDLVNNPDLMFNQDGPDHLRLRRTLRRAFTPRA

VARWRPWISAIVDELLDRLEERPRPADVVREFTLPLPVAVISRLMGLDGS

ARDRMRHWSEHAFSDGSHAGEEVESVLKEFSAFGADLLAARRHTPGDDLV

SGLVRAADEEGGLPEAQLVSLVCGLVVGGHDSTMTMLGNGLLYLLGERPE

SWPRIGADEEAAGRVADRLIHLVPLGDDRGTARHAAADIEVGGVTIPAGA

IVLADCGAANRDPDVFPRHTLDDLFAPLEAPTLSLGAGAHYCLGAWLART

ELQLALHRLAARFPELRLAEPVERVTWRTGTTSRSPRRLTVSW

>CYP170A(2566312130)Streptomyces sp. GXT6

MTVESVKPGIRTEGGRHRGEPPLAGGAVPVLGHGLKLVRDPLAFMAQLRD

HGDIVRLRLGPKTVYAVTAPELTGALALSPDYIIAGPLWESLESLLGKQG

VATANGALHRRQRRTIQPAFRLDVIPEYGPIMAEEAHALVERWRSGEVLD

VTAESFRVAVRVAARCLMRGSYMDARADRICSALASLFSGMYQRMVVPLG

PLYKVPLKANREFNRALADLHLLVDEIVADRRASGQKPDDLLTALLEAKD

DNGEPIGEQEIHDQVIAILTPGSETVGSTIMSLLLVLTEHPELGDKIRDE

VKTVVGDRPVAFEDVRKLTYTAHVVVETMRLYPAVWILTRRTVAETELGG

YRIPKGADVIYSPYAVQRDPRSYDRHAEFDPERWQPERAKQVPKFAMVPF

SVGNRKCPSDHFSMAELTLLTAAIASAYRFEQAPGSDPRPRIGITLRPRD

LRLRPLPR

>CYP107P(2566312980)Streptomyces sp. GXT6

MRSAPPGQAPVGRASVPGMAAVRDLAFDPWDPAFVADPYPAFAELRARGR

VLYYEPSDQWLVPHHADVSALLRDRRLGRTYLHRFTHEEFGRTPPPPEHE

PFHVLNDHGMLDLEPPDHTRIRRLVSKAFTPRTVERLKPYVHRLADELVA

ALVREGGGDLLTDVAEPLPVAVIAEMLGIPQADRARLRPWSAAICGMYEL

NPSAETAAKAVRASVEFSDYLRHLIAERREQPGDDLISGLIAAHDEGDRL

TEQEMISTAVLLLNAGHEATVNATVNGWWALFRHPGQLAALRADHALIPS

AVEELLRYDTPLQLFERWVLDDIEIDGTTIPRGAEIAMLFGSANHDPAVF

TDPERLDLTRRDNPHISFSAGIHYCIGAPLARIELAASLAALLRQAPTLR

PAEEPQRRPNFVMRGLTGLRVEVG

>CYP107U(2566313715)Streptomyces sp. GXT6

MHDQPPTAPTPGDTPPTLFTWEFASDPYPAYAWLREHAPVHRTRLPSGVE

AWLVTRYADARQALADQRLSKNPAHHDEPAHAKGKTGIPGERKAELMTHL

LNIDPPDHTRLRRLVSKAFTPRRVAEFAPRVQELADGLIDTFAGRGTADL

IHEFAFPLPIYAICDMLGVPREDQDDFRDWAGMMIRHGKGPRGGVARSVK

KMRGYLAELIHKKREALPAEPVPGEDLISGLIRASDHGEHLTENEAAAMA

FILLFAGFETTVNLIGNGTYALLTHPEQRHRLQDSLAAGERELLETGVEE

LLRYDGPVEMATWRFATRPLTVGGQDIAAGDPVLVVLAAADRDPERFTDP

DTLDLARRDNQHLGYGHGIHYCLGAPLARLEGQTALATLLTRLPDLRLDA

DPADLRRRGGLIMRGLRTLPVRFTPRPGPPAEGPRSRAR

>CYP157C(2566315046)Streptomyces sp. GXT6

VTSDTSFPAGPGDRTLDPPPGCPAHGLGPGGLHRLHEAPDLRELYERLRE

EYGPVAPVLLHDDVPIWVVLGHAENLHMVRTPAQFTRDSRIWTPLRDGMV

KPDHPLMPHIAWQPICSHAEGDEHKRLRGAVTAAMQTIDPRSLRRVIGRA

TQRLVNRFCETGRAELVGQFAEHLPMAVMCEILGMADEYDDRIVQAARDM

LKGTETAIASNEYIMDCLRRLVARSRSRPGEDFAGHLIRHPAGLDDDEVA

QHLRLVLIAAYEATANLLANTLRRVLTDPGFRAQLNGGQMTVPQAVEQSL

WDEPPFSTIFGYFAKQDTELGGRRIREGDGLLLGIAPGNVDPRVRPDPTA

DMQGNRSHLAFSGGPHECPGQDIGRAIADVGVDMLLMRLTDVRLDCAEED

LRWTESIASQHLVELPVRFTPQPQQDVKSLPTHTPMPVPQPRAAWEISTV

RAASGAHPASEAGAAPDGAPGERRRAAWPGPEPAPAAVGAVRTAAPVGKA

GAAEGGPDATHLPPEAGRGRPRGPWQRLVRWWRGH

>CYP158A(2566315418)Streptomyces sp. GXT6

MTAEPLVEQAAVDEALPAGPPVEQARAECALLARPPAATTGAAEPDTASL

PPVRHWPALDLTGVDFDPVLADLMEEGPVTRIELPNGEGWAWLVTRMRDV

RTVANDPRFSREAVMDRQVTRLAPHFIPSRGAVGFLDPPDHTRLRRAVAP

AFTARGVERVRESARRMLDELVDALEAAGPPADLTAAVLSPFPIAVICEL

MGVPPADRDAMHTWTQLILSSAHGADVSERAKEEMGAYFTELIDARADST

GEDVASLLGSAVGRGEMSVAEAVGLAVLLQIGGEAVTNNSGQMFYILLTR

PELADRLRADPQIRPRAIDELLRYIPHRNAVGLSRIALEDVEIEGVRIRA

GDAVYVSYLAANRDPEVFADPERIDFTRSPNPHVAFGFGPHYCPGGQLAR

MESQLLVDALLDRLPALRLAVPPDQVPFRKGALIRGPEALPVTW

>CYP158A(2566315598)Streptomyces sp. GXT6

MTQEITTPTPARATPHGSAPVAPSPAGPPAPPPVRDWPALDLEGTAFDPV

LAELMREGPLTRVRLPHGEGWAWLATRYDDVRLITNDPRFSRAEVTRRQV

TRMAPNFAPRPGSLAWADQPDHNRLRKPIAGAFTVSAMKRLRPRAQEILD

ELVDGVLRDGPPADLVERVLEPFPIGVVCEVMGVPAADRARVRAWTREII

STNGAEAAGRAKEGLYGWITATVRARADRAGEDVYTLLGRAVARGEITEE

EAVGLAGPLQIGGEAVTHNCGQMLYLLLTRPELMARMRRRPEARGPVLDE

LLRWIPHRSSVGLARIALEDVDLHGVRIAAGEAVYVSYLAANRDADLFPD

PDAIDPDRDPNPHLAFGNGPHYCTGAVLARLQTELLINTLLARMPHLALA

VPADRVPWRRRTMIRGPRSLPVTW

>CYP152D(2566315826)Streptomyces sp. GXT6

MDATPRPTRRPLIDRTLAASATGYTWLPRRMRAGPDRVVRTRLLGRPALA

VRGPDAVRFFYDEHNVRRHGALPEPVRGTLFGQDAVHTLDGAAHRQRKRF

FLPLLQADRIAGAVEQVTRAWDEAVADWARRPRVVLFDEAAVVLTRGVHR

WAGIPLADAEAAQVAADLVAMVDGFATLGPRHWRARRARGRQEERLARLV

RDVRSGRTTAEAGSLLERAAGHRDTKGEPLPGEPLTERTAAVELLNVVRP

TAAVAWFVVFAAHALHHRPEVARRLREGDPVFATAFAHEVRRFYPFAPFL

GGRAAQDLSWHGRSVPAGGVVLLDVYGQLHDEDLWGDPYVFRPERFQQHP

AAGDELIAQGGGDPRTGHRCPGEGVVLGLLQALAVRLARLEYEVPDQDLR

ISLRRVPTRPRSGFVISAVRPPGRPTAAAATAQTA

>CYP147F(2566315864)Streptomyces sp. GXT6

MTDASLLRRITDFSSRADPYPLYEELRRTPVLHEEEGGPYAVSSYYDIVA

LLHDPRISSDARNLAAPGEDELSGAEGTGLPPSFIRLDPPEHDRLRRIAN

SSFGPPHWPHRIDGLRGELSAIVGELIDDLADGLAGGGEVDVVDQFAYPF

PVTVICRLLGVPREDEPRFRTWVDPIVAALDPETRNDPGAPAQRAAQEAR

MQLGMYLAGLVEERTREPRDDMLSDLVAGHGPDGSMSMMEVLSTAVLLLI

AGHETTVNLITNGTLTLLRHPEHLQRLREDPGLSVRIVEELLRYEPPVQL

VPSRTCVTDIEVRGVTIPKGSRIWLMLAAGNRDPERFKDPDRFDPDRQDI

EHLGFGSGVHICFGAPLARLEAQIALTALARRLDGPRLLQDPPPYRQSPV

LRGPRHLNIGVDGVRPRQAH

>CYP105B(2566315922)Streptomyces sp. GXT6

MTTASLPPLPTRPPSGCPFDPPEGLARLRVEEPLSKVALDDGTWAWLATR

YADVRAILGDARFSSDTSTPGYPISGMTGGSPRPGATRGFIRMDPPEHTR

LRRMVTRDFMVKRVEALRPTLQKLTDELCDAMERVDRSEHPVDLVKALAL

PLPSLAISLLLGVPYEDHDTFQRLTGALLSREISEEDRGPARTELLAYID

NLVETKKAAPGDDIISRLITEQYAKGELTHEDLIAFAVLLLVAGHETTAN

MIGLSALTLMLDRETADRLREDPSLVRGAVEELLRFHSIIRNGPRRAALE

DVEVGGQLIRKGEGVIVAVPSANRDEDTFPDAGRLDITRPNAQHHVAFGY

GIHQCLGQALARAELQVVITTLLRRFPGMRPAVPVEEIPFRTDMVIYGCH

ALPVTW

>CYP154A(2566316046)Streptomyces sp. GXT6

MTEHPLLVLDPTGSDHHAEHRALYARGAAARVDILGVSAWAVTDPALLKR

LLTSPDVSKDAARHWPAFAETATTWPLALWITARNMFTAYGADHRRLRRL

VAPAFSARRVEALRPAVEALVAAAVDRLASLPPGQSVDLREHLAYPLPIA

VIGRLMGVPDHQRDGFRAMVDGVFDTTLTTEQAAANTAALYQALDRLIAV

KRAEPGDDMTSLLIATRDDEGDGGGLSDTELRDTLLLMISAGYETTVNVI

DQAITALLSDPELLAHVRAGRAGWSDVVEETLRHEPAVKHLPLRYALKDI

ALPDGRTIARGEAILASYAAANRHPDWHGPDADRFDPTRPVKDHLAFGYG

VHFCLGASLARLEVATALRMLFERFPGIRLAVPVAELRPYPSLISNGHRA

LPVVLRPADH

>CYP105CD3(2566317091)Streptomyces sp. GXT6

MTAAVPMPHNRRPAGCPFRPDPALEQLRDDPELPRVPTFNPQLGNFDAVL

VTRPDQVRQALADPRYEAGFAFDRIGPRTVMNQPGILLNYDGDEHTRYRR

MLSGAFTVKRVRSLAPAIRRVVDERLDALEQAGPGADLIETFAGPVPLLV

ICELLGIPAEDRDGIQRRSATGTDVANSLETQLENFAAMAAYMGELIMRR

RREPSDDILGDLIRRHGHELSDDELIGMGNSILVAGHETVSSMIGLSTLA

LLRDEEQLAVVRDDESASAGAVEELLRLLSVAPPLVRQAAEDLELGGRTI

KAGERVLLSTLAADHAPELVPDGPGRLDVRRRPVAHLAFGYGAHQCIGQQ

LARLELQIALPALLRRLPGLRPGVAFEDVEYRDDALVFGVARLPVTW

>CYP107L(2566317150)Streptomyces sp. GXT6

MADLIDLTEYGEGFRADPHPVYAELRARGPVHRVRLPEPESYDTWLVVGY

EEARAALADPRLAKDAGAVEGTYREEEVIGKHLLIADPPQHTRLRALVTR

AFTARRVEALRPRVQQITDELLDAMLPRGRADLVEAFAYPLPITVICELL

GVPEMDRAAFREMSNEVVAPTSEEASLRAVERLGAYLTGLIEDKRAAGPA

DDLLGALIRTTAEDGDRLSAGELRGMAFLLLIAGHETTVNLITNSVHTLL

THPEQLAAVRADLDLVDGAVEETLRYEGPVENATFRYAAEPLEIAGVAIP

KGDPVMIGLTAAGRDGSRYAEPDRFDPHRDTRGHLAFGHGIHFCLGAPLA

RLEARTALRALLRRAPGLALDGPPGAWLPGMLIRGVRSLPVRW

>CYP107L(2566318214)Streptomyces sp. GXT6

MTAAEVIDLVAMGEDFVRDPYPVYAALRERGPVHKVRIPEGTEAWLVVGY

EAGRAALADPRLSKQWKNASPSVPFPSPAAGPHMLNSDPPDHERLRKLVV

REFTPRRIEQLAPRVRQITDELLAEMLALPEGRADLVEALSFPLPISVIC

ELLGVPMLDRAAFRAWTSTVLTDPDPAARLAATAETSAYLVDLLERKRLS

PGPDLMSALIRTTDEDGDRLSVDELRGTAWLLLVAGHETTVNLISNGVLA

LLTHPEQLAALRADMTLIDNAVEEMLRYDGPVETSTFRFTTEPVEIGATV

IPGGGELVLVALADADRDPARFPDPGRFDIRRPAGGHVAFGHGIHYCLGA

PLARLEARIAIRSLLERCPDLALDAHPAALNWRHGMLIRGPRQLPVRWTS

PA

>CYP125A(645210978)Streptomyces roseosporus NRRL 15998

MRCPHLPEGFDFTDPDLLQDRVPHPEFALMRETAPVWWCTQPPNISGFGD

EGYWVVTRHADVKYVSTHPELFSSNTNTAVIRFNETISRDQIDVQKLIML

NMDPPEHTRVRQIVQRGFTPRAVRSLEAALRSRARSIVETALASADADGS

FDFVTNIAVELPLQAIAELIGVPQDDRSKIFDWSNKMAAYDDPEYAITEE

VGAEAAMEIVAYSMNLAAARKECPAKDIVSQLVAAEGEGNLSSDEFGFFV

ILLAVAGNETTRNAISHGMHAFLTHPEQWELYKRARPKTTAEEIVRWATP

VVSFQRTATQDLELGGQRIRKGERVGLFYSSANNDPEVFDSPETFDITRD

PNPHLGFGGGGPHFCLGKSLAVMEIDLIFNAIADVLPDLRLLEDPRRLRS

AWLNGIKQLQVSVTPGP

>CYP107U(645211948)Streptomyces roseosporus NRRL 15998

MNDTPACPHSATTPESAPELFTWEFASDPYPAYAWLREHRPVHRTALPSG

VEAWLVTRYGDARQALADARLSKNPANHAESPHARGKTGIPGERKAELMT

HLLNIDPPDHTRLRRLVSKAFTPRRVAEFAPRVQELTDRLIDGFIEEGKA

DLIHDFAFPLPIYAICDLLGVPREDQDDFRDWAGMMIRHGGGPRGGVARS

VKKMRGYLAELIHRKRENPGDDLISGLIRASDHGEHLTENEAAAMAFILL

FAGFETTVNLIGNGTYALLRHPEQRAALQASLDAGESALLATGLEELLRF

DGPVEMATWRYATEPLTLGGEEIAAGDPVLVVLAAADRDPDRFTDPDTLD

LARSDNQHLGYGHGIHYCLGAPLARLEGQTALATLLRRLPDLRLAEEPAD

LRWRGGLIMRGLRTLPVEFEPGNRSEKSDTLSTL

>CYP157B(645211983)Streptomyces roseosporus NRRL 15998

MSTSSPSFDPSATAPSGCPVAPGAVRLSGASYQQTPTELYRSLRREHGAV

APVLLDGDVPAWLVLGYAELSYVTTHDELFARDSRRWNRWETIPPDWPLL

PFVGYQPSVLFTEGDEHRRRAGVITEALEGVDQFELARDCRRIAERLIAD

FAGSGRTELMSSYVHALPMRAVVQICGMPVSGSDTQQLVDDLRISLDAGE

GDDPVAAYGRVGDRLRQLVKDKRAVPGPDVTSRMVTHGAGLTDEEIVQDL

ISVIAAAQQPTANWICNTLRLLLTDERFAVNVSGGRLSVGEALNEVLWLD

TPTQNFIGRWAVRDTQLGGRHIRAGDCLVLGIAAANTDPEIWPESYVGAE

NSAHLSFSGGEHRCPYPAPLLADVMARTAVETLLEQLPDLMLAVEPAELT

WRPSIWMRGLSALPVRFSPMAQ

>CYP159A(645211984)Streptomyces roseosporus NRRL 15998

MRQDEPLLWHEATKSYIVSRYEDVERVFKDKAGEFTTENYDWQIEPVHGR

TILQLSGREHAVRRALVAPAFRGADLRDKFLPVIERNSRELIDRFRGSGS

VDLVADYATRFPVNVIADMLGLDKSDYERFHGWYTAVIAFLGNLSGDQDV

ARAGERTRVEFAEYMLPIIRERREAPGDDLLSTLCTAEVDGVRMSDEDVK

AFCSLLLAAGGETTDKAIAGIFANLLTHPEQLAAVRADRSLIARAFAETL

RYTPPVHMIMRQSAADVELSGGTVPAGSTVTCLIGSANRDEDRYRDPDVF

DIFREDLTATNAFSAAADHLAFALGRHFCVGALLARSEVETGVGQLLDAM

PDLRLADGFDPVENGVFTRGPKSLPVRFTPVSG

>CYP1035A(645213036)Streptomyces roseosporus NRRL 15998

MGDVVRAPWNGYFVTGFDTCSQVLRGRNWLAPDLAWQERQDDSKQWDAIA

TREMTTTLARLNAPEHTCQRRSLGNLFDRSTIERLAPDVERDADRLLDEL

AEKLRWGEADFVSTVSEQLPVSTVGSWLGIPPEDYPHILEITHNQVFAQE

LLPTKSQLAVSAEATLALRAYFTELVARRRAEPRHDVLTGWIHTWDAMEP

DREKADEILYRLTMFVTIASLETTATLLTSMAHLLTEPTRWAWLRQYPEH

IDAAVDEVLRYDPPIHINTRIAAEDTVLAGVPIKKDSMIHVLYGAANHDP

RRNADPGGFDILRGGSHLTFGGGVHYCLGAALAKLEARTLLARMLDRFPT

LRTATPPQYAPRMVFRRITSLGVAL

>CYP156B(645213037)Streptomyces roseosporus NRRL 15998

MDPQPGATPYSAPAGCPMHQQQTSLYGPEFAADPHRFYEAARTHGPAAPI

ELAPGVDATLIVQHEAALRVLQNPALFARDSRRWAALREGAVPMDSPVLP

MMIYRPNCLFTDGAEHLRLRKAVTESLARLNSSRLSRDVERIADYLIDQF

IERGTADLLNEYAKLLPLLLFNQLFGCPGDIGDRLTRSMSAIFDGEDVLR

ANAELTECLMELVSIKRRQPGDDITSWLIQHPAGLRDEELKDQLVMLMGA

GVEPERNLIGNALLLMLAGEQPGAPERRGSGMLVEDALDDVLWNNPPIAN

YATHYPVRDIELDGVVLKAETPVLISFAAANSDPGLTDARQTLSKGAHLA

WGAGPHVCPAKSPATLIALTAIEKILNTVPDLSLAVPASGVAWRPGPFHR

ALIALPVRFTPTAARRASAGVQPPAQTSAQLPDPYRNTPAATPVTPRHTS

EPAKKQKGWWSSFLDVFRV

>CYP107P(645213166)Streptomyces roseosporus NRRL 15998

MHVSFDPWSPAFVADPYPAYTALRAAGRAHWFEPTGQWLIPHHSDVSALL

RDRRLGRTYLHRFSHEEFGRTPPPPEHEPFTTLNGQGILDLEAPDHPRIR

RLISKAFTPRTVENLAPTVRRLAAELVDAFVAKGGGDLLAEVAEPLPVAV

IAEMLGVPEADRGLLRPWSAAICGMFELNPSEETARAAVRASVDFSAYLR

GLITERRANPGDDLISALIAAHDEGERLTEQEMISTCVLLLNAGHEATVN

TTVNGWRTLFHHPEQLAALRADPALLPTAIEELLRYDTPLQMFERWVLDD

IEIDGQVIGRGAEVALLFGSANRDPERFARPDTLDLSRTDNPHITFGAGI

HFCLGAPLARLELAASFGELLRKAPALRMTGEPEWQPGYVIRGLKELRAE

V

>CYP154C(645213479)Streptomyces roseosporus NRRL 15998

MTRIALDPFVRDLDGESAALRAAGPLAEVELPGGVHVYAVTRHAEARALL

TDSRVVKDINVWNAWRRGEIPMDWPLIGLANPGRSMLTVDGADHRRLRTL

VAQALTVKRVERLRAGIEALTNASLEKLAALPAGQPVDLKAEFAYPLPMN

VISELMGVDAADHPRLKELFEKFFSTQTPPEEVPQMMADLGTLFTKIVDD

KRANPGDDLTSALIAASENGDHLTDEEIVNTLQLIIAAGHETTISLIVNV

VEALQTHPEQRKKVLNGEIPWDGVIEETLRWNTPTSHVLIRFATEDIEVG

DKVLPKGEGLIISFGALGRDEEQYGPTAGEFDAARTPNRHIAFGHGPHVC

PGAALSRLEAGIALPALYERFPELDLAVPASELRNKPIVTQNDLYELPVE

LGCPFGGDA

>CYP157A(645213480)Streptomyces roseosporus NRRL 15998

MTTVSGCPVTHTSVPLSGPRFQSDPVQLYRDLRRDHGAIAPVVLDGDVPA

WLVLGYRELHQVTGDPVLFSRDSDLWNQWDRIPDDWPLLPMIGRKQPSIL

YTVGERHSVRAMMISNALEGVDPFSLKRYAEEFADELIDRFCTTGSVDII

AEYAKLLPALVLARIYGFSDEEAYPLVGAINDMIDGRERALAGQQHLATS

MFQLLADKHAEPGDDVASRMIADTGGFTDEEVAQDLMVMMAAGHQPTADW

MGNSLRLMLTDDRFAASLSGGRHSVAEAMNEVLWEDTPTQNVAGRWAARD

THLGGRHIRAGDLLLLDLAAANGDPQVRTDGSALTGGNNAFLSFGHGEHR

CPFPAQETAEVIARTGIEVLLDRLPDVDLAVPAEQLTRRPSPWLRGLTDL

PVLFTPTPALGGV

>CYP107E35(645215025)Streptomyces roseosporus NRRL 15998

MSTEKRIRDYPFDMEDISVSAQYARLREDEPMSRVRLPFGEPSWLATRYD

DVKLVMTDPRFSRAIAQGLDQPRLRSQLVGDGIMGMDPPDHTRLRKLVGK

AFTARRMEKMRAEVRGLASRIVDDMVAGGQSADIVEDFARLLPVTVICDL

LGVPFEDRHVFRRWTEGVTNDETAKADVLFEIGGELDDYMAGLVAQRRKE

PTDDLLGALVYARDNGDKLDDTELIALAGAGLLTGGVETVASALPSFVYT

LLTRPELLKRLREEPELLPTAVEELLRYVPINTSAMFARYALEDVWFRDT

LVRTGDPVLPALHAANRDPEVFDDPETIDLARAHNPHVTFGHGPHHCIGA

QLARMELQESLRELLTRLPDLRLADPPESIQWKFGVIVRGPAELRVTW

>CYP113AF2(645215086)Streptomyces roseosporus NRRL 15998

MYLGGRRGTEAVGESREPGVWEVFRYDEAVQVLGDHRTFSSDMNHFIPEE

QRQLARAARGNFVGIDPPDHTQLRGLVSQAFSPRVTAALEPRIGRLAEQL

LDDIVAERGDKASCDLVGEFAGPLSAIVIAELFGIPESDHTMIAEWAKAL

LGSRPAGELSIADEAAMQNTADLVRRAGEYLVHHITERRARPQDDLTSRL

ATTEVDGKRLDDEEIVGVIGMFLIAGYLPASVLTANTVMALDEHPAALAE

VRSDPALLPGAIEEVLRWRPPLVRDQRLTTRDADLGGRTVPAGSMVCVWL

ASAHRDPFRFENPDLFDIHRNAGRHLAFGKGIHYCLGAPLARLEARIAVE

TLLRRFERIEIPRDESVEFHESIGVLGPVRLPTTLFARR

>CYP159A(645215350)Streptomyces roseosporus NRRL 15998

MPVDRPDALPAARRPHRLSGGLPGLLTPGATTDPYRLRLYRLLRTDYPLG

YDPALGAWLLSRYADVALALTDPRFTGYPHDGAPRGRAPVPLGLCRGSLV

CGPLTVEWSTAAPAVERTAYVLARRIAGRDRADLVADFCRWLPAGAAAAA

TGLAHQDLSTLPRGARHRRTGGGAGDCTGTTALREHALASFLANMLDDPD

LLAAATAGEPGGAGGAGGAGSGSLLGRAWAETLRRDPPVQIVLRRTRTEV

AVSGGTLPADAPVACLVGAAGRDPARFAAPDRFDPLRADADPLLIGPAGC

PAALLGRLEAEHGLRALLTAMPGIRWADGFRPVAGGLLTRGPRALLVRPS

>CYP107L(645215500)Streptomyces roseosporus NRRL 15998

MTTEPLVDLAALGDQFTRDPYPAYAALRAKGPVHRVRIPEGADAWLVVGY

EAGRALLADQRLSKHWSRASPTLGVSKVSAGSSMLGSDAPDHTRLRKLVA

REFTPRRMEQLAPRIQEMTDELLDAMLAAPDRSADLVEALSFPLPMAVIC

ELLGVPFLDREDFRTWSGQAVSSLDASLRASSTQAMTAYIAGLLADKREK

PGEDLLSALIHTADEDGDRLSGEELIGMAWLLLVAGHETTVNLITNGVHN

LLAHPGQLAALRADFSLIDNAVEEILRFEGPVETPTYRFTTEPIEVGGTV

IPGGGELVLVAMSDANRDPDRYPGGDRFDITRDARGHIAFGHGIHYCLGA

PLARIEARTAIRSLLERCPDLRLTADPATLAWRTGMLMRGPLSLPVAW

>CYP157A(645215695)Streptomyces roseosporus NRRL 15998

MTNPSSATPASPTGTGGGCPMGSGAGAVALGGPGFDTEPQELYRSMRREH

GPVVPVELPGGVPAWLVIGYRELHQVTSDGELYPRDVSLWNQWGNIPADW

PLLPMVGTPMPSIYFTAGAEHRRHADMVVPALEGADPFEIRQHCEQLADR

LIDAVCSRGTADLVAEYAEPLPVLVLARLVGFPDDEGADIARVLKDLADG

GPDAQKAHLRFGEHMQRLVADKRARPGDDVTSRMLAHPGPFTDEEYALDL

MAITAAGHLTTADWISNSTRLMLTEDQFADALSGGRHSVAEAMNEVLWED

GPTQILAGRWAARDTRLGGRNIARGDMLLLGLGAANADPHIRQQVTASAV

RSGQGGNSAHLAFSHGEYRCPFPAQEIAEIIARTGIEVLLDRLPDLELAV

PATDLVRRPSAFLRGTTALPVRFTPVRTTGDAL

>CYP154C(645215696)Streptomyces roseosporus NRRL 15998

MNCPHAEAAQAGRNTGVITIDPMVQDLDGETVRLRDAGVLARIDLLGVPA

WTVTRHAEARQLLVDPRLVKDIDAWALWQSGVVTRAWPLIGMIDAGRSMF

TVDGSEHRRLRTKTSQALTPRRLEAIRPDIEKFTEELLDALDSAQGEDGV

VDLKTVFAQPLPMKVVGMLMGVDESQHAMLTRRYKAFFSMLTPQEERLAL

LAELDVFYTDLVREKTARPTDDLTSALILAEEGGEPLTEEEVVGNLKAMV

AAGHETTIGLVLNAVRALLAHPDQLRKVLDGEIGWDAVIEETLRWDTPTT

HLLMRFATEDIAVGDEVIRKGEGVVISYRAIGRDFEQHGPDADAFDITRP

TRNRHMTFGHGPHICPGAALSRVEAGIALPALFARYPGLRLAVPDEEITK

LPVMTQNDMAAFPVLLG

>CYP1047A(645215711)Streptomyces roseosporus NRRL 15998

MSTQTGPALDTQPRGHAFTPGPKGLPLVGNLPQFGKNPLAFFELLREHGD

MVRWRFGRNRCVFLADPDCIGELLTETEHTFDQPKLGIAFRTVLGNGMIV

ARGRDWRRKRSLVQPSVRPKQVTSYAATMAASAVELADRWSDGQRVDVKR

EMSALTQQIAVRTIFGVDTPADSEAMGRAMDVAQMEIGKEFAGIGALLPD

WVPTPGRARIKKAAAVIDAEVGRVVARHRDGEEERPDLLSRLLTAVDESG

THLTDDEIRDETVTLYIGGHETTSTTLVWAWYLLARNPRVRDALAEELDR

VLGDREPGFEDYAQLTYTQAVVKETLRLYPAVWLITGVAKEGATIGGLPI

EEGTRVWSSQWATQRDARWFPEPEEFRPERWDAEHGDEIAEYAWFPFGGG

PRVCIGTRFAMVEAVLLLAVLARRFTLDVDPGEITPVTGLTLQPDRDVTA

TVRAR

>CYP157C(645215733)Streptomyces roseosporus NRRL 15998

MTTPFHHEPGAVPPPQCPAHNLDIGPGGLRRLYGPEAENNPAGLYDKLRA

EHGTVAPVLLHGDVPAWLVLGHSENLHLTRTPSQFSRDSRRWRALQDGSV

APDHPLAPIFTWQPVCVFADGAKHERQRGAVTDSMERIDTRGVRRHINRF

SNRLVNDFCEKGTADLVSQFAEHLPMMVMCAIFGMPEEYDERLVQAARDM

TRGTETAVASNAHIVAVLTRLVERRRAEPAHDFASWLVEHPATMTDIEVV

EHLRLILIAAYESTANLIANVLRMVLTDPRFRARLSGGHMTVPEAVEQTL

WDEPPFTAVFGRWAVGDTELGGQQIKAGDALLVGIAPANTDPTVRPDLNA

DMGGNRAHLAFSGGPHECPGQDIGRAIADVGVDALLMRLPDLELGVGESE

LRWVGNIMSRHLVELPVVFAPSPQQKLDADPLSVMARTPRPADAWEISSP

ARTVPEPRHEAAAGAQPAHAPGATPQPAPVAAPPADRPVPAAAPGPAPVA

TIPRQRRPAAPARLWQAVTRWWNGY

>CYP107BX(645215930)Streptomyces roseosporus NRRL 15998

MTTIDPTVPAPSPEPMHRLRFEEPGPPSPTELPGGTPAWRVSRYADVRQV

LSDPRFGRAQLYAPDAPALSDVPDLVNDPDLMFNQDGPDHLRLRRTLRRA

FTPRAVARWRPWIAAIVDELLDRLEARPQPADAVAEFAVPLPVAVISRLM

GLDESAWARLRHWSEHAFSDGTHESGQVAAAREEFSTFGADLLMERRRAP

GEDLVSGLVAAADEEQGIPEAQLVTLVCGLVVGGHDSTMTMLGNALLYLL

GERRDTWPRLGADEEAAGRLADRLVHLVPLGDDRGSTRHAATDIEVGGAT

IPAGAIVLADCGTANRDPEVFPAATLHDLFAPLEAPTLSFGAGPHYCLGA

WLARTELQLALHRLAARFPDLRLAAPADPIVWRTGTTSRSPRRLRVSW

>CYP124G(645216169)Streptomyces roseosporus NRRL 15998

MAWRTVEVVGMTVPYQPAGDRVLRAADVDLADPAFWRLPRPERLRAFALL

RELDAPVLFTPRPGTARTAGKPFYALVRHADVRTASRTPGVFASAPGVTT

PEPAGWAKALFGNSMVNMDGSEHAALRRIISRRFTPRLLAGVEENVERLA

GRLVDELIAERPGDFVPSAASRMPLEVICDLMGIPRAYRARIAEQIDHAS

EHVGVERRGRARIRIPGRGLASLARMQWVMGRLAAERRRRPGDDLVSALV

CADIDGEALSGRQLGAFFSLLLVAGVETTRNAIAHGLFLLDRHPEQAELL

RSDFDRYIGGAVDEIVRHSTPIIQFRRTVTAEFALGGRTFLPGEKVALIY

ASANRDEAVFTHPDRFDITRSPNPHLGYGGGGPHHCLGAHLARLEMTALF

RELIDRRPVMRNLGDPELVDSNFDNRVGSLSFTFGPTFT

>CYP105B(2515816583)Streptomyces sp. LaPpAH-108

VNAMSASKDTLPDFPPPRSAACPFAPSPEMQALAAYGPVSQVRSWGGSTP

WVVTGHAEQRQLLGDPRLSVDFAAPNFPSPIDPRHGHGAATDLSFVGMDD

PEHLRLRRMISGTFTIKRVEAMRPAVQRITDDFIDRMLAGLKPVDLVQAL

ALPLPSLVISDMLGVPYEDHEFFQANSKVIVSATADRDDRTAAHRALADY

LDDLVARKIAAPGPDLISKLGSHVQAGELTAREAATMGVLLLLGGHETTA

NMISLGTLALLQHPEQLKTIRDTDDPQVVVDAVEELLRFLSIVHLGRRRT

ALEDISIAGRTIPAGDGVILLGELANRDPLVFDDPHTLDITRNARQHQAF

GGGTHHCVGQPLARLELQVLYPTLLRRIPTLRAAVPLEDVKFKYDAVIYG

LHELPVTW

>CYP170A(2515816834)Streptomyces sp. LaPpAH-108

MTVESVNPETREAAELREPPFAGGAVPVLGHGLKLARDPLAFMAQLRDHG

EVVRLKLGPKTVYAVTAPALTGALALSPDYKIDGPVWESLEGLLGKQGVA

TANGPVHRRQRRTIQPAFRMAAIPAYGPIMEEEAHALTERWRPGEPVDAT

SESFRVAVRIAARCLLRGQYMDERAERLSTDLATVFRGMYRRMVLPLGPL

YRLPFPANREFNRALADLHLLVDEIVTERRASGQKPDDLLTALLDAKDDN

GDPISEQEIHDQVVAILTPGSETVASTIMWLLQILADHPEHAEKVRAEVE

SVTGGAPVGFEHVRRLTHTNNVVVEALRLRPAVWILTRRALTDTTLGDYR

IPEGADIIYSPYAIQRDHRSYARHLDFDPDRWLPERAAEVPKYAMSPFSV

GNRKCPSDHFSMAQLSLIVAAISAKYRFRQVEDSDATTRVGITLRPHRLL

VEPVEW

>CYP107AH(2515818365)Streptomyces sp. LaPpAH-108

MTQMQEQENADETARAIDAASRGCPMYRADPHPLYRHLRENAPVSRLTPP

HGVESYLITRFEDAKAALADPRISKDMYAAIDAYRSIFGDSSIALDDNML

FSDPPKHSRLRRIVSKAFVPRRIEALRPRVQEITDALLEKCAAQDSVDLL

SAFAFPLPLQVICELIGVPEEEREEVQQWCSVVARTGFSKKDKDKLHEAE

TTLREYFEVLIARKRNAPEDDLLSVLIQTKDEQESLTDGELLSTLWVLLF

AGHKTTAYLIGNAVYTLLMHPDQLKQVIESPDLLPNAIEEVIRYEGSVEN

ATFRHALEDITVAGTVIPKGALVQVAVTSANRDESVFENPDVFDITRPGV

QSNHLGFGTGPHYCLGAPLARMEMQIALTSLFARFPNLSLETPDDEAQWL

KVPFPAFRGLTELRVALGSSEGAGERVARVADRV

>CYP154U(2515818931)Streptomyces sp. LaPpAH-108

LAHTLIAPHSENDSRCADALAFWRVTVTISGPVARAVIDPFGADIPAEIA

ELHSLGPVVPVELPGGIPAWAPTGYDVLKDLILDPRVSKDPRRHWRLWPE

IPEHPSWGWVLGWVGVVNMFTTYGSEHARLRKLVAPSFTHKRTEAMRPRV

ESITGELLDSLAEAGADGRVVDVKRMFAHELPMRMICELFGVPPELRADT

AGLIAAIMDTSDPGPEHTAFVQERIGTVLGGLIAYKSGHPGDDMTTELIR

VRDEDGDRLSDEELLYTLLLVIGAGFETTVNLIGNAVVALLDDPAQLAAI

RSGEISWDAVIDETLRVQPSIAALPLRYAVEDLTVGDITIPAGDAIITTY

AAAGLDPAHYGTDAHTFDAARGADDHLAFGLGVHRCIGAPLARLEARTAL

PALFTHFPDLTSAFRKAELHQVPSFIALGWQEIPVRLRG

>CYP158A(2515819048)Streptomyces sp. LaPpAH-108

MSEKTLTETQPPVRQWPAVDLAGTDFDPVLTELMREGPVTRIQLPNGEGW

AWLVTRHDDVRMVTNDPRFGREAVMDKPVTRLAPHFIPDRGAVGFLDPPD

HTRLRRSVAAAFTAKGVERVREKSRHLLEELVDELLQDGPPADLTRSVLS

PFPIGVICELMGVPAADRHSMHEWTQLILSSAHGKEVSEKAKREMSAYFS

DLIALREGSTGEDVTSLLGAAVGRAEVTLEEAVGLAVLLQIGGEAVTNNS

GQMFYLLLTRPELADRLRADPAIRPRAIDELLRYIPHRNAVGLSRIALED

VDIRGVRIRAGDAIYVSYLAANRDPEVFPDPETIDFERSPNPHVAFGFGP

HYCPGGMLARLESELLVDTLLDQVPGLRLAVPPDQVPFRKGALIRGPEAL

PVTW

>CYP147F(2515819138)Streptomyces sp. LaPpAH-108

MTRASLVRQITDFANRANPYPLYTELRRTPVLHEEEDGPYVISSYWDIES

LLHDPRISSDAANLAAAGADELNLAETTGLPPSFIRLDPPEHDRLRRMTN

SAFGPPDKPRRIDDMRPELHAIVSELIDGFGDAREVDLVDQFAYPFPVTV

ICRLLGVPREDEPRFRAWVDPLVASLDPDTRQGADPEFVKSAQEARMQLG

MYLAGLVEERTKEPRDDMLSDLATHRGPDGTMTMMEVLSTAVLLLIAGHE

TTVNLITNGMLTLLRHPEYLDRLREDPDLAPNIVEELLRYEPPVQLVPQR

TCITDIEVRGVRIPAGSRIWLMLAAGNRDPERFKDPDRFDPDRGDIQHLG

FGSGIHSCFGAPLARLEAQIALAELARRLEGPRLVEDPPPYRPNAVLRGP

RHLSVTFEGVRP

>CYP154D(2515819174)Streptomyces sp. LaPpAH-108

MDTAPAGPPHRLDPAGGCPHADNARLLALGPVAAVELPGGVEGMAVLGHE

ALKEFLQHPEVAKDARHFAALREGRIEPGWPLLTFATVRGMTTADGEDHR

RLRSLVARAFTPRRVEALRPRVVALTDALLDDLAVAAEAGAGVADLRRHF

ALPLPLGVIGELLGVDEKFRDRLHRLSNAVTGTDIGPEAVLAANRELVGV

LGEIAADKAARPGDDLTSALIAARDEGGDRLGHEELIGTLVLLIVAGHET

TLNLITNAVRALCGHPGQLALVRAGGASWADVVEETLRWDAPVSYFPFRY

PVRDLQVGGTVIPAGTPVLAGYSAAGRDTAAHGPDADRFDLTRASRPGAV

RHLSLGHGAHFCLGAPLARLEATVALERLFTRFPDLELAAAEADLRRHPS

FVGNTVHALPVRPAPSARPRTD

>CYP159A1(2515819287)Streptomyces sp. LaPpAH-108

MTTAAQIPDILSPEFAADPYPAYRLMRETAPLIWHEATQSYIISRYEDVE

RVFKDKDGEFTTDNYDWQIEPVHGKTILQLSGREHAVRRALVAPAFRGRD

LQEKFLPVIERNSRELIDAFRHSGSADLVADYATRFPVNVIADMLGLDKA

DHARFHRWYTTVIAFLGNLSGDQGVAAAGERTRVEFAEYMLPIIAERREN

LGDDLLSVLCAAEVDGVRMSDEDIKAFCSLLLAAGGETTDKAIASIFANL

LSHPDQLAAVRADHSLIPRAFAETLRFTPPVHMIMRQSATEVKLSGGTIP

AGVTVTCLIGSANRDENRYREPGRFDIFREDLTATNAFSAAADHLAFALG

RHFCVGALLAKAEVEIGVAQLLDAMPDLRFADGFDPVEQGVFTRGPQALP

VRFTPTA

>CYP157B(2515819288)Streptomyces sp. LaPpAH-108

VTDPGSFSAQQAPPPGCPAHTDAVRLAGLEYQQTPSQMYRALRREHGAVA

PVLLDGDIPAWLVLGYHEVAQVTSHDELFARDSRRWNQWDDIPPDWPLMP

FVGYQPSVLFTEGEEHRRRAGVITEALSGVDQFELARDCELIADELIARF

AGSGRAELMASYVHALPMRAVVQMCGMPLSGADTQQLVDDLRISLDAAEG

DDPVAAYGRVGERLARLVQDKRADPGKDVTSRMVLHPAGLSDEEIVQDLI

SVIAAAQQPTANWICNALRLLLTDERFELSVSGGRVSVGEALNEVLWLDT

PTQNFIGRWAVRDTQLGGRQIRAGDCLVLGLAAANTDPSLWPEGHVGAEN

AAHLSFSNGEHRCPYPAPLLADVMARTAVETLLERLPDLVLAVDPHALTW

RSSIWMRGLTTLPVRFTPVDG

>CYP107L(2515819370)Streptomyces sp. LaPpAH-108

MCPHADFGLRHVCRPLLASGRADERGQTVTTTDVIDLVAMGEDFVRDPYP

VYAALRERGPVHRVRIPEGAEAWLVVGYEAGRAALADPRLSKRWENASPA

FPISSPAAGPHMLNQDPPDHGRLRKLVVREFTPRRIEQLAPRVQQITDEL

LDAMIAVPEGRADLVEALSFPLPISVICELLGVPMLDRAAFRHWTNTILS

SAEPEVRQAATRETAAYLAELMERKRQAPGDDLMSDLVRATDEDGDRLSH

EELLGTAWLLLVAGHETTVNLISNGVLALLTHPGQLDLLRKDMTLMPNAV

EEMLRYDGPVETPTFRFTTEPIEIGGTLVPGDGQLVLVALADANHDPARF

AAPDDFDIRRPTGGHVAFGHGIHYCLGAPLARLEARLALTSLLTRFPTLT

LDAHPAALTWREGMLIRGPHSLPVRWDT

>CYP156A(2515819720)Streptomyces sp. LaPpAH-108

MTSADPQAPARIPLYAPEFAADPHAAYERMRTTYGTLVPVELSPGIPATL

VIGYDAARRILNDPLRFPADPRVWQKAVPRTCPVLPMMEWRPNALRSGGT

EHARYRGANTASLDAVDQHQLRALVETIAEEAIAGFGDTGRADLLTQYAW

PISFRVLSALLGCPDDIGARIADGMAKIFEAKDADHGNALLAQAIGELVG

LRRERPGDDVTSRLILHEAALDDEEMAHQLVTLYGAGIEPLTNLIANTQL

KLLTDDDFSAELHAGQSTLRDALDTVLYSDPPMANYCISYPPYPTDIDGC

LLPAHQPVVISLSACNNDPELTLGMPASSVSGNRAHLAWSIGPHTCPARS

HAYLIAETALSHLLDAIPEMDLAVPASELRWRPGPFHRALEALPVLFPVH

R

>CYP154A(2515819721)Streptomyces sp. LaPpAH-108

VPEQSISATEQSVLVLDPTGADHHAELKALRARGAATQVDILGVTAWSVT

DPDLLKQLLTSPDVSKDARAHWPGFQDALTSWPLILWVAVSNMFTAYGGD

HRRLRRMVAPAFSARRIAVLKPVVEDTVAGLLDALAARPADEVSDLREQL

AYPLPITVIGRLMGVPVDQTAVFRTTVDGVFDTTLSTEEAMKNTAGLYAA

LDELIAAKRAEPGDDMTSLLIATRDEEGDDTGMTDEELRDTLLLVISAGY

ETTVNAIDQAVTALLADREQLEHLRAGRATWEDVVEETLRFEPSVKHLPL

RYAVRDITLPDGRVIAEGDAILASYAAANRHPDWHGATADQFDVTRLSKE

HLAFGHGVHFCIGAPLARMEIAAALRQLFERFPDMEPAVPADELKPVHSL

ISNGHQSLPVRLRPAG

>CYP107P(2515820334)Streptomyces sp. LaPpAH-108

MSATFDPASPGFLADPYPAYDELRARGRVIRYEPTDQWLVPHHADVSALL

RDRRLGRTYQHRYAHEEFGRTPPPPEHEPFHTLNDHGMLDLEPPDHTRIR

RLVAKAFTPRTVERLRGYVEGLAGELVGRLVAEGGGDLVAEVAEPLPVAV

IAEMLGIPEGERGPLRPWSADICGMYELNPAPEVAARAVRASEEFSGYLR

GLIAARRADPRDDLISALIAAHDEGDRLTEQELVSTAVLLLNAGHEATVN

ATANGWLALFRHPAQLAALRADHTLIPSAVEELLRYDTPLQLFERWVLDD

IEIDGTRIPRGAELALLFGSANHDPAVFDTPADLDLTRRDNPHISFSAGI

HYCIGAPLARLELTTSMRALLEQAPTLTLASEPHRKPGFVLRGLNELEVS

VV

>CYP107U(2515820612)Streptomyces sp. LaPpAH-108

VTDQPSCDRPGEPAPDLFTWEFATDPYPAYAWLREHAPVHRTRLPSGVEA

WLVTRYTDARQALADARLSKNPAHHDEPAHAKGKTGIPGERKADLMTHLL

NIDPPDHTRLRRLVSKAFTPRRVAEFAPRVQELTDDLIGSFAAKGSADLI

HDFAFPLPIYAICDLLGVPREDQDDFRDWAGMMIRHGGGPRGGVARSVKK

MRGYLAELIHRKRAALPAEPAPGEDLISGLIRASDHGEHLTENEAAAMAF

ILLFAGFETTVNLIGNGTLALLTHPEERVRLQRSLAAGETGLLQTGVEEL

LRYDGPVELATWRFATEPLTLGGQEVAAGDPVLVVLAAADRDPARFPDPD

VLDLGRADNQHLGYGHGIHYCLGAPLARLEGQAALATLFTRLPDLRLAVE

PAELRWRGGLIMRGLRTLPVEFTPIGPQHN

>CYP102B(2515821105)Streptomyces sp. LaPpAH-108

MAETTRSALPKGFRSAEHGWPELDRIPHPARRLPLVGDVVGVDRRRPLQD

SMRFGRELGPIFRRRAFGKEFVFVWGARLAADLADESRFAKHVGLGVANL

RPVVGDGLFTAYNHEPNWQLAHDVLAPGFSREAMETYHGMMLSVAGRLTA

HWDGELAAGRTVDVPGDMTKLTLETIARTGFGHDFGSFERDRPHPFVRAM

VGTLTYAQRLNSVPGPLAPLLLRTAARRNAADIARLNRTVDALVAARLAS

GGGTGDLLDRMLETSHPVTGERLSAENVRKQVITFLVAGHETTSGALSFA

LYYLARHPEVAARARAEVDRVWGDTPEPGYDQVAKLRYVRRVLDESLRLW

PTAPAYAREATRDTELAGEHPMRRGAWALVLLPLLHRDPEVWGEDAERFD

PDRFDAKAVRGRAPHTFKPFGTGARACIGRQFALHEATLVLGLLLRRYEL

RPDPGYRLEVTERLTLMPEGLRLGLERRGAAGVGAAAGREREVGAGVVGL

GAAGSGAAAPGAGAGSAGAAVAGSAPPASGSAVPGPGSAGRCPVHRADQ

>CYP102G(2515821112)Streptomyces sp. LaPpAH-108

MPPTAPLPEPADQPGVPVVDISATGPGRTPIQQVMGLMRTHGPALVRRLH

GRDALFVADADLVAELADEDRFAKHIGPALENVRAFAADGLFTAYNDEPN

WAKAHDILMPAFALGSMRAYHPVMLKVARRLIASWDRAAGAGRPVNVPDD

MTRMTLDTIGLAGFDYDFGSFDRDEPHPFVEAMVRCLEWSMTRLARVPGG

DYTAADAAFKADADYLAGVVDEVIAARTGTDQSDAQDLLGLMLTAQHPAD

GTTLDTANIRNQVITFLIAGHETTSGAMSFALYYLAKHPTALRLVQREAD

ALWGDAADPEPTYDEVGRLTYTRQVLNEALRLWPTAAAFSREAREDTLLG

GRIPLRAGQAVSVLVPMLHRQDVWGDNPELFDPERFTAEAEAARPVHAFK

PFGTGERACIGRQFALHEATMLLAMLVHRYRLHDHADYVLDIKETLTLKP

EGFTLALTPRTPADRAHAPLPGAAPATAAQDSATDTLPARVRPGTAALFL

HGSNYGTCREFAAQLADEAAALGCATETAALDAYADGLPTDRPVVITAAS

YNGRPTDDATAFHAWLDGDHDLTGVTYAVLGVGDRNWAATYQHVPTKIDT

RLAELGATRLTERAAADASGDLTGTVRDFTAGLRTALLQEYGDPDAIGSD

ATDEPETAYEVRALTGGPLDAAVERHGLVPMTVTEAHDLTAPGHPRVKRF

LRVALPDGVTYRTADHLTVLPANDPDLVERAAGALGVDPGTVLDIRATRP

RRDGLAVDRPLTVRELLTHHVELQEKPTRRQLDLLAEANPCPPERAALAA

LPGDDPRTLLEIVEDHPALRGALGWPLLLDLLTPLRPRHYSVSSSPAADP

HHADLMVSVLDAPARSGKGRYRGTGSNHLAALKPGDTVYARVQPCREVFR

IDGSAPVVMIGAGTGLAPFRGAVADRAAARAQGAELPTALLYFGCDAPDA

DYLHADELRAAEAAGAVSLRPAFSAAPENGALFVQHRIAAEADEVWDLLN

AGARVYVCGDGSRMAPGVREAFRTLYREHTPGADEADAVRWLDSLAADGR

YVEDVYAAG

>CYP105D(2515821124)Streptomyces sp. LaPpAH-108

MTELTDITEPDTPAAPVAFPQDRSCPYHPPTGYGPLREARPLSRVTLFDG

REVWAVTGHTAARALLADPRLSSDRTRPGFPVPSARFAGVRDRRVALLGL

DDPEHKAQRRMMIPSFTVKRAAALRPSIQRIVDDLLDAMIEQGPPAELVS

AFALPVPSMVICGLLGVPYDDHEFFEEQSRRLLRSPTAEETQAARVALEK

YLGDLVDRKAAAAEPGDGVLDDLVHQQLREGALDRTETVSLALILLVAGH

ETTANMISLGTFTLLRHPERLAELRTDPSLLPGAVEELMRMLSIADGLVR

VALEDIEVEGETIRAGEGVLFSTSVINRDAAVYEDPDSLDWNRSARHHVA

FGFGIHQCLGQNLARAELEIALGSLLTRLPGLRLAVPAEEIPFKPGDTIQ

GMLELPVNW

>CYP154C(2515821207)Streptomyces sp. LaPpAH-108

MTTGTATSPIALDPFVTDLDGESAALRAAGPLAAVELPGGVPVWAVTRHA

EAKALLTDPRLVKDINVWSAWRRGEIPADWPLIGLANPGRSMLTVDGADH

RRMRTLVAQALTPRRVELMRERIEKLTQGLLDALPADGGTVDLKAAFAYP

LPMYVVADLMGIDTAELPRLKVLFEKFFSTQTPPAEVLATLTELARIMAA

TVAAKRAEPGDDLTSALIRASADGDHLTDEEIVSTLQLMVAAGHETTISL

IVNAVVNLSTHPDQRALVLSGEADWSAVVEETLRWSTPTSHVLIRFATED

VPVGDKVIPAGDALIVSYGAIGRDEEAHGPSAGEFDITRETKNRHISFGH

GPHVCPGAALSRLEAGVALPALYARFPHLDLAVPAADLRNKPVVTQNDLF

ELPVRLNG

>CYP157A(2515821208)Streptomyces sp. LaPpAH-108

MADAVPLGGPRFQTEPARLYREMRRDHGSVVPVLLDGDLPAWLVLGYREL

HQVTGDPVLFSRDSELWNQWENVPDDWPLLPMIGHRQPSILYTVGERHRQ

RAAMVSNALESVDPFELRGQAERFADELIDALCSSGEADLVGQYAGLLPV

RVLAHLYGFPDEDGPGLVTALNDMIDGRERALAGQAHLASSMARLVAERA

KEPADDVVSHMLADGSGFTEEELVQDLMVMMAAGHQPTADWIGNSLRLML

TDERFAASLFGGRNSVAEAMNEVLWEDTPTQNVAGRWAARDTHLGGRRIR

AGDLLLLGLQGANSDPQVRTDASALTGGNNAHFSFGHGEHRCPFPAQEIA

EVIARTGIEVVLDRLPDIDLAVPAESLTRRPSPWLRGLTELPVRFTPVPA

L

>CYP154B(2515821242)Streptomyces sp. LaPpAH-108

METTACPYVLDITGRDHQSEAAHLRGQGPAVPVELPGGVVAWAVVQQTYV

ERLLADPRVSRSARLHWPAFIEGKITEDWPLYPWVANENMLFAYGEHHSR

LRRLVAGAFTLRRSEALRPRVEELSAELLDSLAAVAPGTPVDLRTDYAEV

LPLRVICELFGVPRGAETDALSDALSTVFSSTTPAAEMEAARLQAFGLLA

RLVAVKREQPGDDLTSSLIAARDNGDRLTEEELLGSLFMFIAAGQDTTAT

LITNAAGALLTHPEQLEHVREGRATWADVVSETMRVHTPGAYSPLRFAVE

DIDLDGVTLRKGDCILVDFASGGHETDRHGPDASRFDVLRTKRDVLGFGH

GPHRCLGAPLGEIEAASALSKLFGLFPDVRLACAPEELRPLPTFMLNGYR

SLPVLLRPTDA

>CYP158A(2515821245)Streptomyces sp. LaPpAH-108

MPFDSTSAPDTAPASPPPPPPVRSWTVEDLPALDPDPFFDELLRDEPITR

ISLPYGDGHAWLVTRYEDVRAVTSDPRFSRAELVGRSVTSMAPQAVASQT

AGLQYIDPPRHTVLRRVVARAFTARSMARLRPVAERIAHRALDGMEQAGP

PGDLMEHLYTPFPIAVVCAFLGADEGDWRAWAGHSEALLSKAADGEDRNR

QARATTRGRVVDLLDRRRAEPRDDLAGVLAAAAATGEITDDEAVSLAMAV

YVSGGHAVRNNSGSMMYALLTHPEQFARLRREPELLPRAVEELFRFVPHR

NGVGIPRIATEDVEIGGHLIRKGEAVYNAYVAANRDPEAFPDPDALDFDR

TGPGHLAFGHGPHFCLAALMARMEAEVLIGSVMERFPALRLAVPPGEVEF

QRDGLIRGPRTLPVAW

>CYP157C(2515821669)Streptomyces sp. LaPpAH-108

VTPESHPPTGTHDPTAGPPPGCPAHGLGPGALHRLHGPGAGDLDELYECL

RERYGPVAPALLHEDVPMWVVLGHAENLQMVRSPSQYTRDSRIWTPLLDG

QVKPDHPLMPHIAWQPICSHAEGDEHQRLRDAVTSAMATIDHREMRRTIG

RYTQGLVNAFCERGHADLVPQFAEHLPMATLCAILGMPEEYNDRMVQAAR

DALKGTETAIQSHTYVMEALSRLALRRRARPENDFTSHLVTHEAGLSDDE

VREHLRLVLFAAYEATANLLANALRMVLTEPGFRARLNGGQMTVPEAIEQ

SLWDEPPFSTVLGYYAKQDTELGGQRIRRGDGLLFAPAPGNVDPRVRSDP

RAQMKGNRSHLAFGGGPHECPGQDIGRAIADVGVDALLTRLPDIRLDCPE

DELRWRSSIASRHLVSLPVRFEPKLQQDVSLPPSQSPIPRPREQWQVTAP

ARAAAPVPEPRQEPVRPEAEPVRVREWESARMPEPVGAAVSGGESGRESV

WRRLMRWWVGE

>CYP1341F2(2515822289)Streptomyces sp. LaPpAH-108

VAIRNQQLDRIGSNVRDLADSIISRRSRSSVSFDFTAEVARPAALLGVCE

LLGIEKPPVHSFAALADAMTRGMDAGLLPEVLAPALAARTELNRRIADWF

DACDDQGLLSEVLTGAQNAGIPREAVWNSARVLFLAGFSTAVGAAANAVL

ALATHPDAFEELRGSDALRSGVDEFMRFDGPVQGTTRACVHETQIGDVTI

TRGDLVTALLGSANRDPEMFKNPDELVLTREPNRHLALGWGIHGCSGAIL

ARIMIRALLESLIQNRGRLSLAEPPERLPRATLRYPDRLPLSFR

>CYP1282B1(2515822290)Streptomyces sp. LaPpAH-108

VKELLVHYDPLAPETIHDPYPTYKAMRELGAPCWNEIMKCWVVTSYDGCK

RVLSDYENFAADWRRGGESMPENALSVHSLDPPDSTVIYRELAATLRQVI

APDLSHRLRQAISDRLRTLAGPPVDLVSDFTEPLACWFLSEVLEVPSLDT

PEIRSMGGAIHRAMDAGLVPEAFQPGRDAHRKLAALIEAWVASMTPQQPL

RTLVDRAAVEGLEGAMTLNSVRTLIANAFTSLPASLGTIVHGFARDPGLL

AGTVNEKRLDMAVHEFLRHESPSQGTTRLCVADTVLSGAKIRRGEDVLVL

VSAGNRDPAKFDDPDQLRPERWPNQHIAFGHGPHSCSAAMLSRVLLRELV

AVLADGSISIRLTGPVTYKPAATIRMRATLPAVVTPAS

>CYP105BT(2546762207)Streptomyces aurantiacus JA 4570

MTMANPTYPMTRQCPMSPPAQYADLRAHGPARVDLPDGGWAWLLTGYDDV

RQAMNDPRFSSDDTKMGRARTELPPNENLNSFWRMDEPEHGRQRHMMMSE

FTAKRIKEWRPRIQKLVDELLDRLQSLPRPLDLYAEFALALPTQVIAQLL

GVPQQDYRTFAEQSRTILSLDKPEESWAAYYKMNDYLNRLIDEREREPQD

DLISRLITERVLTGELPREDLLPMVRFILVSGYETTTSQIALSALTLMTN

PDVRDRLIEDPDRVTAFVEESLRFWSVSQDNVLRVVDQDMTFSGAAMTTG

DLVILAVPGANHDASAFPDPERFDLERGENRHVAFGFGTHLCAGASLARR

EVEIAVTSLLARLPGIRLACDIEDLTFRHKSLVYGLERLPVTW

>CYP247A(2546762226)Streptomyces aurantiacus JA 4570

MRLTPGPARDVDLECLDLFDLDLYSYGDPHPVWDVMRDKAPLHHQVLPDG

REFWSVTRYEDVCRVLGDHREFTSERGTTPTHVGIDDPAAGVLLTSTDPP

RHTEVRRPIGTKLTARAVKSWEDSIRRTIQRFLEPALEGETFDLAEKALL

LPAMVTGPLLGIPEKDWEELVQLTAMVTAPSDPHFKTGSEAATLAIAHHE

LVTYVKEWVRTRRASGAEDDSLLHHLMSVRPGGAPLSDEEIALDGYSILL

GANVTTPHTVSGTVQALIERPEQFEKAQADPSLVPNLVEEGLRWTSAACN

FMRYAVDDVQIGGGTIPARGAVVAWIGSANRDASQFPDPHEFDITRAGAK

RHVAFGFGPHFCIGGPLARMTLRIFFEELLQRFGSIELDGEPQHLRSYFI

AGMTHLPIVAQKRKTP

>CYP179A(2546762383)Streptomyces aurantiacus JA 4570

MDRQQHARLDELLRDPYPLYARARRAEGLTYVPELRAWLVARDADVREVL

RRPDVFSSANAVRGDVVPSQAALAVLGEGIAVRRPVVITADGPAHQRLRE

PIVRGLTPARVAAVLPYVAERAEALADAFAAPGSGRTVDVMEAYALRLPG

QVIGHVLGFDPADVPLMVRGGHSTEQLLFRPLPEEGQLAAAHDVIALQRL

LDGYVRDRHAAPRPDLCSEIVTSLVPDDGDPEDGEGDPEDGGAEPELTLG

QRGELIAHLQNFLLAGHLTTTALIGTTLLHLLRHPAQWKLLCEEPERIPA

AIEEAARYDTALQGFRRVTTRAVTLAGTELPPGAEVLVGFGAAGRDEGRY

ERPDVFDIMRVPVARHVAFGLGAHACPGAQLAREQLRLTLELFTRRFPGL

RLARDGPPVRMRPTLIHRAPEALYVEW

>CYP107P(2546762482)Streptomyces aurantiacus JA 4570

MDAPFDPWSPAFVADPYPAYAALRAAGRVHHYAPSDQYLIPHHADVAALL

RDRRLGRTYLHRYSHEEFGRQAPPPEHEPFHVLNDHGMLDLEAPAHTRVR

RLVAKAFTPRTVERLRPYVEGLADELVRGLVADGGGDLVARVAEPLPVAV

IAEMLGIPEADRGPLRPWSADICGMYELNPGEETAARAVHASVEFSSYLR

ELIAARRREPGDDLISALIAAHDEGTGHDEGDRLSEQEMISTCVLLLNAG

HEATVNSTANGWNALFSHPEQLAALRADPEKLLPTAVEELLRFDTPLQLF

ERWVLDDIEVGGTRIPRGSEVALLFGSANRDPAVFAAPDTLDLARADNPH

ISFSAGIHYCVGAPLARIELAASLGALLRRAPDLRLAAKPTRKPNFVIRG

LGELLVEC

>CYP2365C1(2546762687)Streptomyces aurantiacus JA 4570

MGRLEADPYPIYGRLRAEEPVAWVPAVGAWLVTRWDDVRHVLTSPESYTT

TNDASPLSTYCGSQNVLNQEGDRHAAIRKAVGDRFRQETAPAVTAHARSV

AARRLGDLADRDHADLMAEYFEPVSVETASWLLGLDAVDGVDTATLTRWS

TGLTAALYNPTKAGAAHVTGAAVSGAMDRTLTPHLARLLDQPDDSPLSAL

VHAAGPADDASWGVHQTLATVRMMTSAVREPGWLAGNTLHALLTHPEQMD

ALRADPGLLDAAVYEGIRWAAPVGAVGRLTTKPLVLGGQELPAGAPVAPG

IASANRDEAAFPAADRFDLLRARTTTPLSLGLGRHECLAAHVVPAIVEGA

LRQLLERFPELHLEQDVRPYGWKFRKLDTLPVGWRDRSAAGA

>CYP154A(2546762757)Streptomyces aurantiacus JA 4570

MLDPSGVRRHAEDRRLRAQGPAARVDILGVTAWAVTDPVVLKQLLGSPDV

SKDARAHWPEFDKVVPEWPLALWVAVENMFTAYGADHRRLRRMVAPAFSA

RRVNALTPVVEDIVTGFMDDLAAVPEGAVVDLRERLASPLPIAVIGHLMG

VPAARRPGFRALVDGVFDTTLTGEEAKANTGRLYEALAELIAEKQAEPGD

DMTSLLIAARDEEGDGGRLSATELRDTLLLMISAGYETTVNVIDQAVTAL

LTRPGQLDHVRAGRADWNDVVEETLRHEPAVAHLPLRFALTDITLPGGTT

IAAGEAILASYAAANRHPDWHGPTADTFDVTRALKDHLAFGHGAHFCLGS

ALARLEVATAVRSLFDRFPALELAVPAAELRPLASFISNGHAELPVRLRP

APS

>CYP157C(2546763188)Streptomyces aurantiacus JA 4570

MFTWQPMCSFAEGAEHQRLRGAVTGAMAGLDHRGIRRHINRYSNRLLNDI

CEIGRADLVGQFAEHLPMMVMLHVLGMPEEYSERMVQAARDMIKGTETAI

ASNAYVMEALTRHVVRRRAQPEEDFTSALIAQPAQLTDDEVAAHLRLVLI

AAYEATANLIANVLRMVLTDPRFRAQLSGGQMTVPEAVEQSLWDEPPFSA

MVGYFAKQDTQLGGQQIKAGDGLLLGIQPGNVDPIVRPDLSANMQGNRSH

LAFSGGPHECPGQDIGRAIADTGVDALLMRLPDVELAVDERELRWRSSIL

SRHLVELPVQFGKRPPQDVTARPMSHVPTPRKNWEVSSPMPQPFPQPAAP

AAQPAAPAAPAPQDPAAAPVRRKGAWRRFLEWWRGY

>CYP157K(2546763675)Streptomyces aurantiacus JA 4570

MNAPRPTGPSAAPQEATTSGCPVAHGGQDSLTRLYGFDASTDPQSVYERL

REHHGPVAPVLLEGDVHAWLVLGYRENRRVLENPRQFTRDSRIWRDWKEG

RIEESSPLIQMVGWRPDCVSQEGEAHRRLRAAVTDNLQATAGRGIRRHVT

HFANKQIDAFVSAGRAELVTDYAQSLPMLVLTRLFGLAEGEGRQLAVSCA

QVIKGGEEALAHNDRIMKILGELAERKRAEPGSDFTTGLLEHRAGLDEGE

VLSHLRLVLIAAHTTTSNLLARVLQLLLTHPSHMAGVVSGRLDISAVVEE

VMWNTPPLAVCPGRFATADLELGGQQVKAGDLLVLGLAAGNRDPEVRPDV

TTALHGNKAHLAFSGGSHECPGQGVGQAIIETAVDVLIHRLHGLRLAAAA

EDLTVTASSWEARLDRLPVEFTAQET

>CYP183J(2546763677)Streptomyces aurantiacus JA 4570

MTTVAASPIPTATGALPLLGHAIPLLRDNLAFIASLRDYGPLVRIHLQPR

QSTVVVNEPALIRAMLVDLAPGLDKGRFFEKMGQVLGGDSVVTAAGSDHV

RKRRQLQPAFRHDEIARYVDLMRHEVVCTAEAWTPGGTLNVREAMVKLSL

DMLAATVFASSLDEQAFRRLRRDLSVVMNGVGARVMLPDWVERLPLPANW

RFNSSRAAVRDIVGGAVNGLRASGHDTGDMLSTLLRAEDPETGRPMTVHQ

ICSEVLTLAVAGTETTASVLSWVLYELARNPEVDARLQKELEDVLEGRAV

TFADLPRLPYLRNVITEALRLHHTGWLVTRRTLAAVRLGDWEIPPGTELA

YCQHALHRDASLFPDPLRFDPDRWLDETWQAALPDGAFLPFGAGKHKCIG

DHFAMTELATAIATLARMWRLDLTSGREVRPEARATVRPSTLMMTPRRRE

P

>CYP105DH2(2546763986)Streptomyces aurantiacus JA 4570

MPPVYTKFREESPITQVVLPDGGKAWLVTKYDDVRAVMANPKLSSDRRAP

DFPVVVPGQNAALAKHAPFMIILDGAEHAAARRPVISEFSVRRVAAMKPR

IQEIVDGFIDDMLKMPKPVDLNQVFSLPVPSLVVSEILGMPYEGHEYFME

LAEILLRRTTDEQGRIAVSVELRKYMDKLVEEKIENPGDDLLSRQIELQR

QQGGIDRPQLASLCLLVLLAGHETTANMINLGVFSMLTKPELLAEIKADP

SKTPKAVDELLRFYTIPDFGAHRLALDDVEIGGVLIRKGEAVIASTFAAN

RDPAVFDDPEELDFGRDARHHVAFGYGPHQCLGQNLGRLELQVVFDTLFR

RLPELRLAVPEEELSFKSDALVYGLYELPVTW

>CYP113K(2546764086)Streptomyces aurantiacus JA 4570

MRETGPVRFDEAQGVWHVVDYQGVAAVLADPATYSSDMTPIAPSQEDFDA

FKQGNFVGMDPPEHRKFRTLVSQAFTPRVVHGLEPRIEAVCRRLLDTVAG

RDRFDVVGTLAHPLPIIVIAELLGIPPDDHPLFQEWAATLFGGDQLGDAP

DAADLERALEAIAPTVREMNSYVLDHIRHRRAHPADDLISKLIAAEVDGV

RLEDQEMVGFVALLLVAGHVTTTALLGNALVAFDQQPGTLTALRAEPDRL

PDAVEEVLRLLPPFPELGRRIARPVVLGGHELPVNSLVMAHLGAANRDPS

RFTAPDTLDIRRAPNNHLTFGHGIHFCFGAPLARLEARIALRMLLDDYRD

LAIPSYADVTYQNPAVIVGVRQLPVEVKRP

>CYP1658A1(2546764541)Streptomyces aurantiacus JA 4570

MSGEFPMSWKAVPLVSPSGPSPARCPAHAGQDDAERAEAGEGPAPGGHLE

ELRREPLGLLRRSAKESAHRLARLDTGPGACLVVSDPDAVDVVLTDHDAA

FFKPKMERWKQVLGENVLTSEGPAWARSRRRSMRITGARYVRRAARVVAE

VTDERLRSWAGTGAVGVDGAADSPRSPCIPGSHDIPDIHEEMRTLTLAAV

LRHLCGTDDDFDLRAFGRHLRTVMECIHALESAPAGAEPSARVERAFAHA

AGELRAAVLRLVASHAGGDDLVGLLTAPPDTASDDGRAAEPLTPDQVCDE

VLAHLIAGHESTATALAWSLLLLSRDADVTRRVRTEIGTTVGGRAPGPAD

LAGMPLLQAVFTEALRLCPPAWTIMRATGRPCELSGVRLPAGAVLLASPY

SIQRSGRWFPRPDEFLPDRWLGDAAAGAPRYAYFPFGGGRRSCPGRMLAQ

VTAGVVLTRVLQGFRIEPVGEPPTADVGIILRPSPGTRLRAVPLRRESAG

QREESP

>CYP163B(2546764837)Streptomyces aurantiacus JA 4570

MTVQPARAHDGLRFPDLADPRTFIDTDLPALWRELRAASPVHWNPPAEDR

PGFWAVSRYEDVLAVYKDNKRFTSEQGNVLSTLLQGGDSAGGKMLAVTDG

VRHRETRKLMQKSFSPRVLEHVGQLVRRRTRQLIAEVVARPEADFATDVA

DHIPINTIGDLMDIPVADRGKLVDWNNRTLSRYDEGDSQLDEWLARNEIL

LYFGELARRRRESPGDDVVSALATGLVDGEPLSEDEIIFNCYSLVLGADE

SSRMSSIGALIAFIEHPDQWRALKNGEVSIADATEEVLRWTTPAMHFGRR

AVEDVVLRDELIRAGDVVTLWNSAANFDEDAFPDAHRFDLGRKPNRHVAF

GHGPHFCIGAFLGRAHVSAVLEALRDQVAGIELRGEPRRLYSNFVYGYQG

LPVALTAER

>CYP1031A(2546764957)Streptomyces aurantiacus JA 4570

MTWDLSSNKEHEMTGGVLPQEQIPLFNPFAEGFTDNPYPQYAMLRETAPV

YPHPMGFWVVTRYADLLALLRSGASVELRNVDSGGMENLRQKDENRKTPL

IDGYSMIDRDAPDHTRLRRLVQKAFTPKSIQALAPRIGELVDGMLDRIAD

AGRADLVPELASPLPFAVIAEMLGAPPTDHERIRELSGTMVRSLEPVADP

AVMQAIEAADAEMAEITAEMIEWKRRNPADDLMTALIEAEEDGDKLSDDE

LVAQIELLYIAGHETTVNLLANGTLALLRHPDQLAALRADPALMPNAIDE

LLRYDSPLQSSRRITLEPTVLSGVEIPAGAFVIGGLASANRDEAHWGPDA

DTLRLDREGARGHLAFGSGSHHCLGAALARLEASITLERMLARFPELALD

GEVTWNGRINVRGVASLPVSVG

>CYP159A(2546765704)Streptomyces aurantiacus JA 4570

MSTAQPIPDILSPEFAADPYPVYRAMREHTPLLWHEPTKSYIVSRYEDVA

RVFKDKESQFTTENYDWQVEPVHGRTILQLSGREHAVRRALVAPAFRGAE

LQNTFLPVIRRNSEELIDAFRDRGTADLVADYATRFPVNVIADMLGLDKA

DHDRFHGWYTTVIAFLGNLAGDPEVTAAGERTRVEFAEYMFPVIARRREN

PGDDLLSTLCAAEVDGVRMSDEDIKAFCSLLLAAGGETTDKAIAGIFANL

LTHPEQLAAVRADRSLIDRAFAETLRHTPPVHMIMRQTAAEVALAGGTLP

AGATVTCLIGSANRDGERYAHPDRFDIFRDDLTTTTAFSAAADHLAFALG

RHFCVGALLARAEVETGVNQLLDAMPDLRLADGFVPSEQGVFTRGPSALP

VVFTPVPR

>CYP157B(2546765705)Streptomyces aurantiacus JA 4570

MPLSGLEYQQTPFDLYRDLRGKHGSVAPVLLDGGVPAWLVLGYSEVSYVT

AHDELFARDSRRWNQWDNIPADWPLMPFVGYQPSVLFTEGAEHRRRAGVI

TEALESVDQFELGRECAYIADQLIAAFAGRGNAELMSVYTHPLAMRAAVH

MVGMPPGAADTEALVEDLRMSLDAAEGDDPVAAYVRVGERVHHLVKERRQ

NPGADVTSRMLEHPAGLSDEEIVQDLISVIAAAQQPTANWICNTLRLLLT

DERFALNVSGGRLSVSQALNEVLWLDTPTQNFIGRWAVRDTQLGGRRIRA

GDCLVLGLAAANADPQIWPGAQVGSENSAHLSFSNGEHRCPYPAPLLADV

IARTAVETLLERLPDLVLSVDPAELTWRPSIWMRGLTALPVQFTPVAQ

>CYP107X(2546765819)Streptomyces aurantiacus JA 4570

MEGMATPAAPEPLDGLMNDPYGTYAQLRETAPVRRIAGPDGNPAWLVTRY

ADVRAALADQHLSVDKRHALPGSYRGFSLPPALDANMLNMDPPDHTRIRR

LVVKAFTKHRVESLREPIRRTADALLDAIEEKARTHGRADLVAAYAAPLP

IAAICDLLGVPATHRIDFTAWTDALIAPDPARPHLAKEAVVAMLGFYTGL

VADKRRQPGEDLLSDLIAARDEGDRLSEDELTSLAFLLHFAGYENAVQLI

GNAALALLRHPEQLAALRADPARLPGAVEEFMRYDTSAVFSIRRFAREDL

TIGGARVAAGETVFLGLGSAGRDPGRFPDPDRLDIGRDASGHLALGHGIH

HCVGAPLARLELTIAIGALLERFPDLALGVPEEELRWRPSIRARGLLALP

VVF

>CYP107ND1(2546765863)Streptomyces aurantiacus JA 4570

MMTTLMDSKSAGFKFSAKLGATRAILRAFGVFGDPFTRLLQGQEDPYPLY

AKVRQRGRIHRSHLGTWVTGSHELGSRVLRDRSLFLNREQGGVPGHEMMD

EIPWNSSILGLDPPDHTRLRRLAQPAFGPRMISKYRIQVEKLCHGLLDQM

SARDGFDLIEDFAGPLPLLVIGELLGVPEEYHDRFIRAGRKVGPVIDGVK

SLKMAREFRGAVDDLDQVFTELIALRAEDPGEDLISRLTVANGEGKLTTE

ELLAFCTVLAVTGFETTTNLIGNAVLAMTGDRAQWDLLREDPDLAPRAVE

ETLRYDSPALQAQRVPHQDIALEGHHIRANSSVVVLIGAANRDPEVYEEP

DRFDLTRENPAEHLSFSGGIHYCIGAPLARMEGETALRVLAERLPDLRVA

GPVRRRSSPVISGCTRLPVTGKAG

>CYP154D(2546765877)Streptomyces aurantiacus JA 4570

MDPAGGCPHADNARLLAENAVAEVVLPGEVRGMAVLGHEALKEFLSHPDV

AKGAEHFTALREGEIAEGWPLRTFATVRGMTTADGADHRRLRSLVSKAFS

PRRVEALRPRIETLTAELLDDLAEAAAAGDGIADLRSHFALPLPMGVICE

LLGVDAVYQDRLHRLSNQVVSTSTGPEEAIAANRDMYEVLAAVTATRSEK

PGDDLTSALIAARAEDGDRLGPQELIGTLLLMIVAGHETTLNLITNAVRA

LCAHRDQLELVRTGEAGWPDVVEETLRWDSPVSFFPFRYPTRDLTLDGTP

IPKGTPVLAGYSAAGRDPAAYGPDAALFDVTRPARHLSLGHGAHFCLGAP

LARMEATIALERLFTRFPGLDLAVPESELPRQAGFVGNSVARLPVRPRG

>CYP107L(2546766200)Streptomyces aurantiacus JA 4570

MAESVDLAAYGPRFTTDPHAVYADLRALGPVHRVRFPPPDDDGDVHLIVG

YEEARAALADPRLVKSASAVGVRFPDEELIGTHLLMADPPQHTRLRKLIA

REFTARRVEALRPRIQQITDGLLDEMLAAGDRADLVEALAFPLPITVICE

LLGVPDMDRAAFRAMSNAAVAPPTTEVWQSALADITAYLDELIETKRAAA

PGDDLLGALIRTTAEDGDRLSARELRAMAFLLLIAGHETTVGLISNAVHA

LLTHPGQLAELRADMSLLDGAVEETLRHEGPVENATYRFAAEPVEIGGTV

IPAGSAVIVGLAAADRDPDRFPAPDDFDIHRAPQGHVAFGHGIHFCLGAP

LARLEGKVALGSLLERCPRLALDGAPPKWQPGMLMRGMRHLGVRW

>CYP154B(2546767212)Streptomyces aurantiacus JA 4570

MLDVRGRDQAGEAAVLRGRGAAVEVELPGGVRAWAVVRQRYLRQLLVDER

VSKDARLHWPAFAAGQITRQWPLYPWVALENMLTTHGERRARLRRLVLGA

FTARRIEALRPRLEQETARLVGDLAARPAGQALDLRADFAQVLPIRAISG

LLGVGERSEKVLCAALDVGFSSACSAGQMSAAMAQISEVLTDLVAAKRAA

PGADVTSALLQVRDRGEALSEAELLDTLQLLLAAGLETLTTFITNAIAAL

LIDPRQLEHVRCGRAGWDDVLAETLRTRAPAAFMPLRYAVTDIELDDGTL

IKKGDAIIVSFAAACLDPEAYGKDAAVFDVLRTTGRDNLAFGHGAHYCLG

APMARLETTIALRALFGRFPRMRLAVPPRDLQPVPSFIVNGYQRLPVLLG

PPAR

>CYP1064A(2546767685)Streptomyces aurantiacus JA 4570

MRIPGPEPRTDGGVGAVTEAGGLHHYQLRLHAEYGPVVRFQLPGAETAVS

VADPVLLEATAHIDKRPERLFEFLAPLCEAGNLQVIAADEHTPWRRVLLS

VLAGRPSHERHFARFTELATALADDWATRADGGPAEPGGEPVELQKDLTA

LTLRMISEYALGGAGDLADPDRVIGAFEDVLTEYLGRLYQVPVPGTQDDR

ARRAEDALAFLRATVDRVVAAHRSGGRTDKSDLIGALVEAGESPARIRDT

VMVTMLAAHHTTGVAVSWTLHLLGRHPEAADRVAAELDRVLGERPAPEYA

DLRRLTYLDMALKESMRLYPPGPYGARETTEALVLGDYEIPAGTTVFYPF

WAVHLNPAYWPEPEKFVPERFTPEETAKRPRLAYIPFGLGPRSCEGAALA

MIEAELVLAVLLKRFRFRPAPGHETVTPIERFVLWAEDDIRMLVSPRRPA

>CYP152D(2546767694)Streptomyces aurantiacus JA 4570

MVDNTLPFLTQGYAWLPDLSRRKGPGPVRTRLLGKPAIALRGPAAVAFFY

DENHVRRRTALPEPVLSTLFGKGAVHTLDGPEHRRRKALFVSLLKDASGV

AMLARLVAEEWERSSKEWTGRPQVTLFDEVSVLITRAVCAWAGVPLGDRP

DDEARRTARDLVAMVDGFATAGPRHWQARRARRRQEKRLARLVEEIRSAG

GDAPADSDRPASAVQAVAAHRDADGELLDPHTAAVEILNVIRPTAAITWY

AVFGAHALHRNPGLRERLATDSEGYARAFAHELRRFYPFAPFVAGLAPDD

VQWRGEAIPEGTLVLLDLYGQNHDPELWHDPYMFDPDRFLGREPGRDELV

PQGGGEAAEGHRCPGEDITLAVLSTLLPRLARLQYQVPEQDLRIPLSRMP

TGPRSGFVISEVH

>CYP170B(2546767827)Streptomyces aurantiacus JA 4570

MTVESAASAIPAPAAPERLRIPPVVQGGAPLLGHAWNLVRDPLGFLAALR

DHGDLVRIRLGPKTAYAVCDPRLVGAMLKSTDYVVGGPLWDTLEVLLGKG

VATSNGPLHRRQRRMMQPAFRPERIADYARVMEEEAQATAARWDHGSTVD

VSAEMFRTAVRIVSRSLLEVESIGEKADRISDSLHTVFEGLYRRMVLSVG

PLYRVPTPANRRFARALADLHALVDEIVAERRARGNGSEQDLLAVLLRAR

DESGQPLTDQEIHDHVVSLVVAGAENVASTLGWAFHLLTEHPDQERRLVE

EVNSVTGGRPVVFADLMELRHTRNVVTEAMRIRPAAWIFTRRSVAETSLG

GYRIPADADIVYSVYAMQRDPRSFDRHLEFDPDRWNPERAASVPEFAMMP

FSVGNRKCPGDHFSLAELTIILATVLPKWRLSPVERTDTGTKVGITLHPK

KLVLRAERR

>CYP105D(2546768016)Streptomyces aurantiacus JA 4570

MAQATPFPQDRTCPYHPPTGYRPLSEQGPLNRVTLYDDTEAWLVTGHAEA

QALLTDPRLSADRQNPAFPLIARRFELSRREPIALLGVDDPLHNKQRRTL

IPTFGVKRVAAMRPRIQRVVDELLDAVVAQGPPVELVAAFALPVPSIVIC

ELLGVPYADHDFFEDASRRILRSATAQESDAGRLELMDYLGDLVDRKAKA

AHGDPAGARAAAPAADATPEGVLDELVRDRLSDGTLDREELVRLALILLV

AGHETTANMISLGTFTLLEHPDQLGALRADPELTRDAVEELLRYLSIADG

LVRVATEDIEVAGQTIRADDGVILSSATVNRDATVYSDADDLDVRRNARH

HLAFGFGIHQCLGQNLARAELEIALHSLLTRLPDLRLAVPAHELRMKPGD

TIQGLVELPVTWGRG

>CYP125A(2546768704)Streptomyces aurantiacus JA 4570

MSCPALPDGFDFTDPDVLQDRVPFPEFTRLRQSEPVRWIAQRPGISGFDD

AGYWAVTRHADVKYVSTRPELFSSNLNTAVIRFNESISRDQIEVQKLIML

NMDPPEHTRVRQIVQRGFTPRAIRSLEDALRSRSHRIVETALESADENGS

FDFVTRVAVELPLQAIAELIGVPQEDRSKIFDWSNKMAAYDDPEYAITEE

VGAEAAMEIVSYAMNLAAARKECPAKDIVSRLVAAEDEGNLSSDEFGFFV

ILLAVAGNETTRNAITHGMHAFLTHPDQWELFKRERPVTTAEEIVRWATP

VVSFQRTATEDTELGDARIKKGDRVGIFYSSANHDPEVFDNPDVFDISRD

PNPHLGFGGGGPHFCLGKSLAVLEINLIFNAVAEAMPGLRLTGDPRRLRS

AWLNGVKELQVSTR

>CYP180A(2546769156)Streptomyces aurantiacus JA 4570

MTAPSGAPGASGAPNVPGVPGVPGVPDVFDPRLYAEDVPYDRYRTLRDHH

PVARQEEPPVLGWPAGPGFWAVTRHADVTAVLKDTDTYSSYLGATQIRDP

DPADLPFIRRMMLNQDPPWHGRLRTLVSRAFTPRRVARFEALARERARAL

LARAVDEARAGDGTCDLVATVTDDYALLNLADLLGVPKSDRGLLLHWTQR

VIGYQDPDEAGEPVLGPDGRPVNPRSPAMLADMFAYAAELAAHKRRHPGD

DILTTLATTPELSTAELEMFFFLLTVAGNDTVRGAAPGGVLALAGHPGEL

RRLREGTAPVAVAVEELLRWHPPVLSFRRTAARDTELAGRRIRRGDKVVV

FHAAANRDERVFADPGRLDLTRDPNPHVSFGGGPHVCLGAHFARLQLAAF

YEELLDAMPNPRLAAPPRRLVSHFINGLKTLPLRVMD

>CYP102B(2546769271)Streptomyces aurantiacus JA 4570

MAAMAQTTQPTGSAEPGPGDLPKGFRGAEAGWPQLHRIPHPPRRVPLVGD

VLGVRLRTPVQDSMRIGRRLGPVFRRKAFGKEIVFVWGARLAGELADETR

FAKHVGLGVANLRPVAGDGLFTAYNHEPNWQLAHDILAPGFSRDAMAGYH

PLMLDVAGQLMAHWDGAAAAGRAVDVPGDMTKLTLETIARTGFGHDFGSF

RRDRPHPFVTAMVGTLSHAQRRNVMPPALAPLLLRGAERRNAADIAYLNR

TVDEVVAARRAADASGARGDLLDRMLDVAHPDTGERLSPENIRRQVITFL

VAGHETTSGALSFALHYLARHPDVLTRAREEVDQVWGRAQTPGYEQVAKL

RYVRRVLDESLRLWPTAPAFAREARQDTVLGGVHPVRRGAWALVLAVLLH

RDPEAWGARPEEFDPDRFAPAAVRARPPHVFKPFGTGARACIGRQFALHE

ATLVLALLLRRYDLVPEPGYRLRVAERLTLMPEGLRLRLIRRSG

>CYP107U(2546769430)Streptomyces aurantiacus JA 4570

MHDQPPPACPHSTPQPPAPDLFTWEFATNPYPAYAWLREHAPVHKTTLPS

GVEAWLVTRYADARQALADQRLSKNPAHHDEPAHAKGKTGIPGERKAELM

THLLNIDPPDHTRLRRLVSKAFTPRRVAEFAPRVQELTDQLIDTLLDKHS

ADKGEADLIHEFAFPLPIYAICDLLGVPREDQDDFRDWAGMMIRHGGGPR

GGVARSVKKMRGYLAELIHRKRAEPGDDLISGLIRASDHGEHLTENEAAA

MAFILLFAGFETTVNLIGNGTYALLTHPEQRAELQRSLAAGHTGLLETGI

EELLRFDGPVELATWRFATESLRIGGQDIATGDPVLVVLAAADRDPERFE

RPDLLDLSRRDNPHLGYGHGIHYCLGAPLARLEGRTALATLLRRLPDLQL

AAEPEELRWRGGLIMRGLRTLPVTFTPPGR

>CYP294A3(2546769552)Streptomyces aurantiacus JA 4570

MGPLPEFFAPGEEDVKRIVTPSGDKMWLVRDYALGRLVLADPRFSRAAAV

GPDAPTYNDAQPVPESMMSMDGSEHARLRKTVTGAFTARKMAAMAPGIEK

LTDRYLDALEAAGPGADIMEHLGTPLPLDVLCQLLGVPLEDSERFRGWVE

VLFDISASTPQEKGRKRLELSAYMADLLAAKRGAPQDDLLSSLIAAQDDG

RLSPGELITLGLTLLMAGYGTTVGQIALNLHVLLSEPGAYQELVDHPERV

DATVEELMRLSPTTPLSFSRVATEPVRLGSALVRAGDAVVVSLLNGNRDG

KVYPDPEFLEPEGRDPVHLTFGHGLHRCLGAPLGRIQLQIVFARLVGRFP

GLRFADLPEPAVWKDGMGTRGFARIHVDW

>CYP107X(645391067)Streptomyces hygroscopicus ATCC 53653

MPHTSATSETAVDTAALIEDLYPALAALREAGPVHRIAGTDGRPAWLVTR

YDDVRRAFADPRLSLDKRHAAPGNYSGFSLPPALDTNLLNMDPPDHTRVR

RLVVKAFTPGRVEKLREPVRRVAGELLDAIEAEGRADLLAAYAGQLPIIV

ICDLLGVPEGDRRDFRAWSDALITPDPARPQGAKEAVGAMLRFYTGLIAK

KRAEPGDDLLSDLIRVRDDETDGGADRLSEDELTSLAFLILLAGYENTVH

LIANSVLSLLDHPELLKELREDPARIPAAFDELARYEAPAPLAIRRFPRE

DIEIGGVTIPAGETVLLSVASAHRDPAHFQDPDALNPHIGRSGHLALGHG

IHYCLGAPLARMETDIALATLLSRFPGLRLEVPREELRWRPTIRARGLIS

LPVTW

>CYP161A(645391121)Streptomyces hygroscopicus ATCC 53653

MPTSRELPALTAEARPVLDPSPLRELRASAPVCRVRTPAGDEAWLVTRHA

EVKKLLHDERLGRSHPSPETAPRYVKNSLLDLLVADDPQVAREAHARTRA

LFTPNFSAKRIRALRPRIEEVASDLVEDFAARERPVDLHAYFSSPFSLRI

LCELIGVPDEGRERCAALLAGMGQIDTGQSMVTGPKALFGLLAGVSARKR

AEPGDDVISRMCAAGLPDDHVGSLAAVLLFAGLESVATHIDLGVVLLSAH

PEQRDAVLRDPALLTGTVEEVLRSAKRAGATLPRYASEDIEIAGVTIRAG

ELVLLDFALANFDEQAFSEPELFDITRSPNQHLTFGHGIWHCVGAPLARV

ELSTAFTTLFSRLPGLRPAVPLDELRTRGGELVGGLAELPVTW

>CYP105H(645391122)Streptomyces hygroscopicus ATCC 53653

MSDAAKLVDFPLRRPGDPLPPPQYEDFREREGLVWSTMPTGARVWLVTRY

EDVRAVLTNPKISSDPGHEGFPSPGRTGGPPAQDQVPGWFAALDPPEHDR

YRKVLIPEFSVRRIRELRPRIQEVVDDCVEALLAKGSPADLASDFAVPIP

SLVICALLGVPQADRGFFESRIRVLVTLSATDEERDEASKQILRYFSRMI

AIRKRRPGDDLISVMIKSETMTPMEIGGAAMLLVIAGQETTANNIALGAS

TLLSNRQWIGDDRVVEELLRYYSVADLVPLRVALEDVEIAGQLIKAGEGI

APLVAAANHDGSVFSCPHQFDPGRSEQHHIAFGFGRHLCLGAHLVRIEME

LAYRTLFERIPTLELARPVEELSFRNNGVLFGLDSLPVRW

>CYP102G(645391188)Streptomyces hygroscopicus ATCC 53653

MTKTSLRGIEPIPQRPALPLVGHAFSVPSGADGLLHVMKEAKELGPLFRL

RIFGNDINFVSGLDLVTELADETRFRKNVHPDLVVLRAIGGDGLFTAFND

EPNWRKAHDILMPAFSLGAMRGYHATMLKVARELIGKWDRAAGTEPVDVA

ADMTRLTFDTIGLCGFGYDFESFGRDETHPFVTSLSRALGFAQSKGESIP

GTEVFKWRQAEQFRGDVTLMQDLVDDVIRQRRASGDQSTDDLLGRMLHTR

DAGTGEPLDDVNIRHQAITFLIAGHETTSGALSFALYYLTKHPEVLARAQ

AEVDALWGDTDAPEPDYGDIGKLTYIRQVLNEGLRLWPTAPVYAVEPLED

TVIGGKYAVRKGESLLVLIPQLHRDPAWGENVELFDPDRFRPEREEARPV

HLFKPFGNGERACIGRQFALHEATLVLALLVHRYRLIDHTNYQLKIKQSL

TLKPDEFTLDLVRRTAAEWRRPAAVTSAPAALERPAARRNTGTALTLLHG

SNLGTCAGITRDLAEDGGERGFTATATPLNEAVGKLTPGAGPVVIVAASY

NGRPTDDATEFVAWLEGLEPGSLDGVQYAVLGVGDRNWAATYQRIPTLID

ERLTAAGAVRLLERGAADASGDFAGTVDRWTGDLWTVLFDRYGAEGEAAA

VETEADHDAGLYELQDTTDSVIGELAARHGVQSMEVLEAYELVVMDHPLG

RSKRFVRLRLPDGVTYRTGDHLAVLPRNPDDLVQRVADRFGLDLDRTVRL

CARRRSRNILPVDRPLPLRRLLTDFVELQDAATQEQVAVLAEHTACPPEK

RPLAELAAVEPEAFREQVTVAGRSVLDLLERFRACELPFERFLELLPVLR

PRHYSISSSAQAAPGEVDLMVSLLAAPHRGGEGTFNGIASHYIQTVRAGD

TVQARVLPCSESFRLPEDPSVPVILVSAGTGLAPFRGAVLDRKHAGATGT

MLCYFGCDHPDVDYLHHEEFAPAEAAGAVSMRPTFACAPEDGARFVQDRI

AKESDEVWAALEAGGRVYICGDGRRMAPAVREAFMAIYRKYTGADDEEAA

AWLAVLIESGTYVEDVWAG

>CYP159A(645391246)Streptomyces hygroscopicus ATCC 53653

MTTTPSTAPDILSPEFAADPYPAYRAMRQDFPLIHHPATDAYIISRYEDV

ARAFKDPVFTSHNYDWQIEPVHGRTILQMDGREHSVRRALVAPAFRGKDL

REKFLPVIERNSRELIDAFRDEPEADLVGQYATRFPINVIVDMLGLNRAD

HGRFHIWYTSMIDFLGNLSQDPEVAAAGLRTREELAAYMIPVIQDRRAHP

GDDLLSVLCTAEIDGTRMSDEDIKAFVSLLLAAGGETTDKAIAGLFRNLL

AHPEQLAAVREDRTLIPAAFAETLRFTPPVHMIFRQPCEDVEVSGGTIPA

GATVTCLIGAANRDGSRYADPDTFNIFREDLTTGSAFSAAADHLAFALGR

HFCVGALLAKTEIEVGVNHLLDAFPTMAFADGEVPTETGVFTRGPGSLRV

RF

>CYP157C(645391448)Streptomyces hygroscopicus ATCC 53653

MVDLFGSENEKDPMGLYERLRSEHGPVAPVRLGDDVPAWLLLGYRENLEV

ARTPSRFSRDSRYWSAWGEGRIPDDSPLLPVVGWQPMCTFADGDEHERLR

AALTDSMNRVDRRGIRRHITRFTHQLVNDFCADGQAELVTGFAQRLPMLV

LTQLLGMPDEYGPRLVEATRDLMKGSETALRSDQYVTDTLRQLVERKRTQ

PGSDLASWLLSHPSELTEEEVVQHLRLVLLTGNETTTNLMANTLRMVLTD

PRFRASLAGGHMTLPDAIEHVLWNEPPLTVIPGRWATGDTELGGRQIKAG

DMLLLGLAAGNVDPAIRPDLGAPMYGNRSHLSFSGGPHECPGQDIGRAIA

DTAIDTLLLRLPDLRLDVPETELNWIASWISRHLVALPVAFMPRRAETDP

VDTSGMLLAPKPRPTRWVPADEDEALSAGEDATDGTAAPRARVPWWTSLW

RWLRGTAA

>CYP105B(645391688)Streptomyces hygroscopicus ATCC 53653

MTLAEFPMARAAGCPFDPPPALRETMEEGPLARVRLWDGSTPWLVTRHAE

QRTVLGDPRVSADITRPGYPSSAPLPKGGTAISFILMDDPEHARLRRMVT

APFAIKRVAAMRPAVQKIVDDLIDELLAGPTPVDLVQAFALPVPSLVICE

LLGVPYADHDFFQDNSKVLINREVTPEQRQAAHGALLGYLDGLMGEKIAH

PVDDLLSGLARRITAGEMTREEAAQMGVLLLLAGHETTANMIALGTLALL

ENPGQLALLRESDDPKLVASAVEELLRYLNITHSGRRRVALADIEVAGQV

IRAGEGIILANDIANRDPEVFPDPDRLDLRRDARRHVAFGFGVHQCLGQP

LARMELQVVYSTLYRRIPTLRLATDLEKIPFKHDGSVYGVYELPVAW

>CYP154Q(645392687)Streptomyces hygroscopicus ATCC 53653

MSAELTTGCPFRLDPTAGDIHAEADRLRDRGPATPVELPGGVVAWSVTDP

ALVKRLLTDRRISKDAHRHWPDSYIDERIPPKWQLRIWMDVRNALTAYGP

EHVRLRRLVGAAFTPGRVRALAPRIEGITQSLLDELDATGTTAPGVPVDL

RSRFAWLLPLMVVNTLLGVPDELHNAFRDGIAGVFATGLTEEEAEANTRA

LYGLLTELLAIKRKAPGDDVTSVLIDAHDDETDSRLSEQELADSVLLLIG

AGHETTVNLLDHAIVNLLTHPDQLALLRSGQASWGDAVEETLRHQAPIAS

IIMRFPIEDVHDPATGLTFREGDPIVINYAAANRDPQAHGEHRGRFDLTR

ATRREHLAFGYGPHFCLGAELARLEGRIGLRALFERFPDLALAAPAGELR

PLESFISNGHQQLPVLLRR

>CYP107E(645392705)Streptomyces hygroscopicus ATCC 53653

MTSAQPLTYPFQAEKLNLAPLYAQLRHEEPLAWVQLPYGEPGWLATRYED

ARLVLGDPRFSRAASVGRDSPRVRPYAPGPGTISTFDPPEHSRLRRLVTK

AFTVRQIDRLRPRVQQLSDDLVDAIRAEGASADLVEDFALPLPITVICEL

LGVPFEDRADFRLWSEAFLSTTKYTLEEIKGYRALLRDYMAGLAEQRRTA

PQDDLLSSLVAARENDDRLSEDELLSLSEAILIAGHETTATQLGNFFYVL

LTQPDHLAALRADLSLVPQAVEELLRFTPLGSGSMQPRYALEDIELGGVT

VRAGEPVVVAINSANRDEGVFTDSDHLDLRRREATHLGFGHGPHHCLGAP

LARMELQIALRTLLERLPGLRLADAERDVEWKVGVSTRGVRRLPVTWEA

>CYP157A(645392853)Streptomyces hygroscopicus ATCC 53653

MTAQPRPSENAAATPATPPPGCPAHQGARSLSGPGFQTDPFGVYGEMRGE

HGPVAPVLLDGDIPGWLVLGYRELHQVTSDAELFTRDANQWNQWPNVPEN

WPLAPMISRDQTSVVYATGEDHQRRAEAIGEALEAVDPYELRSQAELIAD

RLVDEFCHEGEAELIADYARLLPELVLARIYGIPDAEGPALVQAMNDAID

GKENALESYQHVYLVMRQLASAKRTAPAPDVASRLVAAYRGYSDELLVQD

FIVIMTAGHQPTADWIGNTLRLLLTDERFALSLSGGRHSVGQAMNEVLWE

DTPVANLAARWATRDTTLAGKHIRKGDLLILGFHGANTDPLVRPDSDAFT

EGNSAFLSFGHGEHRCPHPAQEIAETIAKTAVEVLLDRLPDVRLAVPSDT

LLWRPSAWVRGLSALPVAFTPAPKGAR

>CYP107BW(645393083)Streptomyces hygroscopicus ATCC 53653

MTDTVPDYPFEQPNVLEPPREWAETRAACPVAHVRMPSGDVVGLLTGYEE

VRGLLTDQRFSRNLDREGAARMSTTEDGGMFSRPSQGQVDNKEGPGHRRW

RRLISRAFTVKKMEAWRPRVQEMADALVDDMVAKGSPADLRAALALPLPV

RVICALLGAPSEDQDKFARWSEVLLTLTRYTQAEVDEAQ

>CYP1940A1(645393092)Streptomyces hygroscopicus ATCC 53653

MTAVSASGRRGDARSLPARAGIFWDPRTPVHFDEALNTWNVFSRADVLRV

LNDRETFSAGYGLSEEDRLRAHPSLSGMWAAEGARHDDLRAVVAEPFRRT

VLDSLAREVRDMVSELLDDVVAVGTGRIEVVGALAEALPSRVICRVLGLE

LSYADRMRTWVEEMSRAASATSELPPQPDLVRFFQELIEARRARPGSGLV

DDLIAAQRSGCPVAGRPMTEHDLVGYFAMMLSAGVDTTATSIGNAFLFLT

EYGCWERLGEQPELVPHAIEETLRWYPAFPGVRRHVLADTAIGGQHVRAG

QWVTGWLTSANRDPDKFPDPGRFDMHRRPNPHVSLGNGRHHCLGAPLARL

ELRILIEEAAARLPGLRRDTGEPLTRSQWIVDPLKSLQMTFDTPDPEESG

ILER

>CYP156C(645393132)Streptomyces hygroscopicus ATCC 53653

MSGSAGPLGHGARVPMYGADFAADPHGIYARMRAAGPITPVELAPGVDAF

LVTGYSTALEVLRDTERFAKDPRHWAALTEGRLPADSPVLPMMEFRPNAL

FTDGEQHARYRGAITDSLSRVDSHALSDYVERSADTLIDSFVEQGEAELL

SEYAGIIPLMVFNRLFGAPPEQSSELIRILTIMFDASSAETGQANEDLSR

YMADLVDAKRQKPGADVTSWMIAHPSRLADDELMQQIALLMAAGTEPQVN

LIGNALRLLLSDDRFAGELSGGSLPVQDALDEVLWADPPLANFGTRFARY

DVEMGGVRLRKGDVVLTSYAAANTDPVLTSQGRAGNRAHLAWSVGAHRCP

AEGPARVIASVAIERLLDRLPDMQLAVSSDQLTWRPGPFNRALASLPVRF

PVLTKPAPEPASMPPAAAVPAQRQPAEPQGDNQWSTSPVPSSSTPPAATS

TPKPTASEAKGPRRLWGFLGAWWRGQ

>CYP157G(645394126)Streptomyces hygroscopicus ATCC 53653

MRLAVPAVALIHRSKEDLVTNLQDPQDIPDPQDTPDGTGSVVPPPGCPAH

GGASLLYGPRFLRDPTESYQRMRRDHGQVAPVLLEADIPAWLVLGYRETR

QILGDTQTYGRDPRRWNRWDEVPPDWSLMPWVAYSPMIHYTEGDEHRRRS

AAVSDALSIVDPFELRAHCERFADGLIDKFAGSGKADLVFDYVYSVPALA

MGTMFGLPEDQVEDLAQALTASLDANEDAIAAQQRAAEIVARLVAAKRDA

PGLDITSRFVQNSPDLTEEQLVGDIMVMMVAGLPATSYWIGTTIRLMLTD

TRFAVTLAGGRRSIGEAMTEVLWADSPVPNVIGRFAIRDTVLGGQRIRAG

DLLVLSLAAANMDPLLWPDTTAGFAGNNAYLSFTGGDYGCPAGAPELAKN

IAEMAIEVLVDRVPDLTLAVEPEELEWVDSLWCRAPSSLPVTFTPTHINA

G

>CYP154R2(645394420)Streptomyces hygroscopicus ATCC 53653

MGLTEDDRPVLLDPTIRTSAEESELLYAAGPVVRVELPGGVPAWAVTHDA

PAREVMRDSRFVKDINRWGAWKRGEIPADWPLIGLVDLGPSLVSTDGTEH

RRLRRPVAREFTPQRVEALRGRVEEITRELLDRTERAADDAGGVVDLKDT

FAFPLPITVVGELFGMPTEWHRPLRELYDSFFDDKAAPETVLATLQGLYG

YIARLVRLKRDEPGADLTSALLSELGDEPSEEAVEELIATIQVMITAGHE

TSVHLIVNAVRSLLGDPAQLELVHTGRYSWDDVVEESLRLDSPTLNFLFR

FATEDIDIAGTLVREGEGVVISYGGMGRDRGQHGADADRFDISRRPRHTS

FGHGPHSCPGSALARLETTIALRELFARFPGLRLADPDGPPERSPSVIIN

GQRTLPVRLG

>CYP157H2(645394421)Streptomyces hygroscopicus ATCC 53653

MTQPTSPSEPSGEPGAPDEPGLSGDPGAPTAPEPTAPEPTAPEPTAPEPT

APEPTAPQAAPAIERPASHEDAVELFGPRFQDTPAEVYRELRNTYGPVAP

VLLPGGVPAWLVLGYRELLRVTENTKVFGRGSARWKLWPQIPPDWPLRPM

VEDNGSLLYREGDDHRRRAGAVGSALEAVDLNQMQDATQSFAQGLIDKLS

EGSRADLIADYAHPLPVLALSRVLGLSDAEGVALVKAINDMVDGGPDALR

GQIEVRGTMERLVARKRADPGPDATSRMIADRAGLTDQEVVEDLMVLTLA

GHQPCTDWIGMTLWLLLTDHDYAADMGGGRRSVRQAMHQVLRDHTPVQIF

AGRFTTQDTVLGATRIPAGDLVLLGLAGANTDPYIRPQAGGVAGPEGHNA

YMSYSHGEHRCPYAGQWIAEVVAHTAVETLLEHLPDVELAVPADELRWRP

SPWLRGLTSLPVTYTPAHSYTGGFGWD

>CYP107P(645394939)Streptomyces hygroscopicus ATCC 53653

MRRYGPVMTSPAPAFDPWQPSFVADPYPAYASLREQGRAHWFAPSRQWLI

PHFEDVRALLTDRRLGRTYLHRFTHEEFGRTAPPPEHEPFHTLNGNGMLD

LEAPDHTRLRRLVSKAFTPRTVQGLVPTIRRLAAGLAESFAASGGGDLIA

AVAEPLPVEVIAEMLGIPHADRPALRPWSADICGMFELNPGEETARRAVR

ASEEFSAYLRGLIAARRKDPGEDLISALIAAYDEGDRLSEQEMVSTCVLL

LNAGHEATVNTTGNGWWTLFRHPDQLARLRDAPDALLSTAVDELLRYDTP

LQLFERWVLEDIRVGDTLIPRGSEVALLFGSANRDPARFERPDTLDLART

DNPHLSFGAGIHYCLGAPLARIELAASFGALLRAAPGLRLVREPEWRPGY

VIRGLRELLVEV

>CYP157B(645395081)Streptomyces hygroscopicus ATCC 53653

MTTPDPMPPLEDPFAPAAPPLLGWPEQPDAVRLDMQLLRTDPDELYRKLR

REHGPVVPVLLEPDIPVWMVLGYRELLRVTTDPNLFARDSRRWHSWDRVP

PDWELMTFVAYRPTMLFSEGADHERRSEAIIDSMESVDPSELRAQCERVA

DSLIDVFAGSGRADLMAEYASQIPLRVLNRMYGLDDSPEIVQETVDLVSG

NGGPEAHQNLLNRMVALLENARSNPGANNIASVLAFHPAGLTDEEILNDL

LGNVYAAHQPTTDLIGNALRLMLTDERFAVTMAGGRRSVGQALNEVLWEA

TPVPNWVGRWASEDTMFAGRHIRKGDCMMLGLAAANADPDVRPDFRAGAA

GNQAHMAFSHGTHGCPQVARELAENIAMAAIEVLIDRLPDVTLSVTPDEL

QWRSALMMRGVSSIPVDFTPAFHR

>CYP147F(645395380)Streptomyces hygroscopicus ATCC 53653

MAHGPLLRKITDYANRADPYPIYAELRKTPVVHEEDGPYIVSTYWEIHAL

LHDPRISSDARNLDPRAAGEPDDAETEAEEDDPGLPPAFLKLDPPEHDRL

RRLAMTPFGPPHTPRRIHAMRGELSRIVDELIDGFRDRDGIDLVDDFSYP

FPVTVICRLLGVPREDEPRFHTWADTLAASLDPDPEQDAAERRRVTQQAR

IELGRYLSELIEERRRSPGDDMLSALVAGHGPEGGMSHTELLSTAALLLV

AGHETTVNLITNGMLALLRNPDVLARLRAKPHLAVPLVEELLRYDPPVQL

LPQRTPLSDIDIAGVTIPKGASVWLVLASGNRDPQRFLDPDRFDPQRRDN

QHLGFGSGIHSCFGAPLARLEAQLALTALAKRLDNPRLREDPPPYRRNAV

LRGPRHLPITLDGLR

>CYP107BM(645395411)Streptomyces hygroscopicus ATCC 53653

MSENAMSENATHLTPEQVEDPVRGYADLRTRDELPHAVLPGLTTPVRLVT

RYADVKAALTEPRLIRDRTRIPGDADEAQASDAQDELLEAASAGLPAEYV

KYFAGHLALFDGEEHAHRRAPLTRAFTARRIAALRTFVERTADALVAEAE

ANGGTDLLGAFAYPLATQVICELIGVEERDRDQVCSWMRDFAFGDGSRAG

EALVALVEYAKELIARRRAEPTEDLISALLDGDQLTEDDLIGIVFLLINT

GITPPALFLAHAALALLDHPEQAARLHAAPELLPRAVPELLRYVSIVRIG

ATLYATEDVDFAGTALRRGESVTVALLAADHDPEEFGDGAGQLDVGREFG

RGDGHLAFGHGAHYCIGAALGRLVTSVVLDRLFVRHDGLRLAVPRAELEF

GHWPGDGFHLLRLPVRL

>CYP105AK(645395440)Streptomyces hygroscopicus ATCC 53653

MSNLRSNIVTWLGRKYFTKIQKNGFDLSKMSFLPDATLAPLKRDGLDPVA

DLSKTRAKEPISKIWLPFGINGWLVTGYDETKAVLGKAKGFSSDFTNLAE

KAVGVAEQQHPGGLGFSDPPTHTRLRRLLTPEFTMRRLSRLTPRIHAIVE

ERLDEMAAAEGPVDLVEMFALPIPALVICELLGVPYEDRDDFQRYSTARF

DLFGGANASLGAMSDSLTYLHGIVRKQRESPGDGLLGMLVKEHGDEIDDQ

ELAGLADGVLTGGLETTASMLALGTLVLLQAPEQFAAVRDQEDVVDRFVD

ELLRYLSVVQVAFPRFAREDMEIAGTKISAGDLVLCSLSGANRDEKLGPD

MERVDPHRQTASHLAFGHGIHRCIGAELAKMELRAAYPALVRRFPEMRLA

VDADKLKYRKLSIVYGVDALPVTLR

>CYP156E(645396055)Streptomyces hygroscopicus ATCC 53653

MIDDPAEAGSEAAAVPLHTPEFAKDPSAVYRRLRETYGPLAPVELDPGVT

ATLCVGYDTALEILRRPETFSKDPRRWRAKNEGRVPDTSHAGQLIRYRPQ

PTAVDGEEHQRYRNAIDDSTSRIDPGALRGYVERIADSLIDQFCDAGEAD

LIGEYCALLPLLVLGELFGCPREIADRLFTGLAHLFDGTEVAEADRTITA

ALQELVALKRARPAADVTSWLIAHPERLVDDELVEQLMLLTASGSDPVIN

LIGNALRVLMTDDRFAGGLSGGSMLVEDALDEVLWTDPPLANLAVHFPRH

DVTLAGVRLREGEPVVISFAAANQDALRVSDRNRGNRGHLGFSAGPHMCP

>CYP1035A(645396070)Streptomyces hygroscopicus ATCC 53653

MHTMQRRSLGNIFDRFTLDTLEEPVSRIADTLLDRLGEQLRTTKEADFAT

TVGEEMPAATISHWLRLPESDHDLLKSLTHGQGLAQELLPSKSQLAQANE

AAEGLRTYFTEVIRERRQNLGDDVLSDWIRTWDEIEPDRALADETLYHLV

MFIVIASMETTSTLLSNMVWLLDQHPGSMGWLRSHPDRIPQAVEEVLRYD

PPIHLTTRVATEDTILSGFRISRDEVVHVMLGAANHDPAHHTDPDTFDIL
[truncated: 2,303,901 more chars]
