# Supplementary figures and images for: More P450s Are Involved in Secondary Metabolite Biosynthesis in Streptomyces Compared to Bacillus, Cyanobacteria, and Mycobacterium
Source: Int J Mol Sci. 2020 Jul 7;21(13):4814. doi: 10.3390/ijms21134814 (PMC7369989; doi:10.3390/ijms21134814)

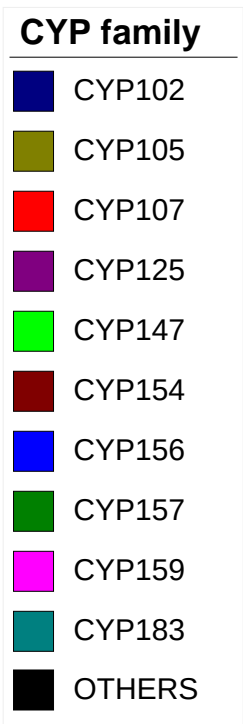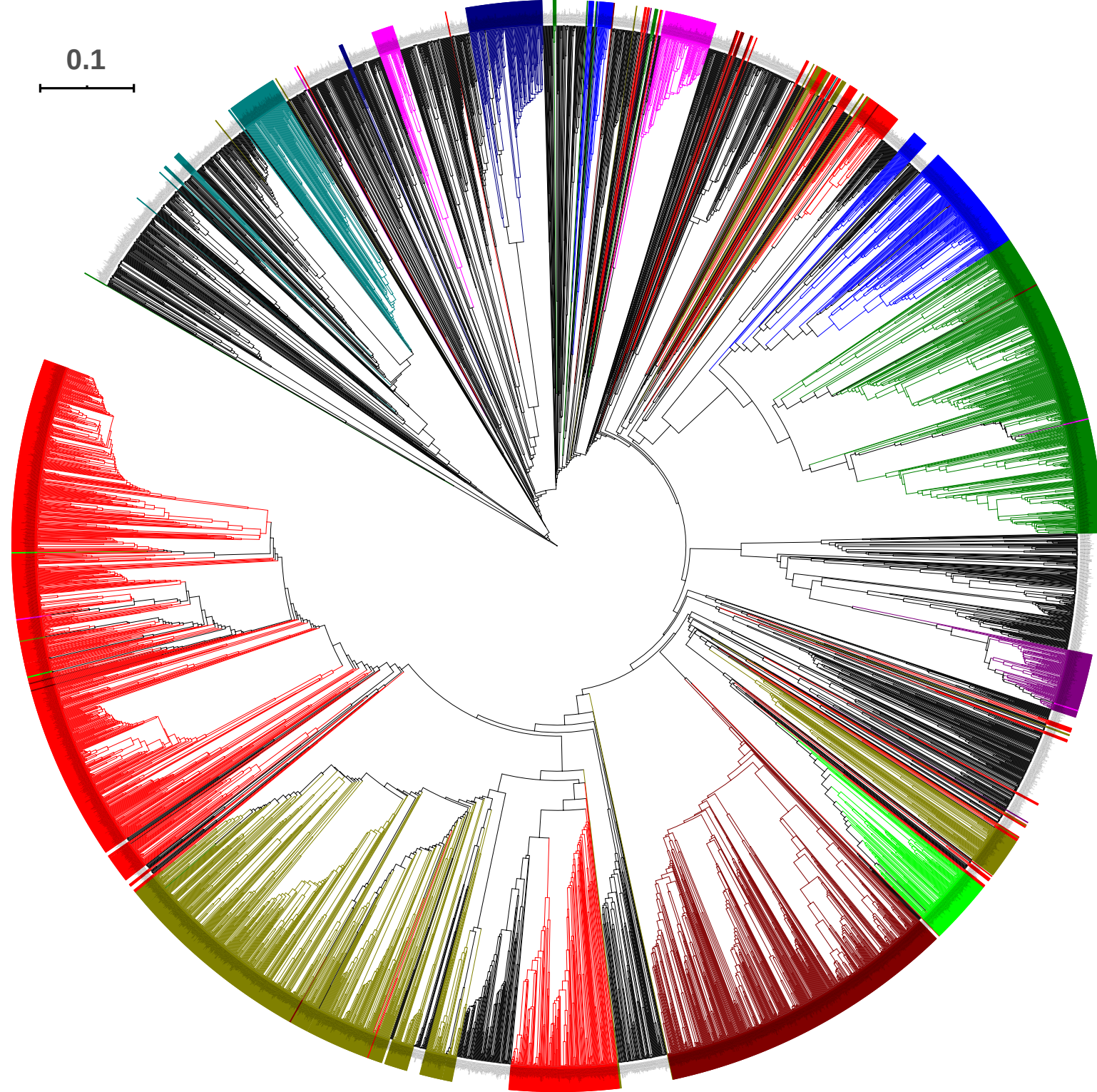

Supplement: Supplementary file 1 [file ijms-21-04814-s001.zip › Supplementary Information/Supplementary Dataset 2.pdf]
